# Upregulated type I interferon responses in asymptomatic COVID-19 infection are associated with improved clinical outcome

**DOI:** 10.1038/s41598-021-02489-4

**Published:** 2021-11-25

**Authors:** Kiran Iqbal Masood, Maliha Yameen, Javeria Ashraf, Saba Shahid, Syed Faisal Mahmood, Asghar Nasir, Nosheen Nasir, Bushra Jamil, Najia Karim Ghanchi, Iffat Khanum, Safina Abdul Razzak, Akbar Kanji, Rabia Hussain, Martin E. Rottenberg, Zahra Hasan

**Affiliations:** 1 Department of Pathology and Laboratory Medicine, The Aga Khan University, Stadium Road, P.O. Box 3500, Karachi, 75400 Pakistan; 2 Department of Medicine, AKU, Karachi, Pakistan; 3 Department of Microbiology and Tumor Cell Biology, Karolinska Institute, Solna, Sweden

**Keywords:** Computational biology and bioinformatics, Immunology, Microbiology

## Abstract

Understanding key host protective mechanisms against SARS-CoV-2 infection can help improve treatment modalities for COVID-19. We used a blood transcriptome approach to study biomarkers associated with differing severity of COVID-19, comparing severe and mild Symptomatic disease with Asymptomatic COVID-19 and uninfected Controls. There was suppression of antigen presentation but upregulation of inflammatory and viral mRNA translation associated pathways in Symptomatic as compared with Asymptomatic cases. In severe COVID-19, CD177 a neutrophil marker, was upregulated while interferon stimulated genes (ISGs) were downregulated. Asymptomatic COVID-19 cases displayed upregulation of ISGs and humoral response genes with downregulation of ICAM3 and TLR8. Compared across the COVID-19 disease spectrum, we found type I interferon (IFN) responses to be significantly upregulated (IFNAR2, IRF2BP1, IRF4, MAVS, SAMHD1, TRIM1), or downregulated (SOCS3, IRF2BP2, IRF2BPL) in Asymptomatic as compared with mild and severe COVID-19, with the dysregulation of an increasing number of ISGs associated with progressive disease. These data suggest that initial early responses against SARS-CoV-2 may be effectively controlled by ISGs. Therefore, we hypothesize that treatment with type I interferons in the early stage of COVID-19 may limit disease progression by limiting SARS-CoV-2 in the host.

## Introduction

SARS-CoV-2 infections have risen to 228 million cases worldwide with 4.7 million deaths as of mid-September, 2021^1^. The variability in disease severity of COVID-19 across the globe requires attention. Pakistan has a population of 220 million, where 67% of the population less than 30 years old and only 6% of individuals are aged greater than 60 years^2^. An estimated 1.2 million cases have been reported in Pakistan with death toll of 27,000 (as of 18 September 2021)^3^. The morbidity and mortality due to COVID-19 has been lower than in many other countries^4^ with a case fatality rate (CFR) of 2.1% with some regional variations^5^. In comparison, countries such as, Germany (pop. 83 million) had 89,592 and the United Kingdom (pop. 66.7 million) had 128,000^6^ COVID-19 related death until end May, 2021.

The first wave of COVID-19 in Pakistan occurred between March and July 2020^1^, the second from October 2020 until January 2021, the third from March to May 2021^7^ and the fourth from July until September 2021^3^. Testing capacity has been relatively limited and in January 2021, 40,000 SARS-CoV-2 PCR tests a day were conducted for approximately 200 million population (at 200 tests/million)^8^. Compare at greater than 10,000 tests per 1 million individuals conducted in a day in the USA^9^. As the majority of individuals with COVID-19 tend to be asymptomatic or with minimal symptoms they are unlikely to be diagnosed, leading to under-reporting of cases. Seroprevalence data from Karachi has shown COVID-19 sero-positivity to range between 20 and 40% in the community or hospital-based populations^10^, rising to 53% in healthy blood donors^11^.

Both pathogen and host-related factors could contribute to disparities between disease outcomes in different populations. The COVID-19 spectrum ranges from asymptomatic or, mild upper respiratory tract infection to moderate, severe and critical systemic disease. Factors such as, primary or acquired immune deficiency and comorbid conditions such as, diabetes, cardiac or kidney disease; male gender and advanced age are associated with poor disease outcomes^13^.

SARS-CoV-2 attaches via its spike protein to host cells by binding to the angiotensin-converting enzyme 2 (ACE2) receptor^14^. Acute inflammation results from SARS-CoV-2 infection of pneumocytes, causing a cytopathic effect as it spreads^15^. A ‘cytokine storm’ induced via pathways such as, inflammasome activation and modulation of host innate immune cells leads to unfavorable COVID-19 outcomes^16^. T helper and T regulatory cells are shown to be dysregulated by SARS-CoV-2 infection, affecting adaptive immunity^17^. SARS-CoV-2 also triggers IL-4 and IL-10 which are Th2 cytokines, possible regulators of inflammatory responses, but IL-10 counteracts antiviral T cell responses^18^.

Effective anti-viral responses require a combination of innate and adaptive immune responses. Innate responses driven by Natural Killer cells, monocytes and B cells are regulated by T cells, cytokines and chemokines which, recruit leucocyte sub-sets to the site of infection. Type I interferon (IFN) responses drive interferon-stimulated genes (ISGs) for timely resolution of infection, such as in protection against SARS-CoV-2 infection^19^. However, whilst SARS-CoV, Middle-East Respiratory Syndrome (MERS) virus and respiratory syncytial virus infections have all been shown to induce type I and type III interferon signaling pathways, SARS-CoV-2 may bypass the induction of type I and III interferon responses to replicate in host cells^20^. Despite the importance of type I interferons in viral clearance, there is still ambiguity regarding its role in COVID-19^19^. Compromised activation of type I IFN responses in SARS-CoV-2 patients are associated with disease severity. However, other studies have shown increased IFN activation to be associated with exacerbated COVID-19^21^.

Here we used an RNA microarray based blood transcriptome approach to study early host biomarkers associated with COVID-19. We focused our studies on a SARS-CoV-2 positive Asymptomatic (or with minimal symptoms) compared to Symptomatic individuals (with mild to severe) disease. We compared transcriptomes from COVID-19 cases with healthy uninfected controls. Our study provides insights into the role of interferon type I-driven responses associated with disease control in COVID-19.

## Results

### Demographic description of study subjects

All COVID-19 cases had a respiratory sample which was positive for SARS-CoV-2 at the time of recruitment. Blood samples were taken within 24–48 h of confirmed COVID-19 diagnosis. COVID-19 patients were classified according to the WHO ordinal score^22^. There were eighteen Asymptomatic COVID-19 cases and eleven Symptomatic COVID-19 cases. Symptomatic cases were further categorized into three with mild and eight with severe disease (Supplementary Table 1). Controls were eighteen uninfected healthy individuals. Controls and Asymptomatic cases was younger than Symptomatic COVID-19 (Table 1). However, within the Symptomatic group, ages of cases with mild disease was comparable with Controls and Asymptomatic cases.

**Table 1:** Description of study population.

Variables	Un-infected controls	Asymptomatic COVID-19#	Symptomatic COVID-19#	^a^p value
	N = 24	N = 18	N = 11	
**Gender**				
Male	9	12	10	NS
Female	15	6	2	
**Age group (years)**				
< 30	15	6	1	
30–59	9	11	4	
≥ 60	0	1	6	
Mean ± SD	32.7 ± 10.8	38 ± 12.9	60.8 ± 15.1	*p < 0.001

Variables	Un-infected controls	Asymptomatic COVID-19	Symptomatic COVID-19	^a^p value
	N = 24	N = 18	N = 11	
Mild, mean ± SD			43 ± 16.8	
Severe, mean ± SD			67.4 ± 8.9	*p < 0.001

^a^p value as compared across age groups between Controls, Asymptomatic and Symptomatic COVID-19 cases.

‘#’, all COVID-19 cases had a respiratory sample which was positive for SARS-CoV-2 by PCR within a 48 h prior to collection of blood samples; ‘*’, significantly different; NS, not statistically significant; the Kruskal–wallis test was conducted to determine non-parametric statistical comparison between groups.

Four of eighteen Asymptomatic COVID-19 cases had a positive serum IgG antibody response to Spike protein. As IgG antibodies are shown to develop 5–7 days after SARS-CoV-2 infection^23^, this further confirms that the blood samples from these individuals were taken early in the infection. All sera from Control cases were negative for IgG antibodies to Spike protein. Asymptomatic and uninfected controls were comparable in age (Table 1), but were younger than Symptomatic COVID-19 cases (p = 0.0001). There was no gender-based difference between the Asymptomatic, Symptomatic or uninfected Controls.

### Differential regulation of genes between COVID-19 cases and uninfected controls

**Figure 1:**
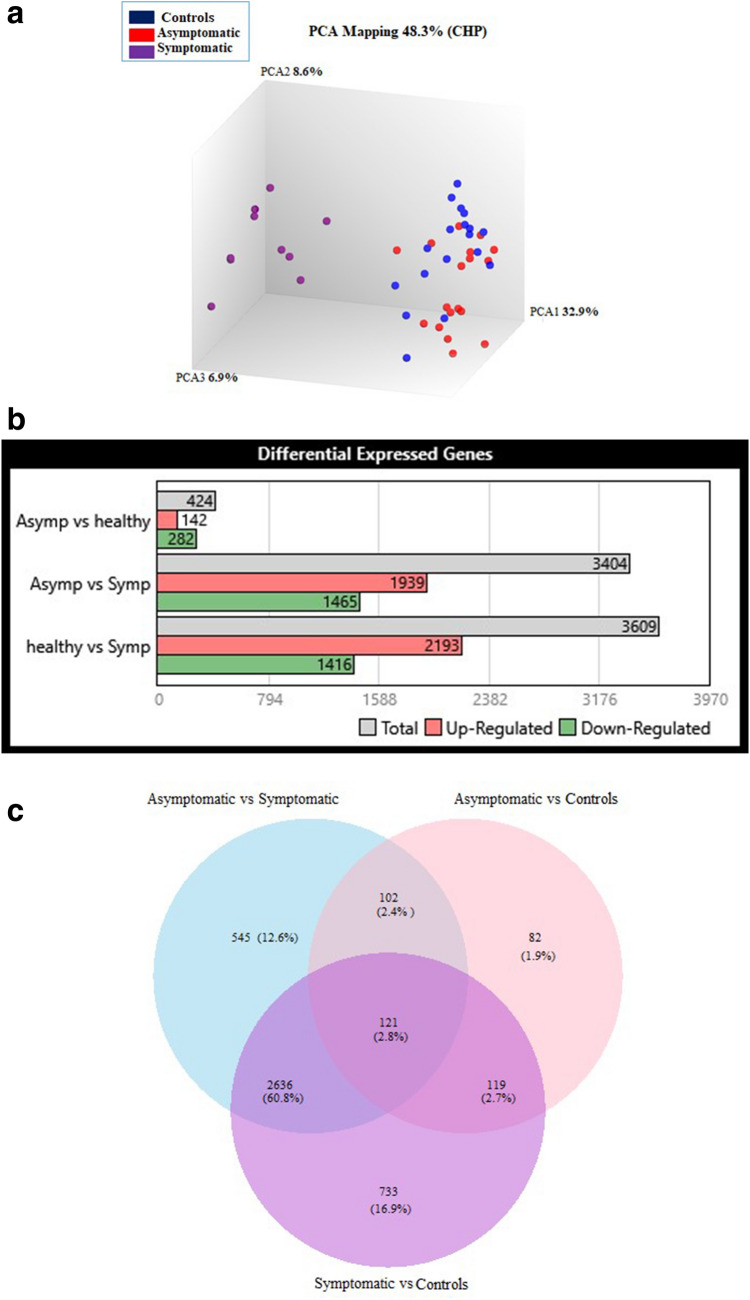
Differential transcriptional expression between COVID-19 patients and uninfected Controls. (**a**) Principal component analysis helps to visualize the clustering of datasets into three groups: Symptomatic, n = 11 (Symp = purple), Asymptomatic, n = 18 (Asymp = red) and Controls, n = 18 (blue). (**b**) A barplot depicts the hierarchical clustering of differentially expressed genes (DEGs) as Up-regulated (red), Down-regulated (green) and Total (gray) genes within three comparative groups: Asymptomatic vs Controls (Asymp vs healthy), Asymptomatic vs Symptomatic (Asymp vs Symp) and Symptomatic vs Controls (healthy vs Symp). (**c**) Venn diagram depicts the overlapping DEGs observed between the COVID-19 Asymptomatic vs Symptomatic cases (medium orchid), Asymptomatic vs Controls (pink) and Symptomatic vs Controls (purple).

We compared blood transcriptome profiles between Asymptomatic and Symptomatic COVID-19 cases and Controls to identify transcriptional differences between groups. Principal component analysis (PCA) demonstrated clustering of datasets, with Asymptomatic and Controls cases segregated together, plotted away from Symptomatic COVID-19 cases (Fig. 1a). Notably, the data points for both mild and severe Symptomatic disease clustered together, although there was an age difference between these COVID-19 cases.

A total of 4338 differentially regulated genes (DEGs) were identified between the three data sets based on a > 2-logFC difference. The greatest number of DEGs were between Symptomatics and uninfected Controls (n = 3609), followed by DEGs between Asymptomatics and Symptomatics (n = 3404), with the least between Asymptomatic and Controls (n = 424) (Fig. 1b).

Further dissection of the DEGs revealed genes that 121 (2.8%) DEGs were commonly modified between Controls and all COVID-19 cases (Asymptomatic and Symptomatic). There were 545 (12.6%) DEGs unique between Asymptomatics and Symptomatics; 733 (16.9%) DEGs unique between Symptomatics and Controls; and, 82 (1.9%) DEGs were unique between Asymptomatics and Control subjects (Fig. 1c).

### Coronavirus disease and inflammatory pathway genes are downregulated in asymptomatic COVID 19 cases

**Figure 2:**
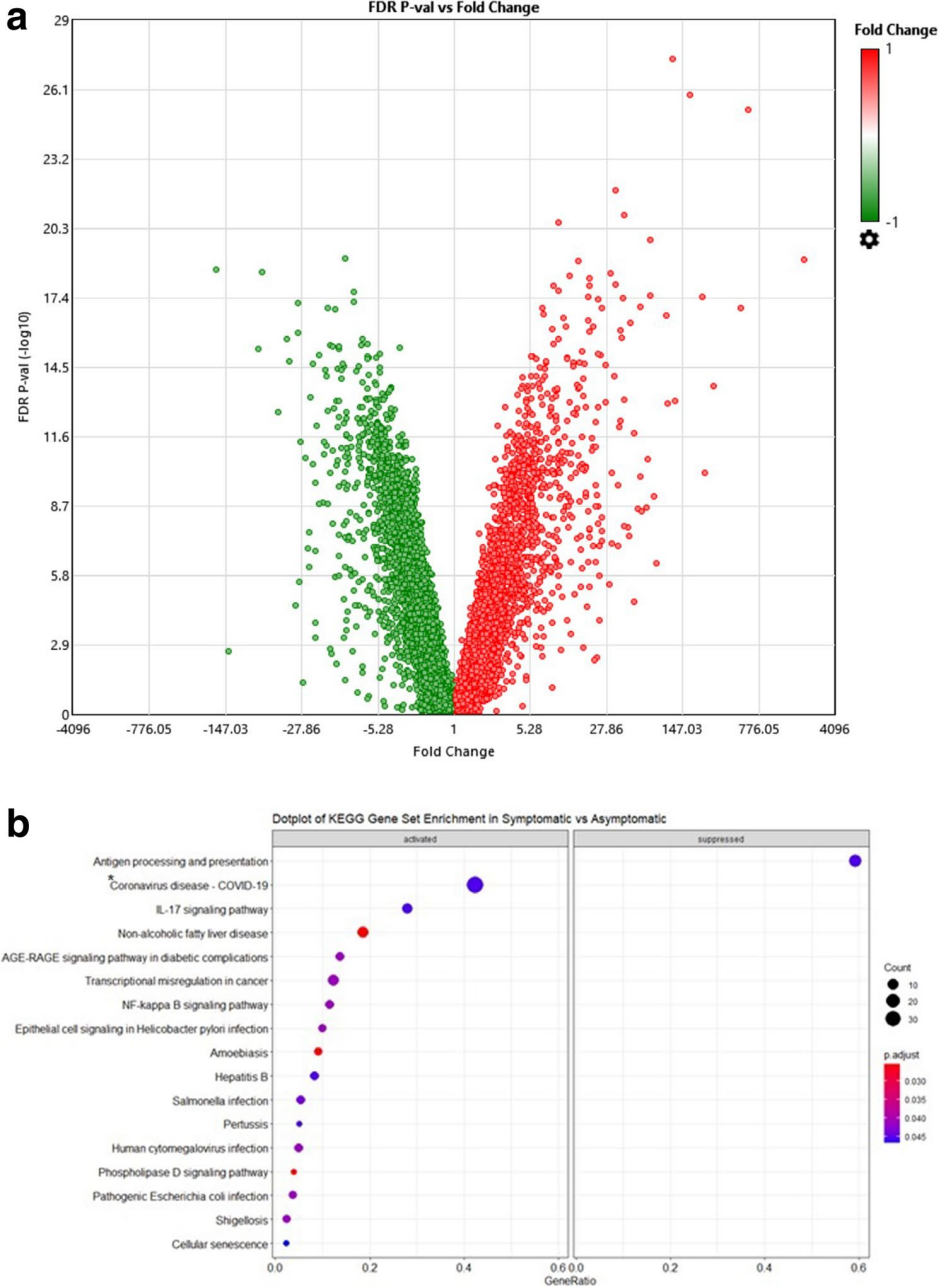
Symptomatic vs Asymptomatic COVID-19. (**a**) Volcano plot of Symptomatic vs Asymptomatic COVID-19 cases. The log2 (FC) (fold change) is plotted on the x-axis, and the negative log10 (FDR) (p-value) is plotted on the y-axis. The red points on the plot show Upregulated expression (n = 1939 genes), Downregulated genes (n = 1465) are shown in green. Analysis with the absolute value of log2 (FC) not less than 1 (FC = 2) and FDR values less than 0.05. (**b**) Dotplot of gene set enrichment analysis on KEGG molecular pathway applied on Symptomatic vs Asymptomatic COVID-19. KEGG molecular pathways on y-axis and gene ratio on x-axis. The greater the size of circle the greater the number of genes involved in a pathway the circles are colored based on p-adjusted value. ‘*’ ‘Coronavirus disease pathways’ ribosomal pathways, CXCL8 and NF-kB pathway.

We investigated the DEGs between Symptomatic and Asymptomatic COVID-19 cases, with 1939 Up- and 1465 Down-regulated genes. A volcano plot displays these using a > 2-logFC a two-way ANOVA paired analysis, with a false discovery rate (FDR) < 0.05 (Fig. 2a). This data was further run through a KEGG enrichment analysis to rank genes according the significant changes that occurred and grouped them according to biological pathways (Fig. 2b, Supplementary Tables 2 and 3).

KEGG enrichment analysis of biological components revealed that genes involved in pathways for antigen processing and presentation were suppressed in Symptomatic as compared with Asymptomatic cases. Further, the most significantly activated genes were those classified as Coronavius disease comprising; CXCL8, highly chemotactic for neutrophils; NF-kB pathway genes (FOS/JUN/NFKBIA) and a number of ribosomal proteins representing small and large sub-unit proteins and accessory proteins found to be affected in during viral infection of cells^24^ (Supplementary Table 2). Further, IL-17- and inflammatory pathways such as, those in non-alcoholic fatty liver disease (NAFLD), AGE-RAGE signaling and NFκβ pathways were upregulated (Fig. 2b). Additionally, genes involved in responses to bacterial, parasitic and viral diseases were also activated, indicating activation of innate and adaptive immune response genes.

### Differential gene expression between symptomatic cases with severe and mild COVID-19

**Figure 3:**
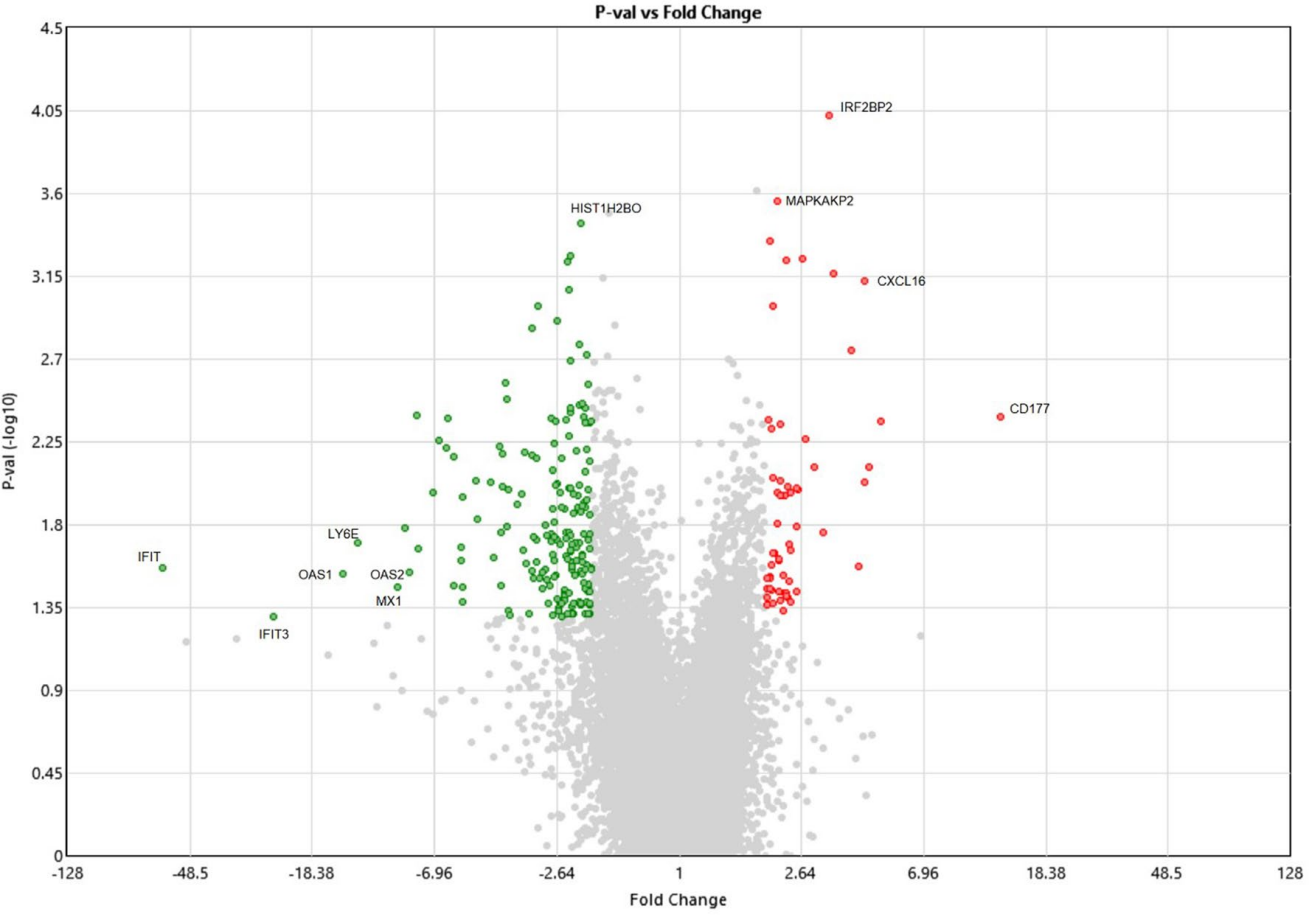
Differential regulation of ISGs between severe and mild Symptomatic COVID-19 cases. Volcano plot of Symptomatic ‘Severe’ vs ‘Mild’ COVID-19 cases. The log2 (FC) (fold change) is plotted on the x-axis, and the negative log10 (FDR) (p-value) is plotted on the y-axis. The red points on the plot show Upregulated expression (n = 53 genes), Downregulated genes (n = 183) are shown in green. Analysis with the absolute value of log2 (FC) not less than 1 (FC = 2) and FDR values less than 0.05. Genes which have a p < 0.05 ANOVA with Benjamini–Hochberg FDR are illustrated. Selected highly differentially regulated genes are labelled.

To further understand regulatory changes in those with Symptomatic COVID-19 we compared transcriptome data of those with severe and mild disease. We found 241 genes to be differentially regulated between the groups, with 53 upregulated and 183 downregulated genes. As depicted by the volcano plot (Fig. 3), CD177, a member of the Ly-6 gene superfamily involved with neutrophil proliferation was the most upregulated in severe COVID-19 cases. Additional markers upregulated in severe disease were MAPKAPK2 (MAP kinase-activated protein kinase 2, which regulates inflammatory cytokines), IRF2BP2 (interferon regulatory factor-2 binding protein-2, a transcriptional corepressor for interferon) and CXCL16 (a chemoattractant for activated CD8 T cells, NKT cells and Th1-polarized T cells that express CXCR6). Downregulated genes included HIST1H2BO (a replication-dependent histone gene cluster), and the ISGs, IFIT, IFIT3, OAS1, OAS3, LY6E and MX1.

### Activation of inflammatory responses and suppression of T cell immunity in severe COVID-19 cases

We validated our data analysis using an RNAseq data set from Germany by Aschenbrenner et al. who compared transcriptomes of COVID-19 cases with healthy controls^25^, focusing on severe COVID-19 and Controls only. These were used to run a gene set enrichment analysis (gseGO-BP/MF/CC, enrichKEGG) (GSEA) between the two groups. GSEA of the data from the report by Aschenbrenner et al.^25^, showed that in COVID-19 cases with severe disease, there was activation pathways belonging to the humoral immune response, complement activation, host innate immune responses such as, phagocytosis, leucocyte and neutrophil activation (Supplementary Fig. 1). However, there was suppression of ribosomal biogenesis and translation pathways together with downregulation of cytotoxic cell functions associated with both Natural Killer (NK) and T cells. Both T cell activation and differentiation pathways were seen to be suppressed.

### Upregulation of immune regulatory genes and interferon pathway genes in asymptomatic COVID-19 cases

**Figure 4:**
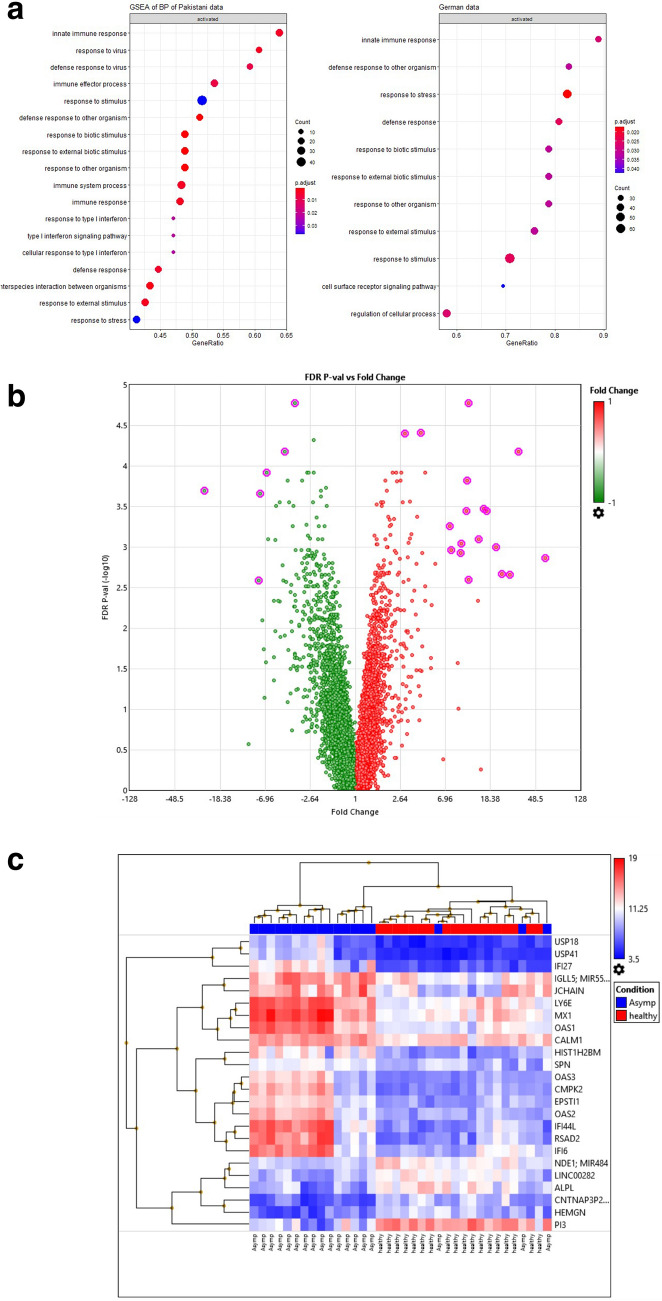
Comparative transcriptomic profiles in Asymptomatic COVID-19 and healthy Controls. (**a**) Dotplot of gene set enrichment analysis (GSEA) on KEGG biological process applied on Asymptomatic COVID-19 and healthy Controls data. (**b**) Volcano plot of DEGs between Asymptomatics (n = 18) and Controls (n = 18) shows an overview of upregulated and suppressed genes. The log2 (FC) (fold change) is plotted on the x-axis, and the negative log10 (FDR) (p-value) is plotted on the y-axis. The red points on the plot show Upregulated- (n = 144) and Down-regulated genes (n = 277) are shown in green. Analysis with the absolute value of log2 (FC) not less than 1 (FC = 2) and FDR values less than 0.05. (**c**) Unsupervised hierarchical clustering of genes identified with FC > 7 and FDR P-val(− log10) > 2.5, highlighted in Fig. 3b is depicted. Each column represents condition of sample [Asymptomatic (blue), Healthy (red)] and each row represents a gene. The heat map indicates the level of gene expression, red is increased and blue indicates decreased expression. (**d**) A histogram of immune genes of DEGs with FC > 2 and FDR p < 0.05. The genes are plotted on x-axis and their related Fold-Change are plotted on y-axis with standard deviation shown as error bars.

We subsequently interrogated transcriptional profiles of Asymptomatic COVID-19 and Controls in our Pakistani dataset to identify gene signatures associated with effective viral restriction of SARS-CoV-2. A Gene Set Enrichment (GSE) analysis for Biological Processes identified DEGs in the most affected pathways. This revealed activation of innate, defense response to virus, effector immune responses and cytokine pathways, which included type I interferon response and type I interferon pathway genes in Asymptomatics (Fig. 4a). Asymptomatic cases displayed the activation of IFN I, II, III and alpha/beta, JAK/STAT pathway, IL-1 mediated Myd88 signaling, IL 2, RIG-1 like receptor pathway and MAPK/ERK signaling pathways. Further, MHC class I along with perforins genes were also upregulated as compared with Controls.

To identify specific genes which could be driving the host-protective responses in Asymptomatic cases, we first examined the DEGs between the groups that included 144 Up and 277 Down genes (Fig. 4b). Twenty-four of the most differentially regulated genes were identified using a fold change of > 7 and a FDR − log10 cut off of 2.5. These comprised eighteen upregulated and six downregulated genes as visualized through hierarchical clustering demonstrated by a heat map (Fig. 4c). Upregulated genes comprised ISGs; *USP18, USP41, IFI *(interferon alpha inducible protein)-*27, MX1, OAS* (oligoadenylate synthase)-*1, OAS3, CMPK2, EPSTI1, OAS2, IFI44L, IFI6*. Further, upregulated genes were B cell related *IGL5, JCHAIN*, *LY6E* (Thymic Shared Antigen-1, proton ATPase) and *CALM1* (calmodulin 1) associated with blood and vasculature. Downregulated genes were *ALPL* (non-tissue specific alkaline phosphatase)*, HEMGN* (erythroid associated hemapoietic gene) and *PI3* (peptidase inhibitor 3).

### Inflammatory cytokines in plasma of asymptomatic COVID-19 cases

**Figure 5:**
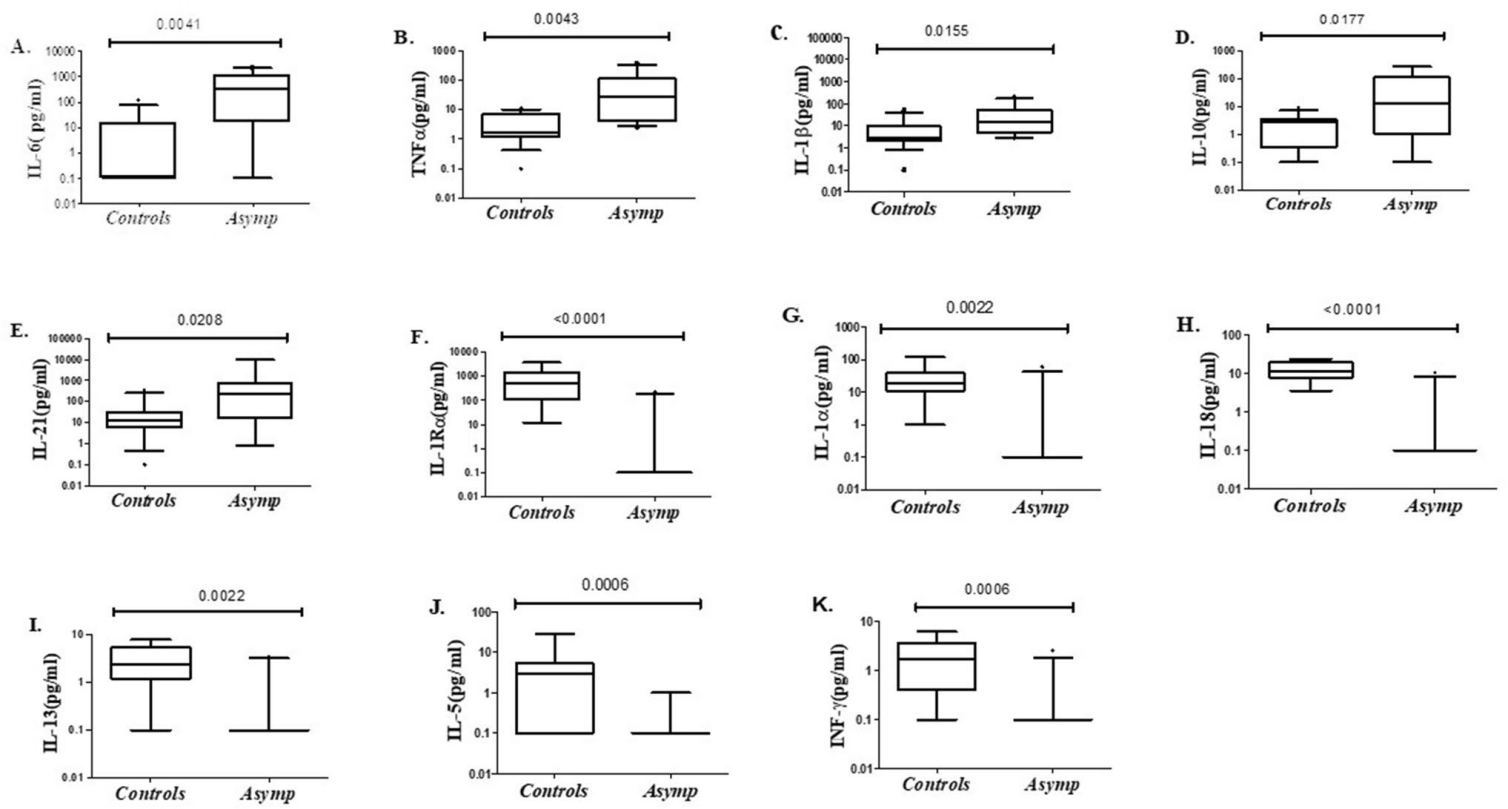
Circulating levels of cytokines in plasma of COVID-19 cases and controls. Plasma from COVID-19 Asymptomatic (n = 12) and healthy Controls (n = 13) were tested using the Th1/Th2 25-plex Procartaplex assay. (**A**) IL-6, (**B**) TNF-α, (**C**) IL-1β, (**D**) IL-10, (**E**) IL-21, (**F**) IL-1Rα, (**G**) IL-1α, (**H**) IL-18, (**I**) IL-13, (**J**) IL-5 and (**K**) IFN-γ. Data is shown as median values (horizontal line) with IQR (10th–90th quartile). Data between group was analysed using the Mann–Whitney *U* non-parametric assay and p values < 0.05 are indicated as significantly different.

We measured circulating levels of cytokines in Asymptomatic COVID-19 and Control groups. Plasma levels of IL-6, TNFα, IL-1β, IL-10 and IL-21 were significantly higher in Asymptomatic COVID-19 cases as compared with Controls (Fig. 5a–e). Whilst, ILR1α, IL-1α and IL-18 levels were reduced in Asymptomatic COVID-19 (Fig. 5f–k). These results illustrate systemic elevation of inflammatory cytokines in the Asymptomatic COVID-19 cases.

### Upregulation of Interferon stimulated genes and cytokine/chemokines pathways in asymptomatic COVID-19 cases as compared with moderate and severe COVID-19 disease

**Figure 6:**
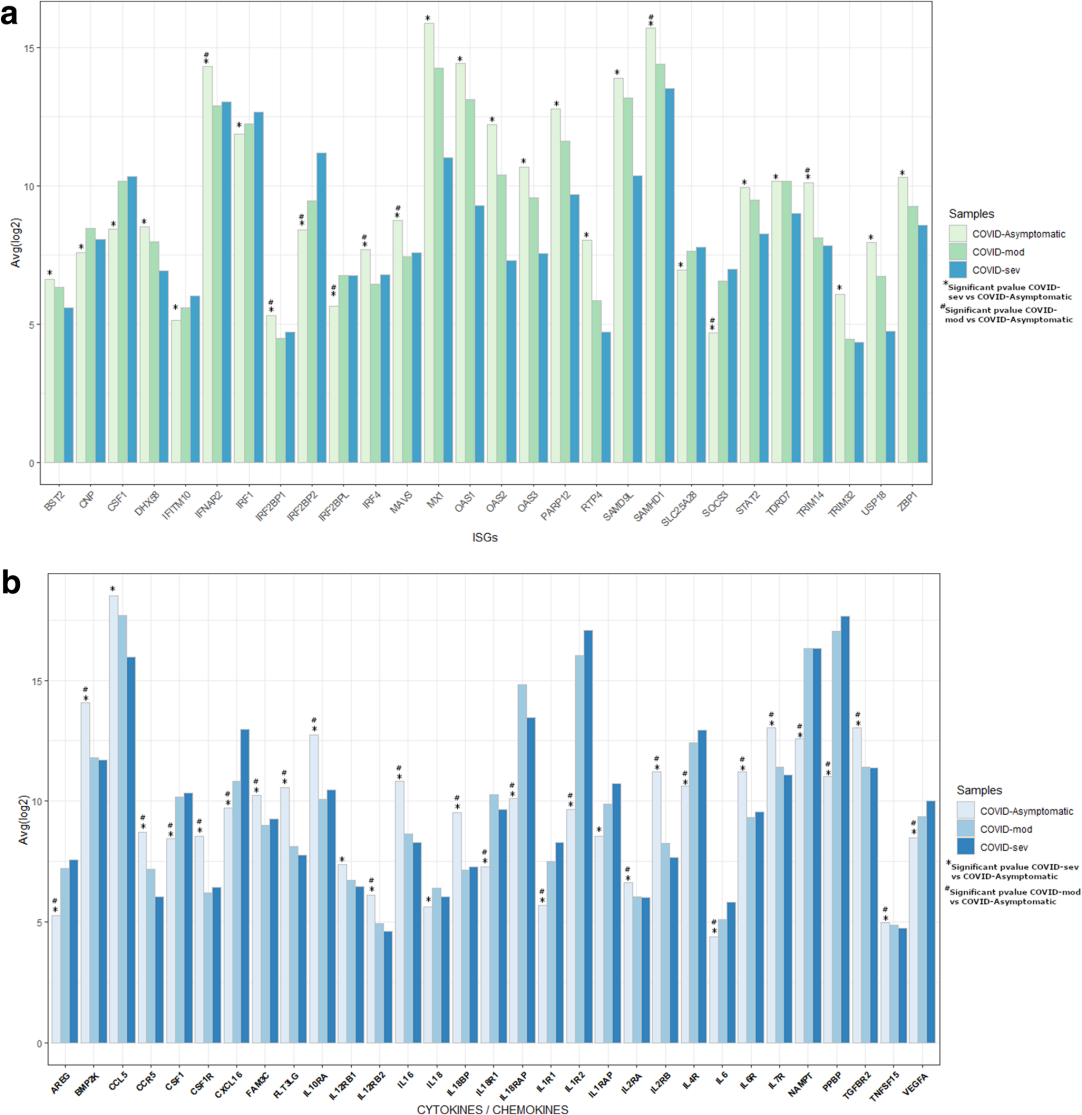
Differential activation of Interferon and cytokine genes across the COVID-19 severity spectrum. (**a**) Histogram of Interferon stimulated genes (ISGs) plotted on x-axis against avg(log2) values for severe (COVID-Sev), mild (COVID-Mild) and Asymptomatic (COVID-Asymp) cases. Higher avg(log2) values are exhibited by IFNAR2, MX1, OAS1 and SAMHD1. (**b**) Histogram of Cytokines/Chemokine genes plotted on x-axis against avg(log2) values for severe (COVID-Sev), mild (COVID-Mild) and Asymptomatic COVID-19 (COVID-Asymp). Higher avg(log2) values are exhibited by CCL5, IL1R2, NAMPT and PPBP. Significant p-values of COVID-Sev vs COVID-Asymp is labelled by * and significant p-values of COVID-Mod vs COVID-Asymp is donated by #.

To further investigate the role of interferon response and cytokine genes in the context of SARS-CoV-2 infection we compared DEGs across severity comparing Asymptomatic, mild and severe COVID-19 cases. We found twenty ISGs to be significantly upregulated in Asymptomatic as compared with severe COVID-19 cases (Fig. 6a). Highest avg (log2) values are exhibited by IFNAR2, MX1, OAS1 and SAMHD1 genes. Additional ISGs upregulated in Asymptomatics were; BST2, DHX58, IRF2BP1, IRF4, MAVS, OAS2, OAS3, PARP12, RTP4, SAMD9L, STAT2, TDR07, TRIM14, TRIM32, USP18 and ZBP1. Eight ISGs were downregulated in Asymptomatic as compared with severe COVID-19 cases; CNP, CSF1, IRF1, IFITM10, IRF2BP2, IRF2BPL, SLC25A28 and SOCS3. Of these, nine ISGs were differentially regulated in Asymptomatic versus mild COVID-19 cases; IFNAR2, IRF2BP1, IRF4, MAVS, and TRIM14 were upregulated; whilst IRF2BP2, IRF2BPL, and SOCS3 were downregulated.

In regard to cytokines and chemokine expression between COVID-19 cases we found that the highest avg (log2) values showing fold change were indicated by CCL5, IL1R2, NAMPT and PPBP (Fig. 6b). BMP2K, OCR5, CSF1R, FAM3C, FLT3LG, IL10RA, IL12RB, IL12RB2, IL16, IL18BP, IL2RA, IL2RB, IL6R, IL7R, TGFBR2, TNSF13, VEGF were all upregulated in Asymptomatic as compared with mild and severe COVID-19 case. Further, AREG, CSF1, IL18, IL18R1, IL18RAP, IL1R1, IL1RAP, IL4R, IL6, NAMPT and PPBP were found downregulated in Asymptomatic as compared with mild and severe COVID-19 cases. IL18 expression was downregulated in Asymptomatic as compared to severe COVID-19 cases.

## Discussion

We investigated host transcriptional responses in SARS-CoV-2 positive Asymptomatic and Symptomatic COVID-19 cases comparing them with uninfected Controls to identify biomarkers of host protective responses in individuals who show improved control of viral infection. Our study highlights the upregulation of type I Interferon-driven response genes in Asymptomatic COVID-19 cases.

Our Asymptomatic COVID-19 cases and those with mild Symptomatic COVID-19 were similar in age to the uninfected Control group, but younger than severe Symptomatic COVID-19 cases. The age range of our groups fits the demographics of COVID-19 patients seen through the first two waves of the pandemic in Pakistan during 2020. Over the same period studied, the Sindh Health Department COVID-19 report indicated that 76% of confirmed cases were below 50 years of age^26^ with a similar trend was observed in national data. Nasir et al. showed that there were no specific factors associated with hospital related morbidity and mortality of COVID-19 in Pakistan, except for an older age group^27^. These study samples were from between March and October 2020. In March, S, L and G clade strains were present with an increasing predominance of the GH clade with the D614G mutation found by October 2020^28^. Importantly, the samples in this study were collected before the emergence of SARS-CoV-2 variants of concern (VoC) known to have a greater transmissibility and have been found to be more virulent^29^.

In the context Pakistan, it is difficult to ascertain if the relatively lower morbidity and mortality from COVID-19 observed may be due to factors such as, other infections which have led to cross-protective immunity against SARS-CoV-2^11,12,30^. The younger age group may be a factor however, albeit our sample size for the mild Symptomatic group was small, we found both mild and severe Symptomatic cases to have transcriptional data segregated away from that of Asymptomatics and Controls.

The overall comparison of Symptomatic and Asymptomatic revealed upregulation of *TNF*α*, NFk*β*, IL1, HIF1A, ICAM, SOCS3* transcripts, and of type 2 cytokines (*IL4, IL6, IL10*), but downregulation of antigen processing and presentation pathways in the former groups. Raised inflammatory responses in COVID-19 individuals with advanced disease reflects the induction of a cytokine storm—up-regulation of IL-6, G-CSF, IL-1RA, and MCP1 has been shown to be associated with severe outcomes leading to mortality in patients with COVID-19^31^. Persistent expression of these inflammatory cytokines may be detrimental as it leads to increased influx of neutrophils, reported to be the source of tissue damage^32^. Heightened Th1, Th2 and Th17 responses along with increased chemokines may result in “cytokine storm”^33^, which can have devastating consequences on host inflammation^34^. Upregulation of DEGs related to protein synthesis genes in Symptomatic cases fits with increased viral mRNA translation in infected individuals, associated with viral replication and spread^35^. Reduced antigen processing responses in Symptomatic cases supports the observation of downregulated adaptive immunity in COVID-19 such as, T cells effector responses which are found to post treatment^36^. Overall, the transcriptional changes we observed in Symptomatic COVID-19 cases are in concordance with previous reports^37^.

We only had a few mild Symptomatic cases and these were younger in age than the severe Symptomatic group. However, PCA analysis revealed that they clustered together within the Symptomatic cases and away from the Asymptomatics and Controls. Comparison of severe and mild Symptomatic COVID-19 cases revealed an upregulation of CD177, the neutrophil marker, CXCL16, MAPKMAPK2 and IRF2BP2 with downregulation of ISGs; IFIT, IFIT3, OAS1, OAS2, LY6E and MX1 in severe cases. OAS is a family of molecules upregulated in response to ISGs is known to play a role in early viral clearance by degrading viral RNA in combination with RNase L^38^. LY6E, a GPI-anchored protein, has an impact on cellular receptors for viruses or viral glycoproteins in terms of their expression, kinetics, or biophysical properties, thus affecting their binding, trafficking, and membrane fusion^39^.

We compared our data set with the transcriptome data set from Germany published by Ashenbrenner et al.^25^, which showed the upregulation of neutrophil/granulocytes related processes and interferon responses in both severe and mild COVID-19 cases as compared with Controls^25^. Our analysis of the same data set comparing severe COVID-19 cases as with Controls also revealed activation of leucocyte and neutrophil activation (Supplementary Fig. 1). Further, we found downregulation of T cell proliferation and activation markers, as shown previously in COVID-19 disease^36^. Of note, Aschenbrenner et al. did not find any differential activation of ISGs between mild and severe COVID-19 cases^25^. However, this may be because their classification for ‘mild’ cases was WHO ordinal score ‘1–4’, whilst we compared cases further stratified into Asymptomatic ‘score 1–2’ and mild Symptomatic cases with a score of 4 only. Hence, our study was able to clearly focus on transcriptome responses of a Asymptomatic COVID-19 cohort with minimal or no symptoms, representing effective early host immune responses.

In this study we used an RNA microarray chip with > 21,000 transcripts including, immune, metabolic and regulatory pathway genes. Unlike in RNA sequencing, only known targets are identified and will not include novel RNAs. Therefore, there may be differences in the precise genes identified between our data and others using microarray or RNAseq based analysis.

Focus on transcriptional responses of Asymptomatics revealed upregulation of ISGs (such as, USP18, IFI27, LY6E, MX1, OAS1, IFI44L, RSAD2, CMPK2), in addition to histone genes and CALM1. CMPK2 has a well described antiviral role in HIV patients^40^. USP18 regulate type 1 IFNs, protecting against severe immune inflammation with calcification and polymicrogyria following viral infection^41^. RSAD2, an anti-viral factor, has been shown to under-expressed in SARS-CoV2^42^. Upregulation of IF44 and IF44L genes has been shown in early protection against respiratory syncytial virus^43^. Upregulated CALM1 gene in Asymptomatics adds another layer of protection by interacting with ACE-2 and inhibits shedding of its ectodomain^44^.

Raised plasma cytokines reflected increased inflammation in Asymptomatic individuals. IL-6, TNFα, IL1β are all proinflammatory responses whilst IL-10 downregulate the response and the balance of these is essential for maintaining immune regulation against pathogens^45^. SARS-CoV-2 has been shown to induce reduced IFN type I responses, increased pro-inflammatory cytokines and chemokines profile with down-regulated IL-10 responses^46^. The balance in which IFN responses are required is controlled by immune modulatory cytokines such as IL10 and TGFβ to prevent the tissue damage^46^.

Investigated the differential activation of Interferon and other cytokine genes across the COVID-19 disease severity spectrum identified an activation signature of type I interferon pathway genes and immune responses genes including cytokine signaling pathways in asymptomatic as compared with symptomatic COVID-19 cases.

Anti-viral ISGs including, MAVS, OAS1, OAS2, OAS3, BST2, DHX58, IFNAR2, IRF2BP1, IRF4, MX1, PARP12, SAMD9L, SAMHD1, STAT2, TDR07, TRIM14, TRIM32, USP18, ZBP1, IRF4 and STAT showed marked downregulation in severe COVID-19. MAVS is known to get activated downstream interferon signaling pathway with delayed kinetics in order to maintain antiviral responses^47^. Theses ISGs have been shown to mediate host anti-viral defense defenses such as, inhibition of viral recognition and of viral replication, and interfering with viral and cellular RNA translation^40,48–51^.

Further, IFNs activate downstream JAK/STAT pathways to regulate ISGs that induce an anti-viral state in the host^52^. Deficiency in STAT2 has been linked with compromised IFNα responses.

When cytokine and chemokine expression was compared between Asymptomatic, mild and severe COVID19, it was apparent that there was upregulation of pro-inflammatory cytokine/chemokine signaling pathways with downregulated AREG, IL18, IL18R1, IL18RAP, IL1R1, IL1R2, IL1RAP, IL6, PPBP and VEGFA gene expression. The association of IL18 with acute respiratory distress syndrome has been described in infections with avian influenza virus (H5N1 and H7N9)^53^. Patients with increased IL-6 responses have shown to be at increased risk for the requirement of mechanical ventilation and therefore, IL-6 is now recommended to be included in the diagnostic workup of severe COVID-19 cases^54^. Up-regulated VEGFA in severe COVID-19 cases is in line with the findings of the study where it has been indicated as a markers of endothelial dysfunction with significant correlation with disease severity^55^. AREG is an epidermal growth factor ligand which plays an important role in tissue repair and pulmonary fibrosis and its increased expression in patients with severe COVID-19 disease has been negatively correlated with genes associated with cytotoxic NK cell functions^56^. Hence, the upregulated inflammatory cytokines and downregulation of IL18, IL6, VEGF and AREG in Asymptomatics are all suggestive of improved control of SARS-CoV-2 infection.

Overall, discrepancies observed between studies investigating the role of type I and III interferons in protecting against SARS-CoV-2 may possibly be due to the different stages of COVID-19 disease in cohorts investigated. Our study highlights upregulated type I interferon responses in Asymptomatic COVID-19 cases. Our findings are supported by studies that show increased production of type I IFNs in patients with mild disease and improved clinical outcomes^57^. Other studies have reported the insufficient activation of IFN I responses in moderate to severe cases of SARS-CoV2 infection, which may explain the failure to restrict viral replication in a timely fashion^20^. Longitudinal studies investigating alterations in immune responses confirmed the late induction of Type I IFNs in peripheral blood of SARS-CoV2 patients^58^.

IFN based therapies are used to treat viral infections such as hepatitis C and HIV^59,60^. A recent clinical trial has shown improvement in recover time when patients with severe COVID-19 were treated with IFN Iβ^61^. In summary, our study provides insights into the role of immune responses which may be associated with early clearance of virus. Therefore, we hypothesize that treatment modalities such as early administration of type I interferon treatment may facilitate clearance of SARS-CoV-2.

## Methods

### Study subjects

This study received approval from the Ethics Review Committee of the Aga Khan University (AKU). All methods were performed in accordance with the relevant guidelines and regulations. All study subjects were aged over 18 years. Informed consent was taken from all study subjects or their adult next of kin. COVID-19 patients included in the study were recruited in the period April–October 2020.

### SARS-CoV-2 PCR testing

Diagnostic testing was conducted at the AKU Hospital Clinical Laboratories, Karachi using the COBAS^®^ SARS-CoV-2 assay (COBAS^®^ 6800 Roche platform). AKUH Clinical Laboratories are College of American Pathologists, USA accredited.

### Study subjects

COVID-19 disease was classified as per WHO ordinal scale (Supplementary Table 2). All COVID-19 patients comprising Asymptomatic and Symptomatic groups all had a positive SARS-CoV-2 PCR for a nasopharyngeal swab sample.

Of the eighteen Asymptomatic COVID-19 cases, two had no symptoms and sixteen had one or two mild symptoms such as a short (1–2 days) history of fever, sore throat or myalgia. These were scored (1–2) as per the WHO ordinal scale. They were identified during screening due to contact with a COVID-19 positive case, or as part of regular screening conducted due to travel from outside of the city into Karachi. Blood samples from Asymptomatic cases were taken within 2- 3 days of their positive SARS-CoV-2 PCR result. None from this group required any medical treatment.

Symptomatic cases were admitted to AKUH at the time of recruitment. Their clinical details are provided in Supplementary Table 1. Blood samples from Symptomatic cases were drawn within 2 days of their positive SARS-CoV-2 PCR result, prior to the administration of any an anti-viral treatment, or IL-6 antagonist. Upon admission, cases had WHO ordinal scores between 4 and 7. Symptomatic cases can be further sub-divided into those with ‘Mild’ (score of 4, n = 3) or ‘Severe’ (score of 5 – 7, n = 8) COVID-19.

Uninfected healthy controls did not have any current or prior history of SARS-CoV-2 infection. These included cases 6 cases recruited in March 2020 and 12 cases from 2018.

### IgG to spike protein ELISA

IgG antibodies to Spike protein were measured using an in-house ELISA method^62^. Recombinant Spike protein for ELISA was kindly provided by Dr. Paula Alves, IBET, NOVA University, Portugal. Sera from Asymptomatic COVID-19 cases and uninfected Controls were available for serology testing. Serum for Symptomatic cases was not available.

### RNA microarray data

RNA was extracted from whole blood collected in plasma/EDTA tube using the Qiagen RNA Blood Mini Kit (Qiagen, GmbH, Germany). One hundred nanogram of RNA was used for the preparation of cDNA for use in the Clariom™ S Assay, human (Affymetrix, USA; 902927). The Clariom S Array has hybridization probes for 21,488 genes The arrays were scanned using an Affymetrix autoloader system. Array data was generated and used for processing. For accession number generation, array output raw files (CEL files) and processed files (CHP) were submitted to Gene Expression Omnibus (GEO) NCBI and available as GSE177477. The accession numbers generated are in Supplementary Table 3.

### Statistical analysis

Differences between age groups and lab parameters were compared using Kruskal–Wallis test. CEL files were analysed using the TAC Transcriptome Analysis Software Suite (TACS version 2) using the Summarization Method: Gene Level—SST-RMA Pos vs Neg AUC Threshold: 0.7 against Genome Version: hg38 (Homo sapiens). Cellular pathway analysis of significant differentially expressed genes (DEGs) up- or down-regulated (log FC (fold change) < − 2 or > 2; FDR adjusted p value < 0.05) were identified by Transcriptome Analysis Console Software (TACS). Further, hierarchical clustering and volcano plots were made using TACS.

### Functional enrichment analysis

To obtain further insights into the function of the differentially expressed genes (DEGs), we performed Gene Ontology (GO) analysis and Kyoto Encyclopedia of Genes and Genomes (KEGG) pathway enrichment analysis, using clusterProfiler^63^ (Fig. 2). The R package clusterProfiler, perform statistical methods to analyze and visualize functional profiles (GO and KEGG) of gene and gene clusters^64^. We use two types of functions from clusterProfiler i.e., enricher function (enrichGO, enrichKEGG) for hypergeometric test and GSEA (gseGO, gseKEGG) function for gene set enrichment analysis on user defined data. GO enrichment analysis is carried out employing enrichGO function which requires a gene list as input vector. The results are annotated along three ontologies: Molecular Functions, Biological Processes and Cellular Components with the following parameters: pvalueCutoff = 0.05, pAdjustMethod = "BH" (Benjamani and Hochberg) and qvalueCutoff = 0.05. While the enrichKEGG is simpler, it requires a gene-list as input, parameter of pvalueCutoff = 0.05 and organism of interest (homo sapiens “hsa”). Gene set enrichment analysis is performed on GO terms using gseGO which requires gene-list in the form of input vector, organism of interest (database: org.Hs.eg.db), pvalueCutoff = 0.05, minGSSize (minimal size of genes annotated by Ontology term for testing) = 10 and maxGSSize (maximum number of genes annotated for testing) = 800. gseKEGG function is similar with respect to input parameters (genelist, organism = hsa, minGSSize, maxGSSize and pvalueCutoff), applied on KEGG database. For visualization of results related R packages such as GOplot, enrichplot, DOSE and pathview were used to generate pathway maps, dotplots, heatmaps and barplot.

### Comparison with a published transcriptional data set

Open data published by Ashenbrennen et al. of *German Covid-19 omics initiative* was extracted online resources for analysis^25^. Data of 20 COVID-19 patients with severe disease symptoms) and 10 healthy control donors was downloaded. Functional enrichment analysis (gseGO-BP/MF/CC, enrichKEGG) was applied on the sample data and same thresholds (as in the reference study) were used to calculate the GSEA for KEGG molecular pathways and GO ontology.

### Cytokine measurements on Luminex assay

Plasma samples from Asymptomatic COVID-19 and healthy Controls were tested using the Cytokine 25-Plex Human ProcartaPlex™ Panel 1B; ThermoFisher Scietific, USA (GM-CSF, IFN gamma, IL-1 beta, IL-2, IL-4, IL-5, IL-6, IL-12p70, IL-13, IL-18, TNF alpha, IL-9, IL-10, IL-17A (CTLA-8), IL-21, IL-22, IL-23, IL-27, IFN alpha, IL-1 alpha, IL-1RA, IL-7, IL-15, IL-31, TNF beta), using the Luminex 100 system. Sera for Symptomatic cases were not available for testing.

## Supplementary Information

Supplementary Information.

## References

[1] JHU. *COVID-19 Data Repository by the Center for Systems Science and Engineering *(*CSSE*)* at Johns Hopkins University*. https://github.com/CSSEGISandData/COVID-19 (2021).

[2] Pakistan Bureau of Statistics (2016). Social Indicators of Pakistan.

[3] Government of Pakistan. *COVID-19 Health Advisory Platform. Ministry of National Health Services Regulations and Coordination.* https://covid.gov.pk/ (2021).

[4] Lai CC (2020). Global epidemiology of coronavirus disease 2019 (COVID-19): Disease incidence, daily cumulative index, mortality, and their association with country healthcare resources and economic status. Int. J. Antimicrob. Agents.

[5] Dil S, Dil N, Maken ZH (2020). COVID-19 trends and forecast in the eastern mediterranean region with a particular focus on Pakistan. Cureus.

[6] Braddick, I. Global coronavirus cases pass 2 million as death toll hits 128,000. News World (2020).

[7] Imran M (2021). COVID-19 situation in Pakistan: A broad overview. Respirology.

[8] GoS. *Daily Situation Report*. https://www.sindhhealth.gov.pk/upload/daily_status_report/Daily_Situation_Report_for_1st_January_2021.pdf (2021).

[9] JHU. *Coronavirus Resource Center* (2021).

[10] Nisar MI (2021). Serial population-based serosurveys for COVID-19 in two neighbourhoods of Karachi, Pakistan. Int. J. Infect. Dis..

[11] Hasan, M. *et al.* Increasing IgG antibodies to SARS-CoV-2 in asymptomatic blood donors through the second COVID-19 wave in Karachi associated with exposure and immunity in the population. 10.21203/rs.3.rs-941908/v1 (2021).

[12] Tso FY (2021). High prevalence of pre-existing serological cross-reactivity against severe acute respiratory syndrome coronavirus-2 (SARS-CoV-2) in sub-Saharan Africa. Int. J. Infect. Dis..

[13] Guzik TJ (2020). COVID-19 and the cardiovascular system: Implications for risk assessment, diagnosis, and treatment options. Cardiovasc. Res..

[14] Walls AC (2020). Structure, function, and antigenicity of the SARS-CoV-2 spike glycoprotein. Cell.

[15] Park WB (2020). Virus isolation from the first patient with SARS-CoV-2 in Korea. J. Korean Med. Sci..

[16] Fung SY, Yuen KS, Ye ZW, Chan CP, Jin DY (2020). A tug-of-war between severe acute respiratory syndrome coronavirus 2 and host antiviral defence: Lessons from other pathogenic viruses. Emerg. Microbes Infect..

[17] Qin C (2020). Dysregulation of immune response in patients with COVID-19 in Wuhan, China. Clin. Infect. Dis..

[18] Tay MZ, Poh CM, Rénia L (2020). The trinity of COVID-19: Immunity, inflammation and intervention. Nat. Rev. Immunol..

[19] King C, Sprent J (2021). Dual nature of type I interferons in SARS-CoV-2-induced inflammation. Trends Immunol..

[20] Blanco-Melo D (2020). Imbalanced host response to SARS-CoV-2 drives development of COVID-19. Cell.

[21] Zanoni I (2021). Interfering with SARS-CoV-2: Are interferons friends or foes in COVID-19?. Curr. Opin. Virol..

[22] WHO. Novel Coronavirus COVID-19 Therapeutic Trial Synopsis (2020).

[23] Liu A, Li Y, Peng J, Huang Y, Xu D (2021). Antibody responses against SARS-CoV-2 in COVID-19 patients. J. Med. Virol..

[24] Li S (2019). Regulation of ribosomal proteins on viral infection. Cells.

[25] Aschenbrenner AC (2021). Disease severity-specific neutrophil signatures in blood transcriptomes stratify COVID-19 patients. Genome Med..

[26] Health Department (2020). Daily Situation Report.

[27] Nasir N (2021). Clinical characteristics and outcomes of COVID-19: Experience at a major tertiary care center in Pakistan. J. Infect. Dev. Ctries..

[28] Ghanchi NK (2021). Higher entropy observed in SAR-CoV-2 genomes from the first COVID-19 wave in Pakistan. PLoS One.

[29] Tao K (2021). The biological and clinical significance of emerging SARS-CoV-2 variants. Nat. Rev. Genet..

[30] Uyoga S (2021). Seroprevalence of anti-SARS-CoV-2 IgG antibodies in Kenyan blood donors. Science.

[31] Mudd PA (2020). Distinct inflammatory profiles distinguish COVID-19 from influenza with limited contributions from cytokine storm. Sci. Adv..

[32] Pelletier M (2010). Evidence for a cross-talk between human neutrophils and Th17 cells. Blood.

[33] Bhaskar S (2020). Cytokine storm in COVID-19-immunopathological mechanisms, clinical considerations, and therapeutic approaches: The REPROGRAM Consortium Position Paper. Front. Immunol..

[34] Zeng F (2020). Association of inflammatory markers with the severity of COVID-19: A meta-analysis. Int. J. Infect. Dis..

[35] Xu LH, Huang M, Fang SG, Liu DX (2011). Coronavirus infection induces DNA replication stress partly through interaction of its nonstructural protein 13 with the p125 subunit of DNA polymerase delta. J. Biol. Chem..

[36] Ouyang Y (2020). Downregulated gene expression spectrum and immune responses changed during the disease progression in patients with COVID-19. Clin. Infect. Dis..

[37] Wang H (2008). SARS coronavirus entry into host cells through a novel clathrin- and caveolae-independent endocytic pathway. Cell Res..

[38] Huang IC (2011). Distinct patterns of IFITM-mediated restriction of filoviruses, SARS coronavirus, and influenza A virus. PLoS Pathog..

[39] Yu J, Liu SL (2019). Emerging role of LY6E in virus–host interactions. Viruses.

[40] El-Diwany R (2018). CMPK2 and BCL-G are associated with type 1 interferon-induced HIV restriction in humans. Sci. Adv..

[41] Jimenez Fernandez D, Hess S, Knobeloch KP (2019). Strategies to target ISG15 and USP18 toward therapeutic applications. Front. Chem..

[42] McClain MT (2020). Dysregulated transcriptional responses to SARS-CoV-2 in the periphery support novel diagnostic approaches. medRxiv..

[43] Busse DC (2020). Interferon-induced protein 44 and interferon-induced protein 44-like restrict replication of respiratory syncytial virus. J. Virol..

[44] Lambert DW, Clarke NE, Hooper NM, Turner AJ (2008). Calmodulin interacts with angiotensin-converting enzyme-2 (ACE2) and inhibits shedding of its ectodomain. FEBS Lett..

[45] Buszko M (2021). Lessons learned: New insights on the role of cytokines in COVID-19. Nat. Immunol..

[46] Lindner HA, Velasquez SY, Thiel M, Kirschning T (2021). Lung protection vs. infection resolution: Interleukin 10 suspected of double-dealing in COVID-19. Front. Immunol..

[47] Dixit E (2010). Peroxisomes are signaling platforms for antiviral innate immunity. Cell.

[48] Dolskiy AA (2020). Deletion of BST2 cytoplasmic and transmembrane N-terminal domains results in SARS-CoV, SARS-CoV-2, and influenza virus production suppression in a vero cell line. Front. Mol. Biosci..

[49] Russell AJ (2021). SAMD9L autoinflammatory or ataxia pancytopenia disease mutations activate cell-autonomous translational repression. Proc. Natl. Acad. Sci. U.S.A..

[50] Chiang HS, Liu HM (2018). The molecular basis of viral inhibition of IRF- and STAT-dependent immune responses. Front. Immunol..

[51] Welsby I (2014). PARP12, an interferon-stimulated gene involved in the control of protein translation and inflammation. J. Biol. Chem..

[52] Schneider WM, Chevillotte MD, Rice CM (2014). Interferon-stimulated genes: A complex web of host defenses. Annu. Rev. Immunol..

[53] Tjan LH (2021). Early differences in cytokine production by severity of coronavirus disease 2019. J. Infect. Dis..

[54] Zhang J (2020). Serum interleukin-6 is an indicator for severity in 901 patients with SARS-CoV-2 infection: A cohort study. J. Transl. Med..

[55] Rovas A (2021). Microvascular dysfunction in COVID-19: The MYSTIC study. Angiogenesis.

[56] Kramer B (2021). Early IFN-alpha signatures and persistent dysfunction are distinguishing features of NK cells in severe COVID-19. Immunity.

[57] Hadjadj J (2020). Impaired type I interferon activity and inflammatory responses in severe COVID-19 patients. Science.

[58] Lucas C (2020). Longitudinal analyses reveal immunological misfiring in severe COVID-19. Nature.

[59] Enomoto H, Nishiguchi S (2015). Factors associated with the response to interferon-based antiviral therapies for chronic hepatitis C. World J. Hepatol..

[60] Azzoni L (2013). Pegylated Interferon alfa-2a monotherapy results in suppression of HIV type 1 replication and decreased cell-associated HIV DNA integration. J. Infect. Dis..

[61] Alavi Darazam I (2021). Role of interferon therapy in severe COVID-19: The COVIFERON randomized controlled trial. Sci. Rep..

[62] Stadlbauer D (2020). SARS-CoV-2 seroconversion in humans: A detailed protocol for a serological assay, antigen production, and test setup. Curr. Protoc. Microbiol..

[63] Yu G, Wang LG, Yan GR, He QY (2015). DOSE: An R/Bioconductor package for disease ontology semantic and enrichment analysis. Bioinformatics.

[64] Yu G (2018). ClusterProfiler: Universal enrichment tool for functional and comparative study. BioRxiV..

