# Supplementary material for: Upregulated type I interferon responses in asymptomatic COVID-19 infection are associated with improved clinical outcome
Source: Sci Rep. 2021 Nov 25;11:22958. doi: 10.1038/s41598-021-02489-4 (PMC8617268; doi:10.1038/s41598-021-02489-4)
Supplement: Supplementary file 1 — Supplementary Information. [file 41598_2021_2489_MOESM1_ESM.pdf]

Upregulated type I interferon responses in asymptomatic COVID-19 infection are associated with improved clinical outcome

Supplementary Figure 1. Suppression of T cell activation in severe COVID-19 cases. We investigated differential regulation of transcriptional pathways between severe COVID-19 (n=20) and healthy controls (n=11) using RNAseq data from a published data set from Germany. A dotplot of GSEA on KEGG biological process applied on data from the study by Ashenbrenner et al. is displayed with pathways depicted on the y-axis and gene ratios on the x-axis. The size of circles in the dot plot is related to the increasing number of genes involved in a pathway. Circles are colored based on a p-adjusted value for differential change between the groups studied.

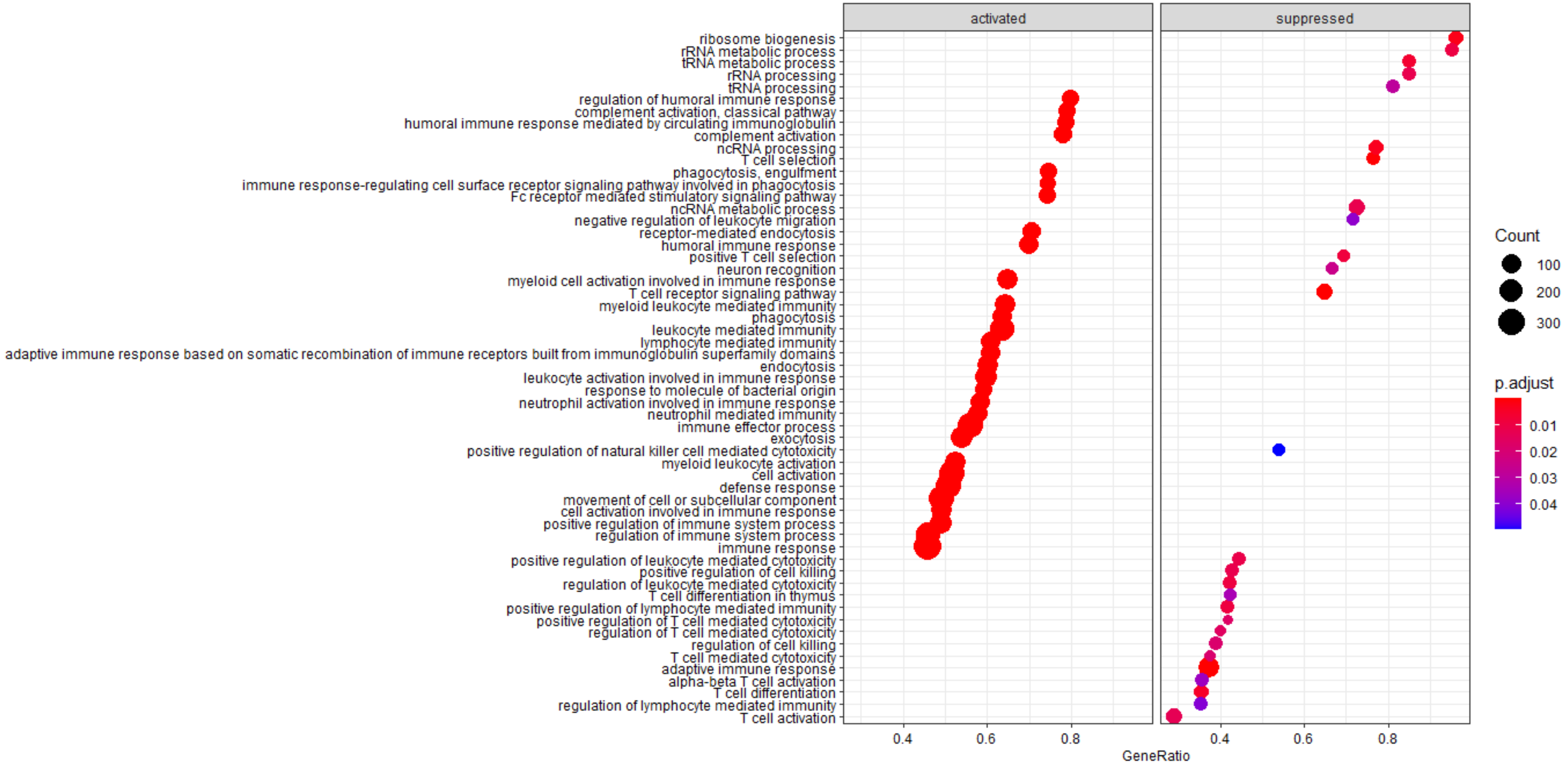

Supplementary Figure 2. Enrichment plot of KEGG GSEA corona virus disease profile of the running enrichment score and position in the ranked list of genes. A positive enrichment score is observed indicating co-relation with first phenotype. A green line is showing a running Enrichment score for the gene set the analysis walks down the ranked gene list and a maximum enrichment score is highlighted with a red dotted line. The blue rectangle shows a leading edge subset; these are the genes that appear in the ranked list prior to the peak score.

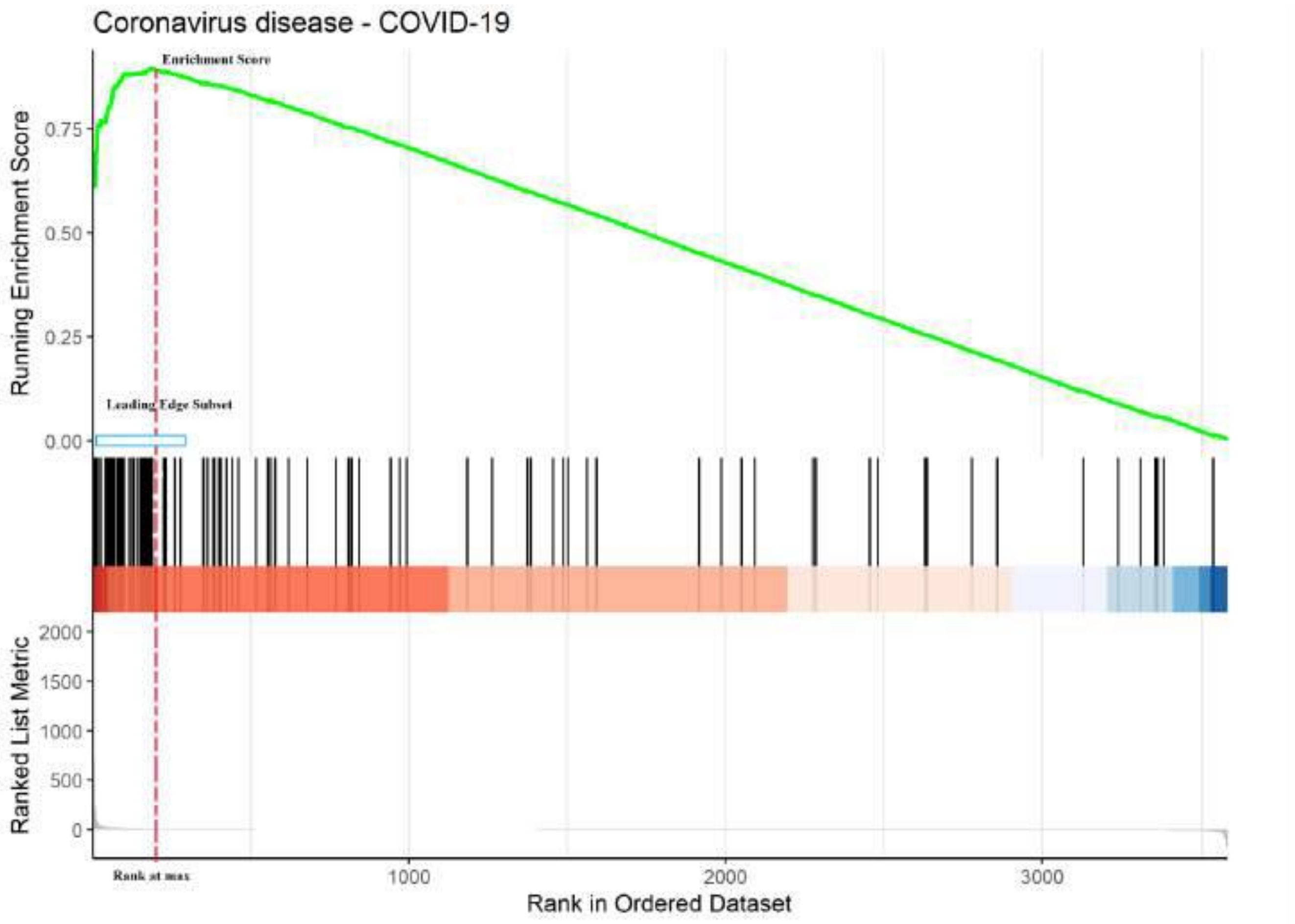

Supplementary Table 1. Clinical description of Symptomatic COVID-19 cases

ID	Age (years)	Sex	WHO_score _admission	Severity	D dimer (mg/L FEU)	CRP (mg/L)	LDH (I.U/L)	Ferritin (ng/mL)	WBC (4.8-11.3x10E9/L)	lymphocytes (17.5-45 %)	NLR	Comorbids	WHO_ score_ discharge	Outcome
S1	20-24	F	4	mild	8.9	142.6	528.0	486.3	3.6	33.0	1.8	NA	2	discharge
S9	44 - 49	M	4	mild	0.4	200.0	47.2	368.9	16.1	18.8	4.2	DM, HTN, IHD	2	discharge
S3	55 - 59	M	4	mild	1.1	73.8	436.0	180.4	3.8	27.2	4.1	DM HTN	2	discharge
S10	55 - 59	M	5	severe	0.6	254.8	570.0	1758.6	9.8	14.9	3.4	DM, HTN, IHD	2	discharge
S11	55 - 59	F	5	severe	1	6.3	3792.0	1393.2	10.1	7.0	2.0	HTN, chronic atrial fibrillation, asthma	2	discharge
S4	60 - 64	M	5	severe	3.2	201.0	354.0	639.5	9.0	9.5	10.8	DM HTN	8	deceased
S6	65 - 69	M	5	severe	NA	196.9	9.6	1797.1	12.3	7.5	8.3	HTN, IHD	8	deceased
S7	65 - 69	M	5	severe	6.3	7.6	4.2	82.7	4.2	20.2	6.7	DM, HTN, IHD (S/P CABG)	8	deceased
S2	70 - 74	M	6	severe	>30	210.0	821.0	642.0	8.6	30.6	19.4	DM HTN CAD	8	deceased
S8	75 - 79	M	7	severe	>30	21.3	5309.0		17.9	17.8	3.8	NA	8	deceased
S5	80 - 84	M	5	severe	4	13.4	619.0	1771.1	11.2	3.5	30.3	DM, IHD	8	deceased
**normal range**					<0.5 mg/L FEU	0-0.5mg/L	120-246 I.U/L	22-322g/mL	4.8-11.3 x10E9/L	17.5-45%	1-4			

CRP, C-reactive protein; LDH, lactate dehydrogenase; NLR, neutrophil lymphocyte ratio; DM, diabetes mellitus type II; HTN, hypertension; IHD, ischemic heart disease; CAD, coronary heart disease; HTN, hypertension; ARDS, acute respiratory distress syndrome.

Supplementary Table2. WHO Ordinal scale for clinical improvement in COVID-19[#]

Patient state	Descriptor	Score
Uninfected	No clinical or virological evidence of infection	0
Ambulatory	No limitation of activities	1
	Limitation of activities	2
Hospitalized, mild Disease	No oxygen therapy	3
	Oxygen by mask or nasal cannulae	4
Hospitalized Severe Disease	Non-invasive ventilation or high-flow oxygen	5
	Invasive mechanical ventilation without other organ support	6
	Invasive ventilation with other organ support (e.g., ECMO, CRRT, vassopressors)	7
Dead	Death	8

ECMO; extracorporeal membrane oxygenation; CRRT, continours renal replacement therapy

#, Adapted from "WHO R&D Blueprint novel Coronavirus COVID-19 Therapeutic Trial Synopsis", World Health Organization, 2020.

Supplementary Table 3. Symptomatic versus Asymptomatic
#%result_name=Analysis_31
#%array_type=Clariom_S_Human
#%annotation=Clariom_S_Human.r1.na36.hg38.a1.transcript.csv
#%comparison=Symp vs Asymp

ID	Symp Avg (	Asymp Avg	Fold Chang	P-val	FDR P-val	Gene Symb	Description	Group
TC1400008940.hg.1	19.16	12.28	117.7	1.94E-32	4.16E-28	NFKBIA	nuclear factor of kappa light polypeptide gene	Multiple_Complex
TC0600009597.hg.1	19.2	11.78	170.91	1.26E-30	1.35E-26	TNFAIP3	tumor necrosis factor, alpha-induced protein 3	Multiple_Complex
TC0100011453.hg.1	15.93	6.66	615.69	7.14E-30	5.10E-26	G0S2	G0/G1 switch 2	Multiple_Complex
TC1000007990.hg.1	11.35	6.26	33.93	2.21E-26	1.18E-22	DDIT4	DNA damage inducible transcript 4	Multiple_Complex
TC0800008801.hg.1	14.53	9.19	40.51	3.00E-25	1.29E-21	TRIB1	tribbles pseudokinase 1	Multiple_Complex
TC0900007977.hg.1	10.07	6.76	9.87	7.77E-25	2.78E-21	CARD19	caspase recruitment domain family, member 1	Multiple_Complex
TC0600011386.hg.1	14.44	8.28	71.69	4.53E-24	1.39E-20	IER3	immediate early response 3	Coding
TC1200009580.hg.1	7.44	10.86	-10.73	3.35E-23	8.98E-20	FBXL14	F-box and leucine-rich repeat protein 14	Multiple_Complex
TC0400007836.hg.1	17.23	6.2	2095.38	4.14E-23	9.88E-20	CXCL8	chemokine (C-X-C motif) ligand 8	Multiple_Complex
TC1900006588.hg.1	12.85	8.93	15.13	5.25E-23	1.13E-19	GADD45B	growth arrest and DNA-damage-inducible, bet	Multiple_Complex
TC0300010775.hg.1	9.63	17.13	-181.32	1.37E-22	2.67E-19	CX3CR1	chemokine (C-X3-C motif) receptor 1	Coding
TC0100017110.hg.1	9.34	15.39	-66.39	1.77E-22	3.17E-19	FCMR	Fc fragment of IgM receptor	Multiple_Complex
TC1000006652.hg.1	12.51	7.6	30	2.13E-22	3.52E-19	PFKFB3	6-phosphofructo-2-kinase/fructose-2,6-bipho	Multiple_Complex
TC1200010866.hg.1	13.61	9.97	12.51	3.14E-22	4.80E-19	CD63	CD63 molecule	Multiple_Complex
TC0300013855.hg.1	15.18	10.93	19.02	4.37E-22	6.25E-19	NFKBIZ	nuclear factor of kappa light polypeptide gene	Multiple_Complex
TC0200007132.hg.1	15.55	10.45	34.29	7.94E-22	1.06E-18	YPEL5	yippee like 5	Multiple_Complex
TC0100015975.hg.1	12.77	8.52	19.02	9.38E-22	1.15E-18	RIT1	Ras-like without CAAX 1	Multiple_Complex
TC1200012270.hg.1	12.15	9.02	8.76	9.62E-22	1.15E-18	RILPL2	Rab interacting lysosomal protein-like 2	Multiple_Complex
TC0100008105.hg.1	9.32	6.03	9.84	1.81E-21	2.04E-18	PLK3	polo-like kinase 3	Multiple_Complex
TC1700007944.hg.1	6.79	9.96	-9.03	2.17E-21	2.33E-18	TMEM106A	transmembrane protein 106A	Multiple_Complex
TC2100007599.hg.1	15.34	9.17	72.34	2.94E-21	3.01E-18	SAMSN1	SAM domain, SH3 domain and nuclear localiza	Multiple_Complex
TC1200008057.hg.1	15.14	10.92	18.58	3.44E-21	3.36E-18	IRAK3; MIR	interleukin 1 receptor associated kinase 3; mic	Multiple_Complex
TC0700007480.hg.1	12.67	4.86	224.42	3.74E-21	3.49E-18	ABCA13	Memczak2013 ALT_ACCEPTOR, ALT_DONOR,	NonCoding
TC0900010769.hg.1	15.51	10.18	40.14	4.39E-21	3.92E-18	NFIL3	nuclear factor, interleukin 3 regulated	Coding
TC0600007012.hg.1	11.01	6.49	22.96	5.32E-21	4.57E-18	CD83	CD83 molecule	Multiple_Complex
TC0700013383.hg.1	12.59	8.06	23.18	5.69E-21	4.69E-18	RABGEF1	RAB guanine nucleotide exchange factor (GEF)	Multiple_Complex
TC1600011505.hg.1	13.47	16.62	-8.86	7.50E-21	5.96E-18	NPIPB4	nuclear pore complex interacting protein fami	Multiple_Complex
TC0100015265.hg.1	7.06	11.99	-30.51	8.10E-21	6.21E-18	DENND2D	DENN/MADD domain containing 2D	Multiple_Complex
TC0900011177.hg.1	13.89	8.04	57.74	1.29E-20	9.57E-18	TXN	thioredoxin	Multiple_Complex
TC1500010184.hg.1	17.96	8.93	523.07	1.40E-20	9.99E-18	BCL2A1	BCL2-related protein A1	Coding
TC1500009056.hg.1	8.22	12.22	-16.07	1.46E-20	1.01E-17	RASGRP1	RAS guanyl releasing protein 1 (calcium and D	Multiple_Complex
TC0100014349.hg.1	8.84	4.21	24.82	1.52E-20	1.02E-17	JUN	jun proto-oncogene	Multiple_Complex
TC1300007161.hg.1	10.01	7.23	6.87	1.58E-20	1.02E-17	CDADC1	cytidine and dCMP deaminase domain contair	Multiple_Complex
TC0200008536.hg.1	6.63	10.4	-13.63	1.77E-20	1.12E-17	ANKRD36	Transcript Identified by AceView, Entrez Gene	Unassigned
TC0X00009256.hg.1	11.31	8.49	7.09	2.89E-20	1.77E-17	KLHL15	kelch-like family member 15	Coding
TC0500012842.hg.1	18.31	11.63	102.49	3.49E-20	2.08E-17	DUSP1	dual specificity phosphatase 1	Multiple_Complex
TC0500013238.hg.1	9.38	5.92	10.95	4.62E-20	2.68E-17	ZMAT2	zinc finger, matrin-type 2	Coding
TC0300010770.hg.1	10.54	6.3	18.87	5.73E-20	3.24E-17	CSRNP1	cysteine-serine-rich nuclear protein 1	Multiple_Complex
TC0800010607.hg.1	17.86	12.31	46.77	7.43E-20	4.09E-17	ASPH	aspartate beta-hydroxylase	Multiple_Complex
TC2100007140.hg.1	14.08	9.69	21.03	1.13E-19	6.07E-17	ETS2	v-ets avian erythroblastosis virus E26 oncoger	Multiple_Complex
TC0700006928.hg.1	12.54	9.02	11.48	1.20E-19	6.30E-17	CCDC126	coiled-coil domain containing 126	Multiple_Complex
TC0100007832.hg.1	11.45	8.37	8.45	1.62E-19	8.28E-17	ZC3H12A; M	zinc finger CCCH-type containing 12A; microRI	Multiple_Complex
TC1900008881.hg.1	13.78	8.56	37.47	1.84E-19	9.19E-17	FCAR	Fc fragment of IgA receptor	Multiple_Complex
TC1900008496.hg.1	11.46	7.21	19.07	2.04E-19	9.93E-17	PPP1R15A	protein phosphatase 1, regulatory subunit 15A	Multiple_Complex
TC1100009200.hg.1	11.02	15.93	-30.08	2.48E-19	1.18E-16	CD3E	CD3e molecule, epsilon (CD3-TCR complex)	Multiple_Complex
TC1200012648.hg.1	14.3	9	39.28	3.70E-19	1.73E-16	MYL6	myosin light chain 6	Multiple_Complex
TC0100016135.hg.1	8.41	13.7	-39.17	4.57E-19	2.09E-16	SLAMF6	SLAM family member 6	Coding
TC0500012179.hg.1	11.32	8.04	9.69	4.79E-19	2.10E-16	ETF1	eukaryotic translation termination factor 1	Multiple_Complex
TC1600011501.hg.1	12.2	15.09	-7.43	4.81E-19	2.10E-16	NPIPB3	nuclear pore complex interacting protein fami	Multiple_Complex
TC0200012854.hg.1	16.23	13.11	8.71	7.76E-19	3.33E-16	RAB1A	RAB1A, member RAS oncogene family	Multiple_Complex
TC0700008360.hg.1	8.93	11.69	-6.74	8.06E-19	3.39E-16	CASD1	CAS1 domain containing 1	Multiple_Complex
TC1300009249.hg.1	7.74	11.64	-14.86	8.92E-19	3.66E-16	KLF12	Kruppel-like factor 12	Multiple_Complex
TC1600009958.hg.1	12.27	15.2	-7.62	9.06E-19	3.66E-16	NPIPB4	nuclear pore complex interacting protein fami	Multiple_Complex
TC0500009046.hg.1	4.31	7.94	-12.33	9.99E-19	3.97E-16	ADRB2	adrenoceptor beta 2, surface	Coding
TC1400008193.hg.1	8.42	12.27	-14.34	1.09E-18	4.23E-16	EVL	Enah/Vasp-like	Multiple_Complex
TC0100008094.hg.1	9.55	11.26	-3.28	1.18E-18	4.51E-16	RNF220	ring finger protein 220	Multiple_Complex
TC0500007138.hg.1	11.38	17.57	-72.95	1.34E-18	5.03E-16	IL7R	interleukin 7 receptor	Multiple_Complex
TC0100017072.hg.1	12.03	15.67	-12.43	1.49E-18	5.52E-16	ELK4	ELK4, ETS-domain protein (SRF accessory prot	Multiple_Complex
TC1400007706.hg.1	10.46	6.43	16.38	1.83E-18	6.64E-16	FOS	FBJ murine osteosarcoma viral oncogene hom	Multiple_Complex
TC0400012811.hg.1	8.31	10.67	-5.12	2.30E-18	8.21E-16	DCK	deoxycytidine kinase	Multiple_Complex
TC1900007096.hg.1	12.15	7.63	22.86	2.45E-18	8.60E-16	JUNB	jun B proto-oncogene	Multiple_Complex
TC0300009661.hg.1	12.7	8.08	24.51	2.83E-18	9.76E-16	B3GNT5	UDP-GlcNAc:betaGal beta-1,3-N-acetylglucosa	Multiple_Complex
TC2000009458.hg.1	6.38	10.64	-19.13	2.87E-18	9.76E-16	NFATC2	nuclear factor of activated T-cells, cytoplasmic	Multiple_Complex
TC1600007353.hg.1	11	13.74	-6.69	3.10E-18	1.04E-15	NPIPB8	nuclear pore complex interacting protein fami	Coding

TC1700011818.hg.1	9.02	6.6	5.37	3.42E-18	1.12E-15	JMJD6	jumonji domain containing 6	Multiple_Complex
TC1500010743.hg.1	10.36	7.91	5.46	3.44E-18	1.12E-15	MAPK6	mitogen-activated protein kinase 6	Multiple_Complex
TC1300006729.hg.1	10.15	6.61	11.64	3.73E-18	1.20E-15	POMP	proteasome maturation protein	Multiple_Complex
TC1000008961.hg.1	6.28	8.74	-5.52	3.95E-18	1.23E-15	NHLRC2	NHL repeat containing 2	Multiple_Complex
TC0300013380.hg.1	8.8	11.5	-6.5	3.96E-18	1.23E-15	ALG3	ALG3, alpha-1,3- mannosyltransferase	Multiple_Complex
TC2200009272.hg.1	7.68	10.4	-6.59	4.55E-18	1.39E-15	APOBEC3D	apolipoprotein B mRNA editing enzyme, cataly	Coding
TC1400010619.hg.1	18.03	14.48	11.72	4.72E-18	1.43E-15	HIF1A	hypoxia inducible factor 1, alpha subunit (basi	Multiple_Complex
TC2200007783.hg.1	9.01	6.41	6.04	4.81E-18	1.43E-15	PIM3	Pim-3 proto-oncogene, serine/threonine kinas	Multiple_Complex
TC2000009180.hg.1	10.58	7.68	7.49	5.72E-18	1.68E-15	OSER1	oxidative stress responsive serine-rich 1	Coding
TC1700010630.hg.1	9.73	14.91	-36.37	5.81E-18	1.68E-15	CCR7	chemokine (C-C motif) receptor 7	Coding
TC0100013348.hg.1	10.61	6.85	13.54	6.81E-18	1.95E-15	SYF2	SYF2 pre-mRNA-splicing factor	Multiple_Complex
TC1200010968.hg.1	8.18	5.27	7.53	7.41E-18	2.08E-15	DDIT3	DNA-damage-inducible transcript 3	Coding
TC0700013605.hg.1	12.52	8.56	15.58	7.47E-18	2.08E-15	PNPLA8	patatin-like phospholipase domain containing	Multiple_Complex
TC2200009271.hg.1	10.94	14.46	-11.47	7.88E-18	2.17E-15	APOBEC3C	apolipoprotein B mRNA editing enzyme, cataly	Multiple_Complex
TC1200010850.hg.1	6.8	11.24	-21.79	8.16E-18	2.22E-15	TESPA1	thymocyte expressed, positive selection assoc	Multiple_Complex
TC1900011657.hg.1	11.44	7.33	17.31	8.65E-18	2.32E-15	MCEMP1	mast cell-expressed membrane protein 1	Multiple_Complex
TC1300009765.hg.1	11.49	6.72	27.3	9.08E-18	2.40E-15	IRS2	insulin receptor substrate 2	Multiple_Complex
TC0600008863.hg.1	7.07	10.56	-11.28	1.02E-17	2.68E-15	MANEA	mannosidase, endo-alpha	Multiple_Complex
TC0200008556.hg.1	6.94	9.93	-7.97	1.10E-17	2.84E-15	ZAP70	zeta chain of T cell receptor associated proteir	Multiple_Complex
TC0800011334.hg.1	13.58	9.84	13.36	1.11E-17	2.84E-15	KLF10	Kruppel-like factor 10	Multiple_Complex
TC1800007905.hg.1	9.56	11.92	-5.15	1.25E-17	3.15E-15	LPIN2	lipin 2	Multiple_Complex
TC0400009936.hg.1	11.45	8.74	6.53	1.26E-17	3.15E-15	GRPEL1	GrpE-like 1, mitochondrial (E. coli)	Multiple_Complex
TC1100011602.hg.1	10.6	14.27	-12.77	1.48E-17	3.65E-15	UCP2	uncoupling protein 2 (mitochondrial, proton c	Multiple_Complex
TC1700011121.hg.1	16.11	13.25	7.24	1.50E-17	3.65E-15	TOB1	transducer of ERBB2, 1	Multiple_Complex
TC2200008637.hg.1	7.81	11.37	-11.78	1.61E-17	3.89E-15	IL2RB	interleukin 2 receptor, beta	Multiple_Complex
TC0700013491.hg.1	7.69	11.77	-16.83	1.76E-17	4.19E-15	GIMAP1-GI	GIMAP1-GIMAP5 readthrough; GTPase, IMAP	Multiple_Complex
TC0600011173.hg.1	11.99	15.08	-8.53	2.02E-17	4.77E-15	GUSBP2	glucuronidase, beta pseudogene 2	Multiple_Complex
TC1300009330.hg.1	8.02	10.35	-5.01	2.23E-17	5.20E-15	RBM26	RNA binding motif protein 26	Multiple_Complex
TC1000011669.hg.1	11.17	13.85	-6.41	2.75E-17	6.34E-15	LDB1	LIM domain binding 1	Multiple_Complex
TC1000010870.hg.1	14.05	9.02	32.7	3.20E-17	7.31E-15	SRGN	Memczak2013 ANTISENSE, coding, INTERNAL,	NonCoding
TC0300011124.hg.1	6.21	10.25	-16.39	3.30E-17	7.46E-15	CISH	cytokine inducible SH2-containing protein	Multiple_Complex
TC0200010362.hg.1	11.61	9.03	5.99	3.38E-17	7.54E-15	COQ10B	coenzyme Q10B	Multiple_Complex
TC1100007394.hg.1	11.53	9.12	5.32	3.42E-17	7.55E-15	CD82	CD82 molecule	Multiple_Complex
TC0700008494.hg.1	8.89	6.03	7.25	3.52E-17	7.71E-15	BUD31	BUD31 homolog	Multiple_Complex
TC1200012818.hg.1	7.98	5.83	4.43	3.80E-17	8.22E-15	LLPH	LLP homolog, long-term synaptic facilitation (/	Coding
TC2200008859.hg.1	10.65	6.6	16.6	4.28E-17	9.19E-15	NDUFA6	NADH dehydrogenase (ubiquinone) 1 alpha su	Multiple_Complex
TC1400007161.hg.1	5.19	8.73	-11.69	4.35E-17	9.23E-15	PTGDR	prostaglandin D2 receptor (DP)	Coding
TC0200007895.hg.1	14.19	11.4	6.91	5.00E-17	1.05E-14	PLEK	pleckstrin	Multiple_Complex
TC0700013594.hg.1	6.68	9.18	-5.63	6.35E-17	1.32E-14	TRIM4	tripartite motif containing 4	Multiple_Complex
TC1600009916.hg.1	11.05	13.93	-7.37	6.60E-17	1.36E-14	NPIPB11	nuclear pore complex interacting protein fami	Coding
TC1700009398.hg.1	7.25	4.58	6.35	7.10E-17	1.45E-14	MIR22HG	MIR22 host gene	Multiple_Complex
TC1100006831.hg.1	12.24	8.38	14.52	8.07E-17	1.63E-14	ADM	adrenomedullin	Coding
TC1500009514.hg.1	6.97	9.42	-5.46	8.12E-17	1.63E-14	RFX7	regulatory factor X, 7	Multiple_Complex
TC0200011889.hg.1	15.7	13.55	4.43	9.17E-17	1.82E-14	LAPTM4A	lysosomal protein transmembrane 4 alpha	Multiple_Complex
TC1800006656.hg.1	13.65	11.2	5.46	9.89E-17	1.94E-14	VAPA	VAMP associated protein A	Multiple_Complex
TC0100015856.hg.1	17.79	9.61	289.86	9.97E-17	1.94E-14	S100A12	S100 calcium binding protein A12	Coding
TC0300007335.hg.1	10.06	7.74	4.99	1.01E-16	1.95E-14	TMA7	translation machinery associated 7 homolog	Multiple_Complex
TC1200011791.hg.1	12.72	8.76	15.57	1.06E-16	2.03E-14	CKAP4	cytoskeleton-associated protein 4	Multiple_Complex
TC0200007176.hg.1	7.61	9.62	-4.02	1.14E-16	2.16E-14	SLC30A6	solute carrier family 30 (zinc transporter), mer	Multiple_Complex
TC1200010837.hg.1	7.2	3.98	9.35	1.21E-16	2.27E-14	GPR84	G protein-coupled receptor 84	Coding
TC0800007419.hg.1	9.69	7.56	4.37	1.23E-16	2.30E-14	GPAT4	glycerol-3-phosphate acyltransferase 4	Multiple_Complex
TC0700010005.hg.1	9.48	7.19	4.88	1.26E-16	2.33E-14	ZFAND2A	zinc finger, AN1-type domain 2A	Multiple_Complex
TC0X00007704.hg.1	9.91	6.02	14.86	1.28E-16	2.35E-14	COX7B	cytochrome c oxidase subunit VIIb	Multiple_Complex
TC0600011661.hg.1	11.17	13.16	-3.99	1.35E-16	2.45E-14	STK38	serine/threonine kinase 38	Multiple_Complex
TC0600011644.hg.1	8.72	6	6.57	1.37E-16	2.46E-14	SRPK1	SRSF protein kinase 1	Multiple_Complex
TC1700010497.hg.1	9.31	12.05	-6.69	1.41E-16	2.52E-14	SYNRG	synergin, gamma	Multiple_Complex
TC0100008152.hg.1	8.02	4.78	9.42	1.50E-16	2.65E-14	UQCRH	ubiquinol-cytochrome c reductase hinge prote	Multiple_Complex
TC0X00011139.hg.1	9.9	6.74	8.93	1.60E-16	2.81E-14	CETN2	centrin 2	Multiple_Complex
TC1000011050.hg.1	8.64	10.66	-4.06	1.61E-16	2.81E-14	AP3M1	adaptor-related protein complex 3, mu 1 subu	Multiple_Complex
TC1600010179.hg.1	11.64	9.33	4.98	1.76E-16	3.05E-14	DNAJA2	DnaJ (Hsp40) homolog, subfamily A, member	Multiple_Complex
TC0400010167.hg.1	8.51	10.57	-4.18	2.02E-16	3.47E-14	DCAF16	DDB1 and CUL4 associated factor 16	Multiple_Complex
TC0300009815.hg.1	9.33	11.61	-4.85	2.29E-16	3.88E-14	LPP	LIM domain containing preferred translocatio	Multiple_Complex
TC0900010607.hg.1	12.33	9.4	7.62	2.30E-16	3.88E-14	AGTPBP1	ATP/GTP binding protein 1	Multiple_Complex
TC0700007477.hg.1	8.66	6.65	4.02	2.58E-16	4.32E-14	UPP1	uridine phosphorylase 1	Multiple_Complex
TC1900010856.hg.1	12.81	8.74	16.78	2.98E-16	4.95E-14	PLAUR	plasminogen activator, urokinase receptor	Multiple_Complex
TC0X00010473.hg.1	15.06	11.85	9.21	3.14E-16	5.18E-14	TSC22D3	TSC22 domain family, member 3	Coding
TC0X00006625.hg.1	5.98	10.49	-22.86	3.19E-16	5.22E-14	TLR7	toll-like receptor 7	Multiple_Complex
TC0200015447.hg.1	12.85	10.11	6.68	3.43E-16	5.58E-14	SUMO1	small ubiquitin-like modifier 1	Multiple_Complex
TC1000007925.hg.1	13.79	15.96	-4.51	3.59E-16	5.78E-14	EIF4EBP2	eukaryotic translation initiation factor 4E bind	Multiple_Complex
TC1000009296.hg.1	10.1	7.7	5.3	3.69E-16	5.90E-14	PTPRE	protein tyrosine phosphatase, receptor type, E	Multiple_Complex

TC0300010566.hg.1	6.22	9.01	-6.87	3.72E-16	5.91E-14	EOMES	eomesodermin	Coding
TC0100015236.hg.1	10.09	7.01	8.44	4.16E-16	6.57E-14	LAMTOR5	late endosomal/lysosomal adaptor, MAPK and	Multiple_Complex
TC2100007254.hg.1	6.89	9.56	-6.36	4.21E-16	6.59E-14	UBASH3A	ubiquitin associated and SH3 domain containi	Multiple_Complex
TC0300011936.hg.1	10.86	13.21	-5.08	4.30E-16	6.69E-14	CD47	CD47 molecule	Multiple_Complex
TC1200012571.hg.1	8.66	11.39	-6.64	4.37E-16	6.75E-14	ITFG2	integrin alpha FG-GAP repeat containing 2	Multiple_Complex
TC0500008157.hg.1	11.29	13.58	-4.92	4.41E-16	6.75E-14	LNPEP	leucyl/cystinyl aminopeptidase	Multiple_Complex
TC1100009191.hg.1	10.25	12.7	-5.46	4.55E-16	6.92E-14	IL10RA	interleukin 10 receptor, alpha	Multiple_Complex
TC1900010651.hg.1	16.97	11.62	40.77	4.74E-16	7.16E-14	GMFG	glia maturation factor, gamma	Multiple_Complex
TC0100007552.hg.1	9.05	6.77	4.86	4.81E-16	7.22E-14	SESN2	sestrin 2	Multiple_Complex
TC1600011417.hg.1	11.02	13.23	-4.64	4.95E-16	7.37E-14	NFATC3	nuclear factor of activated T-cells, cytoplasmic	Multiple_Complex
TC0200008663.hg.1	16.47	9.51	123.89	5.18E-16	7.66E-14	IL1R2	interleukin 1 receptor, type II	Multiple_Complex
TC1200012583.hg.1	7.86	11.57	-13.07	5.30E-16	7.79E-14	CD27	CD27 molecule	Multiple_Complex
TC1700009637.hg.1	5.63	9.27	-12.4	5.34E-16	7.79E-14	PLSCR3; TM	phospholipid scramblase 3; transmembrane p	Multiple_Complex
TC0700008786.hg.1	10.13	7.84	4.88	6.20E-16	8.98E-14	DNAJB9	DnaJ (Hsp40) homolog, subfamily B, member	Multiple_Complex
TC1200009888.hg.1	8.37	4.74	12.37	6.28E-16	9.02E-14	CLEC1B	C-type lectin domain family 1, member B	Coding
TC0400012432.hg.1	12.25	7.48	27.28	6.31E-16	9.02E-14	HMGB2	high mobility group box 2	Multiple_Complex
TC1700008867.hg.1	9.74	12.2	-5.49	6.38E-16	9.06E-14	SLC9A3R1;	solute carrier family 9, subfamily A (NHE3, cat	Multiple_Complex
TC0400011013.hg.1	17.59	10.88	104.26	6.79E-16	9.58E-14	PPBP	pro-platelet basic protein	Coding
TC0300011259.hg.1	7.32	9.79	-5.57	8.34E-16	1.17E-13	ARHGEF3	Rho guanine nucleotide exchange factor 3	Multiple_Complex
TC0X00008259.hg.1	8.1	5.32	6.86	8.43E-16	1.17E-13	NDUFA1	NADH dehydrogenase (ubiquinone) 1 alpha su	Multiple_Complex
TC1600011364.hg.1	12.03	14.42	-5.27	9.94E-16	1.38E-13	NPIPB5	nuclear pore complex interacting protein fami	Multiple_Complex
TC1700012353.hg.1	8.48	5.44	8.24	1.04E-15	1.42E-13	PER1; MIR6	period circadian clock 1; microRNA 6883	Multiple_Complex
TC0200010502.hg.1	8.68	11.55	-7.33	1.04E-15	1.42E-13	FAM117B	family with sequence similarity 117, member	Multiple_Complex
TC1200008675.hg.1	10.03	8.42	3.05	1.04E-15	1.42E-13	TXNRD1	thioredoxin reductase 1	Multiple_Complex
TC1500009079.hg.1	12.1	7.95	17.74	1.06E-15	1.43E-13	SRP14	signal recognition particle 14kDa	Multiple_Complex
TC0100010032.hg.1	19.48	14.93	23.47	1.06E-15	1.43E-13	S100A9	S100 calcium binding protein A9	Multiple_Complex
TC0200009074.hg.1	7.19	10.15	-7.79	1.10E-15	1.47E-13	PTPN4	protein tyrosine phosphatase, non-receptor ty	Multiple_Complex
TC0700008351.hg.1	8.63	4.75	14.69	1.15E-15	1.53E-13	GNG11	guanine nucleotide binding protein (G protein	Multiple_Complex
TC0500008684.hg.1	6.54	10.32	-13.72	1.23E-15	1.62E-13	TCF7	transcription factor 7 (T-cell specific, HMG-bo:	Multiple_Complex
TC0700009886.hg.1	12.21	9.91	4.91	1.26E-15	1.64E-13	DNAJB6	DnaJ (Hsp40) homolog, subfamily B, member	Multiple_Complex
TC1000012567.hg.1	9.01	11	-3.96	1.30E-15	1.69E-13	AGAP5	ArfGAP with GTPase domain, ankyrin repeat a	Multiple_Complex
TC0100016694.hg.1	12.13	9.09	8.26	1.38E-15	1.78E-13	IVNS1ABP	influenza virus NS1A binding protein	Multiple_Complex
TC0600008099.hg.1	5.24	7.14	-3.72	1.48E-15	1.90E-13	POLH	polymerase (DNA directed), eta	Multiple_Complex
TC1600006658.hg.1	9.34	13.33	-15.84	1.52E-15	1.94E-13	IL32	interleukin 32	Multiple_Complex
TC0100007943.hg.1	11.3	9.28	4.04	1.62E-15	2.05E-13	RLF	rearranged L-myc fusion	Multiple_Complex
TC1000007598.hg.1	8.22	10.84	-6.18	1.68E-15	2.13E-13	AGAP6	ArfGAP with GTPase domain, ankyrin repeat a	Multiple_Complex
TC0200008268.hg.1	10.71	16.25	-46.55	1.76E-15	2.21E-13	GNLY	granulysin	Multiple_Complex
TC0100018257.hg.1	12.92	10.53	5.24	1.92E-15	2.39E-13	GNAI3	guanine nucleotide binding protein (G protein	Multiple_Complex
TC0800007127.hg.1	6.46	9.88	-10.67	1.93E-15	2.40E-13	ELP3	elongator acetyltransferase complex subunit 3	Multiple_Complex
TC0500012210.hg.1	9.46	11.5	-4.12	2.01E-15	2.48E-13	TMEM173	transmembrane protein 173	Multiple_Complex
TC0200012072.hg.1	9.56	12.07	-5.72	2.16E-15	2.64E-13	MPV17	MpV17 mitochondrial inner membrane protei	Multiple_Complex
TC2200007088.hg.1	14.08	10.33	13.43	2.18E-15	2.65E-13	LIMK2	LIM domain kinase 2	Multiple_Complex
TC1400010763.hg.1	4.23	6.37	-4.41	2.30E-15	2.79E-13	TMEM229E	transmembrane protein 229B	Coding
TC1700010548.hg.1	8.75	6.28	5.55	2.34E-15	2.82E-13	CWC25	CWC25 spliceosome-associated protein homo	Multiple_Complex
TC2000008985.hg.1	11.87	8.03	14.3	2.45E-15	2.93E-13	RBM39	RNA binding motif protein 39	Multiple_Complex
TC0200016727.hg.1	6.3	8.23	-3.83	2.73E-15	3.25E-13	IWS1	IWS1 homolog (S. cerevisiae)	Multiple_Complex
TC0200015314.hg.1	17.91	15.69	4.65	2.74E-15	3.25E-13	STK17B	serine/threonine kinase 17b	Multiple_Complex
TC1900006977.hg.1	11.59	8.59	8.02	2.81E-15	3.31E-13	ICAM1	intercellular adhesion molecule 1	Multiple_Complex
TC0700011944.hg.1	9.81	6.59	9.31	2.95E-15	3.46E-13	ZNF394	zinc finger protein 394	Multiple_Complex
TC1600009855.hg.1	10.55	13.21	-6.36	3.35E-15	3.90E-13	NPIPB6	nuclear pore complex interacting protein fami	Coding
TC0600013125.hg.1	7.78	11.43	-12.56	3.37E-15	3.91E-13	THEMIS	thymocyte selection associated	Coding
TC0100007283.hg.1	5.86	7.86	-3.99	3.50E-15	4.03E-13	ZBTB40	zinc finger and BTB domain containing 40	Multiple_Complex
TC0100009949.hg.1	10.85	7.66	9.13	3.54E-15	4.05E-13	PSMD4	proteasome 26S subunit, non-ATPase 4	Multiple_Complex
TC1600007037.hg.1	9.79	12.64	-7.21	3.57E-15	4.07E-13	NPIPA7	nuclear pore complex interacting protein fami	Coding
TC0100015246.hg.1	8.49	11.54	-8.28	3.86E-15	4.38E-13	KCNA3	potassium channel, voltage gated shaker relat	Multiple_Complex
TC1700011060.hg.1	6.31	9.72	-10.66	3.92E-15	4.42E-13	FAM117A	family with sequence similarity 117, member A	Multiple_Complex
TC0X00007573.hg.1	10.91	13.03	-4.34	4.04E-15	4.54E-13	OGT	O-linked N-acetylglucosamine (GlcNAc) transfe	Multiple_Complex
TC1900011847.hg.1	10.85	7.64	9.3	4.87E-15	5.44E-13	LRG1	leucine-rich alpha-2-glycoprotein 1	Multiple_Complex
TC1000009536.hg.1	12.3	8.75	11.72	4.95E-15	5.50E-13	IDI1	isopentenyl-diphosphate delta isomerase 1	Multiple_Complex
TC0200014672.hg.1	9.2	3.97	37.52	5.04E-15	5.58E-13	NR4A2	nuclear receptor subfamily 4, group A, membe	Multiple_Complex
TC0900006961.hg.1	12.19	7.72	22.2	5.76E-15	6.33E-13	DNAJA1	DnaJ (Hsp40) homolog, subfamily A, member	Multiple_Complex
TC1200010798.hg.1	7.87	10.29	-5.35	5.79E-15	6.33E-13	AAAS	achalasia, adrenocortical insufficiency, alacrim	Multiple_Complex
TC1500010778.hg.1	8.38	10.79	-5.29	5.95E-15	6.48E-13	IL16	interleukin 16	Multiple_Complex
TC0600013464.hg.1	4.9	7.33	-5.38	6.06E-15	6.57E-13	SHPRH	SNF2 histone linker PHD RING helicase, E3 ubi	Multiple_Complex
TC0700013043.hg.1	6.32	9.14	-7.08	6.29E-15	6.78E-13	GIMAP6	GTPase, IMAP family member 6	Multiple_Complex
TC0500009690.hg.1	10.23	8.21	4.06	6.44E-15	6.91E-13	RUFY1	RUN and FYVE domain containing 1	Multiple_Complex
TC1200006771.hg.1	9.96	12.93	-7.83	6.53E-15	6.93E-13	CLEC2D	C-type lectin domain family 2, member D	Multiple_Complex
TC1000009714.hg.1	6.75	9.57	-7.07	6.54E-15	6.93E-13	PRKCQ	protein kinase C, theta	Multiple_Complex
TC0X00007721.hg.1	5.68	9.17	-11.23	6.56E-15	6.93E-13	GPR174	G protein-coupled receptor 174	Coding
TC2200008427.hg.1	7.86	5.59	4.82	6.61E-15	6.95E-13	OSM	oncostatin M	Coding

TC1900009824.hg.1	12.32	9.69	6.2	7.17E-15	7.51E-13	DNAJB1	DnaJ (Hsp40) homolog, subfamily B, member	Multiple_Complex
TC1200008373.hg.1	5.94	8.32	-5.19	7.50E-15	7.81E-13	TMTC3	transmembrane and tetratricopeptide repeat	Multiple_Complex
TC1600011365.hg.1	9.31	11.75	-5.42	7.73E-15	8.01E-13	NPIPB9	nuclear pore complex interacting protein fami	Coding
TC1700008709.hg.1	10.69	13.29	-6.07	7.79E-15	8.04E-13	BPTF	bromodomain PHD finger transcription factor	Multiple_Complex
TC0100016138.hg.1	5.96	8.34	-5.19	7.96E-15	8.17E-13	SLAMF1	signaling lymphocytic activation molecule fam	Multiple_Complex
TC1700008917.hg.1	11.58	10.21	2.58	8.43E-15	8.61E-13	SAP30BP	SAP30 binding protein	Multiple_Complex
TC0300013960.hg.1	10.52	8.17	5.06	8.49E-15	8.63E-13	HIGD1A	HIG1 hypoxia inducible domain family, membe	Multiple_Complex
TC1000010509.hg.1	8.78	11.14	-5.13	8.81E-15	8.91E-13	AGAP4	ArfGAP with GTPase domain, ankyrin repeat a	Multiple_Complex
TC1200006738.hg.1	6.98	11.31	-20.05	8.87E-15	8.93E-13	KLRG1	killer cell lectin-like receptor subfamily G, mer	Multiple_Complex
TC0100009339.hg.1	11.52	9.28	4.73	9.37E-15	9.39E-13	TMEM167E	transmembrane protein 167B	Multiple_Complex
TC0400007933.hg.1	10.95	5.77	36.28	9.75E-15	9.72E-13	ANXA3	annexin A3	Multiple_Complex
TC0500012470.hg.1	13.42	17.11	-12.83	9.91E-15	9.84E-13	CD74	CD74 molecule, major histocompatibility com	Multiple_Complex
TC1500007829.hg.1	5.68	3.25	5.39	1.02E-14	1.01E-12	LOXL1	lysyl oxidase-like 1	Multiple_Complex
TC0100014964.hg.1	8.76	6.14	6.13	1.04E-14	1.02E-12	DNTTIP2	deoxynucleotidyltransferase, terminal, interac	Multiple_Complex
TSUnmapped00000341.h	4.77	7.08	-4.95	1.05E-14	1.03E-12	ZNF780A	zinc finger protein 780A	Coding
TC1200009876.hg.1	10.5	7.26	9.46	1.07E-14	1.04E-12	CD69	CD69 molecule	Multiple_Complex
TC1400008683.hg.1	12.71	10.15	5.89	1.09E-14	1.06E-12	RBM23	RNA binding motif protein 23	Multiple_Complex
TC0100008326.hg.1	6.78	9.53	-6.72	1.10E-14	1.07E-12	FAM159A	family with sequence similarity 159, member	Multiple_Complex
TC0600013794.hg.1	4.49	6.55	-4.18	1.14E-14	1.09E-12	AGPAT4	1-acylglycerol-3-phosphate O-acyltransferase	Multiple_Complex
TC1500010047.hg.1	9.26	11.53	-4.84	1.16E-14	1.11E-12	SCAMP2	secretory carrier membrane protein 2	Multiple_Complex
TC1100012225.hg.1	6.53	9.01	-5.58	1.28E-14	1.22E-12	NPAT	nuclear protein, ataxia-telangiectasia locus	Multiple_Complex
TC1700009137.hg.1	4.11	6.41	-4.93	1.46E-14	1.38E-12	RPTOR	regulatory associated protein of MTOR, compl	Multiple_Complex
TC0200012809.hg.1	15.56	12.43	8.73	1.60E-14	1.51E-12	PELI1	pellino E3 ubiquitin protein ligase 1	Multiple_Complex
TC0600011385.hg.1	7.58	4.99	5.99	1.62E-14	1.53E-12	FLOT1	Transcript Identified by AceView, Entrez Gene	Unassigned
TC1700010587.hg.1	7.27	9.98	-6.51	1.64E-14	1.53E-12	IKZF3	IKAROS family zinc finger 3	Multiple_Complex
TC1600009731.hg.1	5.95	8.43	-5.58	1.68E-14	1.56E-12	COG7	component of oligomeric golgi complex 7	Multiple_Complex
TC1100010946.hg.1	8.54	10.73	-4.57	1.73E-14	1.60E-12	LPXN	leupaxin	Multiple_Complex
TC2100007803.hg.1	10.72	8.96	3.39	1.74E-14	1.61E-12	APP	amyloid beta (A4) precursor protein	Multiple_Complex
TC0200007999.hg.1	5.92	8.42	-5.68	1.82E-14	1.68E-12	ZNF638	Transcript Identified by AceView, Entrez Gene	Coding
TC0500008384.hg.1	12.34	9.66	6.41	1.87E-14	1.70E-12	AP3S1	adaptor-related protein complex 3, sigma 1 su	Multiple_Complex
TC0800008943.hg.1	6.39	9.21	-7.05	1.87E-14	1.70E-12	PHF20L1	Transcript Identified by AceView, Entrez Gene	Unassigned
TC0X00006799.hg.1	15.91	10.26	50.23	1.91E-14	1.74E-12	SAT1	spermidine/spermine N1-acetyltransferase 1	Multiple_Complex
TC0300007739.hg.1	8.56	6.72	3.58	2.07E-14	1.87E-12	PSMC1	Homo sapiens proteasome (prosome, macrop	Multiple_Complex
TC1700009539.hg.1	15.25	18.14	-7.39	2.10E-14	1.89E-12	PFN1	profilin 1	Multiple_Complex
TC0800012410.hg.1	13.43	15.06	-3.09	2.13E-14	1.91E-12	WHSC1L1	Wolf-Hirschhorn syndrome candidate 1-like 1	Multiple_Complex
TC2200008703.hg.1	14.2	16.5	-4.91	2.16E-14	1.93E-12	DDX17	DEAD (Asp-Glu-Ala-Asp) box helicase 17	Multiple_Complex
TC0100017028.hg.1	13.9	11.59	4.96	2.21E-14	1.96E-12	PPP1R15B	protein phosphatase 1, regulatory subunit 15E	Coding
TC1000011904.hg.1	10.53	13.92	-10.53	2.24E-14	1.99E-12	ABLIM1	actin binding LIM protein 1	Multiple_Complex
TC1200012666.hg.1	5	7.4	-5.3	2.31E-14	2.04E-12	TMEM19	transmembrane protein 19	Multiple_Complex
TC1200011838.hg.1	5.68	8.56	-7.34	2.41E-14	2.12E-12	CMKLR1	chemerin chemokine-like receptor 1	Multiple_Complex
TC0200007067.hg.1	10.79	9.13	3.16	2.43E-14	2.13E-12	NRBP1	nuclear receptor binding protein 1	Multiple_Complex
TC0300008559.hg.1	8.06	11.45	-10.48	2.46E-14	2.15E-12	PARP15	poly(ADP-ribose) polymerase family member	Multiple_Complex
TC1000008800.hg.1	8.28	6.24	4.13	2.51E-14	2.18E-12	GSTO1	glutathione S-transferase omega 1	Multiple_Complex
TC0500013259.hg.1	9.21	11.42	-4.61	2.53E-14	2.19E-12	MFAP3	microfibrillar associated protein 3	Multiple_Complex
TC1600010772.hg.1	5.93	7.69	-3.4	2.86E-14	2.46E-12	VAC14	Vac14 homolog (S. cerevisiae)	Multiple_Complex
TC1300008517.hg.1	13.38	9.37	16.17	2.87E-14	2.46E-12	HMGB1	high mobility group box 1	Multiple_Complex
TC1600006888.hg.1	7.33	10.34	-8.08	2.98E-14	2.55E-12	CIITA	class II, major histocompatibility complex, trar	Multiple_Complex
TC0100008625.hg.1	12.32	8.85	11.04	3.12E-14	2.65E-12	PDE4B	phosphodiesterase 4B, cAMP-specific	Multiple_Complex
TC0300009789.hg.1	8.42	10.81	-5.27	3.17E-14	2.69E-12	ST6GAL1	ST6 beta-galactosamide alpha-2,6-sialyltranfe	Multiple_Complex
TC1200007461.hg.1	8.52	11.81	-9.82	3.24E-14	2.73E-12	PCED1B	PC-esterase domain containing 1B	Multiple_Complex
TC0200010438.hg.1	8.79	6.66	4.36	3.25E-14	2.73E-12	NDUFB3	NADH dehydrogenase (ubiquinone) 1 beta sub	Coding
TC0Y00006642.hg.1	14.89	12	7.39	3.33E-14	2.78E-12	TMSB4Y	thymosin beta 4, Y-linked	Coding
TC0700009604.hg.1	5.91	8.43	-5.74	3.34E-14	2.78E-12	ZNF398	zinc finger protein 398	Multiple_Complex
TC2000009216.hg.1	10.96	6.6	20.51	3.53E-14	2.93E-12	SLPI	secretory leukocyte peptidase inhibitor	Multiple_Complex
TC1000007876.hg.1	11.01	8.52	5.62	3.81E-14	3.16E-12	DDX21	DEAD (Asp-Glu-Ala-Asp) box helicase 21	Multiple_Complex
TC0600014264.hg.1	8.13	6.18	3.86	3.83E-14	3.16E-12	DDAH2	dimethylarginine dimethylaminohydrolase 2	Multiple_Complex
TC0700010925.hg.1	4.99	7.26	-4.81	3.93E-14	3.23E-12	DDX56	DEAD (Asp-Glu-Ala-Asp) box helicase 56	Multiple_Complex
TC1600010680.hg.1	6.68	8.35	-3.17	4.14E-14	3.39E-12	SLC7A6OS	solute carrier family 7, member 6 opposite str	Multiple_Complex
TC1200012417.hg.1	15.38	13.01	5.19	4.21E-14	3.43E-12	SLC15A4	solute carrier family 15 (oligopeptide transpor	Multiple_Complex
TC1200009742.hg.1	9.05	6.95	4.28	4.58E-14	3.72E-12	CHD4	chromodomain helicase DNA binding protein	Multiple_Complex
TC0700007505.hg.1	10.25	12.66	-5.32	4.59E-14	3.72E-12	IKZF1	IKAROS family zinc finger 1	Multiple_Complex
TC0100009555.hg.1	8.97	13.08	-17.38	4.84E-14	3.90E-12	CD2	CD2 molecule	Coding
TC0100010310.hg.1	7.95	10.64	-6.48	4.93E-14	3.96E-12	SLAMF7	SLAM family member 7	Multiple_Complex
TC0200016454.hg.1	8.26	5.58	6.4	4.95E-14	3.96E-12	KIAA1841	KIAA1841	Multiple_Complex
TC1900006955.hg.1	10.38	7.62	6.74	4.99E-14	3.98E-12	UBL5	ubiquitin-like 5	Multiple_Complex
TC0900010366.hg.1	9.26	6.96	4.94	5.04E-14	4.01E-12	KLF9	Kruppel-like factor 9	Multiple_Complex
TC0500009210.hg.1	6.97	9.95	-7.9	5.09E-14	4.03E-12	ITK	IL2-inducible T-cell kinase	Multiple_Complex
TC0700009675.hg.1	6.22	8.79	-5.93	5.11E-14	4.03E-12	GIMAP8	GTPase, IMAP family member 8	Multiple_Complex
TC0200010157.hg.1	6.93	9.36	-5.38	5.25E-14	4.13E-12	ITGA4	integrin alpha 4	Multiple_Complex
TC1900011312.hg.1	9.53	11.69	-4.45	5.32E-14	4.15E-12	ZNF611	zinc finger protein 611	Multiple_Complex

TC1600008005.hg.1	5.4	10.26	-29	5.33E-14	4.15E-12	ADGRG1	adhesion G protein-coupled receptor G1	Multiple_Complex
TC0600007544.hg.1	10.7	12.72	-4.04	5.34E-14	4.15E-12	TUBB	tubulin, beta class I	Multiple_Complex
TC0100011269.hg.1	5.43	7.82	-5.24	5.45E-14	4.22E-12	LINC00260	long intergenic non-protein coding RNA 260	Multiple_Complex
TC0100018246.hg.1	8.34	10.96	-6.13	5.47E-14	4.22E-12	LRRC8C	leucine rich repeat containing 8 family, memb	Multiple_Complex
TC0100009696.hg.1	5.73	7.37	-3.11	5.51E-14	4.24E-12	LOC388692	Homo sapiens uncharacterized LOC388692 (L(	NonCoding
TC0900012159.hg.1	7.81	5.93	3.68	5.58E-14	4.26E-12	DNAJC25	DnaJ (Hsp40) homolog, subfamily C , member	Multiple_Complex
TC0600011071.hg.1	13.15	14.68	-2.89	5.58E-14	4.26E-12	C6orf62	chromosome 6 open reading frame 62	Multiple_Complex
TC0X00008240.hg.1	11.9	8.61	9.81	5.64E-14	4.29E-12	PGRMC1	progesterone receptor membrane component	Multiple_Complex
TC0200008675.hg.1	13.82	10.1	13.14	5.71E-14	4.33E-12	IL18RAP	interleukin 18 receptor accessory protein	Multiple_Complex
TC0X00011309.hg.1	12.39	9.12	9.65	6.05E-14	4.57E-12	HNRNPH2	heterogeneous nuclear ribonucleoprotein H2	Coding
TC1000011749.hg.1	10.46	7.98	5.59	6.43E-14	4.84E-12	ITPRIP	inositol 1,4,5-trisphosphate receptor interacti	Multiple_Complex
TC1400010716.hg.1	5.49	7.93	-5.42	6.51E-14	4.88E-12	HOMEZ	homeobox and leucine zipper encoding	Multiple_Complex
TSUnmapped00000100.h	5.3	7.49	-4.54	6.64E-14	4.96E-12	ZNF780B	zinc finger protein 780B	Coding
TC2000009916.hg.1	13.66	11.48	4.53	6.77E-14	5.04E-12	TGIF2-C20c	TGIF2-C20orf24 readthrough	Multiple_Complex
TC2000009915.hg.1	13.49	11.48	4.02	6.88E-14	5.11E-12	C20orf24	chromosome 20 open reading frame 24	Multiple_Complex
TC0800009752.hg.1	14.72	12.29	5.42	7.12E-14	5.26E-12	ASAH1	N-acylsphingosine amidohydrolase (acid cerar	Multiple_Complex
TC0100018484.hg.1	17.6	15.16	5.44	7.38E-14	5.44E-12	MCL1	myeloid cell leukemia 1	Multiple_Complex
TC0300006580.hg.1	6.95	4.98	3.91	7.48E-14	5.50E-12	IRAK2	interleukin 1 receptor associated kinase 2	Multiple_Complex
TC0200012961.hg.1	10.21	12.52	-4.97	7.84E-14	5.74E-12	AAK1	AP2 associated kinase 1	Multiple_Complex
TC0400011524.hg.1	7.47	9.04	-2.96	7.93E-14	5.79E-12	TBCK	TBC1 domain containing kinase	Multiple_Complex
TC1100006485.hg.1	7.37	8.91	-2.91	8.07E-14	5.86E-12	TMEM80	transmembrane protein 80	Multiple_Complex
TC0200016424.hg.1	7.29	10.3	-8.08	8.08E-14	5.86E-12	LBH	limb bud and heart development	Multiple_Complex
TC0100009241.hg.1	10.54	14.57	-16.39	8.15E-14	5.89E-12	S1PR1	sphingosine-1-phosphate receptor 1	Multiple_Complex
TC0700012115.hg.1	4.81	7.19	-5.21	8.18E-14	5.89E-12	NAPEPLD	N-acyl phosphatidylethanolamine phospholipa	Multiple_Complex
TC0500007143.hg.1	5.73	7.48	-3.36	8.22E-14	5.90E-12	SKP2	S-phase kinase-associated protein 2, E3 ubiqu	Multiple_Complex
TC0900012162.hg.1	6.15	3.83	4.98	8.31E-14	5.94E-12	ORM1	orosomucoid 1	Multiple_Complex
TC0600011381.hg.1	12.12	9.63	5.64	8.54E-14	6.09E-12	FLOT1	flotillin 1	Multiple_Complex
TC1200007709.hg.1	12.19	9.21	7.9	8.66E-14	6.15E-12	PFDN5	prefoldin subunit 5	Multiple_Complex
TC0700011383.hg.1	9.79	5.4	20.94	8.73E-14	6.18E-12	SBDS	Shwachman-Bodian-Diamond syndrome	Multiple_Complex
TC1100013003.hg.1	7.54	10.03	-5.63	8.76E-14	6.18E-12	TMX2; C11	thioredoxin-related transmembrane protein 2	Multiple_Complex
TC0800009961.hg.1	10.77	6.31	22.14	8.85E-14	6.21E-12	CLU; MIR68	clusterin; microRNA 6843	Multiple_Complex
TC1100007217.hg.1	7.69	9.71	-4.06	8.87E-14	6.21E-12	QSER1	glutamine and serine rich 1	Multiple_Complex
TC0100013744.hg.1	8.09	9.71	-3.07	8.88E-14	6.21E-12	MEAF6	MYST/Esa1-associated factor 6	Multiple_Complex
TC1200012646.hg.1	7.07	9.75	-6.39	9.47E-14	6.59E-12	ESYT1	extended synaptotagmin-like protein 1	Multiple_Complex
TC0200012433.hg.1	6.38	8.7	-5.01	9.70E-14	6.73E-12	PREPL	prolyl endopeptidase-like	Multiple_Complex
TC0100016360.hg.1	5.97	9.21	-9.42	9.76E-14	6.75E-12	SELL	Memczak2013 ALT_ACCEPTOR, ALT_DONOR,	NonCoding
TC1600006842.hg.1	12.43	10.73	3.25	9.93E-14	6.85E-12	C16orf72	chromosome 16 open reading frame 72	Multiple_Complex
TC0600014277.hg.1	9.34	12.9	-11.79	1.05E-13	7.20E-12	HLA-DMA	major histocompatibility complex, class II, DM	Multiple_Complex
TC0300013949.hg.1	9.71	11.77	-4.19	1.11E-13	7.62E-12	SATB1	SATB homeobox 1	Multiple_Complex
TSUnmapped00000019.h	5.17	7.19	-4.07	1.12E-13	7.64E-12	DYRK1B	dual specificity tyrosine-(Y)-phosphorylation r	Coding
TC1500009641.hg.1	8.52	11.91	-10.44	1.14E-13	7.75E-12	RORA	RAR-related orphan receptor A	Multiple_Complex
TC1900007839.hg.1	13.62	15.84	-4.65	1.14E-13	7.75E-12	FXYD5	FXYD domain containing ion transport regulat	Multiple_Complex
TC1200009590.hg.1	6.15	8.5	-5.11	1.17E-13	7.89E-12	DCP1B	decapping mRNA 1B	Multiple_Complex
TC0500007668.hg.1	9.57	6.98	6.03	1.19E-13	8.04E-12	CDK7	cyclin-dependent kinase 7	Multiple_Complex
TC0X00008002.hg.1	10.21	7.26	7.72	1.20E-13	8.09E-12	NGFRAP1	nerve growth factor receptor (TNFRSF16) assc	Multiple_Complex
TC0600013300.hg.1	12.76	10.63	4.39	1.22E-13	8.18E-12	IFNGR1	interferon gamma receptor 1	Multiple_Complex
TC1700009556.hg.1	9.63	11.93	-4.9	1.27E-13	8.50E-12	SCIMP	SLP adaptor and CSK interacting membrane pr	Multiple_Complex
TC2000009944.hg.1	11.49	9.8	3.24	1.33E-13	8.84E-12	RTFDC1	replication termination factor 2 domain conta	Multiple_Complex
TC1500010020.hg.1	7.57	10.57	-7.97	1.35E-13	9.00E-12	UBL7	ubiquitin-like 7	Multiple_Complex
TC0600009744.hg.1	8.01	5.97	4.1	1.37E-13	9.04E-12	RAB32	RAB32, member RAS oncogene family	Multiple_Complex
TC1700011363.hg.1	8.32	6.13	4.56	1.39E-13	9.14E-12	NACA2	nascent polypeptide-associated complex alph	Coding
TC1400009104.hg.1	11.67	7.32	20.42	1.40E-13	9.22E-12	RPL36AL	ribosomal protein L36a-like	Coding
TC1700007585.hg.1	8.62	12.46	-14.37	1.41E-13	9.26E-12	SLFN5	schlafen family member 5	Multiple_Complex
TC0200009091.hg.1	12.66	9.97	6.44	1.53E-13	1.00E-11	RALB	v-ral simian leukemia viral oncogene homolog	Multiple_Complex
TC0100016887.hg.1	10.65	8.16	5.6	1.54E-13	1.00E-11	ZNF281	zinc finger protein 281	Multiple_Complex
TC1200010006.hg.1	16.57	13.15	10.68	1.58E-13	1.02E-11	PLBD1	phospholipase B domain containing 1	Multiple_Complex
TC0300009458.hg.1	10.44	8.29	4.44	1.60E-13	1.04E-11	SEC62	SEC62 homolog, preprotein translocation fact	Multiple_Complex
TC1500010923.hg.1	3.76	6.16	-5.29	1.64E-13	1.06E-11	DET1	de-etiolated homolog 1 (Arabidopsis)	Multiple_Complex
TC0900010886.hg.1	6.61	9.08	-5.57	1.66E-13	1.07E-11	PTCH1	patched 1	Multiple_Complex
TC1200009239.hg.1	9.08	7.26	3.53	1.77E-13	1.14E-11	KMT5A	lysine (K)-specific methyltransferase 5A	Multiple_Complex
TC0100011012.hg.1	7.88	4.32	11.75	1.80E-13	1.15E-11	RGS1	regulator of G-protein signaling 1	Multiple_Complex
TC0300011757.hg.1	7.54	3.87	12.72	1.82E-13	1.16E-11	PROS1	protein S (alpha)	Multiple_Complex
TC2000009971.hg.1	5.07	8.42	-10.2	1.88E-13	1.20E-11	SIRPG	signal-regulatory protein gamma	Multiple_Complex
TC0700010504.hg.1	4.63	6.72	-4.27	1.99E-13	1.26E-11	OSBPL3	oxysterol binding protein-like 3	Multiple_Complex
TC1500007513.hg.1	10.25	7.72	5.76	2.00E-13	1.26E-11	LACTB	lactamase, beta	Multiple_Complex
TC0500013316.hg.1	8.34	12.44	-17.12	2.10E-13	1.33E-11	CD180	CD180 molecule	Multiple_Complex
TC2000009023.hg.1	13.56	15.65	-4.27	2.22E-13	1.40E-11	SAMHD1	SAM domain and HD domain 1	Multiple_Complex
TC2000009504.hg.1	10.47	13.33	-7.29	2.24E-13	1.40E-11	ZNF217	zinc finger protein 217	Multiple_Complex
TC1600008407.hg.1	9.31	11.57	-4.8	2.31E-13	1.45E-11	NPIPB15	nuclear pore complex interacting protein fami	Multiple_Complex
TC1000007510.hg.1	7.86	9.99	-4.38	2.36E-13	1.47E-11	AGAP9; CT(	Salzman2013 ANNOTATED, CDS, coding, INTE	Multiple_Complex

TC1900011677.hg.1	7.03	8.77	-3.34	2.37E-13	1.47E-11	IL27RA	interleukin 27 receptor, alpha	Multiple_Complex
TC1000008727.hg.1	10.42	8.15	4.82	2.40E-13	1.49E-11	NFKB2	nuclear factor of kappa light polypeptide gene	Multiple_Complex
TC0400012782.hg.1	9.18	11.13	-3.88	2.45E-13	1.51E-11	STIM2	stromal interaction molecule 2	Multiple_Complex
TC0300006694.hg.1	9.83	11.93	-4.29	2.47E-13	1.52E-11	NR2C2	nuclear receptor subfamily 2, group C, membe	Multiple_Complex
TC0100015864.hg.1	11.4	9.26	4.4	2.51E-13	1.54E-11	S100A6	S100 calcium binding protein A6	Multiple_Complex
TC1000007883.hg.1	19.19	15.88	9.93	2.53E-13	1.55E-11	SRGN	serglycin	Multiple_Complex
TC1900009272.hg.1	11.34	13.86	-5.74	2.59E-13	1.58E-11	AES	amino-terminal enhancer of split	Multiple_Complex
TC1200006721.hg.1	10.86	6.96	14.99	2.59E-13	1.58E-11	CLEC4D	C-type lectin domain family 4, member D	Coding
TC0800012447.hg.1	14.42	12.12	4.93	2.64E-13	1.61E-11	AZIN1	antizyme inhibitor 1	Multiple_Complex
TC2200008477.hg.1	4.77	7.46	-6.44	2.66E-13	1.61E-11	PATZ1	POZ (BTB) and AT hook containing zinc finger	Multiple_Complex
TC0200014834.hg.1	7.97	10.06	-4.26	2.71E-13	1.64E-11	STK39	serine threonine kinase 39	Multiple_Complex
TC0900009274.hg.1	11.39	9.8	3.03	2.76E-13	1.66E-11	TUBB4B	tubulin, beta 4B class IVb	Multiple_Complex
TC1400010617.hg.1	9.62	13.05	-10.76	2.81E-13	1.69E-11	PRKCH	protein kinase C, eta	Multiple_Complex
TC2000007320.hg.1	8.88	10.6	-3.3	2.90E-13	1.74E-11	RALGAPB	Ral GTPase activating protein, beta subunit (n	Multiple_Complex
TC0100017528.hg.1	5.99	8.04	-4.12	2.99E-13	1.79E-11	PARP1	poly(ADP-ribose) polymerase 1	Multiple_Complex
TC0700012836.hg.1	6.62	8.4	-3.43	3.07E-13	1.83E-11	KIAA1147	KIAA1147	Multiple_Complex
TC0600014265.hg.1	14.36	12.02	5.07	3.08E-13	1.83E-11	CLIC1	chloride intracellular channel 1	Coding
TC0900006708.hg.1	7.2	8.72	-2.87	3.09E-13	1.83E-11	SNAPC3	small nuclear RNA activating complex polypep	Multiple_Complex
TC2100006968.hg.1	12.2	9.19	8.08	3.16E-13	1.87E-11	IFNGR2	Memczak2013 ALT_ACCEPTOR, ALT_DONOR,	NonCoding
TC1600008799.hg.1	11.88	13.88	-3.99	3.17E-13	1.87E-11	BANP	BTG3 associated nuclear protein	Multiple_Complex
TC0800010558.hg.1	7.31	10.62	-9.94	3.20E-13	1.88E-11	TOX	thymocyte selection-associated high mobility	Multiple_Complex
TC0500010869.hg.1	9.21	5.97	9.43	3.21E-13	1.88E-11	ELOVL7	ELOVL fatty acid elongase 7	Multiple_Complex
TC2100008000.hg.1	6.65	8.56	-3.74	3.22E-13	1.88E-11	GART	phosphoribosylglycinamide formyltransferase	Multiple_Complex
TC1500010736.hg.1	10.84	8.85	3.97	3.25E-13	1.89E-11	BLOC1S6	biogenesis of lysosomal organelles complex-1,	Multiple_Complex
TC1100011338.hg.1	5.63	7.9	-4.81	3.26E-13	1.89E-11	CDK2AP2	cyclin-dependent kinase 2 associated protein	Multiple_Complex
TC1600008627.hg.1	8.41	6.32	4.26	3.33E-13	1.93E-11	HSBP1	heat shock factor binding protein 1	Multiple_Complex
TC1900010612.hg.1	5.27	7.95	-6.39	3.39E-13	1.96E-11	MAP4K1	mitogen-activated protein kinase kinase kinas	Multiple_Complex
TC0100018323.hg.1	9.94	7.53	5.29	3.41E-13	1.96E-11	IER5	immediate early response 5	Coding
TC0300007257.hg.1	6.21	8.81	-6.03	3.42E-13	1.96E-11	CCR5	chemokine (C-C motif) receptor 5 (gene/pseu	Multiple_Complex
TC1900011940.hg.1	12.76	15.78	-8.14	3.51E-13	2.01E-11	ECH1	enoyl-CoA hydratase 1, peroxisomal	Multiple_Complex
TC2200007138.hg.1	13	17.67	-25.49	3.54E-13	2.02E-11	FBXO7	F-box protein 7	Multiple_Complex
TC0600009712.hg.1	11	13.29	-4.87	3.57E-13	2.04E-11	UTRN	utrophin	Multiple_Complex
TC1400010760.hg.1	7.28	5.07	4.61	3.60E-13	2.05E-11	ATP6V1D	ATPase, H+ transporting, lysosomal 34kDa, V1	Multiple_Complex
TC1200012843.hg.1	14.52	11.39	8.77	3.62E-13	2.05E-11	ARPC3	actin related protein 2/3 complex subunit 3	NonCoding
TC0100013205.hg.1	13.52	10.82	6.52	3.70E-13	2.09E-11	ECE1	endothelin converting enzyme 1	Multiple_Complex
TC1100010761.hg.1	10.1	12.17	-4.2	3.72E-13	2.10E-11	FNBP4	formin binding protein 4	Multiple_Complex
TC0700008141.hg.1	12.84	10.12	6.58	3.77E-13	2.12E-11	PTPN12	protein tyrosine phosphatase, non-receptor ty	Multiple_Complex
TC1200007070.hg.1	8.72	10.05	-2.5	3.87E-13	2.17E-11	ETNK1	ethanolamine kinase 1	Multiple_Complex
TC0200010713.hg.1	5.19	7.4	-4.63	3.91E-13	2.19E-11	ATIC	5-aminoimidazole-4-carboxamide ribonucleot	Multiple_Complex
TC0100010303.hg.1	3.93	6.37	-5.43	3.92E-13	2.19E-11	SLAMF6	Memczak2013 ANTISENSE, coding, INTERNAL,	NonCoding
TC1300009797.hg.1	6.85	5.57	2.43	4.04E-13	2.25E-11	RAB20	RAB20, member RAS oncogene family	Multiple_Complex
TC0400009217.hg.1	11.82	8.41	10.63	4.07E-13	2.26E-11	KLHL2	kelch-like family member 2	Multiple_Complex
TC1700012274.hg.1	13.57	7.49	67.85	4.11E-13	2.28E-11	ITGB3	integrin beta 3	Multiple_Complex
TC1900011755.hg.1	4.94	6.47	-2.89	4.20E-13	2.32E-11	ZNF226	zinc finger protein 226	Multiple_Complex
TC1200009800.hg.1	18.28	14.8	11.14	4.24E-13	2.34E-11	SLC2A3	solute carrier family 2 (facilitated glucose tran	Multiple_Complex
TC0100011487.hg.1	5.42	7.76	-5.06	4.29E-13	2.36E-11	TRAF5	TNF receptor-associated factor 5	Multiple_Complex
TC0800011815.hg.1	13.75	11.69	4.19	4.46E-13	2.45E-11	FAM49B	family with sequence similarity 49, member B	Multiple_Complex
TC0400011548.hg.1	9.88	13.56	-12.84	4.51E-13	2.47E-11	LEF1	lymphoid enhancer-binding factor 1	Multiple_Complex
TC0100009226.hg.1	9.09	10.78	-3.22	4.78E-13	2.61E-11	SLC30A7	solute carrier family 30 (zinc transporter), mer	Multiple_Complex
TC1100012976.hg.1	10.92	8.63	4.89	4.87E-13	2.65E-11	NUCB2	nucleobindin 2	Multiple_Complex
TC0600014337.hg.1	7.23	8.85	-3.07	4.98E-13	2.70E-11	GOPC	golgi-associated PDZ and coiled-coil motif con	Multiple_Complex
TC1000008351.hg.1	4.9	7.43	-5.81	4.99E-13	2.70E-11	MINPP1	multiple inositol-polyphosphate phosphatase	Multiple_Complex
TC1000012121.hg.1	9.88	8.01	3.65	5.02E-13	2.71E-11	OAT	ornithine aminotransferase	Multiple_Complex
TC0200015893.hg.1	9.16	12.12	-7.83	5.12E-13	2.76E-11	DOCK10	dedicator of cytokinesis 10	Multiple_Complex
TC0200015838.hg.1	8.15	9.8	-3.13	5.19E-13	2.79E-11	FARSB	phenylalanyl-tRNA synthetase beta subunit	Multiple_Complex
TC1200012644.hg.1	10.71	12.41	-3.23	5.28E-13	2.83E-11	PA2G4	proliferation-associated 2G4	Multiple_Complex
TC0700008857.hg.1	8.32	10.14	-3.52	5.42E-13	2.90E-11	MDFIC	MyoD family inhibitor domain containing	Multiple_Complex
TC0100006818.hg.1	10.21	7.66	5.84	5.49E-13	2.93E-11	PGD	phosphogluconate dehydrogenase	Multiple_Complex
TC0700013441.hg.1	13.72	10.31	10.65	5.57E-13	2.96E-11	IFRD1	interferon-related developmental regulator 1	Multiple_Complex
TC1400009395.hg.1	6.62	8.27	-3.12	5.61E-13	2.98E-11	SGPP1	sphingosine-1-phosphate phosphatase 1	Coding
TC1600011465.hg.1	6.37	8.66	-4.91	5.74E-13	3.04E-11	ERVK13-1	endogenous retrovirus group K13, member 1	Multiple_Complex
TC0400012763.hg.1	7.36	5.52	3.58	5.80E-13	3.06E-11	S100P	S100 calcium binding protein P	Multiple_Complex
TC1600006628.hg.1	8.25	10.33	-4.25	5.82E-13	3.07E-11	KCTD5	potassium channel tetramerization domain co	Multiple_Complex
TC1300007774.hg.1	8.03	10.15	-4.34	5.86E-13	3.08E-11	MBNL2	muscleblind-like splicing regulator 2	Multiple_Complex
TC0X00006631.hg.1	19.39	16.43	7.77	5.93E-13	3.11E-11	TMSB4X	thymosin beta 4, X-linked	Multiple_Complex
TC0100016166.hg.1	7.18	4.71	5.55	6.38E-13	3.34E-11	APOA2	apolipoprotein A-II	Multiple_Complex
TC0300008550.hg.1	10.92	6.88	16.41	6.44E-13	3.36E-11	CSTA	cystatin A (stefin A)	Multiple_Complex
TC0600011777.hg.1	17.19	13.5	12.89	6.64E-13	3.46E-11	TREM1	triggering receptor expressed on myeloid cells	Multiple_Complex
TC1900010017.hg.1	10.07	8.17	3.72	6.78E-13	3.52E-11	ELL	elongation factor RNA polymerase II	Multiple_Complex
TC0700013615.hg.1	10.53	12.23	-3.23	6.85E-13	3.55E-11	ZNF800	zinc finger protein 800	Multiple_Complex

TC1400010339.hg.1	12.08	9.84	4.72	6.89E-13	3.55E-11	C14orf2	chromosome 14 open reading frame 2	Multiple_Complex
TC0100014227.hg.1	10.36	7.08	9.71	6.90E-13	3.55E-11	MAGOH	mago homolog, exon junction complex core c	Multiple_Complex
TC1100009202.hg.1	7.96	11.32	-10.23	6.90E-13	3.55E-11	CD3G	CD3g molecule, gamma (CD3-TCR complex)	Multiple_Complex
TC0100010243.hg.1	8.68	13.12	-21.78	7.05E-13	3.62E-11	PYHIN1	pyrin and HIN domain family, member 1	Multiple_Complex
TC0500011672.hg.1	12.23	10.44	3.46	7.11E-13	3.64E-11	PJA2	praja ring finger 2, E3 ubiquitin protein ligase	Multiple_Complex
TC0700011770.hg.1	7.78	9.96	-4.55	7.12E-13	3.64E-11	KRIT1	KRIT1, ankyrin repeat containing	Multiple_Complex
TC0100016302.hg.1	6.8	9.14	-5.09	7.69E-13	3.92E-11	CD247	CD247 molecule	Multiple_Complex
TC1800009284.hg.1	11.12	8.92	4.58	7.79E-13	3.96E-11	C18orf32	chromosome 18 open reading frame 32	Multiple_Complex
TC1600011355.hg.1	8.56	10.37	-3.5	7.82E-13	3.96E-11	NPIPA1	nuclear pore complex interacting protein fami	Multiple_Complex
TC1400010612.hg.1	7.75	9.63	-3.7	8.00E-13	4.05E-11	PCNXL4	pecanex-like 4 (Drosophila)	Multiple_Complex
TC1100008041.hg.1	9.59	13.31	-13.17	8.06E-13	4.07E-11	CTSW	cathepsin W	Multiple_Complex
TC2000007083.hg.1	5.58	3.53	4.14	8.37E-13	4.21E-11	ID1	inhibitor of DNA binding 1, dominant negative	Multiple_Complex
TC0100017761.hg.1	5.94	7.94	-4	8.45E-13	4.24E-11	PCNXL2	pecanex-like 2 (Drosophila)	Multiple_Complex
TC0X00011310.hg.1	9.96	5.8	17.87	8.53E-13	4.27E-11	RPL36A-HN	RPL36A-HNRNPH2 readthrough	Coding
TC1400009998.hg.1	10.21	6.25	15.53	8.75E-13	4.37E-11	NDUFB1	NADH dehydrogenase (ubiquinone) 1 beta sul	Multiple_Complex
TC0300006714.hg.1	10.33	8.34	3.99	8.89E-13	4.44E-11	EAF1	ELL associated factor 1	Multiple_Complex
TC0900008441.hg.1	10.45	7.99	5.52	8.98E-13	4.47E-11	UGCG	UDP-glucose ceramide glucosyltransferase	Multiple_Complex
TC1200008182.hg.1	13.79	11.73	4.17	9.08E-13	4.51E-11	RAB21	RAB21, member RAS oncogene family	Multiple_Complex
TC1000011606.hg.1	9.68	7.17	5.69	9.13E-13	4.52E-11	BLOC1S2	biogenesis of lysosomal organelles complex-1,	Multiple_Complex
TC1600011487.hg.1	9.4	11.47	-4.22	9.14E-13	4.52E-11	NPIPA8	nuclear pore complex interacting protein fami	Coding
TC0100013882.hg.1	9.53	11.56	-4.09	9.44E-13	4.65E-11	FOXJ3	forkhead box J3	Multiple_Complex
TC1500006925.hg.1	11.14	6.99	17.81	9.68E-13	4.76E-11	THBS1	thrombospondin 1	Multiple_Complex
TC1600011520.hg.1	6.95	9.88	-7.61	9.93E-13	4.87E-11	1-Sep	septin 1	Multiple_Complex
TC2000009259.hg.1	6.01	8.11	-4.27	9.97E-13	4.88E-11	NCOA5	nuclear receptor coactivator 5	Multiple_Complex
TC1500010744.hg.1	5.94	3.93	4.01	1.05E-12	5.13E-11	GCOM1; M	GRINL1A complex locus 1; myocardial zonula a	Multiple_Complex
TC1900011951.hg.1	4.95	7.27	-4.99	1.07E-12	5.23E-11	ZNF780B	zinc finger protein 780B	Multiple_Complex
TC1600008007.hg.1	12.52	9.73	6.89	1.08E-12	5.23E-11	ADGRG3	adhesion G protein-coupled receptor G3	Multiple_Complex
TC0200016591.hg.1	12.1	9.7	5.29	1.08E-12	5.25E-11	BZW1	basic leucine zipper and W2 domains 1	Multiple_Complex
TC2000006559.hg.1	7.33	9.94	-6.1	1.09E-12	5.26E-11	CDC25B	cell division cycle 25B	Multiple_Complex
TC2200007406.hg.1	11.7	10.1	3.03	1.09E-12	5.26E-11	ATF4	activating transcription factor 4	Multiple_Complex
TC1100008049.hg.1	8.1	10.74	-6.24	1.09E-12	5.27E-11	BANF1	barrier to autointegration factor 1	Multiple_Complex
TC1700010190.hg.1	8.79	6.9	3.71	1.10E-12	5.29E-11	IFT20	intraflagellar transport 20	Multiple_Complex
TC1200007430.hg.1	8.62	6.67	3.88	1.11E-12	5.31E-11	SCAF11	Memczak2013 ANTISENSE, CDS, coding, INTEF	NonCoding
TC0100018496.hg.1	5.85	8.12	-4.83	1.11E-12	5.31E-11	PYGO2	pygopus family PHD finger 2	Coding
TC1300007491.hg.1	7.89	5.55	5.06	1.11E-12	5.31E-11	KLF5	Kruppel-like factor 5 (intestinal)	Multiple_Complex
TC2100006854.hg.1	9.69	5.68	16.08	1.15E-12	5.50E-11	MAP3K7CL	MAP3K7 C-terminal like	Multiple_Complex
TC0X00011277.hg.1	8.91	11.6	-6.49	1.17E-12	5.56E-11	CA5B	carbonic anhydrase VB, mitochondrial	Multiple_Complex
TC1200012764.hg.1	5.48	7.45	-3.92	1.18E-12	5.62E-11	PRH1; TAS2	proline-rich protein HaeIII subfamily 1; taste r	Multiple_Complex
TC0600012875.hg.1	10	12.35	-5.08	1.23E-12	5.81E-11	FYN	FYN proto-oncogene, Src family tyrosine kinas	Multiple_Complex
TC0300013090.hg.1	12	14.08	-4.22	1.24E-12	5.85E-11	PHC3	polyhomeotic homolog 3 (Drosophila)	Multiple_Complex
TC0900008790.hg.1	8.1	6.34	3.41	1.24E-12	5.85E-11	ZBTB43	zinc finger and BTB domain containing 43	Multiple_Complex
TC1900009432.hg.1	7.24	8.45	-2.33	1.26E-12	5.92E-11	DENND1C	DENN/MADD domain containing 1C	Multiple_Complex
TC0100011001.hg.1	15.05	10.95	17.2	1.29E-12	6.08E-11	RGS18	regulator of G-protein signaling 18	Multiple_Complex
TC0100016120.hg.1	5.91	7.84	-3.82	1.30E-12	6.10E-11	PIGM	phosphatidylinositol glycan anchor biosynthes	Coding
TC0700013327.hg.1	6.5	8.52	-4.06	1.32E-12	6.18E-11	CHST12	carbohydrate (chondroitin 4) sulfotransferase	Coding
TC0100014846.hg.1	10.52	7.68	7.17	1.33E-12	6.22E-11	GTF2B	general transcription factor IIB	Multiple_Complex
TC0800007738.hg.1	16.67	13.39	9.73	1.34E-12	6.25E-11	SDCBP	syndecan binding protein	Multiple_Complex
TC0700008450.hg.1	11.39	8.82	5.98	1.35E-12	6.25E-11	LMTK2	lemur tyrosine kinase 2	Multiple_Complex
TC2000007133.hg.1	12.91	11.43	2.8	1.35E-12	6.27E-11	MAPRE1	microtubule-associated protein, RP/EB family,	Multiple_Complex
TC1600008199.hg.1	6.96	8.61	-3.14	1.38E-12	6.36E-11	ZFP90	ZFP90 zinc finger protein	Multiple_Complex
TC1000012011.hg.1	11.85	8.77	8.48	1.38E-12	6.38E-11	RGS10	regulator of G-protein signaling 10	Multiple_Complex
TC0400009694.hg.1	7.08	5.28	3.47	1.39E-12	6.42E-11	ATP5I	ATP synthase, H+ transporting, mitochondrial	Multiple_Complex
TC2100007492.hg.1	11.73	10.29	2.72	1.41E-12	6.47E-11	U2AF1	U2 small nuclear RNA auxiliary factor 1	Multiple_Complex
TC0200009065.hg.1	9.29	6.51	6.85	1.44E-12	6.62E-11	DBI	diazepam binding inhibitor (GABA receptor m	Multiple_Complex
TC0800012287.hg.1	11.76	13.22	-2.75	1.46E-12	6.67E-11	LEPROTL1	leptin receptor overlapping transcript-like 1	Multiple_Complex
TC1100010962.hg.1	11.07	14.5	-10.74	1.52E-12	6.95E-11	MPEG1	macrophage expressed 1	Coding
TC0100009903.hg.1	8.61	10.48	-3.67	1.53E-12	6.95E-11	RPRD2	regulation of nuclear pre-mRNA domain conta	Multiple_Complex
TC0500010553.hg.1	17.21	18.92	-3.28	1.53E-12	6.95E-11	FYB	FYN binding protein	Multiple_Complex
TC0300007679.hg.1	12.06	13.86	-3.49	1.57E-12	7.13E-11	ATXN7	ataxin 7	Multiple_Complex
TC0600008632.hg.1	4.98	6.7	-3.3	1.59E-12	7.20E-11	BCKDHB	branched chain keto acid dehydrogenase E1, b	Multiple_Complex
TC1100008330.hg.1	7.06	9.45	-5.23	1.60E-12	7.21E-11	IL18BP	interleukin 18 binding protein	Multiple_Complex
TC0900012278.hg.1	6.29	7.88	-3.01	1.64E-12	7.37E-11	ST6GALNAC	ST6 (alpha-N-acetyl-neuraminyl-2,3-beta-gala	Multiple_Complex
TC0700012182.hg.1	16.15	12.33	14.14	1.69E-12	7.59E-11	NAMPT	nicotinamide phosphoribosyltransferase	Multiple_Complex
TC1400009839.hg.1	9.2	6.69	5.7	1.71E-12	7.68E-11	STON2	stonin 2	Multiple_Complex
TC0800011062.hg.1	10.15	6.84	9.92	1.73E-12	7.74E-11	NBN	Transcript Identified by AceView, Entrez Gene	Unassigned
TC1000007885.hg.1	12.85	10.24	6.11	1.76E-12	7.85E-11	SRGN	Transcript Identified by AceView, Entrez Gene	Coding
TC1100012186.hg.1	5.85	7.48	-3.09	1.78E-12	7.93E-11	MSANTD4	Myb/SANT-like DNA-binding domain containir	Multiple_Complex
TC0100015858.hg.1	19.54	11.66	236.45	1.80E-12	8.00E-11	S100A8	S100 calcium binding protein A8	Multiple_Complex
TC0200007049.hg.1	6.08	7.94	-3.64	1.81E-12	8.04E-11	TMEM214	transmembrane protein 214	Multiple_Complex
TC0700012131.hg.1	6.75	8.44	-3.22	1.82E-12	8.05E-11	ORC5	origin recognition complex subunit 5	Multiple_Complex

TC1300008840.hg.1	17.75	15.55	4.59	1.83E-12	8.10E-11	LCP1	lymphocyte cytosolic protein 1 (L-plastin)	Multiple_Complex
TC0600008663.hg.1	6.54	8.25	-3.25	1.84E-12	8.13E-11	DOPEY1	dopey family member 1	Multiple_Complex
TC1500007601.hg.1	4.5	6.21	-3.28	1.90E-12	8.36E-11	HACD3	3-hydroxyacyl-CoA dehydratase 3	Multiple_Complex
TC0500011502.hg.1	12.31	10.27	4.11	1.91E-12	8.37E-11	ELL2	elongation factor, RNA polymerase II, 2	Multiple_Complex
TC1700012470.hg.1	15.8	13.65	4.43	1.91E-12	8.37E-11	H3F3B	H3 histone, family 3B (H3.3B)	Multiple_Complex
TC0500007075.hg.1	14.81	9.85	31.12	1.92E-12	8.39E-11	SUB1	SUB1 homolog, transcriptional regulator	Multiple_Complex
TC1600008329.hg.1	8.37	5.45	7.59	1.92E-12	8.39E-11	HP	haptoglobin	Multiple_Complex
TC0100006723.hg.1	11.3	8.65	6.28	1.93E-12	8.39E-11	VAMP3	vesicle associated membrane protein 3	Multiple_Complex
TC1500009669.hg.1	9.12	10.6	-2.8	1.93E-12	8.39E-11	VPS13C	vacuolar protein sorting 13 homolog C (S. cere	Multiple_Complex
TC0200015264.hg.1	10.72	7.4	10.02	1.93E-12	8.40E-11	SDPR	serum deprivation response	Multiple_Complex
TC0X00010837.hg.1	10.19	14.12	-15.21	1.94E-12	8.40E-11	MBNL3	muscleblind-like splicing regulator 3	Multiple_Complex
TC0200010523.hg.1	7	9.64	-6.22	2.00E-12	8.63E-11	CD28	CD28 molecule	Coding
TC1100008955.hg.1	6.43	7.98	-2.93	2.00E-12	8.63E-11	AASDHPPT	aminoadipate-semialdehyde dehydrogenase-p	Multiple_Complex
TC1700009036.hg.1	8.1	9.98	-3.67	2.02E-12	8.71E-11	TMC8	transmembrane channel like 8	Multiple_Complex
TC1600011374.hg.1	9.48	11.1	-3.07	2.05E-12	8.79E-11	SPN	sialophorin	Multiple_Complex
TC0600011531.hg.1	5.59	7.67	-4.22	2.06E-12	8.82E-11	VPS52	vacuolar protein sorting 52 homolog (S. cerevi	Multiple_Complex
TC0900008345.hg.1	14.19	12.55	3.13	2.07E-12	8.88E-11	RAD23B	RAD23 homolog B, nucleotide excision repair	Multiple_Complex
TC1100012514.hg.1	7.96	9.74	-3.44	2.09E-12	8.91E-11	DPAGT1	dolichyl-phosphate (UDP-N-acetylglucosamine	Multiple_Complex
TC1700012089.hg.1	7.63	9.57	-3.84	2.09E-12	8.91E-11	DCXR	dicarbonyl/L-xylulose reductase	Multiple_Complex
TC0100016357.hg.1	8.52	5.37	8.88	2.13E-12	9.06E-11	SELP	selectin P (granule membrane protein 140kDa	Multiple_Complex
TC1100008505.hg.1	8.81	11.12	-4.97	2.13E-12	9.06E-11	EMSY	EMSY BRCA2-interacting transcriptional repres	Multiple_Complex
TC0700013426.hg.1	7.18	9.8	-6.15	2.17E-12	9.20E-11	PVRIG	poliovirus receptor related immunoglobulin d	Multiple_Complex
TC2200009273.hg.1	7.46	9.81	-5.13	2.21E-12	9.36E-11	APOBEC3F	apolipoprotein B mRNA editing enzyme, cataly	Multiple_Complex
TC1300009766.hg.1	9.17	6.04	8.76	2.24E-12	9.45E-11	IRS2	Transcript Identified by AceView, Entrez Gene	Unassigned
TC0100015951.hg.1	7.37	8.87	-2.83	2.27E-12	9.58E-11	SCAMP3	secretory carrier membrane protein 3	Multiple_Complex
TC0100015182.hg.1	10.74	8.92	3.53	2.29E-12	9.63E-11	TAF13	TAF13 RNA polymerase II, TATA box binding p	Multiple_Complex
TC1100012358.hg.1	6.19	8.73	-5.81	2.29E-12	9.63E-11	USP28	ubiquitin specific peptidase 28	Multiple_Complex
TC0200011688.hg.1	10.71	8.93	3.43	2.30E-12	9.63E-11	ADAM17	ADAM metallopeptidase domain 17	Multiple_Complex
TC1300009492.hg.1	7.42	8.64	-2.32	2.35E-12	9.81E-11	TGDS	TDP-glucose 4,6-dehydratase	Multiple_Complex
TC0500008308.hg.1	5.96	8.2	-4.74	2.36E-12	9.85E-11	WDR36	WD repeat domain 36	Multiple_Complex
TC0100007886.hg.1	8.77	6.82	3.87	2.40E-12	1.00E-10	NDUFS5	NADH dehydrogenase (ubiquinone) Fe-S prote	Multiple_Complex
TC1900007743.hg.1	7.38	9.32	-3.83	2.46E-12	1.02E-10	ZNF507	zinc finger protein 507	Multiple_Complex
TC1300008468.hg.1	6.95	8.58	-3.11	2.50E-12	1.04E-10	SLC46A3	solute carrier family 46, member 3	Multiple_Complex
TC0400009462.hg.1	7.25	5.25	4	2.52E-12	1.05E-10	ING2	inhibitor of growth family member 2	Coding
TC0700009676.hg.1	7.84	9.91	-4.18	2.53E-12	1.05E-10	GIMAP7	GTPase, IMAP family member 7	Coding
TC1100012037.hg.1	7.6	10.3	-6.5	2.55E-12	1.05E-10	MAML2	mastermind-like transcriptional coactivator 2	Multiple_Complex
TC1100011260.hg.1	6.19	8.21	-4.05	2.57E-12	1.06E-10	C11orf68	chromosome 11 open reading frame 68	Multiple_Complex
TSUnmapped00000054.h	4.69	7.18	-5.6	2.58E-12	1.06E-10	ZNF780B	zinc finger protein 780B	Coding
TSUnmapped00000177.h	4.69	7.18	-5.6	2.58E-12	1.06E-10	ZNF780B	zinc finger protein 780B	Coding
TC1100011192.hg.1	9.56	7.42	4.39	2.59E-12	1.06E-10	ATG2A	autophagy related 2A	Multiple_Complex
TC0200016581.hg.1	10.02	7.45	5.92	2.74E-12	1.12E-10	C2orf88	chromosome 2 open reading frame 88	Multiple_Complex
TC1100007049.hg.1	9.81	8	3.52	2.76E-12	1.12E-10	HTATIP2	HIV-1 Tat interactive protein 2	Multiple_Complex
TC0200014693.hg.1	15.79	13.73	4.17	2.80E-12	1.14E-10	CYTIP	cytohesin 1 interacting protein	Multiple_Complex
TC0700007178.hg.1	13.11	10.86	4.78	2.86E-12	1.16E-10	7-Sep	septin 7	Multiple_Complex
TC1700010830.hg.1	12.76	6.91	57.69	2.92E-12	1.18E-10	ITGA2B	integrin alpha 2b	Multiple_Complex
TC0500013166.hg.1	6.68	8.36	-3.21	2.93E-12	1.19E-10	ZNF131	zinc finger protein 131	NonCoding
TC0300010427.hg.1	8.01	10.26	-4.77	2.99E-12	1.21E-10	RFTN1	raftlin, lipid raft linker 1	Multiple_Complex
TC1100007785.hg.1	8.69	11.32	-6.21	3.01E-12	1.21E-10	TMEM109	transmembrane protein 109	Multiple_Complex
TC0200010214.hg.1	9.78	7.05	6.64	3.01E-12	1.21E-10	ZC3H15	zinc finger CCCH-type containing 15	Multiple_Complex
TC1200012584.hg.1	8.39	11.09	-6.5	3.07E-12	1.23E-10	TAPBPL	TAP binding protein-like	Multiple_Complex
TC0600010709.hg.1	11.51	7.85	12.6	3.16E-12	1.27E-10	F13A1	coagulation factor XIII, A1 polypeptide	Multiple_Complex
TC0200009440.hg.1	9.54	11.84	-4.91	3.28E-12	1.31E-10	R3HDM1	R3H domain containing 1	Multiple_Complex
TC2000010001.hg.1	5.05	6.57	-2.88	3.31E-12	1.32E-10	PIGU	phosphatidylinositol glycan anchor biosynthes	Multiple_Complex
TC1500009782.hg.1	6.69	8.88	-4.59	3.34E-12	1.33E-10	PDCD7	programmed cell death 7	Multiple_Complex
TC0300006925.hg.1	11.4	13.08	-3.21	3.34E-12	1.33E-10	TGFBR2	transforming growth factor beta receptor II	Multiple_Complex
TC0800008770.hg.1	11.37	9.73	3.13	3.48E-12	1.38E-10	RNF139	ring finger protein 139	Multiple_Complex
TC0300012918.hg.1	15.27	12.89	5.2	3.49E-12	1.38E-10	CCNL1	cyclin L1	Multiple_Complex
TC0300011988.hg.1	4.72	6.65	-3.83	3.51E-12	1.39E-10	GCSAM	germinal center-associated, signaling and mot	Coding
TC0700011554.hg.1	9.49	10.74	-2.37	3.52E-12	1.39E-10	PMS2P3	PMS1 homolog 2, mismatch repair system cor	Multiple_Complex
TC1100011372.hg.1	8.43	5.84	6.01	3.53E-12	1.39E-10	CHKA	choline kinase alpha	Multiple_Complex
TC0100006852.hg.1	8.46	10.56	-4.28	3.57E-12	1.40E-10	UBIAD1	UbiA prenyltransferase domain containing 1	Multiple_Complex
TC1100010705.hg.1	9.64	11.25	-3.05	3.57E-12	1.40E-10	AMBRA1	autophagy/beclin-1 regulator 1	Multiple_Complex
TSUnmapped00000670.h	5.45	7.56	-4.32	3.63E-12	1.42E-10	ZNF780A	zinc finger protein 780A	Coding
TC2200006855.hg.1	6.74	5.18	2.95	3.76E-12	1.47E-10	RGL4	ral guanine nucleotide dissociation stimulator	Multiple_Complex
TC0700008878.hg.1	8.79	6.17	6.16	3.87E-12	1.51E-10	CAPZA2	capping protein (actin filament) muscle Z-line,	Multiple_Complex
TC2200009283.hg.1	6.11	7.9	-3.46	3.93E-12	1.53E-10	PRR5	proline rich 5 (renal)	Multiple_Complex
TC0100012869.hg.1	5.9	8.08	-4.54	3.94E-12	1.54E-10	SRM	spermidine synthase	Multiple_Complex
TC0X00006697.hg.1	8.84	7.24	3.02	4.05E-12	1.57E-10	SYAP1	synapse associated protein 1	Multiple_Complex
TC0300012048.hg.1	9.66	11.88	-4.66	4.11E-12	1.60E-10	ZBTB20; M	zinc finger and BTB domain containing 20; mic	Multiple_Complex
TC0100016685.hg.1	5.95	7.57	-3.06	4.12E-12	1.60E-10	TRMT1L	tRNA methyltransferase 1 like	Multiple_Complex

TC0900007085.hg.1	9.18	10.92	-3.34	4.16E-12	1.61E-10	TESK1; MIR	testis-specific kinase 1; microRNA 4667	Multiple_Complex
TC0100011270.hg.1	5.94	8.34	-5.28	4.23E-12	1.63E-10	LAX1	lymphocyte transmembrane adaptor 1	Multiple_Complex
TC0600014259.hg.1	9.55	11.09	-2.9	4.27E-12	1.64E-10	ATP6V1G2-	ATP6V1G2-DDX39B readthrough (NMD candid	Multiple_Complex
TC0100012101.hg.1	7.21	8.95	-3.34	4.28E-12	1.65E-10	MTR	5-methyltetrahydrofolate-homocysteine meth	Multiple_Complex
TC0300008361.hg.1	6.22	7.79	-2.97	4.32E-12	1.66E-10	SIDT1	SID1 transmembrane family, member 1	Multiple_Complex
TC0600012763.hg.1	5.37	7.47	-4.29	4.42E-12	1.69E-10	SCML4	sex comb on midleg-like 4 (Drosophila)	Multiple_Complex
TC1900008252.hg.1	7.14	8.95	-3.49	4.46E-12	1.71E-10	ZNF224	zinc finger protein 224	Multiple_Complex
TC1100013001.hg.1	7.18	5.5	3.21	4.52E-12	1.73E-10	CLP1	cleavage and polyadenylation factor I subunit	Coding
TC1600007030.hg.1	9.17	10.83	-3.16	4.63E-12	1.76E-10	NPIPA7; NP	nuclear pore complex interacting protein fami	Multiple_Complex
TC1900011779.hg.1	7.65	10.37	-6.59	4.67E-12	1.77E-10	FLT3LG	fms-related tyrosine kinase 3 ligand	Multiple_Complex
TC1000011718.hg.1	7.7	10.14	-5.45	4.68E-12	1.78E-10	CALHM2	calcium homeostasis modulator 2	Multiple_Complex
TC0900010825.hg.1	10.44	8.64	3.48	4.70E-12	1.78E-10	NINJ1	ninjurin 1	Multiple_Complex
TC0100008664.hg.1	8.83	6.94	3.69	4.95E-12	1.87E-10	GADD45A	growth arrest and DNA-damage-inducible, alp	Multiple_Complex
TC1800008035.hg.1	12.83	10.59	4.72	4.98E-12	1.88E-10	PPP4R1	protein phosphatase 4, regulatory subunit 1	Multiple_Complex
TC0100013153.hg.1	5.56	7.23	-3.18	5.04E-12	1.90E-10	TMCO4	transmembrane and coiled-coil domains 4	Multiple_Complex
TC0200012161.hg.1	7.36	4.17	9.15	5.20E-12	1.96E-10	GALNT14	polypeptide N-acetylgalactosaminyltransferas	Multiple_Complex
TC1600009935.hg.1	7.85	10.88	-8.19	5.23E-12	1.96E-10	SPN	Memczak2013 ANTISENSE, CDS, coding, INTER	NonCoding
TC1100013193.hg.1	7.49	10.97	-11.1	5.28E-12	1.98E-10	PTPRCAP	protein tyrosine phosphatase, receptor type, (	Multiple_Complex
TC1200006555.hg.1	11.76	13.84	-4.22	5.30E-12	1.99E-10	CCND2	cyclin D2	Multiple_Complex
TC0100017793.hg.1	10.45	8.27	4.52	5.39E-12	2.01E-10	IRF2BP2	interferon regulatory factor 2 binding protein	Multiple_Complex
TC0200009938.hg.1	8.33	10.17	-3.59	5.40E-12	2.02E-10	GORASP2	golgi reassembly stacking protein 2	Multiple_Complex
TC1600008275.hg.1	7.47	9.04	-2.97	5.47E-12	2.04E-10	SF3B3	splicing factor 3b subunit 3	Multiple_Complex
TC2000007682.hg.1	10.19	8.55	3.11	5.50E-12	2.05E-10	CEBPB	CCAAT/enhancer binding protein (C/EBP), bet	Multiple_Complex
TC1900008166.hg.1	10.31	14.7	-20.97	5.53E-12	2.05E-10	CD79A	CD79a molecule, immunoglobulin-associated	Multiple_Complex
TC0400008947.hg.1	7.88	9.67	-3.46	5.65E-12	2.09E-10	TMEM184C	transmembrane protein 184C	Multiple_Complex
TC0200010643.hg.1	7.87	9.32	-2.72	5.68E-12	2.10E-10	RPE	ribulose-5-phosphate-3-epimerase	Multiple_Complex
TC0700006980.hg.1	11.24	9.08	4.49	5.69E-12	2.10E-10	CBX3	chromobox homolog 3	Multiple_Complex
TC1600011578.hg.1	6.28	7.47	-2.28	5.70E-12	2.10E-10	FBXO31	F-box protein 31	Multiple_Complex
TC1000006782.hg.1	7.36	4.85	5.68	5.81E-12	2.14E-10	ECHDC3	enoyl-CoA hydratase domain containing 3	Multiple_Complex
TC1300009505.hg.1	8.66	6.46	4.6	6.24E-12	2.29E-10	ABCC4	ATP binding cassette subfamily C member 4	Multiple_Complex
TC1800008655.hg.1	9.31	10.55	-2.36	6.26E-12	2.30E-10	DYM	dymeclin	Multiple_Complex
TC1900008180.hg.1	5.77	7.88	-4.33	6.32E-12	2.31E-10	ZNF526	zinc finger protein 526	Coding
TC0100010625.hg.1	5	6.9	-3.72	6.39E-12	2.33E-10	FASLG	Fas ligand (TNF superfamily, member 6)	Coding
TC0500012663.hg.1	9.33	7.3	4.08	6.42E-12	2.34E-10	SLU7	SLU7 homolog, splicing factor	Multiple_Complex
TC1000011892.hg.1	8.99	6.76	4.71	6.45E-12	2.35E-10	CCDC186; I	coiled-coil domain containing 186; microRNA	Multiple_Complex
TC1800006508.hg.1	14.26	11.44	7.03	6.49E-12	2.36E-10	MYL12B	myosin light chain 12B	Coding
TC0500012957.hg.1	9.55	7.75	3.46	6.68E-12	2.43E-10	UIMC1	ubiquitin interaction motif containing 1	Multiple_Complex
TC0300014043.hg.1	9.78	7.47	4.94	6.82E-12	2.47E-10	ISY1	ISY1 splicing factor homolog	Multiple_Complex
TC0400010784.hg.1	6.98	8.44	-2.75	6.84E-12	2.47E-10	NOA1	nitric oxide associated 1	Multiple_Complex
TC0200016403.hg.1	6.45	8.54	-4.28	6.99E-12	2.52E-10	LPIN1; MIR	lipin 1; microRNA 548s	Multiple_Complex
TC1900010707.hg.1	7.35	6.09	2.39	7.08E-12	2.55E-10	SERTAD3	SERTA domain containing 3	Multiple_Complex
TC1500007333.hg.1	7.77	9.52	-3.37	7.10E-12	2.56E-10	TCF12	transcription factor 12	Multiple_Complex
TC0600012544.hg.1	9.36	7.56	3.48	7.14E-12	2.57E-10	RRAGD	Ras-related GTP binding D	Multiple_Complex
TC1000010912.hg.1	12.02	10.74	2.43	7.24E-12	2.60E-10	SAR1A	secretion associated, Ras related GTPase 1A	Multiple_Complex
TC1500010757.hg.1	12.69	11.06	3.08	7.27E-12	2.60E-10	RAB11A	RAB11A, member RAS oncogene family	Multiple_Complex
TC0600013528.hg.1	9.43	11.53	-4.27	7.28E-12	2.60E-10	LATS1	large tumor suppressor kinase 1	Multiple_Complex
TC0900011769.hg.1	4.69	7.4	-6.57	7.36E-12	2.63E-10	FAM78A	family with sequence similarity 78, member A	Multiple_Complex
TC0300010933.hg.1	7.42	9.01	-3	7.43E-12	2.65E-10	FYCO1	FYVE and coiled-coil domain containing 1	Multiple_Complex
TC0800011171.hg.1	10.94	6.5	21.72	7.46E-12	2.65E-10	UQCRB	ubiquinol-cytochrome c reductase binding pro	Multiple_Complex
TC0300012812.hg.1	6.17	8.36	-4.55	7.60E-12	2.70E-10	GPR171	G protein-coupled receptor 171	Coding
TC0300010304.hg.1	8.16	6.07	4.25	7.65E-12	2.71E-10	TMEM40	transmembrane protein 40	Multiple_Complex
TC0400006951.hg.1	9.38	12.74	-10.27	7.71E-12	2.73E-10	CD38	CD38 molecule	Multiple_Complex
TC0100011022.hg.1	17.06	11.9	35.75	7.78E-12	2.74E-10	RGS2	regulator of G-protein signaling 2	Multiple_Complex
TC1500009492.hg.1	9.62	6.6	8.14	7.78E-12	2.74E-10	RSL24D1	ribosomal L24 domain containing 1	Multiple_Complex
TC0200013595.hg.1	9.44	11.56	-4.34	7.80E-12	2.75E-10	MGAT4A	mannosyl (alpha-1,3-)-glycoprotein beta-1,4-N	Multiple_Complex
TC0600014217.hg.1	13.72	11.28	5.42	8.01E-12	2.82E-10	SERPINB1	serpin peptidase inhibitor, clade B (ovalbumin	Multiple_Complex
TC1900007447.hg.1	8.04	9.92	-3.66	8.09E-12	2.84E-10	ZNF101	zinc finger protein 101	Multiple_Complex
TC0900011151.hg.1	5.33	6.94	-3.05	8.19E-12	2.87E-10	TMEM245	transmembrane protein 245	Multiple_Complex
TC1400007307.hg.1	10.91	8.83	4.22	8.19E-12	2.87E-10	ARID4A	AT rich interactive domain 4A (RBP1-like)	Multiple_Complex
TC0200008984.hg.1	6.51	7.9	-2.62	8.23E-12	2.88E-10	RABL2A	RAB, member of RAS oncogene family-like 2A	Multiple_Complex
TC1200008003.hg.1	10.84	8.78	4.18	8.29E-12	2.89E-10	TBK1	TANK-binding kinase 1	Multiple_Complex
TC0100018462.hg.1	7.79	9.09	-2.46	8.37E-12	2.91E-10	CLCC1	chloride channel CLIC-like 1	Multiple_Complex
TC0400009467.hg.1	7.64	9.46	-3.54	8.43E-12	2.93E-10	TRAPPC11	trafficking protein particle complex 11	Multiple_Complex
TC1100012984.hg.1	5	7.13	-4.39	8.55E-12	2.97E-10	NAT10	N-acetyltransferase 10 (GCN5-related)	Multiple_Complex
TC1000010925.hg.1	8.87	12.52	-12.59	8.65E-12	3.00E-10	PRF1	perforin 1 (pore forming protein)	Multiple_Complex
TC0500008855.hg.1	7.93	9.76	-3.56	9.06E-12	3.13E-10	SLC35A4	solute carrier family 35, member A4	Multiple_Complex
TC1800009229.hg.1	10.22	11.8	-2.99	9.07E-12	3.13E-10	SMAD4	SMAD family member 4	Multiple_Complex
TC0600011197.hg.1	5.81	7.35	-2.9	9.08E-12	3.13E-10	ZNF184	zinc finger protein 184	Multiple_Complex
TC1600009137.hg.1	6.44	4.87	2.98	9.15E-12	3.15E-10	TCEB2	transcription elongation factor B (SIII), polype	Multiple_Complex
TC1700007451.hg.1	12.55	9.76	6.89	9.20E-12	3.16E-10	CPD	carboxypeptidase D	Multiple_Complex

TC1200012803.hg.1	13.81	10.47	10.14	9.24E-12	3.17E-10	NACA	nascent polypeptide-associated complex alpha	Multiple_Complex
TC1000008385.hg.1	6.24	8.13	-3.72	9.26E-12	3.17E-10	STAMBPL1	STAM binding protein-like 1	Multiple_Complex
TC1000012582.hg.1	5.96	4.89	2.11	9.33E-12	3.19E-10	RRP12	ribosomal RNA processing 12 homolog	Multiple_Complex
TC1200008184.hg.1	10.72	7.97	6.73	9.38E-12	3.20E-10	TBC1D15	TBC1 domain family, member 15	Multiple_Complex
TC0400012048.hg.1	4.75	6.19	-2.71	9.48E-12	3.23E-10	ZNF827	zinc finger protein 827	Multiple_Complex
TC0300013842.hg.1	8.82	7.04	3.42	9.63E-12	3.28E-10	SLMAP	sarcolemma associated protein	Multiple_Complex
TC1900011925.hg.1	5.17	7.34	-4.52	9.70E-12	3.30E-10	ZNF91	zinc finger protein 91	NonCoding
TC0300010561.hg.1	7.51	9.21	-3.25	9.80E-12	3.33E-10	SLC4A7	solute carrier family 4, sodium bicarbonate co	Multiple_Complex
TC0300011516.hg.1	13.31	11.53	3.42	9.84E-12	3.33E-10	EIF4E3	eukaryotic translation initiation factor 4E fami	Multiple_Complex
TC0100015899.hg.1	19.32	17.88	2.7	9.91E-12	3.35E-10	TPM3	tropomyosin 3	Multiple_Complex
TC0100010609.hg.1	7.98	5.1	7.36	1.00E-11	3.38E-10	DNM3	dynamin 3	Multiple_Complex
TC0X00007031.hg.1	13.2	11.21	3.97	1.01E-11	3.39E-10	DDX3X	DEAD (Asp-Glu-Ala-Asp) box helicase 3, X-link	Multiple_Complex
TC0900011613.hg.1	5.4	8.51	-8.66	1.01E-11	3.40E-10	FAM102A	family with sequence similarity 102, member	Multiple_Complex
TC1900009588.hg.1	6.21	8.21	-4.01	1.02E-11	3.43E-10	ZNF121	zinc finger protein 121	Multiple_Complex
TC0100010314.hg.1	6.61	9.16	-5.87	1.05E-11	3.51E-10	LY9	lymphocyte antigen 9	Multiple_Complex
TC0100016356.hg.1	10.53	8.04	5.63	1.05E-11	3.53E-10	F5	coagulation factor V (proaccelerin, labile facto	Multiple_Complex
TC0100013998.hg.1	7.18	9.02	-3.57	1.07E-11	3.57E-10	HECTD3	HECT domain containing E3 ubiquitin protein l	Multiple_Complex
TC0400012857.hg.1	10.85	8.92	3.8	1.08E-11	3.60E-10	RAPGEF2	Rap guanine nucleotide exchange factor 2	Multiple_Complex
TC0700012293.hg.1	5.77	7.83	-4.17	1.10E-11	3.68E-10	TMEM168	transmembrane protein 168	Multiple_Complex
TC0100014910.hg.1	7.2	10.59	-10.47	1.14E-11	3.79E-10	TGFBR3	transforming growth factor beta receptor III	Multiple_Complex
TC2000008218.hg.1	6.91	8.55	-3.12	1.14E-11	3.80E-10	C20orf194	chromosome 20 open reading frame 194	Multiple_Complex
TC0X00009569.hg.1	7.35	8.84	-2.8	1.15E-11	3.82E-10	ZNF41	zinc finger protein 41	Multiple_Complex
TC0300014047.hg.1	8.4	6.73	3.19	1.16E-11	3.85E-10	ISY1-RAB43	ISY1-RAB43 readthrough	Coding
TC1200012574.hg.1	6.33	7.79	-2.74	1.17E-11	3.87E-10	TULP3	tubby like protein 3	Multiple_Complex
TC0700012596.hg.1	6.75	8.38	-3.09	1.17E-11	3.88E-10	TMEM209	transmembrane protein 209	Multiple_Complex
TC0700008762.hg.1	9.76	12.11	-5.1	1.18E-11	3.91E-10	CBLL1	Cbl proto-oncogene-like 1, E3 ubiquitin protei	Multiple_Complex
TC0400007879.hg.1	10.01	11.55	-2.89	1.25E-11	4.10E-10	USO1	USO1 vesicle transport factor	Multiple_Complex
TC0300013934.hg.1	10.01	7.62	5.25	1.26E-11	4.15E-10	EMC3	ER membrane protein complex subunit 3	Multiple_Complex
TC1100007301.hg.1	5.5	7.82	-4.97	1.27E-11	4.18E-10	PRR5L	proline rich 5 like	Multiple_Complex
TC1200012842.hg.1	7.54	8.72	-2.26	1.28E-11	4.18E-10	ANAPC7	anaphase promoting complex subunit 7	Multiple_Complex
TC2000007928.hg.1	5.73	7.23	-2.84	1.29E-11	4.21E-10	ZNF831	zinc finger protein 831	Coding
TC1200009445.hg.1	9.13	10.65	-2.86	1.29E-11	4.23E-10	SFSWAP	splicing factor, suppressor of white-apricot fa	Multiple_Complex
TC0500009268.hg.1	9.28	7.5	3.43	1.30E-11	4.24E-10	TTC1	tetratricopeptide repeat domain 1	Multiple_Complex
TC1700009651.hg.1	8.03	9.3	-2.42	1.34E-11	4.36E-10	TP53	tumor protein p53	Multiple_Complex
TC1000012130.hg.1	6.57	8.26	-3.22	1.34E-11	4.37E-10	FAM53B	Memczak2013 ALT_ACCEPTOR, ALT_DONOR,	NonCoding
TC0700011929.hg.1	9.99	8.26	3.3	1.36E-11	4.41E-10	SMURF1	SMAD specific E3 ubiquitin protein ligase 1	Multiple_Complex
TC1200011914.hg.1	7.44	9.81	-5.16	1.36E-11	4.41E-10	HVCN1	hydrogen voltage gated channel 1	Multiple_Complex
TC1300007755.hg.1	12.1	10.04	4.2	1.37E-11	4.43E-10	DNAJC3	DnaJ (Hsp40) homolog, subfamily C, member	Multiple_Complex
TC1900008840.hg.1	13.54	11.06	5.56	1.38E-11	4.45E-10	RPS9	ribosomal protein S9	Multiple_Complex
TC1700010914.hg.1	8.22	10.54	-5	1.38E-11	4.46E-10	ARL17B	ADP-ribosylation factor like GTPase 17B	Multiple_Complex
TC2200007904.hg.1	10.29	7.92	5.15	1.39E-11	4.48E-10	ATP6V1E1	ATPase, H+ transporting, lysosomal 31kDa, V1	Multiple_Complex
TC1000009865.hg.1	10.6	8.46	4.43	1.46E-11	4.72E-10	FAM107B	Memczak2013 ALT_ACCEPTOR, ALT_DONOR,	NonCoding
TC0600012542.hg.1	14.96	12.72	4.73	1.47E-11	4.73E-10	UBE2J1	ubiquitin-conjugating enzyme E2, J1	Multiple_Complex
TC1100012687.hg.1	5.23	7.14	-3.75	1.48E-11	4.77E-10	MSANTD2	Myb/SANT-like DNA-binding domain containir	Multiple_Complex
TC1100011833.hg.1	8.17	9.47	-2.46	1.53E-11	4.90E-10	SYTL2	synaptotagmin-like 2	Multiple_Complex
TC1000007404.hg.1	12.8	10.64	4.5	1.54E-11	4.93E-10	CSGALNACT	chondroitin sulfate N-acetylgalactosaminyltra	Multiple_Complex
TC0300006556.hg.1	6.62	7.93	-2.47	1.55E-11	4.94E-10	OGG1	8-oxoguanine DNA glycosylase	Multiple_Complex
TC0400008053.hg.1	13.85	15.78	-3.81	1.55E-11	4.95E-10	AFF1	AF4/FMR2 family, member 1	Multiple_Complex
TC0X00010643.hg.1	11.27	13.26	-3.97	1.61E-11	5.14E-10	6-Sep	septin 6	Multiple_Complex
TC1200009881.hg.1	13.66	9.52	17.56	1.62E-11	5.14E-10	CLEC2B	C-type lectin domain family 2, member B	Multiple_Complex
TC1600011353.hg.1	8.08	10.23	-4.44	1.63E-11	5.18E-10	NPIPA3	nuclear pore complex interacting protein fami	Multiple_Complex
TC0X00007709.hg.1	11.46	9.88	2.99	1.65E-11	5.22E-10	PGK1	phosphoglycerate kinase 1	Multiple_Complex
TSUnmapped00000398.h	7.73	9.41	-3.2	1.68E-11	5.31E-10	KAT6B	K(lysine) acetyltransferase 6B	Coding
TC0500013232.hg.1	8.38	10.58	-4.59	1.68E-11	5.31E-10	SMAD5	SMAD family member 5	Multiple_Complex
TC1200010946.hg.1	6.41	8.19	-3.43	1.69E-11	5.35E-10	NEMP1	nuclear envelope integral membrane protein	Multiple_Complex
TC1700009368.hg.1	11.78	10.03	3.38	1.76E-11	5.54E-10	YWHAE	tyrosine 3-monooxygenase/tryptophan 5-mor	Multiple_Complex
TC0600012713.hg.1	8.25	9.75	-2.84	1.76E-11	5.54E-10	PREP	prolyl endopeptidase	Multiple_Complex
TC1400007947.hg.1	10.82	9.27	2.92	1.77E-11	5.58E-10	PSMC1	proteasome 26S subunit, ATPase 1	Multiple_Complex
TC1600011354.hg.1	8.31	10.19	-3.66	1.78E-11	5.59E-10	NPIPA2	nuclear pore complex interacting protein fami	Coding
TC0800008243.hg.1	8.61	10.08	-2.78	1.80E-11	5.63E-10	PDP1	pyruvate dehyrogenase phosphatase catalytic	Multiple_Complex
TC0X00009069.hg.1	7.69	5.32	5.17	1.80E-11	5.63E-10	ARHGAP6	Rho GTPase activating protein 6	Multiple_Complex
TC1200012758.hg.1	4.47	6.65	-4.52	1.81E-11	5.66E-10	TAS2R14	taste receptor, type 2, member 14	Coding
TC0600006976.hg.1	7.37	5.59	3.46	1.86E-11	5.79E-10	PHACTR1	phosphatase and actin regulator 1	Multiple_Complex
TC1700012438.hg.1	11.83	10.02	3.5	1.88E-11	5.86E-10	SUPT4H1	SPT4 homolog, DSIF elongation factor subunit	Multiple_Complex
TC0100010798.hg.1	10.32	8.77	2.92	1.88E-11	5.86E-10	QSOX1	quiescin Q6 sulfhydryl oxidase 1	Multiple_Complex
TC2000007117.hg.1	10.46	8.75	3.28	1.88E-11	5.86E-10	ASXL1	additional sex combs like transcriptional regul	Multiple_Complex
TC1200010413.hg.1	6.35	8.46	-4.3	1.90E-11	5.89E-10	ABCD2	ATP binding cassette subfamily D member 2	Multiple_Complex
TC2100007402.hg.1	6.66	8.3	-3.12	1.92E-11	5.96E-10	ADARB1	adenosine deaminase, RNA-specific, B1	Multiple_Complex
TC0500007729.hg.1	6.24	7.73	-2.81	1.94E-11	5.99E-10	MCCC2	methylcrotonoyl-CoA carboxylase 2	Multiple_Complex
TC1500010878.hg.1	6.49	8.31	-3.52	1.94E-11	6.00E-10	VWA9	von Willebrand factor A domain containing 9	Multiple_Complex

TC0200015631.hg.1	5.06	7.15	-4.28	1.95E-11	6.01E-10	IKZF2	IKAROS family zinc finger 2	Multiple_Complex
TC0500007314.hg.1	9.12	11.37	-4.74	1.98E-11	6.09E-10	MRPS30	mitochondrial ribosomal protein S30	Multiple_Complex
TC1600007363.hg.1	8.08	6.25	3.54	1.99E-11	6.13E-10	ATXN2L	Transcript Identified by AceView, Entrez Gene	Unassigned
TC2200007982.hg.1	10.94	9.68	2.4	2.00E-11	6.13E-10	UFD1L	ubiquitin fusion degradation 1 like (yeast)	Multiple_Complex
TC1600009777.hg.1	8.39	10.1	-3.25	2.02E-11	6.21E-10	ARHGAP17	Rho GTPase activating protein 17	Multiple_Complex
TC0200008071.hg.1	10.11	8.28	3.56	2.05E-11	6.27E-10	MTHFD2	methylenetetrahydrofolate dehydrogenase (N	Multiple_Complex
TC1800008580.hg.1	10.81	7.3	11.34	2.07E-11	6.34E-10	PSTPIP2	proline-serine-threonine phosphatase interact	Multiple_Complex
TC0900012161.hg.1	9.93	7.9	4.1	2.10E-11	6.42E-10	DNAJC25-G	DNAJC25-GNG10 readthrough	Coding
TC0300011842.hg.1	5.97	7.64	-3.18	2.18E-11	6.64E-10	TOMM70A	translocase of outer mitochondrial membrane	Multiple_Complex
TC0300011899.hg.1	8.64	11.29	-6.27	2.21E-11	6.74E-10	CBLB	Cbl proto-oncogene B, E3 ubiquitin protein lig	Multiple_Complex
TC1500007518.hg.1	13.1	11.28	3.53	2.24E-11	6.81E-10	RAB8B	RAB8B, member RAS oncogene family	Multiple_Complex
TC0300012233.hg.1	6.73	5.14	3.01	2.24E-11	6.82E-10	ITGB5	integrin beta 5	Multiple_Complex
TC0100007326.hg.1	13.63	9.12	22.76	2.29E-11	6.93E-10	RPL11	ribosomal protein L11	Multiple_Complex
TC0900008463.hg.1	10.91	8.17	6.7	2.36E-11	7.14E-10	HSDL2	hydroxysteroid dehydrogenase like 2	Multiple_Complex
TC0100008927.hg.1	12.29	10.35	3.84	2.37E-11	7.15E-10	SH3GLB1	SH3-domain GRB2-like endophilin B1	Multiple_Complex
TC2000009975.hg.1	11.1	12.72	-3.08	2.40E-11	7.25E-10	TMEM230	transmembrane protein 230	Multiple_Complex
TC0300012212.hg.1	8.45	6.13	4.98	2.40E-11	7.25E-10	MYLK	myosin light chain kinase	Multiple_Complex
TC0700013332.hg.1	11.29	12.54	-2.39	2.45E-11	7.39E-10	RBAK; RBAI	RB-associated KRAB zinc finger; RBAK-RBAKDN	Multiple_Complex
TC0800010807.hg.1	14.22	7.93	78.4	2.47E-11	7.42E-10	RPL7	ribosomal protein L7	Multiple_Complex
TC0900012115.hg.1	5.53	7.35	-3.52	2.48E-11	7.45E-10	MTAP	methylthioadenosine phosphorylase	Multiple_Complex
TC1100009611.hg.1	8.02	5.59	5.4	2.48E-11	7.45E-10	JAM3	junctional adhesion molecule 3	Multiple_Complex
TC1900007260.hg.1	14.51	12.54	3.9	2.49E-11	7.46E-10	TPM4	tropomyosin 4	Multiple_Complex
TC0100017860.hg.1	7.27	8.98	-3.27	2.51E-11	7.52E-10	HEATR1	HEAT repeat containing 1	Multiple_Complex
TC1000009095.hg.1	8.6	10.32	-3.29	2.57E-11	7.67E-10	SEC23IP	SEC23 interacting protein	Multiple_Complex
TC0200012307.hg.1	9.72	11.32	-3.03	2.58E-11	7.70E-10	SOS1	SOS Ras/Rac guanine nucleotide exchange fac	Multiple_Complex
TC1100009207.hg.1	7.64	9.44	-3.46	2.60E-11	7.73E-10	KMT2A	lysine (K)-specific methyltransferase 2A	Multiple_Complex
TC0X00008911.hg.1	8.23	9.78	-2.94	2.66E-11	7.89E-10	P2RY8	purinergic receptor P2Y, G-protein coupled, 8	Multiple_Complex
TC0Y00006887.hg.1	8.23	9.78	-2.94	2.66E-11	7.89E-10	P2RY8	Homo sapiens purinergic receptor P2Y, G-prot	Multiple_Complex
TC1000008364.hg.1	13.23	9.62	12.26	2.66E-11	7.89E-10	RP11-380G	Jeck2013 ALT_ACCEPTOR, ALT_DONOR, codin	NonCoding
TC0100009395.hg.1	11.37	8.01	10.21	2.67E-11	7.92E-10	CD53	Memczak2013 ALT_ACCEPTOR, ALT_DONOR,	NonCoding
TC0700008745.hg.1	12.68	8.52	17.81	2.68E-11	7.94E-10	PRKAR2B	protein kinase, cAMP-dependent, regulatory,	Multiple_Complex
TC0600008146.hg.1	10.9	13.17	-4.82	2.73E-11	8.06E-10	RUNX2	runt-related transcription factor 2	Multiple_Complex
TC0200010275.hg.1	8.26	10.19	-3.82	2.74E-11	8.09E-10	GLS	glutaminase	Multiple_Complex
TC0400007938.hg.1	11.76	14.08	-5.01	2.83E-11	8.35E-10	BMP2K	BMP2 inducible kinase	Multiple_Complex
TC0100016715.hg.1	11.21	7.63	11.95	2.90E-11	8.53E-10	PTGS2	prostaglandin-endoperoxide synthase 2 (prost	Multiple_Complex
TC0800010490.hg.1	6.94	8.21	-2.42	2.92E-11	8.57E-10	TMEM68	transmembrane protein 68	Multiple_Complex
TC1200012844.hg.1	12.47	10.03	5.43	2.92E-11	8.57E-10	ARPC3	actin related protein 2/3 complex subunit 3	NonCoding
TC1500010945.hg.1	6.8	9.15	-5.11	2.93E-11	8.59E-10	LINS1	lines homolog 1	Multiple_Complex
TC0400007857.hg.1	6.63	4.71	3.79	2.95E-11	8.62E-10	AREG	amphiregulin	Multiple_Complex
TSUnmapped00000361.h	4.87	6.74	-3.66	2.95E-11	8.62E-10	RASA3	RAS p21 protein activator 3	NonCoding
TC1100007801.hg.1	9.8	11.57	-3.41	2.95E-11	8.62E-10	TMEM138	transmembrane protein 138	Multiple_Complex
TC0300012718.hg.1	7.03	4.68	5.12	2.96E-11	8.63E-10	PLOD2	procollagen-lysine, 2-oxoglutarate 5-dioxygen	Multiple_Complex
TC1700012352.hg.1	12.87	14.75	-3.66	2.98E-11	8.68E-10	TRAPPC1	trafficking protein particle complex 1	Multiple_Complex
TC0500010394.hg.1	6.23	7.97	-3.35	3.03E-11	8.79E-10	DROSHA	drosha, ribonuclease type III	Multiple_Complex
TC2100007032.hg.1	5.22	7.3	-4.23	3.03E-11	8.79E-10	CBR1	carbonyl reductase 1	Multiple_Complex
TC0400011424.hg.1	10.44	8.38	4.18	3.06E-11	8.88E-10	H2AFZ	H2A histone family, member Z	Multiple_Complex
TC1100007948.hg.1	10.4	7.4	8.03	3.08E-11	8.91E-10	PRDX5	peroxiredoxin 5	Coding
TC1000011969.hg.1	8.66	7.06	3.03	3.10E-11	8.95E-10	FAM204A	family with sequence similarity 204, member	Multiple_Complex
TC0700006864.hg.1	6.94	8.48	-2.91	3.12E-11	9.01E-10	SP4	Sp4 transcription factor	Multiple_Complex
TC0100009215.hg.1	10.61	12.29	-3.2	3.14E-11	9.04E-10	CDC14A	cell division cycle 14A	Multiple_Complex
TC1100013012.hg.1	13.33	10.96	5.17	3.15E-11	9.07E-10	STX3	syntaxin 3	Multiple_Complex
TC0100017260.hg.1	7.64	9.33	-3.22	3.15E-11	9.07E-10	NSL1	NSL1, MIS12 kinetochore complex component	Multiple_Complex
TC0200010567.hg.1	10	6.69	9.97	3.21E-11	9.22E-10	EEF1B2; SN	eukaryotic translation elongation factor 1 beta	Multiple_Complex
TC0100010343.hg.1	6.95	5.27	3.22	3.22E-11	9.22E-10	TOMM40L;	translocase of outer mitochondrial membrane	Multiple_Complex
TC0200010788.hg.1	14.71	12.67	4.09	3.23E-11	9.26E-10	ARPC2	actin related protein 2/3 complex subunit 2	Multiple_Complex
TC0300009471.hg.1	9.26	7.24	4.03	3.25E-11	9.30E-10	SKIL	SKI-like proto-oncogene	Multiple_Complex
TC1200010397.hg.1	8.91	10.71	-3.47	3.30E-11	9.42E-10	CPNE8	copine VIII	Multiple_Complex
TC0800007013.hg.1	5.71	7.85	-4.39	3.33E-11	9.49E-10	CHMP7	charged multivesicular body protein 7	Multiple_Complex
TC0100006989.hg.1	7.59	9.11	-2.87	3.36E-11	9.56E-10	DNAJC16	DnaJ (Hsp40) homolog, subfamily C, member	Multiple_Complex
TC0200013515.hg.1	10.24	11.46	-2.33	3.39E-11	9.63E-10	STARD7	StAR-related lipid transfer domain containing	Multiple_Complex
TC0300011485.hg.1	12.18	13.72	-2.9	3.39E-11	9.63E-10	FOXP1	forkhead box P1	Multiple_Complex
TC2100007318.hg.1	5.3	6.78	-2.79	3.53E-11	1.00E-09	RRP1B	ribosomal RNA processing 1B	Multiple_Complex
TC0200010741.hg.1	6.21	7.53	-2.49	3.54E-11	1.00E-09	SMARCAL1	SWI/SNF related, matrix associated, actin dep	Multiple_Complex
TC1300007780.hg.1	6.87	8.51	-3.12	3.61E-11	1.02E-09	RAP2A	RAP2A, member of RAS oncogene family	Coding
TC0900010968.hg.1	7.74	9.91	-4.5	3.62E-11	1.02E-09	TRIM14	tripartite motif containing 14	Multiple_Complex
TC0400009723.hg.1	9.76	11.34	-2.99	3.67E-11	1.03E-09	CTBP1	C-terminal binding protein 1	Multiple_Complex
TC0100014243.hg.1	6.09	8.16	-4.2	3.67E-11	1.03E-09	NDC1	NDC1 transmembrane nucleoporin	Multiple_Complex
TC1100009395.hg.1	8.34	9.82	-2.79	3.73E-11	1.05E-09	TBRG1	transforming growth factor beta regulator 1	Multiple_Complex
TC1900011655.hg.1	7.52	5.75	3.41	3.89E-11	1.09E-09	PET100	PET100 homolog	Multiple_Complex
TC0500012203.hg.1	9.16	7.15	4.03	3.91E-11	1.10E-09	PAIP2; CTB	poly(A) binding protein interacting protein 2 [	NonCoding

TC0500010670.hg.1	14.45	12.36	4.26	4.00E-11	1.12E-09	EMB	embigin	Multiple_Complex
TC0500008698.hg.1	8.15	9.79	-3.13	4.09E-11	1.14E-09	JADE2	jade family PHD finger 2	Multiple_Complex
TC1500008317.hg.1	7.66	9.44	-3.44	4.12E-11	1.15E-09	CRTC3	CREB regulated transcription coactivator 3	Multiple_Complex
TC0200016607.hg.1	10.8	8.86	3.82	4.26E-11	1.19E-09	UBE2F	ubiquitin-conjugating enzyme E2F (putative)	Multiple_Complex
TC0100017079.hg.1	6.6	8.38	-3.42	4.30E-11	1.20E-09	SLC41A1	solute carrier family 41 (magnesium transport	Multiple_Complex
TC0100007760.hg.1	6.19	7.62	-2.7	4.45E-11	1.24E-09	ZMYM1	zinc finger, MYM-type 1	Multiple_Complex
TC0400012213.hg.1	7.16	9.06	-3.73	4.46E-11	1.24E-09	CTSO	cathepsin O	Coding
TC1100009485.hg.1	12.27	13.86	-3.01	4.48E-11	1.25E-09	FLI1	Fli-1 proto-oncogene, ETS transcription factor	Multiple_Complex
TC2200008588.hg.1	6.31	8.33	-4.06	4.52E-11	1.25E-09	APOL3	apolipoprotein L, 3	Multiple_Complex
TC0900012212.hg.1	8.9	6.78	4.34	4.54E-11	1.26E-09	PLIN2	perilipin 2	Multiple_Complex
TC0600013757.hg.1	17.97	14.29	12.8	4.57E-11	1.27E-09	SOD2	superoxide dismutase 2, mitochondrial	Multiple_Complex
TC0500011157.hg.1	4.1	6.42	-4.96	4.59E-11	1.27E-09	GCNT4	glucosaminyl (N-acetyl) transferase 4, core 2	Coding
TC0800011713.hg.1	7.9	9.41	-2.84	4.64E-11	1.28E-09	MTSS1	metastasis suppressor 1	Multiple_Complex
TC0200015194.hg.1	7.8	5.36	5.39	4.67E-11	1.29E-09	TFPI	tissue factor pathway inhibitor (lipoprotein-as	Multiple_Complex
TC0400007839.hg.1	14.85	10.91	15.35	4.73E-11	1.30E-09	PF4V1	platelet factor 4 variant 1	Coding
TC0700008747.hg.1	14.38	12.43	3.86	4.79E-11	1.32E-09	HBP1	HMG-box transcription factor 1	Multiple_Complex
TC1700008180.hg.1	8.59	9.91	-2.5	4.82E-11	1.32E-09	UBE2Z	ubiquitin-conjugating enzyme E2Z	Multiple_Complex
TC0100011400.hg.1	7.33	5.53	3.5	4.83E-11	1.32E-09	PFKFB2	6-phosphofructo-2-kinase/fructose-2,6-bipho:	Multiple_Complex
TC0300010274.hg.1	5.69	7.45	-3.39	4.95E-11	1.36E-09	TAMM41	TAM41 mitochondrial translocator assembly a	Multiple_Complex
TC0700007409.hg.1	8.49	10.57	-4.22	4.97E-11	1.36E-09	CCM2	cerebral cavernous malformation 2	Multiple_Complex
TC0300012435.hg.1	5.5	7.05	-2.94	5.00E-11	1.37E-09	ASTE1	asteroid homolog 1 (Drosophila)	Multiple_Complex
TC0200013298.hg.1	7.55	9.56	-4.03	5.02E-11	1.37E-09	ST3GAL5	ST3 beta-galactoside alpha-2,3-sialyltransfera:	Multiple_Complex
TC0600014097.hg.1	6.5	8.05	-2.92	5.03E-11	1.37E-09	MRPS18B	mitochondrial ribosomal protein S18B	Multiple_Complex
TC0600011517.hg.1	9.28	13.44	-17.84	5.19E-11	1.41E-09	HLA-DPA1	major histocompatibility complex, class II, DP	Multiple_Complex
TC0900011626.hg.1	8.53	10.38	-3.6	5.22E-11	1.42E-09	CIZ1	CDKN1A interacting zinc finger protein 1	Multiple_Complex
TC1700008194.hg.1	4.33	5.69	-2.56	5.25E-11	1.43E-09	ABI3	ABI family, member 3	Multiple_Complex
TC0600012777.hg.1	11.97	10.08	3.72	5.46E-11	1.48E-09	SNX3	sorting nexin 3	Multiple_Complex
TC0500009131.hg.1	8.74	9.99	-2.37	5.52E-11	1.49E-09	G3BP1	GTPase activating protein (SH3 domain) bindi	Multiple_Complex
TC1100009666.hg.1	8.07	9.31	-2.36	5.53E-11	1.49E-09	SIGIRR	single immunoglobulin and toll-interleukin 1 r	Multiple_Complex
TC0200015739.hg.1	6.47	8.17	-3.23	5.55E-11	1.50E-09	ZNF142	zinc finger protein 142	Multiple_Complex
TC1500010041.hg.1	6.8	8.32	-2.87	5.55E-11	1.50E-09	EDC3	enhancer of mRNA decapping 3	Multiple_Complex
TC1700011033.hg.1	8.83	13.09	-19.22	5.60E-11	1.51E-09	PHOSPHO1	phosphatase, orphan 1	Coding
TC1400010759.hg.1	7.81	5.63	4.55	5.61E-11	1.51E-09	ATP6V1D	ATPase, H+ transporting, lysosomal 34kDa, V1	NonCoding
TC1300006699.hg.1	9.78	7.89	3.71	5.72E-11	1.54E-09	ATP5EP2	ATP synthase, H+ transporting, mitochondrial	Multiple_Complex
TC1900008329.hg.1	14.75	12.26	5.59	5.77E-11	1.55E-09	VASP	vasodilator-stimulated phosphoprotein	Multiple_Complex
TC0800008546.hg.1	9.24	6.9	5.04	5.79E-11	1.55E-09	ENY2	enhancer of yellow 2 homolog (Drosophila)	Multiple_Complex
TC1700010459.hg.1	7.92	5.79	4.38	5.80E-11	1.55E-09	CCL3	chemokine (C-C motif) ligand 3	Multiple_Complex
TC1200006788.hg.1	9.43	13.42	-15.88	5.85E-11	1.56E-09	KLRD1	killer cell lectin-like receptor subfamily D, mer	Multiple_Complex
TC0700007831.hg.1	7.15	4.73	5.35	6.03E-11	1.61E-09	TPST1	tyrosylprotein sulfotransferase 1	Multiple_Complex
TC0200016319.hg.1	7.34	8.62	-2.42	6.05E-11	1.61E-09	ANKMY1	ankyrin repeat and MYND domain containing	Multiple_Complex
TC0X00011301.hg.1	6.29	4.7	3.01	6.07E-11	1.62E-09	MSN	moesin	NonCoding
TC1700012387.hg.1	7.33	9.84	-5.68	6.10E-11	1.62E-09	SLFN12L	schlafen family member 12-like	Multiple_Complex
TC0300007256.hg.1	10.04	12.24	-4.6	6.14E-11	1.63E-09	CCR2	chemokine (C-C motif) receptor 2	Multiple_Complex
TC0900011393.hg.1	6.71	8.78	-4.21	6.19E-11	1.64E-09	TRAF1	TNF receptor-associated factor 1	Multiple_Complex
TC0300009126.hg.1	11.05	9.26	3.47	6.42E-11	1.70E-09	GYG1	glycogenin 1	Multiple_Complex
TC2000006957.hg.1	7.67	6.22	2.72	6.48E-11	1.72E-09	GZF1	GDNF-inducible zinc finger protein 1	Multiple_Complex
TC0300009179.hg.1	5.97	3.95	4.07	6.57E-11	1.74E-09	MED12L	mediator complex subunit 12 like	Multiple_Complex
TC1900006520.hg.1	6.24	9.1	-7.26	6.74E-11	1.78E-09	ATP5D	ATP synthase, H+ transporting, mitochondrial	Multiple_Complex
TC0100008132.hg.1	6.65	8.49	-3.56	6.74E-11	1.78E-09	TMEM69	transmembrane protein 69	Multiple_Complex
TC0900011021.hg.1	5.78	7.33	-2.91	6.91E-11	1.82E-09	TEX10	testis expressed 10	Multiple_Complex
TC0400010615.hg.1	7.52	9.8	-4.86	7.04E-11	1.85E-09	TXK	TXK tyrosine kinase	Multiple_Complex
TC0100014226.hg.1	10.05	11.57	-2.86	7.26E-11	1.91E-09	C1orf123	chromosome 1 open reading frame 123	Multiple_Complex
TC1600007262.hg.1	8.99	10.31	-2.5	7.40E-11	1.94E-09	TNRC6A	trinucleotide repeat containing 6A	Multiple_Complex
TC0100008641.hg.1	11.42	9.63	3.47	7.42E-11	1.95E-09	MIER1	mesoderm induction early response 1, transcr	Multiple_Complex
TC0500012519.hg.1	13.23	8.78	21.86	7.45E-11	1.95E-09	SPARC	secreted protein, acidic, cysteine-rich (osteon	Multiple_Complex
TC1700012269.hg.1	6.33	8.21	-3.68	7.59E-11	1.99E-09	MGC57346	Homo sapiens MGC57346-CRHR1 readthrough	Multiple_Complex
TC0100015017.hg.1	9.65	6.12	11.57	7.75E-11	2.02E-09	RPL7	ribosomal protein L7	Multiple_Complex
TC0400011012.hg.1	14.57	10.05	22.94	7.78E-11	2.03E-09	PF4	platelet factor 4	Coding
TC0100008383.hg.1	7.96	9.38	-2.67	7.79E-11	2.03E-09	MRPL37	mitochondrial ribosomal protein L37	Multiple_Complex
TC1700009982.hg.1	12.45	11.21	2.37	7.87E-11	2.05E-09	USP32P2; F	ubiquitin specific peptidase 32 pseudogene 2;	Multiple_Complex
TC0600008622.hg.1	10.54	6.95	12.03	7.95E-11	2.07E-09	SH3BGRL2	SH3 domain binding glutamate-rich protein lik	Multiple_Complex
TC1400008908.hg.1	10.83	8.72	4.32	8.09E-11	2.10E-09	EAPP	E2F-associated phosphoprotein	Multiple_Complex
TC0500008814.hg.1	13.86	12.14	3.29	8.14E-11	2.11E-09	PAIP2	poly(A) binding protein interacting protein 2	Multiple_Complex
TC1100010222.hg.1	6.28	4.04	4.71	8.17E-11	2.12E-09	RPL36A	ribosomal protein L36a	Multiple_Complex
TC0300010722.hg.1	10.15	8.24	3.75	8.20E-11	2.12E-09	LRRFIP2	leucine rich repeat (in FLII) interacting protein	Multiple_Complex
TC0900012225.hg.1	12.26	13.87	-3.04	8.28E-11	2.14E-09	RNF38	ring finger protein 38	Multiple_Complex
TC0300014046.hg.1	13.47	11.13	5.05	8.43E-11	2.18E-09	CNBP	CCHC-type zinc finger, nucleic acid binding pro	Multiple_Complex
TC0100016145.hg.1	7.22	8.79	-2.98	8.61E-11	2.22E-09	CD244	CD244 molecule, natural killer cell receptor 2E	Multiple_Complex
TC1700012271.hg.1	5.06	6.82	-3.38	8.69E-11	2.24E-09	GOSR2	golgi SNAP receptor complex member 2	Multiple_Complex
TC1400009732.hg.1	7.82	9.85	-4.08	8.97E-11	2.31E-09	C14orf1	chromosome 14 open reading frame 1	Multiple_Complex

TC1500008984.hg.1	14.69	12.23	5.52	8.98E-11	2.31E-09	NOP10	NOP10 ribonucleoprotein	Multiple_Complex
TC1000011501.hg.1	6.16	7.61	-2.73	9.00E-11	2.31E-09	TCTN3	tectonic family member 3	Multiple_Complex
TC1400007172.hg.1	9.73	11.3	-2.98	9.06E-11	2.32E-09	STYX	serine/threonine/tyrosine interacting protein	Multiple_Complex
TC1700012356.hg.1	13.58	7.51	67.15	9.08E-11	2.32E-09	RPL26	ribosomal protein L26	Multiple_Complex
TC0400008620.hg.1	5.73	7.46	-3.33	9.09E-11	2.32E-09	SPATA5	spermatogenesis associated 5	Multiple_Complex
TC2200009274.hg.1	7.9	10.12	-4.66	9.28E-11	2.37E-09	APOBEC3G	apolipoprotein B mRNA editing enzyme, cataly	Multiple_Complex
TC1700009713.hg.1	8.6	6.94	3.15	9.50E-11	2.42E-09	STX8	syntaxin 8	Multiple_Complex
TC1200007906.hg.1	6.72	4.72	3.99	9.67E-11	2.46E-09	XRCC6BP1	XRCC6 binding protein 1	Multiple_Complex
TC0400010580.hg.1	4.65	6.24	-3	9.75E-11	2.48E-09	GNPDA2	glucosamine-6-phosphate deaminase 2	Multiple_Complex
TC1100013230.hg.1	7.61	9.47	-3.62	9.83E-11	2.50E-09	BCL9L	B-cell CLL/lymphoma 9-like	Multiple_Complex
TC1000008159.hg.1	15.44	9.67	54.43	9.94E-11	2.52E-09	RPS24	ribosomal protein S24	Multiple_Complex
TC0X00009869.hg.1	6.49	8.11	-3.06	1.00E-10	2.54E-09	ARHGEF9; /	Cdc42 guanine nucleotide exchange factor 9; /	Multiple_Complex
TC0700009211.hg.1	9.34	10.63	-2.45	1.02E-10	2.59E-09	EXOC4	exocyst complex component 4	Multiple_Complex
TC0X00007716.hg.1	7.91	10.28	-5.17	1.03E-10	2.61E-09	P2RY10	purinergic receptor P2Y, G-protein coupled, 1(	Multiple_Complex
TC0100006805.hg.1	7.04	5.15	3.72	1.03E-10	2.61E-09	RBP7	retinol binding protein 7, cellular	Multiple_Complex
TC2000007512.hg.1	7.55	9.64	-4.27	1.04E-10	2.62E-09	PCIF1	PDX1 C-terminal inhibiting factor 1	Multiple_Complex
TC0800008531.hg.1	9.84	7.39	5.47	1.07E-10	2.69E-09	EMC2	ER membrane protein complex subunit 2	Multiple_Complex
TC0600013272.hg.1	11.75	9.62	4.38	1.08E-10	2.71E-09	BCLAF1	BCL2-associated transcription factor 1	Multiple_Complex
TC0300007324.hg.1	7.62	5.35	4.81	1.12E-10	2.80E-09	CAMP	cathelicidin antimicrobial peptide	Coding
TC0100014065.hg.1	9.33	10.98	-3.13	1.12E-10	2.82E-09	EFCAB14	EF-hand calcium binding domain 14	Multiple_Complex
TC0300007842.hg.1	8.22	6.77	2.73	1.16E-10	2.90E-09	EBLN2	endogenous Bornavirus-like nucleoprotein 2	Coding
TC0200016752.hg.1	5.17	7.14	-3.92	1.17E-10	2.93E-09	TTN	titin	Multiple_Complex
TC0200008666.hg.1	8.15	5.61	5.82	1.19E-10	2.97E-09	IL1R1	interleukin 1 receptor, type I	Multiple_Complex
TC0100006812.hg.1	9.23	6.8	5.37	1.19E-10	2.98E-09	KIF1B	kinesin family member 1B	Multiple_Complex
TC1900008989.hg.1	8.33	9.72	-2.61	1.20E-10	2.98E-09	ZNF460	zinc finger protein 460	Multiple_Complex
TC0200012095.hg.1	8.13	10.28	-4.44	1.20E-10	3.00E-09	SUPT7L	SPT7-like STAGA complex gamma subunit	Multiple_Complex
TC0500013385.hg.1	6.6	4.71	3.71	1.23E-10	3.07E-09	SH3TC2	SH3 domain and tetratricopeptide repeats 2	Multiple_Complex
TC1100009068.hg.1	5.24	7.67	-5.4	1.25E-10	3.10E-09	NCAM1	neural cell adhesion molecule 1	Multiple_Complex
TC1200009829.hg.1	15.05	10.82	18.7	1.25E-10	3.10E-09	CLEC4E	C-type lectin domain family 4, member E	Multiple_Complex
TC1700010551.hg.1	13.19	7.27	60.36	1.25E-10	3.10E-09	RPL23; SNC	ribosomal protein L23; small nucleolar RNA, H	Multiple_Complex
TC1400007562.hg.1	17.06	14.59	5.51	1.25E-10	3.10E-09	SRSF5	serine/arginine-rich splicing factor 5	Multiple_Complex
TC0300012216.hg.1	7.93	9.43	-2.82	1.27E-10	3.14E-09	CCDC14	coiled-coil domain containing 14	Multiple_Complex
TC0100008375.hg.1	5.25	6.71	-2.75	1.27E-10	3.14E-09	TCEANC2; I	transcription elongation factor A (SII) N-termi	Multiple_Complex
TC0900008660.hg.1	10.4	6.96	10.83	1.29E-10	3.19E-09	PTGS1	prostaglandin-endoperoxide synthase 1 (prost	Coding
TC0300007083.hg.1	9.69	7.39	4.94	1.30E-10	3.20E-09	WDR48	WD repeat domain 48	Multiple_Complex
TC1100011157.hg.1	11.74	10.01	3.31	1.30E-10	3.21E-09	PPP1R14B	protein phosphatase 1, regulatory (inhibitor) s	Multiple_Complex
TC1500009446.hg.1	6.12	7.36	-2.36	1.33E-10	3.27E-09	LEO1	LEO1 homolog, Paf1/RNA polymerase II comp	Multiple_Complex
TC0600008531.hg.1	6.58	7.94	-2.57	1.33E-10	3.28E-09	MTO1	mitochondrial tRNA translation optimization 1	Multiple_Complex
TC1000007866.hg.1	8.23	9.74	-2.85	1.35E-10	3.31E-09	CCAR1; SN(	cell division cycle and apoptosis regulator 1; s	Multiple_Complex
TC1000008046.hg.1	6.46	7.73	-2.42	1.41E-10	3.45E-09	FUT11	fucosyltransferase 11 (alpha (1,3) fucosyltrans	Multiple_Complex
TC0100007676.hg.1	8.13	11.41	-9.68	1.41E-10	3.45E-09	LCK	LCK proto-oncogene, Src family tyrosine kinas	Multiple_Complex
TC1700008095.hg.1	10.75	12.91	-4.48	1.42E-10	3.47E-09	LRRC37A	leucine rich repeat containing 37A	Multiple_Complex
TC1600011556.hg.1	6.08	7.98	-3.73	1.46E-10	3.58E-09	PDF; COG8	peptide deformylase (mitochondrial); compor	Multiple_Complex
TC0600009279.hg.1	6.49	8.16	-3.18	1.47E-10	3.60E-09	ASF1A	anti-silencing function 1A histone chaperone	Multiple_Complex
TC0400011695.hg.1	9.35	10.62	-2.42	1.48E-10	3.61E-09	SEC24D	SEC24 homolog D, COPII coat complex compo	Multiple_Complex
TC0700013460.hg.1	7.33	8.89	-2.93	1.53E-10	3.73E-09	METTL2B	methyltransferase like 2B	Multiple_Complex
TC1300009597.hg.1	7.33	10.02	-6.46	1.56E-10	3.79E-09	GPR18	G protein-coupled receptor 18	Coding
TC1200010944.hg.1	4.95	6.3	-2.55	1.58E-10	3.85E-09	ZBTB39	zinc finger and BTB domain containing 39	Coding
TC0X00009441.hg.1	9.41	10.95	-2.92	1.59E-10	3.87E-09	MED14	mediator complex subunit 14	Multiple_Complex
TC0200009911.hg.1	9.91	8.17	3.34	1.60E-10	3.87E-09	PPIG	peptidylprolyl isomerase G (cyclophilin G)	Multiple_Complex
TC0200013112.hg.1	10.35	11.69	-2.53	1.61E-10	3.90E-09	AUP1	ancient ubiquitous protein 1	Multiple_Complex
TC1100010089.hg.1	12.95	10.65	4.92	1.64E-10	3.96E-09	RNF141	ring finger protein 141	Multiple_Complex
TC1700008531.hg.1	6.9	8.23	-2.52	1.64E-10	3.96E-09	METTL2A	methyltransferase like 2A	Multiple_Complex
TC1900011915.hg.1	7.7	9.13	-2.69	1.64E-10	3.96E-09	ZNF737	zinc finger protein 737	Multiple_Complex
TC1500007554.hg.1	7.25	9.02	-3.4	1.69E-10	4.06E-09	ZNF609	zinc finger protein 609	Multiple_Complex
TC1800008331.hg.1	9.5	12.2	-6.5	1.69E-10	4.06E-09	SS18	synovial sarcoma translocation, chromosome	Multiple_Complex
TC1700008563.hg.1	7.49	9.36	-3.65	1.69E-10	4.07E-09	DCAF7	DDB1 and CUL4 associated factor 7	Multiple_Complex
TC1600007887.hg.1	11.72	13.13	-2.65	1.70E-10	4.08E-09	RBL2	retinoblastoma-like 2	Multiple_Complex
TC1200012763.hg.1	6.7	8.38	-3.21	1.71E-10	4.10E-09	TAS2R30; T	taste receptor, type 2, member 30; taste rece	Multiple_Complex
TC0700007067.hg.1	7.03	5.14	3.7	1.72E-10	4.13E-09	MTURN	maturin, neural progenitor differentiation reg	Multiple_Complex
TC1700011903.hg.1	6.67	4.75	3.78	1.74E-10	4.17E-09	SOCS3	suppressor of cytokine signaling 3	Coding
TSUnmapped00000256.h	5.59	7.1	-2.84	1.76E-10	4.20E-09	DYRK1B	dual specificity tyrosine-(Y)-phosphorylation r	Coding
TC1700007602.hg.1	8.56	10.39	-3.53	1.79E-10	4.27E-09	TAF15	TATA box binding protein associated factor 15	Multiple_Complex
TC0800010667.hg.1	9.59	11.36	-3.4	1.82E-10	4.34E-09	PDE7A	phosphodiesterase 7A	Multiple_Complex
TC0600007108.hg.1	11.37	8.81	5.92	1.82E-10	4.34E-09	RNF144B	ring finger protein 144B	Multiple_Complex
TC1900011937.hg.1	4.98	6.17	-2.28	1.85E-10	4.41E-09	ZNF607	zinc finger protein 607	Multiple_Complex
TC0500008845.hg.1	9.68	11.26	-2.99	1.85E-10	4.41E-09	PURA	purine-rich element binding protein A	Multiple_Complex
TC0300013962.hg.1	9.75	7.75	4.01	1.87E-10	4.45E-09	ANO10	anoctamin 10	Multiple_Complex
TC0600007402.hg.1	7.03	8.81	-3.43	1.91E-10	4.54E-09	ZKSCAN8	zinc finger with KRAB and SCAN domains 8	Multiple_Complex
TC1000008054.hg.1	8.86	5.97	7.44	1.96E-10	4.66E-09	PLAU	plasminogen activator, urokinase	Multiple_Complex

TC0200008632.hg.1	12.54	8.91	12.41	1.99E-10	4.71E-09	RPL31	ribosomal protein L31	Multiple_Complex
TC0100013432.hg.1	10.39	12.43	-4.13	1.99E-10	4.72E-09	WASF2	WAS protein family, member 2	Multiple_Complex
TC1800009223.hg.1	10.27	8.75	2.86	2.00E-10	4.73E-09	RNF138	ring finger protein 138, E3 ubiquitin protein lig	Multiple_Complex
TC1600009524.hg.1	8.74	10.29	-2.92	2.01E-10	4.75E-09	NPIPA5	nuclear pore complex interacting protein fami	Coding
TC0500010493.hg.1	5.95	7.7	-3.36	2.03E-10	4.79E-09	LMBRD2	LMBR1 domain containing 2	Multiple_Complex
TC1200012717.hg.1	9.29	10.44	-2.21	2.03E-10	4.79E-09	GATC	glutamyl-tRNA(Gln) amidotransferase, subuni	Multiple_Complex
TC0400012757.hg.1	10.19	11.97	-3.43	2.05E-10	4.83E-09	RNF4	ring finger protein 4	Multiple_Complex
TC0800007978.hg.1	9.69	7.37	4.99	2.11E-10	4.95E-09	LY96	lymphocyte antigen 96	Coding
TC2000009874.hg.1	11.63	14.72	-8.5	2.12E-10	4.98E-09	PSMF1	proteasome inhibitor subunit 1	Multiple_Complex
TC1000010737.hg.1	6.72	4.31	5.3	2.13E-10	4.99E-09	RHOBTB1	Rho-related BTB domain containing 1	Multiple_Complex
TC1700006719.hg.1	17.01	18.85	-3.57	2.13E-10	5.00E-09	RNASEK; C1	ribonuclease, RNase K; chromosome 17 open	Multiple_Complex
TC2000006518.hg.1	8.5	9.91	-2.65	2.15E-10	5.04E-09	NOP56; SN	NOP56 ribonucleoprotein; small nucleolar RN/	Multiple_Complex
TC0900006970.hg.1	8.59	10.08	-2.8	2.16E-10	5.06E-09	NFX1	nuclear transcription factor, X-box binding 1	Multiple_Complex
TC2200008489.hg.1	8.23	9.93	-3.24	2.21E-10	5.16E-09	PRR14L	proline rich 14-like	Multiple_Complex
TC1200011400.hg.1	10.82	8.5	5.01	2.22E-10	5.18E-09	CCDC59	coiled-coil domain containing 59	Multiple_Complex
TSUnmapped00000222.h	5.61	7.55	-3.86	2.25E-10	5.25E-09	ZNF780A	zinc finger protein 780A	Coding
TC0300011081.hg.1	8.1	9.58	-2.78	2.25E-10	5.25E-09	UBA7; MIR:	ubiquitin-like modifier activating enzyme 7; m	Multiple_Complex
TC0300007100.hg.1	7.84	9.91	-4.2	2.26E-10	5.26E-09	SLC25A38	solute carrier family 25, member 38	Multiple_Complex
TC0100016625.hg.1	16.95	14.91	4.12	2.26E-10	5.26E-09	GLUL	glutamate-ammonia ligase	Multiple_Complex
TC1200012267.hg.1	10.22	8.65	2.98	2.32E-10	5.38E-09	SBNO1; MI	strawberry notch homolog 1 (Drosophila); mic	Multiple_Complex
TC0800008916.hg.1	9.72	10.88	-2.24	2.34E-10	5.42E-09	EFR3A	EFR3 homolog A	Multiple_Complex
TC0600014074.hg.1	10.5	8.95	2.94	2.34E-10	5.42E-09	HIVEP1	human immunodeficiency virus type I enhanc	Multiple_Complex
TC1500007202.hg.1	10.34	9.05	2.45	2.39E-10	5.54E-09	USP8	ubiquitin specific peptidase 8	Multiple_Complex
TC1200009796.hg.1	19.16	16.62	5.83	2.40E-10	5.54E-09	SLC2A14	solute carrier family 2 (facilitated glucose tran	Multiple_Complex
TC1700012283.hg.1	10.26	12.15	-3.72	2.43E-10	5.60E-09	LUC7L3	LUC7-like 3 pre-mRNA splicing factor	Multiple_Complex
TC0500012163.hg.1	3.9	5.18	-2.42	2.43E-10	5.62E-09	CDC23	cell division cycle 23	Multiple_Complex
TC1900007181.hg.1	8.39	10.45	-4.18	2.45E-10	5.63E-09	TECR; MIR6	trans-2,3-enoyl-CoA reductase; microRNA 639	Multiple_Complex
TC0300013795.hg.1	6.96	8.44	-2.79	2.47E-10	5.69E-09	OXNAD1	oxidoreductase NAD-binding domain containi	Multiple_Complex
TC0100010335.hg.1	7.03	8.96	-3.81	2.54E-10	5.83E-09	USP21	ubiquitin specific peptidase 21	Multiple_Complex
TC0400009088.hg.1	9.8	6.66	8.83	2.54E-10	5.83E-09	GUCY1B3	guanylate cyclase 1, soluble, beta 3	Multiple_Complex
TC0500013342.hg.1	9.61	11.83	-4.66	2.54E-10	5.83E-09	ERAP1	endoplasmic reticulum aminopeptidase 1	Multiple_Complex
TC0100010341.hg.1	16.23	13.86	5.17	2.56E-10	5.86E-09	FCER1G	Fc fragment of IgE, high affinity I, receptor for	Multiple_Complex
TC0X00009255.hg.1	10.75	7.04	13.02	2.56E-10	5.87E-09	RPL9P7	ribosomal protein L9 pseudogene 7	Multiple_Complex
TC1600007992.hg.1	8.21	9.47	-2.39	2.58E-10	5.90E-09	ARL2BP	ADP-ribosylation factor like GTPase 2 binding	Multiple_Complex
TC0800011625.hg.1	8	9.22	-2.33	2.59E-10	5.91E-09	TAF2	TAF2 RNA polymerase II, TATA box binding pro	Multiple_Complex
TC0400007853.hg.1	5.27	3.8	2.76	2.59E-10	5.91E-09	EREG	epiregulin	Multiple_Complex
TC0100013651.hg.1	11.05	7.95	8.56	2.60E-10	5.92E-09	PHC2; MIR:	polyhomeotic homolog 2 (Drosophila); microR	Multiple_Complex
TC1900011934.hg.1	8.55	10.13	-2.99	2.69E-10	6.13E-09	ZNF585B	zinc finger protein 585B	Multiple_Complex
TC0X00011308.hg.1	10.26	5.62	24.83	2.71E-10	6.17E-09	RPL36A	ribosomal protein L36a	Multiple_Complex
TC1100010274.hg.1	8.34	6.96	2.59	2.73E-10	6.20E-09	SPTY2D1	SPT2 chromatin protein domain containing 1	Multiple_Complex
TC0100007458.hg.1	6.92	8.36	-2.71	2.73E-10	6.20E-09	DHDDS	dehydrodolichyl diphosphate synthase subuni	Multiple_Complex
TC1100010992.hg.1	9.09	5.65	10.81	2.76E-10	6.26E-09	TCN1	transcobalamin I (vitamin B12 binding protein	Multiple_Complex
TC1900009633.hg.1	6.22	8.46	-4.74	2.78E-10	6.29E-09	S1PR5	sphingosine-1-phosphate receptor 5	Coding
TC1000010292.hg.1	5.75	4.47	2.44	2.80E-10	6.33E-09	PARD3	par-3 family cell polarity regulator	Multiple_Complex
TC0200016585.hg.1	9.57	7.63	3.83	2.81E-10	6.34E-09	MOB4	MOB family member 4, phocein	Multiple_Complex
TC0800007367.hg.1	7.76	5.84	3.79	2.84E-10	6.41E-09	ADAM9	ADAM metallopeptidase domain 9	Multiple_Complex
TC1300009293.hg.1	9.16	10.83	-3.2	2.84E-10	6.41E-09	MYCBP2	MYC binding protein 2, E3 ubiquitin protein lig	Multiple_Complex
TC1100008673.hg.1	4.47	6.12	-3.13	2.85E-10	6.42E-09	TMEM135	transmembrane protein 135	Multiple_Complex
TSUnmapped00000311.h	5.78	7.55	-3.4	2.85E-10	6.42E-09	ZNF780A	zinc finger protein 780A	Coding
TC1900010673.hg.1	5.71	6.99	-2.42	2.90E-10	6.51E-09	DYRK1B	dual specificity tyrosine-(Y)-phosphorylation r	Multiple_Complex
TC1700010732.hg.1	8.15	9.63	-2.79	2.94E-10	6.60E-09	FAM134C	family with sequence similarity 134, member	Multiple_Complex
TC0500011260.hg.1	7.97	9.26	-2.44	2.94E-10	6.60E-09	SERINC5	serine incorporator 5	Multiple_Complex
TC1000008193.hg.1	9.65	7.49	4.47	2.96E-10	6.64E-09	PPIF	peptidylprolyl isomerase F	Multiple_Complex
TC1800009215.hg.1	12.25	10.3	3.86	3.09E-10	6.93E-09	ANKRD12	ankyrin repeat domain 12	Multiple_Complex
TC0100006698.hg.1	7.45	6.12	2.52	3.13E-10	6.99E-09	PHF13	PHD finger protein 13	Multiple_Complex
TC0700007285.hg.1	10.59	12.01	-2.68	3.16E-10	7.05E-09	CDK13	cyclin-dependent kinase 13	Multiple_Complex
TC0800007439.hg.1	8.33	6.74	3.01	3.31E-10	7.39E-09	POLB	polymerase (DNA directed), beta	Multiple_Complex
TC0200014160.hg.1	7.13	9.37	-4.73	3.34E-10	7.45E-09	BIN1	bridging integrator 1	Multiple_Complex
TC0200006757.hg.1	5.82	8.02	-4.58	3.36E-10	7.48E-09	TRIB2	tribbles pseudokinase 2	Multiple_Complex
TC0400012941.hg.1	8.05	6.43	3.08	3.41E-10	7.58E-09	THAP9-AS1	THAP9 antisense RNA 1	NonCoding
TC0600013160.hg.1	8.17	11.13	-7.76	3.41E-10	7.58E-09	SAMD3	sterile alpha motif domain containing 3	Multiple_Complex
TC1500010755.hg.1	6.29	4.68	3.05	3.42E-10	7.60E-09	ANKDD1A	ankyrin repeat and death domain containing 1	Multiple_Complex
TC0500008558.hg.1	9.07	10.85	-3.43	3.47E-10	7.71E-09	PRRC1	proline-rich coiled-coil 1	Multiple_Complex
TC1500010869.hg.1	10.8	8.44	5.13	3.63E-10	8.04E-09	CCPG1; MII	cell cycle progression 1; microRNA 628	Multiple_Complex
TC1600010792.hg.1	4.73	6.66	-3.79	3.71E-10	8.22E-09	PHLPP2	PH domain and leucine rich repeat protein ph	Multiple_Complex
TC0100006729.hg.1	10.68	8.4	4.85	3.78E-10	8.36E-09	PARK7	parkinson protein 7	Multiple_Complex
TC0700013538.hg.1	9.86	13.25	-10.49	3.86E-10	8.52E-09	TRGJ1; TRG	T cell receptor gamma joining 1; T cell recepto	Multiple_Complex
TC1100010346.hg.1	7.34	8.61	-2.41	3.87E-10	8.54E-09	FANCF	Fanconi anemia complementation group F	Coding
TC0500011408.hg.1	7.45	8.88	-2.71	3.88E-10	8.56E-09	TMEM161E	transmembrane protein 161B	Multiple_Complex
TC0500010169.hg.1	6.82	8.91	-4.24	3.89E-10	8.56E-09	ANKH	ANKH inorganic pyrophosphate transport regu	Multiple_Complex

TC0700008517.hg.1	7.99	9.31	-2.5	3.90E-10	8.57E-09	ZKSCAN1	zinc finger with KRAB and SCAN domains 1	Multiple_Complex
TC1500010080.hg.1	5.78	7.73	-3.88	3.92E-10	8.60E-09	IMP3	IMP3, U3 small nucleolar ribonucleoprotein	Multiple_Complex
TC2000009053.hg.1	6.75	9.09	-5.05	3.93E-10	8.63E-09	TTI1	TELO2 interacting protein 1	Multiple_Complex
TC0300007312.hg.1	6.62	8.28	-3.17	3.94E-10	8.65E-09	DHX30	DEAH (Asp-Glu-Ala-His) box helicase 30	Multiple_Complex
TC0200012713.hg.1	7.18	9.48	-4.91	3.95E-10	8.66E-09	BCL11A	B-cell CLL/lymphoma 11A (zinc finger protein)	Multiple_Complex
TC1900006578.hg.1	9.7	11.26	-2.94	3.97E-10	8.69E-09	SF3A2	splicing factor 3a subunit 2	Multiple_Complex
TC0300011273.hg.1	12.19	10.76	2.69	4.05E-10	8.85E-09	ARF4	ADP-ribosylation factor 4	Multiple_Complex
TC1800008219.hg.1	10.75	8.39	5.12	4.10E-10	8.96E-09	ROCK1	Rho-associated, coiled-coil containing protein	Multiple_Complex
TC0700012734.hg.1	5.46	7.59	-4.38	4.31E-10	9.41E-09	CREB3L2	cAMP responsive element binding protein 3-li	Multiple_Complex
TC1100007287.hg.1	8.51	9.56	-2.07	4.35E-10	9.49E-09	TRIM44	tripartite motif containing 44	Multiple_Complex
TC0200014995.hg.1	16.04	17.51	-2.77	4.37E-10	9.52E-09	WIPF1	WAS/WASL interacting protein family, membe	Multiple_Complex
TC0300013920.hg.1	4.96	5.99	-2.04	4.41E-10	9.59E-09	NRROS	negative regulator of reactive oxygen species	Multiple_Complex
TC0500013109.hg.1	7.42	8.67	-2.36	4.42E-10	9.61E-09	ZFP62	ZFP62 zinc finger protein	Multiple_Complex
TC0200012405.hg.1	6.72	8.82	-4.29	4.45E-10	9.66E-09	THADA	thyroid adenoma associated	Multiple_Complex
TC0400011815.hg.1	7.38	9.31	-3.81	4.49E-10	9.75E-09	MFSD8	major facilitator superfamily domain containir	Multiple_Complex
TC1000007337.hg.1	8.07	9.41	-2.54	4.51E-10	9.78E-09	ZNF37A	zinc finger protein 37A	Multiple_Complex
TC1700009640.hg.1	5.63	7.07	-2.73	4.54E-10	9.81E-09	ZBTB4	zinc finger and BTB domain containing 4	Multiple_Complex
TC1600008126.hg.1	9.18	6.81	5.16	4.54E-10	9.81E-09	CMTM2	CKLF-like MARVEL transmembrane domain co	Multiple_Complex
TC0900010995.hg.1	6.39	8.06	-3.17	4.60E-10	9.92E-09	ALG2	ALG2, alpha-1,3/1,6-mannosyltransferase	Multiple_Complex
TC1500007986.hg.1	12.21	10.6	3.05	4.60E-10	9.92E-09	MORF4L1	mortality factor 4 like 1	Multiple_Complex
TC0100012687.hg.1	7.09	8.77	-3.2	4.64E-10	1.00E-08	NOL9	nucleolar protein 9	Multiple_Complex
TC0100011458.hg.1	9.93	11.6	-3.17	4.65E-10	1.00E-08	TRAF3IP3	TRAF3 interacting protein 3	Multiple_Complex
TC0400012902.hg.1	6.53	10.34	-14.09	4.66E-10	1.00E-08	FGFBP2	fibroblast growth factor binding protein 2	Coding
TC0X00009124.hg.1	9.04	7.27	3.41	4.67E-10	1.00E-08	PIGA	phosphatidylinositol glycan anchor biosynthes	Multiple_Complex
TC0200016648.hg.1	13.21	10.98	4.69	4.79E-10	1.03E-08	CDC42EP3	CDC42 effector protein (Rho GTPase binding)	Coding
TC1000008221.hg.1	5.37	7.08	-3.27	4.79E-10	1.03E-08	TMEM254	transmembrane protein 254	Multiple_Complex
TC1600010780.hg.1	6.5	8.47	-3.9	4.84E-10	1.04E-08	CMTR2	cap methyltransferase 2	Multiple_Complex
TC0500008309.hg.1	7.01	9.18	-4.48	4.88E-10	1.05E-08	CAMK4	calcium/calmodulin-dependent protein kinase	Multiple_Complex
TC0200006524.hg.1	7.39	9.01	-3.06	4.92E-10	1.05E-08	TRAPPC12	trafficking protein particle complex 12	Multiple_Complex
TC0500010635.hg.1	8.23	6.49	3.33	5.09E-10	1.09E-08	HMGCS1	3-hydroxy-3-methylglutaryl-CoA synthase 1 (s	Multiple_Complex
TC0500010842.hg.1	9.9	7.33	5.95	5.14E-10	1.10E-08	PDE4D	phosphodiesterase 4D, cAMP-specific	Multiple_Complex
TC0300007237.hg.1	5.68	7.26	-2.99	5.25E-10	1.12E-08	LIMD1	LIM domains containing 1	Multiple_Complex
TC0700006836.hg.1	11.28	6.73	23.4	5.25E-10	1.12E-08	RPL21	Homo sapiens ribosomal protein L21, mRNA ((	Multiple_Complex
TC1900010528.hg.1	5.16	7.12	-3.9	5.25E-10	1.12E-08	ZNF260	zinc finger protein 260	Multiple_Complex
TC0200014184.hg.1	9.86	11.15	-2.45	5.27E-10	1.12E-08	WDR33	WD repeat domain 33	Multiple_Complex
TC0600011943.hg.1	5.04	7.58	-5.83	5.32E-10	1.13E-08	ENPP5	ectonucleotide pyrophosphatase/phosphodie	Multiple_Complex
TC0M00006437.hg.1	18.25	19.49	-2.36	5.37E-10	1.14E-08	COX2	cytochrome c oxidase subunit II	Multiple_Complex
TC0300012426.hg.1	6.41	7.95	-2.92	5.44E-10	1.15E-08	PIK3R4	phosphoinositide-3-kinase, regulatory subunit	Multiple_Complex
TC1000011515.hg.1	5.17	7.04	-3.66	5.51E-10	1.17E-08	BLNK	B-cell linker	Multiple_Complex
TC0300008999.hg.1	8.52	10.99	-5.53	5.64E-10	1.19E-08	ZBTB38	zinc finger and BTB domain containing 38	Multiple_Complex
TC0600011162.hg.1	4.8	6.61	-3.51	5.66E-10	1.20E-08	ZNF322	zinc finger protein 322	Multiple_Complex
TC1900010076.hg.1	7.91	9.14	-2.35	5.73E-10	1.21E-08	ATP13A1	ATPase type 13A1	Multiple_Complex
TC1700008135.hg.1	6.07	7.33	-2.4	5.75E-10	1.21E-08	TBX21	T-box 21	Multiple_Complex
TC0600014111.hg.1	5.97	8.24	-4.84	5.78E-10	1.22E-08	SYNGAP1; I	synaptic Ras GTPase activating protein 1; micr	Multiple_Complex
TC1200006787.hg.1	11.8	9.6	4.59	5.84E-10	1.23E-08	GABARAPL	GABA(A) receptor-associated protein like 1	Multiple_Complex
TC0100012846.hg.1	6.65	8.08	-2.71	5.85E-10	1.23E-08	DFFA	DNA fragmentation factor, 45kDa, alpha polyp	Multiple_Complex
TC1900010502.hg.1	8.08	6.26	3.52	5.86E-10	1.23E-08	NFKBID	nuclear factor of kappa light polypeptide gene	Multiple_Complex
TC0100016206.hg.1	5.88	9.37	-11.23	5.87E-10	1.23E-08	SH2D1B	SH2 domain containing 1B	Multiple_Complex
TC1900010391.hg.1	7.25	8.8	-2.92	5.88E-10	1.23E-08	PEPD	peptidase D	Multiple_Complex
TC0600009228.hg.1	6.75	8.16	-2.66	5.92E-10	1.24E-08	NT5DC1	5-nucleotidase domain containing 1	Multiple_Complex
TC0X00007188.hg.1	6.38	7.56	-2.28	5.94E-10	1.24E-08	FTSJ1	FtsJ RNA methyltransferase homolog 1 (E. coli	Multiple_Complex
TC0300011152.hg.1	4.6	6.08	-2.79	5.98E-10	1.25E-08	DUSP7	dual specificity phosphatase 7	Multiple_Complex
TC1000006707.hg.1	5.88	7.39	-2.85	6.04E-10	1.26E-08	GATA3	GATA binding protein 3	Multiple_Complex
TC1000008926.hg.1	8.19	10	-3.5	6.05E-10	1.26E-08	ACSL5	acyl-CoA synthetase long-chain family membe	Multiple_Complex
TC0600009808.hg.1	10.24	8.45	3.47	6.08E-10	1.27E-08	PCMT1	protein-L-isoaspartate (D-aspartate) O-methyl	Multiple_Complex
TC0600010375.hg.1	5.74	6.93	-2.28	6.17E-10	1.29E-08	EXOC2	exocyst complex component 2	Multiple_Complex
TC0700012998.hg.1	7.89	9.32	-2.7	6.23E-10	1.30E-08	ZNF767P	zinc finger family member 767, pseudogene	Multiple_Complex
TC0900011259.hg.1	5.38	6.55	-2.24	6.24E-10	1.30E-08	HDHD3	haloacid dehalogenase-like hydrolase domain	Multiple_Complex
TC1300006675.hg.1	13.23	7.88	40.93	6.26E-10	1.30E-08	SNORD102	small nucleolar RNA, C/D box 102; small nucle	Multiple_Complex
TC0300007606.hg.1	5.5	6.85	-2.55	6.31E-10	1.31E-08	ABHD6	abhydrolase domain containing 6	Multiple_Complex
TC1900011720.hg.1	6.47	5.4	2.09	6.40E-10	1.33E-08	HKR1	HKR1, GLI-Kruppel zinc finger family member	NonCoding
TC1100012012.hg.1	7.12	5.1	4.07	6.45E-10	1.34E-08	CWC15	CWC15 spliceosome-associated protein	Multiple_Complex
TC1900008257.hg.1	4.61	6.65	-4.11	6.47E-10	1.34E-08	ZNF227	zinc finger protein 227	Multiple_Complex
TC2200007626.hg.1	9.23	10.91	-3.21	6.47E-10	1.34E-08	ATXN10	ataxin 10	Multiple_Complex
TC0800012285.hg.1	7.79	8.96	-2.25	6.48E-10	1.34E-08	HMBOX1	homeobox containing 1	Multiple_Complex
TC0700012427.hg.1	9.05	10.15	-2.14	6.50E-10	1.34E-08	FAM3C	family with sequence similarity 3, member C	Multiple_Complex
TC1500010292.hg.1	12.25	10.62	3.1	6.51E-10	1.34E-08	SEC11A	SEC11 homolog A, signal peptidase complex sı	Multiple_Complex
TC1700010763.hg.1	7.07	5.53	2.93	6.52E-10	1.34E-08	BRCA1	breast cancer 1, early onset	Multiple_Complex
TC0200016502.hg.1	9.84	7.18	6.34	6.78E-10	1.40E-08	IL18R1	interleukin 18 receptor 1	Multiple_Complex
TC0600013206.hg.1	6.79	8.3	-2.85	6.84E-10	1.40E-08	SLC18B1	solute carrier family 18, subfamily B, member	Multiple_Complex

TC0700013510.hg.1	9.53	11.31	-3.43	6.86E-10	1.41E-08	KDELR2	KDEL (Lys-Asp-Glu-Leu) endoplasmic reticulum	Multiple_Complex
TC1100011280.hg.1	5.77	6.85	-2.11	6.94E-10	1.42E-08	YIF1A	Yip1 interacting factor homolog A (S. cerevisia	Multiple_Complex
TC2200008001.hg.1	7.74	9.23	-2.81	7.02E-10	1.44E-08	TXNRD2	thioredoxin reductase 2	Multiple_Complex
TC1400007603.hg.1	6.69	4.95	3.34	7.11E-10	1.45E-08	RGS6	regulator of G-protein signaling 6	Multiple_Complex
TC0200015506.hg.1	9.05	10.37	-2.51	7.13E-10	1.46E-08	INO80D	INO80 complex subunit D	Multiple_Complex
TC0100016944.hg.1	11.88	10.16	3.3	7.15E-10	1.46E-08	ARL8A	ADP-ribosylation factor like GTPase 8A	Multiple_Complex
TC1100007790.hg.1	7.25	9.79	-5.8	7.44E-10	1.52E-08	CD5	CD5 molecule	Multiple_Complex
TC1100011014.hg.1	7.79	6.13	3.17	7.44E-10	1.52E-08	SLC15A3	solute carrier family 15 (oligopeptide transpor	Multiple_Complex
TC0400008984.hg.1	14.97	9.49	44.43	7.53E-10	1.53E-08	RPS3A; SNO	ribosomal protein S3A; small nucleolar RNA, C	Multiple_Complex
TC1500007510.hg.1	7.8	5.46	5.09	7.53E-10	1.53E-08	TPM1	tropomyosin 1 (alpha)	Multiple_Complex
TC1200009375.hg.1	12.54	10.17	5.14	7.55E-10	1.53E-08	GLT1D1	glycosyltransferase 1 domain containing 1	Multiple_Complex
TC0100015330.hg.1	7.32	5.72	3.03	7.56E-10	1.54E-08	PHTF1	putative homeodomain transcription factor 1	Multiple_Complex
TC1600009746.hg.1	6.89	8	-2.16	7.58E-10	1.54E-08	PALB2	partner and localizer of BRCA2	Multiple_Complex
TC1400009147.hg.1	14.55	10.97	11.98	7.59E-10	1.54E-08	PYGL	phosphorylase, glycogen, liver	Multiple_Complex
TSUnmapped00000068.h	5.08	6.73	-3.15	7.62E-10	1.54E-08	ZNF780A	zinc finger protein 780A	Coding
TC0200012994.hg.1	8.57	7.05	2.88	7.64E-10	1.54E-08	SNRPG	small nuclear ribonucleoprotein polypeptide G	Multiple_Complex
TC1100008144.hg.1	8.97	11.02	-4.14	7.74E-10	1.56E-08	GSTP1	glutathione S-transferase pi 1	Multiple_Complex
TC1900011754.hg.1	3.43	5.19	-3.38	7.75E-10	1.56E-08	ZNF225	zinc finger protein 225	Multiple_Complex
TC0200010112.hg.1	5.65	6.94	-2.44	7.75E-10	1.56E-08	RBM45	RNA binding motif protein 45	Multiple_Complex
TC1100010887.hg.1	6.31	8.03	-3.29	7.77E-10	1.57E-08	SSRP1	structure specific recognition protein 1	Multiple_Complex
TC0900008984.hg.1	9.04	11.02	-3.94	7.88E-10	1.59E-08	NUP214	nucleoporin 214kDa	Multiple_Complex
TC0X00009268.hg.1	7.35	4.6	6.7	7.90E-10	1.59E-08	PCYT1B	phosphate cytidylyltransferase 1, choline, beta	Multiple_Complex
TC1200010794.hg.1	6.87	8.64	-3.39	7.95E-10	1.60E-08	ITGB7	integrin beta 7	Multiple_Complex
TC0800008715.hg.1	7.89	9.29	-2.62	7.97E-10	1.60E-08	ZHX2	zinc fingers and homeoboxes 2	Coding
TC0100010499.hg.1	9.59	11.68	-4.24	8.06E-10	1.62E-08	POU2F1	POU class 2 homeobox 1	Multiple_Complex
TC1100008270.hg.1	8.92	7.16	3.41	8.17E-10	1.64E-08	CTTN	cortactin	Multiple_Complex
TC1800008235.hg.1	6.09	8.04	-3.88	8.19E-10	1.64E-08	ABHD3	Transcript Identified by AceView, Entrez Gene	Unassigned
TC1100013015.hg.1	6.49	8.93	-5.42	8.19E-10	1.64E-08	MS4A14	membrane-spanning 4-domains, subfamily A,	Multiple_Complex
TC0900007663.hg.1	6.29	8.76	-5.55	8.31E-10	1.66E-08	CEP78	centrosomal protein 78kDa	Multiple_Complex
TC0100012673.hg.1	6.75	7.94	-2.28	8.33E-10	1.66E-08	ICMT	isoprenylcysteine carboxyl methyltransferase	Multiple_Complex
TC0100011253.hg.1	12.04	10.3	3.33	8.56E-10	1.71E-08	BTG2	BTG family, member 2	Multiple_Complex
TC0600012003.hg.1	8.09	5.66	5.39	8.67E-10	1.73E-08	CRISP3	cysteine-rich secretory protein 3	Coding
TC0200016115.hg.1	8.15	10.68	-5.79	8.75E-10	1.74E-08	ARL4C	ADP-ribosylation factor like GTPase 4C	Multiple_Complex
TC1600011368.hg.1	8.96	11	-4.11	8.79E-10	1.75E-08	LAT	linker for activation of T-cells	Multiple_Complex
TC1200011527.hg.1	14.23	9.96	19.39	8.91E-10	1.77E-08	RPL41P5	ribosomal protein L41 pseudogene 5	Multiple_Complex
TC0600012061.hg.1	6.15	8.03	-3.69	8.93E-10	1.77E-08	ICK	intestinal cell (MAK-like) kinase	Multiple_Complex
TC1700011237.hg.1	9.2	10.26	-2.08	9.04E-10	1.79E-08	VEZF1	vascular endothelial zinc finger 1	Multiple_Complex
TC0100013293.hg.1	3.44	5.21	-3.41	9.08E-10	1.80E-08	ID3	inhibitor of DNA binding 3, dominant negative	Multiple_Complex
TC0300013912.hg.1	6.63	5.46	2.26	9.11E-10	1.80E-08	DNAJB11	DnaJ (Hsp40) homolog, subfamily B, member	Coding
TC0100013588.hg.1	15.14	13.68	2.75	9.17E-10	1.81E-08	PTP4A2	protein tyrosine phosphatase type IVA, memb	Multiple_Complex
TC0200016586.hg.1	9.6	7.9	3.24	9.17E-10	1.81E-08	HSPE1-MO	HSPE1-MOB4 readthrough	Coding
TC1800006657.hg.1	9.5	7.7	3.49	9.19E-10	1.81E-08	VAPA	Memczak2013 ALT_ACCEPTOR, ALT_DONOR,	NonCoding
TC0600014276.hg.1	9.65	11.5	-3.59	9.27E-10	1.83E-08	HLA-DMB	major histocompatibility complex, class II, DM	Multiple_Complex
TC0600009455.hg.1	8.01	6.03	3.95	9.33E-10	1.84E-08	ARG1	arginase 1	Multiple_Complex
TC2000007169.hg.1	11.13	9.17	3.9	9.36E-10	1.84E-08	CHMP4B	charged multivesicular body protein 4B	Multiple_Complex
TC0300008715.hg.1	5.69	7.25	-2.96	9.40E-10	1.85E-08	EEFSEC	eukaryotic elongation factor, selenocysteine-t	Multiple_Complex
TC1900012040.hg.1	10.5	8.85	3.13	9.42E-10	1.85E-08	LILRA5	leukocyte immunoglobulin-like receptor, subfa	Multiple_Complex
TC0300009282.hg.1	10.86	9.05	3.52	9.47E-10	1.86E-08	TIPARP	TCDD-inducible poly(ADP-ribose) polymerase	Multiple_Complex
TC1500009005.hg.1	6.43	8	-2.95	9.56E-10	1.87E-08	ZNF770	zinc finger protein 770	Multiple_Complex
TC1100007787.hg.1	7.85	10.63	-6.83	9.56E-10	1.87E-08	CD6	CD6 molecule	Multiple_Complex
TC1100013025.hg.1	6.57	7.85	-2.42	9.58E-10	1.88E-08	TTC9C	tetratricopeptide repeat domain 9C	Multiple_Complex
TC2200008186.hg.1	6.01	7.61	-3.04	9.59E-10	1.88E-08	CHCHD10	coiled-coil-helix-coiled-coil-helix domain conta	Multiple_Complex
TC0900012167.hg.1	10.42	8.64	3.42	9.80E-10	1.92E-08	GSN	gelsolin	Multiple_Complex
TC2200007973.hg.1	10.33	9.03	2.46	9.81E-10	1.92E-08	DGCR14	Transcript Identified by AceView, Entrez Gene	Unassigned
TC1100013222.hg.1	13.37	10.12	9.53	9.89E-10	1.93E-08	CARD16	caspase recruitment domain family, member 1	Multiple_Complex
TC1700009535.hg.1	5.84	4.09	3.36	9.93E-10	1.94E-08	CHRNE	cholinergic receptor, nicotinic epsilon	Multiple_Complex
TC0800007766.hg.1	12.54	11.21	2.51	9.98E-10	1.94E-08	RAB2A	RAB2A, member RAS oncogene family	Multiple_Complex
TC0300013701.hg.1	11.22	8.81	5.3	1.00E-09	1.95E-08	UBXN7	UBX domain protein 7	Multiple_Complex
TC1700009293.hg.1	7.77	6.2	2.96	1.02E-09	1.98E-08	METRNL	meteorin, glial cell differentiation regulator-lik	Multiple_Complex
TC0300012000.hg.1	12.07	10.25	3.51	1.02E-09	1.99E-08	ATG3	autophagy related 3	Multiple_Complex
TC0200007200.hg.1	10.04	7.27	6.82	1.02E-09	1.99E-08	LTBP1	latent transforming growth factor beta binding	Multiple_Complex
TC0600007598.hg.1	17.85	13.59	19.09	1.04E-09	2.02E-08	AIF1	allograft inflammatory factor 1	Multiple_Complex
TC1700010982.hg.1	6.11	8.17	-4.15	1.04E-09	2.02E-08	SKAP1	src kinase associated phosphoprotein 1	Multiple_Complex
TC1000012025.hg.1	9.74	11.04	-2.46	1.07E-09	2.07E-08	MCMBP	minichromosome maintenance complex bindi	Multiple_Complex
TC0500009055.hg.1	6.91	4.74	4.49	1.07E-09	2.08E-08	ABLIM3	actin binding LIM protein family, member 3	Multiple_Complex
TC0500010585.hg.1	15.12	10.48	24.89	1.09E-09	2.10E-08	RPL37	ribosomal protein L37	Multiple_Complex
TC0500011212.hg.1	8.38	5.77	6.08	1.09E-09	2.11E-08	TBCA	tubulin folding cofactor A	Multiple_Complex
TC0600011913.hg.1	7.65	6.52	2.19	1.10E-09	2.12E-08	NFKBIE	nuclear factor of kappa light polypeptide gene	Multiple_Complex
TC0200016645.hg.1	8.99	10.46	-2.77	1.11E-09	2.14E-08	ASXL2	additional sex combs like transcriptional regul	Multiple_Complex
TC0100016364.hg.1	7.55	8.89	-2.53	1.12E-09	2.15E-08	SCYL3	SCY1-like, kinase-like 3	Multiple_Complex

TC0200014190.hg.1	6.45	8	-2.94	1.12E-09	2.15E-08	AMMECR1L	AMMECR1 like	Multiple_Complex
TC1100009166.hg.1	13.31	11.48	3.57	1.13E-09	2.17E-08	PAFAH1B2	platelet-activating factor acetylhydrolase 1b, c	Multiple_Complex
TC1100007876.hg.1	13.98	15.94	-3.9	1.13E-09	2.17E-08	TMEM179B	transmembrane protein 179B; microRNA 6748	Multiple_Complex
TC1000009172.hg.1	9.28	11	-3.31	1.13E-09	2.17E-08	BUB3	BUB3 mitotic checkpoint protein	Multiple_Complex
TC1100013110.hg.1	11.89	14.18	-4.88	1.14E-09	2.18E-08	STT3A	STT3A, subunit of the oligosaccharyltransferas	Multiple_Complex
TC1700008218.hg.1	5.15	6.59	-2.72	1.14E-09	2.19E-08	FLJ45513; F	uncharacterized LOC729220; novel transcript	Multiple_Complex
TC1900008992.hg.1	3.99	5.65	-3.16	1.15E-09	2.19E-08	ZNF543	zinc finger protein 543	Multiple_Complex
TC1900008372.hg.1	8.33	9.5	-2.25	1.16E-09	2.21E-08	PPP5C	protein phosphatase 5, catalytic subunit	Multiple_Complex
TC1800007385.hg.1	6.94	8.35	-2.66	1.20E-09	2.28E-08	WDR7	WD repeat domain 7	Multiple_Complex
TC0100017748.hg.1	7.85	6.06	3.46	1.21E-09	2.30E-08	SIPA1L2	signal-induced proliferation-associated 1 like 2	Multiple_Complex
TC1700012245.hg.1	5.56	6.84	-2.42	1.21E-09	2.30E-08	CDK12	cyclin-dependent kinase 12	NonCoding
TC1700012445.hg.1	8.39	10.14	-3.36	1.22E-09	2.32E-08	STRADA	STE20-related kinase adaptor alpha	Multiple_Complex
TC0600011635.hg.1	13.45	10.92	5.79	1.22E-09	2.32E-08	FKBP5	FK506 binding protein 5	Multiple_Complex
TC1100007899.hg.1	8	10.37	-5.19	1.26E-09	2.39E-08	RARRES3	retinoic acid receptor responder (tazarotene ir	Multiple_Complex
TC1000012603.hg.1	9.82	11.38	-2.94	1.27E-09	2.42E-08	FAM53B	family with sequence similarity 53, member B	Multiple_Complex
TC0700013626.hg.1	11.58	9.37	4.62	1.27E-09	2.42E-08	SLC37A3	solute carrier family 37, member 3	Multiple_Complex
TC0100016049.hg.1	6.95	9.97	-8.11	1.27E-09	2.42E-08	FCRL3	Fc receptor-like 3	Multiple_Complex
TC0900009882.hg.1	10.39	14.95	-23.63	1.29E-09	2.44E-08	DCAF12	DDB1 and CUL4 associated factor 12	Multiple_Complex
TC0200015771.hg.1	14.69	13.02	3.18	1.30E-09	2.45E-08	TUBA4A	tubulin, alpha 4a	Multiple_Complex
TC1500009702.hg.1	8.19	5.59	6.06	1.31E-09	2.47E-08	RPS27L	ribosomal protein S27-like	Multiple_Complex
TC1900007125.hg.1	6.06	7.94	-3.67	1.31E-09	2.48E-08	NACC1	nucleus accumbens associated 1, BEN and BTE	Multiple_Complex
TC0400010904.hg.1	12.95	11.21	3.35	1.33E-09	2.50E-08	YTHDC1	YTH domain containing 1	Multiple_Complex
TC1900008019.hg.1	11.52	13.1	-2.99	1.34E-09	2.53E-08	EIF3K	eukaryotic translation initiation factor 3, subu	Multiple_Complex
TC0300011450.hg.1	10.22	8.93	2.44	1.34E-09	2.53E-08	TMF1	TATA element modulatory factor 1	Multiple_Complex
TC1500009363.hg.1	10.47	8.02	5.48	1.35E-09	2.54E-08	COPS2	COP9 signalosome subunit 2	Multiple_Complex
TC0200015266.hg.1	4.99	3.62	2.58	1.37E-09	2.59E-08	TMEFF2	transmembrane protein with EGF-like and two	Multiple_Complex
TC1000007779.hg.1	6.27	7.39	-2.18	1.38E-09	2.59E-08	ADO	2-aminoethanethiol (cysteamine) dioxygenase	Coding
TC1900007012.hg.1	8.57	6.88	3.22	1.39E-09	2.60E-08	LDLR; MIR6	low density lipoprotein receptor; microRNA 68	Multiple_Complex
TC0700012970.hg.1	4.89	6.07	-2.26	1.39E-09	2.61E-08	ZNF786	zinc finger protein 786	Multiple_Complex
TC1900011952.hg.1	7.04	8.71	-3.18	1.39E-09	2.61E-08	ZNF780A	zinc finger protein 780A	Multiple_Complex
TC1500009120.hg.1	7.08	8.3	-2.32	1.41E-09	2.64E-08	RMDN3	regulator of microtubule dynamics 3	Multiple_Complex
TC1700011785.hg.1	7.3	9.16	-3.63	1.41E-09	2.64E-08	EXOC7	Zhang2013 ALT_ACCEPTOR, ALT_DONOR, cod	NonCoding
TC2000008968.hg.1	5.85	7.24	-2.63	1.42E-09	2.66E-08	UQCC1	ubiquinol-cytochrome c reductase complex as	Multiple_Complex
TC0300008320.hg.1	9.3	12.16	-7.28	1.44E-09	2.69E-08	CD96	CD96 molecule	Multiple_Complex
TC0400012992.hg.1	7.45	5.53	3.79	1.44E-09	2.69E-08	MFAP3L	microfibrillar associated protein 3 like	Coding
TC1000008988.hg.1	6.55	7.62	-2.1	1.45E-09	2.70E-08	TRUB1	TruB pseudouridine (psi) synthase family mem	Multiple_Complex
TC0200011086.hg.1	5.87	7.64	-3.41	1.46E-09	2.71E-08	DIS3L2	DIS3 like 3-5 exoribonuclease 2	Multiple_Complex
TC1300008181.hg.1	7.29	9.06	-3.42	1.47E-09	2.74E-08	CHAMP1	chromosome alignment maintaining phospho	Multiple_Complex
TC1600008657.hg.1	8.65	10.34	-3.21	1.48E-09	2.75E-08	USP10	ubiquitin specific peptidase 10	Multiple_Complex
TC1200008081.hg.1	6.43	8.09	-3.16	1.48E-09	2.75E-08	DYRK2	dual specificity tyrosine-(Y)-phosphorylation r	Multiple_Complex
TC0200016442.hg.1	6.27	8.09	-3.51	1.50E-09	2.78E-08	MSH2	mutS homolog 2	Multiple_Complex
TC0300008002.hg.1	9.3	8.16	2.2	1.54E-09	2.85E-08	CHMP2B	charged multivesicular body protein 2B	Multiple_Complex
TC1700007127.hg.1	9.19	11.69	-5.66	1.57E-09	2.91E-08	LGALS9C; L	lectin, galactoside-binding, soluble, 9C; lectin,	Multiple_Complex
TC1800007820.hg.1	10.28	9.49	1.72	1.57E-09	2.91E-08	CTDP1	CTD phosphatase subunit 1	Multiple_Complex
TC0600007677.hg.1	10.95	14.6	-12.55	1.59E-09	2.95E-08	HLA-DPB1	major histocompatibility complex, class II, DP	Multiple_Complex
TC1900007127.hg.1	11.24	8.81	5.39	1.61E-09	2.97E-08	IER2	immediate early response 2	Coding
TC0200012073.hg.1	6.61	8.21	-3.04	1.63E-09	3.00E-08	GTF3C2	general transcription factor IIIC subunit 2	Multiple_Complex
TC1100011545.hg.1	8.38	6.42	3.9	1.64E-09	3.02E-08	ANAPC15	anaphase promoting complex subunit 15	Multiple_Complex
TC0800012306.hg.1	4.85	6.26	-2.65	1.65E-09	3.04E-08	MTFR1	mitochondrial fission regulator 1	NonCoding
TC1600011350.hg.1	6	7.46	-2.74	1.65E-09	3.04E-08	PMM2	phosphomannomutase 2	Multiple_Complex
TC0100015819.hg.1	14.26	16.04	-3.44	1.66E-09	3.06E-08	S100A10	S100 calcium binding protein A10	Multiple_Complex
TC0800012176.hg.1	8.81	7.24	2.96	1.66E-09	3.06E-08	PLEC	plectin	Multiple_Complex
TC1800006902.hg.1	7.17	8.68	-2.84	1.67E-09	3.06E-08	TTC39C	tetratricopeptide repeat domain 39C	Multiple_Complex
TC0300007124.hg.1	5.77	6.95	-2.26	1.68E-09	3.08E-08	ZNF621	zinc finger protein 621	Multiple_Complex
TC0800007004.hg.1	5.02	6.62	-3.04	1.69E-09	3.10E-08	RHOBTB2	Rho-related BTB domain containing 2	Multiple_Complex
TC1200009235.hg.1	7.32	8.6	-2.43	1.70E-09	3.12E-08	C12orf65	chromosome 12 open reading frame 65	Multiple_Complex
TC0900011501.hg.1	6.46	4.98	2.8	1.71E-09	3.14E-08	NR6A1	nuclear receptor subfamily 6, group A, membe	Multiple_Complex
TC1100006576.hg.1	8.67	10.46	-3.44	1.72E-09	3.14E-08	CD81	CD81 molecule	Multiple_Complex
TC1700009318.hg.1	13.39	10.42	7.83	1.72E-09	3.14E-08	FAM101B	family with sequence similarity 101, member	Multiple_Complex
TC0300008114.hg.1	5.05	7.21	-4.47	1.72E-09	3.15E-08	GPR15	G protein-coupled receptor 15	Coding
TC1000009873.hg.1	7.76	8.85	-2.13	1.74E-09	3.18E-08	DCLRE1C	DNA cross-link repair 1C	Multiple_Complex
TC1000012088.hg.1	5.83	6.9	-2.09	1.76E-09	3.20E-08	C10orf88	chromosome 10 open reading frame 88	Multiple_Complex
TC0700013400.hg.1	8.45	10.02	-2.98	1.78E-09	3.24E-08	GATAD1	GATA zinc finger domain containing 1	Multiple_Complex
TC1900011793.hg.1	7.68	9.23	-2.92	1.79E-09	3.25E-08	ZNF808; RF	zinc finger protein 808; ribosomal protein L39	Multiple_Complex
TC0300006942.hg.1	12.53	13.99	-2.75	1.79E-09	3.25E-08	STT3B	STT3B, subunit of the oligosaccharyltransferas	Multiple_Complex
TC1400006686.hg.1	8.47	5.52	7.74	1.80E-09	3.28E-08	CMTM5	CKLF-like MARVEL transmembrane domain co	Multiple_Complex
TC0600007262.hg.1	6.92	4.88	4.12	1.81E-09	3.28E-08	HIST1H3A	histone cluster 1, H3a	Coding
TC1100012126.hg.1	12.32	6.8	45.7	1.82E-09	3.29E-08	MMP8	matrix metallopeptidase 8	Coding
TC1000010725.hg.1	11.35	12.77	-2.69	1.82E-09	3.31E-08	CCDC6	coiled-coil domain containing 6	Multiple_Complex
TC0500012238.hg.1	7.88	6.02	3.64	1.84E-09	3.34E-08	HBEGF	heparin-binding EGF-like growth factor	Multiple_Complex

TC0200016686.hg.1	8.1	9.6	-2.83	1.85E-09	3.35E-08	TEX261	testis expressed 261	Multiple_Complex
TC1900007988.hg.1	7.66	9.38	-3.29	1.87E-09	3.38E-08	SIPA1L3	signal-induced proliferation-associated 1 like 3	Multiple_Complex
TC0800008146.hg.1	4.89	7.01	-4.32	1.87E-09	3.38E-08	WWP1	Transcript Identified by AceView, Entrez Gene	Unassigned
TC0X00009611.hg.1	7.67	10.98	-9.88	1.88E-09	3.39E-08	SLC38A5	solute carrier family 38, member 5	Multiple_Complex
TC0100009560.hg.1	7.43	8.76	-2.51	1.88E-09	3.40E-08	CD101	CD101 molecule	Multiple_Complex
TC2000009440.hg.1	10.11	11.67	-2.95	1.90E-09	3.42E-08	ADNP	activity-dependent neuroprotector homeobox	Multiple_Complex
TC1100013161.hg.1	9.91	8.14	3.41	1.90E-09	3.42E-08	CD59	CD59 molecule, complement regulatory prote	Multiple_Complex
TC2000007819.hg.1	6.04	7.64	-3.04	1.94E-09	3.48E-08	CSTF1	cleavage stimulation factor, 3 pre-RNA, subun	Multiple_Complex
TC0700007905.hg.1	6.18	8.02	-3.59	1.95E-09	3.50E-08	AUTS2	autism susceptibility candidate 2	Multiple_Complex
TC1000011825.hg.1	10.01	8.84	2.25	1.95E-09	3.50E-08	BBIP1	BBSome interacting protein 1	Multiple_Complex
TC2200008578.hg.1	10.24	6.23	16.07	2.00E-09	3.58E-08	RPL41	Synthetic construct Homo sapiens clone IMAG	Multiple_Complex
TC1400009980.hg.1	9.52	11.24	-3.28	2.02E-09	3.63E-08	CCDC88C	coiled-coil domain containing 88C	Multiple_Complex
TC0X00010357.hg.1	7.92	5.8	4.33	2.05E-09	3.67E-08	GLA	galactosidase, alpha	Multiple_Complex
TC1000007954.hg.1	4.6	6.37	-3.4	2.06E-09	3.69E-08	SLC29A3	solute carrier family 29 (equilibrative nucleosi	Multiple_Complex
TC0900011669.hg.1	6.86	8.32	-2.75	2.06E-09	3.69E-08	SH3GLB2	SH3-domain GRB2-like endophilin B2	Multiple_Complex
TC0200016662.hg.1	12.12	9.01	8.65	2.07E-09	3.70E-08	CALM2	calmodulin 2 (phosphorylase kinase, delta)	Multiple_Complex
TC0900006969.hg.1	10.13	8.08	4.15	2.07E-09	3.70E-08	CHMP5	charged multivesicular body protein 5	Multiple_Complex
TC1100013190.hg.1	14.74	16.44	-3.25	2.12E-09	3.78E-08	CFL1	cofilin 1 (non-muscle)	Multiple_Complex
TC1500009585.hg.1	8.35	6.7	3.13	2.14E-09	3.81E-08	SLTM	SAFB-like, transcription modulator	Multiple_Complex
TC1200012753.hg.1	6.31	9.76	-10.9	2.15E-09	3.83E-08	KLRC2	killer cell lectin-like receptor subfamily C, mem	Coding
TC0100013472.hg.1	10.28	7.88	5.28	2.16E-09	3.85E-08	DNAJC8	DnaJ (Hsp40) homolog, subfamily C, member	Multiple_Complex
TC1900009637.hg.1	8.27	6.6	3.17	2.17E-09	3.85E-08	CDKN2D	cyclin-dependent kinase inhibitor 2D (p19, inh	Coding
TC0600014261.hg.1	11.07	12.43	-2.57	2.18E-09	3.87E-08	DDX39B; SI	DEAD (Asp-Glu-Ala-Asp) box polypeptide 39B;	Multiple_Complex
TC0200007900.hg.1	11.26	13.21	-3.85	2.18E-09	3.87E-08	ARHGAP25	Rho GTPase activating protein 25	Multiple_Complex
TC1900011251.hg.1	11.46	14.77	-9.94	2.18E-09	3.87E-08	NKG7	natural killer cell granule protein 7	Coding
TC1500010237.hg.1	10.71	6.44	19.31	2.20E-09	3.89E-08	RPL9	ribosomal protein L9	Multiple_Complex
TC0300006669.hg.1	8.55	5.35	9.2	2.20E-09	3.90E-08	LSM3	LSM3 homolog, U6 small nuclear RNA and mR	Multiple_Complex
TC0300010424.hg.1	7.57	5.95	3.09	2.21E-09	3.91E-08	DPH3	diphthamide biosynthesis 3	Multiple_Complex
TC2200009234.hg.1	11.86	9.79	4.23	2.22E-09	3.92E-08	SPECC1L-AI	SPECC1L-ADORA2A readthrough (NMD candid	Multiple_Complex
TC2200009356.hg.1	9.3	7.76	2.9	2.22E-09	3.92E-08	ARFGAP3	ADP-ribosylation factor GTPase activating prot	Multiple_Complex
TC0500009707.hg.1	6.51	4.93	2.98	2.24E-09	3.96E-08	SQSTM1	Transcript Identified by AceView, Entrez Gene	Unassigned
TC0600010647.hg.1	6.3	7.95	-3.14	2.28E-09	4.02E-08	ECI2	enoyl-CoA delta isomerase 2	Multiple_Complex
TC1100009450.hg.1	5.6	6.92	-2.5	2.30E-09	4.06E-08	DCPS	decapping enzyme, scavenger	Multiple_Complex
TC0100016162.hg.1	5.7	7.44	-3.34	2.32E-09	4.09E-08	B4GALT3	UDP-Gal:betaGlcNAc beta 1,4- galactosyltrans	Multiple_Complex
TC1600008259.hg.1	7.91	9.7	-3.46	2.32E-09	4.09E-08	PDPR	pyruvate dehydrogenase phosphatase regulat	Multiple_Complex
TSUnmapped00000095.h	5.44	4.34	2.15	2.33E-09	4.09E-08	NDUFA6	NADH dehydrogenase (ubiquinone) 1 alpha su	Coding
TC1800007360.hg.1	10.96	8.43	5.77	2.33E-09	4.09E-08	RAB27B	RAB27B, member RAS oncogene family	Multiple_Complex
TC0100017505.hg.1	9.6	8.37	2.33	2.34E-09	4.11E-08	SDE2	SDE2 telomere maintenance homolog (S. pom	Multiple_Complex
TC1400008752.hg.1	8.74	10.77	-4.08	2.35E-09	4.11E-08	CIDEB	cell death-inducing DFFA-like effector b	Multiple_Complex
TC1200012716.hg.1	11.56	9.84	3.29	2.38E-09	4.17E-08	COX6A1	cytochrome c oxidase subunit VIa polypeptide	Multiple_Complex
TC0700008552.hg.1	4.84	6.34	-2.83	2.40E-09	4.20E-08	AGFG2	ArfGAP with FG repeats 2	Multiple_Complex
TC1700012216.hg.1	11.95	14.8	-7.18	2.43E-09	4.25E-08	LGALS9	lectin, galactoside-binding, soluble, 9	Multiple_Complex
TC1600007952.hg.1	7.61	9.25	-3.11	2.44E-09	4.27E-08	OGFOD1	2-oxoglutarate and iron-dependent oxygenase	Multiple_Complex
TC2200008704.hg.1	6.72	4.91	3.49	2.50E-09	4.36E-08	DMC1	DNA meiotic recombinase 1	Multiple_Complex
TC0200010445.hg.1	7.37	8.7	-2.51	2.52E-09	4.39E-08	CASP10	caspase 10	Multiple_Complex
TC1600011186.hg.1	9.13	7.03	4.29	2.53E-09	4.42E-08	SLC7A5	solute carrier family 7 (amino acid transporter	Multiple_Complex
TC0700011698.hg.1	10.75	12.29	-2.92	2.55E-09	4.44E-08	TMEM243	transmembrane protein 243, mitochondrial	Multiple_Complex
TC0600011525.hg.1	9.31	10.5	-2.28	2.56E-09	4.45E-08	RXRB	retinoid X receptor beta	Multiple_Complex
TC0800010823.hg.1	9.7	8.15	2.92	2.56E-09	4.45E-08	TCEB1	transcription elongation factor B (SIII), polypep	Multiple_Complex
TC0900008250.hg.1	6.2	8.04	-3.59	2.57E-09	4.46E-08	ZNF189	zinc finger protein 189	Multiple_Complex
TC1500006663.hg.1	11.43	7.54	14.8	2.58E-09	4.48E-08	RPL41P2	ribosomal protein L41 pseudogene 2	Multiple_Complex
TC0600011282.hg.1	7	8.74	-3.33	2.61E-09	4.53E-08	TRIM27	tripartite motif containing 27	Multiple_Complex
TC1100010349.hg.1	6.73	5.19	2.91	2.62E-09	4.54E-08	SVIP	small VCP/p97-interacting protein	Multiple_Complex
TC1600009417.hg.1	10.86	9.63	2.35	2.65E-09	4.60E-08	ZC3H7A	zinc finger CCCH-type containing 7A	Multiple_Complex
TC0200013875.hg.1	9.36	7.56	3.46	2.69E-09	4.65E-08	RGPD8; RG	RANBP2-like and GRIP domain containing 8; R	Multiple_Complex
TC2200007196.hg.1	10.98	9.47	2.84	2.70E-09	4.66E-08	TOM1	target of myb1 membrane trafficking protein	Multiple_Complex
TC0300013865.hg.1	7.07	8.16	-2.13	2.76E-09	4.77E-08	SEC22A	SEC22 homolog A, vesicle trafficking protein	Multiple_Complex
TC0100015561.hg.1	6.17	7.81	-3.12	2.78E-09	4.79E-08	NBPF15	neuroblastoma breakpoint family, member 15	Multiple_Complex
TC0600008132.hg.1	8.7	7.07	3.08	2.80E-09	4.82E-08	CDC5L	cell division cycle 5-like	Multiple_Complex
TSUnmapped00000148.h	9.26	7.09	4.52	2.80E-09	4.82E-08	PADI4	peptidyl arginine deiminase, type IV	NonCoding
TC1700010599.hg.1	7.39	9.04	-3.14	2.87E-09	4.95E-08	MED24; MI	mediator complex subunit 24; microRNA 6884	Multiple_Complex
TC1800007960.hg.1	7.54	8.57	-2.04	2.87E-09	4.95E-08	ZBTB14	zinc finger and BTB domain containing 14	Multiple_Complex
TC0200014764.hg.1	5.99	8.02	-4.11	2.88E-09	4.95E-08	DPP4	dipeptidyl-peptidase 4	Multiple_Complex
TC1100009453.hg.1	7.82	5.85	3.91	2.89E-09	4.96E-08	ST3GAL4	ST3 beta-galactoside alpha-2,3-sialyltransfera	Multiple_Complex
TC1700011435.hg.1	8.23	10.13	-3.72	2.89E-09	4.96E-08	ICAM2	intercellular adhesion molecule 2	Multiple_Complex
TC1400007710.hg.1	9.16	7.66	2.83	2.89E-09	4.96E-08	JDP2	Jun dimerization protein 2	Multiple_Complex
TC0500009587.hg.1	7.49	9.8	-4.98	2.90E-09	4.96E-08	HIGD2A	HIG1 hypoxia inducible domain family, memb	Multiple_Complex
TC0100013339.hg.1	8.63	11.97	-10.16	2.94E-09	5.04E-08	RUNX3	runt-related transcription factor 3	Multiple_Complex
TC1100008175.hg.1	6.09	7.59	-2.82	2.96E-09	5.07E-08	NDUFS8; M	NADH dehydrogenase (ubiquinone) Fe-S prote	Multiple_Complex
TC0500013359.hg.1	13.09	11.74	2.54	2.99E-09	5.11E-08	PPP2CA	protein phosphatase 2, catalytic subunit, alph	Multiple_Complex

TC0600012968.hg.1	6.41	4.78	3.1	3.02E-09	5.16E-08	TRAPPC3L	trafficking protein particle complex 3-like	Multiple_Complex
TC1700010426.hg.1	5.89	7.25	-2.57	3.02E-09	5.16E-08	SLFN13	schlafen family member 13	Multiple_Complex
TC1700009528.hg.1	12.36	9.72	6.22	3.07E-09	5.23E-08	CXCL16	chemokine (C-X-C motif) ligand 16	Multiple_Complex
TC1600009944.hg.1	7.03	8.43	-2.63	3.08E-09	5.25E-08	KCTD13	potassium channel tetramerization domain co	Multiple_Complex
TC0200013106.hg.1	4.2	5.47	-2.42	3.09E-09	5.26E-08	CCDC142; I	coiled-coil domain containing 142; mitochond	Multiple_Complex
TC0300012816.hg.1	7.2	5.39	3.5	3.12E-09	5.31E-08	P2RY12	purinergic receptor P2Y, G-protein coupled, 1.	Multiple_Complex
TC0200008047.hg.1	6.27	8.05	-3.43	3.20E-09	5.43E-08	ALMS1	Alstrom syndrome protein 1	Multiple_Complex
TC0700013370.hg.1	8.47	10.31	-3.59	3.20E-09	5.45E-08	ZNF107; M	zinc finger protein 107; microRNA 6839	Multiple_Complex
TC1300009332.hg.1	4.5	6.69	-4.55	3.22E-09	5.47E-08	RBM26	Transcript Identified by AceView, Entrez Gene	Unassigned
TC1600006738.hg.1	5.28	6.34	-2.09	3.28E-09	5.56E-08	DNAJA3	DnaJ (Hsp40) homolog, subfamily A, member	Multiple_Complex
TC0200010264.hg.1	8.11	10.03	-3.77	3.30E-09	5.59E-08	MFSD6	major facilitator superfamily domain containi	Multiple_Complex
TC0200014857.hg.1	5.27	6.75	-2.78	3.31E-09	5.61E-08	FASTKD1	FAST kinase domains 1	Multiple_Complex
TC0800008295.hg.1	8.27	9.36	-2.13	3.34E-09	5.65E-08	PTDSS1	phosphatidylserine synthase 1	Multiple_Complex
TC0300013847.hg.1	10.29	8.89	2.63	3.36E-09	5.68E-08	PPP4R2	protein phosphatase 4, regulatory subunit 2	Multiple_Complex
TC0100012670.hg.1	14.82	11.54	9.72	3.37E-09	5.70E-08	RPL22	ribosomal protein L22	Multiple_Complex
TC1700011305.hg.1	7.15	8.6	-2.73	3.41E-09	5.76E-08	HEATR6	HEAT repeat containing 6	Multiple_Complex
TC1200006645.hg.1	6.84	10.03	-9.13	3.42E-09	5.76E-08	CD4	CD4 molecule	Multiple_Complex
TC1600007528.hg.1	12.55	10.09	5.54	3.43E-09	5.77E-08	ITGAM	integrin, alpha M (complement component 3	Multiple_Complex
TC1900011071.hg.1	8.72	10.37	-3.13	3.43E-09	5.77E-08	KDELR1	KDEL (Lys-Asp-Glu-Leu) endoplasmic reticulun	Multiple_Complex
TC1900008699.hg.1	6.15	7.43	-2.43	3.47E-09	5.84E-08	ZNF528	zinc finger protein 528	Multiple_Complex
TC0900007576.hg.1	14.99	10.36	24.81	3.48E-09	5.85E-08	ANXA1	annexin A1	Multiple_Complex
TC2200008482.hg.1	6.75	8.23	-2.8	3.50E-09	5.89E-08	EIF4ENIF1	eukaryotic translation initiation factor 4E nucl	Multiple_Complex
TC0400011395.hg.1	9.1	7.41	3.22	3.50E-09	5.89E-08	EIF4E	eukaryotic translation initiation factor 4E	Multiple_Complex
TC1200007835.hg.1	6.1	7.58	-2.78	3.52E-09	5.91E-08	COQ10A	coenzyme Q10A	Multiple_Complex
TC0100017471.hg.1	13.61	11.84	3.41	3.53E-09	5.92E-08	WDR26; M	WD repeat domain 26; microRNA 4742	Multiple_Complex
TSUnmapped00000288.h	7.54	5.27	4.81	3.54E-09	5.93E-08	PADI4	peptidyl arginine deiminase, type IV	NonCoding
TC1100011131.hg.1	13.89	15.73	-3.6	3.55E-09	5.94E-08	C11orf95	chromosome 11 open reading frame 95	Multiple_Complex
TC1000009149.hg.1	6.9	9.06	-4.44	3.55E-09	5.94E-08	PLEKHA1	pleckstrin homology domain containing, famil	Multiple_Complex
TC2200009204.hg.1	10.37	11.46	-2.14	3.67E-09	6.13E-08	PPIL2	peptidylprolyl isomerase (cyclophilin)-like 2	Multiple_Complex
TC1000008661.hg.1	6.95	8.21	-2.4	3.69E-09	6.17E-08	SLF2	SMC5-SMC6 complex localization factor 2	Multiple_Complex
TC1800008891.hg.1	6.47	8.46	-3.96	3.70E-09	6.17E-08	BCL2	B-cell CLL/lymphoma 2	Multiple_Complex
TC1500008100.hg.1	9.57	8.38	2.29	3.73E-09	6.22E-08	WHAMM	WAS protein homolog associated with actin, g	Multiple_Complex
TC0200008823.hg.1	9.36	7.53	3.56	3.75E-09	6.25E-08	RGPD6; RG	RANBP2-like and GRIP domain containing 6; R	Multiple_Complex
TC0300014041.hg.1	5.98	7.22	-2.35	3.77E-09	6.27E-08	RUVBL1	RuvB-like AAA ATPase 1	Multiple_Complex
TC2200006614.hg.1	8.87	6.24	6.2	3.78E-09	6.28E-08	GP1BB; SEF	glycoprotein Ib (platelet), beta polypeptide; se	Multiple_Complex
TC1400009198.hg.1	7.42	8.99	-2.97	3.78E-09	6.29E-08	DDHD1	DDHD domain containing 1	Multiple_Complex
TC0100017078.hg.1	6.63	7.72	-2.12	3.80E-09	6.32E-08	RAB29	RAB29, member RAS oncogene family	Multiple_Complex
TC0900012229.hg.1	5.75	7.16	-2.66	3.81E-09	6.33E-08	EXOSC3	exosome component 3	Multiple_Complex
TC0800010663.hg.1	5.67	7.17	-2.84	3.82E-09	6.34E-08	ARMC1	armadillo repeat containing 1	Multiple_Complex
TC0900012160.hg.1	7.7	5.88	3.53	3.95E-09	6.55E-08	GNG10	guanine nucleotide binding protein (G protein	Coding
TC0X00006789.hg.1	7.26	8.29	-2.05	3.98E-09	6.59E-08	FAM3C2	FAM3C pseudogene	Multiple_Complex
TC0600006967.hg.1	6.87	5.65	2.34	3.98E-09	6.59E-08	EDN1	endothelin 1	Multiple_Complex
TC1000011585.hg.1	5.3	6.64	-2.52	4.03E-09	6.66E-08	GOT1	glutamic-oxaloacetic transaminase 1, soluble	Multiple_Complex
TC0900010789.hg.1	6.37	7.9	-2.88	4.03E-09	6.67E-08	IARS	isoleucyl-tRNA synthetase	Multiple_Complex
TC2200009357.hg.1	11.11	9.45	3.17	4.09E-09	6.75E-08	PACSIN2	protein kinase C and casein kinase substrate ir	Multiple_Complex
TC1200010265.hg.1	7.81	9.1	-2.45	4.13E-09	6.81E-08	CAPRIN2	caprin family member 2	Multiple_Complex
TC0700013338.hg.1	8.14	10.12	-3.95	4.16E-09	6.87E-08	GLCCI1	glucocorticoid induced 1	Multiple_Complex
TC1500009804.hg.1	11.2	12.48	-2.42	4.17E-09	6.87E-08	DENND4A	DENN/MADD domain containing 4A	Multiple_Complex
TC2000006561.hg.1	7.39	8.64	-2.38	4.18E-09	6.89E-08	MAVS	mitochondrial antiviral signaling protein	Multiple_Complex
TC1100011089.hg.1	9.78	10.94	-2.24	4.21E-09	6.92E-08	MTA2	metastasis associated 1 family member 2	Multiple_Complex
TC1800008285.hg.1	8.96	10.31	-2.55	4.24E-09	6.96E-08	NPC1	Niemann-Pick disease, type C1	Multiple_Complex
TC1100013096.hg.1	8.76	7.36	2.66	4.30E-09	7.06E-08	RBM7	RNA binding motif protein 7	Multiple_Complex
TC1200007652.hg.1	7.41	5.71	3.25	4.33E-09	7.10E-08	GRASP	GRP1 (general receptor for phosphoinositides	Multiple_Complex
TC1600007441.hg.1	11.51	10.37	2.2	4.37E-09	7.16E-08	PPP4C	protein phosphatase 4, catalytic subunit	Multiple_Complex
TC2200007467.hg.1	5.33	6.72	-2.63	4.37E-09	7.17E-08	ZC3H7B	zinc finger CCCH-type containing 7B	Multiple_Complex
TSUnmapped00000801.h	7.82	9.23	-2.66	4.38E-09	7.17E-08	VPS11	VPS11, CORVET/HOPS core subunit [Source:H	Coding
TC2000008200.hg.1	6.19	7.45	-2.38	4.39E-09	7.18E-08	PCED1A	PC-esterase domain containing 1A	Multiple_Complex
TC0200007746.hg.1	9.97	8.59	2.6	4.39E-09	7.18E-08	B3GNT2	UDP-GlcNAc:betaGal beta-1,3-N-acetylglucosa	Coding
TC1200006503.hg.1	6.43	7.85	-2.66	4.40E-09	7.19E-08	FKBP4	FK506 binding protein 4	Multiple_Complex
TC0300009131.hg.1	9.97	11.14	-2.26	4.42E-09	7.22E-08	HPS3	Hermansky-Pudlak syndrome 3	Multiple_Complex
TC1000012032.hg.1	12.69	7.72	31.46	4.43E-09	7.22E-08	RPL21	ribosomal protein L21	Multiple_Complex
TC0200010795.hg.1	13.33	10.48	7.23	4.47E-09	7.28E-08	SLC11A1	solute carrier family 11 (proton-coupled divale	Multiple_Complex
TC0200016719.hg.1	12.85	10.69	4.45	4.51E-09	7.34E-08	SNORD89;	small nucleolar RNA, C/D box 89; ring finger p	Multiple_Complex
TC1300006886.hg.1	5.42	6.98	-2.96	4.54E-09	7.38E-08	RFXAP	regulatory factor X-associated protein	Multiple_Complex
TC1700011319.hg.1	7.27	8.55	-2.44	4.60E-09	7.48E-08	APPBP2	amyloid beta precursor protein (cytoplasmic t	Multiple_Complex
TC1900009769.hg.1	7.77	6.06	3.28	4.61E-09	7.49E-08	STX10	syntaxin 10	Multiple_Complex
TC0900008574.hg.1	4.48	6.04	-2.94	4.69E-09	7.61E-08	TRIM32	tripartite motif containing 32	Coding
TC1900011402.hg.1	5.48	3.19	4.9	4.75E-09	7.71E-08	RPS9	Jeck2013 ANTISENSE, CDS, coding, INTERNAL,	NonCoding
TC1000008908.hg.1	12.35	11.02	2.53	4.79E-09	7.76E-08	SHOC2	SHOC2 leucine-rich repeat scaffold protein	Multiple_Complex
TC1700010571.hg.1	8.25	9.59	-2.53	4.80E-09	7.78E-08	MED1	mediator complex subunit 1	Multiple_Complex

TC0200008534.hg.1	8.55	10.32	-3.41	4.83E-09	7.82E-08	ANKRD36	ankyrin repeat domain 36	Multiple_Complex
TC0200010021.hg.1	17.73	15.94	3.45	4.85E-09	7.84E-08	H3F3AP4; I	Homo sapiens H3 histone, family 3A, pseudog	Multiple_Complex
TC1900011729.hg.1	7.47	8.81	-2.52	4.86E-09	7.85E-08	TIMM50	translocase of inner mitochondrial membrane	Multiple_Complex
TC0100018567.hg.1	9.04	7.34	3.24	4.88E-09	7.88E-08	RBM34	RNA binding motif protein 34	Multiple_Complex
TC0700012165.hg.1	6.22	8.12	-3.73	4.88E-09	7.88E-08	ATXN7L1	ataxin 7-like 1	Multiple_Complex
TC1700007700.hg.1	7.93	9.15	-2.34	4.91E-09	7.91E-08	MRPL45	mitochondrial ribosomal protein L45	Multiple_Complex
TC2000008722.hg.1	7.33	8.33	-2	4.97E-09	8.02E-08	ACSS1	acyl-CoA synthetase short-chain family memb	Multiple_Complex
TC1600006550.hg.1	4.85	6.3	-2.73	5.02E-09	8.08E-08	TMEM204	transmembrane protein 204	Coding
TC0100018476.hg.1	6.45	7.81	-2.56	5.03E-09	8.09E-08	PEX11B	peroxisomal biogenesis factor 11 beta	Multiple_Complex
TC0400009086.hg.1	7.5	5.18	4.98	5.12E-09	8.23E-08	GUCY1A3	guanylate cyclase 1, soluble, alpha 3	Multiple_Complex
TC0700008597.hg.1	11.1	9.44	3.16	5.20E-09	8.35E-08	CUX1	cut-like homeobox 1	Multiple_Complex
TC2200007791.hg.1	7.93	6.75	2.28	5.23E-09	8.40E-08	TRABD	Transcript Identified by AceView, Entrez Gene	Unassigned
TC1300008793.hg.1	10.36	7.85	5.67	5.24E-09	8.41E-08	TSC22D1	TSC22 domain family, member 1	Multiple_Complex
TC1500006870.hg.1	5.64	7.05	-2.67	5.25E-09	8.41E-08	C15orf41	chromosome 15 open reading frame 41	Multiple_Complex
TC0200016626.hg.1	11.8	9.3	5.68	5.29E-09	8.47E-08	MBOAT2	membrane bound O-acyltransferase domain c	Multiple_Complex
TC0300012924.hg.1	7.96	5.92	4.11	5.31E-09	8.49E-08	VEPH1	ventricular zone expressed PH domain contair	Multiple_Complex
TC0900010925.hg.1	4.53	5.82	-2.45	5.31E-09	8.49E-08	ZNF782	zinc finger protein 782	Multiple_Complex
TC1200008629.hg.1	9.64	7.84	3.49	5.36E-09	8.56E-08	DRAM1	DNA-damage regulated autophagy modulator	Multiple_Complex
TC1000011948.hg.1	11.96	9.9	4.18	5.37E-09	8.58E-08	PDZD8	PDZ domain containing 8	Multiple_Complex
TC0400011712.hg.1	9.42	6.45	7.82	5.38E-09	8.59E-08	PDE5A	phosphodiesterase 5A, cGMP-specific	Multiple_Complex
TC0500010894.hg.1	6.6	7.83	-2.35	5.43E-09	8.66E-08	DIMT1	DIM1 dimethyladenosine transferase 1 homol	Multiple_Complex
TC1200007826.hg.1	14.89	10.32	23.8	5.47E-09	8.71E-08	RPL41	ribosomal protein L41	Multiple_Complex
TC0600014374.hg.1	7.73	9.29	-2.95	5.51E-09	8.78E-08	PHF10	PHD finger protein 10	Multiple_Complex
TC1700007976.hg.1	6.89	8.39	-2.82	5.54E-09	8.82E-08	G6PC3	glucose 6 phosphatase, catalytic, 3	Multiple_Complex
TC0300007165.hg.1	11.41	13.16	-3.36	5.59E-09	8.89E-08	NKTR	natural killer cell triggering receptor	Multiple_Complex
TC0100015577.hg.1	6.41	7.95	-2.91	5.61E-09	8.91E-08	PPIAL4F; PI	peptidylprolyl isomerase A (cyclophilin A)-like	Coding
TC2100007336.hg.1	10.63	11.88	-2.38	5.63E-09	8.94E-08	TRAPPC10	trafficking protein particle complex 10	Multiple_Complex
TC1900007917.hg.1	7.01	8.33	-2.49	5.77E-09	9.15E-08	ZNF146	zinc finger protein 146	Multiple_Complex
TC2000006916.hg.1	12.26	7.88	20.79	5.87E-09	9.31E-08	RPL41P1	ribosomal protein L41 pseudogene 1	Multiple_Complex
TC1600008166.hg.1	7.34	8.77	-2.69	5.92E-09	9.37E-08	CTCF	CCCTC-binding factor (zinc finger protein)	Multiple_Complex
TC1200011936.hg.1	7.66	9.53	-3.65	5.95E-09	9.42E-08	ATXN2	ataxin 2	Multiple_Complex
TC1900010863.hg.1	4.95	6.21	-2.41	5.97E-09	9.44E-08	SMG9	SMG9 nonsense mediated mRNA decay factor	Multiple_Complex
TC2000007514.hg.1	15.15	10	35.56	6.05E-09	9.56E-08	MMP9	matrix metallopeptidase 9	Multiple_Complex
TC1400009538.hg.1	9.57	10.94	-2.58	6.07E-09	9.59E-08	DCAF5	DDB1 and CUL4 associated factor 5	Multiple_Complex
TC0100011105.hg.1	18.21	19.27	-2.09	6.08E-09	9.59E-08	PTPRC	protein tyrosine phosphatase, receptor type, (	Multiple_Complex
TC0700012701.hg.1	7.55	9.09	-2.92	6.11E-09	9.64E-08	CNOT4	CCR4-NOT transcription complex subunit 4	Multiple_Complex
TC1400006691.hg.1	9.65	11.43	-3.44	6.24E-09	9.83E-08	THTPA	thiamine triphosphatase	Multiple_Complex
TC0100015715.hg.1	10.55	12.11	-2.95	6.25E-09	9.84E-08	SF3B4	splicing factor 3b, subunit 4, 49kDa	Multiple_Complex
TC0500008705.hg.1	6.39	7.95	-2.95	6.30E-09	9.91E-08	TXNDC15	thioredoxin domain containing 15	Multiple_Complex
TC1500009623.hg.1	10.73	8.9	3.55	6.34E-09	9.96E-08	BNIP2	BCL2/adenovirus E1B 19kDa interacting prote	Multiple_Complex
TC1500008023.hg.1	12.82	10.7	4.34	6.40E-09	1.00E-07	ZFAND6	zinc finger, AN1-type domain 6	Multiple_Complex
TC0500013355.hg.1	6.28	4.88	2.63	6.48E-09	1.02E-07	P4HA2	prolyl 4-hydroxylase, alpha polypeptide II	NonCoding
TC0400008213.hg.1	4.77	6.07	-2.46	6.50E-09	1.02E-07	RAP1GDS1	RAP1, GTP-GDP dissociation stimulator 1	Multiple_Complex
TC2100008384.hg.1	7.52	8.72	-2.3	6.54E-09	1.03E-07	UBE2G2	ubiquitin conjugating enzyme E2G 2	Multiple_Complex
TC1300006989.hg.1	7.05	8.88	-3.56	6.55E-09	1.03E-07	AKAP11	A kinase (PRKA) anchor protein 11	Multiple_Complex
TC0200014990.hg.1	12.31	9.83	5.55	6.56E-09	1.03E-07	CIR1	corepressor interacting with RBPJ, 1	Multiple_Complex
TC0200016474.hg.1	11.7	14.12	-5.37	6.57E-09	1.03E-07	NAGK	N-acetylglucosamine kinase	Multiple_Complex
TC0400011283.hg.1	7.72	9.37	-3.12	6.60E-09	1.03E-07	PPM1K	protein phosphatase, Mg2+/Mn2+ dependent	Multiple_Complex
TC0400012634.hg.1	7.73	9.51	-3.45	6.62E-09	1.03E-07	CFAP97	cilia and flagella associated protein 97	Multiple_Complex
TC0800007976.hg.1	9.53	8.62	1.88	6.67E-09	1.04E-07	TMEM70; F	transmembrane protein 70; ribosomal protein	Multiple_Complex
TC0100013924.hg.1	9.33	10.37	-2.05	6.71E-09	1.05E-07	ELOVL1; M	ELOVL fatty acid elongase 1; microRNA 6734	Multiple_Complex
TC1400009016.hg.1	8.03	6.52	2.85	6.74E-09	1.05E-07	FBXO33	F-box protein 33	Multiple_Complex
TC0100018437.hg.1	9.87	8.46	2.67	6.82E-09	1.06E-07	MKNK1	MAP kinase interacting serine/threonine kinas	Multiple_Complex
TC0900011453.hg.1	5.18	6.59	-2.66	6.83E-09	1.06E-07	ZBTB6	zinc finger and BTB domain containing 6	Coding
TC0700009677.hg.1	8.8	11.34	-5.79	6.93E-09	1.08E-07	GIMAP4	GTPase, IMAP family member 4	Multiple_Complex
TC1200009131.hg.1	8.48	10.23	-3.35	6.96E-09	1.08E-07	DYNLL1	dynein, light chain, LC8-type 1	Multiple_Complex
TC0900006526.hg.1	6.12	7.26	-2.2	7.02E-09	1.09E-07	PLPP6	phospholipid phosphatase 6	Coding
TC1100013144.hg.1	6.5	7.73	-2.35	7.08E-09	1.10E-07	ZBED5	zinc finger, BED-type containing 5	Multiple_Complex
TC1900007456.hg.1	8.74	9.81	-2.1	7.12E-09	1.10E-07	ZNF253	zinc finger protein 253	Multiple_Complex
TC1200009461.hg.1	7.06	9.3	-4.71	7.15E-09	1.11E-07	EP400; SNC	E1A binding protein p400; small nucleolar RN/	Multiple_Complex
TC1600008454.hg.1	9.91	8.23	3.21	7.15E-09	1.11E-07	GABARAPL:	GABA(A) receptor-associated protein like 2	Multiple_Complex
TC1200012760.hg.1	5.19	6.82	-3.09	7.19E-09	1.11E-07	PRR4	proline rich 4 (lacrimal)	Multiple_Complex
TSUnmapped00000194.h	6.24	7.16	-1.89	7.20E-09	1.11E-07	INPP5D	inositol polyphosphate-5-phosphatase D	NonCoding
TC2100008561.hg.1	10.19	7.07	8.71	7.22E-09	1.12E-07	ATP5O	ATP synthase, H+ transporting, mitochondrial	Multiple_Complex
TC0X00008514.hg.1	6.67	8.23	-2.95	7.27E-09	1.12E-07	DDX26B	DEAD/H (Asp-Glu-Ala-Asp/His) box polypeptic	Multiple_Complex
TC1200009846.hg.1	6.59	8.84	-4.76	7.28E-09	1.12E-07	LINC00612	long intergenic non-protein coding RNA 612	Multiple_Complex
TC1800007680.hg.1	10.45	11.72	-2.4	7.31E-09	1.13E-07	CNDP2	CNDP dipeptidase 2 (metallopeptidase M20 fa	Multiple_Complex
TC0X00010097.hg.1	9.48	8.03	2.74	7.34E-09	1.13E-07	RLIM	ring finger protein, LIM domain interacting	Multiple_Complex
TC1600008865.hg.1	7.05	8.01	-1.95	7.35E-09	1.13E-07	ACSF3	acyl-CoA synthetase family member 3	Multiple_Complex
TC0300009357.hg.1	8.08	5.93	4.44	7.37E-09	1.13E-07	NMD3	NMD3 ribosome export adaptor	Multiple_Complex

TC0600013603.hg.1	8.03	6.09	3.82	7.40E-09	1.14E-07	MTRF1L	mitochondrial translational release factor 1-lik	Multiple_Complex
TC0400011643.hg.1	8.84	10.25	-2.66	7.49E-09	1.15E-07	CAMK2D	calcium/calmodulin-dependent protein kinase	Multiple_Complex
TC0600014102.hg.1	10.21	11.8	-3.01	7.49E-09	1.15E-07	HCP5	HLA complex P5 (non-protein coding)	Multiple_Complex
TC0400010230.hg.1	15.85	14.64	2.32	7.60E-09	1.17E-07	GBA3	Jeck2013 ANTISENSE, coding, INTERNAL, intro	Multiple_Complex
TC0100018465.hg.1	8.92	7.47	2.73	7.69E-09	1.18E-07	BCAS2	breast carcinoma amplified sequence 2	Multiple_Complex
TC0200010448.hg.1	10.28	14.9	-24.53	7.93E-09	1.21E-07	STRADB	STE20-related kinase adaptor beta	Multiple_Complex
TC0900007489.hg.1	6.97	8.29	-2.51	7.97E-09	1.22E-07	FAM122A	family with sequence similarity 122A	Coding
TC0700009292.hg.1	14.04	10.06	15.79	8.10E-09	1.24E-07	RPL41	Synthetic construct Homo sapiens clone IMAG	Coding
TC1900011795.hg.1	10.96	12.48	-2.88	8.11E-09	1.24E-07	NDUFA3	NADH dehydrogenase (ubiquinone) 1 alpha su	Multiple_Complex
TC1900009128.hg.1	7.56	8.96	-2.64	8.14E-09	1.24E-07	TMEM259	transmembrane protein 259	Multiple_Complex
TC0300012208.hg.1	9.16	10.97	-3.52	8.17E-09	1.25E-07	HACD2	3-hydroxyacyl-CoA dehydratase 2	Multiple_Complex
TC0500010019.hg.1	5.03	6.33	-2.46	8.24E-09	1.26E-07	FASTKD3	FAST kinase domains 3	Multiple_Complex
TC0300013954.hg.1	5.28	4.04	2.36	8.28E-09	1.26E-07	ZNF385D	zinc finger protein 385D	Multiple_Complex
TC1000008651.hg.1	7.79	8.94	-2.21	8.39E-09	1.28E-07	HIF1AN	hypoxia inducible factor 1, alpha subunit inhib	Multiple_Complex
TSUnmapped00000463.h	6.92	5.36	2.95	8.40E-09	1.28E-07	RPS25	ribosomal protein S25	NonCoding
TC1100009880.hg.1	8.84	10.56	-3.28	8.42E-09	1.28E-07	TRIM21	tripartite motif containing 21	Coding
TC0X00011305.hg.1	7.86	10.35	-5.63	8.44E-09	1.28E-07	NHSL2	NHS-like 2	Multiple_Complex
TC1600010951.hg.1	5.88	7.49	-3.03	8.45E-09	1.28E-07	MAF	v-maf avian musculoaponeurotic fibrosarcoma	Coding
TC1000011549.hg.1	6.66	5.51	2.22	8.71E-09	1.32E-07	RRP12	Memczak2013 ALT_ACCEPTOR, ALT_DONOR,	NonCoding
TC0200016360.hg.1	6.39	7.77	-2.61	8.72E-09	1.32E-07	STK25	serine/threonine kinase 25	Multiple_Complex
TC1200010516.hg.1	12.04	14.47	-5.39	8.76E-09	1.33E-07	SLC38A1	solute carrier family 38, member 1	Multiple_Complex
TC0600012883.hg.1	6.52	8.01	-2.81	9.03E-09	1.37E-07	TUBE1	tubulin, epsilon 1	Multiple_Complex
TC1800006710.hg.1	11.8	10.38	2.67	9.09E-09	1.38E-07	CHMP1B	charged multivesicular body protein 1B	Multiple_Complex
TC0X00008080.hg.1	6.44	8.55	-4.33	9.09E-09	1.38E-07	VSIG1	V-set and immunoglobulin domain containing	Multiple_Complex
TC1100007139.hg.1	6.38	7.9	-2.87	9.23E-09	1.39E-07	METTL15	methyltransferase like 15	Multiple_Complex
TC0800012211.hg.1	5.7	7.06	-2.56	9.70E-09	1.47E-07	CPSF1; MIR	cleavage and polyadenylation specific factor 1	Multiple_Complex
TC1700009681.hg.1	8.47	9.86	-2.62	9.76E-09	1.47E-07	CTC1	CTS telomere maintenance complex compone	Multiple_Complex
TC0200009189.hg.1	9.38	13.77	-20.93	9.85E-09	1.49E-07	GYPC	glycophorin C (Gerbich blood group)	Multiple_Complex
TC0X00010895.hg.1	5.9	7.05	-2.22	9.94E-09	1.50E-07	ZNF75D	zinc finger protein 75D	Multiple_Complex
TC0700009056.hg.1	7	6.11	1.86	9.98E-09	1.50E-07	FAM71F2	family with sequence similarity 71, member F	Multiple_Complex
TC0100010185.hg.1	7.41	8.61	-2.3	1.00E-08	1.51E-07	APOA1BP	apolipoprotein A-I binding protein	Multiple_Complex
TC1000009880.hg.1	6.9	8.72	-3.53	1.00E-08	1.51E-07	NMT2	N-myristoyltransferase 2	Multiple_Complex
TC0100015596.hg.1	6.77	8.25	-2.78	1.01E-08	1.51E-07	LIX1L	limb and CNS expressed 1 like	Multiple_Complex
TC1200007630.hg.1	6.36	7.49	-2.19	1.01E-08	1.51E-07	LETMD1	LETM1 domain containing 1	Multiple_Complex
TC0600010331.hg.1	4.63	6.07	-2.72	1.03E-08	1.55E-07	FAM120B	family with sequence similarity 120B	Multiple_Complex
TC1700012431.hg.1	4.11	5.1	-2	1.04E-08	1.55E-07	HOXB2	homeobox B2	Multiple_Complex
TC0100014650.hg.1	7.04	8.55	-2.83	1.04E-08	1.56E-07	ZZZ3	zinc finger, ZZ-type containing 3	Multiple_Complex
TC0300012007.hg.1	3.94	5.82	-3.67	1.04E-08	1.56E-07	CD200R1	CD200 receptor 1	Coding
TC1900007963.hg.1	6.56	8.34	-3.44	1.04E-08	1.56E-07	ZNF383	zinc finger protein 383	Multiple_Complex
TC0500010932.hg.1	9.98	8.51	2.78	1.05E-08	1.56E-07	SGTB	small glutamine-rich tetratricopeptide repeat	Multiple_Complex
TC0900011378.hg.1	8.94	7.01	3.8	1.07E-08	1.59E-07	CDK5RAP2	CDK5 regulatory subunit associated protein 2	Multiple_Complex
TC1200010927.hg.1	13.6	14.46	-1.82	1.07E-08	1.60E-07	ATP5B	ATP synthase, H+ transporting, mitochondrial	Multiple_Complex
TC0200012256.hg.1	7.91	9.24	-2.52	1.07E-08	1.60E-07	HEATR5B	HEAT repeat containing 5B	Multiple_Complex
TC0400006793.hg.1	6.04	4.27	3.41	1.08E-08	1.61E-07	USP17L15	ubiquitin specific peptidase 17-like family mer	Coding
TC0600013531.hg.1	5.94	7.29	-2.56	1.09E-08	1.62E-07	NUP43	nucleoporin 43kDa	Multiple_Complex
TC0300006968.hg.1	9.53	11.02	-2.83	1.09E-08	1.62E-07	CMTM7	CKLF-like MARVEL transmembrane domain co	Multiple_Complex
TC0800006983.hg.1	8.88	10.27	-2.64	1.10E-08	1.64E-07	PPP3CC	protein phosphatase 3, catalytic subunit, gam	Multiple_Complex
TC0X00007148.hg.1	10.79	12.2	-2.65	1.12E-08	1.66E-07	ARAF	A-Raf proto-oncogene, serine/threonine kinas	Multiple_Complex
TC1400009123.hg.1	10.97	8.95	4.05	1.12E-08	1.67E-07	SOS2	SOS Ras/Rho guanine nucleotide exchange fac	Multiple_Complex
TC0100010075.hg.1	7.56	8.86	-2.46	1.12E-08	1.67E-07	ATP8B2	ATPase, aminophospholipid transporter, class	Multiple_Complex
TC0500009076.hg.1	5.26	6.46	-2.3	1.13E-08	1.68E-07	SLC26A2	solute carrier family 26 (anion exchanger), me	Multiple_Complex
TC0900012024.hg.1	5.09	7.11	-4.04	1.16E-08	1.71E-07	CLIC3	chloride intracellular channel 3	Multiple_Complex
TC1200008678.hg.1	7.15	5.43	3.29	1.16E-08	1.72E-07	EID3	EP300 interacting inhibitor of differentiation 3	Coding
TC2000009469.hg.1	5.38	6.68	-2.46	1.17E-08	1.73E-07	ZFP64	ZFP64 zinc finger protein	Multiple_Complex
TC2100007597.hg.1	8.39	7.13	2.39	1.18E-08	1.75E-07	HSPA13	heat shock protein 70kDa family, member 13	Multiple_Complex
TC1100007003.hg.1	13.38	11.54	3.57	1.20E-08	1.78E-07	LDHA	lactate dehydrogenase A	Multiple_Complex
TC0300011158.hg.1	11.46	12.5	-2.06	1.21E-08	1.79E-07	WDR82	WD repeat domain 82	Multiple_Complex
TC0600009710.hg.1	12.32	10.82	2.82	1.22E-08	1.81E-07	STX11	syntaxin 11	Coding
TC0500009061.hg.1	5.48	6.51	-2.05	1.23E-08	1.82E-07	PCYOX1L	prenylcysteine oxidase 1 like	Multiple_Complex
TC0200008655.hg.1	11.55	10.14	2.66	1.27E-08	1.87E-07	MAP4K4	mitogen-activated protein kinase kinase kinas	Multiple_Complex
TC1600006926.hg.1	8.7	7.34	2.57	1.29E-08	1.90E-07	SNN	stannin	Coding
TC1900010951.hg.1	6.69	5.36	2.51	1.32E-08	1.95E-07	SNRPD2	small nuclear ribonucleoprotein D2 polypeptic	Multiple_Complex
TC1200009735.hg.1	8.53	5.59	7.69	1.33E-08	1.95E-07	MRPL51	mitochondrial ribosomal protein L51	Multiple_Complex
TC0800008726.hg.1	7.56	8.92	-2.57	1.33E-08	1.95E-07	TBC1D31	TBC1 domain family, member 31	Multiple_Complex
TC0200015608.hg.1	5.47	7.02	-2.93	1.33E-08	1.96E-07	LANCL1	LanC lantibiotic synthetase component C-like	Multiple_Complex
TC0X00007529.hg.1	9.33	7.37	3.91	1.34E-08	1.96E-07	IGBP1	immunoglobulin (CD79A) binding protein 1	Multiple_Complex
TC1900011817.hg.1	4.85	6.57	-3.29	1.34E-08	1.97E-07	ZNF773	zinc finger protein 773	Multiple_Complex
TC0500009478.hg.1	6.3	4.87	2.7	1.34E-08	1.97E-07	RPL26L1	ribosomal protein L26-like 1	Coding
TC1500007645.hg.1	4.7	6.62	-3.79	1.37E-08	2.01E-07	MAP2K5	mitogen-activated protein kinase kinase 5	Multiple_Complex
TC1100013062.hg.1	4.93	6.28	-2.55	1.39E-08	2.03E-07	NADSYN1	NAD synthetase 1	Multiple_Complex

TC0200008452.hg.1	6.51	8.99	-5.58	1.39E-08	2.04E-07	MAL	mal, T-cell differentiation protein	Multiple_Complex
TC1200010633.hg.1	7.19	8.51	-2.5	1.40E-08	2.04E-07	MCRS1	microspherule protein 1	Multiple_Complex
TC0600007792.hg.1	6.37	7.76	-2.62	1.40E-08	2.05E-07	PPARD	peroxisome proliferator-activated receptor de	Multiple_Complex
TC0200007785.hg.1	9.26	8.17	2.13	1.41E-08	2.05E-07	UGP2	UDP-glucose pyrophosphorylase 2	Multiple_Complex
TC1000007176.hg.1	11.41	9.79	3.07	1.42E-08	2.07E-07	MAP3K8	mitogen-activated protein kinase kinase kinas	Multiple_Complex
TC0200011080.hg.1	7	8.74	-3.35	1.42E-08	2.08E-07	COPS7B	COP9 signalosome subunit 7B	Multiple_Complex
TC1900006488.hg.1	11.17	12.73	-2.96	1.44E-08	2.10E-07	PTBP1; MIF	polypyrimidine tract binding protein 1; microF	Multiple_Complex
TC0600012413.hg.1	9.11	10.44	-2.51	1.44E-08	2.10E-07	IBTK	inhibitor of Bruton agammaglobulinemia tyros	Multiple_Complex
TC0100007406.hg.1	8.54	10.44	-3.73	1.45E-08	2.11E-07	LDLRAP1	low density lipoprotein receptor adaptor prot	Multiple_Complex
TC0700013397.hg.1	9.44	7.72	3.29	1.46E-08	2.13E-07	CDK14	cyclin-dependent kinase 14	Multiple_Complex
TC0200015226.hg.1	5.56	7.03	-2.77	1.48E-08	2.15E-07	HIBCH	Transcript Identified by AceView, Entrez Gene	Unassigned
TC0X00008829.hg.1	16.92	18.79	-3.65	1.48E-08	2.15E-07	RPL10; SNC	ribosomal protein L10; small nucleolar RNA, H	Multiple_Complex
TC0700010510.hg.1	6.74	8.19	-2.73	1.48E-08	2.15E-07	C7orf31	chromosome 7 open reading frame 31	Multiple_Complex
TC0100016917.hg.1	6.86	8.24	-2.6	1.49E-08	2.16E-07	CSRP1	cysteine and glycine-rich protein 1	Multiple_Complex
TC0600010864.hg.1	10.59	9.43	2.24	1.50E-08	2.17E-07	RANBP9	RAN binding protein 9	Multiple_Complex
TC0800007435.hg.1	7.17	8.28	-2.17	1.54E-08	2.23E-07	IKBKB	inhibitor of kappa light polypeptide gene enha	Multiple_Complex
TC1500008521.hg.1	13.08	11.93	2.23	1.57E-08	2.27E-07	MEF2A	myocyte enhancer factor 2A	Multiple_Complex
TC0300009306.hg.1	8.87	6.94	3.81	1.57E-08	2.27E-07	RSRC1	arginine/serine-rich coiled-coil 1	Multiple_Complex
TC2200009180.hg.1	6.95	8.21	-2.4	1.59E-08	2.30E-07	RABL2B	RAB, member of RAS oncogene family-like 2B	Multiple_Complex
TC0200015246.hg.1	8.61	10.69	-4.21	1.59E-08	2.30E-07	STAT4	signal transducer and activator of transcriptio	Multiple_Complex
TC0500013271.hg.1	8.41	9.81	-2.63	1.60E-08	2.31E-07	LOC728554	THO complex 3 pseudogene; Homo sapiens TH	Multiple_Complex
TC0100016137.hg.1	7.75	9.46	-3.27	1.60E-08	2.31E-07	CD84	CD84 molecule	Multiple_Complex
TC0100007268.hg.1	16.39	15.21	2.27	1.61E-08	2.33E-07	CDC42	cell division cycle 42	Multiple_Complex
TC2200009328.hg.1	6.99	8.16	-2.25	1.61E-08	2.33E-07	HPS4	Hermansky-Pudlak syndrome 4	Multiple_Complex
TC1700012185.hg.1	3.93	5.53	-3.03	1.63E-08	2.34E-07	TNFSF12	tumor necrosis factor (ligand) superfamily, me	Multiple_Complex
TC1100011535.hg.1	3.55	4.72	-2.24	1.67E-08	2.41E-07	LOC100133	transient receptor potential cation channel, su	Multiple_Complex
TC1300009583.hg.1	5.69	6.94	-2.39	1.68E-08	2.41E-07	DOCK9	dedicator of cytokinesis 9	Multiple_Complex
TC1600010734.hg.1	6.15	7.54	-2.61	1.68E-08	2.41E-07	NOB1	NIN1/RPN12 binding protein 1 homolog	Multiple_Complex
TC1100008008.hg.1	5.53	7.05	-2.87	1.68E-08	2.41E-07	POLA2	polymerase (DNA directed), alpha 2, accessory	Multiple_Complex
TC0300009459.hg.1	10.6	8.88	3.29	1.68E-08	2.41E-07	GPR160	G protein-coupled receptor 160	Multiple_Complex
TC0600012040.hg.1	8.33	9.36	-2.04	1.69E-08	2.43E-07	TRAM2	translocation associated membrane protein 2	Multiple_Complex
TC1700006717.hg.1	8.67	6.73	3.86	1.70E-08	2.44E-07	ALOX12	arachidonate 12-lipoxygenase	Multiple_Complex
TC0X00010836.hg.1	11.96	10.29	3.19	1.71E-08	2.46E-07	RAP2C	RAP2C, member of RAS oncogene family	Multiple_Complex
TSUnmapped00000319.h	5.44	4.52	1.89	1.72E-08	2.46E-07	NDUFA6	NADH dehydrogenase (ubiquinone) 1 alpha su	Coding
TC0800006969.hg.1	7.44	11.08	-12.45	1.72E-08	2.46E-07	DMTN	dematin actin binding protein	Multiple_Complex
TC0X00011221.hg.1	9.51	10.83	-2.5	1.73E-08	2.48E-07	GAB3	GRB2-associated binding protein 3	Multiple_Complex
TC1300008388.hg.1	9.37	7.18	4.57	1.78E-08	2.55E-07	MTMR6	Transcript Identified by AceView, Entrez Gene	Unassigned
TC0700010453.hg.1	8.78	6.45	5.01	1.79E-08	2.56E-07	TOMM7	translocase of outer mitochondrial membrane	Multiple_Complex
TC1700007042.hg.1	9.85	11.35	-2.82	1.82E-08	2.59E-07	MPRIP	myosin phosphatase Rho interacting protein	Multiple_Complex
TC0400007609.hg.1	7.95	9.29	-2.54	1.82E-08	2.60E-07	REST	RE1-silencing transcription factor	Multiple_Complex
TC1900011854.hg.1	8.04	9.46	-2.68	1.82E-08	2.60E-07	ELAVL1	ELAV like RNA binding protein 1	Multiple_Complex
TC0800011285.hg.1	8.99	10.21	-2.32	1.83E-08	2.60E-07	ZNF706	zinc finger protein 706	Multiple_Complex
TC0200007458.hg.1	7.48	6.54	1.92	1.83E-08	2.60E-07	EPAS1	endothelial PAS domain protein 1	Multiple_Complex
TC2200007894.hg.1	9.98	12.81	-7.11	1.83E-08	2.61E-07	CECR1	cat eye syndrome chromosome region, candic	Multiple_Complex
TC1200006454.hg.1	6.4	8.25	-3.58	1.85E-08	2.63E-07	WNK1	Transcript Identified by AceView, Entrez Gene	Unassigned
TC0400012222.hg.1	5.8	4.21	3.01	1.86E-08	2.64E-07	PDGFC	platelet derived growth factor C	Multiple_Complex
TC0600012038.hg.1	5.04	6.27	-2.34	1.88E-08	2.67E-07	MCM3	minichromosome maintenance complex comp	Multiple_Complex
TC1000007020.hg.1	9.33	7.98	2.55	1.89E-08	2.69E-07	OTUD1	OTU deubiquitinase 1	Coding
TC1400010596.hg.1	10.83	8.4	5.39	1.92E-08	2.72E-07	PSMA6	proteasome subunit alpha 6	Multiple_Complex
TC0900010580.hg.1	7.21	5.41	3.47	1.92E-08	2.72E-07	GKAP1	G kinase anchoring protein 1	Multiple_Complex
TC0700006562.hg.1	6.8	8.25	-2.74	1.92E-08	2.72E-07	LFNG; MIR4	LFNG O-fucosylpeptide 3-beta-N-acetylglucosa	Multiple_Complex
TC1600011527.hg.1	10.96	12.41	-2.74	1.93E-08	2.73E-07	VKORC1	vitamin K epoxide reductase complex subunit	Multiple_Complex
TC0700009647.hg.1	5.43	6.58	-2.22	1.93E-08	2.73E-07	ZNF862	zinc finger protein 862	Multiple_Complex
TC1900008121.hg.1	9.2	10.44	-2.37	1.93E-08	2.73E-07	SNRPA	small nuclear ribonucleoprotein polypeptide A	Multiple_Complex
TC0100016476.hg.1	10.69	13.12	-5.41	1.94E-08	2.74E-07	KIAA0040	KIAA0040	Multiple_Complex
TC1700008232.hg.1	6.99	8.87	-3.69	1.94E-08	2.74E-07	PDK2	pyruvate dehydrogenase kinase, isozyme 2	Multiple_Complex
TC0100017500.hg.1	8.09	9.52	-2.7	1.95E-08	2.75E-07	TMEM63A	transmembrane protein 63A	Multiple_Complex
TC0X00006676.hg.1	7.94	5.11	7.09	1.95E-08	2.75E-07	BMX	BMX non-receptor tyrosine kinase	Multiple_Complex
TC1800006739.hg.1	9.45	10.6	-2.22	1.96E-08	2.75E-07	PSMG2	proteasome (prosome, macropain) assembly c	Multiple_Complex
TC1700008757.hg.1	9.07	7.3	3.42	1.96E-08	2.75E-07	MAP2K6	mitogen-activated protein kinase kinase 6	Multiple_Complex
TC1200010977.hg.1	6.36	7.78	-2.67	1.96E-08	2.76E-07	CDK4	cyclin-dependent kinase 4	Multiple_Complex
TC0100009344.hg.1	5.78	7.02	-2.37	1.97E-08	2.77E-07	SARS	seryl-tRNA synthetase	Multiple_Complex
TC0600010017.hg.1	8.26	9.79	-2.9	1.98E-08	2.78E-07	TMEM181	transmembrane protein 181	Multiple_Complex
TC0400008134.hg.1	8.4	5.79	6.13	1.98E-08	2.78E-07	MMRN1	multimerin 1	Multiple_Complex
TC1500010907.hg.1	5.25	6.44	-2.28	1.98E-08	2.78E-07	MESDC2	mesoderm development candidate 2	Multiple_Complex
TC0400012621.hg.1	16.06	12.9	8.95	1.99E-08	2.79E-07	ACSL1	acyl-CoA synthetase long-chain family membe	Multiple_Complex
TC1000010498.hg.1	5.82	7.65	-3.54	2.00E-08	2.80E-07	8-Mar	Transcript Identified by AceView, Entrez Gene	Unassigned
TC0100014257.hg.1	8.98	10.13	-2.22	2.01E-08	2.81E-07	SSBP3	single stranded DNA binding protein 3	Multiple_Complex
TC1200007931.hg.1	6.92	7.99	-2.1	2.01E-08	2.81E-07	SLC16A7	solute carrier family 16 (monocarboxylate trar	Multiple_Complex
TC0600006873.hg.1	7.95	5.95	4	2.03E-08	2.83E-07	BMP6	bone morphogenetic protein 6	Multiple_Complex

TC0500013373.hg.1	8	7.1	1.86	2.03E-08	2.84E-07	SRA1	steroid receptor RNA activator 1	Multiple_Complex
TC0100008653.hg.1	5.01	6.41	-2.63	2.03E-08	2.84E-07	IL12RB2	interleukin 12 receptor, beta 2	Multiple_Complex
TC1500007633.hg.1	7.54	8.99	-2.72	2.03E-08	2.84E-07	SMAD3	SMAD family member 3	Multiple_Complex
TC0900011819.hg.1	7.92	9.24	-2.5	2.04E-08	2.85E-07	TSC1	tuberous sclerosis 1	Multiple_Complex
TC0100010674.hg.1	6.87	8.43	-2.94	2.08E-08	2.90E-07	GPR52	G protein-coupled receptor 52	Coding
TC0600012514.hg.1	10.95	9.26	3.21	2.09E-08	2.91E-07	AKIRIN2	akirin 2	Multiple_Complex
TC0700009488.hg.1	8.49	10.07	-2.99	2.11E-08	2.93E-07	CASP2	caspase 2	Multiple_Complex
TC1800007863.hg.1	5.16	6.17	-2.02	2.14E-08	2.97E-07	ENOSF1	enolase superfamily member 1	Multiple_Complex
TC0600012566.hg.1	8.35	9.37	-2.03	2.14E-08	2.98E-07	MAP3K7	mitogen-activated protein kinase kinase kinas	Multiple_Complex
TC1500009952.hg.1	12.46	10.31	4.43	2.16E-08	3.01E-07	PKM	pyruvate kinase, muscle	Multiple_Complex
TC1800008978.hg.1	4.44	6.47	-4.08	2.19E-08	3.04E-07	RTTN	rotatin	Multiple_Complex
TC0200009964.hg.1	10.97	7.82	8.9	2.20E-08	3.05E-07	HAT1	histone acetyltransferase 1	Multiple_Complex
TC0600014241.hg.1	10.55	8.62	3.83	2.20E-08	3.05E-07	TBC1D7	TBC1 domain family, member 7	Multiple_Complex
TC1500008751.hg.1	7.61	9.16	-2.91	2.22E-08	3.08E-07	UBE3A	ubiquitin protein ligase E3A	Multiple_Complex
TC0200012734.hg.1	9.72	10.88	-2.24	2.24E-08	3.10E-07	USP34	ubiquitin specific peptidase 34	Multiple_Complex
TC1000008431.hg.1	10.46	12.7	-4.71	2.25E-08	3.11E-07	PCGF5	polycomb group ring finger 5	Multiple_Complex
TC0100018247.hg.1	8.6	9.7	-2.14	2.27E-08	3.14E-07	LRRC8D	leucine rich repeat containing 8 family, memb	Multiple_Complex
TC0200016530.hg.1	10.03	8.77	2.41	2.28E-08	3.15E-07	PLEKHB2	pleckstrin homology domain containing, famil	Multiple_Complex
TC1700011476.hg.1	15	13.77	2.36	2.29E-08	3.16E-07	GNA13	guanine nucleotide binding protein (G protein	Multiple_Complex
TC1200012861.hg.1	7.75	6.69	2.09	2.29E-08	3.16E-07	DIABLO	diablo, IAP-binding mitochondrial protein	Multiple_Complex
TC1000008795.hg.1	11.86	10.41	2.74	2.31E-08	3.18E-07	SLK	STE20-like kinase	Multiple_Complex
TC0300010329.hg.1	6.51	8.24	-3.3	2.31E-08	3.18E-07	NUP210	nucleoporin 210kDa	Multiple_Complex
TC0600014360.hg.1	10.68	11.66	-1.98	2.32E-08	3.19E-07	IPCEF1	interaction protein for cytohesin exchange fac	Multiple_Complex
TC2000008310.hg.1	10.59	8.8	3.48	2.33E-08	3.20E-07	TRMT6	tRNA methyltransferase 6	Multiple_Complex
TC1000007103.hg.1	11.95	10.92	2.04	2.35E-08	3.23E-07	RAB18	RAB18, member RAS oncogene family	Multiple_Complex
TC0500012455.hg.1	6.46	8.63	-4.5	2.36E-08	3.25E-07	CSF1R	colony stimulating factor 1 receptor	Multiple_Complex
TC0200013603.hg.1	8.23	6.46	3.41	2.37E-08	3.26E-07	MITD1	microtubule interacting and trafficking domai	Multiple_Complex
TC0100008347.hg.1	5.24	7.08	-3.6	2.38E-08	3.27E-07	CPT2	carnitine palmitoyltransferase 2	Multiple_Complex
TSUnmapped00000630.h	4.65	6.24	-3.02	2.38E-08	3.27E-07	ZNF780A	zinc finger protein 780A	Coding
TC0200016679.hg.1	8.73	7.28	2.74	2.40E-08	3.29E-07	SERTAD2	SERTA domain containing 2	Multiple_Complex
TC0600007847.hg.1	8.9	6.45	5.48	2.50E-08	3.43E-07	CDKN1A	cyclin-dependent kinase inhibitor 1A (p21, Cip	Multiple_Complex
TC1000012490.hg.1	8.51	7.12	2.63	2.50E-08	3.43E-07	PI4K2A	phosphatidylinositol 4-kinase type 2 alpha	Multiple_Complex
TC0100006840.hg.1	11.12	12.12	-2	2.55E-08	3.49E-07	TARDBP	TAR DNA binding protein	Multiple_Complex
TC0200016703.hg.1	12.6	10.98	3.09	2.55E-08	3.49E-07	CHMP3	charged multivesicular body protein 3	Multiple_Complex
TC0900007494.hg.1	6.02	7.39	-2.58	2.56E-08	3.50E-07	FXN	frataxin	Multiple_Complex
TC1500007912.hg.1	5.63	7	-2.6	2.57E-08	3.51E-07	FBXO22	F-box protein 22	Multiple_Complex
TC0200010733.hg.1	7.45	9.14	-3.23	2.57E-08	3.51E-07	XRCC5	Memczak2013 ALT_ACCEPTOR, ALT_DONOR,	NonCoding
TC0400012874.hg.1	4.59	6.45	-3.63	2.57E-08	3.51E-07	CYP4V2	cytochrome P450, family 4, subfamily V, polyp	Multiple_Complex
TC0200008005.hg.1	11.37	8.16	9.28	2.57E-08	3.51E-07	DYSF	dysferlin	Multiple_Complex
TC2100006967.hg.1	16.14	14.63	2.85	2.58E-08	3.51E-07	IFNGR2	interferon gamma receptor 2 (interferon gamı	Multiple_Complex
TC0100015885.hg.1	10.12	11.32	-2.29	2.58E-08	3.51E-07	GATAD2B	GATA zinc finger domain containing 2B	Multiple_Complex
TC0200012062.hg.1	5.62	6.77	-2.22	2.60E-08	3.54E-07	PREB	prolactin regulatory element binding	Multiple_Complex
TC1700009686.hg.1	4.73	6.03	-2.47	2.60E-08	3.54E-07	SLC25A35	solute carrier family 25, member 35	Multiple_Complex
TC1000011485.hg.1	10.58	8.02	5.9	2.62E-08	3.56E-07	PDLIM1	PDZ and LIM domain 1	Multiple_Complex
TC1100011030.hg.1	7.92	9.05	-2.18	2.64E-08	3.59E-07	DDB1	damage-specific DNA binding protein 1	Multiple_Complex
TC1900011721.hg.1	5.35	6.78	-2.69	2.69E-08	3.65E-07	HKR1	HKR1, GLI-Kruppel zinc finger family member	NonCoding
TC1900009371.hg.1	6.29	7.72	-2.7	2.70E-08	3.66E-07	ARRDC5	arrestin domain containing 5	Coding
TC1800007471.hg.1	6.32	5.04	2.43	2.73E-08	3.70E-07	PMAIP1	phorbol-12-myristate-13-acetate-induced pro	Multiple_Complex
TC0800011002.hg.1	5.94	4.27	3.19	2.79E-08	3.78E-07	REXO1L10F	REX1, RNA exonuclease 1 homolog-like 10, ps	Multiple_Complex
TC0200007803.hg.1	10.54	7.88	6.3	2.81E-08	3.81E-07	LGALSL	lectin, galactoside-binding-like	Multiple_Complex
TC0100015984.hg.1	7.35	8.54	-2.28	2.85E-08	3.85E-07	UBQLN4	ubiquilin 4	Multiple_Complex
TC0500009521.hg.1	11.7	9.59	4.31	2.86E-08	3.87E-07	CPEB4	cytoplasmic polyadenylation element binding	Multiple_Complex
TC0100007370.hg.1	5.15	7.48	-5.04	2.91E-08	3.94E-07	RCAN3	RCAN family member 3	Multiple_Complex
TC1700008175.hg.1	12	10.81	2.29	2.92E-08	3.94E-07	CALCOCO2	calcium binding and coiled-coil domain 2	Multiple_Complex
TC1700007399.hg.1	4.79	6.19	-2.64	2.94E-08	3.96E-07	ERAL1	Era-like 12S mitochondrial rRNA chaperone 1	Multiple_Complex
TC0600014273.hg.1	11.9	14.32	-5.33	2.94E-08	3.96E-07	HLA-DRB1	major histocompatibility complex, class II, DR	Multiple_Complex
TC2000007219.hg.1	8.07	9.51	-2.72	2.96E-08	3.99E-07	ERGIC3	ERGIC and golgi 3	Multiple_Complex
TC0100010623.hg.1	8.23	6.4	3.55	2.97E-08	4.00E-07	SUCO	SUN domain containing ossification factor	Multiple_Complex
TC0300011048.hg.1	10.09	11.32	-2.34	2.98E-08	4.01E-07	QRICH1	glutamine-rich 1	Multiple_Complex
TC0500007434.hg.1	14.78	10.31	22.13	3.00E-08	4.04E-07	RPL41	Synthetic construct Homo sapiens clone IMAG	Multiple_Complex
TC0800009094.hg.1	13.14	11.3	3.58	3.01E-08	4.05E-07	DENND3	DENN/MADD domain containing 3	Multiple_Complex
TC0200016646.hg.1	9.69	8.24	2.74	3.04E-08	4.09E-07	MEMO1	mediator of cell motility 1	Multiple_Complex
TC0900007116.hg.1	5.39	4.14	2.37	3.06E-08	4.11E-07	CCIN	calicin	Coding
TC1000008276.hg.1	10.88	12.31	-2.69	3.07E-08	4.12E-07	CCSER2	coiled-coil serine rich protein 2	Multiple_Complex
TC1400009791.hg.1	4.95	6.08	-2.18	3.07E-08	4.12E-07	ALKBH1	alkB homolog 1, histone H2A dioxygenase	Multiple_Complex
TC1600007169.hg.1	13.16	11.65	2.85	3.09E-08	4.15E-07	METTL9	methyltransferase like 9	Multiple_Complex
TC1900008214.hg.1	8.87	5.2	12.74	3.11E-08	4.17E-07	CD177	CD177 molecule	Coding
TC0900008742.hg.1	7.23	5.88	2.55	3.11E-08	4.17E-07	ARPC5L	actin related protein 2/3 complex subunit 5-lil	Multiple_Complex
TC1700012109.hg.1	6.81	8.17	-2.58	3.12E-08	4.17E-07	CD7	CD7 molecule	Multiple_Complex
TC1700010436.hg.1	4.99	6.21	-2.32	3.12E-08	4.17E-07	PEX12	peroxisomal biogenesis factor 12	Coding

TC1500010787.hg.1	11.72	9.94	3.43	3.12E-08	4.17E-07	LINC01578	long intergenic non-protein coding RNA 1578	NonCoding
TC1500010660.hg.1	6.66	5.45	2.31	3.13E-08	4.18E-07	PCSK6	proprotein convertase subtilisin/kexin type 6	Multiple_Complex
TC0300011054.hg.1	4.4	5.56	-2.23	3.15E-08	4.20E-07	CCDC71	coiled-coil domain containing 71	Coding
TC1000010071.hg.1	6.97	4.9	4.18	3.20E-08	4.26E-07	ENKUR	enkurin, TRPC channel interacting protein	Multiple_Complex
TC0100018515.hg.1	5.7	4.45	2.37	3.21E-08	4.28E-07	CFAP45	cilia and flagella associated protein 45	Multiple_Complex
TC1600009381.hg.1	5.96	7.03	-2.1	3.24E-08	4.31E-07	DEXI	Dexi homolog (mouse)	Multiple_Complex
TC0100014250.hg.1	12.54	10.95	3.02	3.25E-08	4.32E-07	TMEM59	transmembrane protein 59	Multiple_Complex
TC0400010463.hg.1	12.14	7.93	18.54	3.25E-08	4.33E-07	RPL9	ribosomal protein L9	Multiple_Complex
TC1100012961.hg.1	5.8	4.95	1.8	3.28E-08	4.36E-07	AMPD3	adenosine monophosphate deaminase 3	Multiple_Complex
TC1600007511.hg.1	6.59	7.75	-2.25	3.33E-08	4.42E-07	ZNF646	zinc finger protein 646	Coding
TC0200015976.hg.1	14.15	16.21	-4.17	3.35E-08	4.45E-07	SP110	SP110 nuclear body protein	Multiple_Complex
TC0700011493.hg.1	8.52	9.62	-2.15	3.38E-08	4.48E-07	BAZ1B	bromodomain adjacent to zinc finger domain	Multiple_Complex
TC1200012798.hg.1	11.11	12.92	-3.5	3.38E-08	4.48E-07	ATP5G2	ATP synthase, H+ transporting, mitochondrial	Multiple_Complex
TC1700010916.hg.1	8.12	10.08	-3.88	3.39E-08	4.50E-07	ARL17B; AF	ADP-ribosylation factor like GTPase 17B; ADP-	Multiple_Complex
TC0500008653.hg.1	6.54	5.24	2.46	3.40E-08	4.50E-07	UQCRQ	ubiquinol-cytochrome c reductase complex III	Multiple_Complex
TC0800007330.hg.1	5.94	7.06	-2.17	3.43E-08	4.54E-07	DDHD2	DDHD domain containing 2	Multiple_Complex
TC0400008359.hg.1	14.57	8.22	81.91	3.43E-08	4.54E-07	RPL34	ribosomal protein L34	Multiple_Complex
TC0100016024.hg.1	7.73	8.99	-2.39	3.44E-08	4.55E-07	SH2D2A	SH2 domain containing 2A	Multiple_Complex
TC0300008385.hg.1	5.38	6.45	-2.09	3.44E-08	4.55E-07	TIGIT	T-cell immunoreceptor with Ig and ITIM doma	Multiple_Complex
TC0500013384.hg.1	6.91	8.67	-3.38	3.46E-08	4.57E-07	PPP2R2B	protein phosphatase 2, regulatory subunit B, b	Multiple_Complex
TC0200008807.hg.1	9.39	7.86	2.89	3.49E-08	4.61E-07	RANBP2	RAN binding protein 2	Multiple_Complex
TC1400010777.hg.1	10.53	12.5	-3.93	3.55E-08	4.68E-07	TC2N	tandem C2 domains, nuclear	Multiple_Complex
TC0200013030.hg.1	6.27	4.51	3.4	3.61E-08	4.76E-07	DYSF; kydo	Memczak2013 ANTISENSE, coding, INTERNAL,	NonCoding
TC1800007504.hg.1	7.9	9.15	-2.38	3.62E-08	4.77E-07	KIAA1468	KIAA1468	Multiple_Complex
TC1400008945.hg.1	7.45	9.47	-4.07	3.62E-08	4.77E-07	RALGAPA1	Ral GTPase activating protein, alpha subunit 1	Multiple_Complex
TC1600007557.hg.1	12.16	10.09	4.2	3.66E-08	4.82E-07	ZNF267	zinc finger protein 267	Multiple_Complex
TC0400010251.hg.1	8.51	9.85	-2.53	3.67E-08	4.83E-07	DHX15	DEAH (Asp-Glu-Ala-His) box helicase 15	Multiple_Complex
TC0600007207.hg.1	5.49	4.2	2.45	3.68E-08	4.84E-07	GMNN	geminin, DNA replication inhibitor	Multiple_Complex
TC2200008734.hg.1	7.77	8.87	-2.15	3.69E-08	4.84E-07	CBX7	chromobox homolog 7	Multiple_Complex
TC0700008430.hg.1	7.81	6.08	3.3	3.69E-08	4.85E-07	SDHAF3	succinate dehydrogenase complex assembly fa	Multiple_Complex
TC0500009321.hg.1	9.18	10.44	-2.39	3.70E-08	4.85E-07	MAT2B	methionine adenosyltransferase II, beta	Multiple_Complex
TC0600007608.hg.1	8.85	6.1	6.72	3.70E-08	4.86E-07	C6orf25	chromosome 6 open reading frame 25	Multiple_Complex
TC1400007639.hg.1	7.17	8.46	-2.44	3.73E-08	4.88E-07	C14orf169	chromosome 14 open reading frame 169	Multiple_Complex
TC0700008765.hg.1	8.65	6.74	3.76	3.74E-08	4.90E-07	DLD	dihydrolipoamide dehydrogenase	Multiple_Complex
TC1900007957.hg.1	5.3	6.38	-2.11	3.76E-08	4.92E-07	ZNF568	zinc finger protein 568	Multiple_Complex
TC1200012799.hg.1	7.68	6.16	2.86	3.82E-08	4.99E-07	SARNP	SAP domain containing ribonucleoprotein	Multiple_Complex
TC0400010418.hg.1	12	10.04	3.88	3.84E-08	5.02E-07	RELL1	RELT-like 1	Multiple_Complex
TC0600007597.hg.1	12.82	11.04	3.45	3.85E-08	5.02E-07	LST1	leukocyte specific transcript 1	Multiple_Complex
TC1200012754.hg.1	5.2	7.31	-4.33	3.91E-08	5.10E-07	KLRC1	killer cell lectin-like receptor subfamily C, mem	Multiple_Complex
TC0600007306.hg.1	9.75	11.71	-3.89	3.91E-08	5.10E-07	BTN3A3	butyrophilin, subfamily 3, member A3	Multiple_Complex
TC0900008827.hg.1	6.61	7.75	-2.21	3.94E-08	5.13E-07	FPGS	folylpolyglutamate synthase	Multiple_Complex
TC0900012184.hg.1	5.95	7.07	-2.17	3.95E-08	5.14E-07	PRRC2B	proline-rich coiled-coil 2B	Multiple_Complex
TC0700008081.hg.1	8.56	9.95	-2.62	3.97E-08	5.17E-07	MDH2	malate dehydrogenase 2	Multiple_Complex
TC1200008568.hg.1	9.09	10.47	-2.6	3.98E-08	5.18E-07	TMPO	thymopoietin	Multiple_Complex
TC1600009236.hg.1	5.07	6.69	-3.07	4.05E-08	5.26E-07	UBALD1	UBA-like domain containing 1	Multiple_Complex
TC0600013203.hg.1	10.56	7.33	9.43	4.05E-08	5.26E-07	VNN1	vanin 1	Coding
TC0800008370.hg.1	8.32	6.53	3.46	4.06E-08	5.27E-07	POLR2K	polymerase (RNA) II (DNA directed) polypeptic	Multiple_Complex
TC0100012376.hg.1	7.16	8.5	-2.54	4.06E-08	5.28E-07	ZNF672	zinc finger protein 672	Multiple_Complex
TC2000010010.hg.1	7.1	8.06	-1.95	4.08E-08	5.30E-07	BLCAP	bladder cancer associated protein	Multiple_Complex
TC0200015425.hg.1	8.01	9.47	-2.75	4.10E-08	5.31E-07	TRAK2	trafficking protein, kinesin binding 2	Multiple_Complex
TC0400011014.hg.1	8.9	6.91	3.98	4.15E-08	5.37E-07	CXCL5	chemokine (C-X-C motif) ligand 5	Multiple_Complex
TC2000007507.hg.1	6.69	7.78	-2.13	4.15E-08	5.37E-07	ZSWIM1	zinc finger, SWIM-type containing 1	Multiple_Complex
TC1600008505.hg.1	7.72	8.61	-1.85	4.15E-08	5.37E-07	WWOX	WW domain containing oxidoreductase	Multiple_Complex
TC0700012461.hg.1	8.03	9.49	-2.75	4.22E-08	5.47E-07	WASL	Wiskott-Aldrich syndrome-like	Multiple_Complex
TC1900008533.hg.1	14.94	16.76	-3.54	4.25E-08	5.49E-07	CD37	CD37 molecule	Multiple_Complex
TC1800008824.hg.1	9.7	11.18	-2.79	4.26E-08	5.51E-07	LMAN1	lectin, mannose-binding, 1	Multiple_Complex
TC0300008142.hg.1	10.51	9.07	2.71	4.28E-08	5.53E-07	TBC1D23	TBC1 domain family, member 23	Multiple_Complex
TC1200011845.hg.1	14.84	16.47	-3.11	4.28E-08	5.53E-07	SELPLG	selectin P ligand	Coding
TC1200011385.hg.1	9.28	6.86	5.36	4.37E-08	5.64E-07	LIN7A	lin-7 homolog A (C. elegans)	Multiple_Complex
TC0400008380.hg.1	9.73	11.79	-4.18	4.43E-08	5.71E-07	CCDC109B	coiled-coil domain containing 109B	Multiple_Complex
TC1900009628.hg.1	8.97	7.56	2.67	4.48E-08	5.77E-07	CDC37; MII	cell division cycle 37; microRNA 1181	Multiple_Complex
TC0200013079.hg.1	9.63	10.69	-2.09	4.49E-08	5.78E-07	DUSP11	dual specificity phosphatase 11	Multiple_Complex
TC0700013544.hg.1	8.05	5.78	4.8	4.57E-08	5.88E-07	PSMA2	proteasome subunit alpha 2	Multiple_Complex
TC0300013896.hg.1	12.96	11.18	3.45	4.60E-08	5.91E-07	MFSD1	major facilitator superfamily domain containir	Multiple_Complex
TC0300006784.hg.1	14.47	12.89	2.99	4.63E-08	5.95E-07	RAB5A	RAB5A, member RAS oncogene family	Multiple_Complex
TC2000009204.hg.1	6.32	7.4	-2.11	4.70E-08	6.04E-07	TOMM34	translocase of outer mitochondrial membrane	Multiple_Complex
TC0100011533.hg.1	5.9	4.6	2.45	4.70E-08	6.04E-07	ATF3	activating transcription factor 3	Multiple_Complex
TC0100009444.hg.1	13.81	12.45	2.57	4.73E-08	6.06E-07	CAPZA1	capping protein (actin filament) muscle Z-line,	Multiple_Complex
TC0400006804.hg.1	6.62	5.08	2.91	4.73E-08	6.06E-07	USP17L22;	ubiquitin specific peptidase 17-like family mer	Coding
TC0900011254.hg.1	7.82	6.71	2.16	4.73E-08	6.06E-07	CDC26	cell division cycle 26	Multiple_Complex

TC1100012444.hg.1	6.85	8	-2.22	4.73E-08	6.06E-07	PCSK7	proprotein convertase subtilisin/kexin type 7	Multiple_Complex
TC1900010460.hg.1	5.4	6.56	-2.23	4.74E-08	6.07E-07	ZNF792	zinc finger protein 792	Multiple_Complex
TC0800007321.hg.1	9.1	7.98	2.18	4.74E-08	6.07E-07	ASH2L	ash2 (absent, small, or homeotic)-like (Drosop	Multiple_Complex
TC1300008735.hg.1	8.5	9.8	-2.46	4.75E-08	6.07E-07	VWA8; MIF	von Willebrand factor A domain containing 8;	Multiple_Complex
TC0200016705.hg.1	10.53	8.89	3.12	4.78E-08	6.11E-07	RNF103-CH	RNF103-CHMP3 readthrough; charged multive	Coding
TC0500009488.hg.1	11.95	10.61	2.52	4.89E-08	6.24E-07	CREBRF	CREB3 regulatory factor	Multiple_Complex
TC1200007899.hg.1	4.88	6.39	-2.84	4.92E-08	6.28E-07	9-Mar	membrane associated ring finger 9	Multiple_Complex
TC1900010886.hg.1	5.48	6.62	-2.2	4.95E-08	6.32E-07	ZNF235	zinc finger protein 235	Multiple_Complex
TC0Y00006444.hg.1	12.38	10.58	3.48	4.99E-08	6.36E-07	CSF2RA	colony stimulating factor 2 receptor, alpha, lo	Multiple_Complex
TC1500007939.hg.1	6	7.2	-2.3	5.03E-08	6.40E-07	HMG20A	high mobility group 20A	Multiple_Complex
TC2000007251.hg.1	12.53	9.2	10.07	5.04E-08	6.42E-07	MYL9	myosin light chain 9	Multiple_Complex
TC1100008012.hg.1	9.53	10.73	-2.3	5.07E-08	6.45E-07	DPF2	D4, zinc and double PHD fingers family 2	Multiple_Complex
TC1000011043.hg.1	5.08	4.17	1.87	5.07E-08	6.45E-07	C10orf55	chromosome 10 open reading frame 55	Multiple_Complex
TC0100018498.hg.1	9.61	11.47	-3.63	5.08E-08	6.45E-07	KRTCAP2	keratinocyte associated protein 2	Multiple_Complex
TC1000006880.hg.1	6.66	8.2	-2.92	5.08E-08	6.46E-07	PTER	phosphotriesterase related	Multiple_Complex
TC1100006810.hg.1	7.8	8.87	-2.11	5.12E-08	6.50E-07	IPO7; SNOF	importin 7; small nucleolar RNA, H/ACA box 2	Multiple_Complex
TC0X00008794.hg.1	7.22	11.79	-23.66	5.14E-08	6.52E-07	SLC6A8	solute carrier family 6 (neurotransmitter trans	Multiple_Complex
TC0600014139.hg.1	6.34	7.42	-2.12	5.16E-08	6.54E-07	PRIM2	primase, DNA, polypeptide 2 (58kDa)	Multiple_Complex
TC0300011801.hg.1	6.58	7.91	-2.5	5.20E-08	6.58E-07	MINA	MYC induced nuclear antigen	Multiple_Complex
TC2000008815.hg.1	7.56	11.01	-10.94	5.25E-08	6.65E-07	BCL2L1	BCL2-like 1	Multiple_Complex
TC1000009908.hg.1	15.8	14.11	3.22	5.25E-08	6.65E-07	RSU1	Ras suppressor protein 1	Multiple_Complex
TC1900008103.hg.1	7.54	9.28	-3.34	5.26E-08	6.65E-07	PLD3	phospholipase D family, member 3	Multiple_Complex
TC0200008500.hg.1	6.21	7.51	-2.46	5.29E-08	6.68E-07	CIAO1	cytosolic iron-sulfur assembly component 1	Multiple_Complex
TC0700013067.hg.1	7.84	6.52	2.49	5.29E-08	6.69E-07	SMARCD3	SWI/SNF related, matrix associated, actin dep	Multiple_Complex
TC0100017229.hg.1	5.12	6.8	-3.22	5.38E-08	6.79E-07	INTS7	integrator complex subunit 7	Multiple_Complex
TC1800006889.hg.1	13.31	10.46	7.24	5.41E-08	6.83E-07	RIOK3	RIO kinase 3	Multiple_Complex
TC0400012381.hg.1	7.5	8.68	-2.26	5.45E-08	6.87E-07	NEK1	NIMA-related kinase 1	Multiple_Complex
TC1800006506.hg.1	15.8	14.07	3.32	5.53E-08	6.97E-07	MYL12A	myosin light chain 12A	Multiple_Complex
TC1600009077.hg.1	13.03	15.06	-4.11	5.53E-08	6.97E-07	RPS2; SNOI	ribosomal protein S2; small nucleolar RNA, H/	Multiple_Complex
TC2000010025.hg.1	12.54	13.98	-2.72	5.56E-08	6.99E-07	UBE2V1	ubiquitin conjugating enzyme E2 variant 1	Multiple_Complex
TC0800010154.hg.1	7.42	6	2.68	5.58E-08	7.02E-07	LSM1	LSM1 homolog, mRNA degradation associatec	Multiple_Complex
TC1100010757.hg.1	9.01	10.46	-2.73	5.60E-08	7.04E-07	MTCH2	mitochondrial carrier 2	Multiple_Complex
TC1100012775.hg.1	11.73	13.07	-2.53	5.60E-08	7.04E-07	ETS1	v-ets avian erythroblastosis virus E26 oncoger	Multiple_Complex
TC0500013277.hg.1	7.37	8.75	-2.6	5.61E-08	7.05E-07	MAML1	mastermind-like transcriptional coactivator 1	Multiple_Complex
TC1400007298.hg.1	10.08	8.42	3.17	5.63E-08	7.06E-07	ACTR10	actin-related protein 10 homolog (S. cerevisia	Multiple_Complex
TC2000007925.hg.1	13.53	10.47	8.32	5.64E-08	7.07E-07	TUBB1	tubulin, beta 1 class VI	Coding
TC1400010749.hg.1	6.48	7.83	-2.55	5.66E-08	7.09E-07	LINC01599	long intergenic non-protein coding RNA 1599	Multiple_Complex
TC0600011465.hg.1	5.97	7.39	-2.67	5.67E-08	7.11E-07	EHMT2	euchromatic histone-lysine N-methyltransfera	Multiple_Complex
TC0200011974.hg.1	11.24	8.27	7.82	5.71E-08	7.16E-07	SF3B6	splicing factor 3b subunit 6	Multiple_Complex
TC0X00010648.hg.1	12.55	9.36	9.13	5.77E-08	7.22E-07	RPL39; SNC	ribosomal protein L39; small nucleolar RNA, H	Multiple_Complex
TC0600009236.hg.1	8.14	6.28	3.63	5.77E-08	7.22E-07	RWDD1	RWD domain containing 1	Multiple_Complex
TC1400006656.hg.1	9.42	10.94	-2.86	5.80E-08	7.25E-07	OXA1L	oxidase (cytochrome c) assembly 1-like	Multiple_Complex
TC0600011372.hg.1	8.41	9.77	-2.56	5.81E-08	7.26E-07	PPP1R10	protein phosphatase 1, regulatory subunit 10	Multiple_Complex
TC0200014790.hg.1	4.73	3.52	2.31	5.84E-08	7.29E-07	GRB14	growth factor receptor bound protein 14	Multiple_Complex
TC0300006840.hg.1	9.39	8.19	2.3	5.84E-08	7.29E-07	UBE2E1	ubiquitin conjugating enzyme E2E 1	Multiple_Complex
TC1200009722.hg.1	12.29	14.47	-4.55	5.86E-08	7.31E-07	TNFRSF1A	tumor necrosis factor receptor superfamily, m	Multiple_Complex
TC0800011887.hg.1	8.43	9.58	-2.22	5.89E-08	7.34E-07	ST3GAL1	ST3 beta-galactoside alpha-2,3-sialyltransfera	Multiple_Complex
TC1900011837.hg.1	9.53	8.16	2.57	5.92E-08	7.38E-07	UQCR11	ubiquinol-cytochrome c reductase, complex II	Multiple_Complex
TC0500013430.hg.1	15.67	17.19	-2.86	5.94E-08	7.40E-07	GNB2L1; SI	guanine nucleotide binding protein (G protein	Multiple_Complex
TC0100015817.hg.1	6.67	8.17	-2.84	5.96E-08	7.41E-07	THEM4	thioesterase superfamily member 4	Multiple_Complex
TSUnmapped00000753.h	12.9	8.89	16.09	6.09E-08	7.58E-07	RPS25	ribosomal protein S25	Coding
TC1700011114.hg.1	7	7.9	-1.87	6.11E-08	7.59E-07	ANKRD40	ankyrin repeat domain 40	Multiple_Complex
TC2000008295.hg.1	11.34	9.54	3.46	6.12E-08	7.60E-07	GPCPD1	glycerophosphocholine phosphodiesterase 1	Multiple_Complex
TC2200006881.hg.1	6.18	7.67	-2.8	6.15E-08	7.63E-07	CABIN1	calcineurin binding protein 1	Multiple_Complex
TC1100011187.hg.1	6.59	7.75	-2.24	6.16E-08	7.65E-07	MEN1	multiple endocrine neoplasia I	Multiple_Complex
TC0600014044.hg.1	9.03	7.71	2.5	6.19E-08	7.67E-07	PSMB1	proteasome subunit beta 1	Multiple_Complex
TC0200016421.hg.1	11.8	12.95	-2.22	6.21E-08	7.69E-07	BRE	brain and reproductive organ-expressed (TNFI	Multiple_Complex
TC0300009313.hg.1	7.16	7.99	-1.77	6.22E-08	7.70E-07	GFM1	G elongation factor, mitochondrial 1	Multiple_Complex
TC1900012025.hg.1	5.84	7.41	-2.97	6.25E-08	7.73E-07	ZNF28	zinc finger protein 28	Multiple_Complex
TC0200014167.hg.1	7.59	8.76	-2.25	6.32E-08	7.82E-07	ERCC3	excision repair cross-complementation group	Multiple_Complex
TC1900010073.hg.1	11.31	9.77	2.9	6.37E-08	7.87E-07	LPAR2	lysophosphatidic acid receptor 2	Multiple_Complex
TC1600010028.hg.1	6.08	7.25	-2.26	6.38E-08	7.88E-07	C16orf58	chromosome 16 open reading frame 58	Multiple_Complex
TC0300011378.hg.1	9.86	8.39	2.78	6.46E-08	7.97E-07	THOC7	THO complex 7	Multiple_Complex
TC1700009417.hg.1	7	8.38	-2.6	6.46E-08	7.97E-07	SMG6	SMG6 nonsense mediated mRNA decay factor	Multiple_Complex
TC0900011404.hg.1	11.13	9.44	3.22	6.48E-08	7.99E-07	STOM	stomatin	Multiple_Complex
TC0200012971.hg.1	8.37	6.51	3.64	6.49E-08	8.00E-07	ANXA4	Salzman2013 ANTISENSE, coding, INTERNAL, i	NonCoding
TC0900011597.hg.1	8.39	9.64	-2.38	6.52E-08	8.03E-07	SH2D3C	SH2 domain containing 3C	Multiple_Complex
TC0300014052.hg.1	7.72	9.13	-2.66	6.57E-08	8.09E-07	RYK	receptor-like tyrosine kinase	Multiple_Complex
TC1900008146.hg.1	7.73	8.71	-1.98	6.60E-08	8.12E-07	CCDC97	coiled-coil domain containing 97	Coding
TC2000008095.hg.1	10.69	9.46	2.33	6.61E-08	8.13E-07	TPD52L2	tumor protein D52-like 2	Multiple_Complex

TC1100007688.hg.1	8.13	9.24	-2.15	6.62E-08	8.14E-07	ZDHHC5	zinc finger, DHHC-type containing 5	Multiple_Complex
TC2200008710.hg.1	8.02	6.86	2.23	6.69E-08	8.22E-07	JOSD1	Josephin domain containing 1	Multiple_Complex
TC1900011650.hg.1	10.32	11.62	-2.47	6.70E-08	8.22E-07	ARHGEF18	Rho/Rac guanine nucleotide exchange factor :	Multiple_Complex
TC0200010790.hg.1	5.52	6.71	-2.28	6.76E-08	8.29E-07	GPBAR1	G protein-coupled bile acid receptor 1	Coding
TC0100013072.hg.1	5.37	6.98	-3.07	6.79E-08	8.33E-07	ATP13A2	ATPase type 13A2	Multiple_Complex
TC1700012304.hg.1	7.25	8.55	-2.46	6.85E-08	8.40E-07	COG1	component of oligomeric golgi complex 1	Multiple_Complex
TC0100016833.hg.1	5.59	7.29	-3.24	6.87E-08	8.41E-07	ZBTB41	zinc finger and BTB domain containing 41	Multiple_Complex
TC1600008928.hg.1	7.91	11.19	-9.74	6.89E-08	8.44E-07	NPRL3	NPR3-like, GATOR1 complex subunit	Multiple_Complex
TC1500010422.hg.1	8.13	9.3	-2.25	6.90E-08	8.44E-07	IDH2	isocitrate dehydrogenase 2 (NADP+), mitocho	Multiple_Complex
TC1900011767.hg.1	7.57	8.57	-2	7.04E-08	8.60E-07	BLOC1S3	biogenesis of lysosomal organelles complex-1,	Multiple_Complex
TC0400008443.hg.1	9.16	6.31	7.19	7.12E-08	8.70E-07	LARP7	La ribonucleoprotein domain family, member	Multiple_Complex
TC1700012120.hg.1	6.85	8.14	-2.45	7.15E-08	8.73E-07	OGFOD3	2-oxoglutarate and iron-dependent oxygenase	Multiple_Complex
TC0700011519.hg.1	8.52	9.72	-2.3	7.15E-08	8.73E-07	STAG3L2	stromal antigen 3-like 2 (pseudogene)	Multiple_Complex
TC0100012222.hg.1	7.66	8.93	-2.4	7.25E-08	8.85E-07	DESI2	desumoylating isopeptidase 2	Multiple_Complex
TC0900009912.hg.1	6.6	7.68	-2.12	7.35E-08	8.96E-07	SIGMAR1	sigma non-opioid intracellular receptor 1	Multiple_Complex
TC0600013147.hg.1	8.44	6.85	3.01	7.45E-08	9.07E-07	ARHGAP18	Rho GTPase activating protein 18	Multiple_Complex
TC0X00009007.hg.1	6.54	8.23	-3.23	7.45E-08	9.07E-07	PUDP	pseudouridine 5-phosphatase	Multiple_Complex
TC0200015402.hg.1	12.98	10.92	4.17	7.53E-08	9.17E-07	FAM126B	family with sequence similarity 126, member	Multiple_Complex
TC1400010767.hg.1	7.81	8.64	-1.77	7.56E-08	9.19E-07	ZFYVE26	zinc finger, FYVE domain containing 26	Multiple_Complex
TC0900009770.hg.1	9.01	7.76	2.37	7.61E-08	9.25E-07	PLAA	phospholipase A2-activating protein	Multiple_Complex
TC1800009277.hg.1	7.79	9	-2.32	7.64E-08	9.28E-07	PIAS2	protein inhibitor of activated STAT 2	Multiple_Complex
TC1200007713.hg.1	12.26	13.73	-2.78	7.68E-08	9.33E-07	SP1	Sp1 transcription factor	Multiple_Complex
TC1200007418.hg.1	13.26	11.27	3.96	7.70E-08	9.35E-07	ANO6	anoctamin 6	Multiple_Complex
TC1200010407.hg.1	4.3	6.05	-3.38	7.73E-08	9.38E-07	KIF21A	kinesin family member 21A	Multiple_Complex
TC1700007930.hg.1	10.21	7.2	8.02	7.76E-08	9.41E-07	RPL27	ribosomal protein L27	Multiple_Complex
TC0200016026.hg.1	5.06	6.33	-2.42	7.79E-08	9.44E-07	NMUR1	neuromedin U receptor 1	Coding
TC1900011775.hg.1	5.23	6.55	-2.5	7.81E-08	9.46E-07	GRWD1	glutamate-rich WD repeat containing 1	Multiple_Complex
TC2000007386.hg.1	5.96	7.3	-2.53	7.86E-08	9.51E-07	PLCG1; RPL	phospholipase C, gamma 1; ribosomal protein	Multiple_Complex
TC0100010268.hg.1	7.72	9.08	-2.57	7.91E-08	9.57E-07	FCRL6	Fc receptor-like 6	Multiple_Complex
TC0800008845.hg.1	7.73	9.48	-3.37	7.94E-08	9.60E-07	MYC	v-myc avian myelocytomatosis viral oncogene	Multiple_Complex
TC0100012278.hg.1	7.42	5.91	2.84	7.99E-08	9.65E-07	SCCPDH	saccharopine dehydrogenase (putative)	Multiple_Complex
TC1900007110.hg.1	7.83	9.61	-3.44	8.02E-08	9.68E-07	RAD23A	RAD23 homolog A, nucleotide excision repair	Multiple_Complex
TC0700011147.hg.1	10.82	9.06	3.38	8.02E-08	9.68E-07	VOPP1	Jeck2013 ALT_ACCEPTOR, ALT_DONOR, codin	NonCoding
TC0300013103.hg.1	7.83	6.04	3.45	8.06E-08	9.73E-07	RPL22L1	ribosomal protein L22-like 1	Multiple_Complex
TC0200010374.hg.1	4.87	5.8	-1.9	8.12E-08	9.79E-07	MARS2	methionyl-tRNA synthetase 2, mitochondrial	Coding
TC0800007440.hg.1	9.71	7.82	3.69	8.16E-08	9.83E-07	VDAC3	voltage-dependent anion channel 3	Multiple_Complex
TC0X00007190.hg.1	6.58	7.7	-2.18	8.18E-08	9.85E-07	PORCN	porcupine homolog (Drosophila)	Multiple_Complex
TC0500013155.hg.1	13.83	11.32	5.68	8.28E-08	9.96E-07	BASP1	brain abundant, membrane attached signal pr	Coding
TC1100011184.hg.1	11.39	9.7	3.24	8.28E-08	9.96E-07	SF1	Memczak2013 ALT_ACCEPTOR, ALT_DONOR,	NonCoding
TC0400010965.hg.1	9.19	10.2	-2.01	8.33E-08	1.00E-06	GRSF1	G-rich RNA sequence binding factor 1	Multiple_Complex
TC0500012956.hg.1	10.63	9.01	3.09	8.36E-08	1.00E-06	HK3	hexokinase 3 (white cell)	Multiple_Complex
TC1600007354.hg.1	10.58	11.94	-2.56	8.38E-08	1.01E-06	EIF3C; EIF3	eukaryotic translation initiation factor 3, subu	Multiple_Complex
TC0100014152.hg.1	7.41	8.41	-1.99	8.39E-08	1.01E-06	FAF1	Fas (TNFRSF6) associated factor 1	Multiple_Complex
TC1200007647.hg.1	8.32	6.78	2.91	8.49E-08	1.02E-06	ACVR1B	activin A receptor type IB	Multiple_Complex
TC1300008272.hg.1	7.66	6.38	2.44	8.52E-08	1.02E-06	LATS2	large tumor suppressor kinase 2	Multiple_Complex
TC0800011116.hg.1	7.99	9.08	-2.13	8.55E-08	1.02E-06	RBM12B	RNA binding motif protein 12B	Multiple_Complex
TC0500009411.hg.1	7.58	5.56	4.04	8.61E-08	1.03E-06	RANBP17	RAN binding protein 17	Multiple_Complex
TC1100011331.hg.1	14.1	15.42	-2.49	8.64E-08	1.03E-06	PPP1CA	protein phosphatase 1, catalytic subunit, alpha	Multiple_Complex
TC1000011716.hg.1	9.93	7.17	6.78	8.68E-08	1.04E-06	USMG5; M	up-regulated during skeletal muscle growth 5	Multiple_Complex
TC0600012315.hg.1	7.08	5.92	2.23	8.69E-08	1.04E-06	COX7A2	cytochrome c oxidase subunit VIIa polypeptide	Multiple_Complex
TC1200007622.hg.1	8.08	9.23	-2.21	8.69E-08	1.04E-06	ATF1	activating transcription factor 1	Multiple_Complex
TC0900008516.hg.1	7.06	8.18	-2.17	8.81E-08	1.05E-06	C9orf91	chromosome 9 open reading frame 91	Multiple_Complex
TC0800008279.hg.1	10.25	8.96	2.44	8.81E-08	1.05E-06	PLEKHF2	pleckstrin homology domain containing, famil	Multiple_Complex
TC0900011252.hg.1	9.3	11.05	-3.37	8.82E-08	1.05E-06	FKBP15	FK506 binding protein 15	Multiple_Complex
TC0300011383.hg.1	9.73	8.76	1.96	8.84E-08	1.05E-06	PSMD6	proteasome 26S subunit, non-ATPase 6	Multiple_Complex
TC0X00008253.hg.1	10.3	11.62	-2.5	8.93E-08	1.06E-06	UBE2A	ubiquitin conjugating enzyme E2A	Multiple_Complex
TC1300008280.hg.1	8.19	6.27	3.79	9.04E-08	1.08E-06	SKA3	spindle and kinetochore associated complex s	Multiple_Complex
TC0800010502.hg.1	14.36	11.3	8.36	9.18E-08	1.09E-06	RPS20; SNC	ribosomal protein S20; small nucleolar RNA, C	Multiple_Complex
TC1400010169.hg.1	10.85	9.63	2.32	9.21E-08	1.10E-06	SETD3	SET domain containing 3	Multiple_Complex
TC0500010640.hg.1	5.48	7.19	-3.27	9.22E-08	1.10E-06	C5orf28	chromosome 5 open reading frame 28	Multiple_Complex
TC0600007270.hg.1	14.04	12.26	3.44	9.23E-08	1.10E-06	HIST1H2AC	histone cluster 1, H2ac	Multiple_Complex
TC1700011449.hg.1	16.15	14.88	2.41	9.25E-08	1.10E-06	DDX5; MIR	DEAD (Asp-Glu-Ala-Asp) box helicase 5; micro	Multiple_Complex
TC1900007679.hg.1	5.99	7.32	-2.51	9.28E-08	1.10E-06	PLEKHF1	pleckstrin homology domain containing, famil	Coding
TC1200010481.hg.1	8.95	7.74	2.31	9.30E-08	1.10E-06	TWF1	twinfilin actin binding protein 1	Multiple_Complex
TC0X00006446.hg.1	12.3	10.37	3.8	9.31E-08	1.10E-06	CSF2RA	colony stimulating factor 2 receptor, alpha, lo	Multiple_Complex
TC0100013483.hg.1	7.45	6.1	2.56	9.44E-08	1.12E-06	TAF12	TAF12 RNA polymerase II, TATA box binding p	Multiple_Complex
TC1900011750.hg.1	5.14	6.56	-2.66	9.55E-08	1.13E-06	ZNF155	zinc finger protein 155	Multiple_Complex
TC0800010765.hg.1	13.81	14.64	-1.77	9.56E-08	1.13E-06	TRAM1	translocation associated membrane protein 1	Multiple_Complex
TC1500008052.hg.1	8.02	8.9	-1.83	9.62E-08	1.14E-06	MESDC1	mesoderm development candidate 1	Coding
TC1100007185.hg.1	6.94	8.14	-2.3	9.69E-08	1.15E-06	ELP4	elongator acetyltransferase complex subunit 4	Multiple_Complex

TC0500013191.hg.1	10.19	7.15	8.22	9.81E-08	1.16E-06	NSA2	NSA2 ribosome biogenesis homolog	Multiple_Complex
TC1700008995.hg.1	7.29	8.56	-2.4	9.82E-08	1.16E-06	9-Sep	septin 9	Multiple_Complex
TC1600007141.hg.1	8.99	7.63	2.57	9.84E-08	1.16E-06	LOC81691;	exonuclease NEF-sp; Putative RNA exonucleas	Multiple_Complex
TC1100013094.hg.1	7.81	6.5	2.48	9.84E-08	1.16E-06	PTS	6-pyruvoyltetrahydropterin synthase	Multiple_Complex
TC1700010507.hg.1	7.2	6.25	1.93	9.88E-08	1.17E-06	YWHAEP7	tyrosine 3-monooxygenase/tryptophan 5-mor	Multiple_Complex
TC1700010420.hg.1	7.35	8.77	-2.67	9.91E-08	1.17E-06	SLFN11	schlafen family member 11	Multiple_Complex
TC1900007947.hg.1	5.06	6.31	-2.37	9.97E-08	1.17E-06	ZNF382	zinc finger protein 382	Multiple_Complex
TC1700007097.hg.1	6.54	7.91	-2.57	9.98E-08	1.17E-06	GID4	GID complex subunit 4 homolog	Multiple_Complex
TC1600010647.hg.1	6.29	7.2	-1.89	9.99E-08	1.18E-06	ACD	adrenocortical dysplasia homolog (mouse)	Multiple_Complex
TC0800007809.hg.1	15.12	13.97	2.21	1.00E-07	1.18E-06	YTHDF3	YTH N(6)-methyladenosine RNA binding prote	Multiple_Complex
TC1600011401.hg.1	6.02	6.75	-1.66	1.00E-07	1.18E-06	COQ9	coenzyme Q9	Multiple_Complex
TC0400011087.hg.1	16.95	18.49	-2.91	1.01E-07	1.19E-06	CCNI	cyclin I	Multiple_Complex
TC0200015225.hg.1	6.38	7.77	-2.63	1.01E-07	1.19E-06	HIBCH	3-hydroxyisobutyryl-CoA hydrolase	Multiple_Complex
TC0900008219.hg.1	6.07	4.03	4.12	1.02E-07	1.19E-06	NR4A3	nuclear receptor subfamily 4, group A, membe	Multiple_Complex
TC2200008827.hg.1	7.64	8.79	-2.22	1.03E-07	1.20E-06	TOB2	transducer of ERBB2, 2	Coding
TC0100016006.hg.1	6.04	6.97	-1.89	1.03E-07	1.21E-06	GPATCH4	G-patch domain containing 4	Multiple_Complex
TC0900011612.hg.1	6.92	10.05	-8.74	1.03E-07	1.21E-06	DPM2	dolichyl-phosphate mannosyltransferase poly	Multiple_Complex
TC2200007419.hg.1	10.14	11.06	-1.9	1.03E-07	1.21E-06	TNRC6B	trinucleotide repeat containing 6B	Multiple_Complex
TC1900009752.hg.1	7.87	9.71	-3.6	1.03E-07	1.21E-06	PRDX2	peroxiredoxin 2	Multiple_Complex
TC1200012862.hg.1	8.03	9.25	-2.33	1.03E-07	1.21E-06	VPS33A	vacuolar protein sorting 33 homolog A (S. cere	Multiple_Complex
TC0100014023.hg.1	7.98	9.55	-2.98	1.04E-07	1.21E-06	GPBP1L1	GC-rich promoter binding protein 1-like 1	Multiple_Complex
TC0400010699.hg.1	7.98	9.13	-2.22	1.04E-07	1.21E-06	SCFD2	sec1 family domain containing 2	Multiple_Complex
TC0X00011236.hg.1	4.96	6.47	-2.86	1.04E-07	1.21E-06	RAB39B	Transcript Identified by AceView, Entrez Gene	Unassigned
TC0300009301.hg.1	6.26	4.8	2.76	1.04E-07	1.22E-06	PTX3	pentraxin 3, long	Coding
TC0100008884.hg.1	10.61	8.91	3.26	1.04E-07	1.22E-06	RPF1	ribosome production factor 1 homolog	Coding
TC0300007495.hg.1	9.47	11.12	-3.14	1.05E-07	1.22E-06	SPCS1	signal peptidase complex subunit 1	Multiple_Complex
TC2100006982.hg.1	6.21	7.72	-2.84	1.05E-07	1.22E-06	LINC00649	long intergenic non-protein coding RNA 649	Multiple_Complex
TC1900007265.hg.1	8.09	7.3	1.73	1.06E-07	1.23E-06	FAM32A	family with sequence similarity 32, member A	Coding
TC0100011461.hg.1	6.84	8.31	-2.77	1.06E-07	1.23E-06	DIEXF	digestive organ expansion factor homolog (ze	Multiple_Complex
TC1100007426.hg.1	6.27	7.15	-1.84	1.06E-07	1.23E-06	SLC35C1	solute carrier family 35 (GDP-fucose transport	Coding
TC0300011050.hg.1	6.92	8.24	-2.48	1.06E-07	1.24E-06	QARS; MIR	glutaminyl-tRNA synthetase; microRNA 6890	Multiple_Complex
TC1900007001.hg.1	13.66	11.99	3.18	1.07E-07	1.24E-06	C19orf38	chromosome 19 open reading frame 38	Multiple_Complex
TC0500009580.hg.1	7.44	8.81	-2.6	1.08E-07	1.26E-06	SIMC1	SUMO-interacting motifs containing 1	Multiple_Complex
TC2100008494.hg.1	12.86	14.25	-2.63	1.09E-07	1.26E-06	IFNAR2	interferon (alpha, beta and omega) receptor 2	Multiple_Complex
TC0500008632.hg.1	4.06	5.76	-3.25	1.09E-07	1.27E-06	SLC22A5	solute carrier family 22 (organic cation/carniti	Multiple_Complex
TC0300013834.hg.1	4.64	5.99	-2.55	1.09E-07	1.27E-06	RAD54L2	RAD54-like 2 (S. cerevisiae)	Multiple_Complex
TC1200012761.hg.1	6.8	8.05	-2.39	1.10E-07	1.27E-06	TAS2R31	taste receptor, type 2, member 31	Coding
TC1700009049.hg.1	9.32	7.95	2.59	1.10E-07	1.28E-06	PGS1	phosphatidylglycerophosphate synthase 1	Multiple_Complex
TC0700008010.hg.1	10.34	11.53	-2.27	1.11E-07	1.29E-06	GTF2I	general transcription factor IIi	Multiple_Complex
TC2000007509.hg.1	14.45	12.33	4.36	1.12E-07	1.30E-06	CTSA	cathepsin A	Multiple_Complex
TC1000007878.hg.1	5.32	6.73	-2.66	1.13E-07	1.31E-06	KIF1BP	KIF1 binding protein	Multiple_Complex
TC0400011056.hg.1	9.35	7.84	2.85	1.14E-07	1.31E-06	NUP54	nucleoporin 54kDa	Multiple_Complex
TC1600010490.hg.1	10.74	11.99	-2.37	1.14E-07	1.31E-06	CNOT1; SN	CCR4-NOT transcription complex subunit 1; sr	Multiple_Complex
TC0300010123.hg.1	10.27	8.61	3.16	1.14E-07	1.32E-06	CRBN	cereblon	Multiple_Complex
TC1700008144.hg.1	7.69	9.09	-2.66	1.14E-07	1.32E-06	SP2	Sp2 transcription factor	Multiple_Complex
TC1700008703.hg.1	7.56	9.11	-2.93	1.16E-07	1.34E-06	NOL11	nucleolar protein 11	Multiple_Complex
TC1700011953.hg.1	9.74	8.52	2.34	1.17E-07	1.35E-06	CBX4	chromobox homolog 4	Multiple_Complex
TC0300007251.hg.1	5.05	7	-3.86	1.17E-07	1.35E-06	CXCR6	chemokine (C-X-C motif) receptor 6	Multiple_Complex
TC0600014060.hg.1	4.28	5.34	-2.09	1.19E-07	1.37E-06	PSMG4	proteasome (prosome, macropain) assembly (	Multiple_Complex
TC2000009895.hg.1	7.02	5.74	2.42	1.19E-07	1.37E-06	LINC00493	long intergenic non-protein coding RNA 493	Multiple_Complex
TC2100008529.hg.1	7.88	8.92	-2.05	1.21E-07	1.39E-06	NDUFV3	NADH dehydrogenase (ubiquinone) flavoprote	Multiple_Complex
TC1100013026.hg.1	6.81	7.86	-2.08	1.21E-07	1.39E-06	TTC9C	tetratricopeptide repeat domain 9C	NonCoding
TC0100007556.hg.1	6.85	8.18	-2.51	1.23E-07	1.41E-06	PHACTR4	phosphatase and actin regulator 4	Multiple_Complex
TC0600008109.hg.1	9.93	8.49	2.72	1.25E-07	1.43E-06	VEGFA	vascular endothelial growth factor A	Multiple_Complex
TC1000008736.hg.1	6.29	7.3	-2.03	1.25E-07	1.43E-06	SUFU	suppressor of fused homolog (Drosophila)	Multiple_Complex
TC1200010833.hg.1	7.45	8.6	-2.22	1.25E-07	1.43E-06	CBX5; MIR	chromobox homolog 5; microRNA 3198-2	Multiple_Complex
TC0600007303.hg.1	9.32	11.14	-3.54	1.25E-07	1.43E-06	BTN3A1	butyrophilin, subfamily 3, member A1	Multiple_Complex
TC0100011276.hg.1	8.15	6.67	2.79	1.25E-07	1.43E-06	SNRPE	small nuclear ribonucleoprotein polypeptide E	Multiple_Complex
TC0500010835.hg.1	4.92	3.66	2.4	1.25E-07	1.44E-06	PLK2	polo-like kinase 2	Multiple_Complex
TC0100010654.hg.1	7.14	8.09	-1.93	1.26E-07	1.44E-06	KLHL20	kelch-like family member 20	Multiple_Complex
TC1900009003.hg.1	9.54	10.81	-2.4	1.27E-07	1.45E-06	ZNF134	zinc finger protein 134	Multiple_Complex
TC0800011105.hg.1	8.48	7.05	2.7	1.27E-07	1.45E-06	TRIQK	triple QxxK/R motif containing	Multiple_Complex
TC1000010172.hg.1	8.21	6.91	2.47	1.27E-07	1.45E-06	SVIL	supervillin	Multiple_Complex
TC1000009240.hg.1	6.27	7.43	-2.24	1.28E-07	1.46E-06	EDRF1	erythroid differentiation regulatory factor 1	Multiple_Complex
TC1800006749.hg.1	7.18	8.31	-2.19	1.28E-07	1.47E-06	CEP192	centrosomal protein 192kDa	Multiple_Complex
TC0400012917.hg.1	8.97	10.64	-3.18	1.29E-07	1.47E-06	ARAP2	ArfGAP with RhoGAP domain, ankyrin repeat a	Multiple_Complex
TC0200015336.hg.1	10.44	11.16	-1.65	1.29E-07	1.47E-06	ANKRD44	ankyrin repeat domain 44	Multiple_Complex
TC0400009221.hg.1	8.84	10.26	-2.66	1.29E-07	1.48E-06	MSMO1	methylsterol monooxygenase 1	Multiple_Complex
TC2200009236.hg.1	8.23	7.35	1.84	1.30E-07	1.48E-06	GGT1	gamma-glutamyltransferase 1	Multiple_Complex
TC1600008190.hg.1	6.83	8.19	-2.58	1.30E-07	1.48E-06	SLC7A6	solute carrier family 7 (amino acid transporter	Multiple_Complex

TC0900009654.hg.1	13.33	10.03	9.85	1.32E-07	1.50E-06	RPS6	ribosomal protein S6	Multiple_Complex
TC0400011421.hg.1	8.74	7.12	3.09	1.32E-07	1.50E-06	LAMTOR3	late endosomal/lysosomal adaptor, MAPK anc	Multiple_Complex
TC0600014101.hg.1	6.75	7.92	-2.25	1.32E-07	1.50E-06	MICA	MHC class I polypeptide-related sequence A	Coding
TC1600007312.hg.1	12.9	10.59	4.93	1.32E-07	1.50E-06	IL4R	interleukin 4 receptor	Multiple_Complex
TC1700012263.hg.1	11.75	10.56	2.27	1.32E-07	1.50E-06	DHX8	DEAH (Asp-Glu-Ala-His) box polypeptide 8	Multiple_Complex
TC0600010817.hg.1	6.11	7.43	-2.51	1.32E-07	1.50E-06	NEDD9	Memczak2013 ALT_ACCEPTOR, ALT_DONOR,	NonCoding
TC1500010251.hg.1	6.95	5.68	2.42	1.33E-07	1.50E-06	HOMER2	homer scaffolding protein 2	Multiple_Complex
TC1400010628.hg.1	4.61	5.64	-2.04	1.34E-07	1.52E-06	YLPM1	YLP motif containing 1	NonCoding
TC1300009994.hg.1	10.32	11.24	-1.89	1.35E-07	1.52E-06	ERCC5	excision repair cross-complementation group	Multiple_Complex
TC0300009010.hg.1	9.76	8.73	2.04	1.35E-07	1.53E-06	RNF7	ring finger protein 7	Multiple_Complex
TC0200007702.hg.1	9.72	11	-2.44	1.36E-07	1.54E-06	PAPOLG	poly(A) polymerase gamma	Multiple_Complex
TC0200006536.hg.1	12.8	8.21	24.04	1.36E-07	1.54E-06	RPS7	ribosomal protein S7	Multiple_Complex
TC1400009548.hg.1	7.88	5.9	3.94	1.36E-07	1.54E-06	ERH	enhancer of rudimentary homolog (Drosophila	Multiple_Complex
TC1100012686.hg.1	8.4	6.52	3.66	1.36E-07	1.54E-06	ESAM	endothelial cell adhesion molecule	Multiple_Complex
TC1100007050.hg.1	5.94	7.28	-2.53	1.36E-07	1.54E-06	PRMT3	protein arginine methyltransferase 3	Multiple_Complex
TC0200016578.hg.1	5.74	6.92	-2.26	1.37E-07	1.55E-06	NUP35	nucleoporin 35kDa	Multiple_Complex
TC1400010576.hg.1	11.46	10.6	1.83	1.37E-07	1.55E-06	PABPN1	poly(A) binding protein, nuclear 1	Multiple_Complex
TC0100014340.hg.1	5.79	4.52	2.41	1.37E-07	1.55E-06	TACSTD2	tumor-associated calcium signal transducer 2	Coding
TC0300008849.hg.1	6.52	7.84	-2.5	1.37E-07	1.55E-06	UBA5	ubiquitin-like modifier activating enzyme 5	Multiple_Complex
TC0200008930.hg.1	8.42	9.89	-2.78	1.38E-07	1.55E-06	SLC20A1	solute carrier family 20 (phosphate transporte	Multiple_Complex
TC1700008151.hg.1	8.99	9.84	-1.8	1.39E-07	1.57E-06	NFE2L1	nuclear factor, erythroid 2-like 1	Multiple_Complex
TC0500013414.hg.1	7.19	8.62	-2.7	1.42E-07	1.60E-06	THOC3	THO complex 3	Multiple_Complex
TC0100015194.hg.1	10.73	8.97	3.39	1.42E-07	1.60E-06	SORT1	sortilin 1	Multiple_Complex
TC0X00010642.hg.1	4.67	5.95	-2.43	1.43E-07	1.61E-06	NKRF	NFKB repressing factor	Multiple_Complex
TC1700010826.hg.1	5.56	9.19	-12.32	1.44E-07	1.61E-06	SLC25A39	solute carrier family 25, member 39	Multiple_Complex
TC0700012849.hg.1	6.85	5.32	2.89	1.45E-07	1.63E-06	CLEC5A	C-type lectin domain family 5, member A	Multiple_Complex
TC1100008010.hg.1	7.3	8.21	-1.88	1.45E-07	1.63E-06	CDC42EP2	CDC42 effector protein (Rho GTPase binding)	Multiple_Complex
TC2200009157.hg.1	6.02	7.2	-2.27	1.45E-07	1.63E-06	SBF1	SET binding factor 1	Multiple_Complex
TC1500007238.hg.1	6.15	7.41	-2.39	1.45E-07	1.63E-06	TMOD2	tropomodulin 2 (neuronal)	Multiple_Complex
TC0200016669.hg.1	7.21	8.16	-1.93	1.45E-07	1.63E-06	ASB3	ankyrin repeat and SOCS box containing 3	Multiple_Complex
TC0700012797.hg.1	9.53	12.79	-9.56	1.46E-07	1.63E-06	PARP12	poly(ADP-ribose) polymerase family member	Multiple_Complex
TC0200007628.hg.1	10.73	7.91	7.06	1.46E-07	1.64E-06	RPS27A	ribosomal protein S27a	Multiple_Complex
TC0700007198.hg.1	6.6	5.57	2.05	1.47E-07	1.65E-06	ANLN	anillin actin binding protein	Multiple_Complex
TC1600011442.hg.1	12.28	10.87	2.67	1.47E-07	1.65E-06	MAP1LC3B	microtubule-associated protein 1 light chain 3	Multiple_Complex
TC1700008102.hg.1	9.03	10.83	-3.5	1.48E-07	1.65E-06	LRRC37A2	leucine rich repeat containing 37, member A2	Multiple_Complex
TC0700011782.hg.1	8.7	7.33	2.59	1.48E-07	1.65E-06	FAM133B	family with sequence similarity 133, member	Multiple_Complex
TC0500011173.hg.1	6.1	7.54	-2.7	1.48E-07	1.65E-06	POC5	POC5 centriolar protein	Multiple_Complex
TC0700013080.hg.1	10.26	8.57	3.24	1.50E-07	1.67E-06	RHEB	Ras homolog enriched in brain	Multiple_Complex
TC1600011228.hg.1	4.53	5.66	-2.19	1.50E-07	1.68E-06	PIEZO1	piezo-type mechanosensitive ion channel com	Multiple_Complex
TC2000008678.hg.1	10.58	13.03	-5.46	1.51E-07	1.69E-06	CST3	cystatin C	Multiple_Complex
TC1400009410.hg.1	6.98	8.76	-3.42	1.52E-07	1.69E-06	ZBTB25	zinc finger and BTB domain containing 25	Multiple_Complex
TC0400012769.hg.1	13.85	12.21	3.12	1.54E-07	1.72E-06	BST1	bone marrow stromal cell antigen 1	Multiple_Complex
TC1100012974.hg.1	12.29	10.95	2.53	1.56E-07	1.73E-06	C11orf58	chromosome 11 open reading frame 58	Multiple_Complex
TC1900012046.hg.1	8.3	7.36	1.92	1.57E-07	1.74E-06	RDH13	retinol dehydrogenase 13 (all-trans/9-cis)	Multiple_Complex
TC1000009065.hg.1	5.57	7.02	-2.72	1.60E-07	1.78E-06	FAM45A	Transcript Identified by AceView, Entrez Gene	Unassigned
TC1800009242.hg.1	6.08	4.61	2.77	1.60E-07	1.78E-06	SERPINB2	serpin peptidase inhibitor, clade B (ovalbumin	Multiple_Complex
TC0500012059.hg.1	8.74	9.86	-2.18	1.60E-07	1.78E-06	VDAC1	voltage-dependent anion channel 1	Multiple_Complex
TC0100013013.hg.1	5.38	4.25	2.19	1.62E-07	1.80E-06	UQCRHL	ubiquinol-cytochrome c reductase hinge prote	Multiple_Complex
TC2000009447.hg.1	8.58	6.85	3.31	1.63E-07	1.81E-06	DPM1	dolichyl-phosphate mannosyltransferase poly	Multiple_Complex
TC1200011311.hg.1	14.14	12.6	2.9	1.63E-07	1.81E-06	NAP1L1	nucleosome assembly protein 1-like 1	Multiple_Complex
TC0100011192.hg.1	8.25	9.54	-2.45	1.64E-07	1.81E-06	IPO9	importin 9	Multiple_Complex
TC0300008561.hg.1	11.61	15.37	-13.55	1.64E-07	1.82E-06	PARP14	poly(ADP-ribose) polymerase family member	Multiple_Complex
TC1600007335.hg.1	3.65	4.76	-2.17	1.65E-07	1.83E-06	SBK1	SH3 domain binding kinase 1	Multiple_Complex
TC1900011683.hg.1	6.76	8.43	-3.18	1.66E-07	1.83E-06	HSH2D	hematopoietic SH2 domain containing	Multiple_Complex
TC0700007024.hg.1	10.64	8.64	3.98	1.66E-07	1.84E-06	TAX1BP1	Tax1 (human T-cell leukemia virus type I) bind	Multiple_Complex
TC1700010859.hg.1	7.23	9	-3.41	1.66E-07	1.84E-06	DCAKD	dephospho-CoA kinase domain containing	Multiple_Complex
TC0200009428.hg.1	11.06	12.27	-2.31	1.67E-07	1.84E-06	CCNT2	cyclin T2	Multiple_Complex
TC0100017472.hg.1	7.58	6.72	1.82	1.67E-07	1.84E-06	WDR26	Transcript Identified by AceView, Entrez Gene	Unassigned
TC0400008912.hg.1	5.97	6.9	-1.9	1.68E-07	1.85E-06	MMAA	methylmalonic aciduria (cobalamin deficiency	Multiple_Complex
TC1900012007.hg.1	8	9.03	-2.04	1.70E-07	1.87E-06	NUP62; IL4	nucleoporin 62kDa; interleukin 4 induced 1	Multiple_Complex
TC1300007483.hg.1	5.72	6.86	-2.2	1.70E-07	1.87E-06	BORA	bora, aurora kinase A activator	Multiple_Complex
TC1700008690.hg.1	8.57	10.09	-2.86	1.70E-07	1.87E-06	PITPNC1	phosphatidylinositol transfer protein, cytoplas	Multiple_Complex
TC1900011816.hg.1	5.07	6.48	-2.65	1.71E-07	1.88E-06	ZNF419	zinc finger protein 419	Multiple_Complex
TC0300012323.hg.1	7.69	5.63	4.18	1.74E-07	1.91E-06	MGLL	monoglyceride lipase	Multiple_Complex
TC2200008594.hg.1	13.68	11.71	3.93	1.75E-07	1.92E-06	MYH9	myosin, heavy chain 9, non-muscle	Multiple_Complex
TC1900011668.hg.1	5.9	7.16	-2.4	1.75E-07	1.93E-06	ZNF763	zinc finger protein 763	Coding
TC1400007170.hg.1	6.86	4.99	3.63	1.76E-07	1.93E-06	PSMC6	proteasome 26S subunit, ATPase 6	Multiple_Complex
TC1500008167.hg.1	9.49	10.85	-2.57	1.76E-07	1.93E-06	ZNF592	zinc finger protein 592	Multiple_Complex
TC0X00006614.hg.1	10.58	8.68	3.73	1.76E-07	1.94E-06	MSL3	male-specific lethal 3 homolog (Drosophila)	Multiple_Complex
TC1800009243.hg.1	6.12	4.82	2.47	1.77E-07	1.94E-06	SERPINB10	serpin peptidase inhibitor, clade B (ovalbumin	Multiple_Complex

TC1400010717.hg.1	6.24	7.57	-2.52	1.78E-07	1.95E-06	PPP1R3E	protein phosphatase 1, regulatory subunit 3E	Multiple_Complex
TC1700010960.hg.1	5.75	6.62	-1.83	1.81E-07	1.99E-06	MRPL10	mitochondrial ribosomal protein L10	Multiple_Complex
TC2000010027.hg.1	11.12	11.98	-1.81	1.83E-07	2.01E-06	TMEM189-	TMEM189-UBE2V1 readthrough	Multiple_Complex
TC1600007531.hg.1	12.97	11.02	3.87	1.84E-07	2.01E-06	ITGAX	integrin alpha X	Multiple_Complex
TC0700008667.hg.1	10.28	8.64	3.12	1.84E-07	2.01E-06	PSMC2	proteasome 26S subunit, ATPase 2	Multiple_Complex
TC0500012162.hg.1	6.63	7.75	-2.18	1.85E-07	2.02E-06	BRD8	bromodomain containing 8	Multiple_Complex
TC2000009914.hg.1	6.29	7.7	-2.67	1.85E-07	2.02E-06	TGIF2	TGFB-induced factor homeobox 2	Multiple_Complex
TC1200012234.hg.1	8.38	7.11	2.41	1.85E-07	2.03E-06	CLIP1	CAP-GLY domain containing linker protein 1	Multiple_Complex
TC1100008792.hg.1	7.99	9	-2.02	1.88E-07	2.05E-06	MED17	mediator complex subunit 17	Multiple_Complex
TC1400010257.hg.1	11.46	8.75	6.56	1.88E-07	2.05E-06	HSP90AA1	heat shock protein 90kDa alpha (cytosolic), cla	Multiple_Complex
TC0600012647.hg.1	8.1	9.38	-2.43	1.89E-07	2.06E-06	FBXL4	F-box and leucine-rich repeat protein 4	Multiple_Complex
TC0300013826.hg.1	4.56	5.6	-2.05	1.90E-07	2.08E-06	P4HTM	prolyl 4-hydroxylase, transmembrane (endopl	Multiple_Complex
TC1900011327.hg.1	6.94	7.88	-1.93	1.92E-07	2.09E-06	ZNF160	zinc finger protein 160	Multiple_Complex
TC1100009203.hg.1	10.09	11.69	-3.03	1.93E-07	2.11E-06	UBE4A	ubiquitination factor E4A	Multiple_Complex
TC1000007199.hg.1	10.48	8.51	3.92	1.93E-07	2.11E-06	ZEB1	zinc finger E-box binding homeobox 1	Multiple_Complex
TC0400007495.hg.1	7.58	5.48	4.29	1.94E-07	2.11E-06	DCUN1D4	DCN1, defective in cullin neddylation 1, doma	Multiple_Complex
TC0300008352.hg.1	6.42	7.85	-2.69	1.94E-07	2.11E-06	GTPBP8	GTP-binding protein 8 (putative)	Multiple_Complex
TC0200008806.hg.1	11.23	9.49	3.34	1.95E-07	2.12E-06	LIMS1	LIM and senescent cell antigen-like domains 1	Multiple_Complex
TC1500006956.hg.1	5.39	6.42	-2.05	1.95E-07	2.12E-06	BAHD1	bromo adjacent homology domain containing	Multiple_Complex
TC0100017267.hg.1	8.24	9.21	-1.96	1.96E-07	2.13E-06	ANGEL2	angel homolog 2 (Drosophila)	Multiple_Complex
TC0X00009981.hg.1	15.72	17.17	-2.72	1.97E-07	2.14E-06	IL2RG	interleukin 2 receptor, gamma	Multiple_Complex
TC1600007893.hg.1	8.45	9.55	-2.15	1.98E-07	2.15E-06	FTO	fat mass and obesity associated	Multiple_Complex
TC1200012752.hg.1	6.19	9.07	-7.35	1.98E-07	2.15E-06	KLRC3	killer cell lectin-like receptor subfamily C, mem	Coding
TC0500008863.hg.1	11.69	10.11	2.99	1.98E-07	2.15E-06	IK; MIR365	IK cytokine, down-regulator of HLA II; microRN	Multiple_Complex
TC2200008832.hg.1	6.04	7.21	-2.25	1.98E-07	2.15E-06	DESI1	desumoylating isopeptidase 1	Multiple_Complex
TC0100011873.hg.1	7.2	8.42	-2.34	2.00E-07	2.17E-06	RNF187	ring finger protein 187	Multiple_Complex
TC0800009764.hg.1	5.1	3.93	2.24	2.01E-07	2.18E-06	PSD3	pleckstrin and Sec7 domain containing 3	Multiple_Complex
TC0100013676.hg.1	7.68	8.6	-1.89	2.02E-07	2.18E-06	ZMYM6NB;	ZMYM6 neighbor; zinc finger, MYM-type 6	Multiple_Complex
TC1600011045.hg.1	8.88	10.17	-2.45	2.02E-07	2.19E-06	MBTPS1	membrane bound transcription factor peptida	Multiple_Complex
TC0100014579.hg.1	7.91	9.54	-3.1	2.04E-07	2.20E-06	ZRANB2	zinc finger, RAN-binding domain containing 2	Multiple_Complex
TC1400007500.hg.1	5	3.79	2.32	2.04E-07	2.20E-06	ARG2	arginase 2	Multiple_Complex
TC1900008986.hg.1	8.86	9.71	-1.81	2.04E-07	2.20E-06	ZNF264	zinc finger protein 264	Coding
TC2200008411.hg.1	9.23	11.41	-4.54	2.06E-07	2.22E-06	ASCC2	activating signal cointegrator 1 complex subur	Multiple_Complex
TC0100011777.hg.1	18.32	16.84	2.78	2.08E-07	2.25E-06	H3F3A; H3I	H3 histone, family 3A; H3 histone, family 3A, p	Multiple_Complex
TC0200014414.hg.1	17.4	15.5	3.75	2.09E-07	2.25E-06	CXCR4	chemokine (C-X-C motif) receptor 4	Multiple_Complex
TC0700010716.hg.1	10.23	8.57	3.16	2.09E-07	2.25E-06	DPY19L1	dpy-19-like 1 (C. elegans)	Multiple_Complex
TC0900009470.hg.1	6.73	8.1	-2.59	2.11E-07	2.27E-06	ERMP1	endoplasmic reticulum metallopeptidase 1	Multiple_Complex
TC1900010023.hg.1	8.45	10.51	-4.15	2.12E-07	2.28E-06	FKBP8	FK506 binding protein 8	Multiple_Complex
TC0400012620.hg.1	6.67	5.28	2.62	2.13E-07	2.29E-06	CENPU	centromere protein U	Multiple_Complex
TC0500012497.hg.1	11.95	10.55	2.64	2.14E-07	2.30E-06	TNIP1	TNFAIP3 interacting protein 1	Multiple_Complex
TC0600007301.hg.1	10.81	13.2	-5.23	2.14E-07	2.30E-06	BTN3A2	butyrophilin, subfamily 3, member A2	Multiple_Complex
TC0300008346.hg.1	7.18	8.85	-3.18	2.17E-07	2.33E-06	SLC35A5	solute carrier family 35, member A5	Multiple_Complex
TC1500009159.hg.1	6.75	7.81	-2.08	2.18E-07	2.34E-06	RPAP1	RNA polymerase II associated protein 1	Multiple_Complex
TC0200014991.hg.1	8.45	9.86	-2.66	2.18E-07	2.34E-06	GPR155	G protein-coupled receptor 155	Multiple_Complex
TC0900012163.hg.1	5.55	4.51	2.05	2.19E-07	2.35E-06	ORM2	orosomucoid 2	Multiple_Complex
TC1900012057.hg.1	5.64	6.88	-2.37	2.19E-07	2.35E-06	ZNF814	zinc finger protein 814	Multiple_Complex
TC1300007884.hg.1	8.88	10.53	-3.13	2.19E-07	2.35E-06	TPP2	tripeptidyl peptidase II	Multiple_Complex
TC1900010743.hg.1	14.18	12.73	2.73	2.20E-07	2.35E-06	TGFB1	transforming growth factor beta 1	Multiple_Complex
TC1200009910.hg.1	4.44	5.82	-2.6	2.20E-07	2.36E-06	KLRAP1	killer cell lectin-like receptor subfamily A pseu	Multiple_Complex
TC0400006808.hg.1	5.47	4.13	2.54	2.21E-07	2.36E-06	USP17L22	ubiquitin specific peptidase 17-like family mer	Coding
TC2000008299.hg.1	10.83	9.45	2.6	2.21E-07	2.37E-06	GPCPD1	Transcript Identified by AceView, Entrez Gene	Unassigned
TC1900011733.hg.1	7.44	8.93	-2.82	2.23E-07	2.39E-06	RAB4B; MI/	RAB4B, member RAS oncogene family; MIA-R/	Multiple_Complex
TC1900009608.hg.1	8.64	10	-2.57	2.25E-07	2.40E-06	EIF3G	eukaryotic translation initiation factor 3, subu	Multiple_Complex
TC1700010447.hg.1	16.47	18.48	-4.05	2.25E-07	2.40E-06	CCL5	chemokine (C-C motif) ligand 5	Coding
TC0400012310.hg.1	9.08	10.85	-3.42	2.26E-07	2.41E-06	1-Mar	membrane associated ring finger 1	Multiple_Complex
TC1900007173.hg.1	15.76	14.24	2.87	2.28E-07	2.44E-06	ADGRE5	adhesion G protein-coupled receptor E5	Multiple_Complex
TC0100018477.hg.1	9.07	8.08	1.98	2.32E-07	2.47E-06	RBM8A	RNA binding motif protein 8A	Multiple_Complex
TC1700007361.hg.1	5.52	6.39	-1.82	2.32E-07	2.47E-06	TNFAIP1	tumor necrosis factor, alpha-induced protein 1	Multiple_Complex
TC1600011347.hg.1	4.89	5.79	-1.88	2.33E-07	2.49E-06	ALG1; NAG	ALG1, chitobiosyldiphosphodolichol beta-man	Multiple_Complex
TC1100013050.hg.1	6.78	8.27	-2.82	2.34E-07	2.49E-06	RBM14	RNA binding motif protein 14	Multiple_Complex
TC0800009208.hg.1	4.91	6.04	-2.19	2.35E-07	2.50E-06	ZNF623	zinc finger protein 623	Coding
TC0100012602.hg.1	8.48	9.72	-2.36	2.37E-07	2.52E-06	CEP104	centrosomal protein 104kDa	Multiple_Complex
TC0200013943.hg.1	7.91	9.33	-2.68	2.39E-07	2.54E-06	SLC35F5	solute carrier family 35, member F5	Multiple_Complex
TC0400007572.hg.1	9.21	10.46	-2.37	2.41E-07	2.56E-06	EXOC1	exocyst complex component 1	Multiple_Complex
TC0200014736.hg.1	6.39	7.93	-2.89	2.41E-07	2.56E-06	RBMS1	Jeck2013 ALT_ACCEPTOR, ALT_DONOR, codin	NonCoding
TC0X00008945.hg.1	6.94	8.8	-3.63	2.45E-07	2.60E-06	PRKX	protein kinase, X-linked	Multiple_Complex
TC0600014103.hg.1	6.76	5.45	2.48	2.47E-07	2.62E-06	LY6G6F	lymphocyte antigen 6 complex, locus G6F	Coding
TC0700011556.hg.1	7.58	6.5	2.12	2.47E-07	2.62E-06	HIP1	huntingtin interacting protein 1	Multiple_Complex
TC0300006993.hg.1	10.62	12.03	-2.66	2.48E-07	2.63E-06	CRTAP	cartilage associated protein	Multiple_Complex
TSUnmapped00000135.h	11.72	12.66	-1.91	2.49E-07	2.64E-06	INPP5D	inositol polyphosphate-5-phosphatase D	Coding

TC1700012289.hg.1	6.57	5.38	2.29	2.55E-07	2.70E-06	CA4	carbonic anhydrase IV	Multiple_Complex
TC1700012437.hg.1	10.59	11.55	-1.95	2.58E-07	2.73E-06	SRSF1	serine/arginine-rich splicing factor 1	Multiple_Complex
TC0700006977.hg.1	5.4	6.67	-2.42	2.58E-07	2.73E-06	NFE2L3	nuclear factor, erythroid 2-like 3	Multiple_Complex
TC0100012601.hg.1	8.12	9.07	-1.93	2.59E-07	2.74E-06	LRRC47	leucine rich repeat containing 47	Multiple_Complex
TC0200007854.hg.1	6.51	4.94	2.98	2.59E-07	2.74E-06	MEIS1	Meis homeobox 1	Multiple_Complex
TC0300007387.hg.1	6.83	8.53	-3.24	2.59E-07	2.74E-06	RNF123	ring finger protein 123	Multiple_Complex
TC1200007701.hg.1	6.84	8.15	-2.47	2.59E-07	2.74E-06	ZNF740	zinc finger protein 740	Multiple_Complex
TC2000008237.hg.1	8.98	11.99	-8.02	2.61E-07	2.75E-06	MAVS	Jeck2013 ANTISENSE, coding, INTERNAL, OVE)	NonCoding
TC1000012427.hg.1	5.66	4.36	2.46	2.63E-07	2.77E-06	AKR1C1	aldo-keto reductase family 1, member C1	Multiple_Complex
TC1200007804.hg.1	5.46	4.71	1.68	2.64E-07	2.79E-06	METTL7B	methyltransferase like 7B	Multiple_Complex
TC1200007954.hg.1	12.93	10.74	4.56	2.64E-07	2.79E-06	USP15; MIF	ubiquitin specific peptidase 15; microRNA 612	Multiple_Complex
TC1700009256.hg.1	11.66	9.85	3.51	2.68E-07	2.82E-06	NARF	nuclear prelamin A recognition factor	Multiple_Complex
TC0900009164.hg.1	7.52	9	-2.79	2.69E-07	2.83E-06	MRPS2	mitochondrial ribosomal protein S2	Multiple_Complex
TC0100013636.hg.1	12.54	11.15	2.62	2.70E-07	2.85E-06	RNF19B	ring finger protein 19B	Multiple_Complex
TC0600014332.hg.1	5.66	7.1	-2.72	2.73E-07	2.87E-06	ZBTB24	zinc finger and BTB domain containing 24	Multiple_Complex
TC0400012754.hg.1	6.94	8.28	-2.53	2.75E-07	2.89E-06	ZNF141	zinc finger protein 141	Multiple_Complex
TC0200013046.hg.1	5.6	6.63	-2.05	2.75E-07	2.89E-06	EXOC6B	exocyst complex component 6B	Multiple_Complex
TC1000010026.hg.1	6.08	7.26	-2.27	2.76E-07	2.89E-06	PIP4K2A	phosphatidylinositol-5-phosphate 4-kinase, ty	NonCoding
TC0700009399.hg.1	4.97	6.17	-2.31	2.76E-07	2.90E-06	TAS2R4	taste receptor, type 2, member 4	Coding
TC1500007979.hg.1	8.85	6.14	6.54	2.76E-07	2.90E-06	PSMA4	proteasome subunit alpha 4	Multiple_Complex
TC0200016543.hg.1	7.73	8.95	-2.33	2.77E-07	2.90E-06	RIF1	replication timing regulatory factor 1	Multiple_Complex
TC0200016418.hg.1	6.44	7.47	-2.05	2.77E-07	2.90E-06	ZNF512	zinc finger protein 512	Multiple_Complex
TC0800011005.hg.1	5.57	4.17	2.65	2.81E-07	2.95E-06	REXO1L2P	REX1, RNA exonuclease 1 homolog-like 2, pse	Multiple_Complex
TC0400009978.hg.1	5.99	7.04	-2.07	2.82E-07	2.95E-06	ACOX3	acyl-CoA oxidase 3, pristanoyl	Multiple_Complex
TC2200007037.hg.1	11.27	9.59	3.21	2.83E-07	2.96E-06	MTMR3	myotubularin related protein 3	Multiple_Complex
TC1700010820.hg.1	8.35	13.26	-29.97	2.85E-07	2.98E-06	SLC4A1	solute carrier family 4 (anion exchanger), men	Multiple_Complex
TC1100008827.hg.1	7.03	7.99	-1.95	2.85E-07	2.98E-06	SRSF8	serine/arginine-rich splicing factor 8	Multiple_Complex
TC1700011465.hg.1	7.64	8.8	-2.24	2.85E-07	2.98E-06	LRRC37A3	leucine rich repeat containing 37, member A3	Multiple_Complex
TC2000008001.hg.1	6.83	7.97	-2.21	2.86E-07	2.99E-06	ADRM1	adhesion regulating molecule 1	Multiple_Complex
TC0500013261.hg.1	7.42	8.41	-1.98	2.86E-07	2.99E-06	GALNT10	polypeptide N-acetylgalactosaminyltransferas	Multiple_Complex
TC2100008496.hg.1	6.16	7.99	-3.55	2.87E-07	2.99E-06	SLC5A3; MI	solute carrier family 5 (sodium/myo-inositol c	Multiple_Complex
TC0X00009387.hg.1	9.46	7.47	3.97	2.88E-07	3.01E-06	RPGR	retinitis pigmentosa GTPase regulator	Multiple_Complex
TC1600009942.hg.1	10.4	11.93	-2.88	2.89E-07	3.01E-06	CDIPT	CDP-diacylglycerol--inositol 3-phosphatidyltra	Multiple_Complex
TC0X00009896.hg.1	4.91	5.89	-1.98	2.90E-07	3.03E-06	LAS1L	LAS1-like, ribosome biogenesis factor	Multiple_Complex
TC1800007071.hg.1	7.3	8.13	-1.79	2.90E-07	3.03E-06	ZNF397	zinc finger protein 397	Multiple_Complex
TC0100008517.hg.1	4.8	6.61	-3.51	2.98E-07	3.10E-06	NFIA	nuclear factor I/A	Multiple_Complex
TC0600014240.hg.1	6.61	5.29	2.5	2.99E-07	3.11E-06	LOC10013C	uncharacterized LOC100130357; novel transcı	Multiple_Complex
TC0100012315.hg.1	9.58	8.24	2.52	3.01E-07	3.13E-06	NLRP3	NLR family, pyrin domain containing 3	Multiple_Complex
TC0M00006434.hg.1	14.58	12.36	4.64	3.04E-07	3.16E-06	ND2	MTND2	Multiple_Complex
TC2000010015.hg.1	4.78	4.03	1.69	3.04E-07	3.16E-06	WFDC6	WAP four-disulfide core domain 6	Coding
TC1900011719.hg.1	5.7	6.8	-2.14	3.06E-07	3.18E-06	ZNF420	zinc finger protein 420	Multiple_Complex
TC0200016680.hg.1	4.73	5.73	-2	3.07E-07	3.18E-06	SERTAD2	SERTA domain containing 2	NonCoding
TC1600010651.hg.1	8.05	9.8	-3.36	3.08E-07	3.19E-06	RANBP10	RAN binding protein 10	Multiple_Complex
TC0100007940.hg.1	16.82	14.97	3.59	3.09E-07	3.20E-06	CAP1	CAP, adenylate cyclase-associated protein 1 (y	Multiple_Complex
TC2000007923.hg.1	6.61	7.76	-2.22	3.13E-07	3.25E-06	NELFCD	negative elongation factor complex member C	Multiple_Complex
TC1700007057.hg.1	7.51	6.48	2.04	3.14E-07	3.26E-06	NT5M	5,3-nucleotidase, mitochondrial	Multiple_Complex
TC2000008055.hg.1	8.56	9.46	-1.87	3.14E-07	3.26E-06	ARFGAP1; I	ADP-ribosylation factor GTPase activating pro	Multiple_Complex
TC1000006924.hg.1	11.53	9.65	3.67	3.15E-07	3.26E-06	ARL5B	ADP-ribosylation factor like GTPase 5B	Multiple_Complex
TC0300013970.hg.1	8.35	6.59	3.4	3.17E-07	3.27E-06	PFKFB4; MI	6-phosphofructo-2-kinase/fructose-2,6-bipho:	Multiple_Complex
TC0900009268.hg.1	6.75	7.55	-1.73	3.18E-07	3.29E-06	NDOR1	NADPH dependent diflavin oxidoreductase 1	Multiple_Complex
TC0600010305.hg.1	6.3	7.38	-2.13	3.19E-07	3.30E-06	C6orf120	chromosome 6 open reading frame 120	Coding
TC0200008900.hg.1	7.89	9.13	-2.37	3.20E-07	3.31E-06	TMEM87B	transmembrane protein 87B	Multiple_Complex
TC0100017534.hg.1	7.87	8.99	-2.17	3.27E-07	3.38E-06	ITPKB	inositol-trisphosphate 3-kinase B	Multiple_Complex
TC0600014220.hg.1	10.33	11.63	-2.47	3.28E-07	3.38E-06	SERPINB9	serpin peptidase inhibitor, clade B (ovalbumin	Multiple_Complex
TC0300010680.hg.1	4.73	6.2	-2.77	3.28E-07	3.38E-06	CLASP2	Transcript Identified by AceView, Entrez Gene	Unassigned
TC0400007786.hg.1	5.23	6.47	-2.35	3.31E-07	3.41E-06	UTP3	UTP3, small subunit (SSU) processome compo	Coding
TC0X00008760.hg.1	8.9	7.12	3.44	3.34E-07	3.44E-06	ZNF185	zinc finger protein 185 (LIM domain)	Multiple_Complex
TC0300014081.hg.1	7.27	8.58	-2.49	3.35E-07	3.45E-06	TMEM41A	transmembrane protein 41A	Multiple_Complex
TC1600007007.hg.1	9.32	8.05	2.4	3.35E-07	3.45E-06	PDXDC1	pyridoxal-dependent decarboxylase domain c	Multiple_Complex
TC0600010757.hg.1	5.09	6.67	-2.99	3.37E-07	3.47E-06	SLC35B3	solute carrier family 35 (adenosine 3-phospho	Multiple_Complex
TC0500008785.hg.1	10.42	5.55	29.3	3.41E-07	3.51E-06	EGR1	early growth response 1	Coding
TC0400009110.hg.1	5.37	4.48	1.85	3.42E-07	3.52E-06	GRIA2	glutamate receptor, ionotropic, AMPA 2	Multiple_Complex
TSUnmapped00000336.h	9.53	10.84	-2.48	3.45E-07	3.55E-06	RCC2	regulator of chromosome condensation 2	Coding
TC0700009394.hg.1	7.09	8.2	-2.16	3.46E-07	3.55E-06	AGK	acylglycerol kinase	Multiple_Complex
TC0900011284.hg.1	8.57	9.97	-2.65	3.47E-07	3.56E-06	AKNA	AT-hook transcription factor	Multiple_Complex
TC0200012977.hg.1	10.58	7.97	6.09	3.47E-07	3.56E-06	MXD1	Memczak2013 ANTISENSE, coding, INTERNAL,	NonCoding
TC1900010943.hg.1	7.16	6.16	2.01	3.49E-07	3.58E-06	RTN2	reticulon 2	Multiple_Complex
TC1800007734.hg.1	7.61	8.87	-2.4	3.54E-07	3.63E-06	ZNF236	zinc finger protein 236	Multiple_Complex
TC0X00011401.hg.1	11.09	12.28	-2.28	3.58E-07	3.66E-06	RBMX; SNC	RNA binding motif protein, X-linked; small nuc	Multiple_Complex
TC1100012165.hg.1	13.94	12.05	3.71	3.63E-07	3.72E-06	CASP4	caspase 4	Multiple_Complex

TC1900007819.hg.1	6.48	7.9	-2.69	3.64E-07	3.73E-06	ZNF302	zinc finger protein 302	Multiple_Complex
TC1700007985.hg.1	8.58	7.73	1.8	3.65E-07	3.73E-06	TMUB2	transmembrane and ubiquitin-like domain con	Multiple_Complex
TC0100010152.hg.1	8.34	7.04	2.47	3.68E-07	3.76E-06	LMNA	lamin A/C	Multiple_Complex
TC1200010336.hg.1	5.56	6.74	-2.26	3.69E-07	3.77E-06	YARS2	tyrosyl-tRNA synthetase 2, mitochondrial	Multiple_Complex
TC0200010975.hg.1	12.86	11.63	2.34	3.69E-07	3.77E-06	AGFG1; MII	ArfGAP with FG repeats 1; microRNA 5703	Multiple_Complex
TC0200013894.hg.1	6.4	7.87	-2.77	3.70E-07	3.77E-06	ANAPC1	anaphase promoting complex subunit 1	Multiple_Complex
TC2000010009.hg.1	5.61	6.65	-2.05	3.70E-07	3.77E-06	BLCAP	bladder cancer associated protein	NonCoding
TC0700010046.hg.1	5.16	6.04	-1.83	3.72E-07	3.79E-06	FTSJ2	FtsJ RNA methyltransferase homolog 2 (E. coli	Multiple_Complex
TC0500008304.hg.1	9.2	10.4	-2.29	3.73E-07	3.81E-06	SLC25A46	solute carrier family 25, member 46	Multiple_Complex
TC1500007084.hg.1	11.1	9.93	2.25	3.77E-07	3.84E-06	CASC4	cancer susceptibility candidate 4	Multiple_Complex
TC0600008989.hg.1	6.03	7.01	-1.98	3.78E-07	3.85E-06	QRSL1	glutaminyl-tRNA synthase (glutamine-hydroly	Multiple_Complex
TC2000009887.hg.1	5.15	6.73	-2.98	3.78E-07	3.85E-06	PLCB1	phospholipase C, beta 1 (phosphoinositide-spe	Multiple_Complex
TC1700010204.hg.1	8.44	7.37	2.1	3.79E-07	3.86E-06	SDF2	stromal cell-derived factor 2	Multiple_Complex
TC1900007419.hg.1	4.44	5.42	-1.97	3.79E-07	3.86E-06	SLC25A42	solute carrier family 25, member 42	Multiple_Complex
TC1100007763.hg.1	8.23	6.13	4.28	3.80E-07	3.86E-06	MS4A3	membrane-spanning 4-domains, subfamily A,	Multiple_Complex
TC0400009765.hg.1	8.11	9.48	-2.59	3.82E-07	3.89E-06	MXD4; MIF	MAX dimerization protein 4; microRNA 4800	Multiple_Complex
TC1900006532.hg.1	13.45	16.09	-6.23	3.91E-07	3.98E-06	RPS15	ribosomal protein S15	Multiple_Complex
TC1200010793.hg.1	8.45	7.15	2.46	3.94E-07	4.00E-06	CSAD	cysteine sulfinic acid decarboxylase	Multiple_Complex
TC0600009819.hg.1	6.43	5.54	1.84	3.97E-07	4.03E-06	ULBP2	UL16 binding protein 2	Coding
TC0M00006442.hg.1	18.97	19.67	-1.62	4.05E-07	4.11E-06	ND5	NADH dehydrogenase, subunit 5 (complex I)	Multiple_Complex
TC0500011497.hg.1	11.49	9.49	4.02	4.06E-07	4.11E-06	GLRX	glutaredoxin	Multiple_Complex
TC1200009164.hg.1	7.85	8.76	-1.88	4.08E-07	4.14E-06	RNF34	ring finger protein 34, E3 ubiquitin protein liga	Multiple_Complex
TC1000008056.hg.1	13.11	10.98	4.37	4.11E-07	4.17E-06	VCL	vinculin	Multiple_Complex
TC2200008183.hg.1	5.65	7.79	-4.41	4.14E-07	4.19E-06	VPREB3	pre-B lymphocyte 3	Coding
TC0600011375.hg.1	6.33	7.35	-2.03	4.15E-07	4.20E-06	DHX16	DEAH (Asp-Glu-Ala-His) box polypeptide 16	Multiple_Complex
TC0500007925.hg.1	7.58	8.55	-1.96	4.19E-07	4.24E-06	RASGRF2	Ras protein-specific guanine nucleotide-releas	Multiple_Complex
TC0100010379.hg.1	10.6	11.58	-1.97	4.19E-07	4.24E-06	UHMK1	U2AF homology motif (UHM) kinase 1	Multiple_Complex
TC2000009392.hg.1	12.71	10.33	5.19	4.20E-07	4.25E-06	B4GALT5	UDP-Gal:betaGlcNAc beta 1,4- galactosyltrans	Multiple_Complex
TC1500010866.hg.1	10.61	9.29	2.51	4.23E-07	4.28E-06	RAB27A	RAB27A, member RAS oncogene family	Multiple_Complex
TC1600006685.hg.1	6.06	7.27	-2.31	4.26E-07	4.30E-06	ZNF75A	zinc finger protein 75a	Multiple_Complex
TC1600009954.hg.1	13.29	11.75	2.91	4.31E-07	4.35E-06	MAPK3	mitogen-activated protein kinase 3	Multiple_Complex
TC0600008348.hg.1	9.38	10.43	-2.08	4.32E-07	4.36E-06	ZNF451	zinc finger protein 451	Multiple_Complex
TC0200013567.hg.1	9.69	11.08	-2.64	4.33E-07	4.37E-06	ANKRD36B	ankyrin repeat domain 36B	Multiple_Complex
TC1900006890.hg.1	12.76	14.86	-4.28	4.33E-07	4.37E-06	RPS28	ribosomal protein S28	Multiple_Complex
TC1100006658.hg.1	5.23	6.98	-3.35	4.35E-07	4.38E-06	OR52K1	olfactory receptor, family 52, subfamily K, me	Coding
TC1200012206.hg.1	7.76	8.7	-1.92	4.38E-07	4.41E-06	KDM2B	lysine (K)-specific demethylase 2B	Multiple_Complex
TC0800011239.hg.1	8.58	6.69	3.72	4.38E-07	4.41E-06	COX6C	cytochrome c oxidase subunit VIc	Multiple_Complex
TC1800008750.hg.1	8.1	10.07	-3.91	4.40E-07	4.43E-06	TCF4	transcription factor 4	Multiple_Complex
TC1500009204.hg.1	10.89	11.99	-2.14	4.40E-07	4.43E-06	UBR1	ubiquitin protein ligase E3 component n-recog	Multiple_Complex
TC0100009364.hg.1	10.2	8.42	3.44	4.48E-07	4.50E-06	CSF1	colony stimulating factor 1 (macrophage)	Multiple_Complex
TC1300008113.hg.1	8.17	9.21	-2.05	4.49E-07	4.51E-06	CUL4A	cullin 4A	Multiple_Complex
TC1600010476.hg.1	8.91	9.92	-2.01	4.53E-07	4.54E-06	CSNK2A2	casein kinase 2, alpha prime polypeptide	Multiple_Complex
TC1700011313.hg.1	10.71	9.23	2.8	4.53E-07	4.54E-06	USP32	ubiquitin specific peptidase 32	Multiple_Complex
TC1800007895.hg.1	8.05	9.27	-2.33	4.59E-07	4.60E-06	METTL4	methyltransferase like 4	Multiple_Complex
TC2200006894.hg.1	5.22	3.75	2.77	4.61E-07	4.62E-06	UPB1	ureidopropionase, beta	Multiple_Complex
TC1600007143.hg.1	9.16	8.06	2.14	4.61E-07	4.62E-06	LYRM1	LYR motif containing 1	Multiple_Complex
TC0100014937.hg.1	5.58	6.62	-2.06	4.62E-07	4.62E-06	FAM69A	family with sequence similarity 69, member A	Coding
TC1700007606.hg.1	6.6	7.9	-2.46	4.62E-07	4.62E-06	TAF15	Transcript Identified by AceView, Entrez Gene	Unassigned
TC1400009829.hg.1	6.47	7.65	-2.27	4.66E-07	4.66E-06	CEP128	centrosomal protein 128kDa	Multiple_Complex
TC1500010747.hg.1	8.5	9.66	-2.23	4.66E-07	4.66E-06	RNF111	ring finger protein 111	Multiple_Complex
TC1800009285.hg.1	14.2	9.96	18.92	4.70E-07	4.70E-06	RPL17; SNC	ribosomal protein L17; small nucleolar RNA, C	Multiple_Complex
TC0300009855.hg.1	10.52	8.65	3.64	4.71E-07	4.71E-06	IL1RAP	interleukin 1 receptor accessory protein	Multiple_Complex
TC0200007825.hg.1	8.17	9.18	-2.01	4.72E-07	4.72E-06	CEP68	centrosomal protein 68kDa	Multiple_Complex
TC1200007759.hg.1	8.82	10.07	-2.38	4.73E-07	4.73E-06	COPZ1	coatomer protein complex subunit zeta 1	Multiple_Complex
TC0Y00006730.hg.1	10.58	6.6	15.71	4.79E-07	4.78E-06	EIF1AY	eukaryotic translation initiation factor 1A, Y-lin	Multiple_Complex
TC0700006890.hg.1	5.75	4.38	2.58	4.84E-07	4.83E-06	IL6	interleukin 6	Multiple_Complex
TC1100012613.hg.1	12.91	14.98	-4.17	4.85E-07	4.83E-06	HSPA8; SNO	heat shock 70kDa protein 8; small nucleolar R	Multiple_Complex
TC0800008266.hg.1	7.17	8.43	-2.39	4.85E-07	4.83E-06	DPY19L4	dpy-19-like 4 (C. elegans)	Multiple_Complex
TC1600011342.hg.1	10.78	12.19	-2.67	4.87E-07	4.85E-06	NAA60	N(alpha)-acetyltransferase 60, NatF catalytic s	Multiple_Complex
TC0800009853.hg.1	9.63	8.56	2.11	4.87E-07	4.85E-06	BIN3	bridging integrator 3	Multiple_Complex
TC1500008105.hg.1	9.06	8.12	1.92	4.88E-07	4.85E-06	FAM103A1	family with sequence similarity 103, member	Coding
TC0100012828.hg.1	7.85	7.01	1.78	4.88E-07	4.86E-06	LZIC	leucine zipper and CTNNBIP1 domain containi	Multiple_Complex
TC1400007554.hg.1	6.49	7.73	-2.37	4.90E-07	4.87E-06	SLC39A9	solute carrier family 39, member 9	Multiple_Complex
TC0500011316.hg.1	14.89	11.62	9.64	4.90E-07	4.87E-06	RPS23	ribosomal protein S23	Multiple_Complex
TC1700008895.hg.1	6.26	7.2	-1.92	5.01E-07	4.97E-06	NUP85	nucleoporin 85kDa	Multiple_Complex
TC1700012423.hg.1	6.58	7.69	-2.17	5.02E-07	4.98E-06	LRRC37A4F	leucine rich repeat containing 37, member A4	Multiple_Complex
TSUnmapped00000342.h	7.66	8.39	-1.66	5.03E-07	4.99E-06	TCF20	transcription factor 20 (AR1)	Coding
TSUnmapped00000362.h	7.66	8.39	-1.66	5.03E-07	4.99E-06	TCF20	transcription factor 20 (AR1)	Coding
TC1100013014.hg.1	7.47	9.11	-3.13	5.06E-07	5.02E-06	MS4A7	membrane-spanning 4-domains, subfamily A,	Multiple_Complex
TC1000011964.hg.1	9.39	8.47	1.9	5.07E-07	5.02E-06	RAB11FIP2	RAB11 family interacting protein 2 (class I)	Multiple_Complex

TC0800008547.hg.1	5.23	3.85	2.6	5.15E-07	5.10E-06	PKHD1L1	polycystic kidney and hepatic disease 1 (autos	Multiple_Complex
TC0700009977.hg.1	5.16	3.98	2.28	5.17E-07	5.12E-06	PDGFA	platelet-derived growth factor alpha polypept	Multiple_Complex
TC1200006935.hg.1	10.95	12.73	-3.45	5.19E-07	5.13E-06	ATF7IP	activating transcription factor 7 interacting pr	Multiple_Complex
TC1200010023.hg.1	8.35	9.71	-2.56	5.20E-07	5.14E-06	ERP27	endoplasmic reticulum protein 27	Multiple_Complex
TC0100011417.hg.1	13.08	10.82	4.8	5.22E-07	5.16E-06	CR1L	complement component (3b/4b) receptor 1-li	Multiple_Complex
TC0100007105.hg.1	10.16	7.92	4.73	5.26E-07	5.20E-06	PADI4	peptidyl arginine deiminase, type IV	Multiple_Complex
TC1000009802.hg.1	9.01	7.99	2.04	5.28E-07	5.22E-06	UPF2	UPF2 regulator of nonsense transcripts homol	Multiple_Complex
TC1200012240.hg.1	11.91	10.6	2.48	5.30E-07	5.23E-06	RSRC2	arginine/serine-rich coiled-coil 2	Multiple_Complex
TC1400010774.hg.1	9.06	10.37	-2.48	5.32E-07	5.25E-06	FOXN3	forkhead box N3	Multiple_Complex
TC0600014142.hg.1	14.71	12.81	3.75	5.33E-07	5.25E-06	PTP4A1	protein tyrosine phosphatase type IVA, memb	Multiple_Complex
TC0X00011399.hg.1	5.91	6.98	-2.1	5.35E-07	5.27E-06	FAM127B	family with sequence similarity 127, member	Multiple_Complex
TC0200011362.hg.1	8.43	10.2	-3.41	5.43E-07	5.35E-06	RNPEPL1	arginyl aminopeptidase (aminopeptidase B)-lil	Multiple_Complex
TC0500013387.hg.1	10.11	7.98	4.38	5.50E-07	5.42E-06	RPS14	ribosomal protein S14	Multiple_Complex
TC0X00010506.hg.1	6.18	7.14	-1.95	5.50E-07	5.42E-06	AMMECR1	Alport syndrome, mental retardation, midface	Multiple_Complex
TC1200007959.hg.1	9.28	10.32	-2.06	5.51E-07	5.42E-06	MON2	MON2 homolog, regulator of endosome-to-Go	Multiple_Complex
TC1300008533.hg.1	7.76	9.22	-2.74	5.56E-07	5.47E-06	HSPH1	heat shock 105kDa/110kDa protein 1	Multiple_Complex
TC0100015786.hg.1	10.13	11.48	-2.55	5.56E-07	5.47E-06	POGZ	pogo transposable element with ZNF domain	Multiple_Complex
TC0400008170.hg.1	5.78	7.33	-2.93	5.65E-07	5.55E-06	SMARCAD1	SWI/SNF-related, matrix-associated actin-dep	Multiple_Complex
TC1600010334.hg.1	8.56	6.65	3.74	5.65E-07	5.55E-06	LOC643802	u3 small nucleolar ribonucleoprotein protein I	Multiple_Complex
TC0200012318.hg.1	5.31	4.41	1.87	5.71E-07	5.60E-06	CDKL4	cyclin-dependent kinase-like 4	Multiple_Complex
TC0700010182.hg.1	7.75	8.97	-2.34	5.72E-07	5.61E-06	PMS2	PMS1 homolog 2, mismatch repair system cor	Multiple_Complex
TC1500010881.hg.1	7.55	6.56	1.98	5.72E-07	5.61E-06	TIPIN; RPL9	TIMELESS interacting protein; ribosomal prote	Multiple_Complex
TC1100011930.hg.1	7.58	9.56	-3.93	5.72E-07	5.61E-06	CHORDC1	cysteine and histidine rich domain containing	Multiple_Complex
TC1400006519.hg.1	7.85	9.03	-2.26	5.74E-07	5.62E-06	APEX1	APEX nuclease (multifunctional DNA repair en	Multiple_Complex
TC0800012422.hg.1	11.14	9.81	2.5	5.81E-07	5.70E-06	VCPIP1	valosin containing protein (p97)/p47 complex	Multiple_Complex
TC1900008326.hg.1	6.83	5.05	3.44	5.95E-07	5.82E-06	FOSB	FBJ murine osteosarcoma viral oncogene hom	Multiple_Complex
TC1700006525.hg.1	7.72	8.91	-2.28	5.95E-07	5.83E-06	RPA1	replication protein A1	Multiple_Complex
TC0200016668.hg.1	5.28	6.17	-1.85	5.97E-07	5.85E-06	GPR75	G protein-coupled receptor 75	Coding
TC0200016702.hg.1	5.2	6.2	-2	6.00E-07	5.87E-06	POLR1A	polymerase (RNA) I polypeptide A	Multiple_Complex
TC1600010341.hg.1	9.77	8.48	2.44	6.05E-07	5.91E-06	AKTIP	AKT interacting protein	Multiple_Complex
TC1200010119.hg.1	8.48	9.67	-2.28	6.08E-07	5.94E-06	C2CD5	C2 calcium-dependent domain containing 5	Multiple_Complex
TC0900010352.hg.1	8.78	9.8	-2.03	6.11E-07	5.97E-06	PTAR1	protein prenyltransferase alpha subunit repea	Multiple_Complex
TC0300012498.hg.1	8.94	7.43	2.85	6.16E-07	6.01E-06	ANAPC13	anaphase promoting complex subunit 13	Multiple_Complex
TC0600009961.hg.1	6.09	7.34	-2.39	6.16E-07	6.01E-06	ARID1B	AT rich interactive domain 1B (SWI1-like)	Multiple_Complex
TC1300007861.hg.1	5.39	6.54	-2.23	6.32E-07	6.16E-06	PCCA	propionyl-CoA carboxylase alpha subunit	Multiple_Complex
TC1500010256.hg.1	10.04	11.29	-2.38	6.33E-07	6.17E-06	BTBD1	BTB (POZ) domain containing 1	Multiple_Complex
TC0200015216.hg.1	8.07	9.55	-2.78	6.37E-07	6.21E-06	ORMDL1	ORMDL sphingolipid biosynthesis regulator 1	Multiple_Complex
TC0X00011180.hg.1	6.09	5.28	1.75	6.41E-07	6.24E-06	RENBP	renin binding protein	Multiple_Complex
TC0300009799.hg.1	4.9	8.08	-9.04	6.46E-07	6.29E-06	RTP4	receptor (chemosensory) transporter protein ·	Coding
TC1900010967.hg.1	9.2	6.71	5.65	6.52E-07	6.35E-06	PGLYRP1	peptidoglycan recognition protein 1	Coding
TC1900007405.hg.1	6.36	7.1	-1.68	6.53E-07	6.35E-06	CRTC1	CREB regulated transcription coactivator 1	Multiple_Complex
TC0400006706.hg.1	11.79	10.05	3.34	6.55E-07	6.37E-06	TBC1D14	TBC1 domain family, member 14	Multiple_Complex
TC2000006852.hg.1	6.12	4.88	2.36	6.56E-07	6.38E-06	SLC24A3	solute carrier family 24 (sodium/potassium/ca	Coding
TC1600008580.hg.1	4.76	5.83	-2.1	6.61E-07	6.42E-06	GAN; MIR4	gigaxonin; microRNA 4720	Multiple_Complex
TC1400008929.hg.1	7.68	6.8	1.84	6.62E-07	6.43E-06	PPP2R3C	protein phosphatase 2, regulatory subunit B, g	Multiple_Complex
TC1400010616.hg.1	6.69	7.89	-2.3	6.62E-07	6.43E-06	SLC38A6	solute carrier family 38, member 6	Multiple_Complex
TC0600006793.hg.1	6.49	7.48	-2	6.63E-07	6.43E-06	CDYL	chromodomain protein, Y-like	Multiple_Complex
TC1400010630.hg.1	8.24	9.28	-2.05	6.63E-07	6.43E-06	TTLL5	tubulin tyrosine ligase-like family member 5	Multiple_Complex
TC1400008333.hg.1	13.19	14.47	-2.43	6.64E-07	6.44E-06	PPP2R5C	protein phosphatase 2, regulatory subunit B, g	Multiple_Complex
TC0100007666.hg.1	7.26	5.59	3.18	6.65E-07	6.44E-06	KPNA6	Jeck2013 ALT_ACCEPTOR, ALT_DONOR, codin	NonCoding
TC0600008253.hg.1	6.32	7.44	-2.16	6.66E-07	6.45E-06	PAQR8	progestin and adipoQ receptor family membe	Coding
TC0200012959.hg.1	7.82	6.52	2.47	6.74E-07	6.52E-06	NFU1	NFU1 iron-sulfur cluster scaffold	Multiple_Complex
TC0700010585.hg.1	8.59	10.15	-2.96	6.74E-07	6.52E-06	JAZF1	JAZF zinc finger 1	Multiple_Complex
TC0700010509.hg.1	9.48	8.11	2.58	6.76E-07	6.53E-06	CYCS	cytochrome c, somatic	Coding
TC0800009511.hg.1	3.62	4.99	-2.58	6.78E-07	6.55E-06	SGK223	homolog of rat pragma of Rnd2; Tyrosine-prot	Coding
TC1400007558.hg.1	11.08	9.61	2.76	6.80E-07	6.57E-06	SUSD6	sushi domain containing 6	Multiple_Complex
TC1400010629.hg.1	8.38	6.21	4.5	6.82E-07	6.58E-06	FLVCR2	feline leukemia virus subgroup C cellular rece	Multiple_Complex
TC0900009106.hg.1	6.55	7.52	-1.96	6.82E-07	6.58E-06	WDR5	WD repeat domain 5	Multiple_Complex
TC1100013081.hg.1	7.01	5.9	2.17	6.84E-07	6.60E-06	FUT4	fucosyltransferase 4 (alpha (1,3) fucosyltransf	Coding
TC0300010714.hg.1	10.34	11.49	-2.22	6.85E-07	6.61E-06	TRANK1	tetratricopeptide repeat and ankyrin repeat co	Multiple_Complex
TC0200007363.hg.1	5.4	6.5	-2.14	6.86E-07	6.62E-06	MTA3	metastasis associated 1 family member 3	Multiple_Complex
TC2000007859.hg.1	10.29	13.03	-6.65	6.87E-07	6.62E-06	RBM38	RNA binding motif protein 38	Multiple_Complex
TC0100010140.hg.1	8.87	10.16	-2.44	6.92E-07	6.67E-06	SYT11	synaptotagmin XI	Coding
TC0900009829.hg.1	7.4	6.27	2.2	6.93E-07	6.67E-06	NDUFB6	NADH dehydrogenase (ubiquinone) 1 beta sul	Coding
TC1000012483.hg.1	6.24	5.19	2.07	6.97E-07	6.71E-06	C10orf131	chromosome 10 open reading frame 131	Coding
TC0700011562.hg.1	9.31	8.17	2.21	6.98E-07	6.71E-06	TMEM120A	transmembrane protein 120A	Multiple_Complex
TC0300006622.hg.1	4.74	3.8	1.93	7.08E-07	6.81E-06	PPARG	peroxisome proliferator-activated receptor ga	Multiple_Complex
TC0900008483.hg.1	7.88	9.46	-3.01	7.11E-07	6.83E-06	SLC31A1	solute carrier family 31 (copper transporter), r	Multiple_Complex
TC1200008028.hg.1	6.49	5.11	2.6	7.28E-07	7.00E-06	MSRB3	methionine sulfoxide reductase B3	Multiple_Complex
TC0100018390.hg.1	9.02	10.1	-2.11	7.35E-07	7.05E-06	SLC35E2	solute carrier family 35, member E2	Multiple_Complex

TC2100007797.hg.1	13.08	10.37	6.53	7.37E-07	7.07E-06	ATP5J	ATP synthase, H+ transporting, mitochondrial	Multiple_Complex
TC0500013301.hg.1	8.27	6.71	2.96	7.38E-07	7.08E-06	DAB2	Dab, mitogen-responsive phosphoprotein, hom	Multiple_Complex
TC1900010941.hg.1	6.2	7.26	-2.09	7.42E-07	7.11E-06	ERCC1	excision repair cross-complementation group	Multiple_Complex
TC1200011573.hg.1	7.75	8.91	-2.23	7.42E-07	7.11E-06	NR2C1	nuclear receptor subfamily 2, group C, membe	Multiple_Complex
TC2100006903.hg.1	8.44	7.14	2.46	7.44E-07	7.13E-06	SOD1	superoxide dismutase 1, soluble	Multiple_Complex
TC2200007206.hg.1	6.41	7.6	-2.27	7.47E-07	7.15E-06	MCM5	minichromosome maintenance complex comp	Multiple_Complex
TC1500010908.hg.1	8.09	9.32	-2.35	7.61E-07	7.28E-06	STARD5	StAR-related lipid transfer domain containing	NonCoding
TC2200008597.hg.1	6.17	5.18	1.99	7.62E-07	7.29E-06	MYH9	Zhang2013 ALT_ACCEPTOR, ALT_DONOR, cod	NonCoding
TC0500008854.hg.1	11.3	10.6	1.63	7.63E-07	7.30E-06	ANKHD1; E	ankyrin repeat and KH domain containing 1; e	Multiple_Complex
TC0200007954.hg.1	6.94	8.34	-2.64	7.65E-07	7.32E-06	PCYOX1	prenylcysteine oxidase 1	Multiple_Complex
TC0300010891.hg.1	5.71	6.77	-2.1	7.67E-07	7.32E-06	ZNF852	zinc finger protein 852	Multiple_Complex
TC1000010499.hg.1	7.07	8.07	-2	7.69E-07	7.34E-06	ZFAND4	zinc finger, AN1-type domain 4	Multiple_Complex
TC0900012176.hg.1	4.98	6.27	-2.44	7.79E-07	7.43E-06	URM1	ubiquitin related modifier 1	Multiple_Complex
TC1000010438.hg.1	11.41	12.48	-2.1	7.85E-07	7.49E-06	HNRNPF	heterogeneous nuclear ribonucleoprotein F	Multiple_Complex
TC0900011655.hg.1	7.79	9.86	-4.19	7.86E-07	7.50E-06	ZER1	zyg-11 related, cell cycle regulator	Multiple_Complex
TC1100008409.hg.1	5.49	6.51	-2.03	7.89E-07	7.52E-06	PPME1	protein phosphatase methylesterase 1	Multiple_Complex
TC1700012442.hg.1	8.6	7.79	1.76	7.90E-07	7.53E-06	TBC1D3P1-	TBC1D3P1-DHX40P1 readthrough transcribed	Multiple_Complex
TC0100009195.hg.1	7.95	9.03	-2.12	7.94E-07	7.56E-06	SLC35A3	solute carrier family 35 (UDP-N-acetylglucosar	Multiple_Complex
TC0M00006454.hg.1	17	14.77	4.68	7.98E-07	7.59E-06	ND6	NADH dehydrogenase, subunit 6 (complex I)	Multiple_Complex
TC0X00007132.hg.1	7.6	6.16	2.71	7.99E-07	7.60E-06	CDK16	cyclin-dependent kinase 16	Multiple_Complex
TC1600008003.hg.1	7.76	9.03	-2.42	8.00E-07	7.60E-06	ADGRG5	adhesion G protein-coupled receptor G5	Multiple_Complex
TC0600013727.hg.1	12.04	13.53	-2.81	8.00E-07	7.60E-06	EZR	ezrin	Multiple_Complex
TC0600013341.hg.1	9.19	8.1	2.13	8.01E-07	7.61E-06	GVQW2	GVQW motif containing 2	Multiple_Complex
TC1800007198.hg.1	5.93	8.51	-5.99	8.02E-07	7.62E-06	SLC14A1	solute carrier family 14 (urea transporter), me	Multiple_Complex
TC0100011267.hg.1	10.09	12.3	-4.63	8.03E-07	7.63E-06	ATP2B4	ATPase, Ca++ transporting, plasma membrane	Multiple_Complex
TC1500009904.hg.1	13.14	11.33	3.49	8.06E-07	7.65E-06	TLE3	transducin-like enhancer of split 3	Multiple_Complex
TC0400012945.hg.1	14.82	12.85	3.92	8.11E-07	7.69E-06	HSD17B11	hydroxysteroid (17-beta) dehydrogenase 11	Multiple_Complex
TC0600007585.hg.1	8.69	10.06	-2.59	8.15E-07	7.73E-06	MICB	MHC class I polypeptide-related sequence B	Multiple_Complex
TC0600011516.hg.1	4.09	5.38	-2.45	8.26E-07	7.83E-06	HLA-DOA	major histocompatibility complex, class II, DO	Multiple_Complex
TC0400007840.hg.1	6.09	5.21	1.84	8.33E-07	7.89E-06	CXCL1	chemokine (C-X-C motif) ligand 1 (melanoma	Multiple_Complex
TC0400012078.hg.1	5.72	7.37	-3.14	8.33E-07	7.89E-06	NR3C2	nuclear receptor subfamily 3, group C, membe	Multiple_Complex
TC1200007595.hg.1	8.25	9.52	-2.42	8.38E-07	7.93E-06	SMARCD1	SWI/SNF related, matrix associated, actin depe	Multiple_Complex
TC0200016452.hg.1	12.32	11.59	1.65	8.41E-07	7.95E-06	REL	v-rel avian reticuloendotheliosis viral oncogen	Multiple_Complex
TC1400007890.hg.1	10.8	12.05	-2.37	8.43E-07	7.97E-06	GPR65	G protein-coupled receptor 65	Coding
TC1300009273.hg.1	9.34	6.24	8.59	8.46E-07	8.00E-06	COMMD6	COMM domain containing 6	Multiple_Complex
TC1100012812.hg.1	5.51	6.7	-2.28	8.52E-07	8.05E-06	PRDM10	PR domain containing 10	Multiple_Complex
TC0600009144.hg.1	7.1	7.92	-1.76	8.58E-07	8.10E-06	KIAA1919	KIAA1919	Multiple_Complex
TC0300007485.hg.1	5.38	6.74	-2.57	8.61E-07	8.13E-06	SMIM4	small integral membrane protein 4	Multiple_Complex
TC1600009856.hg.1	9.85	10.91	-2.09	8.64E-07	8.15E-06	EIF3C; EIF3	eukaryotic translation initiation factor 3, subu	Multiple_Complex
TC1500010407.hg.1	15.66	13.08	6.01	8.67E-07	8.18E-06	ANPEP	alanyl (membrane) aminopeptidase	Multiple_Complex
TC1100009918.hg.1	8.9	10.51	-3.05	8.68E-07	8.19E-06	HBD	hemoglobin, delta	Coding
TC0500007966.hg.1	7.98	6.07	3.77	8.69E-07	8.19E-06	XRCC4	X-ray repair complementing defective repair in	Multiple_Complex
TC0100009418.hg.1	13.29	12.17	2.17	8.74E-07	8.24E-06	RAP1A	RAP1A, member of RAS oncogene family	Multiple_Complex
TC0100014762.hg.1	7.17	6.03	2.21	8.76E-07	8.24E-06	SSX2IP	synovial sarcoma, X breakpoint 2 interacting p	Multiple_Complex
TC0800008183.hg.1	10.46	8.99	2.76	8.79E-07	8.27E-06	OSGIN2	oxidative stress induced growth inhibitor fami	Multiple_Complex
TC0200008099.hg.1	8.03	6.78	2.38	8.81E-07	8.29E-06	HK2	hexokinase 2	Multiple_Complex
TC1700010041.hg.1	7.25	8.39	-2.21	8.84E-07	8.32E-06	AKAP10	A kinase (PRKA) anchor protein 10	Multiple_Complex
TC1600009514.hg.1	8.49	9.66	-2.25	8.99E-07	8.45E-06	RRN3	RRN3 homolog, RNA polymerase I transcriptio	Multiple_Complex
TC2000009550.hg.1	7.73	6.91	1.77	9.00E-07	8.46E-06	AURKA	aurora kinase A	Multiple_Complex
TC0500012248.hg.1	6.95	5.42	2.88	9.05E-07	8.50E-06	NDUFA2	NADH dehydrogenase (ubiquinone) 1 alpha su	Multiple_Complex
TC0900012256.hg.1	7.57	5.8	3.41	9.06E-07	8.50E-06	CDC14B	cell division cycle 14B	Multiple_Complex
TC0500008691.hg.1	12.98	11.63	2.56	9.11E-07	8.55E-06	UBE2B	ubiquitin conjugating enzyme E2B	Multiple_Complex
TC1500008286.hg.1	7.64	9	-2.57	9.14E-07	8.57E-06	ZNF710	zinc finger protein 710	Multiple_Complex
TC1100013100.hg.1	11.07	9.24	3.57	9.24E-07	8.66E-06	ATP5L	ATP synthase, H+ transporting, mitochondrial	Multiple_Complex
TC1700011038.hg.1	9.52	11.28	-3.4	9.29E-07	8.70E-06	ZNF652	zinc finger protein 652	Multiple_Complex
TC1000008712.hg.1	5.12	6.76	-3.11	9.35E-07	8.76E-06	PPRC1	peroxisome proliferator-activated receptor ga	Multiple_Complex
TC0900009946.hg.1	5.31	6.37	-2.09	9.49E-07	8.88E-06	SIT1	signaling threshold regulating transmembrane	Multiple_Complex
TC0100008692.hg.1	9.56	10.54	-1.97	9.54E-07	8.92E-06	SRSF11	serine/arginine-rich splicing factor 11	Multiple_Complex
TC0700009020.hg.1	8.2	9.2	-2	9.55E-07	8.93E-06	SND1; SND	staphylococcal nuclease and tudor domain co	Multiple_Complex
TC1000008713.hg.1	7.79	9.07	-2.42	9.56E-07	8.94E-06	NOLC1	nucleolar and coiled-body phosphoprotein 1	Multiple_Complex
TC1200006520.hg.1	6.94	5.4	2.92	9.58E-07	8.95E-06	TSPAN9	tetraspanin 9	Multiple_Complex
TC1500009004.hg.1	7.51	8.14	-1.55	9.60E-07	8.97E-06	AQR	aquarius intron-binding spliceosomal factor	Multiple_Complex
TC0500010197.hg.1	7.41	6.55	1.81	9.67E-07	9.03E-06	ZNF622	zinc finger protein 622	Multiple_Complex
TC0900008223.hg.1	7.49	8.46	-1.95	9.67E-07	9.03E-06	INVS	inversin	Multiple_Complex
TC0400010078.hg.1	5.03	6.39	-2.56	9.68E-07	9.03E-06	RAB28	Jeck2013 ALT_ACCEPTOR, ALT_DONOR, codin	NonCoding
TC0100013995.hg.1	6.43	7.33	-1.86	9.73E-07	9.08E-06	EIF2B3	eukaryotic translation initiation factor 2B, sub	Multiple_Complex
TC0100018299.hg.1	6.15	7.16	-2.02	9.74E-07	9.08E-06	SLC27A3	solute carrier family 27 (fatty acid transporter	Multiple_Complex
TC0100016973.hg.1	8	6.76	2.36	9.80E-07	9.13E-06	CYB5R1	cytochrome b5 reductase 1	Multiple_Complex
TC0100012167.hg.1	5.02	6.08	-2.09	9.81E-07	9.14E-06	KMO	kynurenine 3-monooxygenase (kynurenine 3-l	Multiple_Complex
TC0400012947.hg.1	8.6	9.45	-1.81	9.84E-07	9.16E-06	GPRIN3	GPRIN family member 3	Coding

TC1700010927.hg.1	10.53	11.55	-2.03	9.87E-07	9.18E-06	CDC27	cell division cycle 27	Multiple_Complex
TC2000009782.hg.1	9.75	10.52	-1.7	9.87E-07	9.18E-06	DIDO1	death inducer-obliterator 1	Multiple_Complex
TC1600007973.hg.1	7.09	8.2	-2.16	9.93E-07	9.24E-06	NUP93	nucleoporin 93kDa	Multiple_Complex
TC0400006810.hg.1	5.97	4.73	2.36	9.96E-07	9.25E-06	USP17L23	ubiquitin specific peptidase 17-like family mer	Coding
TC0400006800.hg.1	6.05	5.01	2.06	1.00E-06	9.31E-06	USP17L18;	ubiquitin specific peptidase 17-like family mer	Coding
TC0700007857.hg.1	9.7	10.78	-2.1	1.01E-06	9.36E-06	TMEM248	transmembrane protein 248	Multiple_Complex
TC1200011846.hg.1	11.41	10.13	2.45	1.01E-06	9.40E-06	CORO1C	coronin, actin binding protein, 1C	Multiple_Complex
TC0900010922.hg.1	5.02	6.32	-2.46	1.01E-06	9.41E-06	ZNF510	zinc finger protein 510	Multiple_Complex
TC0700013420.hg.1	13.02	11.27	3.36	1.02E-06	9.44E-06	ARPC1B	actin related protein 2/3 complex subunit 1B	Multiple_Complex
TC0400006789.hg.1	5.44	4.13	2.47	1.02E-06	9.48E-06	USP17L13	ubiquitin specific peptidase 17-like family mer	Coding
TC2100008114.hg.1	4.09	3.24	1.8	1.02E-06	9.48E-06	KCNJ6	potassium channel, inwardly rectifying subfan	Multiple_Complex
TC0600011441.hg.1	10.49	12.32	-3.55	1.03E-06	9.50E-06	BAG6	BCL2 associated athanogene 6	Multiple_Complex
TC1800007218.hg.1	10.12	8.76	2.56	1.03E-06	9.51E-06	C18orf25	chromosome 18 open reading frame 25	Multiple_Complex
TC1100010990.hg.1	7.01	8.42	-2.66	1.03E-06	9.51E-06	MRPL16	mitochondrial ribosomal protein L16	Multiple_Complex
TC1700012276.hg.1	5.54	6.59	-2.06	1.03E-06	9.55E-06	PNPO	pyridoxamine 5-phosphate oxidase	Multiple_Complex
TC1200011961.hg.1	5.61	7.16	-2.93	1.05E-06	9.72E-06	TMEM116	transmembrane protein 116	Multiple_Complex
TC1700008842.hg.1	17.47	18.75	-2.43	1.05E-06	9.72E-06	RPL38	ribosomal protein L38	Multiple_Complex
TC1900008240.hg.1	7.99	9.28	-2.45	1.05E-06	9.72E-06	ZNF283	zinc finger protein 283	Multiple_Complex
TSUnmapped00000439.h	6.11	5.14	1.96	1.05E-06	9.72E-06	TRAPPC4	trafficking protein particle complex 4	Coding
TC1900012021.hg.1	6.21	7.25	-2.06	1.05E-06	9.73E-06	ZNF841	zinc finger protein 841	Multiple_Complex
TC0300009673.hg.1	9.57	8.53	2.05	1.06E-06	9.73E-06	KLHL24	kelch-like family member 24	Multiple_Complex
TC0800006978.hg.1	6.17	7.2	-2.03	1.06E-06	9.73E-06	POLR3D	polymerase (RNA) III (DNA directed) polypepti	Multiple_Complex
TC1900006687.hg.1	8.06	7	2.09	1.07E-06	9.85E-06	AC007292.1	Transcript Identified by AceView, Entrez Gene	NonCoding
TC0600011439.hg.1	7.4	8.46	-2.09	1.07E-06	9.85E-06	NCR3	natural cytotoxicity triggering receptor 3	Multiple_Complex
TSUnmapped00000180.h	5.2	6.06	-1.82	1.07E-06	9.87E-06	KIAA1143	KIAA1143 [Source:HGNC Symbol;Acc:HGNC:29	Coding
TC0700010275.hg.1	10.03	7.91	4.35	1.07E-06	9.87E-06	NDUFA4	NDUFA4, mitochondrial complex associated	Multiple_Complex
TC1700009731.hg.1	9.23	7.78	2.73	1.07E-06	9.89E-06	GAS7	growth arrest-specific 7	Multiple_Complex
TC1200006700.hg.1	8.12	9.48	-2.57	1.08E-06	9.90E-06	FOXJ2	forkhead box J2	Multiple_Complex
TC1800009056.hg.1	5.95	7.31	-2.57	1.08E-06	9.90E-06	ZADH2	zinc binding alcohol dehydrogenase domain co	Coding
TC1000008467.hg.1	9.87	8.94	1.9	1.08E-06	9.92E-06	5-Mar	membrane associated ring finger 5	Multiple_Complex
TC1600008128.hg.1	7.17	8.18	-2.02	1.09E-06	9.96E-06	CMTM3	CKLF-like MARVEL transmembrane domain co	Multiple_Complex
TC1100012508.hg.1	14.59	10.8	13.91	1.09E-06	9.96E-06	RPS25	ribosomal protein S25	Multiple_Complex
TC0500008641.hg.1	7.69	9.28	-3.02	1.09E-06	1.00E-05	RAD50	RAD50 homolog, double strand break repair p	Multiple_Complex
TC2200008829.hg.1	5.43	6.5	-2.1	1.09E-06	1.00E-05	POLR3H	polymerase (RNA) III (DNA directed) polypepti	Multiple_Complex
TC1300008660.hg.1	5.89	7.52	-3.09	1.10E-06	1.01E-05	PROSER1	proline and serine rich 1	Multiple_Complex
TC1000006796.hg.1	7.61	6.3	2.48	1.10E-06	1.01E-05	SEC61A2	Sec61 translocon alpha 2 subunit	Multiple_Complex
TC0400006785.hg.1	6.23	4.85	2.59	1.10E-06	1.01E-05	USP17L11;	ubiquitin specific peptidase 17-like family mer	Coding
TC1700009801.hg.1	5.89	6.68	-1.74	1.11E-06	1.02E-05	ELAC2	elaC ribonuclease Z 2	Multiple_Complex
TC2100008297.hg.1	6.53	5.07	2.75	1.12E-06	1.03E-05	SIK1	salt-inducible kinase 1	Multiple_Complex
TC0600013409.hg.1	7.02	8.21	-2.29	1.12E-06	1.03E-05	HIVEP2	human immunodeficiency virus type I enhanc	Multiple_Complex
TC1900010011.hg.1	7.78	8.5	-1.64	1.13E-06	1.03E-05	LSM4	LSM4 homolog, U6 small nuclear RNA and mR	Multiple_Complex
TSUnmapped00000475.h	9.94	7.92	4.06	1.13E-06	1.03E-05	PADI4	peptidyl arginine deiminase, type IV	NonCoding
TC0300006493.hg.1	7.13	6.02	2.16	1.13E-06	1.03E-05	EDEM1	Transcript Identified by AceView, Entrez Gene	Unassigned
TC0800008150.hg.1	9.04	8.17	1.82	1.13E-06	1.03E-05	CPNE3	copine III	Multiple_Complex
TC0600007864.hg.1	6.64	8.06	-2.67	1.13E-06	1.03E-05	TBC1D22B	TBC1 domain family, member 22B	Multiple_Complex
TC1600011494.hg.1	10.61	9.45	2.23	1.14E-06	1.04E-05	ARL6IP1	ADP-ribosylation factor like GTPase 6 interacti	Multiple_Complex
TC0100008073.hg.1	5.6	6.86	-2.4	1.15E-06	1.05E-05	KDM4A	lysine (K)-specific demethylase 4A	Multiple_Complex
TC0300010299.hg.1	9.8	8.33	2.79	1.15E-06	1.05E-05	RAF1	Raf-1 proto-oncogene, serine/threonine kinas	Multiple_Complex
TC0300011200.hg.1	13.39	11.87	2.87	1.15E-06	1.05E-05	TKT	transketolase	Multiple_Complex
TC1700010590.hg.1	6.66	7.61	-1.93	1.16E-06	1.06E-05	GSDMB	gasdermin B	Multiple_Complex
TC0300013908.hg.1	10.01	8.53	2.8	1.17E-06	1.07E-05	FXR1	fragile X mental retardation, autosomal homo	Multiple_Complex
TC2200008067.hg.1	5.69	6.87	-2.27	1.17E-06	1.07E-05	THAP7	THAP domain containing 7	Multiple_Complex
TC0X00007267.hg.1	4.68	5.86	-2.26	1.17E-06	1.07E-05	MAGED1	MAGE family member D1	Multiple_Complex
TC1600009200.hg.1	4.4	5.58	-2.27	1.18E-06	1.07E-05	TRAP1	TNF receptor-associated protein 1	Multiple_Complex
TC1300009218.hg.1	6.03	4.74	2.44	1.18E-06	1.07E-05	DACH1	dachshund family transcription factor 1	Multiple_Complex
TC1100013007.hg.1	10.23	9.51	1.65	1.19E-06	1.08E-05	ZFP91-CNT	ZFP91-CNTF readthrough (NMD candidate)	Multiple_Complex
TC1600010794.hg.1	8.11	7.11	2	1.19E-06	1.08E-05	AP1G1	adaptor-related protein complex 1, gamma 1 s	Multiple_Complex
TC0300006827.hg.1	6.26	7.6	-2.52	1.19E-06	1.08E-05	UBE2E2	ubiquitin conjugating enzyme E2E 2	Multiple_Complex
TC0200009299.hg.1	4.67	5.93	-2.4	1.20E-06	1.09E-05	IMP4	IMP4 homolog, U3 small nucleolar ribonucleo	Multiple_Complex
TC1400007354.hg.1	13.97	12.46	2.84	1.20E-06	1.09E-05	PPM1A	protein phosphatase, Mg2+/Mn2+ dependent	Multiple_Complex
TC0600007657.hg.1	8.55	11.07	-5.75	1.20E-06	1.09E-05	HLA-DQA1	major histocompatibility complex, class II, DQ	Multiple_Complex
TC2200009338.hg.1	5.58	6.52	-1.93	1.20E-06	1.09E-05	TBC1D10A	TBC1 domain family, member 10A	Multiple_Complex
TC2000007488.hg.1	8.45	9.57	-2.17	1.21E-06	1.10E-05	PIGT	phosphatidylinositol glycan anchor biosynthes	Multiple_Complex
TC0X00007127.hg.1	6.1	6.97	-1.84	1.21E-06	1.10E-05	RBM10	RNA binding motif protein 10	Multiple_Complex
TC1700012199.hg.1	4.77	6.1	-2.53	1.22E-06	1.11E-05	PIGL	phosphatidylinositol glycan anchor biosynthes	NonCoding
TC2100007935.hg.1	11.09	12.33	-2.36	1.23E-06	1.11E-05	SCAF4	SR-related CTD-associated factor 4	Multiple_Complex
TC0300013866.hg.1	6.28	7.43	-2.23	1.23E-06	1.11E-05	ALG1L2	ALG1, chitobiosyldiphosphodolichol beta-man	Multiple_Complex
TC0100008772.hg.1	8.42	7.56	1.82	1.23E-06	1.11E-05	RABGGTB	Zhang2013 ALT_ACCEPTOR, ALT_DONOR, cod	NonCoding
TC0900008902.hg.1	6.36	7.54	-2.28	1.25E-06	1.13E-05	PPP2R4	protein phosphatase 2A activator, regulatory s	Multiple_Complex
TC2200008654.hg.1	10.02	11.18	-2.24	1.25E-06	1.13E-05	MFNG	MFNG O-fucosylpeptide 3-beta-N-acetylgluco:	Multiple_Complex

TC1000006862.hg.1	6.65	4.62	4.09	1.26E-06	1.14E-05	OLAH	oleoyl-ACP hydrolase	Multiple_Complex
TC0600011483.hg.1	11.65	10.33	2.5	1.27E-06	1.15E-05	PBX2	pre-B-cell leukemia homeobox 2	Multiple_Complex
TC1400007201.hg.1	5.18	4.33	1.81	1.28E-06	1.15E-05	CDKN3	cyclin-dependent kinase inhibitor 3	Multiple_Complex
TC1700012191.hg.1	12.62	14.13	-2.84	1.28E-06	1.16E-05	CD68	CD68 molecule	Coding
TC1200007706.hg.1	7.46	8.35	-1.85	1.29E-06	1.16E-05	MFSD5	major facilitator superfamily domain containir	Multiple_Complex
TC0800011139.hg.1	7.88	8.8	-1.89	1.29E-06	1.16E-05	KIAA1429	KIAA1429	Multiple_Complex
TC0700009827.hg.1	11.08	12.09	-2.02	1.29E-06	1.16E-05	RBM33	RNA binding motif protein 33	Multiple_Complex
TC1000008575.hg.1	6.93	7.72	-1.73	1.30E-06	1.17E-05	ZDHHC16	zinc finger, DHHC-type containing 16	Multiple_Complex
TC2100006974.hg.1	10.05	11.04	-1.98	1.30E-06	1.17E-05	SON; MIR6	SON DNA binding protein; microRNA 6501	Multiple_Complex
TC1000010982.hg.1	6.86	5.26	3.01	1.30E-06	1.17E-05	ASCC1	Transcript Identified by AceView, Entrez Gene	Coding
TC0800011006.hg.1	5.35	4.17	2.28	1.31E-06	1.18E-05	REXO1L2P;	Homo sapiens REX1, RNA exonuclease 1 homo	Multiple_Complex
TC0100018180.hg.1	5.1	4.28	1.77	1.31E-06	1.18E-05	CLCNKA	chloride channel, voltage-sensitive Ka	Multiple_Complex
TC1200006701.hg.1	8.79	7.72	2.1	1.32E-06	1.18E-05	NECAP1	NECAP endocytosis associated 1	Multiple_Complex
TSUnmapped00000492.h	5.46	6.36	-1.88	1.32E-06	1.19E-05	KAT6B	K(lysine) acetyltransferase 6B	Coding
TC1200010518.hg.1	11.75	13.31	-2.94	1.33E-06	1.19E-05	SLC38A2	solute carrier family 38, member 2	Multiple_Complex
TC1700009402.hg.1	4.96	6.6	-3.14	1.33E-06	1.19E-05	SMYD4	SET and MYND domain containing 4	Multiple_Complex
TC2000007337.hg.1	8.97	7.94	2.04	1.34E-06	1.20E-05	PPP1R16B	Transcript Identified by AceView, Entrez Gene	Unassigned
TC1500009185.hg.1	9.47	8.36	2.15	1.35E-06	1.21E-05	TMEM87A	transmembrane protein 87A	Multiple_Complex
TC0100018260.hg.1	11.3	9.07	4.69	1.36E-06	1.22E-05	AMPD2	adenosine monophosphate deaminase 2	Multiple_Complex
TC1900011794.hg.1	8.08	9.41	-2.52	1.37E-06	1.23E-05	ZNF701; ZN	zinc finger protein 701; zinc finger protein 137	Multiple_Complex
TC1100011097.hg.1	4.78	6	-2.33	1.38E-06	1.24E-05	ZBTB3	zinc finger and BTB domain containing 3	Multiple_Complex
TC2200009266.hg.1	9.22	7.99	2.36	1.38E-06	1.24E-05	GTPBP1	GTP binding protein 1	Multiple_Complex
TC1700011418.hg.1	6.43	7.43	-2	1.39E-06	1.24E-05	LIMD2	LIM domain containing 2	Multiple_Complex
TC1200011820.hg.1	8.08	9.39	-2.48	1.39E-06	1.24E-05	PRDM4	PR domain containing 4	Multiple_Complex
TC0200010852.hg.1	6.15	7.03	-1.84	1.39E-06	1.25E-05	GMPPA	GDP-mannose pyrophosphorylase A	Multiple_Complex
TC0100011414.hg.1	14.66	12.23	5.38	1.39E-06	1.25E-05	CR1	complement component (3b/4b) receptor 1 (I	Multiple_Complex
TC0600010057.hg.1	6.41	5.59	1.76	1.40E-06	1.25E-05	ACAT2	acetyl-CoA acetyltransferase 2	Multiple_Complex
TC0X00008540.hg.1	4.32	5.91	-3.03	1.40E-06	1.25E-05	CD40LG	CD40 ligand	Multiple_Complex
TC0200016683.hg.1	5.58	6.56	-1.98	1.41E-06	1.25E-05	C2orf42	chromosome 2 open reading frame 42	Multiple_Complex
TC0900011496.hg.1	9.27	7.95	2.5	1.41E-06	1.25E-05	PSMB7	proteasome subunit beta 7	Multiple_Complex
TC0200016772.hg.1	5.62	6.95	-2.51	1.41E-06	1.26E-05	NHEJ1	nonhomologous end-joining factor 1	Multiple_Complex
TC0300013428.hg.1	13.32	12.33	1.99	1.42E-06	1.27E-05	TRA2B	transformer 2 beta homolog (Drosophila)	Multiple_Complex
TC0800009988.hg.1	5.59	7.1	-2.85	1.42E-06	1.27E-05	INTS9	integrator complex subunit 9	Multiple_Complex
TC0500009100.hg.1	7.68	6.9	1.71	1.43E-06	1.27E-05	SMIM3	small integral membrane protein 3	Multiple_Complex
TC0700013472.hg.1	14.12	10.73	10.48	1.43E-06	1.27E-05	MGAM	maltase-glucoamylase	Multiple_Complex
TC1700007720.hg.1	10.17	9.09	2.11	1.43E-06	1.27E-05	PSMB3	proteasome subunit beta 3	Multiple_Complex
TC1600008020.hg.1	10.17	9.15	2.02	1.44E-06	1.28E-05	USB1	U6 snRNA biogenesis 1	Multiple_Complex
TC2100008286.hg.1	10.51	9.47	2.06	1.44E-06	1.28E-05	U2AF1	U2 small nuclear RNA auxiliary factor 1	Multiple_Complex
TC0200006583.hg.1	5.14	6.22	-2.1	1.44E-06	1.28E-05	RNF144A	ring finger protein 144A	Multiple_Complex
TC0X00006623.hg.1	8.14	9.18	-2.05	1.45E-06	1.29E-05	PRPS2	phosphoribosyl pyrophosphate synthetase 2	Multiple_Complex
TC1200011599.hg.1	14.22	12.12	4.27	1.46E-06	1.30E-05	LTA4H	leukotriene A4 hydrolase	Multiple_Complex
TC2200009248.hg.1	9.33	7.31	4.03	1.47E-06	1.31E-05	KREMEN1	kringle containing transmembrane protein 1	Multiple_Complex
TC1200007053.hg.1	5.76	3.85	3.76	1.47E-06	1.31E-05	SPX	spexin hormone	Multiple_Complex
TC1900011061.hg.1	10.19	11.4	-2.31	1.47E-06	1.31E-05	CARD8	caspase recruitment domain family, member 8	Multiple_Complex
TC0100017107.hg.1	5.43	4.37	2.08	1.48E-06	1.31E-05	IL10	interleukin 10	Multiple_Complex
TC0X00006764.hg.1	6.4	7.24	-1.79	1.48E-06	1.31E-05	MBTPS2; Y	membrane bound transcription factor peptida	Multiple_Complex
TC0100014284.hg.1	8.28	9.93	-3.14	1.48E-06	1.31E-05	USP24	ubiquitin specific peptidase 24	Multiple_Complex
TC1600007829.hg.1	11.04	9.93	2.17	1.49E-06	1.32E-05	CYLD	cylindromatosis (turban tumor syndrome)	Multiple_Complex
TC1900011860.hg.1	6.13	7.38	-2.37	1.50E-06	1.32E-05	DNMT1	DNA (cytosine-5-)-methyltransferase 1	Multiple_Complex
TC1700010218.hg.1	10.95	9.31	3.12	1.50E-06	1.33E-05	FLOT2	flotillin 2	Multiple_Complex
TC1500008757.hg.1	6.87	8.16	-2.44	1.50E-06	1.33E-05	ATP10A	ATPase, class V, type 10A	Multiple_Complex
TC0100012534.hg.1	6.3	7.19	-1.85	1.52E-06	1.34E-05	PANK4	pantothenate kinase 4	Multiple_Complex
TC1100007366.hg.1	8.67	9.77	-2.14	1.52E-06	1.34E-05	TTC17	tetratricopeptide repeat domain 17	Multiple_Complex
TC1600008178.hg.1	5.37	6.39	-2.03	1.52E-06	1.35E-05	PSKH1	protein serine kinase H1	Multiple_Complex
TC0400006816.hg.1	5.67	4.82	1.8	1.53E-06	1.35E-05	USP17L26;	ubiquitin specific peptidase 17-like family mer	Coding
TC0500010511.hg.1	5.37	6.42	-2.07	1.53E-06	1.35E-05	C5orf42	chromosome 5 open reading frame 42	Multiple_Complex
TC0500008921.hg.1	10.62	11.68	-2.08	1.53E-06	1.35E-05	NDFIP1	Nedd4 family interacting protein 1	Multiple_Complex
TC1700007638.hg.1	10.64	9.46	2.27	1.54E-06	1.36E-05	GGNBP2	gametogenetin binding protein 2	Multiple_Complex
TC0800006738.hg.1	6.12	7.54	-2.67	1.55E-06	1.36E-05	MTMR9	myotubularin related protein 9	Multiple_Complex
TC0100007716.hg.1	6.54	7.48	-1.92	1.56E-06	1.37E-05	ZNF362	zinc finger protein 362	Multiple_Complex
TC0800008131.hg.1	5.72	4.81	1.88	1.56E-06	1.38E-05	REXO1L11F	REX1, RNA exonuclease 1 homolog-like 11, ps	Multiple_Complex
TC0100018552.hg.1	5.97	5.22	1.68	1.57E-06	1.38E-05	CHIT1	chitinase 1 (chitotriosidase)	NonCoding
TC0200011890.hg.1	6.42	5.11	2.48	1.57E-06	1.38E-05	LAPTM4A	Memczak2013 ALT_ACCEPTOR, ALT_DONOR,	NonCoding
TC0100018539.hg.1	5.28	6.39	-2.15	1.57E-06	1.38E-05	LHX4-AS1;	LHX4 antisense RNA 1; acyl-CoA binding doma	NonCoding
TC0600014198.hg.1	7.59	8.61	-2.02	1.58E-06	1.39E-05	TULP4	tubby like protein 4	Multiple_Complex
TC0600014362.hg.1	5.38	6.47	-2.13	1.59E-06	1.40E-05	TFB1M	transcription factor B1, mitochondrial	Multiple_Complex
TC1700007636.hg.1	4.62	5.57	-1.94	1.59E-06	1.40E-05	PIGW	phosphatidylinositol glycan anchor biosynthes	Coding
TC0500012498.hg.1	9.31	10.61	-2.47	1.60E-06	1.40E-05	ANXA6	annexin A6	Multiple_Complex
TC0100013696.hg.1	9.45	10.33	-1.84	1.60E-06	1.41E-05	PSMB2	proteasome subunit beta 2	Multiple_Complex
TC2000008755.hg.1	5.81	6.62	-1.75	1.61E-06	1.41E-05	ZNF337	zinc finger protein 337	Multiple_Complex

TSUnmapped00000546.h	7.29	8.19	-1.86	1.61E-06	1.41E-05	RCC2	regulator of chromosome condensation 2	Coding
TC1900011990.hg.1	12.12	13.89	-3.4	1.61E-06	1.42E-05	RPL18	ribosomal protein L18	Multiple_Complex
TC1600010500.hg.1	6	7.11	-2.16	1.64E-06	1.43E-05	GOT2	glutamic-oxaloacetic transaminase 2, mitocho	Multiple_Complex
TC1700010050.hg.1	10.38	12.15	-3.42	1.64E-06	1.44E-05	LGALS9B	lectin, galactoside-binding, soluble, 9B	Multiple_Complex
TC1900007908.hg.1	13.53	15.07	-2.91	1.64E-06	1.44E-05	CAPNS1	calpain, small subunit 1	Multiple_Complex
TC1700006653.hg.1	9.43	7.19	4.74	1.64E-06	1.44E-05	GP1BA	glycoprotein Ib (platelet), alpha polypeptide	Coding
TC1700011424.hg.1	9.42	10.32	-1.86	1.65E-06	1.44E-05	SMARCD2	SWI/SNF related, matrix associated, actin dep	Multiple_Complex
TC0400008007.hg.1	9.28	7.55	3.32	1.65E-06	1.45E-05	GPAT3	glycerol-3-phosphate acyltransferase 3	Multiple_Complex
TC0800010685.hg.1	5.82	8.2	-5.2	1.66E-06	1.45E-05	MYBL1	v-myb avian myeloblastosis viral oncogene ho	Multiple_Complex
TC0700008147.hg.1	9.16	10.71	-2.93	1.66E-06	1.45E-05	RSBN1L	round spermatid basic protein 1-like	Multiple_Complex
TC0800008321.hg.1	4.94	3.54	2.63	1.67E-06	1.46E-05	LAPTM4B	lysosomal protein transmembrane 4 beta	Multiple_Complex
TC1200009524.hg.1	5.5	6.59	-2.12	1.69E-06	1.47E-05	ZNF84	zinc finger protein 84	Multiple_Complex
TC1900008993.hg.1	4.58	5.55	-1.96	1.71E-06	1.49E-05	ZNF304	zinc finger protein 304	Multiple_Complex
TC0100006861.hg.1	6.33	7.1	-1.71	1.71E-06	1.49E-05	FBXO44	F-box protein 44	Multiple_Complex
TC0800008126.hg.1	9.19	8	2.28	1.74E-06	1.51E-05	CA2	carbonic anhydrase II	Multiple_Complex
TC0100018398.hg.1	7.7	8.63	-1.91	1.74E-06	1.51E-05	TNFRSF25	tumor necrosis factor receptor superfamily, m	Multiple_Complex
TSUnmapped00000171.h	6.77	5.46	2.49	1.74E-06	1.51E-05	TRAPPC4	trafficking protein particle complex 4	Coding
TC0300008290.hg.1	8.93	10.81	-3.7	1.74E-06	1.51E-05	TRAT1	T cell receptor associated transmembrane ada	Multiple_Complex
TC2200008781.hg.1	9.64	10.75	-2.17	1.74E-06	1.52E-05	MKL1	megakaryoblastic leukemia (translocation) 1	Multiple_Complex
TC0300013828.hg.1	9.48	10.47	-1.99	1.75E-06	1.53E-05	RBM6	RNA binding motif protein 6	Multiple_Complex
TC0100010532.hg.1	4.97	6.59	-3.08	1.76E-06	1.53E-05	XCL1	chemokine (C motif) ligand 1	Coding
TC1900011172.hg.1	10.52	11.95	-2.69	1.76E-06	1.53E-05	VRK3	vaccinia related kinase 3	Multiple_Complex
TC0700011054.hg.1	6.54	4.93	3.04	1.76E-06	1.53E-05	GRB10	growth factor receptor bound protein 10	Multiple_Complex
TC0500008156.hg.1	7.36	10.83	-11.1	1.79E-06	1.56E-05	ERAP2	endoplasmic reticulum aminopeptidase 2	Multiple_Complex
TC0X00006658.hg.1	6.56	7.96	-2.64	1.82E-06	1.58E-05	OFD1	oral-facial-digital syndrome 1	Multiple_Complex
TC0400009284.hg.1	10.36	8.94	2.66	1.82E-06	1.58E-05	CLCN3	chloride channel, voltage-sensitive 3	Multiple_Complex
TC2000006628.hg.1	5.31	6.97	-3.16	1.83E-06	1.59E-05	MCM8	minichromosome maintenance 8 homologous	Multiple_Complex
TC1700011181.hg.1	10.7	8.53	4.48	1.84E-06	1.60E-05	MMD	monocyte to macrophage differentiation-asso	Multiple_Complex
TC0100017488.hg.1	13.24	11.98	2.38	1.85E-06	1.60E-05	LBR	lamin B receptor	Multiple_Complex
TC1300008250.hg.1	5.12	3.97	2.22	1.85E-06	1.60E-05	GJB6	gap junction protein beta 6	Multiple_Complex
TC1400007087.hg.1	6.73	7.82	-2.13	1.85E-06	1.60E-05	MGAT2	mannosyl (alpha-1,6-)-glycoprotein beta-1,2-N	Coding
TC1100009408.hg.1	6.23	7.39	-2.24	1.87E-06	1.62E-05	SLC37A2	solute carrier family 37 (glucose-6-phosphate	Multiple_Complex
TC0700007374.hg.1	8.55	7.7	1.8	1.87E-06	1.62E-05	YKT6	YKT6 v-SNARE homolog (S. cerevisiae)	Multiple_Complex
TC1400009714.hg.1	9.64	10.78	-2.19	1.88E-06	1.62E-05	TMED10	transmembrane p24 trafficking protein 10	Multiple_Complex
TC0700007479.hg.1	6.72	4.9	3.53	1.88E-06	1.62E-05	ABCA13	ATP binding cassette subfamily A member 13	Multiple_Complex
TC2100007668.hg.1	9.61	10.65	-2.06	1.89E-06	1.63E-05	C21orf91; (	chromosome 21 open reading frame 91; C21o	Multiple_Complex
TC0100007091.hg.1	9.37	8.19	2.27	1.89E-06	1.63E-05	CROCC	Zhang2013 ALT_ACCEPTOR, ALT_DONOR, cod	NonCoding
TC0700007098.hg.1	9.16	10.07	-1.89	1.89E-06	1.63E-05	GARS	glycyl-tRNA synthetase	Multiple_Complex
TC0X00010426.hg.1	8.1	6.9	2.3	1.90E-06	1.64E-05	MORF4L2	mortality factor 4 like 2	Multiple_Complex
TC1000010115.hg.1	11.35	10.4	1.94	1.90E-06	1.64E-05	YME1L1	YME1-like 1 ATPase	Multiple_Complex
TC0800011259.hg.1	5.68	6.74	-2.07	1.90E-06	1.64E-05	ANKRD46	ankyrin repeat domain 46	Multiple_Complex
TC0700007633.hg.1	7.75	8.59	-1.8	1.90E-06	1.64E-05	SUMF2	sulfatase modifying factor 2	Multiple_Complex
TC1600008328.hg.1	5.86	6.69	-1.78	1.91E-06	1.65E-05	DHODH	dihydroorotate dehydrogenase (quinone)	Multiple_Complex
TC1700006773.hg.1	7.67	6.26	2.66	1.92E-06	1.65E-05	TMEM88	transmembrane protein 88	Coding
TC0800010880.hg.1	4.74	5.68	-1.93	1.93E-06	1.66E-05	IL7	interleukin 7	Multiple_Complex
TC0200016496.hg.1	5.74	6.81	-2.09	1.93E-06	1.67E-05	CNNM3	cyclin and CBS domain divalent metal cation t	Multiple_Complex
TC1800006437.hg.1	8.45	9.46	-2.01	1.94E-06	1.67E-05	USP14	ubiquitin specific peptidase 14 (tRNA-guanine	Multiple_Complex
TC0700013618.hg.1	4.64	5.74	-2.15	1.95E-06	1.68E-05	MGC27345	uncharacterized protein MGC27345	Multiple_Complex
TC0900011634.hg.1	4.66	5.59	-1.91	1.95E-06	1.68E-05	TRUB2	TruB pseudouridine (psi) synthase family mem	Multiple_Complex
TC0200015729.hg.1	6.04	7.15	-2.16	1.95E-06	1.68E-05	USP37	ubiquitin specific peptidase 37	Multiple_Complex
TC1100010058.hg.1	10.43	9.06	2.59	1.96E-06	1.68E-05	DENND5A	DENN/MADD domain containing 5A	Multiple_Complex
TC1100010207.hg.1	5.25	6.44	-2.28	1.96E-06	1.69E-05	SOX6; MIR6	SRY box 6; microRNA 6073	Multiple_Complex
TC0200013137.hg.1	5.04	6.04	-1.99	1.96E-06	1.69E-05	GCFC2	GC-rich sequence DNA-binding factor 2	Multiple_Complex
TSUnmapped00000597.h	9.53	10.67	-2.21	1.97E-06	1.69E-05	PSMC4	proteasome 26S subunit, ATPase 4 [Source:HG	Coding
TC0300009856.hg.1	5.94	5.18	1.69	1.97E-06	1.69E-05	IL1RAP	interleukin 1 receptor accessory protein	NonCoding
TC1300006690.hg.1	9.16	10.43	-2.4	1.97E-06	1.69E-05	POLR1D	polymerase (RNA) I polypeptide D	Multiple_Complex
TC1200007295.hg.1	6.14	7.18	-2.05	1.98E-06	1.70E-05	ALG10	ALG10, alpha-1,2-glucosyltransferase	Multiple_Complex
TC0X00010804.hg.1	5.33	6.74	-2.66	1.99E-06	1.70E-05	ZNF280C	zinc finger protein 280C	Multiple_Complex
TC0300012857.hg.1	8.31	9.39	-2.11	1.99E-06	1.71E-05	DHX36	DEAH (Asp-Glu-Ala-His) box polypeptide 36	Multiple_Complex
TC1500010724.hg.1	8.19	9.29	-2.14	2.02E-06	1.73E-05	GANC	glucosidase, alpha; neutral C	Multiple_Complex
TC0400008105.hg.1	8.15	9.85	-3.25	2.02E-06	1.73E-05	HERC6	HECT and RLD domain containing E3 ubiquitin	Multiple_Complex
TC1300008171.hg.1	7.96	9.54	-2.99	2.02E-06	1.73E-05	CDC16	cell division cycle 16	Multiple_Complex
TC1900008925.hg.1	6.54	7.67	-2.19	2.02E-06	1.73E-05	U2AF2	Memczak2013 ALT_ACCEPTOR, ALT_DONOR,	NonCoding
TC0200008569.hg.1	7.56	8.57	-2.01	2.03E-06	1.73E-05	INPP4A	inositol polyphosphate-4-phosphatase type I A	Multiple_Complex
TC0100014774.hg.1	7.66	6.58	2.11	2.05E-06	1.75E-05	C1orf52	chromosome 1 open reading frame 52	Multiple_Complex
TC0600014267.hg.1	10.89	12.37	-2.77	2.05E-06	1.75E-05	ATF6B	activating transcription factor 6 beta	Multiple_Complex
TC0700006727.hg.1	6.73	7.68	-1.94	2.05E-06	1.75E-05	TMEM106B	transmembrane protein 106B	Multiple_Complex
TC1000010497.hg.1	10.14	12.33	-4.57	2.06E-06	1.76E-05	8-Mar	membrane associated ring finger 8	Multiple_Complex
TC2200007816.hg.1	4.37	4.96	-1.51	2.06E-06	1.76E-05	KLHDC7B	kelch domain containing 7B	Coding
TC0400008284.hg.1	5.37	4.22	2.22	2.06E-06	1.76E-05	UBE2D3	Memczak2013 ANTISENSE, coding, INTERNAL,	NonCoding

TC0100008807.hg.1	7.13	5.69	2.71	2.07E-06	1.76E-05	NEXN	nexilin (F actin binding protein)	Multiple_Complex
TC1700010247.hg.1	5.16	5.96	-1.73	2.07E-06	1.77E-05	ABHD15	abhydrolase domain containing 15	Coding
TC0500011150.hg.1	6.23	7.25	-2.02	2.08E-06	1.77E-05	GFM2	G elongation factor, mitochondrial 2	Multiple_Complex
TC0100016470.hg.1	7.1	7.86	-1.7	2.09E-06	1.78E-05	MRPS14	mitochondrial ribosomal protein S14	Multiple_Complex
TC1600007425.hg.1	5.91	6.79	-1.84	2.10E-06	1.78E-05	KIF22	kinesin family member 22	Multiple_Complex
TC0300007136.hg.1	10.93	10.2	1.65	2.11E-06	1.79E-05	CTNNB1	catenin (cadherin-associated protein), beta 1	Multiple_Complex
TC0400007264.hg.1	12.63	13.63	-2	2.11E-06	1.79E-05	KLF3	Kruppel-like factor 3 (basic)	Multiple_Complex
TC2200009196.hg.1	5.33	6.23	-1.86	2.11E-06	1.80E-05	LZTR1	leucine-zipper-like transcription regulator 1	Multiple_Complex
TC1800009268.hg.1	10.47	7.63	7.18	2.11E-06	1.80E-05	DSC2	desmocollin 2	Multiple_Complex
TC1900011752.hg.1	5.07	6.34	-2.42	2.13E-06	1.81E-05	ZNF223	zinc finger protein 223	Multiple_Complex
TC0600011127.hg.1	7.08	5.99	2.13	2.14E-06	1.82E-05	HIST1H1C	histone cluster 1, H1c	Multiple_Complex
TC0100009561.hg.1	5.25	6.08	-1.77	2.16E-06	1.83E-05	TTF2	transcription termination factor, RNA polymer	Multiple_Complex
TC1100010185.hg.1	5.36	6.61	-2.39	2.16E-06	1.83E-05	CYP2R1	cytochrome P450, family 2, subfamily R, polyp	Multiple_Complex
TC1700009954.hg.1	7.34	8.51	-2.25	2.16E-06	1.83E-05	TOM1L2	target of myb1 like 2 membrane trafficking pr	Multiple_Complex
TC1100008612.hg.1	14.46	16.53	-4.19	2.16E-06	1.83E-05	RPS28	ribosomal protein S28	Multiple_Complex
TC1900012044.hg.1	7.27	6.12	2.22	2.18E-06	1.85E-05	GP6	glycoprotein VI (platelet)	Multiple_Complex
TC0500007048.hg.1	4.92	5.82	-1.87	2.19E-06	1.86E-05	C5orf22	chromosome 5 open reading frame 22	Multiple_Complex
TC0200008501.hg.1	6.45	7.69	-2.36	2.21E-06	1.87E-05	ITPRIPL1	inositol 1,4,5-trisphosphate receptor interacti	Coding
TSUnmapped00000128.h	5.02	6.23	-2.3	2.22E-06	1.88E-05	ZNF852	zinc finger protein 852	NonCoding
TC1400009649.hg.1	13.45	11.83	3.07	2.23E-06	1.89E-05	NUMB	numb homolog (Drosophila)	Multiple_Complex
TC1200006604.hg.1	10.82	8.56	4.78	2.23E-06	1.89E-05	CD9	CD9 molecule	Multiple_Complex
TC0700008926.hg.1	8.33	6.74	3.02	2.24E-06	1.90E-05	ING3	inhibitor of growth family member 3	Multiple_Complex
TC0100009939.hg.1	7.61	8.62	-2.02	2.24E-06	1.90E-05	GABPB2	GA binding protein transcription factor, beta s	Multiple_Complex
TC1700008028.hg.1	8.03	8.66	-1.55	2.24E-06	1.90E-05	NMT1	N-myristoyltransferase 1	Multiple_Complex
TC0200015214.hg.1	4.47	6.17	-3.23	2.25E-06	1.90E-05	OSGEPL1	O-sialoglycoprotein endopeptidase-like 1	Multiple_Complex
TC1100012019.hg.1	7.64	8.68	-2.05	2.25E-06	1.90E-05	SESN3	sestrin 3	Multiple_Complex
TC0200010838.hg.1	5.59	4.82	1.7	2.26E-06	1.91E-05	STK16	serine/threonine kinase 16	Multiple_Complex
TC0100009305.hg.1	4.81	5.59	-1.72	2.27E-06	1.92E-05	PRMT6	protein arginine methyltransferase 6	Coding
TC0300008173.hg.1	12	11.14	1.81	2.28E-06	1.92E-05	PCNP	PEST proteolytic signal containing nuclear pro	Multiple_Complex
TC2200006601.hg.1	6.48	5.11	2.58	2.28E-06	1.92E-05	MRPL40	mitochondrial ribosomal protein L40	Multiple_Complex
TC0900011659.hg.1	4.17	5.17	-2	2.28E-06	1.92E-05	C9orf114	chromosome 9 open reading frame 114	Multiple_Complex
TC0500013361.hg.1	8.85	8.04	1.75	2.31E-06	1.94E-05	SAR1B	secretion associated, Ras related GTPase 1B	Multiple_Complex
TC0800011061.hg.1	9.34	8.01	2.51	2.32E-06	1.95E-05	NBN	nibrin	Multiple_Complex
TSUnmapped00000378.h	10.5	8.06	5.42	2.32E-06	1.96E-05	PADI4	peptidyl arginine deiminase, type IV	Coding
TC0100015947.hg.1	18.42	17.51	1.89	2.32E-06	1.96E-05	THBS3	Salzman2013 ALT_ACCEPTOR, ALT_DONOR, c	NonCoding
TC1000007272.hg.1	6.92	5.63	2.45	2.32E-06	1.96E-05	CREM	cAMP responsive element modulator	Multiple_Complex
TC1000012584.hg.1	9.44	7.81	3.08	2.33E-06	1.96E-05	ERLIN1	ER lipid raft associated 1	Multiple_Complex
TC0900009846.hg.1	8.53	7.72	1.76	2.33E-06	1.96E-05	B4GALT1	UDP-Gal:betaGlcNAc beta 1,4- galactosyltrans	Multiple_Complex
TC1400010778.hg.1	5.36	6.54	-2.27	2.33E-06	1.96E-05	TRIP11	thyroid hormone receptor interactor 11	Multiple_Complex
TC1900006978.hg.1	6.11	5.34	1.71	2.33E-06	1.96E-05	ICAM4	intercellular adhesion molecule 4 (Landsteine	Multiple_Complex
TC1100008398.hg.1	5.16	6.14	-1.98	2.35E-06	1.97E-05	PAAF1	proteasomal ATPase-associated factor 1	Multiple_Complex
TC1700010811.hg.1	8.82	7.54	2.42	2.36E-06	1.98E-05	HDAC5	histone deacetylase 5	Multiple_Complex
TC0800010285.hg.1	6.55	7.56	-2.02	2.38E-06	1.99E-05	SLC20A2	solute carrier family 20 (phosphate transporte	Multiple_Complex
TC1800007523.hg.1	8.57	7.22	2.54	2.39E-06	2.01E-05	PHLPP1	PH domain and leucine rich repeat protein ph	Multiple_Complex
TC0200006665.hg.1	5.54	6.78	-2.36	2.41E-06	2.02E-05	TAF1B	TATA box binding protein (TBP)-associated fac	Multiple_Complex
TC0100009858.hg.1	7.83	9.04	-2.31	2.43E-06	2.03E-05	PPIAL4C	peptidylprolyl isomerase A (cyclophilin A)-like	Coding
TC2100007467.hg.1	7.24	8.29	-2.06	2.43E-06	2.04E-05	DIP2A	disco-interacting protein 2 homolog A	Multiple_Complex
TC1700009730.hg.1	6.47	5.49	1.96	2.44E-06	2.04E-05	RCVRN	recoverin	Multiple_Complex
TC0300010949.hg.1	15.44	9.76	51.45	2.45E-06	2.05E-05	LTF	lactotransferrin	Multiple_Complex
TC0400010995.hg.1	8.76	9.83	-2.1	2.48E-06	2.08E-05	ANKRD17	ankyrin repeat domain 17	Multiple_Complex
TC1600008991.hg.1	6.38	7.44	-2.09	2.50E-06	2.09E-05	LMF1	lipase maturation factor 1	Multiple_Complex
TC0800008062.hg.1	7.69	6.68	2.01	2.51E-06	2.10E-05	ZBTB10	zinc finger and BTB domain containing 10	Coding
TC0100018388.hg.1	8.68	9.7	-2.02	2.52E-06	2.11E-05	SLC35E2B	solute carrier family 35, member E2B	Multiple_Complex
TC0600011145.hg.1	10.59	9.19	2.64	2.54E-06	2.12E-05	HIST1H4H	histone cluster 1, H4h	Coding
TC1200012735.hg.1	8.19	7.15	2.06	2.58E-06	2.15E-05	ZNF26	zinc finger protein 26	NonCoding
TC0500011751.hg.1	10.06	9.25	1.75	2.59E-06	2.16E-05	FEM1C	fem-1 homolog c (C. elegans)	Multiple_Complex
TC1200006946.hg.1	6.05	5.26	1.73	2.59E-06	2.16E-05	H2AFJ	H2A histone family, member J	Multiple_Complex
TC1900010748.hg.1	7.68	8.76	-2.11	2.59E-06	2.16E-05	ATP5SL	ATP5S-like	Multiple_Complex
TC1000008088.hg.1	9.83	8.63	2.3	2.61E-06	2.17E-05	SAMD8	sterile alpha motif domain containing 8	Multiple_Complex
TC0100016431.hg.1	7.69	6.28	2.65	2.63E-06	2.19E-05	TNFSF4	tumor necrosis factor (ligand) superfamily, me	Multiple_Complex
TC0100010193.hg.1	8.73	9.95	-2.34	2.63E-06	2.19E-05	PRCC	papillary renal cell carcinoma (translocation-a	Multiple_Complex
TC0800007949.hg.1	8.99	10.06	-2.09	2.65E-06	2.21E-05	TERF1	telomeric repeat binding factor (NIMA-interac	Multiple_Complex
TC1100011739.hg.1	7.65	9	-2.55	2.66E-06	2.21E-05	ALG8	ALG8, alpha-1,3-glucosyltransferase	Multiple_Complex
TC0200012418.hg.1	7.82	8.85	-2.05	2.70E-06	2.25E-05	LRPPRC	leucine-rich pentatricopeptide repeat containi	Multiple_Complex
TC1100011222.hg.1	5.65	6.51	-1.81	2.70E-06	2.25E-05	SLC25A45	solute carrier family 25, member 45	Multiple_Complex
TC0600009611.hg.1	9.75	8.66	2.12	2.76E-06	2.29E-05	HEBP2	heme binding protein 2	Multiple_Complex
TC1500009298.hg.1	5.04	5.92	-1.84	2.76E-06	2.30E-05	SLC30A4	solute carrier family 30 (zinc transporter), me	Multiple_Complex
TC0800008145.hg.1	8.67	9.68	-2.02	2.79E-06	2.32E-05	WWP1	WW domain containing E3 ubiquitin protein li	Multiple_Complex
TC0200011919.hg.1	5.98	7.21	-2.35	2.79E-06	2.32E-05	LDAH	lipid droplet associated hydrolase	Multiple_Complex
TC0100008797.hg.1	5.5	7.01	-2.86	2.80E-06	2.32E-05	AK5	adenylate kinase 5	Multiple_Complex

TC2100008562.hg.1	12.34	13.52	-2.27	2.81E-06	2.33E-05	RUNX1	runt-related transcription factor 1	Multiple_Complex
TC2200008370.hg.1	9.17	10.04	-1.83	2.82E-06	2.34E-05	XBP1	X-box binding protein 1	Multiple_Complex
TC1700010814.hg.1	6.81	8.33	-2.86	2.83E-06	2.34E-05	ASB16-AS1	ASB16 antisense RNA 1	Multiple_Complex
TC0100017241.hg.1	5.3	6.08	-1.72	2.84E-06	2.35E-05	TMEM206	transmembrane protein 206	Multiple_Complex
TC1600010175.hg.1	6.97	5.67	2.47	2.84E-06	2.35E-05	C16orf87	chromosome 16 open reading frame 87	Multiple_Complex
TC0400010626.hg.1	8.19	9.18	-1.98	2.85E-06	2.36E-05	FRYL	FRY like transcription coactivator	Multiple_Complex
TC0100011384.hg.1	11.86	10.52	2.53	2.85E-06	2.36E-05	MAPKAPK2	mitogen-activated protein kinase-activated pr	Multiple_Complex
TC1200010984.hg.1	9.86	11.16	-2.47	2.89E-06	2.39E-05	CTDSP2	CTD small phosphatase 2	Multiple_Complex
TC1900008279.hg.1	10.34	8.89	2.74	2.90E-06	2.40E-05	BCL3; MIR8	B-cell CLL/lymphoma 3; microRNA 8085	Multiple_Complex
TC0500013217.hg.1	6.63	7.53	-1.86	2.91E-06	2.41E-05	COMMD10	COMM domain containing 10	Multiple_Complex
TC2200009282.hg.1	6.78	7.83	-2.08	2.93E-06	2.42E-05	SAMM50	SAMM50 sorting and assembly machinery cor	Multiple_Complex
TC0100016786.hg.1	9.93	11.07	-2.2	2.93E-06	2.42E-05	UCHL5	ubiquitin C-terminal hydrolase L5	Multiple_Complex
TC0500012486.hg.1	11.47	10.55	1.9	2.94E-06	2.43E-05	DCTN4	dynactin 4 (p62)	Multiple_Complex
TC0300013796.hg.1	6.2	6.98	-1.72	2.96E-06	2.45E-05	OXNAD1	oxidoreductase NAD-binding domain containi	NonCoding
TC1000010249.hg.1	11.75	11.06	1.6	2.97E-06	2.45E-05	KIF5B	kinesin family member 5B	Multiple_Complex
TC1200009892.hg.1	6.63	5.34	2.45	2.98E-06	2.46E-05	OLR1	oxidized low density lipoprotein (lectin-like) re	Multiple_Complex
TC0400008388.hg.1	6.74	7.94	-2.3	2.98E-06	2.46E-05	GAR1	GAR1 homolog, ribonucleoprotein	Multiple_Complex
TC0X00011173.hg.1	10.65	12.07	-2.67	3.00E-06	2.47E-05	IDH3G	isocitrate dehydrogenase 3 (NAD+) gamma	Multiple_Complex
TC0200012429.hg.1	8.57	5.96	6.11	3.02E-06	2.49E-05	PPM1B	Memczak2013 ANTISENSE, coding, INTERNAL,	NonCoding
TSUnmapped00000275.h	5.24	4.43	1.75	3.03E-06	2.50E-05	NDUFA6	NADH dehydrogenase (ubiquinone) 1 alpha su	Coding
TC1900007844.hg.1	4.36	5.29	-1.9	3.03E-06	2.50E-05	LSR	lipolysis stimulated lipoprotein receptor	Multiple_Complex
TC1600010475.hg.1	8.09	9.35	-2.39	3.07E-06	2.53E-05	CFAP20	cilia and flagella associated protein 20	Multiple_Complex
TC0200014893.hg.1	10.34	9.12	2.33	3.08E-06	2.53E-05	TLK1	tousled-like kinase 1	Multiple_Complex
TC0300007055.hg.1	8.52	6.21	4.96	3.08E-06	2.53E-05	CTDSPL	CTD small phosphatase like	Multiple_Complex
TC2000008852.hg.1	9.69	10.72	-2.05	3.12E-06	2.56E-05	NOL4L	nucleolar protein 4-like	Multiple_Complex
TC0100007634.hg.1	7.53	5.92	3.04	3.18E-06	2.61E-05	ZCCHC17	zinc finger, CCHC domain containing 17	Multiple_Complex
TC1800006589.hg.1	6.34	8.24	-3.72	3.18E-06	2.61E-05	PTPRM	protein tyrosine phosphatase, receptor type, I	Multiple_Complex
TC1000007396.hg.1	5.3	6.54	-2.36	3.18E-06	2.61E-05	BMS1	BMS1 ribosome biogenesis factor	Multiple_Complex
TC0800011372.hg.1	8.15	9.23	-2.11	3.20E-06	2.62E-05	SLC25A32	solute carrier family 25 (mitochondrial folate c	Multiple_Complex
TSUnmapped00000250.h	4.85	4.15	1.62	3.20E-06	2.63E-05	ZDHHC3	zinc finger, DHHC-type containing 3	NonCoding
TC1300008819.hg.1	16.74	13.74	8	3.20E-06	2.63E-05	TPT1; SNOI	tumor protein, translationally-controlled 1; sm	Multiple_Complex
TC1200008921.hg.1	7.14	12.13	-31.69	3.23E-06	2.65E-05	OAS2	2-5-oligoadenylate synthetase 2	Multiple_Complex
TC1200009913.hg.1	12.14	15.27	-8.77	3.24E-06	2.66E-05	YBX3	Y box binding protein 3	Multiple_Complex
TSUnmapped00000471.h	6.51	5.57	1.93	3.25E-06	2.66E-05	TRAPPC4	trafficking protein particle complex 4	Coding
TC0100007765.hg.1	7.35	8.35	-2.01	3.26E-06	2.67E-05	ZMYM4	zinc finger, MYM-type 4	Multiple_Complex
TC2000009198.hg.1	4.27	5.17	-1.87	3.26E-06	2.67E-05	ADA	adenosine deaminase	Multiple_Complex
TC1700011731.hg.1	9.64	8.28	2.57	3.27E-06	2.68E-05	ATP5H	ATP synthase, H+ transporting, mitochondrial	Multiple_Complex
TC0300011997.hg.1	5.53	6.59	-2.09	3.27E-06	2.68E-05	BTLA	B and T lymphocyte associated	Multiple_Complex
TC1300009639.hg.1	6.48	7.36	-1.84	3.28E-06	2.68E-05	TMTC4	transmembrane and tetratricopeptide repeat	Multiple_Complex
TC0100008564.hg.1	6.39	7.28	-1.85	3.29E-06	2.69E-05	ATG4C	autophagy related 4C, cysteine peptidase	Multiple_Complex
TC1900011909.hg.1	9.98	11.54	-2.94	3.29E-06	2.69E-05	COPE	coatomer protein complex subunit epsilon	Multiple_Complex
TC0200007115.hg.1	7.68	8.81	-2.19	3.30E-06	2.69E-05	CLIP4	CAP-GLY domain containing linker protein fan	Multiple_Complex
TC0300012027.hg.1	10.76	9.75	2	3.32E-06	2.71E-05	USF3	upstream transcription factor family member	Multiple_Complex
TC0100010244.hg.1	12.12	15.14	-8.08	3.33E-06	2.72E-05	IFI16	interferon, gamma-inducible protein 16	Multiple_Complex
TC0300010413.hg.1	8.98	7.97	2.01	3.33E-06	2.72E-05	ANKRD28	ankyrin repeat domain 28	Multiple_Complex
TC1900009176.hg.1	7.78	9.08	-2.47	3.34E-06	2.72E-05	TCF3	transcription factor 3	Multiple_Complex
TC1700009495.hg.1	9.26	7.78	2.8	3.36E-06	2.74E-05	P2RX1	purinergic receptor P2X, ligand gated ion char	Multiple_Complex
TC0200012404.hg.1	14.32	15.44	-2.19	3.40E-06	2.77E-05	ZFP36L2	ZFP36 ring finger protein-like 2	Multiple_Complex
TC0600007664.hg.1	6.52	7.78	-2.4	3.40E-06	2.77E-05	HLA-DQA2	major histocompatibility complex, class II, DQ	Coding
TC0800011007.hg.1	5.31	4.21	2.15	3.40E-06	2.77E-05	REXO1L4P	REX1, RNA exonuclease 1 homolog-like 4, pseu	Multiple_Complex
TC0300009224.hg.1	5.2	4.37	1.77	3.41E-06	2.77E-05	P2RY1	purinergic receptor P2Y, G-protein coupled, 1	Multiple_Complex
TC0500008582.hg.1	7.43	8.21	-1.71	3.42E-06	2.78E-05	ISOC1	isochorismatase domain containing 1	Multiple_Complex
TC1900011318.hg.1	5.52	6.3	-1.72	3.45E-06	2.80E-05	ZNF888	zinc finger protein 888	Multiple_Complex
TC0200016787.hg.1	7.49	8.31	-1.76	3.45E-06	2.81E-05	NDUFA10	NADH dehydrogenase (ubiquinone) 1 alpha su	Multiple_Complex
TC0400010770.hg.1	6.15	7.13	-1.97	3.46E-06	2.81E-05	PPAT	phosphoribosyl pyrophosphate amidotransfer	Multiple_Complex
TC1200009740.hg.1	6.75	7.78	-2.03	3.49E-06	2.84E-05	IFFO1	intermediate filament family orphan 1	Multiple_Complex
TC0500009678.hg.1	5.58	6.59	-2.02	3.50E-06	2.84E-05	ZNF354C	zinc finger protein 354C	Coding
TC0100010661.hg.1	5.72	6.79	-2.1	3.51E-06	2.85E-05	DARS2	aspartyl-tRNA synthetase 2, mitochondrial	Multiple_Complex
TC1000011553.hg.1	6.14	7.33	-2.28	3.52E-06	2.85E-05	MMS19	MMS19 homolog, cytosolic iron-sulfur assemb	Multiple_Complex
TC0900011423.hg.1	4.85	5.96	-2.16	3.52E-06	2.86E-05	TTLL11	tubulin tyrosine ligase-like family member 11	Multiple_Complex
TC1900011627.hg.1	3.75	4.82	-2.1	3.54E-06	2.87E-05	ZNF132	zinc finger protein 132	Multiple_Complex
TC1400009834.hg.1	8.03	9.34	-2.48	3.54E-06	2.87E-05	GTF2A1	general transcription factor IIA 1	Multiple_Complex
TC1700006774.hg.1	6.37	5.38	1.99	3.55E-06	2.87E-05	CYB5D1	cytochrome b5 domain containing 1	Multiple_Complex
TC0300013609.hg.1	7.64	8.65	-2	3.57E-06	2.89E-05	LSG1	large 60S subunit nuclear export GTPase 1	Multiple_Complex
TC2000006899.hg.1	11.3	10.45	1.8	3.57E-06	2.89E-05	XRN2	5-3 exoribonuclease 2	Multiple_Complex
TC0100018568.hg.1	11.02	9.64	2.59	3.59E-06	2.91E-05	ARID4B	AT rich interactive domain 4B (RBP1-like)	Multiple_Complex
TC1400008749.hg.1	7.3	8.46	-2.24	3.61E-06	2.92E-05	RABGGTA	Rab geranylgeranyltransferase, alpha subunit	Multiple_Complex
TC0200010582.hg.1	4.7	6	-2.46	3.62E-06	2.93E-05	FASTKD2	FAST kinase domains 2	Multiple_Complex
TC1200010795.hg.1	4.13	5.04	-1.88	3.62E-06	2.93E-05	RARG	retinoic acid receptor, gamma	Multiple_Complex
TC0300013019.hg.1	4.59	3.55	2.05	3.62E-06	2.93E-05	SI	sucrase-isomaltase (alpha-glucosidase)	Multiple_Complex

TC0300013270.hg.1	7.4	6.34	2.09	3.63E-06	2.93E-05	MRPL47	mitochondrial ribosomal protein L47	Multiple_Complex
TC0100008103.hg.1	11.48	9.58	3.72	3.64E-06	2.94E-05	RPS8; SNOI	ribosomal protein S8; small nucleolar RNA, C/I	Multiple_Complex
TC0300007194.hg.1	9.02	7.64	2.59	3.64E-06	2.94E-05	ABHD5	abhydrolase domain containing 5	Multiple_Complex
TC0900011803.hg.1	10.45	11.24	-1.74	3.64E-06	2.94E-05	SETX	senataxin	Multiple_Complex
TC1800006635.hg.1	8.59	7.6	1.99	3.67E-06	2.96E-05	TWSG1	twisted gastrulation BMP signaling modulator	Multiple_Complex
TC1000008529.hg.1	6.04	7.11	-2.1	3.69E-06	2.98E-05	CCNJ	cyclin J	Multiple_Complex
TC0400007556.hg.1	7.19	7.92	-1.65	3.70E-06	2.98E-05	SRD5A3	steroid 5 alpha-reductase 3	Multiple_Complex
TC0100018562.hg.1	8.88	9.85	-1.96	3.73E-06	3.00E-05	AIDA	axin interactor, dorsalization associated	Multiple_Complex
TC0200007261.hg.1	14.21	11.9	4.96	3.75E-06	3.02E-05	QPCT	glutaminyl-peptide cyclotransferase	Multiple_Complex
TC1400009065.hg.1	7.49	6.32	2.25	3.75E-06	3.02E-05	FKBP3	FK506 binding protein 3	Multiple_Complex
TC1800008965.hg.1	8.29	9.35	-2.08	3.77E-06	3.03E-05	TMX3	thioredoxin-related transmembrane protein 3	Multiple_Complex
TC0100015558.hg.1	6.6	7.62	-2.02	3.79E-06	3.05E-05	PPIAL4E	peptidylprolyl isomerase A (cyclophilin A)-like	Coding
TC0400009856.hg.1	7.36	8.56	-2.3	3.79E-06	3.05E-05	LYAR	Ly1 antibody reactive	Multiple_Complex
TSUnmapped00000661.h	10.43	11.21	-1.72	3.80E-06	3.05E-05	INPP5D	inositol polyphosphate-5-phosphatase D	Coding
TC0200011014.hg.1	10.41	9.5	1.88	3.81E-06	3.06E-05	FBXO36	Transcript Identified by AceView, Entrez Gene	Unassigned
TC1500009797.hg.1	10.26	10.88	-1.54	3.83E-06	3.08E-05	DPP8	dipeptidyl-peptidase 8	Multiple_Complex
TC0500012094.hg.1	5.71	5.08	1.55	3.84E-06	3.09E-05	C5orf66-AS	C5orf66 antisense RNA 1	Multiple_Complex
TC2200008181.hg.1	5.62	6.33	-1.63	3.85E-06	3.09E-05	ZNF70	zinc finger protein 70	Multiple_Complex
TC2000009848.hg.1	5.79	6.55	-1.68	3.87E-06	3.10E-05	UCKL1; MIF	uridine-cytidine kinase 1-like 1; microRNA 191	Multiple_Complex
TC1800009286.hg.1	13.8	9.6	18.4	3.90E-06	3.13E-05	RPL17-C18	RPL17-C18orf32 readthrough	Coding
TC0500007915.hg.1	6.22	5.14	2.13	3.98E-06	3.19E-05	FAM151B	family with sequence similarity 151, member	Multiple_Complex
TC0200016018.hg.1	9.35	10.38	-2.04	4.00E-06	3.21E-05	NCL	nucleolin	Multiple_Complex
TSUnmapped00000399.h	5.87	6.85	-1.97	4.01E-06	3.21E-05	ZNF852	zinc finger protein 852	Coding
TC0300013888.hg.1	10.9	9.94	1.95	4.01E-06	3.21E-05	TSC22D2	TSC22 domain family, member 2	Multiple_Complex
TC0100018080.hg.1	11.28	10.13	2.23	4.02E-06	3.22E-05	AHCTF1	AT hook containing transcription factor 1	Multiple_Complex
TC0300014063.hg.1	6.23	5.48	1.68	4.04E-06	3.24E-05	ANKUB1	ankyrin repeat and ubiquitin domain containir	NonCoding
TC1500009865.hg.1	14.05	12.72	2.51	4.06E-06	3.25E-05	ANP32A	acidic nuclear phosphoprotein 32 family mem	Multiple_Complex
TC1900009603.hg.1	3.98	4.57	-1.51	4.10E-06	3.28E-05	OLFM2	olfactomedin 2	Multiple_Complex
TC0300009147.hg.1	15.58	14.2	2.59	4.11E-06	3.28E-05	RNF13	ring finger protein 13	Multiple_Complex
TC0100015895.hg.1	7.22	6.27	1.92	4.12E-06	3.29E-05	RAB13	RAB13, member RAS oncogene family	Multiple_Complex
TC0100015254.hg.1	5.48	6.76	-2.43	4.13E-06	3.30E-05	LRIF1	ligand dependent nuclear receptor interacting	Multiple_Complex
TC0800008510.hg.1	6.91	8.05	-2.2	4.14E-06	3.31E-05	OXR1	oxidation resistance 1	Multiple_Complex
TC0700013442.hg.1	7.52	6.1	2.67	4.16E-06	3.32E-05	LSMEM1	leucine-rich single-pass membrane protein 1	Multiple_Complex
TC1400007860.hg.1	3.91	3.39	1.43	4.18E-06	3.33E-05	FLRT2	fibronectin leucine rich transmembrane prote	Multiple_Complex
TC1200011971.hg.1	11.92	8.95	7.83	4.18E-06	3.33E-05	RPL6	ribosomal protein L6	Multiple_Complex
TC0500009059.hg.1	6.89	8.03	-2.22	4.19E-06	3.34E-05	GRPEL2	GrpE-like 2, mitochondrial (E. coli)	Multiple_Complex
TC0100007412.hg.1	4.54	5.85	-2.47	4.20E-06	3.35E-05	MAN1C1	mannosidase, alpha, class 1C, member 1	Multiple_Complex
TC1900008907.hg.1	19.07	19.57	-1.42	4.22E-06	3.36E-05	RPL28; MIF	ribosomal protein L28; microRNA 6805	Multiple_Complex
TC1200010702.hg.1	5.59	6.66	-2.11	4.25E-06	3.39E-05	SMAGP	small cell adhesion glycoprotein	Multiple_Complex
TC1700010699.hg.1	9.48	8.57	1.89	4.29E-06	3.42E-05	DNAJC7	DnaJ (Hsp40) homolog, subfamily C, member	Multiple_Complex
TC0200016757.hg.1	7.25	6.15	2.14	4.30E-06	3.42E-05	NCKAP1	NCK-associated protein 1	Multiple_Complex
TC1800008776.hg.1	7.12	9.09	-3.91	4.33E-06	3.44E-05	FECH	ferrochelatase	Multiple_Complex
TC1700012198.hg.1	5.51	6.6	-2.12	4.33E-06	3.44E-05	PIGL	phosphatidylinositol glycan anchor biosynthes	Multiple_Complex
TC0100016290.hg.1	5.33	6.21	-1.84	4.33E-06	3.44E-05	TADA1	transcriptional adaptor 1	Multiple_Complex
TC0600011927.hg.1	5.74	6.92	-2.26	4.35E-06	3.46E-05	SUPT3H	SPT3 homolog, SAGA and STAGA complex con	Multiple_Complex
TC1700008888.hg.1	7.44	8.43	-1.98	4.36E-06	3.46E-05	ARMC7	armadillo repeat containing 7	Multiple_Complex
TC2000009956.hg.1	8.28	9.38	-2.14	4.37E-06	3.47E-05	PCMTD2	protein-L-isoaspartate (D-aspartate) O-methyl	Multiple_Complex
TC1900010009.hg.1	9.69	7.96	3.31	4.38E-06	3.48E-05	JUND	jun D proto-oncogene	Coding
TC0600006808.hg.1	5.84	6.73	-1.86	4.40E-06	3.49E-05	FARS2	phenylalanyl-tRNA synthetase 2, mitochondria	Multiple_Complex
TC1000011588.hg.1	7.62	6.85	1.71	4.40E-06	3.49E-05	SLC25A28	solute carrier family 25 (mitochondrial iron tra	Multiple_Complex
TC0700010947.hg.1	6.65	7.68	-2.04	4.41E-06	3.49E-05	TBRG4; SN(	transforming growth factor beta regulator 4; s	Multiple_Complex
TC0800008179.hg.1	12.1	10.63	2.75	4.45E-06	3.53E-05	RIPK2	receptor-interacting serine-threonine kinase 2	Multiple_Complex
TC1700010563.hg.1	6.15	7.04	-1.85	4.46E-06	3.53E-05	CACNB1	calcium channel, voltage-dependent, beta 1 su	Multiple_Complex
TC2200007677.hg.1	7.73	8.9	-2.24	4.46E-06	3.53E-05	TBC1D22A	TBC1 domain family, member 22A	Multiple_Complex
TC1100008385.hg.1	9.64	8.47	2.25	4.46E-06	3.53E-05	RELT	RELT tumor necrosis factor receptor	Multiple_Complex
TC0600011646.hg.1	8.87	6.78	4.26	4.48E-06	3.54E-05	SLC26A8	solute carrier family 26 (anion exchanger), me	Multiple_Complex
TC1400010703.hg.1	6.81	7.76	-1.93	4.50E-06	3.56E-05	MTA1	metastasis associated 1	Multiple_Complex
TC1100009521.hg.1	15.42	13.73	3.22	4.53E-06	3.58E-05	APLP2	amyloid beta (A4) precursor-like protein 2	Multiple_Complex
TC2000009292.hg.1	9.95	11	-2.07	4.54E-06	3.59E-05	ZMYND8	zinc finger, MYND-type containing 8	Multiple_Complex
TC0300013051.hg.1	11.79	9.85	3.85	4.54E-06	3.59E-05	PDCD10	programmed cell death 10	Multiple_Complex
TC0700010687.hg.1	10.27	9.18	2.13	4.56E-06	3.60E-05	KBTBD2	kelch repeat and BTB (POZ) domain containing	Multiple_Complex
TC0300009677.hg.1	7	7.92	-1.9	4.60E-06	3.63E-05	YEATS2	YEATS domain containing 2	Multiple_Complex
TC1200008734.hg.1	6.43	7.4	-1.95	4.60E-06	3.63E-05	RIC8B	RIC8 guanine nucleotide exchange factor B	Multiple_Complex
TC0700010081.hg.1	6.61	7.43	-1.77	4.60E-06	3.63E-05	CARD11	caspase recruitment domain family, member :	Multiple_Complex
TC1600008137.hg.1	5.72	6.52	-1.74	4.61E-06	3.64E-05	CES3; CES2	carboxylesterase 3; carboxylesterase 2	Multiple_Complex
TC1600007113.hg.1	8.69	9.82	-2.19	4.68E-06	3.69E-05	C16orf62	chromosome 16 open reading frame 62	Multiple_Complex
TSUnmapped00000293.h	8.85	10.2	-2.55	4.68E-06	3.69E-05	RCC2	regulator of chromosome condensation 2	NonCoding
TC1700009260.hg.1	8.64	9.65	-2.02	4.68E-06	3.69E-05	FOXK2	forkhead box K2	Multiple_Complex
TC0200016464.hg.1	7.14	5.74	2.64	4.69E-06	3.70E-05	APLF	aprataxin and PNKP like factor	Multiple_Complex
TC0600007650.hg.1	13	15.8	-6.97	4.70E-06	3.70E-05	HLA-DRA	major histocompatibility complex, class II, DR	Coding

TC1400008913.hg.1	11.27	10.13	2.19	4.70E-06	3.70E-05	SNX6	sorting nexin 6	Multiple_Complex
TC0600010867.hg.1	8.91	7.95	1.95	4.72E-06	3.71E-05	MCUR1	mitochondrial calcium uniporter regulator 1	Multiple_Complex
TC0100009428.hg.1	6.62	7.39	-1.72	4.77E-06	3.75E-05	DDX20	DEAD (Asp-Glu-Ala-Asp) box polypeptide 20	Multiple_Complex
TC0300008331.hg.1	3.89	4.8	-1.87	4.77E-06	3.75E-05	ABHD10	abhydrolase domain containing 10	Multiple_Complex
TC0100011721.hg.1	12.32	13.66	-2.53	4.80E-06	3.77E-05	CAPN2	calpain 2, (m/II) large subunit	Multiple_Complex
TC1100006491.hg.1	12.56	14.71	-4.43	4.81E-06	3.78E-05	RPLP2; SNC	ribosomal protein, large, P2; small nucleolar R	Multiple_Complex
TC0900007457.hg.1	9.09	6.35	6.66	4.84E-06	3.80E-05	CNTNAP3P	contactin associated protein-like 3 pseudogen	Multiple_Complex
TSUnmapped00000705.h	10.06	8.57	2.8	4.88E-06	3.83E-05	RPS25	ribosomal protein S25	NonCoding
TC0700013343.hg.1	9.55	10.43	-1.84	4.89E-06	3.84E-05	ZNRF2	zinc and ring finger 2, E3 ubiquitin protein liga	Multiple_Complex
TC1100007728.hg.1	10.28	11.56	-2.43	4.90E-06	3.85E-05	FAM111A	family with sequence similarity 111, member .	Multiple_Complex
TC0600011770.hg.1	9.12	7.07	4.15	4.90E-06	3.85E-05	TREML1	triggering receptor expressed on myeloid cells	Multiple_Complex
TC1600007985.hg.1	5	3.97	2.05	4.94E-06	3.87E-05	CETP	cholesteryl ester transfer protein, plasma	Multiple_Complex
TC0400008882.hg.1	10.86	9.51	2.55	4.97E-06	3.89E-05	SMARCA5	SWI/SNF related, matrix associated, actin dep	Multiple_Complex
TC0300013417.hg.1	6.51	5.19	2.5	4.98E-06	3.90E-05	LIPH	lipase, member H	Multiple_Complex
TC0200007353.hg.1	11.09	12.05	-1.94	5.00E-06	3.91E-05	EML4	echinoderm microtubule associated protein lil	Multiple_Complex
TC0100014201.hg.1	5.11	5.93	-1.76	5.02E-06	3.93E-05	CC2D1B	coiled-coil and C2 domain containing 1B	Multiple_Complex
TC1100012303.hg.1	7.46	8.34	-1.84	5.03E-06	3.93E-05	PPP2R1B	protein phosphatase 2, regulatory subunit A, I	Multiple_Complex
TC0900010311.hg.1	6.18	5.24	1.91	5.03E-06	3.93E-05	FAM27E3	family with sequence similarity 27, member E	Multiple_Complex
TC0500010872.hg.1	5.21	5.96	-1.68	5.04E-06	3.94E-05	ERCC8	excision repair cross-complementation group	Multiple_Complex
TC0700013224.hg.1	6.57	5.61	1.94	5.06E-06	3.95E-05	LMBR1	Transcript Identified by AceView, Entrez Gene	Unassigned
TC1300009933.hg.1	9.58	10.28	-1.61	5.06E-06	3.96E-05	RASA3	RAS p21 protein activator 3	Multiple_Complex
TC0100017788.hg.1	5.76	6.99	-2.35	5.08E-06	3.97E-05	TARBP1	TAR (HIV-1) RNA binding protein 1	Multiple_Complex
TC2000010026.hg.1	9.16	9.73	-1.47	5.08E-06	3.97E-05	TMEM189	transmembrane protein 189	Multiple_Complex
TC1400007478.hg.1	6.36	7.38	-2.03	5.10E-06	3.98E-05	GPHN	gephyrin	Multiple_Complex
TC0X00009462.hg.1	7.25	8.28	-2.04	5.10E-06	3.98E-05	CASK	calcium/calmodulin-dependent serine protein	Multiple_Complex
TC0400012933.hg.1	8.07	9.88	-3.51	5.11E-06	3.98E-05	NAAA	N-acylethanolamine acid amidase	Multiple_Complex
TC0900009579.hg.1	6.62	8.03	-2.67	5.13E-06	4.00E-05	ZDHHC21	zinc finger, DHHC-type containing 21	Multiple_Complex
TC0600009783.hg.1	4.71	5.91	-2.28	5.14E-06	4.01E-05	UST	uronyl-2-sulfotransferase	Multiple_Complex
TC1600011345.hg.1	5.74	6.51	-1.71	5.16E-06	4.02E-05	DNASE1	deoxyribonuclease I	Multiple_Complex
TC0600013425.hg.1	5.78	6.65	-1.83	5.18E-06	4.03E-05	ADAT2	adenosine deaminase, tRNA-specific 2	Multiple_Complex
TC0X00007158.hg.1	4.67	5.99	-2.5	5.21E-06	4.06E-05	ZNF81	zinc finger protein 81	Multiple_Complex
TC1600010409.hg.1	5.17	6.63	-2.75	5.23E-06	4.07E-05	BBS2	Bardet-Biedl syndrome 2	Multiple_Complex
TC2000009744.hg.1	5.08	4.3	1.71	5.23E-06	4.07E-05	LAMA5; MI	laminin, alpha 5; microRNA 4758	Multiple_Complex
TC2100007816.hg.1	5.17	4.26	1.88	5.25E-06	4.09E-05	CYYR1	cysteine/tyrosine-rich 1	Coding
TC0700008104.hg.1	9.22	9.96	-1.67	5.27E-06	4.10E-05	LOC100133	uncharacterized LOC100133091; Salzman2013	Multiple_Complex
TC0600011912.hg.1	10.51	9.64	1.82	5.27E-06	4.10E-05	SLC35B2; N	solute carrier family 35 (adenosine 3-phospho	Multiple_Complex
TC0X00008526.hg.1	6.67	6.01	1.59	5.29E-06	4.11E-05	FHL1	four and a half LIM domains 1	Multiple_Complex
TC0400008902.hg.1	8.07	9.39	-2.51	5.31E-06	4.13E-05	ABCE1	ATP binding cassette subfamily E member 1	Multiple_Complex
TC0500007691.hg.1	9.06	10.3	-2.35	5.33E-06	4.14E-05	SMN1; SMI	survival of motor neuron 1, telomeric; surviva	Multiple_Complex
TC0600008087.hg.1	6.36	7.18	-1.77	5.33E-06	4.14E-05	ABCC10	ATP binding cassette subfamily C member 10	Multiple_Complex
TC0500011418.hg.1	10.52	11.91	-2.63	5.33E-06	4.14E-05	MEF2C	myocyte enhancer factor 2C	Multiple_Complex
TC0500012642.hg.1	8.37	9.36	-1.99	5.35E-06	4.14E-05	RNF145	ring finger protein 145	Multiple_Complex
TC0500009422.hg.1	10.02	7.91	4.32	5.38E-06	4.17E-05	NPM1	nucleophosmin (nucleolar phosphoprotein B2	Multiple_Complex
TC0300009194.hg.1	5.24	4.51	1.66	5.39E-06	4.18E-05	SUCNR1	succinate receptor 1	Coding
TC0300011525.hg.1	9.45	8.11	2.53	5.41E-06	4.19E-05	RYBP	RING1 and YY1 binding protein	Multiple_Complex
TC0100011374.hg.1	7.73	8.61	-1.84	5.44E-06	4.21E-05	IKBKE	inhibitor of kappa light polypeptide gene enha	Multiple_Complex
TC1000006816.hg.1	7.88	9.03	-2.23	5.45E-06	4.22E-05	OPTN	optineurin	Multiple_Complex
TC1500009089.hg.1	11.26	10.41	1.8	5.46E-06	4.23E-05	PLCB2	phospholipase C, beta 2	Multiple_Complex
TC1900006932.hg.1	6.21	7.25	-2.06	5.52E-06	4.27E-05	ZNF559-ZN	ZNF559-ZNF177 readthrough; zinc finger prot	Multiple_Complex
TC0X00008481.hg.1	6.94	8.33	-2.61	5.57E-06	4.31E-05	FAM122C	family with sequence similarity 122C	Multiple_Complex
TC0100013969.hg.1	6.48	7.32	-1.79	5.58E-06	4.31E-05	ERI3; ERI3-I	ERI1 exoribonuclease family member 3; ERI3 i	Multiple_Complex
TC0900007029.hg.1	12.4	11.1	2.47	5.59E-06	4.32E-05	UBAP1	ubiquitin associated protein 1	Multiple_Complex
TC1200008488.hg.1	15.58	13.7	3.68	5.61E-06	4.33E-05	PLXNC1	plexin C1	Multiple_Complex
TC1400007584.hg.1	5.04	7.03	-4	5.62E-06	4.34E-05	TTC9	tetratricopeptide repeat domain 9	Coding
TC0100007690.hg.1	8.14	8.85	-1.64	5.63E-06	4.34E-05	RBBP4	retinoblastoma binding protein 4	Multiple_Complex
TC1000012505.hg.1	4.27	5.38	-2.15	5.70E-06	4.39E-05	LHPP	phospholysine phosphohistidine inorganic pyr	Multiple_Complex
TC0900008515.hg.1	10.29	8.83	2.74	5.72E-06	4.41E-05	ATP6V1G1	ATPase, H+ transporting, lysosomal 13kDa, V1	Multiple_Complex
TC1100009234.hg.1	7.76	6.71	2.07	5.76E-06	4.44E-05	TRAPPC4; I	trafficking protein particle complex 4; microRI	Multiple_Complex
TC2200009187.hg.1	7.8	6.75	2.08	5.76E-06	4.44E-05	TUBA8	tubulin, alpha 8	Multiple_Complex
TC0X00007134.hg.1	7.73	8.71	-1.98	5.85E-06	4.51E-05	USP11	ubiquitin specific peptidase 11	Multiple_Complex
TC1900010221.hg.1	6.44	7.42	-1.97	5.91E-06	4.55E-05	ZNF681	zinc finger protein 681	Multiple_Complex
TC0200016699.hg.1	6.13	7.52	-2.61	5.91E-06	4.55E-05	TRABD2A	TraB domain containing 2A	Multiple_Complex
TC1400010636.hg.1	7.91	6.65	2.39	5.96E-06	4.59E-05	SLIRP	SRA stem-loop interacting RNA binding protei	Multiple_Complex
TC1700009939.hg.1	4.31	4.94	-1.55	5.96E-06	4.59E-05	PEMT	phosphatidylethanolamine N-methyltransfera	Multiple_Complex
TC0600007694.hg.1	5.74	6.73	-1.99	5.97E-06	4.59E-05	B3GALT4	UDP-Gal:betaGlcNAc beta 1,3-galactosyltransf	Multiple_Complex
TC1400010085.hg.1	7.81	9.62	-3.51	5.97E-06	4.59E-05	TCL1A	T-cell leukemia/lymphoma 1A	Multiple_Complex
TC0600008468.hg.1	5.29	6.59	-2.47	6.02E-06	4.63E-05	FAM135A	family with sequence similarity 135, member .	Multiple_Complex
TC1200008729.hg.1	6.15	6.88	-1.65	6.03E-06	4.63E-05	POLR3B	polymerase (RNA) III (DNA directed) polypepti	Multiple_Complex
TC0300011860.hg.1	11.5	8.91	6.03	6.06E-06	4.66E-05	RPL24	ribosomal protein L24	Multiple_Complex
TC1700007599.hg.1	10.59	11.28	-1.61	6.11E-06	4.69E-05	AP2B1	adaptor-related protein complex 2, beta 1 sub	Multiple_Complex

TC0300009202.hg.1	15.8	16.75	-1.94	6.12E-06	4.70E-05	MBNL1	muscleblind like splicing regulator 1	Multiple_Complex
TC1800006748.hg.1	6.41	7.22	-1.75	6.12E-06	4.70E-05	SEH1L	SEH1-like nucleoporin	Multiple_Complex
TC0600011553.hg.1	7.39	8.4	-2.01	6.14E-06	4.71E-05	BAK1	BCL2-antagonist/killer 1	Multiple_Complex
TC1500008616.hg.1	6.14	6.96	-1.76	6.17E-06	4.73E-05	HERC2P3	hect domain and RLD 2 pseudogene 3	Multiple_Complex
TC0200008507.hg.1	9.62	8.8	1.77	6.19E-06	4.74E-05	ARID5A	AT rich interactive domain 5A (MRF1-like)	Multiple_Complex
TC0500009996.hg.1	7.08	8.08	-2.01	6.19E-06	4.74E-05	NSUN2	NOP2/Sun RNA methyltransferase family, mer	Multiple_Complex
TC0500013212.hg.1	8.69	7.4	2.45	6.26E-06	4.79E-05	SRP19; ZRS	signal recognition particle 19kDa; zinc finger (	Multiple_Complex
TC1900011414.hg.1	6.99	9.66	-6.37	6.28E-06	4.81E-05	LAIR1	leukocyte-associated immunoglobulin-like rec	Multiple_Complex
TC1000011135.hg.1	6.2	7.09	-1.86	6.31E-06	4.83E-05	POLR3A	polymerase (RNA) III (DNA directed) polypepti	Multiple_Complex
TC1900007871.hg.1	11.03	10.15	1.85	6.34E-06	4.85E-05	COX6B1	cytochrome c oxidase subunit VIb polypeptide	Multiple_Complex
TC0900010470.hg.1	6.94	5.79	2.22	6.35E-06	4.85E-05	PRUNE2	prune homolog 2 (Drosophila)	Multiple_Complex
TC1200009903.hg.1	8.92	12.3	-10.42	6.36E-06	4.86E-05	KLRC4-KLRI	KLRC4-KLRK1 readthrough; killer cell lectin-lik	Multiple_Complex
TC0300009625.hg.1	7.28	8.36	-2.12	6.37E-06	4.87E-05	TTC14	tetratricopeptide repeat domain 14	Multiple_Complex
TC0600012529.hg.1	8.16	9.25	-2.13	6.39E-06	4.88E-05	RNGTT	RNA guanylyltransferase and 5-phosphatase	Multiple_Complex
TC0X00006704.hg.1	9.26	7.25	4.03	6.40E-06	4.89E-05	REPS2	RALBP1 associated Eps domain containing 2	Multiple_Complex
TC1100009962.hg.1	9.62	8.51	2.16	6.41E-06	4.89E-05	FAM160A2	family with sequence similarity 160, member	Multiple_Complex
TC0900011603.hg.1	7.32	9.7	-5.19	6.41E-06	4.89E-05	ENG	endoglin	Multiple_Complex
TC0100015200.hg.1	4.44	5.36	-1.89	6.46E-06	4.92E-05	AMIGO1	adhesion molecule with Ig-like domain 1	Coding
TC1200007408.hg.1	4.34	5.6	-2.4	6.46E-06	4.93E-05	TMEM117	transmembrane protein 117	Multiple_Complex
TC0500010599.hg.1	6.33	7.3	-1.95	6.47E-06	4.93E-05	OXCT1	3-oxoacid CoA-transferase 1	Multiple_Complex
TC1400007906.hg.1	6.37	7.54	-2.25	6.47E-06	4.93E-05	ZC3H14	zinc finger CCCH-type containing 14	Multiple_Complex
TC0100011215.hg.1	5.7	6.58	-1.84	6.51E-06	4.96E-05	LGR6	leucine-rich repeat containing G protein-coup	Multiple_Complex
TC0200016422.hg.1	12.28	11.12	2.24	6.56E-06	4.99E-05	PPP1CB	protein phosphatase 1, catalytic subunit, beta	Multiple_Complex
TC1700007560.hg.1	4.01	5.11	-2.14	6.56E-06	4.99E-05	CCL8	chemokine (C-C motif) ligand 8	Coding
TC0100017568.hg.1	6.94	7.65	-1.64	6.59E-06	5.02E-05	JMJD4	jumonji domain containing 4	Multiple_Complex
TC0700011570.hg.1	5.51	6.1	-1.5	6.61E-06	5.03E-05	YWHAG	tyrosine 3-monooxygenase/tryptophan 5-mor	Multiple_Complex
TC0600008095.hg.1	6.59	7.79	-2.3	6.63E-06	5.04E-05	POLR1C	polymerase (RNA) I polypeptide C	Multiple_Complex
TC0100015273.hg.1	7.84	8.64	-1.73	6.63E-06	5.04E-05	WDR77	WD repeat domain 77	Multiple_Complex
TC1200009036.hg.1	6.2	7.13	-1.9	6.65E-06	5.05E-05	FBXW8	F-box and WD repeat domain containing 8	Multiple_Complex
TC2200009353.hg.1	8.49	9.41	-1.89	6.73E-06	5.11E-05	SUN2	Sad1 and UNC84 domain containing 2	Multiple_Complex
TC0300013727.hg.1	8.02	8.88	-1.81	6.76E-06	5.13E-05	NCBP2	nuclear cap binding protein subunit 2	Multiple_Complex
TC1000008954.hg.1	5.96	6.63	-1.59	6.79E-06	5.15E-05	CASP7	caspase 7	Multiple_Complex
TC1300007256.hg.1	7.36	4.54	7.03	6.83E-06	5.18E-05	OLFM4	olfactomedin 4	Coding
TC0100008874.hg.1	10.17	11.52	-2.54	6.84E-06	5.19E-05	PRKACB	protein kinase, cAMP-dependent, catalytic, be	Multiple_Complex
TC0300014095.hg.1	4.71	5.66	-1.93	6.85E-06	5.19E-05	TM4SF19	transmembrane 4 L six family member 19	Multiple_Complex
TC0200016093.hg.1	6.1	6.88	-1.72	6.85E-06	5.20E-05	USP40	ubiquitin specific peptidase 40	Multiple_Complex
TC0800009865.hg.1	5.46	6.33	-1.82	6.86E-06	5.20E-05	RP11-875O	Salzman2013 ANTISENSE, CDS, coding, INTERN	NonCoding
TC1700010214.hg.1	4.47	5.39	-1.89	6.87E-06	5.20E-05	FAM222B	family with sequence similarity 222, member	Multiple_Complex
TC1700008923.hg.1	6.55	7.4	-1.81	6.88E-06	5.21E-05	UNK	unkempt family zinc finger	Multiple_Complex
TC0700009398.hg.1	4.87	5.82	-1.93	6.88E-06	5.21E-05	TAS2R3	taste receptor, type 2, member 3	Coding
TC0100008260.hg.1	6.49	5.87	1.53	6.89E-06	5.21E-05	CDKN2C	cyclin-dependent kinase inhibitor 2C (p18, inh	Coding
TC0600012554.hg.1	6.7	8.28	-2.99	6.94E-06	5.25E-05	BACH2	BTB and CNC homology 1, basic leucine zipper	Multiple_Complex
TC0400011538.hg.1	9.29	7.91	2.6	6.97E-06	5.27E-05	PAPSS1	3-phosphoadenosine 5-phosphosulfate syntha	Multiple_Complex
TC1500007217.hg.1	6.44	7.6	-2.23	6.97E-06	5.27E-05	AP4E1	adaptor-related protein complex 4, epsilon 1 s	Multiple_Complex
TC0X00011389.hg.1	8.15	7.23	1.9	6.99E-06	5.28E-05	NKAP	NFKB activating protein	Multiple_Complex
TC0100009468.hg.1	4.52	5.65	-2.19	7.00E-06	5.29E-05	LRIG2	leucine-rich repeats and immunoglobulin-like	Multiple_Complex
TC0600012702.hg.1	4.63	5.53	-1.86	7.03E-06	5.30E-05	HACE1	HECT domain and ankyrin repeat containing E	Multiple_Complex
TC1500010792.hg.1	11.1	9.58	2.85	7.10E-06	5.35E-05	MCTP2	multiple C2 domains, transmembrane 2	Multiple_Complex
TC1600010375.hg.1	4.74	3.74	2	7.10E-06	5.36E-05	CRNDE	colorectal neoplasia differentially expressed (r	Multiple_Complex
TC0300007067.hg.1	11.91	13.53	-3.05	7.13E-06	5.38E-05	MYD88	myeloid differentiation primary response 88	Multiple_Complex
TC0500013237.hg.1	6.64	7.37	-1.65	7.16E-06	5.40E-05	HARS2	histidyl-tRNA synthetase 2, mitochondrial	Multiple_Complex
TC1700012383.hg.1	5.48	4.56	1.89	7.18E-06	5.41E-05	OMG	oligodendrocyte myelin glycoprotein	Multiple_Complex
TC1200007832.hg.1	5.74	6.85	-2.16	7.18E-06	5.41E-05	NABP2	nucleic acid binding protein 2	Multiple_Complex
TC2200007312.hg.1	11.41	9.69	3.3	7.24E-06	5.45E-05	LGALS1	lectin, galactoside-binding, soluble, 1	Multiple_Complex
TC0100007449.hg.1	15.09	16.22	-2.19	7.24E-06	5.45E-05	SH3BGRL3	SH3 domain binding glutamate-rich protein lik	Multiple_Complex
TC1700008463.hg.1	8.3	7.1	2.29	7.27E-06	5.47E-05	DHX40	DEAH (Asp-Glu-Ala-His) box polypeptide 40	Multiple_Complex
TC1700010849.hg.1	8.3	9.21	-1.88	7.28E-06	5.48E-05	EFTUD2	elongation factor Tu GTP binding domain cont	Multiple_Complex
TC1100011258.hg.1	6.27	7.13	-1.82	7.29E-06	5.48E-05	FIBP	fibroblast growth factor (acidic) intracellular b	Multiple_Complex
TC0700006633.hg.1	9.38	8.42	1.95	7.29E-06	5.48E-05	CCZ1; CCZ1	CCZ1 homolog, vacuolar protein trafficking an	Multiple_Complex
TC1200007842.hg.1	6.74	7.61	-1.83	7.30E-06	5.48E-05	SPRYD4	SPRY domain containing 4	Coding
TC0900012033.hg.1	6.08	7.09	-2.01	7.30E-06	5.48E-05	DPP7	dipeptidyl-peptidase 7	Multiple_Complex
TC0400011168.hg.1	12.47	13.45	-1.96	7.31E-06	5.49E-05	HNRNPD	heterogeneous nuclear ribonucleoprotein D	Multiple_Complex
TC1000008535.hg.1	7.3	8.12	-1.76	7.33E-06	5.50E-05	ZNF518A	zinc finger protein 518A	Multiple_Complex
TC1100011587.hg.1	4.31	5.69	-2.59	7.34E-06	5.51E-05	FAM168A	family with sequence similarity 168, member	Multiple_Complex
TC0500013351.hg.1	10.4	9.38	2.03	7.38E-06	5.54E-05	FNIP1	folliculin interacting protein 1	Multiple_Complex
TC0500007841.hg.1	8.14	9.36	-2.32	7.39E-06	5.55E-05	AGGF1	angiogenic factor with G-patch and FHA doma	Multiple_Complex
TC0X00009184.hg.1	6.19	4.96	2.34	7.42E-06	5.56E-05	BEND2	BEN domain containing 2	Multiple_Complex
TC0200016710.hg.1	9.12	10.46	-2.53	7.42E-06	5.56E-05	ANKRD36C	ankyrin repeat domain 36C	Multiple_Complex
TC0700008579.hg.1	7.63	8.62	-1.99	7.46E-06	5.59E-05	TRIM56	tripartite motif containing 56	Multiple_Complex
TC0100017167.hg.1	4.89	3.92	1.96	7.47E-06	5.59E-05	LAMB3; MI	laminin, beta 3; microRNA 4260	Multiple_Complex

TC1600007990.hg.1	7.75	9.02	-2.4	7.47E-06	5.59E-05	RSPRY1	ring finger and SPRY domain containing 1	Multiple_Complex
TC0800007080.hg.1	14.71	16.63	-3.78	7.49E-06	5.60E-05	BNIP3L	BCL2/adenovirus E1B 19kDa interacting prote	Multiple_Complex
TC1400007625.hg.1	6.93	7.77	-1.79	7.51E-06	5.62E-05	RBM25	RNA binding motif protein 25	Multiple_Complex
TC0100017617.hg.1	6.41	5.46	1.92	7.53E-06	5.63E-05	HIST3H2A	histone cluster 3, H2a	Coding
TC1100010483.hg.1	8.25	9.17	-1.89	7.54E-06	5.64E-05	CSTF3	cleavage stimulation factor, 3 pre-RNA, subun	Multiple_Complex
TC1900011858.hg.1	4.6	5.51	-1.88	7.54E-06	5.64E-05	ZNF846	zinc finger protein 846	Multiple_Complex
TC0100010074.hg.1	6.19	5.25	1.91	7.61E-06	5.69E-05	AQP10	aquaporin 10	Multiple_Complex
TC2100006585.hg.1	5.91	4.97	1.92	7.64E-06	5.71E-05	RBM11	RNA binding motif protein 11	Multiple_Complex
TC2200008449.hg.1	5.63	6.59	-1.96	7.67E-06	5.72E-05	DUSP18	dual specificity phosphatase 18	Multiple_Complex
TC0200013511.hg.1	7.73	6.56	2.26	7.67E-06	5.73E-05	DUSP2	dual specificity phosphatase 2	Multiple_Complex
TC0600014045.hg.1	6.93	7.74	-1.75	7.71E-06	5.75E-05	PDCD2	programmed cell death 2	Multiple_Complex
TC0300012029.hg.1	11.66	12.53	-1.83	7.74E-06	5.77E-05	NAA50	N(alpha)-acetyltransferase 50, NatE catalytic s	Multiple_Complex
TC1200012593.hg.1	10.28	8.53	3.37	7.76E-06	5.78E-05	CLEC4A	C-type lectin domain family 4, member A	Coding
TC1700010817.hg.1	8.75	9.49	-1.67	7.76E-06	5.78E-05	UBTF	upstream binding transcription factor, RNA pc	Multiple_Complex
TSUnmapped00000277.h	5.34	4.55	1.74	7.79E-06	5.80E-05	PSMC4	proteasome 26S subunit, ATPase 4 [Source:HC	NonCoding
TC2200008837.hg.1	9.03	9.69	-1.58	7.84E-06	5.84E-05	SNU13	SNU13 homolog, small nuclear ribonucleoprot	Multiple_Complex
TC0500012271.hg.1	9.13	7.27	3.62	7.84E-06	5.84E-05	TAF7	TAF7 RNA polymerase II, TATA box binding pro	Multiple_Complex
TC1600010439.hg.1	10.87	9.65	2.34	7.88E-06	5.86E-05	FAM192A	family with sequence similarity 192, member .	Multiple_Complex
TC2200009278.hg.1	8.28	6.54	3.35	7.92E-06	5.89E-05	RBX1	ring-box 1, E3 ubiquitin protein ligase	Multiple_Complex
TC1600007362.hg.1	11.61	12.69	-2.12	7.93E-06	5.90E-05	ATXN2L	ataxin 2-like	Multiple_Complex
TC1100011091.hg.1	5.43	6.18	-1.68	7.97E-06	5.93E-05	B3GAT3	beta-1,3-glucuronyltransferase 3	Multiple_Complex
TC1900009001.hg.1	4.31	5.45	-2.22	7.97E-06	5.93E-05	ZIK1	zinc finger protein interacting with K protein 1	Multiple_Complex
TC0700006737.hg.1	6.72	5.61	2.15	7.98E-06	5.93E-05	ARL4A	ADP-ribosylation factor like GTPase 4A	Coding
TC0200015397.hg.1	9.55	7.87	3.21	7.98E-06	5.93E-05	CLK1	CDC like kinase 1	Multiple_Complex
TC0X00009882.hg.1	4.03	4.81	-1.71	7.99E-06	5.93E-05	MTMR8	myotubularin related protein 8	Multiple_Complex
TC1500010290.hg.1	5.04	6.23	-2.29	8.00E-06	5.94E-05	WDR73	WD repeat domain 73	Multiple_Complex
TC0200009949.hg.1	7.1	8.04	-1.92	8.03E-06	5.95E-05	DCAF17	DDB1 and CUL4 associated factor 17	Multiple_Complex
TC0200016624.hg.1	11.34	10.15	2.29	8.13E-06	6.03E-05	KIDINS220	kinase D-interacting substrate 220kDa	Multiple_Complex
TC0900010903.hg.1	6.15	5.39	1.69	8.14E-06	6.04E-05	RP11-569G	uncharacterized protein LOC158434 [Source:I	Multiple_Complex
TC0600007635.hg.1	5.13	6.09	-1.94	8.15E-06	6.04E-05	PPT2; EGFL	palmitoyl-protein thioesterase 2; EGF-like-dor	Multiple_Complex
TC0300012023.hg.1	5.64	6.71	-2.1	8.20E-06	6.07E-05	SPICE1	spindle and centriole associated protein 1	Multiple_Complex
TC0100015591.hg.1	6.7	7.59	-1.86	8.20E-06	6.07E-05	PIAS3	protein inhibitor of activated STAT 3	Multiple_Complex
TC0100006875.hg.1	10.47	8.79	3.19	8.23E-06	6.09E-05	MFN2	mitofusin 2	Multiple_Complex
TC0700010257.hg.1	6.51	5.27	2.36	8.23E-06	6.09E-05	ICA1	islet cell autoantigen 1	Multiple_Complex
TC1200010693.hg.1	7.36	8.54	-2.27	8.25E-06	6.10E-05	TFCP2	transcription factor CP2	Multiple_Complex
TC1000009111.hg.1	7.22	8.19	-1.96	8.26E-06	6.11E-05	WDR11	WD repeat domain 11	Multiple_Complex
TC0800007416.hg.1	5.55	4.57	1.97	8.26E-06	6.11E-05	GINS4	GINS complex subunit 4 (Sld5 homolog)	Multiple_Complex
TC1200012636.hg.1	16.6	17.58	-1.98	8.29E-06	6.12E-05	PCBP2; PCE	poly(rC) binding protein 2; PCBP2 overlapping	Multiple_Complex
TC2000007336.hg.1	8.46	9.28	-1.76	8.44E-06	6.24E-05	PPP1R16B	protein phosphatase 1, regulatory subunit 16E	Multiple_Complex
TC0100010824.hg.1	6.5	5.25	2.37	8.44E-06	6.24E-05	CACNA1E	calcium channel, voltage-dependent, R type, a	Multiple_Complex
TC0200012320.hg.1	5.36	6.34	-1.97	8.48E-06	6.26E-05	MAP4K3	mitogen-activated protein kinase kinase kinas	Multiple_Complex
TC0600011377.hg.1	9.4	10.41	-2.01	8.49E-06	6.27E-05	NRM	nurim (nuclear envelope membrane protein)	Multiple_Complex
TC2200007001.hg.1	5.74	6.64	-1.86	8.51E-06	6.28E-05	HSCB	HscB mitochondrial iron-sulfur cluster co-chap	Multiple_Complex
TC1900008366.hg.1	6.88	6.13	1.68	8.53E-06	6.29E-05	IGFL1	IGF like family member 1	Coding
TC0700010170.hg.1	8.46	9.23	-1.7	8.54E-06	6.30E-05	RNF216	ring finger protein 216	Multiple_Complex
TC2100008487.hg.1	7.02	6.31	1.63	8.56E-06	6.31E-05	TPTE	transmembrane phosphatase with tensin hom	Multiple_Complex
TC1100007247.hg.1	12.99	13.85	-1.82	8.56E-06	6.31E-05	CAPRIN1	cell cycle associated protein 1	Multiple_Complex
TC1200010601.hg.1	7.78	8.93	-2.2	8.60E-06	6.33E-05	FKBP11; AF	FK506 binding protein 11; ADP-ribosylation fa	Multiple_Complex
TC1200010838.hg.1	7.9	9.22	-2.5	8.63E-06	6.36E-05	ZNF385A	zinc finger protein 385A	Multiple_Complex
TC0700008000.hg.1	12.74	11.68	2.09	8.64E-06	6.36E-05	LAT2	linker for activation of T-cells family member 2	Multiple_Complex
TC0700007065.hg.1	5.25	6.38	-2.19	8.67E-06	6.38E-05	PLEKHA8	pleckstrin homology domain containing, famil	Multiple_Complex
TC1700009708.hg.1	10.59	11.33	-1.67	8.73E-06	6.42E-05	PIK3R5	phosphoinositide-3-kinase, regulatory subunit	Multiple_Complex
TC0300009609.hg.1	5.76	6.53	-1.71	8.73E-06	6.42E-05	ACTL6A	actin-like 6A	Multiple_Complex
TC0300013821.hg.1	4.99	5.75	-1.69	8.74E-06	6.43E-05	ZNF197	zinc finger protein 197	Multiple_Complex
TC0100008584.hg.1	6.4	7.68	-2.43	8.75E-06	6.43E-05	ALG6	ALG6, alpha-1,3-glucosyltransferase	Multiple_Complex
TC2200008620.hg.1	4.82	5.59	-1.71	8.77E-06	6.44E-05	IFT27	intraflagellar transport 27	Multiple_Complex
TC1800009280.hg.1	4.42	5.87	-2.72	8.79E-06	6.46E-05	HDHD2	haloacid dehalogenase-like hydrolase domain	Multiple_Complex
TC0400012930.hg.1	6.37	7.32	-1.93	8.82E-06	6.47E-05	COX18	COX18 cytochrome c oxidase assembly factor	Multiple_Complex
TC0900009414.hg.1	7.49	8.32	-1.77	8.82E-06	6.47E-05	RFX3	regulatory factor X, 3 (influences HLA class II e	Multiple_Complex
TC0200015779.hg.1	4.95	5.91	-1.94	8.85E-06	6.49E-05	DNPEP	aspartyl aminopeptidase	Multiple_Complex
TC0100016971.hg.1	14.67	16.89	-4.65	8.95E-06	6.56E-05	ADIPOR1	adiponectin receptor 1	Multiple_Complex
TC0500009211.hg.1	10.9	11.82	-1.89	8.96E-06	6.57E-05	CYFIP2	cytoplasmic FMR1 interacting protein 2	Multiple_Complex
TC0500010639.hg.1	5.19	6.24	-2.06	8.97E-06	6.57E-05	CCL28	chemokine (C-C motif) ligand 28	Multiple_Complex
TC0X00007744.hg.1	13.04	11.99	2.07	8.98E-06	6.58E-05	SH3BGRL	SH3 domain binding glutamate-rich protein lik	Multiple_Complex
TSUnmapped00000038.h	6.3	7.2	-1.87	9.07E-06	6.64E-05	ZNF780A	zinc finger protein 780A	Coding
TC1700010560.hg.1	5.68	6.92	-2.36	9.07E-06	6.64E-05	PLXDC1	plexin domain containing 1	Multiple_Complex
TC0200007205.hg.1	7.17	8.28	-2.17	9.07E-06	6.64E-05	RASGRP3	RAS guanyl releasing protein 3 (calcium and D	Multiple_Complex
TC2000007820.hg.1	5.03	6.42	-2.63	9.15E-06	6.69E-05	CASS4	Cas scaffolding protein family member 4	Multiple_Complex
TC0800012288.hg.1	7.92	6.51	2.65	9.20E-06	6.72E-05	DCTN6	dynactin 6	Multiple_Complex
TC1100008664.hg.1	7.18	8.33	-2.22	9.23E-06	6.75E-05	C11orf73	chromosome 11 open reading frame 73	Multiple_Complex

TC2200007495.hg.1	7.66	8.63	-1.96	9.23E-06	6.75E-05	SREBF2	sterol regulatory element binding transcriptio	Multiple_Complex
TC0300012187.hg.1	4.6	5.89	-2.45	9.27E-06	6.77E-05	WDR5B	WD repeat domain 5B	Coding
TC1900011724.hg.1	5.1	5.98	-1.84	9.31E-06	6.80E-05	ZNF540; ZN	zinc finger protein 540; ZNF571 antisense RNA	Multiple_Complex
TC0800011305.hg.1	4.09	4.89	-1.74	9.31E-06	6.80E-05	NCALD	neurocalcin delta	Multiple_Complex
TC1500010093.hg.1	9.05	8.22	1.78	9.46E-06	6.90E-05	ETFA	electron-transfer-flavoprotein, alpha polypept	Multiple_Complex
TC1100012681.hg.1	8.32	6.73	3.02	9.46E-06	6.90E-05	SIAE	sialic acid acetylesterase	Multiple_Complex
TC0100009064.hg.1	5.65	4.59	2.08	9.47E-06	6.91E-05	FNBP1L	formin binding protein 1-like	Multiple_Complex
TC1900011636.hg.1	10.8	9.66	2.2	9.48E-06	6.91E-05	UBE2M	ubiquitin-conjugating enzyme E2M	Multiple_Complex
TC1100008123.hg.1	9.68	11	-2.49	9.48E-06	6.91E-05	ADRBK1	adrenergic, beta, receptor kinase 1	Multiple_Complex
TC0300013874.hg.1	6.12	7.24	-2.17	9.50E-06	6.92E-05	SRPRB	signal recognition particle receptor, B subunit	Multiple_Complex
TC1500008312.hg.1	16.19	14.8	2.61	9.58E-06	6.97E-05	IQGAP1	IQ motif containing GTPase activating protein	Multiple_Complex
TC0500007699.hg.1	9.23	10.23	-2	9.59E-06	6.98E-05	SMN1	survival of motor neuron 1, telomeric	Multiple_Complex
TC1000010022.hg.1	13.9	15.29	-2.62	9.65E-06	7.02E-05	PIP4K2A	phosphatidylinositol-5-phosphate 4-kinase, ty	Multiple_Complex
TC0200007064.hg.1	8.34	9.09	-1.68	9.67E-06	7.04E-05	SNX17	sorting nexin 17	Multiple_Complex
TC0200011972.hg.1	4.32	5.35	-2.04	9.72E-06	7.07E-05	C2orf44	chromosome 2 open reading frame 44	Coding
TC1300006965.hg.1	7.81	6.99	1.76	9.74E-06	7.08E-05	WBP4	WW domain binding protein 4	Coding
TC0100010102.hg.1	7.64	8.29	-1.57	9.75E-06	7.09E-05	FLAD1	flavin adenine dinucleotide synthetase 1	Multiple_Complex
TC1100012831.hg.1	8.5	9.4	-1.86	9.79E-06	7.11E-05	ZBTB44	zinc finger and BTB domain containing 44	Multiple_Complex
TC1900011725.hg.1	5.65	4.74	1.88	9.80E-06	7.12E-05	SPINT2	serine peptidase inhibitor, Kunitz type, 2	Multiple_Complex
TC0100009876.hg.1	11.93	10.23	3.25	9.81E-06	7.12E-05	HIST2H2AA	histone cluster 2, H2aa3; histone cluster 2, H2	Coding
TC2100008533.hg.1	6.24	5.35	1.85	9.85E-06	7.15E-05	PCBP3	poly(rC) binding protein 3	NonCoding
TC0100010044.hg.1	6.82	7.89	-2.09	9.94E-06	7.21E-05	CHTOP	chromatin target of PRMT1	Multiple_Complex
TC1200010690.hg.1	4.95	5.74	-1.73	9.96E-06	7.22E-05	CSRNP2	cysteine-serine-rich nuclear protein 2	Multiple_Complex
TC0100009568.hg.1	9.63	10.71	-2.12	1.01E-05	7.32E-05	MAN1A2	mannosidase, alpha, class 1A, member 2	Multiple_Complex
TC1500009261.hg.1	8.32	9.39	-2.11	1.01E-05	7.33E-05	SPG11	spastic paraplegia 11 (autosomal recessive)	Multiple_Complex
TC0700012680.hg.1	6.03	7	-1.95	1.01E-05	7.34E-05	SLC35B4	solute carrier family 35 (UDP-xylose/UDP-N-ac	Multiple_Complex
TC1100011514.hg.1	6.11	5.05	2.08	1.02E-05	7.37E-05	DHCR7	7-dehydrocholesterol reductase	Multiple_Complex
TC1900009491.hg.1	7.24	8.89	-3.14	1.02E-05	7.37E-05	FCER2	Fc fragment of IgE, low affinity II, receptor for	Multiple_Complex
TC0600014127.hg.1	7.26	7.84	-1.49	1.02E-05	7.39E-05	RPL7L1	ribosomal protein L7-like 1	Multiple_Complex
TC0500006816.hg.1	8.79	7.74	2.07	1.02E-05	7.40E-05	FAM105A	family with sequence similarity 105, member	Multiple_Complex
TC1900008084.hg.1	6.69	7.8	-2.16	1.02E-05	7.41E-05	PSMC4	proteasome 26S subunit, ATPase 4	Multiple_Complex
TC1000009420.hg.1	7.02	6.16	1.82	1.03E-05	7.43E-05	INPP5A	Transcript Identified by AceView, Entrez Gene	Unassigned
TC0700012451.hg.1	5.2	4.36	1.79	1.03E-05	7.43E-05	TAS2R16	taste receptor, type 2, member 16	Coding
TC0900012276.hg.1	6.03	7.02	-1.98	1.03E-05	7.47E-05	SCAI; GOLG	suppressor of cancer cell invasion; golgin A1	Multiple_Complex
TC0200013357.hg.1	6.99	6.33	1.58	1.04E-05	7.51E-05	KRCC1	lysine-rich coiled-coil 1	Multiple_Complex
TC0600009884.hg.1	6.82	5.67	2.21	1.05E-05	7.58E-05	MYCT1	myc target 1	Coding
TC0700011489.hg.1	4.91	4.4	1.43	1.05E-05	7.58E-05	TRIM50	tripartite motif containing 50	Multiple_Complex
TC0300013123.hg.1	7.39	6.42	1.96	1.05E-05	7.58E-05	PLD1	phospholipase D1, phosphatidylcholine-specif	Multiple_Complex
TC1600011406.hg.1	7.14	6.06	2.11	1.06E-05	7.62E-05	CKLF-CMTN	CKLF-CMTM1 readthrough	Coding
TC0600011823.hg.1	7.79	8.67	-1.84	1.06E-05	7.65E-05	TRERF1	transcriptional regulating factor 1	Multiple_Complex
TC1900006576.hg.1	7.14	5.91	2.36	1.06E-05	7.66E-05	DOT1L	DOT1-like histone H3K79 methyltransferase	Multiple_Complex
TC0100007574.hg.1	12.81	14.5	-3.21	1.07E-05	7.68E-05	EPB41	erythrocyte membrane protein band 4.1	Multiple_Complex
TC0500009009.hg.1	8.13	9.27	-2.21	1.07E-05	7.71E-05	TCERG1	transcription elongation regulator 1	Multiple_Complex
TC1100007467.hg.1	5.1	6.03	-1.91	1.08E-05	7.78E-05	C11orf49	chromosome 11 open reading frame 49	Multiple_Complex
TC0500007408.hg.1	7.56	10.32	-6.75	1.08E-05	7.78E-05	GZMK	granzyme K	Multiple_Complex
TC0600014279.hg.1	6.9	7.77	-1.82	1.08E-05	7.78E-05	DAXX	death-domain associated protein	Multiple_Complex
TC1000012536.hg.1	10.38	9.32	2.07	1.08E-05	7.78E-05	MPP7	membrane protein, palmitoylated 7	Multiple_Complex
TC1900010957.hg.1	9.06	10.06	-1.99	1.08E-05	7.78E-05	SYMPK	symplekin	Multiple_Complex
TC0X00010198.hg.1	5.06	6.11	-2.07	1.08E-05	7.79E-05	HDX	highly divergent homeobox	Multiple_Complex
TC0300012567.hg.1	7.73	8.66	-1.9	1.09E-05	7.86E-05	DBR1	debranching RNA lariats 1	Multiple_Complex
TC0200007144.hg.1	7.93	9.06	-2.18	1.10E-05	7.88E-05	LCLAT1	lysocardiolipin acyltransferase 1	Multiple_Complex
TC0500011896.hg.1	6.53	5.23	2.47	1.11E-05	7.94E-05	ZNF608	zinc finger protein 608	Multiple_Complex
TC0100012097.hg.1	11.29	9.85	2.7	1.12E-05	8.06E-05	LGALS8	lectin, galactoside-binding, soluble, 8	Multiple_Complex
TC1000011690.hg.1	8.15	7.2	1.94	1.12E-05	8.07E-05	ARL3	ADP-ribosylation factor like GTPase 3	Multiple_Complex
TC0400009179.hg.1	5.88	6.91	-2.04	1.13E-05	8.12E-05	TMA16	translation machinery associated 16 homolog	Multiple_Complex
TC0300010913.hg.1	6.12	5.46	1.58	1.13E-05	8.13E-05	CDCP1	CUB domain containing protein 1	Multiple_Complex
TC1200012642.hg.1	7.2	6.41	1.74	1.14E-05	8.16E-05	BLOC1S1-R	BLOC1S1-RDH5 readthrough; Uncharacterized	Multiple_Complex
TC0200012755.hg.1	7.91	8.8	-1.85	1.14E-05	8.17E-05	CCT4	chaperonin containing TCP1, subunit 4 (delta)	Multiple_Complex
TC0300012764.hg.1	4.34	5.56	-2.32	1.14E-05	8.20E-05	HLTF	helicase-like transcription factor	Multiple_Complex
TC0300013788.hg.1	9.42	8.66	1.69	1.15E-05	8.21E-05	ARPC4-TTL	ARPC4-TTLL3 readthrough	Coding
TC1900011781.hg.1	7.79	8.58	-1.73	1.15E-05	8.21E-05	PRMT1	protein arginine methyltransferase 1	Multiple_Complex
TC0100013758.hg.1	6.55	7.49	-1.92	1.15E-05	8.22E-05	INPP5B	inositol polyphosphate-5-phosphatase B	Multiple_Complex
TC1400009225.hg.1	9.71	8.7	2.01	1.15E-05	8.23E-05	GMFB	glia maturation factor, beta	Multiple_Complex
TC0100007884.hg.1	9.86	8.98	1.84	1.15E-05	8.26E-05	AKIRIN1	akirin 1	Multiple_Complex
TC1200006773.hg.1	6.38	8.65	-4.84	1.16E-05	8.26E-05	KLRF1	killer cell lectin-like receptor subfamily F, men	Multiple_Complex
TC1500007711.hg.1	5.57	4.81	1.7	1.16E-05	8.27E-05	TLE3	Memczak2013 ANTISENSE, coding, INTERNAL,	NonCoding
TC1400009529.hg.1	13.66	11.88	3.45	1.16E-05	8.29E-05	ACTN1; HM	actinin, alpha 1; high mobility group nucleoso	Multiple_Complex
TC0100011364.hg.1	8.66	9.83	-2.24	1.16E-05	8.29E-05	SRGAP2	SLIT-ROBO Rho GTPase activating protein 2	Multiple_Complex
TC0500011881.hg.1	6.54	7.9	-2.56	1.16E-05	8.29E-05	CEP120	centrosomal protein 120kDa	Multiple_Complex
TC2000007281.hg.1	6.8	7.53	-1.65	1.17E-05	8.35E-05	MANBAL	mannosidase, beta A, lysosomal-like	Multiple_Complex

TC0800009830.hg.1	6.9	7.99	-2.12	1.17E-05	8.35E-05	REEP4	receptor accessory protein 4	Multiple_Complex
TC0700010760.hg.1	10.22	11.28	-2.09	1.17E-05	8.35E-05	ELMO1	engulfment and cell motility 1	Multiple_Complex
TC0800012324.hg.1	4.88	5.58	-1.63	1.18E-05	8.45E-05	LRRC69; M	leucine rich repeat containing 69; microRNA 4	Multiple_Complex
TC0800007370.hg.1	4.64	3.85	1.73	1.19E-05	8.45E-05	ADAM32	ADAM metallopeptidase domain 32	Multiple_Complex
TC1500009173.hg.1	6.93	6.21	1.65	1.19E-05	8.48E-05	EHD4	EH domain containing 4	Multiple_Complex
TC0100011940.hg.1	6.55	7.76	-2.31	1.20E-05	8.54E-05	COG2	component of oligomeric golgi complex 2	Multiple_Complex
TC1100011395.hg.1	7.57	6.63	1.91	1.20E-05	8.54E-05	CPT1A	carnitine palmitoyltransferase 1A (liver)	Multiple_Complex
TC0100006675.hg.1	10.2	11.08	-1.85	1.21E-05	8.63E-05	KCNAB2	potassium channel, voltage gated subfamily A	Multiple_Complex
TC1200006967.hg.1	9.35	8.66	1.6	1.21E-05	8.64E-05	DERA	deoxyribose-phosphate aldolase (putative)	Multiple_Complex
TC0900008959.hg.1	7.07	8.01	-1.92	1.21E-05	8.64E-05	FUBP3	far upstream element (FUSE) binding protein 3	Multiple_Complex
TC1700009383.hg.1	7.38	6.06	2.51	1.23E-05	8.72E-05	PITPNA	Memczak2013 ALT_ACCEPTOR, ALT_DONOR,	NonCoding
TC0100010877.hg.1	5.53	6.38	-1.81	1.23E-05	8.75E-05	TSEN15	TSEN15 tRNA splicing endonuclease subunit	Multiple_Complex
TC0300014020.hg.1	10.09	11.36	-2.42	1.23E-05	8.77E-05	CLDND1	claudin domain containing 1	Multiple_Complex
TC1700008945.hg.1	5.62	4.73	1.85	1.23E-05	8.77E-05	SPHK1	sphingosine kinase 1	Multiple_Complex
TC1400009386.hg.1	5.31	4.69	1.55	1.24E-05	8.79E-05	GPHB5	glycoprotein hormone beta 5	Multiple_Complex
TC1600011408.hg.1	9.63	8.48	2.21	1.24E-05	8.81E-05	CKLF; CKLF-	chemokine-like factor; CKLF-CMTM1 readthro	Multiple_Complex
TC0100006494.hg.1	4.89	5.55	-1.58	1.24E-05	8.81E-05	B3GALT6	UDP-Gal:betaGal beta 1,3-galactosyltransferas	Coding
TC1400008764.hg.1	10.74	12.42	-3.19	1.24E-05	8.82E-05	GZMB	granzyme B	Coding
TC1000012519.hg.1	8.46	9.16	-1.63	1.25E-05	8.84E-05	IL15RA	interleukin 15 receptor, alpha	Multiple_Complex
TC1500007180.hg.1	6.03	7.27	-2.36	1.25E-05	8.89E-05	GALK2	galactokinase 2	Multiple_Complex
TC0700012658.hg.1	7.33	8.35	-2.03	1.25E-05	8.89E-05	CHCHD3	coiled-coil-helix-coiled-coil-helix domain conta	Multiple_Complex
TC0900009861.hg.1	4.38	5.17	-1.72	1.26E-05	8.90E-05	NOL6	nucleolar protein 6 (RNA-associated)	Multiple_Complex
TC0700011003.hg.1	8.9	9.76	-1.82	1.26E-05	8.92E-05	HUS1	HUS1 checkpoint clamp component	Multiple_Complex
TC1200009137.hg.1	7.95	8.87	-1.89	1.26E-05	8.93E-05	MLEC	malectin	Multiple_Complex
TC0200008040.hg.1	6.29	7.15	-1.81	1.26E-05	8.93E-05	SMYD5	SMYD family member 5	Multiple_Complex
TC1200010490.hg.1	7.09	8.92	-3.56	1.27E-05	8.96E-05	NELL2	neural EGFL like 2	Multiple_Complex
TC0700012684.hg.1	6.78	7.81	-2.04	1.27E-05	8.98E-05	AKR1B1	aldo-keto reductase family 1, member B1 (ald	Multiple_Complex
TC1300008125.hg.1	10.23	9.19	2.04	1.27E-05	9.00E-05	LAMP1	lysosomal-associated membrane protein 1	Multiple_Complex
TC0600012656.hg.1	4.85	5.94	-2.13	1.27E-05	9.00E-05	USP45	ubiquitin specific peptidase 45	Multiple_Complex
TC1100009858.hg.1	11.57	10.56	2.02	1.27E-05	9.00E-05	CHRNA10;	cholinergic receptor, nicotinic alpha 10; nucle	Multiple_Complex
TC1400008763.hg.1	6.66	8.82	-4.47	1.28E-05	9.03E-05	GZMH	granzyme H	Coding
TC0400010168.hg.1	8.62	9.64	-2.03	1.28E-05	9.05E-05	LCORL	ligand dependent nuclear receptor corepresso	Multiple_Complex
TC1100013128.hg.1	5.85	5.09	1.7	1.29E-05	9.10E-05	IFITM10	interferon induced transmembrane protein 1(	Multiple_Complex
TC0600008035.hg.1	9.78	10.82	-2.06	1.30E-05	9.15E-05	TAF8	TATA box binding protein associated factor 8	Multiple_Complex
TC0900006597.hg.1	7.54	8.59	-2.07	1.30E-05	9.16E-05	KDM4C	lysine (K)-specific demethylase 4C	Multiple_Complex
TC1900008669.hg.1	4.88	5.81	-1.91	1.31E-05	9.22E-05	FPR3	formyl peptide receptor 3	Coding
TC1000012589.hg.1	6.71	7.7	-2	1.31E-05	9.23E-05	MRPL43	mitochondrial ribosomal protein L43	Multiple_Complex
TC0300010209.hg.1	8.57	9.71	-2.2	1.31E-05	9.24E-05	TADA3	transcriptional adaptor 3	Multiple_Complex
TC0700012959.hg.1	7.56	6.83	1.66	1.32E-05	9.28E-05	EZH2	enhancer of zeste 2 polycomb repressive com	Multiple_Complex
TC1300009979.hg.1	7.91	6.45	2.76	1.32E-05	9.28E-05	UCHL3	ubiquitin C-terminal hydrolase L3	Multiple_Complex
TC0200015424.hg.1	5.6	6.89	-2.44	1.32E-05	9.32E-05	ALS2CR12	amyotrophic lateral sclerosis 2 chromosome r	Multiple_Complex
TC0500008656.hg.1	6.37	5.02	2.56	1.33E-05	9.35E-05	AFF4	Memczak2013 ANTISENSE, CDS, coding, INTEI	NonCoding
TC0200013578.hg.1	9.87	11.41	-2.92	1.35E-05	9.49E-05	TMEM131	transmembrane protein 131	Multiple_Complex
TC0600013361.hg.1	9.59	11.23	-3.12	1.35E-05	9.51E-05	CITED2	Cbp/p300-interacting transactivator, with Glu,	Coding
TC0600009206.hg.1	11.25	10.13	2.18	1.35E-05	9.51E-05	MARCKS	myristoylated alanine-rich protein kinase C su	Multiple_Complex
TC0200015211.hg.1	11.04	9.67	2.58	1.35E-05	9.52E-05	SLC40A1	solute carrier family 40 (iron-regulated transp	Multiple_Complex
TC0400008348.hg.1	5.8	6.9	-2.15	1.36E-05	9.53E-05	HADH	hydroxyacyl-CoA dehydrogenase	Multiple_Complex
TC2000009957.hg.1	9.8	8.68	2.17	1.36E-05	9.53E-05	LINC00266-	long intergenic non-protein coding RNA 266-1	Multiple_Complex
TC0200007181.hg.1	8.84	10.12	-2.43	1.36E-05	9.55E-05	YIPF4	Yip1 domain family member 4	Multiple_Complex
TC1500007355.hg.1	17.51	15.78	3.32	1.36E-05	9.55E-05	AQP9	aquaporin 9	Multiple_Complex
TC0300009782.hg.1	10.45	9.02	2.68	1.36E-05	9.57E-05	EIF4A2; SN(	eukaryotic translation initiation factor 4A2; sm	Multiple_Complex
TC0100009745.hg.1	7.01	8.18	-2.26	1.36E-05	9.58E-05	POLR3C	polymerase (RNA) III (DNA directed) polypepti	Multiple_Complex
TC0200013902.hg.1	11.42	10.46	1.95	1.37E-05	9.63E-05	RGPD5; RG	RANBP2-like and GRIP domain containing 5; R	Multiple_Complex
TC1200011321.hg.1	13.94	12.31	3.11	1.38E-05	9.67E-05	OSBPL8	oxysterol binding protein-like 8	Multiple_Complex
TC0500008014.hg.1	7.47	8.25	-1.72	1.38E-05	9.70E-05	RASA1	RAS p21 protein activator (GTPase activating p	Multiple_Complex
TC0300012315.hg.1	6.19	7.15	-1.95	1.39E-05	9.71E-05	TPRA1	transmembrane protein, adipocyte asscociate	Multiple_Complex
TC1900010372.hg.1	5.01	6.1	-2.13	1.39E-05	9.73E-05	CEP89	centrosomal protein 89kDa	Multiple_Complex
TC0900008207.hg.1	8.07	7.13	1.91	1.39E-05	9.73E-05	SEC61B	Sec61 translocon beta subunit	Multiple_Complex
TC0800012190.hg.1	5.74	6.77	-2.04	1.39E-05	9.75E-05	SHARPIN	SHANK-associated RH domain interactor	Multiple_Complex
TC1600006644.hg.1	6.22	7.17	-1.93	1.40E-05	9.79E-05	FLYWCH1	FLYWCH-type zinc finger 1	Multiple_Complex
TC0300013751.hg.1	4.17	4.91	-1.68	1.40E-05	9.80E-05	BDH1	3-hydroxybutyrate dehydrogenase, type 1	Multiple_Complex
TC0500008849.hg.1	9.98	8.75	2.36	1.41E-05	9.83E-05	CYSTM1	cysteine-rich transmembrane module contain	Multiple_Complex
TC2000007687.hg.1	8.46	6.96	2.83	1.41E-05	9.86E-05	LINC01270	long intergenic non-protein coding RNA 1270	Multiple_Complex
TC0500008633.hg.1	8.29	9.32	-2.04	1.41E-05	9.87E-05	C5orf56	chromosome 5 open reading frame 56	Multiple_Complex
TC0200016750.hg.1	10.97	10	1.97	1.42E-05	9.92E-05	NFE2L2	nuclear factor, erythroid 2-like 2	Multiple_Complex
TC0600006442.hg.1	6.64	7.59	-1.93	1.42E-05	9.94E-05	IRF4	interferon regulatory factor 4	Multiple_Complex
TC1100013032.hg.1	8.67	10.01	-2.53	1.43E-05	9.95E-05	DNAJC4	DnaJ (Hsp40) homolog, subfamily C, member	Multiple_Complex
TC0100007439.hg.1	6.33	7.26	-1.91	1.43E-05	9.99E-05	PDIK1L	PDLIM1 interacting kinase 1 like	Coding
TC0100015750.hg.1	7.45	6.16	2.45	1.43E-05	9.99E-05	HORMAD1	HORMA domain containing 1	Multiple_Complex
TSUnmapped00000517.h	7.02	8.23	-2.31	1.43E-05	0.0001	HYOU1	hypoxia up-regulated 1	Coding

TC1400009248.hg.1	5.14	4.12	2.03	1.44E-05	0.0001	DLGAP5	discs, large (Drosophila) homolog-associated p	Multiple_Complex
TC0100007784.hg.1	10.7	9.11	3.01	1.45E-05	0.0001	AGO4	argonaute RISC catalytic component 4	Multiple_Complex
TC1500008245.hg.1	14.92	13.75	2.24	1.46E-05	0.0001	ABHD2	abhydrolase domain containing 2	Multiple_Complex
TC1000012561.hg.1	14.29	13.08	2.32	1.47E-05	0.0001	JMJD1C	jumonji domain containing 1C	Multiple_Complex
TC0200011803.hg.1	5.38	6.61	-2.35	1.47E-05	0.0001	NBAS	neuroblastoma amplified sequence	Multiple_Complex
TC0200010801.hg.1	8.94	9.74	-1.75	1.48E-05	0.0001	RQCD1	RCD1 required for cell differentiation1 homolo	Multiple_Complex
TC0200016684.hg.1	8.62	9.5	-1.83	1.48E-05	0.0001	TIA1	TIA1 cytotoxic granule-associated RNA binding	Multiple_Complex
TC0700013619.hg.1	5.84	6.82	-1.97	1.49E-05	0.0001	RBM28	RNA binding motif protein 28	Multiple_Complex
TC0900009860.hg.1	6.23	7.11	-1.83	1.49E-05	0.0001	AQP3	aquaporin 3 (Gill blood group)	Multiple_Complex
TC1100009884.hg.1	6.1	6.7	-1.52	1.49E-05	0.0001	TRIM68	tripartite motif containing 68	Multiple_Complex
TC0800012344.hg.1	6.62	5.39	2.35	1.49E-05	0.0001	ZFPM2	zinc finger protein, FOG family member 2	Multiple_Complex
TC0300010664.hg.1	9.11	10.1	-1.99	1.49E-05	0.0001	GLB1; TMP	galactosidase beta 1; transmembrane protein	Multiple_Complex
TC1100009241.hg.1	7.18	8.1	-1.89	1.49E-05	0.0001	HINFP	histone H4 transcription factor	Multiple_Complex
TC0600013519.hg.1	6.34	7.39	-2.07	1.49E-05	0.0001	ZC3H12D	zinc finger CCCH-type containing 12D	Multiple_Complex
TC1200007037.hg.1	5.12	4.08	2.06	1.50E-05	0.0001	PDE3A	phosphodiesterase 3A, cGMP-inhibited	Multiple_Complex
TC0100007672.hg.1	7.74	6.74	2	1.51E-05	0.0001	EIF3I	eukaryotic translation initiation factor 3, subu	Multiple_Complex
TC0500010779.hg.1	4.76	6.16	-2.64	1.51E-05	0.0001	SLC38A9	solute carrier family 38, member 9	Multiple_Complex
TC0300013601.hg.1	6.41	5.59	1.76	1.51E-05	0.0001	ATP13A3	Jeck2013 ALT_ACCEPTOR, ALT_DONOR, codin	NonCoding
TC0100015728.hg.1	10.82	11.72	-1.87	1.52E-05	0.0001	APH1A	APH1A gamma secretase subunit	Multiple_Complex
TC0200014688.hg.1	6.67	5.4	2.42	1.52E-05	0.0001	ERMN	ermin	Coding
TSUnmapped00000169.h	8.69	9.72	-2.04	1.53E-05	0.0001	TCF20	transcription factor 20 (AR1)	Coding
TSUnmapped00000402.h	8.69	9.72	-2.04	1.53E-05	0.0001	TCF20	transcription factor 20 (AR1)	Coding
TC1100008477.hg.1	13.04	11.51	2.88	1.54E-05	0.0001	DGAT2	diacylglycerol O-acyltransferase 2	Multiple_Complex
TC1100013054.hg.1	6.14	7.22	-2.12	1.54E-05	0.0001	RAD9A	RAD9 checkpoint clamp component A	Multiple_Complex
TC0300008735.hg.1	14.73	16.3	-2.96	1.56E-05	0.0001	RAB7A	RAB7A, member RAS oncogene family	Multiple_Complex
TC0700013405.hg.1	8.72	7.86	1.81	1.56E-05	0.0001	BRI3	brain protein I3	Multiple_Complex
TC0200009829.hg.1	15.49	13.17	4.99	1.56E-05	0.0001	GCA	grancalcin, EF-hand calcium binding protein	Multiple_Complex
TC0400006806.hg.1	5.68	4.86	1.77	1.56E-05	0.0001	USP17L21	ubiquitin specific peptidase 17-like family mer	Coding
TC1700012227.hg.1	7.35	8.38	-2.03	1.56E-05	0.0001	LRRC37B	leucine rich repeat containing 37B	NonCoding
TC0500011754.hg.1	11.22	12.12	-1.86	1.57E-05	0.0001	TMED7-TIC	TMED7-TICAM2 readthrough; toll-like recepto	Multiple_Complex
TC1200012718.hg.1	7.23	6.59	1.56	1.57E-05	0.0001	PSMD9	proteasome 26S subunit, non-ATPase 9	Multiple_Complex
TC1100009237.hg.1	7.95	9.01	-2.08	1.57E-05	0.0001	VPS11	vacuolar protein sorting 11 homolog (S. cerevi	Multiple_Complex
TC0200016601.hg.1	8.08	8.69	-1.53	1.57E-05	0.0001	GIGYF2	GRB10 interacting GYF protein 2	Multiple_Complex
TC1200006648.hg.1	4.91	5.96	-2.06	1.57E-05	0.0001	USP5	ubiquitin specific peptidase 5 (isopeptidase T)	Multiple_Complex
TC2200009219.hg.1	12.02	14.38	-5.15	1.59E-05	0.0001	IGLL5; MIR!	immunoglobulin lambda-like polypeptide 5; m	Multiple_Complex
TC1200012606.hg.1	12.37	11.2	2.26	1.59E-05	0.0001	LRMP	lymphoid-restricted membrane protein	Multiple_Complex
TC1900007108.hg.1	14.07	15.39	-2.5	1.59E-05	0.0001	CALR	calreticulin	Multiple_Complex
TC0800012240.hg.1	13.67	15.11	-2.7	1.60E-05	0.0001	RPL8; MIR6	ribosomal protein L8; microRNA 6850	Multiple_Complex
TC2100006618.hg.1	10.97	12.18	-2.31	1.60E-05	0.0001	USP25	ubiquitin specific peptidase 25	Multiple_Complex
TC1200011985.hg.1	8.89	9.68	-1.73	1.60E-05	0.0001	DDX54	DEAD (Asp-Glu-Ala-Asp) box polypeptide 54	Multiple_Complex
TC2200007645.hg.1	5.38	4.52	1.81	1.60E-05	0.0001	LOC730668	dynein heavy chain -like pseudogene; TEC	NonCoding
TC1200011367.hg.1	10.66	9.54	2.16	1.60E-05	0.0001	PPP1R12A	protein phosphatase 1, regulatory subunit 12A	Multiple_Complex
TC0200016344.hg.1	5.62	6.84	-2.34	1.60E-05	0.0001	MTERF4	mitochondrial transcription termination factor	Multiple_Complex
TC1700006733.hg.1	6.15	6.85	-1.62	1.61E-05	0.0001	ELP5	elongator acetyltransferase complex subunit 5	Multiple_Complex
TC1600011351.hg.1	6.9	7.85	-1.93	1.61E-05	0.0001	CARHSP1	Jeck2013 ANTISENSE, CDS, coding, INTERNAL,	NonCoding
TC0200009447.hg.1	12.14	11.42	1.65	1.62E-05	0.0001	UBXN4	UBX domain protein 4	Multiple_Complex
TC0600007143.hg.1	7.33	8.38	-2.06	1.63E-05	0.0001	CDKAL1	CDK5 regulatory subunit associated protein 1-	Multiple_Complex
TC0500010515.hg.1	5.86	7.06	-2.29	1.63E-05	0.0001	NUP155	nucleoporin 155kDa	Multiple_Complex
TC1400010692.hg.1	7.82	6.89	1.91	1.64E-05	0.0001	APOPT1	apoptogenic 1, mitochondrial	Multiple_Complex
TC0100007243.hg.1	12.35	8.78	11.92	1.65E-05	0.0001	ALPL	alkaline phosphatase, liver/bone/kidney	Multiple_Complex
TC1200012801.hg.1	10.3	11.18	-1.84	1.65E-05	0.0001	CS	citrate synthase	Multiple_Complex
TC0200008063.hg.1	7.33	8.18	-1.81	1.65E-05	0.0001	DGUOK	deoxyguanosine kinase	Multiple_Complex
TC1400006674.hg.1	5.99	6.72	-1.66	1.66E-05	0.0001	C14orf119	chromosome 14 open reading frame 119	Multiple_Complex
TC1900008999.hg.1	4.28	4.88	-1.51	1.67E-05	0.0001	ZNF549	zinc finger protein 549	Coding
TC0500011804.hg.1	5.53	6.52	-1.99	1.67E-05	0.0001	DTWD2	DTW domain containing 2	Multiple_Complex
TC0800011597.hg.1	6.7	5.84	1.81	1.68E-05	0.0001	EXT1; spaw	Jeck2013 ALT_ACCEPTOR, ALT_DONOR, codin	NonCoding
TC1300009538.hg.1	4.84	4.22	1.54	1.69E-05	0.0001	OXGR1	oxoglutarate (alpha-ketoglutarate) receptor 1	Coding
TC1100010175.hg.1	6.61	7.96	-2.56	1.70E-05	0.0001	RRAS2	related RAS viral (r-ras) oncogene homolog 2	Multiple_Complex
TC1300006599.hg.1	4.91	3.9	2.02	1.70E-05	0.0001	C1QTNF9B-	C1QTNF9B antisense RNA 1	Multiple_Complex
TC0200016704.hg.1	9.78	8.81	1.96	1.71E-05	0.0001	RNF103	ring finger protein 103	Multiple_Complex
TC0900011120.hg.1	7.01	5.82	2.27	1.71E-05	0.0001	KLF4	Kruppel-like factor 4 (gut)	Multiple_Complex
TC1300006770.hg.1	17.92	16.96	1.95	1.73E-05	0.0001	ALOX5AP	arachidonate 5-lipoxygenase-activating protei	Multiple_Complex
TC1700010298.hg.1	12.46	13.73	-2.4	1.74E-05	0.0001	CRLF3	Memczak2013 ALT_ACCEPTOR, ALT_DONOR,	NonCoding
TC1000011025.hg.1	8.58	9.61	-2.04	1.74E-05	0.0001	PPP3CB	protein phosphatase 3, catalytic subunit, beta	Multiple_Complex
TC1500010910.hg.1	13.88	9.32	23.67	1.74E-05	0.0001	RPS17	ribosomal protein S17	Multiple_Complex
TC0500012935.hg.1	7.18	8.07	-1.84	1.74E-05	0.0001	KIAA1191	KIAA1191	Multiple_Complex
TC0900009592.hg.1	8.9	9.88	-1.98	1.75E-05	0.0001	PSIP1	PC4 and SFRS1 interacting protein 1	Multiple_Complex
TC0900006937.hg.1	6.22	7.05	-1.78	1.75E-05	0.0001	ACO1	aconitase 1, soluble	Multiple_Complex
TC0800008184.hg.1	9.56	10.37	-1.76	1.77E-05	0.0001	DECR1	2,4-dienoyl-CoA reductase 1, mitochondrial	Multiple_Complex
TC0700011797.hg.1	10.58	13.98	-10.54	1.77E-05	0.0001	SAMD9L	sterile alpha motif domain containing 9-like	Multiple_Complex

TC0200014584.hg.1	9.52	8.49	2.05	1.78E-05	0.0001	MMADHC	methylmalonic aciduria (cobalamin deficiency	Multiple_Complex
TC1500008341.hg.1	11.4	9.94	2.76	1.78E-05	0.0001	MAN2A2	mannosidase, alpha, class 2A, member 2	Multiple_Complex
TC0600007534.hg.1	5.77	6.7	-1.91	1.78E-05	0.0001	PRR3	proline rich 3	Multiple_Complex
TSUnmapped00000635.h	6.49	7.59	-2.15	1.78E-05	0.0001	SLC25A26	solute carrier family 25 (S-adenosylmethionin	Coding
TC0300007068.hg.1	12.73	12.05	1.6	1.78E-05	0.0001	OXSR1	oxidative stress responsive 1	Multiple_Complex
TC0900011260.hg.1	4.15	5.25	-2.14	1.79E-05	0.0001	ALAD	aminolevulinate dehydratase	Multiple_Complex
TC1100012958.hg.1	4.16	5.43	-2.41	1.79E-05	0.0001	OR56B1	olfactory receptor, family 56, subfamily B, me	Coding
TC0X00011382.hg.1	9.15	8.29	1.81	1.79E-05	0.0001	ACSL4	acyl-CoA synthetase long-chain family membe	Multiple_Complex
TC0100018482.hg.1	11.89	10.17	3.31	1.79E-05	0.0001	HIST2H2AA	histone cluster 2, H2aa4; histone cluster 2, H2	Coding
TC2200007489.hg.1	4.37	5.07	-1.63	1.80E-05	0.0001	C22orf46	chromosome 22 open reading frame 46	Multiple_Complex
TC0300011309.hg.1	5.64	4.81	1.77	1.81E-05	0.0001	ACOX2	acyl-CoA oxidase 2, branched chain	Multiple_Complex
TC0200014004.hg.1	8.82	9.89	-2.1	1.81E-05	0.0001	CCDC93	coiled-coil domain containing 93	Multiple_Complex
TC1200010264.hg.1	7.53	8.61	-2.11	1.81E-05	0.0001	IPO8	importin 8	Multiple_Complex
TC0600010095.hg.1	6.22	7.3	-2.11	1.82E-05	0.0001	MAP3K4	mitogen-activated protein kinase kinase kinas	Multiple_Complex
TC0300007078.hg.1	6.47	7.37	-1.87	1.83E-05	0.0001	EXOG	endo/exonuclease (5-3), endonuclease G-like	Multiple_Complex
TC0800007554.hg.1	6.99	5.41	2.99	1.83E-05	0.0001	UBE2V2	ubiquitin conjugating enzyme E2 variant 2	Multiple_Complex
TC0400012934.hg.1	7.43	8.43	-2	1.84E-05	0.0001	SDAD1	SDA1 domain containing 1	Multiple_Complex
TC1900007754.hg.1	5.91	6.83	-1.89	1.84E-05	0.0001	NUDT19	nudix hydrolase 19	Multiple_Complex
TC0100015364.hg.1	9.27	7.91	2.57	1.85E-05	0.0001	TSPAN2	tetraspanin 2	Multiple_Complex
TC0100015352.hg.1	10.29	9.61	1.6	1.85E-05	0.0001	NRAS	neuroblastoma RAS viral (v-ras) oncogene hor	Multiple_Complex
TC0500011984.hg.1	9.88	8.46	2.67	1.85E-05	0.0001	HINT1	histidine triad nucleotide binding protein 1	Multiple_Complex
TC1200009157.hg.1	6.89	7.74	-1.8	1.85E-05	0.0001	P2RX7	purinergic receptor P2X, ligand gated ion char	Multiple_Complex
TC0600007196.hg.1	4.33	5.37	-2.06	1.86E-05	0.0001	MRS2	MRS2 magnesium transporter	Multiple_Complex
TC0100018237.hg.1	9.03	7.85	2.27	1.86E-05	0.0001	RABGGTB;	Rab geranylgeranyltransferase, beta subunit; s	Multiple_Complex
TC0700013584.hg.1	7.13	5.96	2.25	1.86E-05	0.0001	CYP51A1	cytochrome P450, family 51, subfamily A, poly	Multiple_Complex
TC0300007121.hg.1	4.43	5.48	-2.07	1.87E-05	0.0001	ZNF619	zinc finger protein 619	Multiple_Complex
TC0500008431.hg.1	8.63	9.45	-1.77	1.87E-05	0.0001	DMXL1	Dmx-like 1	Multiple_Complex
TC0900008091.hg.1	6.57	7.66	-2.12	1.88E-05	0.0001	ERCC6L2	excision repair cross-complementation group	Multiple_Complex
TC0600013251.hg.1	8.91	8	1.88	1.89E-05	0.0001	HBS1L	HBS1-like translational GTPase	Multiple_Complex
TC1100011606.hg.1	7.87	8.72	-1.81	1.89E-05	0.0001	C2CD3	C2 calcium-dependent domain containing 3	Multiple_Complex
TC0500007249.hg.1	8.64	9.23	-1.5	1.89E-05	0.0001	C5orf51	chromosome 5 open reading frame 51	Multiple_Complex
TC0100008124.hg.1	5.16	6.08	-1.89	1.91E-05	0.0001	MMACHC	methylmalonic aciduria (cobalamin deficiency	Multiple_Complex
TC0200013746.hg.1	6.47	7.37	-1.86	1.91E-05	0.0001	TGFBRAP1	transforming growth factor beta receptor asso	Multiple_Complex
TC2200007027.hg.1	4.61	5.16	-1.47	1.91E-05	0.0001	NF2	neurofibromin 2 (merlin)	Multiple_Complex
TC0300009738.hg.1	8.09	6.78	2.47	1.91E-05	0.0001	SENP2	SUMO1/sentrin/SMT3 specific peptidase 2	Multiple_Complex
TC0900008795.hg.1	6.8	7.77	-1.95	1.93E-05	0.0001	RALGPS1	Ral GEF with PH domain and SH3 binding mot	Multiple_Complex
TC1100011568.hg.1	12.48	13.78	-2.46	1.94E-05	0.0001	FCHSD2	FCH and double SH3 domains 2	Multiple_Complex
TC1000007461.hg.1	6.69	7.7	-2.02	1.94E-05	0.0001	RASSF4	Ras association (RalGDS/AF-6) domain family	Multiple_Complex
TC1900011753.hg.1	4.74	5.67	-1.91	1.95E-05	0.0001	ZNF284	zinc finger protein 284	Multiple_Complex
TC0400011466.hg.1	18.54	17.78	1.7	1.95E-05	0.0001	UBE2D3	ubiquitin conjugating enzyme E2D 3	Multiple_Complex
TC0500010998.hg.1	9.03	7.64	2.62	1.97E-05	0.0001	CCDC125	coiled-coil domain containing 125	Multiple_Complex
TC0300006577.hg.1	9.06	7.99	2.11	1.97E-05	0.0001	BRK1	BRICK1, SCAR/WAVE actin-nucleating complex	Multiple_Complex
TC2200007109.hg.1	5.97	7.03	-2.08	1.97E-05	0.0001	DEPDC5	DEP domain containing 5	Multiple_Complex
TC1700011249.hg.1	7.06	5.71	2.55	1.97E-05	0.0001	MPO	myeloperoxidase	Multiple_Complex
TC0100006884.hg.1	6.71	7.58	-1.82	1.98E-05	0.0001	VPS13D	vacuolar protein sorting 13 homolog D (S. cere	Multiple_Complex
TC0100014218.hg.1	8.17	9.19	-2.04	1.98E-05	0.0001	SCP2	Jeck2013 ANTISENSE, coding, INTERNAL, OVEX	Multiple_Complex
TC1500008988.hg.1	8.93	9.95	-2.03	1.99E-05	0.0001	GOLGA8A;	golgin A8 family, member A; golgin A8 family,	Multiple_Complex
TC0700007267.hg.1	4.56	5.67	-2.15	2.00E-05	0.0001	YAE1D1	Yae1 domain containing 1	Multiple_Complex
TC1700007851.hg.1	18.42	17.6	1.76	2.00E-05	0.0001	EIF1	eukaryotic translation initiation factor 1	Multiple_Complex
TC1600006976.hg.1	5.25	6.54	-2.45	2.01E-05	0.0001	ERCC4	excision repair cross-complementation group	Multiple_Complex
TSUnmapped00000175.h	5.43	6.21	-1.72	2.01E-05	0.0001	NDUFA10	NADH dehydrogenase (ubiquinone) 1 alpha su	Coding
TC1300007549.hg.1	5.45	6.37	-1.89	2.01E-05	0.0001	CLN5	ceroid-lipofuscinosis, neuronal 5	Multiple_Complex
TC0X00010813.hg.1	5.84	6.89	-2.07	2.02E-05	0.0001	ENOX2	ecto-NOX disulfide-thiol exchanger 2	Multiple_Complex
TC0X00010966.hg.1	8.06	9.13	-2.09	2.02E-05	0.0001	ATP11C	ATPase, class VI, type 11C	Multiple_Complex
TC1200010299.hg.1	9.5	8.37	2.19	2.03E-05	0.0001	H3F3C	H3 histone, family 3C	Coding
TC0X00010211.hg.1	5.76	6.82	-2.07	2.04E-05	0.0001	CHM	choroideremia (Rab escort protein 1)	Multiple_Complex
TC0300011039.hg.1	4.33	3.69	1.55	2.05E-05	0.0001	ARIH2OS	ariadne homolog 2 opposite strand	Coding
TC1000012423.hg.1	9.12	9.86	-1.66	2.06E-05	0.0001	PFKP	phosphofructokinase, platelet	Multiple_Complex
TC1700009566.hg.1	10.15	11.2	-2.07	2.06E-05	0.0001	NLRP1	NLR family, pyrin domain containing 1	Multiple_Complex
TC0200012925.hg.1	8.69	7.6	2.14	2.07E-05	0.0001	C1D	C1D nuclear receptor corepressor	Multiple_Complex
TC2000009731.hg.1	9.48	8.15	2.51	2.08E-05	0.0001	PSMA7	proteasome subunit alpha 7	Multiple_Complex
TC0100016318.hg.1	8.17	7.62	1.46	2.09E-05	0.0001	MPC2	mitochondrial pyruvate carrier 2	Multiple_Complex
TC0100015725.hg.1	7.84	6.9	1.92	2.10E-05	0.0001	ANP32E	acidic nuclear phosphoprotein 32 family mem	Multiple_Complex
TC1100007771.hg.1	7.94	9.56	-3.09	2.11E-05	0.0001	MS4A1	membrane-spanning 4-domains, subfamily A,	Multiple_Complex
TC0600007605.hg.1	11.66	12.78	-2.16	2.11E-05	0.0001	LY6G5B; CS	lymphocyte antigen 6 complex, locus G5B; cas	Multiple_Complex
TC0400007617.hg.1	10.26	11.11	-1.81	2.12E-05	0.0001	POLR2B	polymerase (RNA) II (DNA directed) polypeptic	Multiple_Complex
TC0300013108.hg.1	7.6	8.78	-2.27	2.12E-05	0.0001	TNIK	TRAF2 and NCK interacting kinase	Multiple_Complex
TC0100015822.hg.1	18.73	19.43	-1.63	2.13E-05	0.0001	S100A11	S100 calcium binding protein A11	Multiple_Complex
TC0800009232.hg.1	5.33	4.65	1.59	2.13E-05	0.0001	SPATC1	spermatogenesis and centriole associated 1	Coding
TC0500011579.hg.1	12.3	13.58	-2.43	2.13E-05	0.0001	ST8SIA4	ST8 alpha-N-acetyl-neuraminide alpha-2,8-sia	Multiple_Complex

TC0700009369.hg.1	5.07	4.23	1.79	2.13E-05	0.0001	RAB19	RAB19, member RAS oncogene family	Multiple_Complex
TC0500010523.hg.1	7.51	6.68	1.78	2.13E-05	0.0001	GDNF	glial cell derived neurotrophic factor	Multiple_Complex
TC0500011095.hg.1	6.23	7.09	-1.81	2.15E-05	0.0001	MRPS27	mitochondrial ribosomal protein S27	Multiple_Complex
TC0800012446.hg.1	17.53	16.56	1.96	2.15E-05	0.0001	YWHAZ	tyrosine 3-monooxygenase/tryptophan 5-mon	Multiple_Complex
TC0700013596.hg.1	7.08	6.24	1.79	2.15E-05	0.0001	TSC22D4	TSC22 domain family, member 4	Multiple_Complex
TC1500009639.hg.1	6.09	7.32	-2.34	2.16E-05	0.0001	ICE2	interactor of little elongation complex ELL sub	Multiple_Complex
TC1100009225.hg.1	6.24	7.69	-2.73	2.16E-05	0.0001	CXCR5	chemokine (C-X-C motif) receptor 5	Coding
TC1000011323.hg.1	9.24	10.13	-1.86	2.16E-05	0.0001	GLUD1	glutamate dehydrogenase 1	Multiple_Complex
TC1800006655.hg.1	6.32	5.58	1.67	2.17E-05	0.0001	TXNDC2	thioredoxin domain containing 2 (spermatozo	Multiple_Complex
TC0900010485.hg.1	12.93	11.13	3.5	2.18E-05	0.0001	GNAQ	guanine nucleotide binding protein (G protein	Multiple_Complex
TC0100016337.hg.1	4.84	6.22	-2.6	2.18E-05	0.0001	XCL2	chemokine (C motif) ligand 2	Coding
TC1200012708.hg.1	10.24	14.63	-20.93	2.19E-05	0.0001	OAS1	2-5-oligoadenylate synthetase 1	Multiple_Complex
TC1400010045.hg.1	15.12	14.11	2.01	2.19E-05	0.0001	SERPINA1	serpin peptidase inhibitor, clade A (alpha-1 an	Multiple_Complex
TC1600011314.hg.1	5.86	7.45	-3	2.19E-05	0.0001	HBM	hemoglobin, mu	Coding
TC0600013205.hg.1	17.83	14.73	8.58	2.20E-05	0.0001	VNN2	vanin 2	Multiple_Complex
TC2100007327.hg.1	5.11	5.69	-1.5	2.20E-05	0.0001	RRP1	ribosomal RNA processing 1	Multiple_Complex
TC1200012841.hg.1	8.49	7.51	1.97	2.22E-05	0.0001	SSH1	slingshot protein phosphatase 1	Multiple_Complex
TC1600008709.hg.1	7.1	5.83	2.41	2.22E-05	0.0001	COX4I1	cytochrome c oxidase subunit IV isoform 1	Multiple_Complex
TC0900009950.hg.1	11.87	9.74	4.38	2.23E-05	0.0001	TLN1; MIR6	talin 1; microRNA 6852	Multiple_Complex
TC0700008626.hg.1	9.1	7.92	2.26	2.23E-05	0.0001	ORAI2	ORAI calcium release-activated calcium modu	Multiple_Complex
TC0100018466.hg.1	5.59	4.17	2.68	2.23E-05	0.0001	DENND2C	DENN/MADD domain containing 2C	Multiple_Complex
TC0900011396.hg.1	10	10.64	-1.56	2.24E-05	0.0001	RAB14	RAB14, member RAS oncogene family	Coding
TC0700011813.hg.1	6.65	7.84	-2.29	2.24E-05	0.0001	BET1	Bet1 golgi vesicular membrane trafficking prot	Multiple_Complex
TC0600009628.hg.1	11.75	10.87	1.84	2.24E-05	0.0001	HECA	hdc homolog, cell cycle regulator	Multiple_Complex
TC0700007392.hg.1	9.83	10.66	-1.78	2.24E-05	0.0001	OGDH	oxoglutarate (alpha-ketoglutarate) dehydroge	Multiple_Complex
TC0600011499.hg.1	6.54	8.57	-4.08	2.25E-05	0.0001	HLA-DQB1	major histocompatibility complex, class II, DQ	Multiple_Complex
TC1900009369.hg.1	8.39	7.72	1.6	2.25E-05	0.0001	PLIN3	perilipin 3	Multiple_Complex
TC1700007993.hg.1	4.85	6.09	-2.38	2.26E-05	0.0001	RUNDC3A	RUN domain containing 3A	Multiple_Complex
TC0700013162.hg.1	9.56	10.46	-1.86	2.26E-05	0.0001	PAXIP1	PAX interacting (with transcription-activation	Multiple_Complex
TC0100014768.hg.1	4.59	5.74	-2.21	2.26E-05	0.0001	MCOLN2	mucolipin 2	Multiple_Complex
TC0200015764.hg.1	9.57	10.48	-1.88	2.29E-05	0.0002	CNPPD1	cyclin Pas1/PHO80 domain containing 1	Multiple_Complex
TC1600007537.hg.1	6.19	5.56	1.55	2.29E-05	0.0002	TGFB1I1	transforming growth factor beta 1 induced tra	Multiple_Complex
TC0100006501.hg.1	5.44	6.27	-1.78	2.29E-05	0.0002	CPTP	ceramide-1-phosphate transfer protein	Multiple_Complex
TC0300010213.hg.1	8.74	9.45	-1.63	2.30E-05	0.0002	RPUSD3	RNA pseudouridylate synthase domain contai	Multiple_Complex
TC1900009838.hg.1	12.34	10.68	3.17	2.30E-05	0.0002	ADGRE2	adhesion G protein-coupled receptor E2	Multiple_Complex
TC0X00008731.hg.1	8.03	8.72	-1.62	2.31E-05	0.0002	VMA21	VMA21 vacuolar H+-ATPase homolog (S. cerev	Multiple_Complex
TC0700008870.hg.1	6.15	4.88	2.41	2.32E-05	0.0002	CAV2	caveolin 2	Multiple_Complex
TC1700008467.hg.1	16.93	14.86	4.18	2.35E-05	0.0002	VMP1; MIR	vacuole membrane protein 1; microRNA 21	Multiple_Complex
TC0600010797.hg.1	7.83	6.54	2.44	2.36E-05	0.0002	MAK	male germ cell-associated kinase	Coding
TC1700008378.hg.1	7.11	8.21	-2.14	2.36E-05	0.0002	MSI2	musashi RNA binding protein 2	Multiple_Complex
TC1200011946.hg.1	8.48	7.6	1.83	2.36E-05	0.0002	BRAP	BRCA1 associated protein	Multiple_Complex
TC0700006781.hg.1	5.54	6.41	-1.83	2.37E-05	0.0002	BZW2	basic leucine zipper and W2 domains 2	Multiple_Complex
TC0100010284.hg.1	8.24	9.28	-2.05	2.37E-05	0.0002	PEA15	phosphoprotein enriched in astrocytes 15	Multiple_Complex
TC0400011203.hg.1	9.12	7.66	2.75	2.38E-05	0.0002	HPSE	heparanase	Multiple_Complex
TC2000007900.hg.1	9.68	10.76	-2.12	2.40E-05	0.0002	VAPB	VAMP (vesicle-associated membrane protein)	Multiple_Complex
TC1300009895.hg.1	6.5	7.29	-1.73	2.40E-05	0.0002	PCID2	PCI domain containing 2	Multiple_Complex
TC0500011655.hg.1	7.56	8.57	-2.01	2.40E-05	0.0002	FBXL17	F-box and leucine-rich repeat protein 17	Multiple_Complex
TC0300007242.hg.1	8.79	9.93	-2.2	2.40E-05	0.0002	SACM1L	SAC1 suppressor of actin mutations 1-like (yea	Multiple_Complex
TC1200006643.hg.1	6.9	8.35	-2.74	2.42E-05	0.0002	PTMS	parathymosin	Multiple_Complex
TC0800010171.hg.1	4.3	3.72	1.49	2.42E-05	0.0002	C8orf86	chromosome 8 open reading frame 86	Coding
TC0100017947.hg.1	5.56	6.54	-1.97	2.42E-05	0.0002	OPN3	opsin 3	Multiple_Complex
TSUnmapped00000183.h	8.08	8.74	-1.58	2.43E-05	0.0002	VPS11	VPS11, CORVET/HOPS core subunit [Source:H	NonCoding
TC0100014743.hg.1	5.25	3.95	2.47	2.44E-05	0.0002	TTLL7	tubulin tyrosine ligase-like family member 7	Multiple_Complex
TC0900012218.hg.1	4.89	5.96	-2.1	2.45E-05	0.0002	KLHL9	kelch-like family member 9	Coding
TC1700006808.hg.1	5.32	6.11	-1.74	2.46E-05	0.0002	RANGRF	RAN guanine nucleotide release factor	Multiple_Complex
TC1200012281.hg.1	7.97	8.84	-1.84	2.46E-05	0.0002	EIF2B1	eukaryotic translation initiation factor 2B, sub	Multiple_Complex
TC1700011171.hg.1	6.63	7.68	-2.06	2.46E-05	0.0002	COX11	COX11 cytochrome c oxidase copper chaperon	Multiple_Complex
TC0900011623.hg.1	5.09	5.67	-1.49	2.48E-05	0.0002	PTGES2	prostaglandin E synthase 2	Multiple_Complex
TC1900010533.hg.1	5.64	6.24	-1.52	2.48E-05	0.0002	ZNF850	zinc finger protein 850	Multiple_Complex
TC1300007164.hg.1	6.98	7.8	-1.77	2.48E-05	0.0002	SETDB2	SET domain, bifurcated 2	Multiple_Complex
TC1700012341.hg.1	8.71	9.54	-1.78	2.50E-05	0.0002	C1QBP	complement component 1, q subcomponent b	Multiple_Complex
TC0100007789.hg.1	8.96	9.77	-1.76	2.51E-05	0.0002	AGO3	argonaute RISC catalytic component 3	Multiple_Complex
TC0200011386.hg.1	8.33	9.39	-2.09	2.52E-05	0.0002	PPP1R7	protein phosphatase 1, regulatory subunit 7	Multiple_Complex
TC0100018298.hg.1	9.08	10.03	-1.93	2.52E-05	0.0002	INTS3	integrator complex subunit 3	Multiple_Complex
TC1600007275.hg.1	9.34	10.24	-1.86	2.53E-05	0.0002	LCMT1	leucine carboxyl methyltransferase 1	Multiple_Complex
TC0300012281.hg.1	9.2	10.22	-2.04	2.54E-05	0.0002	ZXDC	ZXD family zinc finger C	Multiple_Complex
TC1600008971.hg.1	6.27	7.15	-1.84	2.54E-05	0.0002	JMJD8	jumonji domain containing 8	Multiple_Complex
TC0100012242.hg.1	7.58	6.41	2.26	2.55E-05	0.0002	EFCAB2	EF-hand calcium binding domain 2	Multiple_Complex
TC0700013355.hg.1	7.24	5.89	2.55	2.55E-05	0.0002	NME8	NME/NM23 family member 8	Multiple_Complex
TC0500009250.hg.1	11.45	12.54	-2.14	2.55E-05	0.0002	UBLCP1	ubiquitin-like domain containing CTD phospha	Multiple_Complex

TC0700010918.hg.1	6.36	7.33	-1.96	2.55E-05	0.0002	NUDCD3	NudC domain containing 3	Multiple_Complex
TC0200015721.hg.1	8.37	9.27	-1.86	2.56E-05	0.0002	TMBIM1; M	transmembrane BAX inhibitor motif containin	Multiple_Complex
TC1200011968.hg.1	9.11	10.1	-1.99	2.56E-05	0.0002	HECTD4; M	HECT domain containing E3 ubiquitin protein	Multiple_Complex
TC1000011363.hg.1	4.38	4.8	-1.34	2.57E-05	0.0002	RNLS	renalase, FAD-dependent amine oxidase	Multiple_Complex
TC1000006768.hg.1	13.72	14.87	-2.21	2.57E-05	0.0002	CELF2	Transcript Identified by AceView, Entrez Gene	Unassigned
TC0100015337.hg.1	6.06	6.94	-1.84	2.58E-05	0.0002	AP4B1	adaptor-related protein complex 4, beta 1 sub	Multiple_Complex
TC0600013719.hg.1	8.58	7.47	2.16	2.59E-05	0.0002	DYNLT1	dynein, light chain, Tctex-type 1	Multiple_Complex
TC0700007361.hg.1	6.45	7.11	-1.58	2.59E-05	0.0002	UBE2D4	ubiquitin-conjugating enzyme E2D 4 (putative	Multiple_Complex
TC1700006758.hg.1	10	10.86	-1.82	2.60E-05	0.0002	MPDU1	mannose-P-dolichol utilization defect 1	Multiple_Complex
TC1600009530.hg.1	9.91	10.97	-2.09	2.60E-05	0.0002	KIAA0430;	KIAA0430; microRNA 6506	Multiple_Complex
TC1000010411.hg.1	7.45	8.17	-1.65	2.61E-05	0.0002	ZNF33B	zinc finger protein 33B	Multiple_Complex
TC1100011190.hg.1	10.3	9.8	1.41	2.61E-05	0.0002	EHD1	EH domain containing 1	Multiple_Complex
TC0100016129.hg.1	10.23	9.57	1.57	2.62E-05	0.0002	COPA	coatomer protein complex subunit alpha	Multiple_Complex
TC1100011888.hg.1	11.22	12.61	-2.62	2.63E-05	0.0002	CTSC	cathepsin C	Multiple_Complex
TC0400011705.hg.1	6.86	6.02	1.8	2.63E-05	0.0002	C4orf3	chromosome 4 open reading frame 3	Multiple_Complex
TSUnmapped00000459.h	7.39	6.43	1.95	2.64E-05	0.0002	TRAPPC4	trafficking protein particle complex 4	Coding
TC1000009508.hg.1	5.51	4.87	1.56	2.67E-05	0.0002	TUBB8	tubulin, beta 8 class VIII	Multiple_Complex
TC1100013005.hg.1	9.7	9.17	1.44	2.67E-05	0.0002	ZFP91	ZFP91 zinc finger protein	Multiple_Complex
TC0300009337.hg.1	9.46	10.49	-2.03	2.68E-05	0.0002	SMC4	structural maintenance of chromosomes 4	Multiple_Complex
TC0400009048.hg.1	11.4	9.04	5.13	2.68E-05	0.0002	TLR2	toll-like receptor 2	Multiple_Complex
TC1000007226.hg.1	9.17	7.57	3.02	2.68E-05	0.0002	CCDC7	coiled-coil domain containing 7	Multiple_Complex
TC1500009984.hg.1	11.55	10.4	2.22	2.69E-05	0.0002	NPTN	neuroplastin	Multiple_Complex
TC1600007448.hg.1	15.16	17.1	-3.84	2.69E-05	0.0002	CORO1A	coronin, actin binding protein, 1A	Multiple_Complex
TC0700006603.hg.1	6.3	6.99	-1.62	2.70E-05	0.0002	FOXK1	forkhead box K1	Multiple_Complex
TC1100011010.hg.1	5.92	6.85	-1.91	2.70E-05	0.0002	PRPF19	pre-mRNA processing factor 19	Multiple_Complex
TC0X00009986.hg.1	4.75	5.4	-1.57	2.71E-05	0.0002	RP5-1091N	Transcript Identified by AceView, Entrez Gene	NonCoding
TC1000008315.hg.1	5.11	6.28	-2.25	2.71E-05	0.0002	BMPR1A	bone morphogenetic protein receptor type IA	Multiple_Complex
TC0400009458.hg.1	6.23	7.13	-1.88	2.72E-05	0.0002	CDKN2AIP	CDKN2A interacting protein	Multiple_Complex
TC0800011000.hg.1	5.1	4.42	1.6	2.72E-05	0.0002	REXO1L1P;	REX1, RNA exonuclease 1 homolog-like 1, pse	Multiple_Complex
TC0100015971.hg.1	13.7	14.53	-1.77	2.73E-05	0.0002	GON4L	gon-4-like (C. elegans)	Multiple_Complex
TC0500013130.hg.1	6.47	7.26	-1.73	2.74E-05	0.0002	TRIM52	tripartite motif containing 52	Multiple_Complex
TC0400008335.hg.1	8.26	7.08	2.27	2.75E-05	0.0002	AIMP1	aminoacyl tRNA synthetase complex-interacti	Multiple_Complex
TC1600007392.hg.1	7.75	8.7	-1.94	2.75E-05	0.0002	SNX29P2; S	sorting nexin 29 pseudogene 2; sorting nexin	Multiple_Complex
TC1700012457.hg.1	4.64	5.71	-2.1	2.76E-05	0.0002	AXIN2	axin 2	Multiple_Complex
TC1500008995.hg.1	8.67	10.11	-2.73	2.77E-05	0.0002	GOLGA8B	golgin A8 family, member B	Multiple_Complex
TC0200013308.hg.1	6.48	7.54	-2.08	2.77E-05	0.0002	IMMT	inner membrane protein, mitochondrial	Multiple_Complex
TC1100010739.hg.1	6.31	5.64	1.59	2.79E-05	0.0002	RAPSN	receptor-associated protein of the synapse	Multiple_Complex
TC1800007080.hg.1	7.34	6.63	1.64	2.79E-05	0.0002	GALNT1	polypeptide N-acetylgalactosaminyltransferas	Multiple_Complex
TC0400006812.hg.1	5.89	5.07	1.77	2.79E-05	0.0002	USP17L24;	ubiquitin specific peptidase 17-like family mer	Coding
TC0400006814.hg.1	5.89	5.07	1.77	2.79E-05	0.0002	USP17L25;	ubiquitin specific peptidase 17-like family mer	Coding
TC2200008103.hg.1	5.88	6.62	-1.67	2.83E-05	0.0002	YPEL1	yippee like 1	Multiple_Complex
TC1700009277.hg.1	6.48	7.34	-1.82	2.85E-05	0.0002	TBCD	tubulin folding cofactor D	Multiple_Complex
TC0900012181.hg.1	5.81	6.59	-1.72	2.87E-05	0.0002	NUP188	nucleoporin 188kDa	Multiple_Complex
TC1000010930.hg.1	4.83	5.68	-1.81	2.87E-05	0.0002	PCBD1	pterin-4 alpha-carbinolamine dehydratase/dir	Multiple_Complex
TC0500007494.hg.1	4.55	3.95	1.52	2.89E-05	0.0002	RAB3C	RAB3C, member RAS oncogene family	Multiple_Complex
TC0500007115.hg.1	7.96	6.97	1.98	2.89E-05	0.0002	RAI14	Transcript Identified by AceView, Entrez Gene	Unassigned
TC0100010197.hg.1	6.61	6	1.52	2.90E-05	0.0002	PEAR1	platelet endothelial aggregation receptor 1	Multiple_Complex
TC2000008721.hg.1	11.51	9.84	3.18	2.90E-05	0.0002	APMAP	adipocyte plasma membrane associated prote	Multiple_Complex
TC0200007717.hg.1	9.13	10.47	-2.53	2.90E-05	0.0002	AHSA2	AHA1, activator of heat shock 90kDa protein A	Multiple_Complex
TC1200010566.hg.1	8.96	9.77	-1.75	2.90E-05	0.0002	SENP1	SUMO1/sentrin specific peptidase 1	Multiple_Complex
TC1200009071.hg.1	5.9	6.72	-1.77	2.91E-05	0.0002	PEBP1	phosphatidylethanolamine binding protein 1	Multiple_Complex
TC0100017836.hg.1	15.06	13.85	2.32	2.91E-05	0.0002	LYST	lysosomal trafficking regulator	Multiple_Complex
TC0800009673.hg.1	7.3	6.31	2	2.94E-05	0.0002	LONRF1	LON peptidase N-terminal domain and ring fir	Multiple_Complex
TC0500009353.hg.1	7.34	6.34	2	2.95E-05	0.0002	RARS	arginyl-tRNA synthetase	Multiple_Complex
TC1800007667.hg.1	6.63	7.41	-1.71	2.96E-05	0.0002	TIMM21	translocase of inner mitochondrial membrane	Multiple_Complex
TC0100015747.hg.1	6.78	7.55	-1.7	2.96E-05	0.0002	GOLPH3L	golgi phosphoprotein 3-like	Multiple_Complex
TC0800007459.hg.1	6.89	7.47	-1.49	2.96E-05	0.0002	POMK	protein-O-mannose kinase	Multiple_Complex
TC0400011207.hg.1	6.69	7.52	-1.78	2.97E-05	0.0002	HELQ	helicase, POLQ-like	Multiple_Complex
TC0300014001.hg.1	6.81	7.89	-2.1	2.97E-05	0.0002	RFT1	RFT1 homolog	Multiple_Complex
TC1200012199.hg.1	9.82	8.46	2.57	2.98E-05	0.0002	CAMKK2	calcium/calmodulin-dependent protein kinase	Multiple_Complex
TC0500007927.hg.1	4.19	5.14	-1.93	2.98E-05	0.0002	RASGRF2	Jeck2013 ALT_ACCEPTOR, ALT_DONOR, codin	NonCoding
TC0900009825.hg.1	9.75	11.98	-4.7	2.98E-05	0.0002	DDX58	DEAD (Asp-Glu-Ala-Asp) box polypeptide 58	Multiple_Complex
TSUnmapped00000056.h	7.09	6.2	1.86	2.98E-05	0.0002	TRAPPC4	trafficking protein particle complex 4	Coding
TC0200006674.hg.1	8.44	7.64	1.73	3.02E-05	0.0002	KLF11	Kruppel-like factor 11	Multiple_Complex
TC0100010926.hg.1	6.75	8.15	-2.62	3.05E-05	0.0002	OCLM	Transcript Identified by AceView, Entrez Gene	Unassigned
TC0200013772.hg.1	8.28	7.42	1.82	3.05E-05	0.0002	RGPD3	RANBP2-like and GRIP domain containing 3	Coding
TSUnmapped00000075.h	5.12	6.13	-2.02	3.06E-05	0.0002	ZNF780A	zinc finger protein 780A	Coding
TC0100015598.hg.1	17.83	18.58	-1.68	3.06E-05	0.0002	TXNIP	thioredoxin interacting protein	Multiple_Complex
TC1900009479.hg.1	6.51	5.99	1.43	3.07E-05	0.0002	PEX11G	peroxisomal biogenesis factor 11 gamma	Multiple_Complex
TC1900011132.hg.1	5.11	5.9	-1.74	3.07E-05	0.0002	SLC6A16	solute carrier family 6, member 16	Multiple_Complex

TC0100011855.hg.1	10.01	11.73	-3.3	3.07E-05	0.0002	GUK1	guanylate kinase 1	Multiple_Complex
TSUnmapped00000494.h	9.11	8.23	1.85	3.08E-05	0.0002	ZDHHC3	zinc finger, DHHC-type containing 3	Coding
TC1000006434.hg.1	6.7	7.96	-2.39	3.09E-05	0.0002	ZMYND11	zinc finger, MYND-type containing 11	Multiple_Complex
TC0200008260.hg.1	7.91	7.06	1.81	3.09E-05	0.0002	VAMP8	vesicle associated membrane protein 8	Multiple_Complex
TC0400010688.hg.1	5.64	6.41	-1.7	3.09E-05	0.0002	USP46	ubiquitin specific peptidase 46	Multiple_Complex
TC0X00010331.hg.1	5.63	4.66	1.95	3.09E-05	0.0002	SYTL4	synaptotagmin-like 4	Multiple_Complex
TC1100007978.hg.1	11.55	12.39	-1.79	3.09E-05	0.0002	MEN1	Jeck2013 ANTISENSE, CDS, coding, INTERNAL,	NonCoding
TC0600014300.hg.1	7.42	8.23	-1.76	3.09E-05	0.0002	GCLC	glutamate-cysteine ligase, catalytic subunit	Multiple_Complex
TC0900009458.hg.1	4.45	5.51	-2.09	3.10E-05	0.0002	RLN1	relaxin 1	Multiple_Complex
TC1100010987.hg.1	7.17	6.42	1.68	3.10E-05	0.0002	FABP5	fatty acid binding protein 5 (psoriasis-associat	Multiple_Complex
TC1200012003.hg.1	7.74	8.3	-1.48	3.10E-05	0.0002	RBM19	RNA binding motif protein 19	Multiple_Complex
TC1700011298.hg.1	6.74	7.77	-2.05	3.12E-05	0.0002	TUBD1	tubulin, delta 1	Multiple_Complex
TC0600014123.hg.1	9.18	8.07	2.16	3.13E-05	0.0002	NFYA	nuclear transcription factor Y subunit alpha	Multiple_Complex
TC0900009932.hg.1	7.57	8.26	-1.61	3.14E-05	0.0002	STOML2	stomatin (EPB72)-like 2	Multiple_Complex
TC0100009944.hg.1	9.19	9.93	-1.67	3.15E-05	0.0002	PIP5K1A	phosphatidylinositol-4-phosphate 5-kinase, ty	Multiple_Complex
TC0100013692.hg.1	9.7	10.64	-1.91	3.16E-05	0.0002	KIAA0319L	KIAA0319-like	Multiple_Complex
TC0700009236.hg.1	7.13	6.06	2.11	3.21E-05	0.0002	CALD1	caldesmon 1	Multiple_Complex
TC0900012153.hg.1	6.53	5.9	1.55	3.21E-05	0.0002	MSANTD3	Myb/SANT-like DNA-binding domain containir	Multiple_Complex
TC0400011313.hg.1	11.52	13.69	-4.51	3.21E-05	0.0002	SNCA	synuclein alpha	Multiple_Complex
TC0600007855.hg.1	7.77	8.78	-2.01	3.21E-05	0.0002	FGD2	FYVE, RhoGEF and PH domain containing 2	Multiple_Complex
TC1400006970.hg.1	11.88	10.95	1.91	3.22E-05	0.0002	PNN	pinin, desmosome associated protein	Multiple_Complex
TC0200010443.hg.1	17.94	16.72	2.32	3.25E-05	0.0002	CFLAR	CASP8 and FADD like apoptosis regulator	Multiple_Complex
TC1900011632.hg.1	5.47	6.27	-1.74	3.25E-05	0.0002	ZBTB45	zinc finger and BTB domain containing 45	Multiple_Complex
TC2200009194.hg.1	12.67	13.65	-1.98	3.25E-05	0.0002	MED15	mediator complex subunit 15	Multiple_Complex
TC0200011188.hg.1	5.06	6.13	-2.09	3.26E-05	0.0002	AGAP1	ArfGAP with GTPase domain, ankyrin repeat a	Multiple_Complex
TC0X00010356.hg.1	9.19	8.29	1.86	3.27E-05	0.0002	BTK	Bruton agammaglobulinemia tyrosine kinase	Multiple_Complex
TC0100007660.hg.1	5.83	6.93	-2.13	3.28E-05	0.0002	TMEM39B	transmembrane protein 39B	Multiple_Complex
TC1000009092.hg.1	4.66	5.78	-2.17	3.29E-05	0.0002	INPP5F	inositol polyphosphate-5-phosphatase F	Multiple_Complex
TC1300006658.hg.1	6.14	4.85	2.46	3.31E-05	0.0002	WASF3	WAS protein family, member 3	Multiple_Complex
TC0600009370.hg.1	6.01	6.63	-1.54	3.32E-05	0.0002	TRMT11	tRNA methyltransferase 11 homolog	Multiple_Complex
TC0100011406.hg.1	18.38	17.64	1.67	3.33E-05	0.0002	CD55	CD55 molecule, decay accelerating factor for (	Multiple_Complex
TC1600008330.hg.1	6.49	5.22	2.42	3.34E-05	0.0002	HPR	haptoglobin-related protein	Multiple_Complex
TC1600008214.hg.1	4.53	5.09	-1.48	3.34E-05	0.0002	TANGO6	transport and golgi organization 6 homolog	Multiple_Complex
TC1200011711.hg.1	8.84	9.97	-2.19	3.34E-05	0.0002	GNPTAB	N-acetylglucosamine-1-phosphate transferase	Multiple_Complex
TC0900010910.hg.1	8.74	7.9	1.8	3.36E-05	0.0002	SLC35D2	solute carrier family 35 (UDP-GlcNAc/UDP-glu	Multiple_Complex
TC1100007184.hg.1	5.16	6.04	-1.84	3.37E-05	0.0002	DNAJC24	DnaJ (Hsp40) homolog, subfamily C, member	Multiple_Complex
TC2000009648.hg.1	8.73	7.98	1.68	3.37E-05	0.0002	PRELID3B;	PRELI domain containing 3B; ATP synthase, H+	Multiple_Complex
TC0300006477.hg.1	6.81	7.62	-1.75	3.38E-05	0.0002	ITPR1	inositol 1,4,5-trisphosphate receptor, type 1	Multiple_Complex
TC1200007137.hg.1	9.57	8.38	2.27	3.38E-05	0.0002	FGFR1OP2	FGFR1 oncogene partner 2	Multiple_Complex
TC1900011012.hg.1	6.97	7.64	-1.59	3.39E-05	0.0002	TMEM160	transmembrane protein 160	Multiple_Complex
TC0900008858.hg.1	3.74	4.29	-1.46	3.40E-05	0.0002	COQ4	coenzyme Q4	Multiple_Complex
TC0100006707.hg.1	10.81	9.82	1.98	3.41E-05	0.0002	CAMTA1	calmodulin binding transcription activator 1	Multiple_Complex
TC0200010431.hg.1	6.18	6.99	-1.75	3.43E-05	0.0002	NIF3L1	NIF3 NGG1 interacting factor 3-like 1	Multiple_Complex
TSUnmapped00000291.h	7.57	6.96	1.52	3.43E-05	0.0002	VPS11	VPS11, CORVET/HOPS core subunit [Source:H	Coding
TC0700013550.hg.1	6.13	7.03	-1.86	3.44E-05	0.0002	URGCP; UR	upregulator of cell proliferation; URGCP-MRPS	Multiple_Complex
TC0200009548.hg.1	9.04	8.17	1.82	3.45E-05	0.0002	ARHGAP15	Rho GTPase activating protein 15	NonCoding
TC0400012366.hg.1	8.46	11.57	-8.62	3.46E-05	0.0002	DDX60	DEAD (Asp-Glu-Ala-Asp) box polypeptide 60	Multiple_Complex
TC0100009482.hg.1	8.55	7.22	2.51	3.46E-05	0.0002	HIPK1	Memczak2013 ALT_DONOR, coding, INTERNA	NonCoding
TC1100009048.hg.1	5.66	6.34	-1.6	3.47E-05	0.0002	DLAT	dihydrolipoamide S-acetyltransferase	Multiple_Complex
TC2100008526.hg.1	5.59	4.82	1.71	3.47E-05	0.0002	SLC37A1	solute carrier family 37 (glucose-6-phosphate	Multiple_Complex
TC0300012143.hg.1	7.41	8.33	-1.89	3.47E-05	0.0002	LRRC58	leucine rich repeat containing 58	Multiple_Complex
TC0X00010366.hg.1	6.75	5.75	2.01	3.47E-05	0.0002	ARMCX6	armadillo repeat containing, X-linked 6	Multiple_Complex
TC1100008133.hg.1	8.03	8.93	-1.86	3.48E-05	0.0002	TBC1D10C	TBC1 domain family, member 10C	Multiple_Complex
TC0300011182.hg.1	7.73	8.63	-1.87	3.49E-05	0.0002	GLT8D1	glycosyltransferase 8 domain containing 1	Multiple_Complex
TC0100015743.hg.1	9.8	8.73	2.09	3.52E-05	0.0002	ENSA	endosulfine alpha	Multiple_Complex
TC0200007102.hg.1	8.87	7.88	1.98	3.52E-05	0.0002	PLB1	phospholipase B1	Multiple_Complex
TC0700008252.hg.1	6.21	7.31	-2.14	3.53E-05	0.0002	CROT	carnitine O-octanoyltransferase	Multiple_Complex
TC1700011773.hg.1	12.44	13.81	-2.6	3.55E-05	0.0002	WBP2	WW domain binding protein 2	Multiple_Complex
TC0400006820.hg.1	4.65	3.95	1.62	3.55E-05	0.0002	USP17L27;	ubiquitin specific peptidase 17-like family mer	Coding
TC0400006822.hg.1	4.65	3.95	1.62	3.55E-05	0.0002	USP17L28;	ubiquitin specific peptidase 17-like family mer	Coding
TC0400006824.hg.1	4.65	3.95	1.62	3.55E-05	0.0002	USP17L29;	ubiquitin specific peptidase 17-like family mer	Coding
TC0400006828.hg.1	4.65	3.95	1.62	3.55E-05	0.0002	USP17L25;	ubiquitin specific peptidase 17-like family mer	Coding
TC0400007422.hg.1	6.06	7.02	-1.95	3.55E-05	0.0002	ATP10D	ATPase, class V, type 10D	Multiple_Complex
TC0200009223.hg.1	5.43	6.21	-1.71	3.55E-05	0.0002	SFT2D3	SFT2 domain containing 3	Coding
TC1100007935.hg.1	8.49	9.23	-1.67	3.56E-05	0.0002	STIP1	stress-induced phosphoprotein 1	Multiple_Complex
TC0X00006603.hg.1	7.08	8.03	-1.94	3.56E-05	0.0002	HCCS	holocytochrome c synthase	Multiple_Complex
TC0400007399.hg.1	7.3	8.05	-1.68	3.57E-05	0.0002	GUF1	GUF1 homolog, GTPase	Multiple_Complex
TC0600012655.hg.1	10.8	9.7	2.15	3.57E-05	0.0002	PNISR	PNN-interacting serine/arginine-rich protein	Multiple_Complex
TC0300013602.hg.1	6.17	5.09	2.11	3.58E-05	0.0002	ATP13A3	Transcript Identified by AceView, Entrez Gene	Unassigned
TC0100006822.hg.1	5.95	6.8	-1.8	3.58E-05	0.0002	PEX14	peroxisomal biogenesis factor 14	Multiple_Complex

TC0400012949.hg.1	8.57	7.7	1.83	3.59E-05	0.0002	SLC39A8	solute carrier family 39 (zinc transporter), mer	Multiple_Complex
TC1300009992.hg.1	5.92	6.82	-1.87	3.59E-05	0.0002	BIVM-ERCC	BIVM-ERCC5 readthrough	Multiple_Complex
TC0400011383.hg.1	7.49	9.57	-4.22	3.63E-05	0.0002	TSPAN5	tetraspanin 5	Multiple_Complex
TC1700011795.hg.1	6.16	7.06	-1.87	3.64E-05	0.0002	PRPSAP1	phosphoribosyl pyrophosphate synthetase-as:	Multiple_Complex
TC1300008091.hg.1	5.56	6.68	-2.18	3.65E-05	0.0002	ATP11A	Transcript Identified by AceView, Entrez Gene	Unassigned
TC1900008292.hg.1	5.88	6.57	-1.61	3.65E-05	0.0002	TOMM40	translocase of outer mitochondrial membrane	Multiple_Complex
TC1900008927.hg.1	7.38	8.03	-1.56	3.67E-05	0.0002	EPN1	epsin 1	Multiple_Complex
TC2000009959.hg.1	10.61	11.6	-2	3.69E-05	0.0002	CSNK2A1	casein kinase 2, alpha 1 polypeptide	Multiple_Complex
TSUnmapped00000592.h	4.63	3.82	1.76	3.69E-05	0.0002	FMN1	formin 1	Coding
TC0200012219.hg.1	5.64	6.61	-1.96	3.70E-05	0.0002	FAM98A	family with sequence similarity 98, member A	Multiple_Complex
TC0300011108.hg.1	4.96	5.68	-1.65	3.70E-05	0.0002	NPRL2	NPR2-like, GATOR1 complex subunit	Multiple_Complex
TC0400012551.hg.1	8.22	8.95	-1.65	3.70E-05	0.0002	DCTD	dCMP deaminase	Multiple_Complex
TC1100013082.hg.1	6.24	4.95	2.44	3.72E-05	0.0002	PIWIL4	piwi-like RNA-mediated gene silencing 4	Multiple_Complex
TC1500010244.hg.1	5.15	4.56	1.5	3.72E-05	0.0002	AP3B2	adaptor-related protein complex 3, beta 2 sub	Multiple_Complex
TC1100007800.hg.1	7.85	8.51	-1.58	3.73E-05	0.0002	TKFC	triokinase/FMN cyclase	Multiple_Complex
TC1100008447.hg.1	6.28	6.98	-1.63	3.73E-05	0.0002	SPCS2	signal peptidase complex subunit 2	Multiple_Complex
TC0100013723.hg.1	8.59	7.71	1.84	3.74E-05	0.0002	MRPS15	mitochondrial ribosomal protein S15	Multiple_Complex
TC1300009271.hg.1	6.34	6.98	-1.56	3.75E-05	0.0002	TBC1D4	TBC1 domain family, member 4	Multiple_Complex
TC0100012089.hg.1	7.68	8.81	-2.19	3.75E-05	0.0002	GPR137B	G protein-coupled receptor 137B	Multiple_Complex
TC1800006650.hg.1	15.11	13.08	4.08	3.76E-05	0.0002	RAB31	RAB31, member RAS oncogene family	Multiple_Complex
TC0600014282.hg.1	13.39	10.66	6.61	3.77E-05	0.0002	RPS10	ribosomal protein S10	Multiple_Complex
TSUnmapped00000469.h	6.31	5.23	2.11	3.78E-05	0.0002	INPP5D	inositol polyphosphate-5-phosphatase D	Coding
TC0200009920.hg.1	10.34	8.61	3.32	3.79E-05	0.0002	SSB	Sjogren syndrome antigen B (autoantigen La)	Multiple_Complex
TC1700012460.hg.1	7.63	8.6	-1.95	3.80E-05	0.0002	ABCA5	ATP binding cassette subfamily A member 5	Multiple_Complex
TC0500007163.hg.1	10.24	11.15	-1.88	3.80E-05	0.0002	NIPBL	Nipped-B homolog (Drosophila)	Multiple_Complex
TC1900011084.hg.1	7.54	8.49	-1.94	3.82E-05	0.0002	DBP	D site of albumin promoter (albumin D-box) b	Multiple_Complex
TC0100006991.hg.1	7.95	8.58	-1.55	3.82E-05	0.0002	RSC1A1; DI	regulatory solute carrier protein, family 1, me	Multiple_Complex
TC0300010240.hg.1	7.43	6.81	1.53	3.83E-05	0.0002	SEC13	SEC13 homolog, nuclear pore and COPII coat c	Multiple_Complex
TC0100014775.hg.1	9.98	9.38	1.52	3.83E-05	0.0002	BCL10	B-cell CLL/lymphoma 10	Coding
TC1700011652.hg.1	5.58	6.32	-1.68	3.84E-05	0.0002	FAM104A	family with sequence similarity 104, member .	Multiple_Complex
TC0600009350.hg.1	6.52	5.52	1.99	3.84E-05	0.0002	RNF217	ring finger protein 217	Multiple_Complex
TC1900009583.hg.1	7.21	8.06	-1.8	3.84E-05	0.0002	ZNF266	zinc finger protein 266	Multiple_Complex
TC0300010358.hg.1	7.83	8.71	-1.84	3.89E-05	0.0002	XPC	xeroderma pigmentosum, complementation g	Multiple_Complex
TC1200008107.hg.1	14.09	12.93	2.25	3.89E-05	0.0002	RAP1B	RAP1B, member of RAS oncogene family	Multiple_Complex
TC0200016676.hg.1	5.12	5.98	-1.82	3.90E-05	0.0002	WDPCP	WD repeat containing planar cell polarity effe	Multiple_Complex
TC0300007512.hg.1	12.3	11.13	2.25	3.92E-05	0.0002	PRKCD	protein kinase C, delta	Multiple_Complex
TSUnmapped00000208.h	5.19	6.13	-1.92	3.94E-05	0.0002	ZDHHC3	zinc finger, DHHC-type containing 3	NonCoding
TC1600009102.hg.1	8.96	9.87	-1.87	3.95E-05	0.0002	RNPS1	RNA binding protein S1, serine-rich domain	Multiple_Complex
TSUnmapped00000351.h	5.75	6.58	-1.79	3.96E-05	0.0002	VPS11	VPS11, CORVET/HOPS core subunit [Source:H	NonCoding
TC0300011423.hg.1	6.48	7.42	-1.93	3.99E-05	0.0002	LRIG1	leucine-rich repeats and immunoglobulin-like	Multiple_Complex
TC0400007330.hg.1	6.91	5.42	2.8	4.00E-05	0.0002	NSUN7	NOP2/Sun domain family, member 7	Multiple_Complex
TC1700012039.hg.1	7.29	6.27	2.02	4.01E-05	0.0002	LOC10013C	uncharacterized LOC100130370; novel transc	Multiple_Complex
TC1500008986.hg.1	5.26	6.09	-1.78	4.01E-05	0.0002	LPCAT4	lysophosphatidylcholine acyltransferase 4	Multiple_Complex
TC0800011561.hg.1	12.23	13.42	-2.27	4.02E-05	0.0002	EIF3H	eukaryotic translation initiation factor 3, subu	Multiple_Complex
TC0100015790.hg.1	5.49	4.9	1.5	4.03E-05	0.0002	POGZ	Transcript Identified by AceView, Entrez Gene	Unassigned
TC1300008267.hg.1	7.51	8.55	-2.07	4.04E-05	0.0002	XPO4	exportin 4	Multiple_Complex
TC1900007270.hg.1	11.24	12.95	-3.27	4.04E-05	0.0002	KLF2	Kruppel-like factor 2	Coding
TC1900009850.hg.1	6.7	5.84	1.82	4.05E-05	0.0003	SLC1A6	solute carrier family 1 (high affinity aspartate/	Multiple_Complex
TC0800012393.hg.1	5.47	4.58	1.85	4.06E-05	0.0003	ADRA1A	adrenoceptor alpha 1A	Multiple_Complex
TC0100014050.hg.1	7.94	8.63	-1.61	4.07E-05	0.0003	LRRC41	leucine rich repeat containing 41	Multiple_Complex
TC0800007245.hg.1	6.17	7.06	-1.86	4.07E-05	0.0003	MAK16	MAK16 homolog	Multiple_Complex
TC0200007722.hg.1	8.2	6.7	2.83	4.07E-05	0.0003	USP34	Memczak2013 ANTISENSE, CDS, coding, INTEF	NonCoding
TC1600008942.hg.1	6.5	5.63	1.83	4.08E-05	0.0003	MRPL28	mitochondrial ribosomal protein L28	Multiple_Complex
TC0200010796.hg.1	8.95	10.01	-2.09	4.10E-05	0.0003	CTDSP1	CTD small phosphatase 1	Multiple_Complex
TC2000008909.hg.1	9.96	9.24	1.65	4.11E-05	0.0003	EIF2S2	eukaryotic translation initiation factor 2, subu	Multiple_Complex
TC0X00009549.hg.1	6.91	7.95	-2.06	4.13E-05	0.0003	NDUFB11	NADH dehydrogenase (ubiquinone) 1 beta sul	Multiple_Complex
TC0500008919.hg.1	8.29	9.11	-1.76	4.14E-05	0.0003	RNF14	ring finger protein 14	Multiple_Complex
TC1200006640.hg.1	5.15	5.88	-1.66	4.16E-05	0.0003	COPS7A	COP9 signalosome subunit 7A	Multiple_Complex
TC2000006464.hg.1	7.18	8.27	-2.14	4.17E-05	0.0003	FAM110A	family with sequence similarity 110, member .	Multiple_Complex
TC0500012057.hg.1	8.37	9.26	-1.85	4.17E-05	0.0003	C5orf15	chromosome 5 open reading frame 15	Multiple_Complex
TC0100010078.hg.1	9.79	11.38	-3	4.18E-05	0.0003	IL6R	interleukin 6 receptor	Multiple_Complex
TC1600010244.hg.1	4.58	3.99	1.51	4.21E-05	0.0003	ZNF423	zinc finger protein 423	Multiple_Complex
TC0400007799.hg.1	4.53	5.33	-1.74	4.21E-05	0.0003	SLC4A4	solute carrier family 4 (sodium bicarbonate co	Multiple_Complex
TC0200010907.hg.1	8.76	7.73	2.04	4.21E-05	0.0003	ACSL3	acyl-CoA synthetase long-chain family membe	Multiple_Complex
TC1100013221.hg.1	11.6	10.2	2.63	4.22E-05	0.0003	CASP1	caspase 1	Multiple_Complex
TC1900007950.hg.1	5.74	6.96	-2.33	4.24E-05	0.0003	ZNF567	zinc finger protein 567	Multiple_Complex
TC1900009433.hg.1	7.29	6.51	1.72	4.25E-05	0.0003	TUBB4A	tubulin, beta 4A class IVa	Coding
TC1100008518.hg.1	7.82	6.93	1.86	4.25E-05	0.0003	ACER3	alkaline ceramidase 3	Multiple_Complex
TSUnmapped00000282.h	7.03	8.11	-2.11	4.28E-05	0.0003	KAT6B	K(lysine) acetyltransferase 6B	Coding
TC1600009422.hg.1	9.78	11.59	-3.52	4.30E-05	0.0003	GSPT1	G1 to S phase transition 1	Multiple_Complex

TC0400010768.hg.1	4.69	5.65	-1.96	4.30E-05	0.0003	AASDH	aminoadipate-semialdehyde dehydrogenase	Multiple_Complex
TC2200007309.hg.1	9.35	10.43	-2.11	4.30E-05	0.0003	SH3BP1; PI	SH3-domain binding protein 1; pyridoxal (pyri	Multiple_Complex
TC0100014806.hg.1	12.61	11.49	2.19	4.31E-05	0.0003	15-Sep	15 kDa selenoprotein; Salzman2013 ANNOTAT	Multiple_Complex
TC1900011936.hg.1	4	4.71	-1.63	4.31E-05	0.0003	ZNF781	zinc finger protein 781	Multiple_Complex
TC1200007895.hg.1	13.43	12.42	2.02	4.31E-05	0.0003	OS9	osteosarcoma amplified 9, endoplasmic reticu	Multiple_Complex
TC0100010510.hg.1	8.07	8.82	-1.68	4.32E-05	0.0003	RCSD1	RCSD domain containing 1	Multiple_Complex
TC1500010720.hg.1	4.84	5.59	-1.68	4.32E-05	0.0003	IVD	isovaleryl-CoA dehydrogenase	Multiple_Complex
TC1900012036.hg.1	10.27	9.02	2.38	4.34E-05	0.0003	LILRB3	leukocyte immunoglobulin-like receptor, subf	Multiple_Complex
TC0900012197.hg.1	5.3	6.35	-2.07	4.34E-05	0.0003	PTGDS	prostaglandin D2 synthase 21kDa (brain)	Multiple_Complex
TC0X00007107.hg.1	5.26	6.2	-1.92	4.36E-05	0.0003	KRBOX4	KRAB box domain containing 4	Multiple_Complex
TC1600009198.hg.1	5.78	6.57	-1.72	4.38E-05	0.0003	NLRC3	NLR family, CARD domain containing 3	Multiple_Complex
TC2000009986.hg.1	8.93	9.65	-1.64	4.39E-05	0.0003	SNX5	sorting nexin 5	Multiple_Complex
TC0900012209.hg.1	7.25	8.24	-1.99	4.40E-05	0.0003	TTC39B	tetratricopeptide repeat domain 39B	Multiple_Complex
TC1000006907.hg.1	8.88	9.82	-1.92	4.44E-05	0.0003	STAM	Memczak2013 ALT_ACCEPTOR, ALT_DONOR,	NonCoding
TC0200011075.hg.1	14.86	16.04	-2.26	4.46E-05	0.0003	PTMA	prothymosin, alpha	Multiple_Complex
TC0100014392.hg.1	9.04	9.74	-1.63	4.46E-05	0.0003	TM2D1	TM2 domain containing 1	Multiple_Complex
TC0700008809.hg.1	6.4	7.67	-2.42	4.46E-05	0.0003	LRRN3	leucine rich repeat neuronal 3	Multiple_Complex
TC1900011667.hg.1	7.54	8.4	-1.82	4.49E-05	0.0003	ZNF700	zinc finger protein 700	Multiple_Complex
TC0600008092.hg.1	6.87	7.71	-1.79	4.51E-05	0.0003	TJAP1	tight junction associated protein 1 (peripheral	Multiple_Complex
TSUnmapped00000524.h	5.66	6.66	-2	4.52E-05	0.0003	INPP5D	inositol polyphosphate-5-phosphatase D	Coding
TC2200009277.hg.1	4.51	5.66	-2.21	4.53E-05	0.0003	XPNPEP3	X-prolyl aminopeptidase 3, mitochondrial	Multiple_Complex
TC1000008668.hg.1	6.5	7.64	-2.19	4.54E-05	0.0003	SFXN3	sideroflexin 3	Multiple_Complex
TC1900011642.hg.1	6.44	7.09	-1.56	4.55E-05	0.0003	SPPL2B	signal peptide peptidase like 2B	Multiple_Complex
TC2200009285.hg.1	4.7	5.57	-1.82	4.55E-05	0.0003	PRR5-ARHC	PRR5-ARHGAP8 readthrough	Multiple_Complex
TC0600008697.hg.1	7.33	8.97	-3.12	4.55E-05	0.0003	NT5E	5-nucleotidase, ecto (CD73)	Multiple_Complex
TC1200010699.hg.1	5.9	6.73	-1.78	4.55E-05	0.0003	POU6F1	POU class 6 homeobox 1	Multiple_Complex
TC0900008967.hg.1	5.33	6.06	-1.65	4.56E-05	0.0003	EXOSC2	exosome component 2	Multiple_Complex
TC0300006582.hg.1	6.61	7.57	-1.94	4.58E-05	0.0003	TATDN2; G	TatD DNase domain containing 2; ghrelin opp	Multiple_Complex
TC0900011837.hg.1	4.44	3.76	1.6	4.59E-05	0.0003	ABO	ABO blood group (transferase A, alpha 1-3-N-a	Multiple_Complex
TC1500010729.hg.1	11.68	10.52	2.24	4.61E-05	0.0003	SERF2	small EDRK-rich factor 2	Multiple_Complex
TC1900011968.hg.1	8.25	9.06	-1.75	4.66E-05	0.0003	XRCC1	X-ray repair complementing defective repair i	Multiple_Complex
TC0200008094.hg.1	4.98	5.69	-1.63	4.67E-05	0.0003	SEMA4F	sema domain, immunoglobulin domain (Ig), tr	Multiple_Complex
TC0200016416.hg.1	7.9	8.59	-1.62	4.67E-05	0.0003	SLC35F6	solute carrier family 35, member F6	Multiple_Complex
TC1100007922.hg.1	6.04	6.82	-1.72	4.68E-05	0.0003	NAA40	N(alpha)-acetyltransferase 40, NatD catalytic s	Multiple_Complex
TC0700009585.hg.1	7.85	9.08	-2.35	4.69E-05	0.0003	CUL1	cullin 1	Multiple_Complex
TC1600011330.hg.1	14.18	15.64	-2.74	4.70E-05	0.0003	ATP6V0C	ATPase, H+ transporting, lysosomal 16kDa, V0	Multiple_Complex
TC1200009587.hg.1	5.82	6.61	-1.72	4.71E-05	0.0003	CACNA2D4	calcium channel, voltage-dependent, alpha 2/	Multiple_Complex
TC0100012889.hg.1	7.41	6.99	1.34	4.72E-05	0.0003	MAD2L2	MAD2 mitotic arrest deficient-like 2 (yeast)	Multiple_Complex
TC0300006596.hg.1	5.69	4.87	1.76	4.72E-05	0.0003	SLC6A11	solute carrier family 6 (neurotransmitter trans	Multiple_Complex
TC1700010248.hg.1	8.1	8.83	-1.66	4.73E-05	0.0003	GIT1	G protein-coupled receptor kinase interacting	Multiple_Complex
TC1900007003.hg.1	7.88	9.03	-2.22	4.75E-05	0.0003	CARM1	coactivator-associated arginine methyltransfe	Multiple_Complex
TC1900007319.hg.1	6.84	7.61	-1.7	4.75E-05	0.0003	MRPL34	mitochondrial ribosomal protein L34	Multiple_Complex
TC0300013859.hg.1	4.85	6.15	-2.45	4.76E-05	0.0003	CD200	CD200 molecule	Multiple_Complex
TSUnmapped00000206.h	6.03	6.68	-1.56	4.82E-05	0.0003	ZNF780B	zinc finger protein 780B	NonCoding
TC0400011518.hg.1	9.17	7.86	2.48	4.82E-05	0.0003	INTS12	integrator complex subunit 12	Multiple_Complex
TC1500010871.hg.1	8.64	9.64	-2.01	4.82E-05	0.0003	ZNF280D	zinc finger protein 280D	Multiple_Complex
TC1100007408.hg.1	3.4	4.14	-1.67	4.83E-05	0.0003	PRDM11	PR domain containing 11	Multiple_Complex
TC1100006923.hg.1	9.4	10.36	-1.95	4.83E-05	0.0003	PDE3B	phosphodiesterase 3B, cGMP-inhibited	Multiple_Complex
TC1000011548.hg.1	4.86	3.71	2.23	4.85E-05	0.0003	RRP12	Zhang2013 ALT_ACCEPTOR, ALT_DONOR, cod	NonCoding
TC1700010828.hg.1	5.61	5.1	1.43	4.86E-05	0.0003	FAM171A2	family with sequence similarity 171, member	Multiple_Complex
TC1500007671.hg.1	10	10.72	-1.65	4.86E-05	0.0003	FEM1B	fem-1 homolog b (C. elegans)	Coding
TC1200007891.hg.1	5.87	6.59	-1.64	4.87E-05	0.0003	DTX3	deltex 3, E3 ubiquitin ligase	Multiple_Complex
TC0200016366.hg.1	6.21	7.09	-1.85	4.88E-05	0.0003	THAP4	THAP domain containing 4	Multiple_Complex
TC1900008057.hg.1	16.3	15.04	2.4	4.90E-05	0.0003	ZFP36	ZFP36 ring finger protein	Coding
TC0400006712.hg.1	9.98	9.04	1.92	4.90E-05	0.0003	TADA2B	transcriptional adaptor 2B	Multiple_Complex
TC1800009214.hg.1	8.31	7.18	2.19	4.90E-05	0.0003	NDUFV2	NADH dehydrogenase (ubiquinone) flavoprot	Multiple_Complex
TC1900009859.hg.1	12.48	13.67	-2.29	4.91E-05	0.0003	BRD4	bromodomain containing 4	Multiple_Complex
TC0X00007116.hg.1	12.26	11.27	1.98	4.93E-05	0.0003	RP2	retinitis pigmentosa 2 (X-linked recessive)	Multiple_Complex
TC1100007030.hg.1	6.63	5.68	1.93	4.95E-05	0.0003	NAV2	neuron navigator 2	Multiple_Complex
TC1100010063.hg.1	7.74	7	1.67	4.96E-05	0.0003	TMEM41B	transmembrane protein 41B	Multiple_Complex
TC2000007329.hg.1	4.93	5.63	-1.62	4.96E-05	0.0003	ACTR5	ARP5 actin-related protein 5 homolog (yeast)	Multiple_Complex
TSUnmapped00000440.h	6.51	7.53	-2.04	4.98E-05	0.0003	HYOU1	hypoxia up-regulated 1	Coding
TC0200013610.hg.1	7.47	7.92	-1.37	4.99E-05	0.0003	REV1	REV1, DNA directed polymerase	Multiple_Complex
TC2000009980.hg.1	5.4	4.73	1.59	5.00E-05	0.0003	LINC00654	long intergenic non-protein coding RNA 654	NonCoding
TC1500010128.hg.1	9.23	10.21	-1.97	5.00E-05	0.0003	TBC1D2B	TBC1 domain family, member 2B	Multiple_Complex
TC0600013054.hg.1	12.41	11.33	2.12	5.01E-05	0.0003	SERINC1	serine incorporator 1	Multiple_Complex
TC0700013612.hg.1	7.03	5.9	2.19	5.01E-05	0.0003	NDUFA5	NADH dehydrogenase (ubiquinone) 1 alpha su	Multiple_Complex
TC0600014089.hg.1	7.83	6.99	1.79	5.06E-05	0.0003	PGBD1	piggyBac transposable element derived 1	Multiple_Complex
TC0600011859.hg.1	5.26	5.96	-1.62	5.08E-05	0.0003	MRPL2	mitochondrial ribosomal protein L2	Multiple_Complex
TC1300008708.hg.1	11.07	9.62	2.75	5.09E-05	0.0003	ELF1	Memczak2013 ALT_ACCEPTOR, ALT_DONOR,	NonCoding

TC1600006995.hg.1	9.71	10.33	-1.54	5.10E-05	0.0003	BFAR	bifunctional apoptosis regulator	Multiple_Complex
TC2200009195.hg.1	6.88	6.22	1.58	5.11E-05	0.0003	AIFM3	apoptosis-inducing factor, mitochondrion-asso	Multiple_Complex
TC1500008175.hg.1	3.64	4.49	-1.8	5.12E-05	0.0003	PDE8A	phosphodiesterase 8A	Multiple_Complex
TC0800009856.hg.1	5.9	3.89	4.05	5.13E-05	0.0003	EGR3	early growth response 3	Multiple_Complex
TC1900011926.hg.1	7.66	8.72	-2.08	5.13E-05	0.0003	ZNF91	zinc finger protein 91	Multiple_Complex
TC0500008233.hg.1	6.53	4.97	2.95	5.13E-05	0.0003	C5orf30	chromosome 5 open reading frame 30	Coding
TC0500008437.hg.1	8.43	9.21	-1.71	5.16E-05	0.0003	TNFAIP8	tumor necrosis factor, alpha-induced protein 8	Multiple_Complex
TC0700013323.hg.1	6.14	6.96	-1.76	5.17E-05	0.0003	SUN1	Sad1 and UNC84 domain containing 1	Multiple_Complex
TC0900007096.hg.1	8.25	7.43	1.76	5.17E-05	0.0003	CREB3	cAMP responsive element binding protein 3	Multiple_Complex
TC0600011138.hg.1	7.8	6.89	1.87	5.19E-05	0.0003	HIST1H1D	histone cluster 1, H1d	Multiple_Complex
TC1400007093.hg.1	7.66	6.97	1.61	5.21E-05	0.0003	KLHDC1	kelch domain containing 1	Multiple_Complex
TC0500007465.hg.1	10.89	9.52	2.6	5.23E-05	0.0003	GPBP1	GC-rich promoter binding protein 1	Multiple_Complex
TC0600011067.hg.1	10.54	9.3	2.36	5.23E-05	0.0003	TDP2	tyrosyl-DNA phosphodiesterase 2	Multiple_Complex
TC0800007688.hg.1	16.05	14.94	2.14	5.24E-05	0.0003	LYN	LYN proto-oncogene, Src family tyrosine kinas	Multiple_Complex
TC1500010659.hg.1	9.86	8.78	2.11	5.26E-05	0.0003	SNRPA1	small nuclear ribonucleoprotein polypeptide A	Multiple_Complex
TC1900012056.hg.1	3.56	4.42	-1.81	5.26E-05	0.0003	ZNF772	zinc finger protein 772	Multiple_Complex
TC1600007240.hg.1	13.7	13.06	1.55	5.28E-05	0.0003	PRKCB	protein kinase C, beta	Multiple_Complex
TC0800012371.hg.1	11.78	15.19	-10.63	5.28E-05	0.0003	LY6E	lymphocyte antigen 6 complex, locus E	Multiple_Complex
TC1000007275.hg.1	11.23	10.36	1.83	5.28E-05	0.0003	CCNY	cyclin Y	Multiple_Complex
TC0300013296.hg.1	6.05	6.93	-1.83	5.31E-05	0.0003	DNAJC19	DnaJ (Hsp40) homolog, subfamily C, member	Multiple_Complex
TC1700011783.hg.1	6.88	7.79	-1.88	5.33E-05	0.0003	SRP68	signal recognition particle 68kDa	Multiple_Complex
TC0200013323.hg.1	8.26	10.06	-3.49	5.35E-05	0.0003	CD8A	CD8a molecule	Multiple_Complex
TC1300008289.hg.1	11.21	10.34	1.83	5.36E-05	0.0003	ZDHHC20	zinc finger, DHHC-type containing 20	Multiple_Complex
TC0100014988.hg.1	4.74	3.96	1.71	5.38E-05	0.0003	F3	coagulation factor III (thromboplastin, tissue f	Multiple_Complex
TC0100011847.hg.1	14.33	13.56	1.7	5.40E-05	0.0003	ARF1; MIR3	ADP-ribosylation factor 1; microRNA 3620	Multiple_Complex
TC0200014792.hg.1	5.44	6.98	-2.91	5.41E-05	0.0003	COBLL1	cordon-bleu WH2 repeat protein like 1	Multiple_Complex
TC0200012412.hg.1	4.23	3.53	1.63	5.41E-05	0.0003	C1GALT1C1	C1GALT1-specific chaperone 1 like	Multiple_Complex
TC1600008869.hg.1	7.18	6.38	1.74	5.44E-05	0.0003	ZNF778	zinc finger protein 778	Multiple_Complex
TC0300006791.hg.1	11.1	12.18	-2.11	5.44E-05	0.0003	KAT2B	K(lysine) acetyltransferase 2B	Multiple_Complex
TC1200007769.hg.1	6.34	5.62	1.65	5.44E-05	0.0003	PDE1B	phosphodiesterase 1B, calmodulin-dependent	Multiple_Complex
TC0500013240.hg.1	5.3	4.53	1.71	5.45E-05	0.0003	PCDHB4	protocadherin beta 4	Multiple_Complex
TC0900012042.hg.1	5.83	5.17	1.58	5.48E-05	0.0003	RNF208	ring finger protein 208	Coding
TC0700007630.hg.1	8.69	9.45	-1.69	5.48E-05	0.0003	CCT6A	chaperonin containing TCP1, subunit 6A (zeta	Multiple_Complex
TC0700013548.hg.1	5.41	6.31	-1.87	5.48E-05	0.0003	URGCP	upregulator of cell proliferation	Multiple_Complex
TC1900007850.hg.1	7.17	8.86	-3.24	5.49E-05	0.0003	CD22; MIR5	CD22 molecule; microRNA 5196	Multiple_Complex
TC0300014075.hg.1	7.35	8.41	-2.08	5.51E-05	0.0003	TRIM59	tripartite motif containing 59	Coding
TC1600008979.hg.1	4.03	4.79	-1.7	5.51E-05	0.0003	NARFL	nuclear prelamin A recognition factor-like	Multiple_Complex
TC0400012126.hg.1	5.62	6.18	-1.47	5.56E-05	0.0003	GATB	glutamyl-tRNA(Gln) amidotransferase, subunit	Multiple_Complex
TC1600007315.hg.1	3.69	4.51	-1.76	5.58E-05	0.0003	IL21R	interleukin 21 receptor	Multiple_Complex
TC0800010427.hg.1	10.06	9.03	2.05	5.61E-05	0.0003	RB1CC1	RB1-inducible coiled-coil 1	Multiple_Complex
TC1600011478.hg.1	3.74	4.38	-1.56	5.62E-05	0.0003	TMEM186	transmembrane protein 186	Multiple_Complex
TC0700010692.hg.1	11.95	10.13	3.52	5.64E-05	0.0003	NT5C3A	5-nucleotidase, cytosolic IIIA	Multiple_Complex
TC0X00008776.hg.1	4.79	5.43	-1.56	5.65E-05	0.0003	ZNF275	zinc finger protein 275	Multiple_Complex
TC1200012597.hg.1	10.08	8.8	2.42	5.65E-05	0.0003	MGST1	microsomal glutathione S-transferase 1	Multiple_Complex
TC1700006654.hg.1	7.42	8.72	-2.48	5.65E-05	0.0003	RNF167	ring finger protein 167	Multiple_Complex
TC0900010404.hg.1	12.98	12.3	1.6	5.67E-05	0.0003	ZFAND5	zinc finger, AN1-type domain 5	Multiple_Complex
TC1000008236.hg.1	10.02	11.32	-2.47	5.68E-05	0.0003	TSPAN14	tetraspanin 14	Multiple_Complex
TC1500010740.hg.1	6.6	7.52	-1.9	5.68E-05	0.0003	DTWD1	DTW domain containing 1	Multiple_Complex
TC1200010901.hg.1	7.14	7.98	-1.79	5.68E-05	0.0003	RNF41	ring finger protein 41, E3 ubiquitin protein liga	Multiple_Complex
TC0Y00007319.hg.1	7.86	6.85	2.02	5.70E-05	0.0003	AKAP17A; S	Homo sapiens A kinase (PRKA) anchor protein	Multiple_Complex
TC1100013202.hg.1	6.09	6.96	-1.83	5.71E-05	0.0003	ORAOV1	oral cancer overexpressed 1	Multiple_Complex
TC1700012249.hg.1	6.54	7.46	-1.9	5.72E-05	0.0003	MIR4728; E	microRNA 4728; erb-b2 receptor tyrosine kina	Multiple_Complex
TC1900009564.hg.1	4.22	3.56	1.58	5.72E-05	0.0003	OR7G3	olfactory receptor, family 7, subfamily G, mem	Coding
TC2000010018.hg.1	4.95	4.24	1.63	5.72E-05	0.0003	EPPIN-WFD	EPPIN-WFDC6 readthrough	Coding
TC1900010521.hg.1	5.26	6.14	-1.84	5.73E-05	0.0003	ZFP14	ZFP14 zinc finger protein	Multiple_Complex
TC1900008172.hg.1	4.93	5.78	-1.8	5.76E-05	0.0003	ZNF574	zinc finger protein 574	Coding
TC0100008938.hg.1	7.59	8.58	-1.98	5.78E-05	0.0003	LMO4	LIM domain only 4	Multiple_Complex
TC0100013303.hg.1	5.19	6.16	-1.96	5.80E-05	0.0003	HMGCL	3-hydroxymethyl-3-methylglutaryl-CoA lyase	Multiple_Complex
TC1000007471.hg.1	11.78	10.18	3.03	5.88E-05	0.0003	ALOX5	arachidonate 5-lipoxygenase	Multiple_Complex
TC1600011367.hg.1	7.52	8.33	-1.75	5.88E-05	0.0003	SPNS1	spinster homolog 1 (Drosophila)	Multiple_Complex
TC1900011820.hg.1	5.63	6.44	-1.76	5.91E-05	0.0003	ZNF551	zinc finger protein 551	Multiple_Complex
TC0100007398.hg.1	14.45	13.41	2.06	5.93E-05	0.0004	TMEM50A	transmembrane protein 50A	Multiple_Complex
TC0M00006435.hg.1	19.26	19.65	-1.31	5.99E-05	0.0004	COX1	cytochrome c oxidase subunit I	Coding
TC0300013639.hg.1	10.46	9.72	1.67	6.04E-05	0.0004	PPP1R2	protein phosphatase 1, regulatory (inhibitor) s	Multiple_Complex
TC0100009204.hg.1	6.57	7.56	-1.99	6.06E-05	0.0004	TRMT13	tRNA methyltransferase 13 homolog (S. cerevi	Multiple_Complex
TC0100009907.hg.1	5.07	5.68	-1.53	6.08E-05	0.0004	TARS2; MIF	threonyl-tRNA synthetase 2, mitochondrial (pu	Multiple_Complex
TC1900007975.hg.1	5.09	6.09	-2	6.08E-05	0.0004	ZNF570	zinc finger protein 570	Multiple_Complex
TC0300007278.hg.1	11.75	10.57	2.28	6.08E-05	0.0004	NBEAL2	neurobeachin like 2	Multiple_Complex
TC1500006946.hg.1	7.5	6.53	1.96	6.10E-05	0.0004	INAFM2	InaF-motif containing 2	Multiple_Complex
TC1100011539.hg.1	8.25	9.16	-1.88	6.16E-05	0.0004	NUMA1	nuclear mitotic apparatus protein 1	Multiple_Complex

TC1800007431.hg.1	8.86	10.19	-2.51	6.20E-05	0.0004	MALT1	MALT1 paracaspase	Multiple_Complex
TSUnmapped00000432.h	6.92	6.22	1.62	6.21E-05	0.0004	NDUFA6	NADH dehydrogenase (ubiquinone) 1 alpha su	Coding
TC1700008104.hg.1	7.49	8.97	-2.79	6.21E-05	0.0004	NSF	N-ethylmaleimide-sensitive factor	Multiple_Complex
TC0600006836.hg.1	9.69	10.82	-2.19	6.22E-05	0.0004	LY86	lymphocyte antigen 86	Multiple_Complex
TC0X00011211.hg.1	12.18	11.01	2.24	6.23E-05	0.0004	G6PD	glucose-6-phosphate dehydrogenase	Multiple_Complex
TC0500006442.hg.1	8.38	9.03	-1.57	6.24E-05	0.0004	SDHA	succinate dehydrogenase complex subunit A,	Multiple_Complex
TC0200008259.hg.1	9.02	9.91	-1.85	6.29E-05	0.0004	MAT2A	methionine adenosyltransferase II, alpha	Multiple_Complex
TC0700013509.hg.1	7.53	8.33	-1.73	6.29E-05	0.0004	DAGLB	diacylglycerol lipase, beta	Multiple_Complex
TC1300009637.hg.1	5.25	6.1	-1.81	6.29E-05	0.0004	GGACT	gamma-glutamylamine cyclotransferase	Multiple_Complex
TC1000006754.hg.1	14.51	15.49	-1.97	6.34E-05	0.0004	CELF2	CUGBP, Elav-like family member 2	Multiple_Complex
TC0300014082.hg.1	4.47	3.65	1.76	6.34E-05	0.0004	ETV5	ets variant 5	Multiple_Complex
TC1000008269.hg.1	12.31	11.55	1.69	6.36E-05	0.0004	GHITM	growth hormone inducible transmembrane pr	Multiple_Complex
TC0700011499.hg.1	4.09	4.71	-1.55	6.37E-05	0.0004	DNAJC30	DnaJ (Hsp40) homolog, subfamily C, member	Coding
TC1600011325.hg.1	6.04	6.85	-1.74	6.38E-05	0.0004	CRAMP1	cramped chromatin regulator homolog 1	Multiple_Complex
TC0100015023.hg.1	13.31	14.43	-2.18	6.39E-05	0.0004	DPYD	dihydropyrimidine dehydrogenase	Multiple_Complex
TC0100013087.hg.1	8.36	9.39	-2.04	6.40E-05	0.0004	RCC2	regulator of chromosome condensation 2	Multiple_Complex
TC0800009674.hg.1	6.23	5.34	1.86	6.43E-05	0.0004	LONRF1	Transcript Identified by AceView, Entrez Gene	Unassigned
TC1600011234.hg.1	5.35	5.91	-1.48	6.45E-05	0.0004	APRT	adenine phosphoribosyltransferase	Multiple_Complex
TC0700010637.hg.1	4.79	5.33	-1.45	6.46E-05	0.0004	NOD1	nucleotide-binding oligomerization domain co	Multiple_Complex
TC1900009872.hg.1	9.23	10.02	-1.72	6.46E-05	0.0004	RASAL3	RAS protein activator like 3	Multiple_Complex
TC0500008008.hg.1	10.44	8.52	3.79	6.55E-05	0.0004	COX7C; MII	cytochrome c oxidase subunit VIIc; microRNA	Multiple_Complex
TSUnmapped00000195.h	8.53	9.2	-1.6	6.56E-05	0.0004	NDUFA10	NADH dehydrogenase (ubiquinone) 1 alpha su	Coding
TC0100018198.hg.1	4.22	3.43	1.73	6.58E-05	0.0004	SRRM1	serine/arginine repetitive matrix 1	NonCoding
TC2100008462.hg.1	7.24	7.98	-1.66	6.58E-05	0.0004	LSS	lanosterol synthase (2,3-oxidosqualene-lanost	Multiple_Complex
TC1900008085.hg.1	7.22	8.58	-2.56	6.59E-05	0.0004	ZNF546	zinc finger protein 546	Multiple_Complex
TC1900012048.hg.1	3.77	3.16	1.53	6.59E-05	0.0004	DNAAF3	dynein, axonemal, assembly factor 3	Multiple_Complex
TC1600008567.hg.1	6.39	5.49	1.86	6.60E-05	0.0004	CENPN	centromere protein N	Multiple_Complex
TC1900011759.hg.1	4.91	4.17	1.67	6.61E-05	0.0004	APOC1	apolipoprotein C-I	Multiple_Complex
TC0200010800.hg.1	4.67	4.02	1.56	6.62E-05	0.0004	VIL1	villin 1	Multiple_Complex
TC0600007257.hg.1	9.38	11.31	-3.81	6.62E-05	0.0004	TRIM38	tripartite motif containing 38	Multiple_Complex
TC0300014053.hg.1	10.68	9.61	2.11	6.65E-05	0.0004	PIK3CB	phosphatidylinositol-4,5-bisphosphate 3-kinas	Multiple_Complex
TC1900011679.hg.1	11.44	9.4	4.1	6.66E-05	0.0004	CYP4F3	cytochrome P450, family 4, subfamily F, polyp	Multiple_Complex
TC1200012334.hg.1	18.14	17.43	1.64	6.68E-05	0.0004	UBC	ubiquitin C	Multiple_Complex
TC0300006985.hg.1	7.37	8.69	-2.5	6.71E-05	0.0004	CCR4	chemokine (C-C motif) receptor 4	Coding
TC0400009746.hg.1	8.44	7.79	1.57	6.76E-05	0.0004	SLBP	stem-loop binding protein	Multiple_Complex
TC0X00009760.hg.1	7.12	8.01	-1.86	6.79E-05	0.0004	SMC1A	structural maintenance of chromosomes 1A	Multiple_Complex
TC0200016603.hg.1	10.78	10.14	1.55	6.80E-05	0.0004	LRRFIP1	leucine rich repeat (in FLII) interacting protein	Multiple_Complex
TC0900011713.hg.1	11.32	12.07	-1.69	6.80E-05	0.0004	FNBP1	formin binding protein 1	Multiple_Complex
TC0200010416.hg.1	4.52	6.38	-3.65	6.81E-05	0.0004	SPATS2L	Transcript Identified by AceView, Entrez Gene	Unassigned
TC1700011083.hg.1	4.49	3.78	1.64	6.85E-05	0.0004	SAMD14	sterile alpha motif domain containing 14	Multiple_Complex
TC0300008381.hg.1	6.5	7.35	-1.8	6.87E-05	0.0004	QTRTD1	queuine tRNA-ribosyltransferase domain cont	Multiple_Complex
TC0900010444.hg.1	8.34	8.96	-1.53	6.88E-05	0.0004	CARNMT1	carnosine N-methyltransferase 1	Multiple_Complex
TC1100012166.hg.1	8.59	6.68	3.74	6.91E-05	0.0004	CASP5	caspase 5	Multiple_Complex
TC1700011448.hg.1	5.8	6.72	-1.88	6.91E-05	0.0004	POLG2	polymerase (DNA directed), gamma 2, access	Multiple_Complex
TC0400011220.hg.1	9.67	8.34	2.51	6.92E-05	0.0004	WDFY3	WD repeat and FYVE domain containing 3	Multiple_Complex
TC0600012671.hg.1	6.14	7.66	-2.87	6.94E-05	0.0004	ASCC3	activating signal cointegrator 1 complex subur	Multiple_Complex
TC0100010360.hg.1	7.48	8.59	-2.15	6.95E-05	0.0004	HSPA6	heat shock 70kDa protein 6 (HSP70B)	Multiple_Complex
TC1200011994.hg.1	4.76	4.09	1.59	6.97E-05	0.0004	SDS	serine dehydratase	Multiple_Complex
TC1400010019.hg.1	6.96	6.4	1.47	6.99E-05	0.0004	MOAP1	modulator of apoptosis 1	Coding
TC1100008069.hg.1	5.44	4.6	1.8	7.02E-05	0.0004	CNIH2	cornichon family AMPA receptor auxiliary prot	Multiple_Complex
TC1400009190.hg.1	6.91	8.03	-2.16	7.04E-05	0.0004	GNPNAT1	glucosamine-phosphate N-acetyltransferase 1	Multiple_Complex
TC0300013763.hg.1	8.35	9.79	-2.71	7.08E-05	0.0004	RUBCN; MI	RUN domain and cysteine-rich domain contair	Multiple_Complex
TC0100014855.hg.1	9.05	10.41	-2.57	7.12E-05	0.0004	GBP4	guanylate binding protein 4	Multiple_Complex
TC0100010672.hg.1	9.33	8.45	1.84	7.12E-05	0.0004	RABGAP1L	RAB GTPase activating protein 1-like	Multiple_Complex
TC0200013516.hg.1	13.23	14.25	-2.03	7.16E-05	0.0004	TMEM127	transmembrane protein 127	Coding
TC1600008228.hg.1	7.19	8.11	-1.89	7.17E-05	0.0004	CYB5B	cytochrome b5 type B (outer mitochondrial m	Multiple_Complex
TC1600007308.hg.1	4.5	5.2	-1.62	7.22E-05	0.0004	KDM8	lysine (K)-specific demethylase 8	Multiple_Complex
TSUnmapped00000299.h	6.87	5.85	2.03	7.26E-05	0.0004	TRAPPC4	trafficking protein particle complex 4	NonCoding
TC0100010334.hg.1	9.14	7.77	2.59	7.27E-05	0.0004	UFC1	ubiquitin-fold modifier conjugating enzyme 1	Multiple_Complex
TC1900010792.hg.1	8.32	7.43	1.85	7.30E-05	0.0004	DEDD2	death effector domain containing 2	Multiple_Complex
TC1200010835.hg.1	8.03	9.87	-3.57	7.33E-05	0.0004	NFE2	nuclear factor, erythroid 2	Multiple_Complex
TC0400006802.hg.1	4.86	4.3	1.47	7.33E-05	0.0004	USP17L19	ubiquitin specific peptidase 17-like family mer	Coding
TC2200007607.hg.1	11.39	12.59	-2.29	7.35E-05	0.0004	NUP50	nucleoporin 50kDa	Multiple_Complex
TC0600014274.hg.1	4.7	5.73	-2.03	7.36E-05	0.0004	HLA-DOB	major histocompatibility complex, class II, DO	Multiple_Complex
TC2000006781.hg.1	10.3	9	2.47	7.37E-05	0.0004	SNRPB2	small nuclear ribonucleoprotein polypeptide E	Multiple_Complex
TC1600008585.hg.1	11.65	10.39	2.4	7.41E-05	0.0004	CMIP	c-Maf inducing protein	Multiple_Complex
TC0300012269.hg.1	6.58	5.54	2.05	7.42E-05	0.0004	ALDH1L1	aldehyde dehydrogenase 1 family, member L1	Multiple_Complex
TC0600007748.hg.1	8.92	9.65	-1.66	7.42E-05	0.0004	HMGA1	high mobility group AT-hook 1	Multiple_Complex
TC0800007697.hg.1	6.75	6.07	1.61	7.43E-05	0.0004	CHCHD7	coiled-coil-helix-coiled-coil-helix domain conta	Multiple_Complex
TC0500006744.hg.1	8.6	7.45	2.21	7.43E-05	0.0004	ROPN1L	rhophilin associated tail protein 1-like	Multiple_Complex

TC1000012595.hg.1	8.25	9.05	-1.74	7.43E-05	0.0004	ZDHHC6	zinc finger, DHHC-type containing 6	Multiple_Complex
TC1100006572.hg.1	6.27	6.83	-1.47	7.45E-05	0.0004	TSPAN32	tetraspanin 32	Multiple_Complex
TC0100016629.hg.1	6.81	7.85	-2.06	7.46E-05	0.0004	RNASEL	ribonuclease L (2,5-oligoisoadenylate syntheta	Coding
TSUnmapped00000236.h	8.61	9.59	-1.97	7.46E-05	0.0004	TCF20	transcription factor 20 (AR1)	Coding
TSUnmapped00000511.h	8.61	9.59	-1.97	7.46E-05	0.0004	TCF20	transcription factor 20 (AR1)	Coding
TC0100017446.hg.1	4.71	5.66	-1.93	7.49E-05	0.0004	SUSD4	sushi domain containing 4	Multiple_Complex
TC2000008007.hg.1	12.78	9.11	12.67	7.51E-05	0.0004	RPS21	ribosomal protein S21	Multiple_Complex
TC2000009918.hg.1	8.76	6.6	4.47	7.52E-05	0.0004	BPI	bactericidal/permeability-increasing protein	Multiple_Complex
TC0900009308.hg.1	7.52	8.38	-1.81	7.53E-05	0.0004	EHMT1	euchromatic histone-lysine N-methyltransfera	Multiple_Complex
TC1900009316.hg.1	14.13	15.38	-2.38	7.54E-05	0.0004	EEF2; SNOF	eukaryotic translation elongation factor 2; sm	Multiple_Complex
TC1100009419.hg.1	7.26	7.9	-1.56	7.56E-05	0.0004	EI24	etoposide induced 2.4	Multiple_Complex
TC0200013912.hg.1	5.39	4.73	1.58	7.56E-05	0.0004	CKAP2L	cytoskeleton associated protein 2-like	Multiple_Complex
TC1900008259.hg.1	3.78	4.55	-1.71	7.62E-05	0.0004	ZNF233	zinc finger protein 233	Multiple_Complex
TC0400006513.hg.1	5.16	5.88	-1.65	7.66E-05	0.0004	CTBP1-AS2	CTBP1 antisense RNA 2 (head to head)	Multiple_Complex
TC0200013779.hg.1	5.55	4.33	2.32	7.68E-05	0.0004	ST6GAL2	ST6 beta-galactosamide alpha-2,6-sialyltranfe	Multiple_Complex
TC1800007215.hg.1	6.27	5.2	2.1	7.72E-05	0.0004	HAUS1	HAUS augmin like complex subunit 1	Multiple_Complex
TC1400010709.hg.1	4.21	4.83	-1.54	7.75E-05	0.0004	TTC5	tetratricopeptide repeat domain 5	Multiple_Complex
TC1900007025.hg.1	10.48	8.88	3.03	7.75E-05	0.0004	PLPPR2	phospholipid phosphatase related 2	Multiple_Complex
TC1100011980.hg.1	7.83	8.95	-2.18	7.76E-05	0.0004	TAF1D; SNC	TATA box binding protein associated factor 1D	Multiple_Complex
TC0800007328.hg.1	7.39	8.09	-1.62	7.81E-05	0.0004	BAG4	BCL2-associated athanogene 4	Multiple_Complex
TC0500012100.hg.1	12.75	11.92	1.77	7.81E-05	0.0004	H2AFY	H2A histone family, member Y	Multiple_Complex
TSUnmapped00000531.h	8.06	9.12	-2.1	7.82E-05	0.0004	RASA3	RAS p21 protein activator 3	Coding
TC0100017664.hg.1	5.8	6.69	-1.85	7.82E-05	0.0004	NUP133	nucleoporin 133kDa	Multiple_Complex
TC1200010412.hg.1	4.73	5.97	-2.37	7.82E-05	0.0004	ABCD2	Transcript Identified by AceView, Entrez Gene	Unassigned
TC1300006923.hg.1	6.61	7.63	-2.03	7.84E-05	0.0005	NHLRC3	NHL repeat containing 3	Multiple_Complex
TC1900011935.hg.1	5.02	5.92	-1.87	7.86E-05	0.0005	ZFP30	ZFP30 zinc finger protein	Multiple_Complex
TC0100014788.hg.1	5.78	6.92	-2.2	7.87E-05	0.0005	ZNHIT6	zinc finger, HIT-type containing 6	Multiple_Complex
TC0200008458.hg.1	3.5	4.38	-1.84	7.89E-05	0.0005	ZNF2	zinc finger protein 2	Coding
TC1600010752.hg.1	6.42	7.18	-1.69	7.90E-05	0.0005	AARS	alanyl-tRNA synthetase	Multiple_Complex
TC0700012255.hg.1	5.31	6.06	-1.68	7.91E-05	0.0005	IMMP2L	inner mitochondrial membrane peptidase sub	Multiple_Complex
TC1400008919.hg.1	7.6	6.28	2.5	7.92E-05	0.0005	CFL2	cofilin 2 (muscle)	Multiple_Complex
TC1100012121.hg.1	15.09	17.6	-5.71	7.94E-05	0.0005	TMEM123	transmembrane protein 123	Multiple_Complex
TC0200016746.hg.1	4.62	5.36	-1.66	7.94E-05	0.0005	TTC21B	tetratricopeptide repeat domain 21B	NonCoding
TC1700009538.hg.1	8.43	9.44	-2.01	7.95E-05	0.0005	SLC25A11	solute carrier family 25 (mitochondrial carrier	Multiple_Complex
TC1300006767.hg.1	9.43	8.32	2.16	7.99E-05	0.0005	USPL1	ubiquitin specific peptidase like 1	Multiple_Complex
TC2100006852.hg.1	9.7	8.02	3.2	8.00E-05	0.0005	USP16	ubiquitin specific peptidase 16	Multiple_Complex
TC1700011744.hg.1	4.9	5.64	-1.66	8.02E-05	0.0005	MIF4GD	MIF4G domain containing	Multiple_Complex
TC0X00009123.hg.1	5.12	4.64	1.39	8.03E-05	0.0005	ASB11	ankyrin repeat and SOCS box containing 11, E3	Coding
TC0400012312.hg.1	6.6	5.88	1.65	8.04E-05	0.0005	ANP32C	acidic nuclear phosphoprotein 32 family mem	Multiple_Complex
TC0100015353.hg.1	12.9	12.08	1.77	8.04E-05	0.0005	CSDE1	cold shock domain containing E1, RNA binding	Multiple_Complex
TC1600009412.hg.1	10.81	9.87	1.91	8.05E-05	0.0005	TXNDC11	thioredoxin domain containing 11	Multiple_Complex
TC0600013177.hg.1	8.15	8.99	-1.78	8.07E-05	0.0005	MED23	mediator complex subunit 23	Multiple_Complex
TC0300006462.hg.1	6.11	7.04	-1.92	8.08E-05	0.0005	TRNT1	tRNA nucleotidyl transferase, CCA-adding, 1	Multiple_Complex
TC1900011813.hg.1	5.92	6.66	-1.67	8.08E-05	0.0005	ZNF548	zinc finger protein 548	Multiple_Complex
TC0X00010465.hg.1	5.71	6.45	-1.67	8.09E-05	0.0005	NUP62CL	nucleoporin 62kDa C-terminal like	Multiple_Complex
TC0600014233.hg.1	12.28	13.16	-1.84	8.13E-05	0.0005	SSR1	signal sequence receptor, alpha	Multiple_Complex
TC0900011473.hg.1	8.96	8.18	1.73	8.14E-05	0.0005	DENND1A	DENN/MADD domain containing 1A	Multiple_Complex
TC0700013055.hg.1	6.52	7.45	-1.9	8.17E-05	0.0005	CDK5	cyclin-dependent kinase 5	Multiple_Complex
TC1700011366.hg.1	5.23	6.44	-2.31	8.21E-05	0.0005	INTS2	integrator complex subunit 2	Multiple_Complex
TC0X00007574.hg.1	4.45	5.48	-2.05	8.23E-05	0.0005	ACRC	acidic repeat containing	Multiple_Complex
TC1000007700.hg.1	14.71	13.74	1.95	8.26E-05	0.0005	UBE2D1	ubiquitin conjugating enzyme E2D 1	Multiple_Complex
TC1500007659.hg.1	13.91	14.76	-1.81	8.28E-05	0.0005	PIAS1	protein inhibitor of activated STAT 1	Multiple_Complex
TC2100006442.hg.1	5.58	5.1	1.39	8.30E-05	0.0005	SIK1	salt-inducible kinase 1	Multiple_Complex
TC1700008178.hg.1	9.82	10.52	-1.62	8.31E-05	0.0005	ATP5G1	ATP synthase, H+ transporting, mitochondrial	Multiple_Complex
TC0300007445.hg.1	4.41	3.92	1.4	8.39E-05	0.0005	GRM2	glutamate receptor, metabotropic 2	Multiple_Complex
TC1400010575.hg.1	7.79	7.22	1.48	8.40E-05	0.0005	BCL2L2	BCL2-like 2	Multiple_Complex
TC1800007868.hg.1	7.71	6.34	2.59	8.42E-05	0.0005	YES1	YES proto-oncogene 1, Src family tyrosine kin	Multiple_Complex
TC0700009061.hg.1	9.3	8.32	1.98	8.44E-05	0.0005	CALU	calumenin	Multiple_Complex
TC0500012390.hg.1	4.96	6.2	-2.36	8.45E-05	0.0005	JAKMIP2	janus kinase and microtubule interacting prot	Multiple_Complex
TC1100006494.hg.1	8.02	7.13	1.85	8.51E-05	0.0005	CD151	CD151 molecule (Raph blood group)	Multiple_Complex
TC1200009416.hg.1	8.24	7.16	2.12	8.54E-05	0.0005	RAN	RAN, member RAS oncogene family	Multiple_Complex
TC0900007775.hg.1	8.13	8.78	-1.58	8.55E-05	0.0005	NAA35	N(alpha)-acetyltransferase 35, NatC auxiliary s	Multiple_Complex
TC2000009357.hg.1	15.42	13.93	2.81	8.60E-05	0.0005	PREX1	phosphatidylinositol-3,4,5-trisphosphate-dep	Multiple_Complex
TC0100009365.hg.1	9.85	10.72	-1.83	8.61E-05	0.0005	AHCYL1	adenosylhomocysteinase like 1	Multiple_Complex
TC0X00010926.hg.1	6.64	7.54	-1.87	8.61E-05	0.0005	MAP7D3	MAP7 domain containing 3	Multiple_Complex
TC0100006550.hg.1	5.76	6.65	-1.84	8.64E-05	0.0005	PRKCZ	protein kinase C, zeta	Multiple_Complex
TC0300007474.hg.1	8.64	9.29	-1.57	8.65E-05	0.0005	DNAH1	dynein, axonemal, heavy chain 1	Multiple_Complex
TC1900008879.hg.1	4.53	5.99	-2.74	8.66E-05	0.0005	KIR2DS5; K	killer cell immunoglobulin-like receptor, two d	Multiple_Complex
TC0300007607.hg.1	5.44	6.52	-2.12	8.67E-05	0.0005	RPP14	ribonuclease P/MRP 14kDa subunit	Multiple_Complex
TC1200009744.hg.1	6.83	7.72	-1.86	8.68E-05	0.0005	LPAR5	lysophosphatidic acid receptor 5	Multiple_Complex

TC0400006437.hg.1	6.25	7.04	-1.72	8.70E-05	0.0005	ZNF718	zinc finger protein 718	Multiple_Complex
TC1000009844.hg.1	6.42	7.37	-1.94	8.74E-05	0.0005	SEPHS1	selenophosphate synthetase 1	Multiple_Complex
TC0500009095.hg.1	5.63	4.94	1.62	8.76E-05	0.0005	NDST1	N-deacetylase/N-sulfotransferase (heparan gl	Multiple_Complex
TC0800006692.hg.1	8.04	7	2.06	8.77E-05	0.0005	MSRA	methionine sulfoxide reductase A	Multiple_Complex
TC0400007592.hg.1	6.79	8.11	-2.5	8.82E-05	0.0005	PAICS	phosphoribosylaminoimidazole carboxylase, p	Multiple_Complex
TC0600009112.hg.1	11.08	9.82	2.38	8.84E-05	0.0005	CDC40	cell division cycle 40	Multiple_Complex
TC0500013401.hg.1	14.32	15.63	-2.49	8.85E-05	0.0005	LCP2	lymphocyte cytosolic protein 2	Multiple_Complex
TC1200012083.hg.1	6.8	7.59	-1.72	8.86E-05	0.0005	FBXO21	F-box protein 21	Multiple_Complex
TC1700012218.hg.1	4.78	5.47	-1.62	8.88E-05	0.0005	SARM1	sterile alpha and TIR motif containing 1	Multiple_Complex
TC1100013057.hg.1	12.79	11.35	2.7	8.91E-05	0.0005	TCIRG1	T-cell, immune regulator 1, ATPase, H+ transp	Multiple_Complex
TC0400012018.hg.1	4.63	6.54	-3.76	8.93E-05	0.0005	GYPB	glycophorin B (MNS blood group)	Multiple_Complex
TC1900006526.hg.1	4.23	5.52	-2.44	8.94E-05	0.0005	MUM1	melanoma associated antigen (mutated) 1	Multiple_Complex
TC1400010784.hg.1	5.36	6	-1.56	8.94E-05	0.0005	SYNE3	spectrin repeat containing, nuclear envelope f	Multiple_Complex
TSUnmapped00000610.h	5.48	4.81	1.59	8.95E-05	0.0005	HNRNPCL2	heterogeneous nuclear ribonucleoprotein C-lil	Coding
TC1600011499.hg.1	5.77	5.05	1.65	8.99E-05	0.0005	ERI2	ERI1 exoribonuclease family member 2	Multiple_Complex
TC1100011829.hg.1	6.26	7.28	-2.02	9.01E-05	0.0005	CREBZF	CREB/ATF bZIP transcription factor	Multiple_Complex
TC1700009378.hg.1	10.54	9.84	1.62	9.08E-05	0.0005	PITPNA	phosphatidylinositol transfer protein, alpha	Multiple_Complex
TC0700009485.hg.1	9.2	10.45	-2.38	9.08E-05	0.0005	GSTK1	glutathione S-transferase kappa 1	Multiple_Complex
TC0200008996.hg.1	15.65	14.55	2.14	9.09E-05	0.0005	ACTR3	ARP3 actin-related protein 3 homolog (yeast)	Multiple_Complex
TC0100017666.hg.1	6.63	7.54	-1.88	9.10E-05	0.0005	ABCB10	ATP binding cassette subfamily B member 10	Multiple_Complex
TC0300012790.hg.1	7.6	7.09	1.42	9.10E-05	0.0005	TMEM183E	transmembrane protein 183B	Multiple_Complex
TC0700011550.hg.1	11.41	12.73	-2.5	9.11E-05	0.0005	POM121C	POM121 transmembrane nucleoporin C	Multiple_Complex
TC0800007077.hg.1	10.56	11.12	-1.47	9.12E-05	0.0005	PPP2R2A	protein phosphatase 2, regulatory subunit B, a	Multiple_Complex
TC1900009352.hg.1	5.45	4.85	1.52	9.15E-05	0.0005	SEMA6B	sema domain, transmembrane domain (TM), a	Multiple_Complex
TC0100011242.hg.1	8.8	8	1.74	9.16E-05	0.0005	TMEM183A	transmembrane protein 183A	Multiple_Complex
TC0100016678.hg.1	15.17	13.26	3.77	9.17E-05	0.0005	FAM129A	family with sequence similarity 129, member .	Multiple_Complex
TC0600011136.hg.1	7.82	6.39	2.69	9.24E-05	0.0005	HIST1H2BG	histone cluster 1, H2bg	Multiple_Complex
TC1100009091.hg.1	5.18	4.42	1.69	9.26E-05	0.0005	CLDN25	claudin 25	Coding
TC1200009649.hg.1	7.48	8.03	-1.47	9.26E-05	0.0005	CRACR2A	calcium release activated channel regulator 2A	Multiple_Complex
TC0100013287.hg.1	5.73	6.7	-1.95	9.29E-05	0.0005	E2F2	E2F transcription factor 2	Multiple_Complex
TC1600007814.hg.1	7.19	8.04	-1.81	9.30E-05	0.0005	ADCY7	adenylate cyclase 7	Multiple_Complex
TC2100008165.hg.1	8.49	9.32	-1.77	9.32E-05	0.0005	BRWD1	bromodomain and WD repeat domain contair	Multiple_Complex
TC0100008536.hg.1	5.85	6.71	-1.82	9.32E-05	0.0005	INADL	InaD-like (Drosophila)	Multiple_Complex
TC0500007337.hg.1	14.1	15.25	-2.21	9.33E-05	0.0005	PARP8	poly(ADP-ribose) polymerase family member .	Multiple_Complex
TC0300008088.hg.1	4.35	5.1	-1.68	9.33E-05	0.0005	CRYBG3	crystallin beta-gamma domain containing 3	Multiple_Complex
TC1700010618.hg.1	6.29	5.22	2.11	9.35E-05	0.0005	TOP2A	topoisomerase (DNA) II alpha	Multiple_Complex
TC0300010076.hg.1	11.53	8.72	7.02	9.39E-05	0.0005	RPL35A	ribosomal protein L35a	Multiple_Complex
TC0200010164.hg.1	6.71	5.6	2.16	9.42E-05	0.0005	SSFA2	sperm specific antigen 2	Multiple_Complex
TC0900008847.hg.1	9.98	7.41	5.92	9.43E-05	0.0005	LCN2	lipocalin 2	Multiple_Complex
TC1500008983.hg.1	13.34	12.53	1.75	9.44E-05	0.0005	SLC12A6	solute carrier family 12 (potassium/chloride tr	Multiple_Complex
TC0100018553.hg.1	6.89	6.33	1.48	9.50E-05	0.0005	PLEKHA6	pleckstrin homology domain containing, famil	Multiple_Complex
TSUnmapped00000480.h	6.98	7.69	-1.64	9.55E-05	0.0005	ZNF780B	zinc finger protein 780B	Coding
TC1200011301.hg.1	8.49	7.71	1.71	9.56E-05	0.0005	KRR1	KRR1, small subunit (SSU) processome compo	Multiple_Complex
TC0700008560.hg.1	11.97	10.9	2.1	9.56E-05	0.0005	GNB2	guanine nucleotide binding protein (G protein	Multiple_Complex
TC0600007384.hg.1	5.29	4.33	1.95	9.57E-05	0.0005	HIST1H2BC	histone cluster 1, H2bo	Coding
TC1700012092.hg.1	5.67	6.28	-1.53	9.61E-05	0.0005	DUS1L	dihydrouridine synthase 1-like	Multiple_Complex
TC1400008036.hg.1	5.65	5.04	1.53	9.63E-05	0.0005	COX8C	cytochrome c oxidase subunit VIIIC	Coding
TC1100009827.hg.1	5.14	5.88	-1.66	9.69E-05	0.0005	OSBPL5	oxysterol binding protein-like 5	Multiple_Complex
TC0100016656.hg.1	13.72	12.61	2.16	9.72E-05	0.0005	NCF2	neutrophil cytosolic factor 2	Multiple_Complex
TC2000008268.hg.1	6.35	7.39	-2.05	9.73E-05	0.0005	SLC23A2	solute carrier family 23 (ascorbic acid transpo	Multiple_Complex
TC0400006462.hg.1	4.91	5.85	-1.91	9.77E-05	0.0005	PIGG	phosphatidylinositol glycan anchor biosynthes	Multiple_Complex
TC0100013384.hg.1	4.59	6.68	-4.25	9.77E-05	0.0005	ZNF683	zinc finger protein 683	Coding
TSUnmapped00000461.h	5.2	5.94	-1.67	9.81E-05	0.0005	ZNF35	zinc finger protein 35	Coding
TC0X00007353.hg.1	6.54	7.32	-1.71	9.83E-05	0.0006	TSR2	TSR2, 20S rRNA accumulation, homolog (S. cer	Coding
TC0700007949.hg.1	8.31	9.79	-2.81	9.83E-05	0.0006	POM121	POM121 transmembrane nucleoporin	Multiple_Complex
TC1300007104.hg.1	6.6	7.68	-2.11	9.83E-05	0.0006	LRCH1	leucine-rich repeats and calponin homology ((	Multiple_Complex
TC0300012166.hg.1	14.01	12.87	2.22	9.84E-05	0.0006	HCLS1	hematopoietic cell-specific Lyn substrate 1	Multiple_Complex
TC0200006647.hg.1	6.12	7.01	-1.86	9.90E-05	0.0006	CPSF3	cleavage and polyadenylation specific factor 3	Multiple_Complex
TC1100012353.hg.1	4.87	5.65	-1.72	9.91E-05	0.0006	ZW10	zw10 kinetochore protein	Multiple_Complex
TC0400006578.hg.1	11.08	11.86	-1.72	9.92E-05	0.0006	SH3BP2	SH3-domain binding protein 2	Multiple_Complex
TC0600014333.hg.1	8.63	7.71	1.88	9.92E-05	0.0006	MICAL1	microtubule associated monooxygenase, calp	Multiple_Complex
TC1000012429.hg.1	7.61	6.63	1.97	9.98E-05	0.0006	PRPF18	pre-mRNA processing factor 18	Multiple_Complex
TC1900011196.hg.1	5.11	5.94	-1.77	0.0001	0.0006	NAPSA	napsin A aspartic peptidase	Multiple_Complex
TC0600011546.hg.1	8.37	9.29	-1.9	0.0001	0.0006	CUTA	cutA divalent cation tolerance homolog (E. col	Multiple_Complex
TC1900012026.hg.1	6.41	7.09	-1.6	0.0001	0.0006	ZNF468	zinc finger protein 468	Multiple_Complex
TC0100009414.hg.1	10.87	9.48	2.62	0.0001	0.0006	ATP5F1	ATP synthase, H+ transporting, mitochondrial	Multiple_Complex
TC0100014245.hg.1	8.83	8.15	1.6	0.0001	0.0006	YIPF1	Yip1 domain family member 1	Multiple_Complex
TC0100013323.hg.1	4.8	5.72	-1.89	0.0001	0.0006	STPG1	sperm-tail PG-rich repeat containing 1	Multiple_Complex
TC1100008370.hg.1	7.66	6.54	2.17	0.0001	0.0006	ATG16L2	autophagy related 16-like 2	Multiple_Complex
TC0100009116.hg.1	5.5	6.33	-1.77	0.0001	0.0006	RWDD3; TN	RWD domain containing 3; transmembrane pr	Multiple_Complex

TC0300010943.hg.1	10.2	12.21	-4.03	0.0001	0.0006	CCR1	chemokine (C-C motif) receptor 1	Coding
TC0200016584.hg.1	6.95	5.71	2.37	0.0001	0.0006	HSPE1	heat shock 10kDa protein 1	Multiple_Complex
TC1100009122.hg.1	5.1	3.95	2.22	0.0001	0.0006	NXPE2	neurexophilin and PC-esterase domain family,	Multiple_Complex
TC1400010171.hg.1	7.9	7.11	1.73	0.0001	0.0006	CCDC85C	coiled-coil domain containing 85C	Multiple_Complex
TC0600009800.hg.1	10.26	9.3	1.94	0.0001	0.0006	GINM1	glycoprotein integral membrane 1	Multiple_Complex
TC1300008633.hg.1	10.44	11.45	-2.02	0.0001	0.0006	SUPT20H	SPT20 homolog, SAGA complex component	Multiple_Complex
TC0800012392.hg.1	9.73	10.43	-1.62	0.0001	0.0006	CNOT7	CCR4-NOT transcription complex subunit 7	Multiple_Complex
TC0X00009795.hg.1	13.29	16.78	-11.19	0.0001	0.0006	ALAS2	5-aminolevulinate synthase 2	Multiple_Complex
TC2200007468.hg.1	4.22	5.01	-1.73	0.0001	0.0006	TEF	thyrotrophic embryonic factor	Multiple_Complex
TC0500012977.hg.1	9.34	8.42	1.89	0.0001	0.0006	PDLIM7	PDZ and LIM domain 7 (enigma)	Multiple_Complex
TSUnmapped00000123.h	5.05	5.75	-1.63	0.0001	0.0006	SLC2A6	solute carrier family 2 (facilitated glucose tran	Coding
TC1900007820.hg.1	4.76	5.75	-1.99	0.0001	0.0006	ZNF181	zinc finger protein 181	Multiple_Complex
TSUnmapped00000113.h	8.3	6.87	2.7	0.0001	0.0006	PADI4	peptidyl arginine deiminase, type IV	Coding
TC0300007255.hg.1	4.42	6.51	-4.27	0.0001	0.0006	CCR3	chemokine (C-C motif) receptor 3	Multiple_Complex
TC0700007870.hg.1	6.6	7.34	-1.67	0.0001	0.0006	STAG3L4	stromal antigen 3-like 4 (pseudogene)	Multiple_Complex
TC1400008591.hg.1	7.88	7.1	1.73	0.0001	0.0006	TMEM55B	transmembrane protein 55B	Multiple_Complex
TC0900011829.hg.1	6.52	7.67	-2.22	0.0001	0.0006	RALGDS	ral guanine nucleotide dissociation stimulator	Multiple_Complex
TC0300006575.hg.1	5.63	6.43	-1.74	0.0001	0.0006	FANCD2	Fanconi anemia complementation group D2	Multiple_Complex
TC0100008803.hg.1	6.73	7.53	-1.74	0.0001	0.0006	FAM73A	family with sequence similarity 73, member A	Multiple_Complex
TSUnmapped00000369.h	6.71	7.43	-1.64	0.0001	0.0006	ZNF780B	zinc finger protein 780B	Coding
TC1700007620.hg.1	6.02	7.36	-2.54	0.0001	0.0006	CCL4L2; CC	chemokine (C-C motif) ligand 4-like 2; chemok	Multiple_Complex
TC1500007077.hg.1	5.34	6.28	-1.92	0.0001	0.0006	WDR76	WD repeat domain 76	Multiple_Complex
TC1100011966.hg.1	7.57	6.88	1.6	0.0001	0.0006	SLC36A4	solute carrier family 36 (proton/amino acid sy	Multiple_Complex
TC1600011387.hg.1	9.2	8.61	1.51	0.0001	0.0006	STX4	syntaxin 4	Multiple_Complex
TC1000009327.hg.1	4.29	4.73	-1.36	0.0001	0.0006	MGMT	O-6-methylguanine-DNA methyltransferase	Multiple_Complex
TC1300006683.hg.1	12.38	13.25	-1.83	0.0001	0.0006	GTF3A	general transcription factor IIIA	Multiple_Complex
TC1400007259.hg.1	9.99	8.78	2.31	0.0001	0.0006	PELI2	pellino E3 ubiquitin protein ligase family mem	Multiple_Complex
TC0300012393.hg.1	9.97	9.14	1.78	0.0001	0.0006	MBD4	methyl-CpG binding domain 4 DNA glycosylas	Multiple_Complex
TC0800007414.hg.1	12.22	11.48	1.67	0.0001	0.0006	GOLGA7	golgin A7	Multiple_Complex
TC0700009317.hg.1	7.56	8.53	-1.97	0.0001	0.0006	TRIM24	tripartite motif containing 24	Multiple_Complex
TC1200007809.hg.1	3.95	4.69	-1.67	0.0001	0.0006	GDF11	growth differentiation factor 11	Coding
TC1200011699.hg.1	6.57	7.31	-1.67	0.0001	0.0006	ARL1	ADP-ribosylation factor like GTPase 1	Multiple_Complex
TC0300010999.hg.1	8.79	10.22	-2.68	0.0001	0.0006	MAP4	microtubule associated protein 4	Multiple_Complex
TC0100007038.hg.1	7.81	8.74	-1.9	0.0001	0.0006	NECAP2	NECAP endocytosis associated 2	Multiple_Complex
TC1700008133.hg.1	10.91	11.92	-2.01	0.0001	0.0006	KPNB1	karyopherin (importin) beta 1	Multiple_Complex
TC1000009685.hg.1	4.51	5.17	-1.58	0.0001	0.0006	ANKRD16	ankyrin repeat domain 16	Multiple_Complex
TC0200013535.hg.1	5.7	6.39	-1.61	0.0001	0.0006	LMAN2L	lectin, mannose-binding 2-like	Multiple_Complex
TC0600008156.hg.1	7.21	8.78	-2.99	0.0001	0.0006	ENPP4	ectonucleotide pyrophosphatase/phosphodie	Coding
TC1700007907.hg.1	5.38	6.02	-1.56	0.0001	0.0006	TUBG2	tubulin, gamma 2	Multiple_Complex
TC1400008635.hg.1	7.37	8.26	-1.86	0.0001	0.0006	RAB2B	RAB2B, member RAS oncogene family	Multiple_Complex
TC1400008625.hg.1	15.71	14.81	1.88	0.0001	0.0006	HNRNPC	heterogeneous nuclear ribonucleoprotein C (C	Multiple_Complex
TC1900011582.hg.1	4.85	5.54	-1.61	0.0001	0.0006	ZNF671	zinc finger protein 671	Multiple_Complex
TC1000010065.hg.1	7.36	6.03	2.52	0.0001	0.0006	ARHGAP21	Rho GTPase activating protein 21	Multiple_Complex
TC0600014328.hg.1	10.31	11.49	-2.27	0.0001	0.0006	OSTM1	osteopetrosis associated transmembrane prot	Multiple_Complex
TC0100014203.hg.1	5.86	6.94	-2.13	0.0001	0.0006	ZCCHC11	zinc finger, CCHC domain containing 11	Multiple_Complex
TC0800011683.hg.1	5.86	6.75	-1.86	0.0001	0.0006	FBXO32	F-box protein 32	Multiple_Complex
TC0900008804.hg.1	5.01	5.88	-1.82	0.0001	0.0006	ZNF79	zinc finger protein 79	Multiple_Complex
TC0600007375.hg.1	8.93	7.47	2.76	0.0001	0.0006	HIST1H3H	histone cluster 1, H3h	Coding
TC1500007885.hg.1	6.4	5.6	1.74	0.0001	0.0006	NEIL1	nei-like DNA glycosylase 1	Multiple_Complex
TC0900009679.hg.1	3.71	4.71	-2.01	0.0001	0.0006	MLLT3	Jeck2013 ALT_ACCEPTOR, ALT_DONOR, codin	NonCoding
TC1800009258.hg.1	6.77	7.33	-1.48	0.0001	0.0006	ZNF519	zinc finger protein 519	Multiple_Complex
TC0500008568.hg.1	6	6.83	-1.78	0.0001	0.0006	SLC12A2	solute carrier family 12 (sodium/potassium/ch	Multiple_Complex
TC0400006579.hg.1	11.25	12.23	-1.96	0.0001	0.0006	ADD1	adducin 1 (alpha)	Multiple_Complex
TC1200010788.hg.1	4.67	5.51	-1.79	0.0001	0.0006	SPRYD3	SPRY domain containing 3	Multiple_Complex
TC0100017722.hg.1	5.85	6.74	-1.85	0.0001	0.0006	C1orf131	chromosome 1 open reading frame 131	Multiple_Complex
TC1200012739.hg.1	7.91	8.93	-2.03	0.0001	0.0006	PARP11	poly(ADP-ribose) polymerase family member	Multiple_Complex
TC1000011524.hg.1	12.76	11.8	1.95	0.0001	0.0006	PIK3AP1	phosphoinositide-3-kinase adaptor protein 1	Multiple_Complex
TC1100013165.hg.1	7.84	8.81	-1.96	0.0001	0.0006	SLC43A3	solute carrier family 43, member 3	Multiple_Complex
TC0100010586.hg.1	3.67	4.26	-1.5	0.0001	0.0006	FMO4	flavin containing monooxygenase 4	Multiple_Complex
TC0400009543.hg.1	4.86	6.08	-2.34	0.0001	0.0006	TLR3	toll-like receptor 3	Multiple_Complex
TC2200008036.hg.1	3.79	8.17	-20.85	0.0001	0.0006	USP41	ubiquitin specific peptidase 41	Multiple_Complex
TC0100016963.hg.1	9.92	9	1.9	0.0001	0.0006	KDM5B	lysine (K)-specific demethylase 5B	Multiple_Complex
TC0500013415.hg.1	7.82	7.11	1.63	0.0001	0.0006	RAB24	RAB24, member RAS oncogene family	Multiple_Complex
TC0100007365.hg.1	5.87	6.58	-1.63	0.0001	0.0006	NIPAL3	NIPA-like domain containing 3	Multiple_Complex
TC2200009317.hg.1	6.41	7.01	-1.52	0.0001	0.0006	KLHL22	kelch-like family member 22	Multiple_Complex
TC1200012863.hg.1	7.61	8.72	-2.17	0.0001	0.0006	CCDC92	coiled-coil domain containing 92	NonCoding
TC0700007137.hg.1	5.2	6.49	-2.44	0.0001	0.0006	BBS9	Bardet-Biedl syndrome 9	Multiple_Complex
TC1500007060.hg.1	4.9	4.1	1.74	0.0001	0.0006	MAP1A	microtubule associated protein 1A	Multiple_Complex
TC0500007868.hg.1	6.93	7.81	-1.85	0.0001	0.0006	SCAMP1	secretory carrier membrane protein 1	Multiple_Complex
TC1400009388.hg.1	9.01	9.78	-1.7	0.0001	0.0006	PPP2R5E	protein phosphatase 2, regulatory subunit B, ε	Multiple_Complex

TC1100011543.hg.1	8.51	7.66	1.8	0.0001	0.0006	LAMTOR1	late endosomal/lysosomal adaptor, MAPK anc	Multiple_Complex
TC1900011716.hg.1	4.6	4.94	-1.27	0.0001	0.0007	LRFN3	leucine rich repeat and fibronectin type III dor	Coding
TC0X00008827.hg.1	9.72	8.75	1.95	0.0001	0.0007	FLNA	Memczak2013 ANTISENSE, CDS, coding, INTEF	NonCoding
TC0900009905.hg.1	4.32	3.79	1.45	0.0001	0.0007	CNTFR	ciliary neurotrophic factor receptor	Multiple_Complex
TC1600011569.hg.1	6.91	7.52	-1.52	0.0001	0.0007	ADAT1	adenosine deaminase, tRNA-specific 1	Multiple_Complex
TC0200016507.hg.1	5.3	4.75	1.46	0.0001	0.0007	LIMS3L; LIN	LIM and senescent cell antigen-like domains 3	Multiple_Complex
TC0900009844.hg.1	9.64	8.64	2.01	0.0001	0.0007	SMU1	smu-1 suppressor of mec-8 and unc-52 homol	Multiple_Complex
TC1200009928.hg.1	3.97	5.15	-2.27	0.0001	0.0007	TAS2R19	taste receptor, type 2, member 19	Coding
TC1900007067.hg.1	6.86	6.05	1.75	0.0001	0.0007	ZNF136	zinc finger protein 136	Multiple_Complex
TC1600007348.hg.1	6.91	8.14	-2.36	0.0001	0.0007	SGF29	SAGA complex associated factor 29	Multiple_Complex
TC1600007234.hg.1	7.3	8.3	-1.99	0.0001	0.0007	DCTN5	dynactin 5 (p25)	Multiple_Complex
TC0100017024.hg.1	5.04	4.42	1.53	0.0001	0.0007	PLEKHA6	Transcript Identified by AceView, Entrez Gene	Unassigned
TC1700012286.hg.1	5.14	6.22	-2.11	0.0001	0.0007	SMG8	SMG8 nonsense mediated mRNA decay factor	Multiple_Complex
TC0600007377.hg.1	9.81	11.58	-3.43	0.0001	0.0007	HIST1H2BN	histone cluster 1, H2bm	Coding
TC0600011878.hg.1	5.52	6.55	-2.04	0.0001	0.0007	XPO5	exportin 5	Multiple_Complex
TC0900010589.hg.1	6.16	5.09	2.1	0.0001	0.0007	HNRNPK	Zhang2013 ALT_ACCEPTOR, ALT_DONOR, cod	NonCoding
TC0200011219.hg.1	4.87	6.32	-2.73	0.0001	0.0007	ACKR3	atypical chemokine receptor 3	Multiple_Complex
TC0400009818.hg.1	10.64	9.98	1.59	0.0001	0.0007	LRPAP1	LDL receptor related protein associated protei	Multiple_Complex
TC1700007016.hg.1	6.89	7.63	-1.67	0.0001	0.0007	TRPV2	transient receptor potential cation channel, su	Multiple_Complex
TC1500010893.hg.1	6.86	7.69	-1.78	0.0001	0.0007	PTPN9	protein tyrosine phosphatase, non-receptor ty	Multiple_Complex
TC0900011523.hg.1	12.66	11.64	2.02	0.0001	0.0007	HSPA5	heat shock 70kDa protein 5 (glucose-regulatec	Multiple_Complex
TC1100009840.hg.1	6.82	7.55	-1.66	0.0001	0.0007	ZNF195	zinc finger protein 195	Multiple_Complex
TSUnmapped00000527.h	4.81	5.72	-1.87	0.0001	0.0007	LRIG1		Coding
TC0300007617.hg.1	6.55	7.5	-1.93	0.0001	0.0007	KCTD6	potassium channel tetramerization domain co	Multiple_Complex
TC1900009186.hg.1	5.29	4.68	1.53	0.0001	0.0007	ATP8B3	ATPase, aminophospholipid transporter, class	Multiple_Complex
TSUnmapped00000450.h	6.22	5.34	1.84	0.0001	0.0007	RPS6KA1	ribosomal protein S6 kinase, 90kDa, polypepti	NonCoding
TC0X00008070.hg.1	4.09	4.8	-1.63	0.0001	0.0007	FRMPD3	FERM and PDZ domain containing 3	Multiple_Complex
TC0800008194.hg.1	4.6	3.91	1.62	0.0001	0.0007	NECAB1	N-terminal EF-hand calcium binding protein 1	Multiple_Complex
TC0600010066.hg.1	12.22	10.63	3.01	0.0001	0.0007	IGF2R	insulin-like growth factor 2 receptor	Multiple_Complex
TC0100008779.hg.1	6.44	5.7	1.66	0.0001	0.0007	ST6GALNAC	ST6 (alpha-N-acetyl-neuraminyl-2,3-beta-gala	Multiple_Complex
TC1600008661.hg.1	12.15	9.91	4.71	0.0001	0.0007	CRISPLD2	cysteine-rich secretory protein LCCL domain c	Multiple_Complex
TC0700008246.hg.1	11.16	11.93	-1.7	0.0001	0.0007	DMTF1	cyclin D binding myb-like transcription factor	Multiple_Complex
TC0100018499.hg.1	5.64	5.02	1.54	0.0001	0.0007	MUC1	mucin 1, cell surface associated	Multiple_Complex
TC0700006945.hg.1	6.52	5.38	2.2	0.0001	0.0007	MPP6	membrane protein, palmitoylated 6	Multiple_Complex
TC0300009727.hg.1	5.14	6.05	-1.88	0.0001	0.0007	VPS8	Transcript Identified by AceView, Entrez Gene	Unassigned
TC1800008162.hg.1	5.13	5.83	-1.62	0.0001	0.0007	FAM210A	family with sequence similarity 210, member	Multiple_Complex
TC2200008881.hg.1	5.41	6.41	-2.01	0.0001	0.0007	RRP7A	ribosomal RNA processing 7 homolog A	Multiple_Complex
TC1800007696.hg.1	5.25	5.96	-1.64	0.0001	0.0007	TSHZ1	teashirt zinc finger homeobox 1	Multiple_Complex
TC0200009923.hg.1	5.79	6.56	-1.7	0.0001	0.0007	UBR3	Transcript Identified by AceView, Entrez Gene	Unassigned
TC1300008892.hg.1	7.72	9.76	-4.12	0.0001	0.0007	LPAR6	lysophosphatidic acid receptor 6	Multiple_Complex
TC1000010362.hg.1	5.15	6.01	-1.81	0.0001	0.0007	ZNF248	zinc finger protein 248	Multiple_Complex
TC0400008228.hg.1	6.16	7.12	-1.95	0.0001	0.0007	METAP1	methionyl aminopeptidase 1	Multiple_Complex
TC1000012482.hg.1	12.48	11.26	2.32	0.0001	0.0007	ENTPD1	ectonucleoside triphosphate diphosphohydrol	Multiple_Complex
TC0600010609.hg.1	4.35	3.44	1.88	0.0001	0.0007	PXDC1	PX domain containing 1	Multiple_Complex
TC2200009233.hg.1	9	8.51	1.4	0.0001	0.0007	ADORA2A	adenosine A2a receptor	Multiple_Complex
TC0700010796.hg.1	10.15	10.88	-1.66	0.0001	0.0007	VPS41	vacuolar protein sorting 41 homolog (S. cerevi	Multiple_Complex
TC1200007182.hg.1	9.86	8.62	2.37	0.0001	0.0007	FAR2	fatty acyl-CoA reductase 2	Multiple_Complex
TC1500009438.hg.1	6.47	7.55	-2.11	0.0001	0.0007	LYSMD2	LysM, putative peptidoglycan-binding, domair	Coding
TC0800007681.hg.1	7	7.73	-1.67	0.0001	0.0007	TGS1	trimethylguanosine synthase 1	Multiple_Complex
TC1100006500.hg.1	8.72	9.46	-1.66	0.0001	0.0007	AP2A2	adaptor-related protein complex 2, alpha 2 su	Multiple_Complex
TC0600011853.hg.1	5.1	4.44	1.59	0.0001	0.0007	MEA1	male-enhanced antigen 1	Multiple_Complex
TC0100009331.hg.1	10.34	9.24	2.16	0.0001	0.0007	STXBP3	syntaxin binding protein 3	Multiple_Complex
TC1200011916.hg.1	11.91	12.83	-1.88	0.0001	0.0007	PPP1CC	protein phosphatase 1, catalytic subunit, gam	Multiple_Complex
TC1500010776.hg.1	10.87	10.25	1.53	0.0001	0.0007	IDH3A	isocitrate dehydrogenase 3 (NAD+) alpha	Multiple_Complex
TC0600008972.hg.1	12.72	13.97	-2.37	0.0001	0.0007	PRDM1	PR domain containing 1, with ZNF domain	Multiple_Complex
TC2000008072.hg.1	7.03	8.2	-2.25	0.0001	0.0007	PPDPF	pancreatic progenitor cell differentiation and	Multiple_Complex
TC0900012295.hg.1	5.72	6.47	-1.67	0.0001	0.0007	TMEM203	transmembrane protein 203	Coding
TC0600011248.hg.1	4.2	4.84	-1.55	0.0001	0.0007	ZKSCAN4	zinc finger with KRAB and SCAN domains 4	Multiple_Complex
TC0400012015.hg.1	6.56	7.89	-2.51	0.0001	0.0007	GYPE	glycophorin E (MNS blood group)	Multiple_Complex
TC0500009010.hg.1	4.88	5.89	-2.01	0.0001	0.0007	TCERG1	Transcript Identified by AceView, Entrez Gene	Unassigned
TC0100013629.hg.1	6.47	7.36	-1.85	0.0001	0.0007	YARS	tyrosyl-tRNA synthetase	Multiple_Complex
TC0500011483.hg.1	10.92	9.83	2.12	0.0001	0.0007	MCTP1	multiple C2 domains, transmembrane 1	Multiple_Complex
TC0200013104.hg.1	7.91	8.38	-1.39	0.0001	0.0007	MOGS	mannosyl-oligosaccharide glucosidase	Multiple_Complex
TC0400010484.hg.1	10.03	10.75	-1.65	0.0001	0.0007	PDS5A	PDS5 cohesin associated factor A	Multiple_Complex
TC0200007446.hg.1	8.92	9.99	-2.11	0.0001	0.0007	PRKCE	protein kinase C, epsilon	Multiple_Complex
TC1500010737.hg.1	9.01	8.27	1.68	0.0001	0.0007	SQRDL	sulfide quinone reductase-like (yeast)	Multiple_Complex
TC0400008785.hg.1	6.36	5.77	1.51	0.0001	0.0007	NOCT	nocturnin	Multiple_Complex
TC0300011540.hg.1	5.85	6.74	-1.86	0.0001	0.0007	SHQ1	SHQ1, H/ACA ribonucleoprotein assembly fact	Multiple_Complex
TC1200007406.hg.1	9.23	10.13	-1.86	0.0001	0.0007	IRAK4	interleukin 1 receptor associated kinase 4	Multiple_Complex
TC1200012864.hg.1	7.96	8.73	-1.71	0.0001	0.0008	CCDC92	coiled-coil domain containing 92	Multiple_Complex

TC1100013227.hg.1	3.18	3.86	-1.59	0.0001	0.0008	FDXACB1	ferredoxin-fold anticodon binding domain con	Multiple_Complex
TC2200007271.hg.1	13.47	12.34	2.19	0.0001	0.0008	NCF4	neutrophil cytosolic factor 4	Multiple_Complex
TC0500013296.hg.1	5.76	6.37	-1.53	0.0001	0.0008	C1QTNF3-A	C1QTNF3-AMACR readthrough (NMD candida	Multiple_Complex
TC0800009236.hg.1	6.57	5.7	1.83	0.0001	0.0008	EXOSC4; M	exosome component 4; microRNA 6847	Multiple_Complex
TC0800009819.hg.1	10.75	11.72	-1.96	0.0001	0.0008	DOK2	docking protein 2	Multiple_Complex
TC0300006471.hg.1	4.76	5.41	-1.57	0.0001	0.0008	SETMAR	SET domain and mariner transposase fusion g	Multiple_Complex
TC0X00007540.hg.1	4.97	5.74	-1.71	0.0001	0.0008	DLG3	discs, large homolog 3 (Drosophila)	Multiple_Complex
TC0100017668.hg.1	6.28	7.27	-1.98	0.0001	0.0008	TAF5L	TAF5-like RNA polymerase II, p300/CBP-associ	Multiple_Complex
TC0400009518.hg.1	5.68	6.24	-1.48	0.0001	0.0008	SLC25A4	solute carrier family 25 (mitochondrial carrier,	Multiple_Complex
TC1200012706.hg.1	5.66	6.55	-1.85	0.0001	0.0008	ACAD10	acyl-CoA dehydrogenase family, member 10	Multiple_Complex
TC1400009426.hg.1	13.02	12.22	1.75	0.0001	0.0008	MAX	MYC associated factor X	Multiple_Complex
TC0300006544.hg.1	7.46	8.14	-1.6	0.0001	0.0008	SETD5	SET domain containing 5	Multiple_Complex
TC1900007004.hg.1	5.75	6.42	-1.6	0.0001	0.0008	C19orf52	chromosome 19 open reading frame 52	Multiple_Complex
TC0400012880.hg.1	6.41	5.34	2.1	0.0001	0.0008	DUX4	double homeobox 4	Multiple_Complex
TC0100015796.hg.1	7.47	6.01	2.74	0.0001	0.0008	POGZ	Transcript Identified by AceView, Entrez Gene	Unassigned
TC1100009026.hg.1	7.09	7.55	-1.38	0.0001	0.0008	SIK2	salt-inducible kinase 2	Multiple_Complex
TC0100017436.hg.1	4.5	5.58	-2.12	0.0001	0.0008	TAF1A	TATA box binding protein (TBP)-associated fac	Multiple_Complex
TSUnmapped00000223.h	4.95	4.72	1.17	0.0001	0.0008	RPS6KA1	ribosomal protein S6 kinase, 90kDa, polypepti	NonCoding
TC1600006666.hg.1	8.26	9.08	-1.76	0.0001	0.0008	CASP16P	caspase 16, pseudogene	Multiple_Complex
TC1900007831.hg.1	5.29	5.85	-1.47	0.0001	0.0008	HPN	hepsin	Multiple_Complex
TC1200007897.hg.1	8.6	9.31	-1.63	0.0001	0.0008	TSPAN31	tetraspanin 31	Multiple_Complex
TC1600011426.hg.1	9.3	8.63	1.58	0.0001	0.0008	ATXN1L	ataxin 1-like	Multiple_Complex
TC0300009701.hg.1	8.44	7.88	1.47	0.0001	0.0008	PSMD2	proteasome 26S subunit, non-ATPase 2	Multiple_Complex
TC1000009612.hg.1	14.72	13.73	1.98	0.0001	0.0008	KLF6	Kruppel-like factor 6	Multiple_Complex
TC0200007872.hg.1	5.32	6.32	-2	0.0001	0.0008	ETAA1	Ewing tumor-associated antigen 1	Multiple_Complex
TC1300007565.hg.1	8.93	7.85	2.11	0.0001	0.0008	SLAIN1	SLAIN motif family member 1	Multiple_Complex
TC2000007519.hg.1	5.9	6.74	-1.8	0.0001	0.0008	CD40	CD40 molecule, TNF receptor superfamily mer	Multiple_Complex
TC1500010733.hg.1	4.96	4.39	1.48	0.0001	0.0008	C15orf48; I	chromosome 15 open reading frame 48; micro	Multiple_Complex
TC1600010778.hg.1	3.95	3.39	1.47	0.0001	0.0008	HYDIN	HYDIN, axonemal central pair apparatus prote	Multiple_Complex
TC0X00009980.hg.1	7.61	6.65	1.95	0.0001	0.0008	CXorf65	chromosome X open reading frame 65	Multiple_Complex
TC0200015231.hg.1	6.34	6.85	-1.43	0.0001	0.0008	NEMP2	nuclear envelope integral membrane protein 2	Multiple_Complex
TC1700008326.hg.1	5.87	5.02	1.81	0.0001	0.0008	TOM1L1	target of myb1 like 1 membrane trafficking pr	Multiple_Complex
TC0800012301.hg.1	6.07	5.5	1.49	0.0001	0.0008	FAM110B	family with sequence similarity 110, member	Multiple_Complex
TC1100012315.hg.1	6.61	5.88	1.65	0.0001	0.0008	TIMM8B	translocase of inner mitochondrial membrane	Multiple_Complex
TC0900008178.hg.1	7.31	6.58	1.66	0.0001	0.0008	NANS	N-acetylneuraminic acid synthase	Multiple_Complex
TC1500007874.hg.1	8.75	9.99	-2.36	0.0001	0.0008	C15orf39	chromosome 15 open reading frame 39	Coding
TC0500010497.hg.1	5.96	6.71	-1.68	0.0001	0.0008	NADK2	NAD kinase 2, mitochondrial	Multiple_Complex
TC0600008569.hg.1	4.34	5.35	-2	0.0001	0.0008	MYO6	myosin VI	Multiple_Complex
TC1600009183.hg.1	6.19	7.03	-1.8	0.0001	0.0008	ZNF200	Transcript Identified by AceView, Entrez Gene	Unassigned
TC0X00008884.hg.1	8.81	7.88	1.9	0.0002	0.0008	VAMP7	vesicle associated membrane protein 7	Multiple_Complex
TC1100008661.hg.1	6.41	7.45	-2.06	0.0002	0.0008	EED; MIR67	embryonic ectoderm development; microRNA	Multiple_Complex
TC0200012648.hg.1	10.73	11.25	-1.44	0.0002	0.0008	PPP4R3B	protein phosphatase 4, regulatory subunit 3B	Multiple_Complex
TC0900008316.hg.1	4.71	5.58	-1.84	0.0002	0.0008	FKTN	fukutin	Multiple_Complex
TC1400006718.hg.1	10.1	11.33	-2.35	0.0002	0.0008	PSME1	proteasome activator subunit 1	Multiple_Complex
TC1700011128.hg.1	11.39	10.53	1.82	0.0002	0.0008	SPAG9	sperm associated antigen 9	Multiple_Complex
TC0800012299.hg.1	9.34	8.58	1.69	0.0002	0.0008	HOOK3	hook microtubule-tethering protein 3	Multiple_Complex
TC0200013330.hg.1	5.59	6.64	-2.07	0.0002	0.0008	PLGLB1; PL	plasminogen-like B1; plasminogen-like B2	Multiple_Complex
TC0100010389.hg.1	6.2	6.94	-1.67	0.0002	0.0008	HSD17B7	hydroxysteroid (17-beta) dehydrogenase 7	Multiple_Complex
TC1400010755.hg.1	10.23	9.04	2.27	0.0002	0.0008	PSMA3-AS1	PSMA3 antisense RNA 1	Multiple_Complex
TC0500013229.hg.1	7.47	8.02	-1.46	0.0002	0.0008	C5orf24	chromosome 5 open reading frame 24	Multiple_Complex
TC0600007836.hg.1	8.61	7.97	1.56	0.0002	0.0008	KCTD20	potassium channel tetramerization domain co	Multiple_Complex
TC1500009395.hg.1	6.82	7.89	-2.09	0.0002	0.0008	TRPM7	transient receptor potential cation channel, su	Multiple_Complex
TC0100018280.hg.1	12.76	13.73	-1.96	0.0002	0.0008	FAM231D;	family with sequence similarity 231, member	Multiple_Complex
TC0600014204.hg.1	5.65	7.14	-2.83	0.0002	0.0008	CCR6	chemokine (C-C motif) receptor 6	Coding
TC1400010757.hg.1	5.73	6.28	-1.47	0.0002	0.0008	L3HYPDH	L-3-hydroxyproline dehydratase (trans-)	Multiple_Complex
TC1100009709.hg.1	5.27	6.11	-1.79	0.0002	0.0008	CHID1	chitinase domain containing 1	Multiple_Complex
TC1100011241.hg.1	12.27	11.59	1.6	0.0002	0.0008	MAP3K11	mitogen-activated protein kinase kinase kinas	Multiple_Complex
TC1100011375.hg.1	5.91	6.94	-2.04	0.0002	0.0008	C11orf24	chromosome 11 open reading frame 24	Multiple_Complex
TC0100015803.hg.1	5.83	6.64	-1.75	0.0002	0.0008	TDRKH	tudor and KH domain containing	Multiple_Complex
TC0400012854.hg.1	5.47	6.31	-1.8	0.0002	0.0008	TMEM144	transmembrane protein 144	Multiple_Complex
TC1900008505.hg.1	9.16	9.95	-1.72	0.0002	0.0008	BAX	BCL2-associated X protein	Multiple_Complex
TC1200012161.hg.1	4.85	4.4	1.37	0.0002	0.0008	TRIAP1	TP53 regulated inhibitor of apoptosis 1	Multiple_Complex
TC0200013324.hg.1	4.47	5.45	-1.97	0.0002	0.0008	CD8B	CD8b molecule	Coding
TC1600011147.hg.1	5.8	6.7	-1.87	0.0002	0.0009	MTHFSD	methenyltetrahydrofolate synthetase domain	Multiple_Complex
TC0600013898.hg.1	6.8	5.87	1.91	0.0002	0.0009	FAM103A1	Homo sapiens family with sequence similarity	Multiple_Complex
TC0100009800.hg.1	7.4	8.27	-1.82	0.0002	0.0009	GPR89B	G protein-coupled receptor 89B	Multiple_Complex
TC1200010454.hg.1	8.69	7.25	2.72	0.0002	0.0009	ZCRB1; MIF	zinc finger CCHC-type and RNA binding motif 1	Multiple_Complex
TC1700007520.hg.1	6.07	5.73	1.26	0.0002	0.0009	CDK5R1	cyclin-dependent kinase 5, regulatory subunit	Multiple_Complex
TC1900012018.hg.1	6.28	6.98	-1.62	0.0002	0.0009	ZNF577	zinc finger protein 577	Multiple_Complex
TC0300011077.hg.1	7.44	8.2	-1.7	0.0002	0.0009	IP6K1	inositol hexakisphosphate kinase 1	Multiple_Complex

TC0100012465.hg.1	7.34	8.03	-1.6	0.0002	0.0009	CPSF3L; MI	cleavage and polyadenylation specific factor 3	Multiple_Complex
TC0900011445.hg.1	7.36	6.63	1.66	0.0002	0.0009	PDCL	phosducin like	Coding
TC0100018261.hg.1	6.09	7.53	-2.72	0.0002	0.0009	GSTM4	glutathione S-transferase mu 4	Multiple_Complex
TC0300012686.hg.1	8.27	9.21	-1.92	0.0002	0.0009	SLC9A9	solute carrier family 9, subfamily A (NHE9, cat	Multiple_Complex
TC1500007529.hg.1	12.78	12.05	1.66	0.0002	0.0009	USP3	ubiquitin specific peptidase 3	Multiple_Complex
TC0100017321.hg.1	5.83	6.49	-1.58	0.0002	0.0009	GPATCH2	G-patch domain containing 2	Multiple_Complex
TC1100007430.hg.1	7.48	8.12	-1.56	0.0002	0.0009	GYLTL1B	glycosyltransferase-like 1B	Multiple_Complex
TC0900007994.hg.1	7.61	8.49	-1.84	0.0002	0.0009	PHF2	PHD finger protein 2	Multiple_Complex
TC1900007695.hg.1	8.8	9.74	-1.92	0.0002	0.0009	URI1	URI1, prefoldin-like chaperone	Multiple_Complex
TC0100013992.hg.1	5.89	4.86	2.05	0.0002	0.0009	TCTEX1D4	Tctex1 domain containing 4	Coding
TC1100007448.hg.1	6.39	7.3	-1.87	0.0002	0.0009	DGKZ	diacylglycerol kinase, zeta	Multiple_Complex
TC0X00008751.hg.1	6.24	6.82	-1.49	0.0002	0.0009	MAGEA2; M	MAGE family member A2; MAGE family meml	Multiple_Complex
TC1900008543.hg.1	7.53	6.75	1.72	0.0002	0.0009	FCGRT	Fc fragment of IgG, receptor, transporter, alph	Multiple_Complex
TC1700012295.hg.1	5.31	6.18	-1.83	0.0002	0.0009	ARSG	arylsulfatase G	Multiple_Complex
TC1400010577.hg.1	6.69	5.51	2.28	0.0002	0.0009	NGDN	neuroguidin, EIF4E binding protein	Multiple_Complex
TC0300008755.hg.1	4.84	4.21	1.54	0.0002	0.0009	GP9	glycoprotein IX (platelet)	Coding
TC1000010916.hg.1	4.5	3.92	1.5	0.0002	0.0009	NPFFR1	neuropeptide FF receptor 1	Coding
TC0300006846.hg.1	13.1	14.36	-2.39	0.0002	0.0009	RPL15	ribosomal protein L15	Multiple_Complex
TC0700006466.hg.1	5.03	5.77	-1.67	0.0002	0.0009	DNAAF5	dynein, axonemal, assembly factor 5	Multiple_Complex
TC0900006758.hg.1	8.34	9.23	-1.85	0.0002	0.0009	DENND4C	DENN/MADD domain containing 4C	Multiple_Complex
TC0800010034.hg.1	11.06	10.07	1.98	0.0002	0.0009	GSR	glutathione reductase	Multiple_Complex
TC0100010454.hg.1	7.15	8.15	-2	0.0002	0.0009	MGST3	microsomal glutathione S-transferase 3	Multiple_Complex
TC0100012008.hg.1	5.54	4.9	1.56	0.0002	0.0009	KCNK1	Transcript Identified by AceView, Entrez Gene	Unassigned
TC1000007852.hg.1	13.56	12.55	2.02	0.0002	0.0009	HNRNPH3	heterogeneous nuclear ribonucleoprotein H3	Multiple_Complex
TC0Y00007219.hg.1	4.79	3.58	2.31	0.0002	0.0009	DAZ4; DAZ	deleted in azoospermia 4; deleted in azoosper	Multiple_Complex
TC0200014509.hg.1	9.9	9.02	1.84	0.0002	0.0009	GTDC1	glycosyltransferase like domain containing 1	Multiple_Complex
TC0100018233.hg.1	5.49	6.31	-1.77	0.0002	0.0009	FPGT	fucose-1-phosphate guanylyltransferase	Coding
TC1800008680.hg.1	5.81	6.8	-1.98	0.0002	0.0009	CXXC1	CXXC finger protein 1	Multiple_Complex
TC1100006771.hg.1	9.83	11.14	-2.49	0.0002	0.0009	EIF3F	eukaryotic translation initiation factor 3, subu	Multiple_Complex
TC0800008946.hg.1	4.93	4.45	1.4	0.0002	0.0009	TG	thyroglobulin	Multiple_Complex
TC0100009651.hg.1	6.44	6.98	-1.45	0.0002	0.0009	PPIAL4A	peptidylprolyl isomerase A (cyclophilin A)-like	Coding
TC0700013490.hg.1	5.71	6.27	-1.48	0.0002	0.0009	ZNF775	zinc finger protein 775	Multiple_Complex
TC0600008120.hg.1	7.02	8.54	-2.85	0.0002	0.0009	TMEM63B	transmembrane protein 63B	Multiple_Complex
TC1000010265.hg.1	13.96	12.93	2.04	0.0002	0.0009	ITGB1	integrin beta 1	Multiple_Complex
TC0100018471.hg.1	5.71	6.3	-1.51	0.0002	0.0009	WARS2	tryptophanyl tRNA synthetase 2, mitochondri	Multiple_Complex
TC1200009253.hg.1	6.85	7.77	-1.9	0.0002	0.0009	ATP6V0A2	ATPase, H+ transporting, lysosomal V0 subuni	Multiple_Complex
TC1900006994.hg.1	9.52	8.75	1.7	0.0002	0.0009	DNM2	dynamin 2	Multiple_Complex
TC0300012132.hg.1	12.19	11.48	1.63	0.0002	0.0009	GSK3B	glycogen synthase kinase 3 beta	Multiple_Complex
TC1800008707.hg.1	7.76	8.44	-1.6	0.0002	0.0009	MEX3C	mex-3 RNA binding family member C	Multiple_Complex
TC2000008381.hg.1	6.02	5.39	1.55	0.0002	0.0009	JAG1	jagged 1	Multiple_Complex
TC0400007095.hg.1	4.9	5.64	-1.67	0.0002	0.0009	ZCCHC4	zinc finger, CCHC domain containing 4	Multiple_Complex
TC1600011235.hg.1	9.21	8.2	2.02	0.0002	0.0009	GALNS	galactosamine (N-acetyl)-6-sulfatase	Multiple_Complex
TC0400012251.hg.1	6.94	6.43	1.42	0.0002	0.0009	C4orf46	chromosome 4 open reading frame 46	Multiple_Complex
TC2000008345.hg.1	9.63	8.63	2	0.0002	0.0009	TMX4	thioredoxin-related transmembrane protein 4	Multiple_Complex
TC1600009949.hg.1	5.07	4.35	1.66	0.0002	0.0009	FAM57B	family with sequence similarity 57, member B	Multiple_Complex
TC1400009124.hg.1	5.03	5.51	-1.39	0.0002	0.0009	L2HGDH	L-2-hydroxyglutarate dehydrogenase	Multiple_Complex
TC0X00009427.hg.1	7.07	7.95	-1.84	0.0002	0.0009	BCOR	BCL6 corepressor	Multiple_Complex
TC2200008887.hg.1	6.2	5.21	1.98	0.0002	0.0009	ATP5L2	ATP synthase, H+ transporting, mitochondrial	Coding
TC1400008694.hg.1	8.37	8.98	-1.52	0.0002	0.0009	ACIN1	apoptotic chromatin condensation inducer 1	Multiple_Complex
TC1200010900.hg.1	12.36	12.96	-1.51	0.0002	0.0009	SMARCC2	SWI/SNF related, matrix associated, actin dep	Multiple_Complex
TC0600013033.hg.1	5.83	6.53	-1.63	0.0002	0.0009	TBC1D32	TBC1 domain family, member 32	Multiple_Complex
TC1100013004.hg.1	6.09	6.95	-1.82	0.0002	0.0009	CTNND1	catenin (cadherin-associated protein), delta 1	Multiple_Complex
TC1200012657.hg.1	6.82	7.71	-1.85	0.0002	0.0009	CAND1	cullin-associated and neddylation-dissociated	Multiple_Complex
TC1500010941.hg.1	4.63	5.25	-1.54	0.0002	0.0009	LYSMD4	LysM, putative peptidoglycan-binding, domair	Coding
TC1900009866.hg.1	7.58	6.95	1.55	0.0002	0.0009	AKAP8	A kinase (PRKA) anchor protein 8	Multiple_Complex
TC0300013989.hg.1	6.79	7.45	-1.58	0.0002	0.001	ABHD14B	abhydrolase domain containing 14B	Multiple_Complex
TC1400007112.hg.1	6.16	6.95	-1.74	0.0002	0.001	ATP5S	ATP synthase, H+ transporting, mitochondrial	Multiple_Complex
TC1100008985.hg.1	12.16	13.12	-1.94	0.0002	0.001	ATM	ATM serine/threonine kinase	Multiple_Complex
TC0800010002.hg.1	5.32	4.69	1.55	0.0002	0.001	DUSP4	dual specificity phosphatase 4	Multiple_Complex
TC1900009867.hg.1	10.13	9.15	1.97	0.0002	0.001	AKAP8L	A kinase (PRKA) anchor protein 8-like	Multiple_Complex
TC0100018486.hg.1	13.48	14.13	-1.57	0.0002	0.001	CDC42SE1	CDC42 small effector 1	Multiple_Complex
TSUnmapped00000523.h	8.02	6.96	2.09	0.0002	0.001	RPS25	ribosomal protein S25	NonCoding
TC0400009681.hg.1	6.37	7.37	-1.99	0.0002	0.001	ZNF721; AE	zinc finger protein 721; ATP binding cassette s	Multiple_Complex
TC1600010711.hg.1	7.79	8.62	-1.78	0.0002	0.001	CHTF8	chromosome transmission fidelity factor 8	Multiple_Complex
TC1100008002.hg.1	5.93	7.13	-2.29	0.0002	0.001	MRPL49	mitochondrial ribosomal protein L49	Multiple_Complex
TC1400010593.hg.1	9.34	8.8	1.45	0.0002	0.001	FAM177A1	family with sequence similarity 177, member	Multiple_Complex
TC0X00010346.hg.1	8.31	8.75	-1.35	0.0002	0.001	TRMT2B	tRNA methyltransferase 2 homolog B	Multiple_Complex
TC1100011735.hg.1	6.15	7.12	-1.97	0.0002	0.001	INTS4	integrator complex subunit 4	Multiple_Complex
TC2200009262.hg.1	9.5	10.5	-2	0.0002	0.001	EIF3L	eukaryotic translation initiation factor 3, subu	Multiple_Complex
TC0100016020.hg.1	8.93	9.63	-1.63	0.0002	0.001	ISG20L2	interferon stimulated exonuclease gene 20kD	Multiple_Complex

TC0100015660.hg.1	6.86	7.68	-1.76	0.0002	0.001	PPIAL4G	peptidylprolyl isomerase A (cyclophilin A)-like	Coding
TC0900008173.hg.1	11.38	12.35	-1.95	0.0002	0.001	ANP32B	acidic nuclear phosphoprotein 32 family mem	Multiple_Complex
TC1500007009.hg.1	7.24	8.27	-2.05	0.0002	0.001	MGA	MGA, MAX dimerization protein	Multiple_Complex
TC1800008607.hg.1	5.71	5.14	1.49	0.0002	0.001	TCEB3CL2;	transcription elongation factor B polypeptide :	Coding
TC1700011561.hg.1	9	8.1	1.86	0.0002	0.001	WIPI1	WD repeat domain, phosphoinositide interact	Multiple_Complex
TC0X00010434.hg.1	4.86	5.55	-1.61	0.0002	0.001	SLC25A53	solute carrier family 25, member 53	Multiple_Complex
TC0200016544.hg.1	5.83	6.8	-1.96	0.0002	0.001	RIF1	replication timing regulatory factor 1	NonCoding
TC1000011605.hg.1	7.8	6.77	2.04	0.0002	0.001	CWF19L1; S	CWF19-like 1, cell cycle control (S. pombe); sn	Multiple_Complex
TC0400012019.hg.1	4.85	6.6	-3.37	0.0002	0.001	GYPA	glycophorin A (MNS blood group)	Multiple_Complex
TC1000012430.hg.1	3.44	4.51	-2.1	0.0002	0.001	RP11-295P	novel transcript; Transcript Identified by AceV	NonCoding
TC0300010066.hg.1	9.97	9.08	1.86	0.0002	0.001	FYTTD1	forty-two-three domain containing 1	Multiple_Complex
TC0100016112.hg.1	15.24	13.98	2.4	0.0002	0.001	TAGLN2	transgelin 2	Multiple_Complex
TC1200009111.hg.1	5.36	6.19	-1.78	0.0002	0.001	CCDC64	coiled-coil domain containing 64	Multiple_Complex
TC1700010701.hg.1	7.36	6.68	1.6	0.0002	0.001	DNAJC7	DnaJ (Hsp40) homolog, subfamily C, member	NonCoding
TC1900011871.hg.1	4.36	4.94	-1.5	0.0002	0.001	ZNF653	zinc finger protein 653	Multiple_Complex
TC2200006641.hg.1	6.29	7.13	-1.79	0.0002	0.001	RANBP1	RAN binding protein 1	Multiple_Complex
TC0X00007302.hg.1	4.86	5.31	-1.36	0.0002	0.001	FAM156B;	family with sequence similarity 156, member	Multiple_Complex
TC1200012835.hg.1	7.39	6.17	2.33	0.0002	0.001	NT5DC3	5-nucleotidase domain containing 3	Multiple_Complex
TC1600011371.hg.1	6.8	7.38	-1.5	0.0002	0.001	SLX1A; SLX	SLX1 homolog A, structure-specific endonucle	Multiple_Complex
TC2000008951.hg.1	5.34	5.97	-1.55	0.0002	0.001	GSS	glutathione synthetase	Multiple_Complex
TC0900010762.hg.1	8.21	8.85	-1.55	0.0002	0.001	AUH	AU RNA binding protein/enoyl-CoA hydratase	Multiple_Complex
TC0300011151.hg.1	10.38	9.31	2.1	0.0002	0.001	RPL29	ribosomal protein L29	Multiple_Complex
TC0700010398.hg.1	6.05	5.48	1.48	0.0002	0.001	TMEM196	transmembrane protein 196	Coding
TC0X00009384.hg.1	6.32	5.58	1.67	0.0002	0.001	DYNLT3	dynein, light chain, Tctex-type 3	Coding
TC0500011121.hg.1	3.93	3.39	1.45	0.0002	0.001	FOXD1	forkhead box D1	Multiple_Complex
TC0100009449.hg.1	6.42	7.92	-2.83	0.0002	0.001	MOV10	Mov10 RISC complex RNA helicase	Multiple_Complex
TC1100008054.hg.1	8.16	8.94	-1.71	0.0002	0.001	PACS1	phosphofurin acidic cluster sorting protein 1	Multiple_Complex
TC1700009900.hg.1	3.81	4.93	-2.18	0.0002	0.001	ZNF287	zinc finger protein 287	Multiple_Complex
TC0600008447.hg.1	4.58	3.98	1.52	0.0002	0.001	ADGRB3	adhesion G protein-coupled receptor B3	Multiple_Complex
TC0600008064.hg.1	7.53	8.59	-2.08	0.0002	0.001	PPP2R5D	protein phosphatase 2, regulatory subunit B, c	Multiple_Complex
TC2100007883.hg.1	4	3.52	1.39	0.0002	0.001	KRTAP24-1	keratin associated protein 24-1	Coding
TC1200006737.hg.1	7.7	8.35	-1.57	0.0002	0.001	PHC1	polyhomeotic homolog 1 (Drosophila)	Multiple_Complex
TC1600009646.hg.1	4.96	5.82	-1.81	0.0002	0.001	KNOP1	lysine-rich nucleolar protein 1	Multiple_Complex
TC0200016682.hg.1	13.23	11.92	2.48	0.0002	0.001	PPP3R1	protein phosphatase 3, regulatory subunit B, a	Coding
TC0X00007199.hg.1	10.64	11.96	-2.5	0.0002	0.001	WAS	Wiskott-Aldrich syndrome	Multiple_Complex
TC0700008090.hg.1	10.46	11.88	-2.67	0.0002	0.001	HSPB1	heat shock 27kDa protein 1	Multiple_Complex
TC0300010082.hg.1	10.28	9.04	2.36	0.0002	0.001	FAM157A	family with sequence similarity 157, member .	Multiple_Complex
TC0400006487.hg.1	8.7	9.3	-1.52	0.0002	0.001	TMEM175	transmembrane protein 175	Multiple_Complex
TC0900009569.hg.1	5.77	4.52	2.38	0.0002	0.001	NFIB	nuclear factor I/B	Multiple_Complex
TC0700008565.hg.1	8.37	7.27	2.15	0.0002	0.001	SLC12A9	solute carrier family 12, member 9	Multiple_Complex
TC2000007277.hg.1	12.08	12.63	-1.46	0.0002	0.001	RPN2	ribophorin II	Multiple_Complex
TC2000008217.hg.1	5.37	4.85	1.43	0.0002	0.001	SLC4A11	solute carrier family 4, sodium borate transpo	Multiple_Complex
TC0800012213.hg.1	7.25	8.47	-2.33	0.0002	0.001	VPS28	vacuolar protein sorting 28 homolog (S. cerevi	Multiple_Complex
TC1000007596.hg.1	8.82	9.4	-1.5	0.0002	0.001	TIMM23B	translocase of inner mitochondrial membrane	Multiple_Complex
TSUnmapped00000243.h	10.79	9.21	3	0.0002	0.001	MANSC1	MANSC domain containing 1	Coding
TC1500010906.hg.1	5.87	6.65	-1.72	0.0002	0.001	RP11-351M	Transcript Identified by AceView, Entrez Gene	Multiple_Complex
TC1700007942.hg.1	8.74	9.29	-1.47	0.0002	0.001	NBR1	neighbor of BRCA1 gene 1	Multiple_Complex
TC1200011671.hg.1	11.21	10.39	1.77	0.0002	0.001	UHRF1BP1L	UHRF1 binding protein 1-like	Multiple_Complex
TC1700010707.hg.1	7.13	8.56	-2.7	0.0002	0.001	DHX58	DEXH (Asp-Glu-X-His) box polypeptide 58	Multiple_Complex
TC1100010897.hg.1	12.61	14.72	-4.32	0.0002	0.0011	UBE2L6	ubiquitin-conjugating enzyme E2L 6	Multiple_Complex
TC0400012375.hg.1	6.73	7.66	-1.91	0.0002	0.0011	CBR4	carbonyl reductase 4	Multiple_Complex
TC1100009940.hg.1	5.25	6.64	-2.62	0.0002	0.0011	TRIM5	tripartite motif containing 5	Multiple_Complex
TC0700010941.hg.1	10.12	10.85	-1.65	0.0002	0.0011	MYO1G	myosin IG	Multiple_Complex
TC0100009959.hg.1	5.85	5.15	1.62	0.0002	0.0011	TUFT1	tuftelin 1	Multiple_Complex
TC0500013309.hg.1	6.61	6.13	1.4	0.0002	0.0011	ESM1	endothelial cell-specific molecule 1	Coding
TC0600012631.hg.1	7.27	8.05	-1.72	0.0002	0.0011	MMS22L	MMS22-like, DNA repair protein	Multiple_Complex
TC0200015332.hg.1	5.76	6.95	-2.28	0.0002	0.0011	PGAP1	post-GPI attachment to proteins 1	Multiple_Complex
TC0700009067.hg.1	9.95	11.08	-2.19	0.0002	0.0011	ATP6V1F	ATPase, H+ transporting, lysosomal 14kDa, V1	Multiple_Complex
TC0200009895.hg.1	8.82	9.64	-1.77	0.0002	0.0011	CERS6	ceramide synthase 6	Multiple_Complex
TC0600014263.hg.1	6.32	5.8	1.43	0.0002	0.0011	LY6G6E	lymphocyte antigen 6 complex, locus G6E (pse	Multiple_Complex
TC2000007300.hg.1	7.99	8.72	-1.66	0.0002	0.0011	RPRD1B	regulation of nuclear pre-mRNA domain conta	Multiple_Complex
TC0900007992.hg.1	11.25	11.99	-1.67	0.0002	0.0011	FAM120A	family with sequence similarity 120A	Multiple_Complex
TC2200008799.hg.1	8.78	9.83	-2.06	0.0002	0.0011	ST13	suppression of tumorigenicity 13 (colon carcir	Multiple_Complex
TC1200007820.hg.1	11.33	12.43	-2.13	0.0002	0.0011	RAB5B	RAB5B, member RAS oncogene family	Multiple_Complex
TC2200009218.hg.1	5.46	6.19	-1.65	0.0002	0.0011	MIR650; IG	microRNA 650; immunoglobulin lambda varial	Multiple_Complex
TC1300007731.hg.1	4.12	5.05	-1.9	0.0002	0.0011	GPR180	G protein-coupled receptor 180	Multiple_Complex
TC0300011016.hg.1	3.88	4.69	-1.75	0.0002	0.0011	NME6	NME/NM23 nucleoside diphosphate kinase 6	Multiple_Complex
TC0600012747.hg.1	9.83	8.01	3.53	0.0002	0.0011	CD24	CD24 molecule	Multiple_Complex
TC1100009245.hg.1	9.14	10.19	-2.07	0.0002	0.0011	CBL	Cbl proto-oncogene, E3 ubiquitin protein ligas	Multiple_Complex
TC0600014177.hg.1	9.01	8.03	1.97	0.0002	0.0011	DSE	dermatan sulfate epimerase	Multiple_Complex

TC1200007108.hg.1	6.18	5.3	1.84	0.0002	0.0011	KRAS	Memczak2013 ANTISENSE, coding, INTERNAL,	NonCoding
TC1200010655.hg.1	5.78	6.49	-1.64	0.0002	0.0011	LIMA1	LIM domain and actin binding 1	Multiple_Complex
TC2000009945.hg.1	5.4	4.73	1.58	0.0002	0.0011	FAM209A	family with sequence similarity 209, member	Coding
TC0100006983.hg.1	4.42	3.94	1.39	0.0002	0.0011	CTRC	chymotrypsin C (caldecrin)	Multiple_Complex
TC1900006522.hg.1	12.69	11.38	2.49	0.0002	0.0011	MIDN	midnolin	Multiple_Complex
TC0400012487.hg.1	5.64	4.9	1.67	0.0002	0.0011	VEGFC	vascular endothelial growth factor C	Multiple_Complex
TC0300009610.hg.1	8.2	9.19	-2	0.0002	0.0011	NDUFB5	NADH dehydrogenase (ubiquinone) 1 beta sul	Multiple_Complex
TC0600008674.hg.1	5.89	5.04	1.8	0.0002	0.0011	RIPPLY2	ripply transcriptional repressor 2	Multiple_Complex
TC1900012019.hg.1	7.12	7.99	-1.83	0.0002	0.0011	ZNF649	zinc finger protein 649	Multiple_Complex
TC0X00007195.hg.1	10.88	9.85	2.04	0.0002	0.0011	RBM3	RNA binding motif (RNP1, RRM) protein 3	Multiple_Complex
TC1100012981.hg.1	6.44	7.49	-2.06	0.0002	0.0011	RCN1	reticulocalbin 1, EF-hand calcium binding dom	Multiple_Complex
TC0200016568.hg.1	7.48	6.85	1.55	0.0002	0.0011	HOXD3	homeobox D3	NonCoding
TC0200016595.hg.1	13.39	14.73	-2.52	0.0002	0.0011	RPL37A	ribosomal protein L37a	Multiple_Complex
TC1600007186.hg.1	6.88	7.53	-1.57	0.0002	0.0011	C16orf52	chromosome 16 open reading frame 52	Multiple_Complex
TC0600008571.hg.1	4.14	4.87	-1.66	0.0002	0.0011	MYO6	Transcript Identified by AceView, Entrez Gene	Unassigned
TC1200010931.hg.1	11.67	12.49	-1.76	0.0002	0.0011	PTGES3	prostaglandin E synthase 3 (cytosolic)	Multiple_Complex
TC1600006886.hg.1	6.56	7.38	-1.76	0.0002	0.0011	NUBP1	nucleotide binding protein 1	Multiple_Complex
TC1900011957.hg.1	8.14	9.13	-1.99	0.0002	0.0011	GSK3A	glycogen synthase kinase 3 alpha	Multiple_Complex
TC1900009304.hg.1	4.85	4.05	1.73	0.0002	0.0011	PIP5K1C	Zhang2013 ALT_ACCEPTOR, ALT_DONOR, cod	NonCoding
TC1200007050.hg.1	4.9	4.1	1.74	0.0002	0.0011	IAPP	islet amyloid polypeptide	Coding
TC1200012745.hg.1	9.44	8.48	1.94	0.0002	0.0011	C1RL	complement component 1, r subcomponent-l	Multiple_Complex
TC1600008878.hg.1	4.36	3.72	1.56	0.0002	0.0011	LOC100287	uncharacterized LOC100287036; novel transcı	Multiple_Complex
TC1300008837.hg.1	9.49	10.51	-2.04	0.0002	0.0011	ZC3H13	zinc finger CCCH-type containing 13	Multiple_Complex
TC1300007843.hg.1	12.83	13.5	-1.59	0.0002	0.0011	TM9SF2	transmembrane 9 superfamily member 2	Multiple_Complex
TC1900008415.hg.1	7.31	6.42	1.86	0.0002	0.0011	CCDC9	coiled-coil domain containing 9	Multiple_Complex
TC0800006603.hg.1	5.21	4.35	1.81	0.0002	0.0011	DEFB105B;	defensin, beta 105B; defensin, beta 105A	Coding
TC1900009302.hg.1	7.61	8.32	-1.63	0.0002	0.0011	PIP5K1C	phosphatidylinositol-4-phosphate 5-kinase, ty	Multiple_Complex
TC0300014042.hg.1	8.99	8.17	1.77	0.0002	0.0011	RAB43	RAB43, member RAS oncogene family	Coding
TC1800008862.hg.1	6.2	7.09	-1.85	0.0002	0.0011	PIGN	phosphatidylinositol glycan anchor biosynthes	Multiple_Complex
TC2200008055.hg.1	7.95	9.28	-2.51	0.0002	0.0011	PI4KA	phosphatidylinositol 4-kinase, catalytic, alpha	Multiple_Complex
TC0700009253.hg.1	4.91	6.21	-2.47	0.0002	0.0011	NUP205	nucleoporin 205kDa	Multiple_Complex
TC0X00009667.hg.1	5.15	6.4	-2.39	0.0002	0.0011	FOXP3	forkhead box P3	Coding
TC1900009893.hg.1	9.52	8.15	2.59	0.0002	0.0011	CYP4F2	cytochrome P450, family 4, subfamily F, polyp	Multiple_Complex
TC0300009500.hg.1	14.59	15.49	-1.86	0.0002	0.0012	FNDC3B	fibronectin type III domain containing 3B	Multiple_Complex
TC0X00007026.hg.1	12.6	11.78	1.78	0.0002	0.0012	USP9X	ubiquitin specific peptidase 9, X-linked	Multiple_Complex
TC1200007626.hg.1	10.88	12.12	-2.36	0.0002	0.0012	METTL7A	methyltransferase like 7A	Coding
TC0X00009767.hg.1	7.82	8.79	-1.96	0.0002	0.0012	HUWE1	HECT, UBA and WWE domain containing 1, E3	Multiple_Complex
TC1100012886.hg.1	5.78	5.25	1.44	0.0002	0.0012	OPCML	opioid binding protein/cell adhesion molecule	NonCoding
TC1900011234.hg.1	4.9	4.28	1.54	0.0002	0.0012	KLK11	kallikrein related peptidase 11	Multiple_Complex
TC1900008249.hg.1	5.62	6.47	-1.81	0.0002	0.0012	ZNF230	zinc finger protein 230	Multiple_Complex
TC0800008122.hg.1	6.52	7.24	-1.65	0.0002	0.0012	E2F5	E2F transcription factor 5, p130-binding	Multiple_Complex
TC0200010216.hg.1	5.68	6.33	-1.57	0.0002	0.0012	ITGAV	integrin alpha V	Multiple_Complex
TC0900011665.hg.1	4.87	5.32	-1.36	0.0002	0.0012	DOLK	dolichol kinase	Coding
TC0200008291.hg.1	8.52	7.63	1.86	0.0002	0.0012	KDM3A	lysine (K)-specific demethylase 3A	Multiple_Complex
TC1300010036.hg.1	4.3	3.68	1.54	0.0002	0.0012	LINC00598	long intergenic non-protein coding RNA 598	NonCoding
TC1000008363.hg.1	17.35	16.02	2.5	0.0002	0.0012	PTEN	phosphatase and tensin homolog	Multiple_Complex
TC0100013748.hg.1	6.85	5.68	2.26	0.0002	0.0012	GNL2	guanine nucleotide binding protein-like 2 (nuc	Multiple_Complex
TC2100007341.hg.1	4.9	5.73	-1.79	0.0002	0.0012	PWP2	PWP2 periodic tryptophan protein homolog (y	Multiple_Complex
TC0200012339.hg.1	8.39	7.32	2.11	0.0002	0.0012	SLC8A1	solute carrier family 8 (sodium/calcium excha	Multiple_Complex
TC0200011022.hg.1	8.28	9.07	-1.73	0.0002	0.0012	SP140L	SP140 nuclear body protein-like	Multiple_Complex
TC1900008745.hg.1	5.7	6.34	-1.56	0.0002	0.0012	ZNF525	zinc finger protein 525	Multiple_Complex
TC0600007684.hg.1	6.76	6.07	1.61	0.0002	0.0012	SLC39A7	solute carrier family 39 (zinc transporter), mei	Multiple_Complex
TC0100007344.hg.1	11.14	10.18	1.94	0.0002	0.0012	PNRC2	proline-rich nuclear receptor coactivator 2	Multiple_Complex
TC2000007486.hg.1	7.24	6.75	1.41	0.0002	0.0012	RBPJL	recombination signal binding protein for immı	Multiple_Complex
TC1200007789.hg.1	4.12	3.39	1.67	0.0002	0.0012	OR6C75	olfactory receptor, family 6, subfamily C, mem	Coding
TC0200012995.hg.1	4.59	5.18	-1.5	0.0002	0.0012	FAM136A	family with sequence similarity 136, member	Multiple_Complex
TC1000011469.hg.1	4.51	5.51	-1.99	0.0002	0.0012	NOC3L	NOC3-like DNA replication regulator	Multiple_Complex
TC1200010105.hg.1	7.51	8.48	-1.97	0.0002	0.0012	RECQL	RecQ helicase-like	Multiple_Complex
TC1300008487.hg.1	6.22	7.1	-1.83	0.0002	0.0012	SLC7A1	solute carrier family 7 (cationic amino acid tra	Multiple_Complex
TC2200008226.hg.1	9.87	10.84	-1.96	0.0002	0.0012	GUCD1	guanylyl cyclase domain containing 1	Multiple_Complex
TC0100008025.hg.1	13.74	14.76	-2.02	0.0002	0.0012	YBX1	Y box binding protein 1	Multiple_Complex
TC1700008263.hg.1	5.82	4.66	2.24	0.0002	0.0012	ABCC3	ATP binding cassette subfamily C member 3	Multiple_Complex
TC0700012731.hg.1	4.25	3.87	1.29	0.0002	0.0012	DGKI	diacylglycerol kinase, iota	Multiple_Complex
TC1100012495.hg.1	13.03	12.4	1.54	0.0002	0.0012	DDX6	DEAD (Asp-Glu-Ala-Asp) box helicase 6	Multiple_Complex
TC1900008287.hg.1	6.65	4.84	3.5	0.0002	0.0012	PVRL2	poliovirus receptor-related 2 (herpesvirus entı	Multiple_Complex
TC1600008193.hg.1	6.73	7.44	-1.63	0.0002	0.0012	PRMT7	protein arginine methyltransferase 7	Multiple_Complex
TC0900011146.hg.1	5.67	6.79	-2.17	0.0002	0.0012	IKBKAP	inhibitor of kappa light polypeptide gene enha	Multiple_Complex
TC0700010728.hg.1	13.46	14.14	-1.61	0.0002	0.0012	HERPUD2	HERPUD family member 2	Multiple_Complex
TC1700007425.hg.1	3.83	4.53	-1.62	0.0002	0.0012	TP53I13	tumor protein p53 inducible protein 13	Multiple_Complex
TC0900008836.hg.1	5.73	6.74	-2	0.0002	0.0012	FAM102A	Memczak2013 ANTISENSE, CDS, coding, INTEI	NonCoding

TC0300011036.hg.1	7.48	6.69	1.73	0.0002	0.0012	PRKAR2A	protein kinase, cAMP-dependent, regulatory,	Multiple_Complex
TC1700007772.hg.1	7.76	8.55	-1.73	0.0002	0.0012	PSMD3	proteasome 26S subunit, non-ATPase 3	Multiple_Complex
TC0400012057.hg.1	6.69	7.55	-1.82	0.0002	0.0012	SLC10A7	solute carrier family 10, member 7	Multiple_Complex
TC1700012256.hg.1	5.28	4.65	1.55	0.0003	0.0013	KRTAP9-9	keratin associated protein 9-9	Coding
TC2200008434.hg.1	10.75	11.67	-1.89	0.0003	0.0013	SF3A1	splicing factor 3a, subunit 1, 120kDa	Multiple_Complex
TC0300007004.hg.1	11.28	11.87	-1.5	0.0003	0.0013	PDCD6IP	programmed cell death 6 interacting protein	Multiple_Complex
TC0100013796.hg.1	6.89	7.78	-1.85	0.0003	0.0013	PABPC4	poly(A) binding protein, cytoplasmic 4 (inducil	Multiple_Complex
TC0700009256.hg.1	9.81	10.76	-1.93	0.0003	0.0013	C7orf73	chromosome 7 open reading frame 73	Multiple_Complex
TC0200015087.hg.1	7.07	6.34	1.66	0.0003	0.0013	SESTD1	SEC14 and spectrin domains 1	Multiple_Complex
TC0100018045.hg.1	5.86	6.64	-1.71	0.0003	0.0013	SMYD3	SET and MYND domain containing 3	Multiple_Complex
TC0600014322.hg.1	9.46	10.45	-1.99	0.0003	0.0013	SYNCRIP	synaptotagmin binding, cytoplasmic RNA inte	Multiple_Complex
TC1000011988.hg.1	9.81	10.97	-2.23	0.0003	0.0013	EIF3A	eukaryotic translation initiation factor 3, subu	Multiple_Complex
TC0500013174.hg.1	11.7	11.1	1.51	0.0003	0.0013	KIF2A	kinesin heavy chain member 2A	Multiple_Complex
TC0700013371.hg.1	7.07	7.91	-1.8	0.0003	0.0013	ZNF138	zinc finger protein 138	Multiple_Complex
TC1900010761.hg.1	9.28	7.92	2.57	0.0003	0.0013	CEACAM4	carcinoembryonic antigen-related cell adhesic	Multiple_Complex
TC1600009182.hg.1	5.28	6.17	-1.86	0.0003	0.0013	ZNF200	zinc finger protein 200	Multiple_Complex
TC1300009725.hg.1	6.44	5.77	1.59	0.0003	0.0013	FAM155A	family with sequence similarity 155, member	Coding
TC0200016509.hg.1	5.03	4.44	1.5	0.0003	0.0013	NT5DC4	5-nucleotidase domain containing 4	NonCoding
TC0600013186.hg.1	5.06	5.7	-1.56	0.0003	0.0013	CTGF	connective tissue growth factor	Multiple_Complex
TC1900007313.hg.1	8.39	8.99	-1.52	0.0003	0.0013	USE1	unconventional SNARE in the ER 1 homolog (S	Multiple_Complex
TC0400008329.hg.1	5.88	6.69	-1.76	0.0003	0.0013	GSTCD	glutathione S-transferase, C-terminal domain	Multiple_Complex
TC0900008628.hg.1	7.28	8.2	-1.89	0.0003	0.0013	CNTRL	centriolin	Multiple_Complex
TC0300013978.hg.1	10.4	9.68	1.65	0.0003	0.0013	USP4	ubiquitin specific peptidase 4 (proto-oncogen	Multiple_Complex
TC0300012189.hg.1	9.2	8.2	2	0.0003	0.0013	KPNA1	karyopherin alpha 1	Multiple_Complex
TC2100007663.hg.1	6.97	6.07	1.86	0.0003	0.0013	BTG3	BTG family, member 3	Multiple_Complex
TC1200006957.hg.1	6.18	7.14	-1.94	0.0003	0.0013	PTPRO	protein tyrosine phosphatase, receptor type, (	Multiple_Complex
TC0300008989.hg.1	7.38	6.54	1.79	0.0003	0.0013	SLC25A36	solute carrier family 25 (pyrimidine nucleotide	Multiple_Complex
TC0400010077.hg.1	6.4	7.01	-1.53	0.0003	0.0013	RAB28	RAB28, member RAS oncogene family	Multiple_Complex
TC1100010818.hg.1	4.55	4.07	1.39	0.0003	0.0013	OR4C46	olfactory receptor, family 4, subfamily C, mem	Coding
TC0200006977.hg.1	4.35	5.05	-1.62	0.0003	0.0014	CENPO	centromere protein O	Multiple_Complex
TC0300011175.hg.1	8.02	8.55	-1.45	0.0003	0.0014	PBRM1	polybromo 1	Multiple_Complex
TC0900011510.hg.1	12.17	9.58	6.01	0.0003	0.0014	RPL35	ribosomal protein L35	Multiple_Complex
TC0200011257.hg.1	5.22	5.66	-1.36	0.0003	0.0014	PRLH	prolactin releasing hormone	Coding
TSUnmapped00000125.h	9.16	8.55	1.52	0.0003	0.0014	KIAA1143	KIAA1143 [Source:HGNC Symbol;Acc:HGNC:2	NonCoding
TC0300007033.hg.1	7.07	7.62	-1.47	0.0003	0.0014	MLH1	mutL homolog 1	Multiple_Complex
TC1100010948.hg.1	5.04	4.57	1.39	0.0003	0.0014	GLYAT	glycine-N-acyltransferase	Multiple_Complex
TC1200012629.hg.1	17.94	18.63	-1.62	0.0003	0.0014	DAZAP2	DAZ associated protein 2	Multiple_Complex
TC0500007452.hg.1	11.47	12.28	-1.75	0.0003	0.0014	MAP3K1	mitogen-activated protein kinase kinase kinas	Multiple_Complex
TC1400009422.hg.1	5.14	5.98	-1.78	0.0003	0.0014	RAB15	RAB15, member RAS oncogene family	Multiple_Complex
TC2000008894.hg.1	4.33	3.77	1.48	0.0003	0.0014	E2F1	E2F transcription factor 1	Multiple_Complex
TC1900007848.hg.1	4.9	4.18	1.64	0.0003	0.0014	HAMP	hepcidin antimicrobial peptide	Multiple_Complex
TC2000006571.hg.1	9.1	7.68	2.68	0.0003	0.0014	SMOX	spermine oxidase	Multiple_Complex
TC2200007547.hg.1	7.62	6.71	1.88	0.0003	0.0014	TSPO	translocator protein (18kDa)	Multiple_Complex
TC0200015401.hg.1	7.73	8.41	-1.6	0.0003	0.0014	ORC2	origin recognition complex subunit 2	Multiple_Complex
TC0700011156.hg.1	4.69	3.99	1.63	0.0003	0.0014	14-Sep	septin 14	Multiple_Complex
TC1100008523.hg.1	5.18	5.7	-1.43	0.0003	0.0014	CAPN5	calpain 5	Multiple_Complex
TC1100009287.hg.1	8.75	7.58	2.25	0.0003	0.0014	ARHGEF12	Rho guanine nucleotide exchange factor (GEF)	Multiple_Complex
TC1500010706.hg.1	5.45	4.67	1.72	0.0003	0.0014	ARHGAP11	Rho GTPase activating protein 11B	NonCoding
TC0100009402.hg.1	8.47	9.35	-1.83	0.0003	0.0014	CEPT1	choline/ethanolamine phosphotransferase 1	Multiple_Complex
TC1700010831.hg.1	9.35	10.36	-2.01	0.0003	0.0014	GPATCH8	G-patch domain containing 8	Multiple_Complex
TC1900009757.hg.1	8.59	9.41	-1.76	0.0003	0.0014	FARSA	phenylalanyl-tRNA synthetase alpha subunit	Multiple_Complex
TC0900011970.hg.1	4.75	5.51	-1.69	0.0003	0.0014	QSOX2	quiescin Q6 sulfhydryl oxidase 2	Multiple_Complex
TC1400006537.hg.1	8.65	7.65	2.01	0.0003	0.0014	RNASE3	ribonuclease, RNase A family, 3	Coding
TC1700008602.hg.1	7.55	6.46	2.14	0.0003	0.0014	DDX5	Memczak2013 ANTISENSE, CDS, coding, INTEF	NonCoding
TC1200009931.hg.1	4.33	5.4	-2.11	0.0003	0.0014	TAS2R46	taste receptor, type 2, member 46	Coding
TC0X00011274.hg.1	6.03	6.77	-1.67	0.0003	0.0014	TCEANC	transcription elongation factor A (SII) N-termi	Multiple_Complex
TC1900012024.hg.1	5	5.68	-1.61	0.0003	0.0014	ZNF600	zinc finger protein 600	Multiple_Complex
TC0500011146.hg.1	5.76	4.85	1.89	0.0003	0.0014	ENC1	ectodermal-neural cortex 1 (with BTB domain	Multiple_Complex
TC1200008606.hg.1	6.12	6.87	-1.69	0.0003	0.0014	UTP20	UTP20 small subunit (SSU) processome comp	Multiple_Complex
TC0900012066.hg.1	5.22	6.18	-1.95	0.0003	0.0014	NSMF; MIR	NMDA receptor synaptonuclear signaling and	Multiple_Complex
TC0900006438.hg.1	13.38	14.1	-1.65	0.0003	0.0014	DOCK8	dedicator of cytokinesis 8	Multiple_Complex
TC1200010976.hg.1	8.04	8.82	-1.71	0.0003	0.0014	AGAP2	ArfGAP with GTPase domain, ankyrin repeat a	Multiple_Complex
TC0500007303.hg.1	7.84	8.55	-1.63	0.0003	0.0014	NNT	nicotinamide nucleotide transhydrogenase	Multiple_Complex
TC1100013002.hg.1	5.9	6.61	-1.63	0.0003	0.0014	TMX2-CTNI	TMX2-CTNND1 readthrough (NMD candidate)	Multiple_Complex
TC1600010276.hg.1	6.89	7.92	-2.05	0.0003	0.0014	SNX20	sorting nexin 20	Multiple_Complex
TC0600008057.hg.1	4.9	4.28	1.54	0.0003	0.0014	PTCRA	pre T-cell antigen receptor alpha	Coding
TC1300008137.hg.1	8.4	7.07	2.51	0.0003	0.0014	TMCO3	transmembrane and coiled-coil domains 3	Multiple_Complex
TC0700010887.hg.1	9.75	10.39	-1.55	0.0003	0.0014	COA1	cytochrome c oxidase assembly factor 1 homo	Multiple_Complex
TC0300011059.hg.1	10.84	9.48	2.57	0.0003	0.0014	GPX1	glutathione peroxidase 1	Multiple_Complex
TC0100014505.hg.1	7.08	7.93	-1.81	0.0003	0.0014	SLC35D1	solute carrier family 35 (UDP-GlcA/UDP-GalN/	Multiple_Complex

TC1900011880.hg.1	8.24	7.56	1.6	0.0003	0.0014	ZNF44	zinc finger protein 44	Multiple_Complex
TC0400006930.hg.1	8.95	7.9	2.08	0.0003	0.0015	CPEB2	cytoplasmic polyadenylation element binding	Multiple_Complex
TC1100009248.hg.1	4.87	5.56	-1.61	0.0003	0.0015	RNF26	ring finger protein 26	Coding
TC1900006904.hg.1	8.94	7.98	1.96	0.0003	0.0015	MYO1F	Memczak2013 ANTISENSE, CDS, coding, INTEF	NonCoding
TC0X00008724.hg.1	5.31	4.65	1.59	0.0003	0.0015	HMGB3	high mobility group box 3	Multiple_Complex
TC0700006652.hg.1	8.43	7.63	1.73	0.0003	0.0015	ZNF316	zinc finger protein 316	Multiple_Complex
TC0400012798.hg.1	4.12	3.57	1.46	0.0003	0.0015	ADGRL3	adhesion G protein-coupled receptor L3	Multiple_Complex
TC1000008388.hg.1	10.26	9.21	2.06	0.0003	0.0015	FAS	Fas cell surface death receptor	Multiple_Complex
TC1700010198.hg.1	11.81	11.16	1.57	0.0003	0.0015	UNC119	unc-119 lipid binding chaperone	Multiple_Complex
TC1600006594.hg.1	5.75	6.27	-1.44	0.0003	0.0015	TRAF7	TNF receptor-associated factor 7, E3 ubiquitin	Multiple_Complex
TC1300008359.hg.1	10.04	11.01	-1.96	0.0003	0.0015	PARP4	poly(ADP-ribose) polymerase family member	Multiple_Complex
TC0900011148.hg.1	6.12	5.46	1.58	0.0003	0.0015	CTNNAL1	catenin (cadherin-associated protein), alpha-li	Multiple_Complex
TC1700012187.hg.1	8.06	9.39	-2.52	0.0003	0.0015	TNFSF12-TI	TNFSF12-TNFSF13 readthrough	Coding
TC1900011328.hg.1	17.47	16.79	1.6	0.0003	0.0015	ZNF160	Transcript Identified by AceView, Entrez Gene	Coding
TC0400007225.hg.1	5.39	6.9	-2.87	0.0003	0.0015	DTHD1	death domain containing 1	Multiple_Complex
TC1700006748.hg.1	5.22	5.82	-1.51	0.0003	0.0015	TMEM102	transmembrane protein 102	Coding
TC1900012022.hg.1	6.05	6.86	-1.75	0.0003	0.0015	ZNF616	zinc finger protein 616	Multiple_Complex
TC0400008030.hg.1	5.98	5.36	1.54	0.0003	0.0015	ARHGAP24	Rho GTPase activating protein 24	Multiple_Complex
TC1400010022.hg.1	8	8.79	-1.73	0.0003	0.0015	BTBD7	BTB (POZ) domain containing 7	Multiple_Complex
TC0400012956.hg.1	4.88	5.73	-1.8	0.0003	0.0015	ELOVL6	ELOVL fatty acid elongase 6	Multiple_Complex
TC1100006925.hg.1	7.66	8.44	-1.72	0.0003	0.0015	PDE3B	phosphodiesterase 3B, cGMP-inhibited	NonCoding
TC1100006656.hg.1	6.82	8.03	-2.31	0.0003	0.0015	OR52K2	olfactory receptor, family 52, subfamily K, me	Coding
TC0200016548.hg.1	7.37	8.19	-1.76	0.0003	0.0015	GPD2	glycerol-3-phosphate dehydrogenase 2	Multiple_Complex
TC0100010815.hg.1	7.95	8.67	-1.65	0.0003	0.0015	MR1	major histocompatibility complex, class I-relat	Multiple_Complex
TC1900011029.hg.1	4.38	3.69	1.61	0.0003	0.0015	MEIS3	Meis homeobox 3	Multiple_Complex
TC0300013696.hg.1	4.87	4.26	1.52	0.0003	0.0015	ZDHHC19	zinc finger, DHHC-type containing 19	Multiple_Complex
TC1600007806.hg.1	7.75	8.17	-1.34	0.0003	0.0015	HEATR3	HEAT repeat containing 3	Multiple_Complex
TC1700010199.hg.1	8.02	8.59	-1.48	0.0003	0.0015	PIGS	phosphatidylinositol glycan anchor biosynthes	Multiple_Complex
TC1200010940.hg.1	4.54	4.14	1.32	0.0003	0.0015	SDR9C7	short chain dehydrogenase/reductase family 9	Coding
TC0100018182.hg.1	8.52	8.02	1.41	0.0003	0.0015	SZRD1	SUZ RNA binding domain containing 1	Multiple_Complex
TC0500010680.hg.1	8.52	9.74	-2.33	0.0003	0.0015	PARP8	Memczak2013 ANTISENSE, CDS, coding, INTEF	NonCoding
TC1100011103.hg.1	7.87	6.95	1.9	0.0003	0.0015	STX5	syntaxin 5	Multiple_Complex
TC0300010399.hg.1	10.13	9.43	1.62	0.0003	0.0015	SH3BP5	SH3-domain binding protein 5 (BTK-associatec	Multiple_Complex
TC0900007165.hg.1	6.64	7.32	-1.61	0.0003	0.0015	TRMT10B	tRNA methyltransferase 10B	Multiple_Complex
TC1300008329.hg.1	5.61	6.65	-2.06	0.0003	0.0015	SACS	sacsin molecular chaperone	Multiple_Complex
TC0100006627.hg.1	5.15	5.7	-1.46	0.0003	0.0015	DFFB	DNA fragmentation factor, 40kDa, beta polype	Multiple_Complex
TC0300012527.hg.1	12.34	12.92	-1.49	0.0003	0.0015	MSL2	male-specific lethal 2 homolog (Drosophila)	Coding
TC0200007486.hg.1	6.98	7.7	-1.64	0.0003	0.0015	TTC7A	tetratricopeptide repeat domain 7A	Multiple_Complex
TC1100012730.hg.1	5.7	6.47	-1.71	0.0003	0.0015	RPUSD4	RNA pseudouridylate synthase domain contai	Multiple_Complex
TC0500007792.hg.1	12.42	11.33	2.13	0.0003	0.0016	HEXB	hexosaminidase B (beta polypeptide)	Multiple_Complex
TC1700010518.hg.1	6.21	5.64	1.49	0.0003	0.0016	GPR179	G protein-coupled receptor 179	Multiple_Complex
TC1000010911.hg.1	6.21	6.82	-1.53	0.0003	0.0016	TYSND1	trypsin domain containing 1	Multiple_Complex
TC1600008963.hg.1	7.84	8.33	-1.41	0.0003	0.0016	C16orf13	chromosome 16 open reading frame 13	Multiple_Complex
TC2000007012.hg.1	5.89	6.71	-1.76	0.0003	0.0016	ENTPD6	ectonucleoside triphosphate diphosphohydrol	Multiple_Complex
TC0600008757.hg.1	18.48	17.06	2.66	0.0003	0.0016	PNRC1	proline-rich nuclear receptor coactivator 1	Multiple_Complex
TC0400007128.hg.1	7.08	7.91	-1.78	0.0003	0.0016	TBC1D19	TBC1 domain family, member 19	Multiple_Complex
TC0700009678.hg.1	6.9	7.83	-1.91	0.0003	0.0016	GIMAP2	GTPase, IMAP family member 2	Multiple_Complex
TC0Y00006632.hg.1	11.54	7.46	16.91	0.0003	0.0016	DDX3Y	DEAD (Asp-Glu-Ala-Asp) box helicase 3, Y-link	Multiple_Complex
TC1100008831.hg.1	7.16	6.49	1.6	0.0003	0.0016	ENDOD1	endonuclease domain containing 1	Multiple_Complex
TC0800012405.hg.1	6.69	7.6	-1.88	0.0003	0.0016	FUT10	fucosyltransferase 10 (alpha (1,3) fucosyltrans	Multiple_Complex
TC1900008013.hg.1	8.51	9.46	-1.93	0.0003	0.0016	PSMD8	proteasome 26S subunit, non-ATPase 8	Multiple_Complex
TC0300008518.hg.1	6.13	6.82	-1.61	0.0003	0.0016	GTF2E1	general transcription factor IIE subunit 1	Multiple_Complex
TC0200008128.hg.1	4.28	4.75	-1.38	0.0003	0.0016	MRPL19	mitochondrial ribosomal protein L19	Multiple_Complex
TC1000009244.hg.1	7.42	6.53	1.86	0.0003	0.0016	BCCIP	BRCA2 and CDKN1A interacting protein	Multiple_Complex
TC0100016658.hg.1	14.2	12.94	2.38	0.0003	0.0016	ARPC5	actin related protein 2/3 complex subunit 5	Multiple_Complex
TC0X00008857.hg.1	6.65	7.24	-1.5	0.0003	0.0016	DKC1; SNO	dyskeratosis congenita 1, dyskerin; small nucl	Multiple_Complex
TC0600014281.hg.1	9.52	8.59	1.9	0.0003	0.0016	RPS10-NUD	RPS10-NUDT3 readthrough	Multiple_Complex
TC1600009952.hg.1	15.08	16.15	-2.1	0.0003	0.0016	YPEL3	yippee like 3	Multiple_Complex
TC0700010965.hg.1	5.24	5.96	-1.64	0.0003	0.0016	IGFBP3	insulin like growth factor binding protein 3	Multiple_Complex
TC1500010856.hg.1	5.88	7.71	-3.55	0.0003	0.0016	EPB42	erythrocyte membrane protein band 4.2	NonCoding
TC1100009615.hg.1	9.82	10.49	-1.58	0.0003	0.0016	VPS26B	VPS26 retromer complex component B	Multiple_Complex
TC1400007310.hg.1	7.06	7.71	-1.56	0.0003	0.0016	KIAA0586	KIAA0586	Multiple_Complex
TC2000009949.hg.1	12.21	11.55	1.59	0.0003	0.0016	STX16	syntaxin 16	Multiple_Complex
TC1900010047.hg.1	5.34	5.7	-1.29	0.0003	0.0016	NR2C2AP	nuclear receptor 2C2-associated protein	Multiple_Complex
TC1700012296.hg.1	11.81	12.21	-1.32	0.0003	0.0016	PRKAR1A; /	protein kinase, cAMP-dependent, regulatory,	Multiple_Complex
TC1700006702.hg.1	7.07	6.31	1.7	0.0003	0.0016	TXNDC17	thioredoxin domain containing 17	Multiple_Complex
TC0900007517.hg.1	4.79	4.04	1.68	0.0003	0.0016	C9orf135	chromosome 9 open reading frame 135	Multiple_Complex
TC0800007767.hg.1	7.68	6.29	2.63	0.0003	0.0016	RAB2A	Memczak2013 ALT_ACCEPTOR, ALT_DONOR,	NonCoding
TC0200015548.hg.1	7.47	8.1	-1.55	0.0003	0.0016	METTL21A	methyltransferase like 21A	Multiple_Complex
TC0100009046.hg.1	13.13	10.39	6.71	0.0003	0.0016	RPL5; SNOI	ribosomal protein L5; small nucleolar RNA, C/I	Multiple_Complex

TC1700008256.hg.1	5.83	6.28	-1.37	0.0003	0.0016	RSAD1	radical S-adenosyl methionine domain contair	Multiple_Complex
TC0700006984.hg.1	11.49	10.23	2.41	0.0003	0.0016	SNX10	sorting nexin 10	Multiple_Complex
TC1900011731.hg.1	6.15	5.41	1.67	0.0003	0.0016	LGALS17A	Charcot-Leyden crystal protein pseudogene	Multiple_Complex
TC0900011241.hg.1	4.85	5.99	-2.19	0.0003	0.0016	SLC46A2	solute carrier family 46, member 2	Coding
TC0X00010155.hg.1	8.29	9.32	-2.04	0.0003	0.0017	ITM2A	integral membrane protein 2A	Multiple_Complex
TC0200006648.hg.1	8.08	8.8	-1.65	0.0003	0.0017	IAH1	isoamyl acetate-hydrolyzing esterase 1 homol	Multiple_Complex
TC0400009919.hg.1	7.82	8.48	-1.57	0.0003	0.0017	MRFAP1L1	Morf4 family associated protein 1-like 1	Multiple_Complex
TC1800007394.hg.1	3.8	3.3	1.41	0.0003	0.0017	ST8SIA3	ST8 alpha-N-acetyl-neuraminide alpha-2,8-sia	Multiple_Complex
TC0700011463.hg.1	6.61	7.01	-1.32	0.0003	0.0017	TYW1B	tRNA-yW synthesizing protein 1 homolog B (S.	Multiple_Complex
TC0400011744.hg.1	12.49	13.66	-2.25	0.0003	0.0017	ANXA5	annexin A5	Multiple_Complex
TC0700013595.hg.1	4.39	3.88	1.42	0.0003	0.0017	C7orf61	chromosome 7 open reading frame 61	Coding
TC1200010250.hg.1	8.57	7.23	2.53	0.0003	0.0017	ERGIC2	ERGIC and golgi 2	Multiple_Complex
TC1200006874.hg.1	5.68	6.49	-1.75	0.0003	0.0017	BORCS5	BLOC-1 related complex subunit 5	Multiple_Complex
TC0100013374.hg.1	5.77	5.14	1.55	0.0003	0.0017	TRIM63	tripartite motif containing 63, E3 ubiquitin prc	Multiple_Complex
TC0200016494.hg.1	6.01	6.64	-1.55	0.0003	0.0017	CNNM4	cyclin and CBS domain divalent metal cation tı	Multiple_Complex
TC0100014562.hg.1	6.89	7.4	-1.43	0.0004	0.0017	LRRC40	leucine rich repeat containing 40	Multiple_Complex
TC0400008376.hg.1	9.19	9.7	-1.42	0.0004	0.0017	SEC24B	SEC24 homolog B, COPII coat complex compoı	Multiple_Complex
TC0800007735.hg.1	11.91	11.09	1.76	0.0004	0.0017	UBXN2B	UBX domain protein 2B	Multiple_Complex
TC0800008641.hg.1	7.11	6.4	1.64	0.0004	0.0017	MED30	mediator complex subunit 30	Multiple_Complex
TC1900008755.hg.1	7.39	6.17	2.32	0.0004	0.0017	ZNF331	zinc finger protein 331	Multiple_Complex
TC1400010728.hg.1	5.08	5.86	-1.72	0.0004	0.0017	HEATR5A	HEAT repeat containing 5A	Multiple_Complex
TC1100010762.hg.1	7.49	8.18	-1.61	0.0004	0.0017	NUP160	nucleoporin 160kDa	Multiple_Complex
TC1700008498.hg.1	8.8	9.19	-1.31	0.0004	0.0017	PPM1D	protein phosphatase, Mg2+/Mn2+ dependent	Multiple_Complex
TC1000012570.hg.1	8.93	8.16	1.69	0.0004	0.0017	NDST2	N-deacetylase/N-sulfotransferase (heparan gl	Multiple_Complex
TC1200012702.hg.1	5.46	6.03	-1.49	0.0004	0.0017	FICD	FIC domain containing	Coding
TC0600014148.hg.1	12.29	11.41	1.84	0.0004	0.0017	CYB5R4	cytochrome b5 reductase 4	Multiple_Complex
TC0700013609.hg.1	7.58	8.83	-2.37	0.0004	0.0017	TFEC	transcription factor EC	Multiple_Complex
TC0200011965.hg.1	7.12	7.94	-1.76	0.0004	0.0017	ATAD2B	ATPase family, AAA domain containing 2B	Multiple_Complex
TC0200006476.hg.1	6.5	7.1	-1.53	0.0004	0.0017	SNTG2	syntrophin, gamma 2	Multiple_Complex
TC1600011341.hg.1	5.15	5.97	-1.77	0.0004	0.0017	ZNF174	zinc finger protein 174	Multiple_Complex
TC0100015061.hg.1	6.13	6.57	-1.36	0.0004	0.0017	DBT	dihydrolipoamide branched chain transacylase	Multiple_Complex
TC1900011637.hg.1	4.16	4.82	-1.58	0.0004	0.0017	MZF1	myeloid zinc finger 1	Multiple_Complex
TC1600008646.hg.1	5.37	4.87	1.42	0.0004	0.0018	ATP2C2	ATPase, Ca++ transporting, type 2C, member 2	Multiple_Complex
TC1100011083.hg.1	8.05	8.87	-1.76	0.0004	0.0018	AHNAK	AHNAK nucleoprotein	Multiple_Complex
TC0300007479.hg.1	5.07	4.4	1.59	0.0004	0.0018	PHF7	PHD finger protein 7	Multiple_Complex
TC0300013356.hg.1	5.24	4.85	1.31	0.0004	0.0018	MAP6D1	MAP6 domain containing 1	Multiple_Complex
TC0800008467.hg.1	8.81	7.87	1.91	0.0004	0.0018	ATP6V1C1	ATPase, H+ transporting, lysosomal 42kDa, V1	Multiple_Complex
TC1900008114.hg.1	8.27	7.71	1.47	0.0004	0.0018	LTBP4	Transcript Identified by AceView, Entrez Gene	Unassigned
TC0600007825.hg.1	13.38	11.41	3.91	0.0004	0.0018	MAPK14	mitogen-activated protein kinase 14	Multiple_Complex
TC0500007122.hg.1	8.35	7.71	1.56	0.0004	0.0018	BRIX1	BRX1, biogenesis of ribosomes	Multiple_Complex
TC1800008903.hg.1	6.76	7.38	-1.54	0.0004	0.0018	KDSR	3-ketodihydrosphingosine reductase	Multiple_Complex
TC1600011570.hg.1	7.6	6.86	1.67	0.0004	0.0018	CMC2	C-x(9)-C motif containing 2	Multiple_Complex
TC1700012141.hg.1	10.22	9.54	1.6	0.0004	0.0018	WDR45B	WD repeat domain 45B	Multiple_Complex
TC1100009004.hg.1	6.26	6.88	-1.54	0.0004	0.0018	FDX1	ferredoxin 1	Coding
TC1700008282.hg.1	7.01	6.23	1.72	0.0004	0.0018	NME1; NM	NME/NM23 nucleoside diphosphate kinase 1;	Multiple_Complex
TC1100013185.hg.1	9.03	7.79	2.36	0.0004	0.0018	FAU	Finkel-Biskis-Reilly murine sarcoma virus (FBR	Multiple_Complex
TC1200012875.hg.1	4.4	5.26	-1.81	0.0004	0.0018	GOLGA3	golgin A3	Multiple_Complex
TC0200008084.hg.1	6.24	6.82	-1.49	0.0004	0.0018	TTC31	tetratricopeptide repeat domain 31	Multiple_Complex
TC1700006612.hg.1	7.39	6.45	1.92	0.0004	0.0018	CYB5D2	Transcript Identified by AceView, Entrez Gene	Unassigned
TC0400006818.hg.1	5.01	4.47	1.45	0.0004	0.0018	USP17L28;	ubiquitin specific peptidase 17-like family mer	Coding
TC0300006793.hg.1	4.69	5.47	-1.71	0.0004	0.0018	KAT2B	Transcript Identified by AceView, Entrez Gene	Unassigned
TC1700009274.hg.1	5.58	6.33	-1.68	0.0004	0.0018	FN3KRP	fructosamine 3 kinase related protein	Multiple_Complex
TC0100009580.hg.1	5.05	5.75	-1.63	0.0004	0.0018	WDR3	WD repeat domain 3	Multiple_Complex
TC0X00011083.hg.1	6.52	5.8	1.64	0.0004	0.0018	TMEM185A	transmembrane protein 185A	Multiple_Complex
TC0100009900.hg.1	3.97	3.42	1.46	0.0004	0.0018	CIART	circadian associated repressor of transcriptior	Multiple_Complex
TC0400012938.hg.1	6.93	5.93	2	0.0004	0.0018	RASGEF1B	RasGEF domain family member 1B	Multiple_Complex
TC0500011490.hg.1	6.3	7.33	-2.03	0.0004	0.0018	TTC37	tetratricopeptide repeat domain 37	Multiple_Complex
TC1600009165.hg.1	5.65	6.79	-2.2	0.0004	0.0018	MMP25-AS	MMP25 antisense RNA 1	NonCoding
TC1900011770.hg.1	15.37	14.11	2.38	0.0004	0.0018	C5AR1	complement component 5a receptor 1	Multiple_Complex
TC0400011978.hg.1	5.93	6.97	-2.06	0.0004	0.0018	TBC1D9	TBC1 domain family, member 9 (with GRAM c	Multiple_Complex
TC2100007208.hg.1	12	15.91	-15.03	0.0004	0.0018	MX1	MX dynamin-like GTPase 1	Multiple_Complex
TC2000007817.hg.1	7.86	8.93	-2.1	0.0004	0.0018	FAM210B	family with sequence similarity 210, member	Multiple_Complex
TC0500008296.hg.1	7.76	8.22	-1.38	0.0004	0.0018	MAN2A1	mannosidase, alpha, class 2A, member 1	Multiple_Complex
TC1200007043.hg.1	5.2	4.32	1.84	0.0004	0.0018	SLCO1C1	solute carrier organic anion transporter family	Multiple_Complex
TC1100009309.hg.1	14.68	13.35	2.52	0.0004	0.0018	SORL1	sortilin-related receptor, L(DLR class) A repeat	Multiple_Complex
TC1300006520.hg.1	10.7	9.69	2.02	0.0004	0.0018	SAP18	Sin3A associated protein 18kDa	Multiple_Complex
TC1200009981.hg.1	7.08	8.28	-2.29	0.0004	0.0019	HEBP1	heme binding protein 1	Multiple_Complex
TC1900007744.hg.1	7.84	6.81	2.04	0.0004	0.0019	DPY19L3	dpy-19-like 3 (C. elegans)	Multiple_Complex
TC1100010945.hg.1	4.62	4.99	-1.29	0.0004	0.0019	OR5B21	olfactory receptor, family 5, subfamily B, mer	Coding
TC1600011545.hg.1	7.04	7.83	-1.73	0.0004	0.0019	PLLP	plasmolipin	NonCoding

TC1600007803.hg.1	10.31	9.3	2.02	0.0004	0.0019	CNEP1R1	CTD nuclear envelope phosphatase 1 regulato	Multiple_Complex
TC0100015783.hg.1	7.86	8.49	-1.55	0.0004	0.0019	RFX5	regulatory factor X, 5 (influences HLA class II e	Multiple_Complex
TC0X00008862.hg.1	7.62	6.45	2.25	0.0004	0.0019	FUNDC2	FUN14 domain containing 2	Multiple_Complex
TC0200011901.hg.1	12.02	12.8	-1.72	0.0004	0.0019	PUM2	pumilio RNA binding family member 2	Multiple_Complex
TC0800012308.hg.1	5.98	6.43	-1.37	0.0004	0.0019	ADHFE1; C8	alcohol dehydrogenase, iron containing 1; chr	Multiple_Complex
TC0100010806.hg.1	9.02	9.82	-1.74	0.0004	0.0019	XPR1	xenotropic and polytropic retrovirus receptor	Multiple_Complex
TC0900012178.hg.1	6.96	7.6	-1.56	0.0004	0.0019	GLE1	GLE1 RNA export mediator	Multiple_Complex
TC0100018353.hg.1	5.63	6.22	-1.5	0.0004	0.0019	SNAP47	synaptosome associated protein 47kDa	Multiple_Complex
TC1200012704.hg.1	7.74	8.25	-1.42	0.0004	0.0019	TCHP	trichoplein, keratin filament binding	Multiple_Complex
TC1000012162.hg.1	7.68	8.35	-1.59	0.0004	0.0019	UROS	uroporphyrinogen III synthase	Multiple_Complex
TC1300007969.hg.1	10.73	9.72	2.01	0.0004	0.0019	ABHD13	abhydrolase domain containing 13	Coding
TC0100018540.hg.1	6.16	6.77	-1.53	0.0004	0.0019	ACBD6	acyl-CoA binding domain containing 6	Multiple_Complex
TC2200009310.hg.1	8.53	7.96	1.48	0.0004	0.0019	GAB4	GRB2-associated binding protein family, mem	Multiple_Complex
TC1400006551.hg.1	8.57	7.38	2.28	0.0004	0.0019	ARHGEF40	Rho guanine nucleotide exchange factor (GEF)	Multiple_Complex
TC1500007860.hg.1	6.12	6.94	-1.77	0.0004	0.0019	MPI	mannose phosphate isomerase	Multiple_Complex
TC1900009299.hg.1	8.14	8.69	-1.46	0.0004	0.0019	CACTIN	cactin, spliceosome C complex subunit	Multiple_Complex
TC0600008539.hg.1	5.15	4.47	1.6	0.0004	0.0019	CD109	CD109 molecule	Multiple_Complex
TC1200012265.hg.1	8.37	7.19	2.26	0.0004	0.0019	CDK2AP1	cyclin-dependent kinase 2 associated protein	Multiple_Complex
TC1400008597.hg.1	4.89	4.35	1.45	0.0004	0.0019	RNASE12; F	ribonuclease, RNase A family, 12 (non-active);	Multiple_Complex
TC1100013205.hg.1	9.69	8.46	2.34	0.0004	0.0019	ARAP1	ArfGAP with RhoGAP domain, ankyrin repeat a	Multiple_Complex
TC0600013204.hg.1	8.53	7.06	2.78	0.0004	0.0019	VNN3	vanin 3	Multiple_Complex
TC0100007254.hg.1	7.05	6.47	1.5	0.0004	0.0019	LDLRAD2	low density lipoprotein receptor class A doma	Multiple_Complex
TC0200009487.hg.1	12.26	11.17	2.13	0.0004	0.0019	SPOPL	speckle-type POZ protein-like	Multiple_Complex
TC2000007670.hg.1	4.44	3.74	1.63	0.0004	0.0019	SNAI1	snail family zinc finger 1	Coding
TC0500011255.hg.1	4.43	5.04	-1.53	0.0004	0.0019	MTX3	metaxin 3	Multiple_Complex
TC0100015155.hg.1	9.09	8.48	1.53	0.0004	0.0019	VAV3	vav 3 guanine nucleotide exchange factor	Multiple_Complex
TC0400010558.hg.1	8.03	8.48	-1.36	0.0004	0.002	ATP8A1	ATPase, aminophospholipid transporter (APLT	Multiple_Complex
TC0400012989.hg.1	5.56	6.13	-1.49	0.0004	0.002	TMEM192	transmembrane protein 192	Multiple_Complex
TC0700012712.hg.1	14.29	13.6	1.61	0.0004	0.002	LUZP6; MT	leucine zipper protein 6; myotrophin	Multiple_Complex
TC0100013182.hg.1	6.42	7.2	-1.72	0.0004	0.002	CAMK2N1	calcium/calmodulin-dependent protein kinase	Multiple_Complex
TC0200008554.hg.1	8.33	7.46	1.83	0.0004	0.002	COX5B	cytochrome c oxidase subunit Vb	Multiple_Complex
TC1100011240.hg.1	6.13	5.58	1.46	0.0004	0.002	KCNK7	potassium channel, two pore domain subfami	Coding
TC1100010033.hg.1	4.2	5	-1.74	0.0004	0.002	TRIM66	tripartite motif containing 66	Multiple_Complex
TC0100013854.hg.1	6.73	7.44	-1.64	0.0004	0.002	SCMH1	sex comb on midleg homolog 1 (Drosophila)	Multiple_Complex
TC0300013143.hg.1	4.19	3.73	1.38	0.0004	0.002	GHSR	growth hormone secretagogue receptor	Coding
TC0700012760.hg.1	5.93	5.31	1.53	0.0004	0.002	ZC3HAV1L	zinc finger CCCH-type, antiviral 1-like	Multiple_Complex
TC0600007060.hg.1	12.21	10.81	2.63	0.0004	0.002	MYLIP; MIF	myosin regulatory light chain interacting prote	Multiple_Complex
TC1000011368.hg.1	7.59	6.14	2.72	0.0004	0.002	ANKRD22	ankyrin repeat domain 22	Multiple_Complex
TC1300007130.hg.1	5.29	4.66	1.55	0.0004	0.002	NUDT15	nudix hydrolase 15	Coding
TC1600010406.hg.1	6.99	6.31	1.6	0.0004	0.002	AMFR	autocrine motility factor receptor, E3 ubiquitin	Multiple_Complex
TC0100018406.hg.1	5.88	5.09	1.72	0.0004	0.002	HNRNPCL1	heterogeneous nuclear ribonucleoprotein C-lil	Coding
TC0100014790.hg.1	4.76	4.1	1.57	0.0004	0.002	COL24A1	collagen, type XXIV, alpha 1	Multiple_Complex
TC1200006909.hg.1	4.06	3.56	1.41	0.0004	0.002	EMP1	epithelial membrane protein 1	Multiple_Complex
TC0200015741.hg.1	5.68	6.31	-1.55	0.0004	0.002	RNF25	ring finger protein 25	Multiple_Complex
TC0800007110.hg.1	5.02	5.93	-1.88	0.0004	0.002	EPHX2	epoxide hydrolase 2, cytoplasmic	Multiple_Complex
TC0300013983.hg.1	6.08	6.66	-1.5	0.0004	0.002	NAT6	N-acetyltransferase 6 (GCN5-related)	Coding
TC0X00006590.hg.1	7.32	6.46	1.82	0.0004	0.002	WWC3	WWC family member 3	Multiple_Complex
TC0700011586.hg.1	7.64	8.59	-1.93	0.0004	0.002	GSAP	gamma-secretase activating protein	Multiple_Complex
TC0100009889.hg.1	7.68	8.84	-2.23	0.0004	0.002	VPS45	vacuolar protein sorting 45 homolog (S. cerevi	Multiple_Complex
TC1700010738.hg.1	8.84	9.45	-1.52	0.0004	0.002	EZH1	enhancer of zeste 1 polycomb repressive com	Multiple_Complex
TC0700008617.hg.1	6.56	5.8	1.7	0.0004	0.002	SH2B2	SH2B adaptor protein 2	Multiple_Complex
TC0100007659.hg.1	10.06	10.79	-1.66	0.0004	0.002	KHDRBS1	KH domain containing, RNA binding, signal tra	Multiple_Complex
TC0100010060.hg.1	15.89	12.51	10.4	0.0004	0.002	RPS27	ribosomal protein S27	Multiple_Complex
TC1600011472.hg.1	8.94	9.46	-1.43	0.0004	0.0021	NMRAL1	NmrA-like family domain containing 1	Multiple_Complex
TC0X00007266.hg.1	3.55	4.35	-1.75	0.0004	0.0021	GSPT2	G1 to S phase transition 2	Coding
TC0100017733.hg.1	6.17	5.09	2.11	0.0004	0.0021	TSNAX	Memczak2013 ANTISENSE, CDS, coding, INTEF	NonCoding
TC1700012228.hg.1	9.55	10.11	-1.48	0.0004	0.0021	LRRC37B	leucine rich repeat containing 37B	Multiple_Complex
TC0800009854.hg.1	6.8	6.03	1.71	0.0004	0.0021	BIN3-IT1	BIN3 intronic transcript 1	Multiple_Complex
TC0100014228.hg.1	5.31	6.05	-1.67	0.0004	0.0021	LRP8	LDL receptor related protein 8	Multiple_Complex
TC0800006760.hg.1	9.47	9.97	-1.42	0.0004	0.0021	FDFT1	farnesyl-diphosphate farnesyltransferase 1	Multiple_Complex
TC0100007188.hg.1	6.89	6.17	1.65	0.0004	0.0021	PLA2G5	phospholipase A2, group V	Multiple_Complex
TC0100013466.hg.1	7.21	7.81	-1.52	0.0004	0.0021	SPCS2	signal peptidase complex subunit 2	Multiple_Complex
TC1600009864.hg.1	4.02	3.55	1.38	0.0004	0.0021	IL27	interleukin 27	Coding
TC1600006941.hg.1	7.85	8.61	-1.69	0.0004	0.0021	SNX29	sorting nexin 29	Multiple_Complex
TC1200010205.hg.1	5.5	4.76	1.67	0.0004	0.0021	C12orf71	chromosome 12 open reading frame 71	Coding
TC0100008033.hg.1	6.14	6.88	-1.66	0.0004	0.0021	ERMAP	erythroblast membrane-associated protein (Sc	Multiple_Complex
TC0900007604.hg.1	14.6	13.49	2.15	0.0004	0.0021	OSTF1	osteoclast stimulating factor 1	Multiple_Complex
TC1100011643.hg.1	10.74	11.37	-1.55	0.0004	0.0021	ARRB1	arrestin, beta 1	Multiple_Complex
TC1000011975.hg.1	11.93	11.56	1.29	0.0004	0.0021	CACUL1	CDK2-associated, cullin domain 1	Multiple_Complex
TC0800010103.hg.1	9.17	9.74	-1.49	0.0004	0.0021	LSM12	Homo sapiens LSM12 homolog (S. cerevisiae),	Multiple_Complex

TC1900012058.hg.1	7.92	8.89	-1.96	0.0004	0.0021	ZNF417	zinc finger protein 417	Multiple_Complex
TC0400012759.hg.1	8.62	9.31	-1.62	0.0004	0.0021	FAM193A	family with sequence similarity 193, member .	Multiple_Complex
TC1900011228.hg.1	4.88	4.42	1.38	0.0004	0.0021	KLK5	kallikrein related peptidase 5	Multiple_Complex
TC2000006541.hg.1	5.08	5.76	-1.61	0.0004	0.0021	ITPA	inosine triphosphatase (nucleoside triphospha	Multiple_Complex
TC0800007016.hg.1	10.47	9.16	2.47	0.0005	0.0021	CHMP7	Transcript Identified by AceView, Entrez Gene	Unassigned
TC0700011488.hg.1	7.19	7.76	-1.49	0.0005	0.0021	NSUN5	NOP2/Sun domain family, member 5	Multiple_Complex
TC1700007913.hg.1	4.8	4.02	1.72	0.0005	0.0021	RAMP2	receptor (G protein-coupled) activity modifyin	Multiple_Complex
TC1900011872.hg.1	5.41	6.13	-1.64	0.0005	0.0021	ECSIT	ECSIT signalling integrator	Multiple_Complex
TSUnmapped00000582.h	7.86	8.65	-1.73	0.0005	0.0021	ZNF780A	zinc finger protein 780A	Coding
TC1100012646.hg.1	4.34	3.73	1.53	0.0005	0.0021	TMEM225	transmembrane protein 225	Coding
TC0100009738.hg.1	7.36	8.25	-1.86	0.0005	0.0021	GPR89A	G protein-coupled receptor 89A	Multiple_Complex
TC0700008662.hg.1	6.89	7.41	-1.43	0.0005	0.0021	ARMC10	armadillo repeat containing 10	Multiple_Complex
TSUnmapped00000722.h	5.82	5.2	1.54	0.0005	0.0021	TRAPPC4	trafficking protein particle complex 4	NonCoding
TC0600011808.hg.1	5.24	5.99	-1.69	0.0005	0.0022	MED20	mediator complex subunit 20	Multiple_Complex
TC0700009481.hg.1	4.2	3.83	1.29	0.0005	0.0022	PIP	prolactin-induced protein	Coding
TC0200008921.hg.1	6.39	6.88	-1.4	0.0005	0.0022	TTL	tubulin tyrosine ligase	Multiple_Complex
TC0100007693.hg.1	7.36	8.21	-1.8	0.0005	0.0022	S100PBP	S100P binding protein	Multiple_Complex
TC0X00008470.hg.1	7.97	8.46	-1.4	0.0005	0.0022	PHF6	PHD finger protein 6	Multiple_Complex
TC1300008997.hg.1	9.35	8	2.55	0.0005	0.0022	DHRS12	dehydrogenase/reductase (SDR family) memb	Multiple_Complex
TC1700012093.hg.1	4.91	5.57	-1.58	0.0005	0.0022	FASN	fatty acid synthase	Multiple_Complex
TC0300008467.hg.1	6.3	7.51	-2.33	0.0005	0.0022	ARHGAP31	Rho GTPase activating protein 31	Multiple_Complex
TC1900006895.hg.1	7.23	6.4	1.78	0.0005	0.0022	2-Mar	membrane associated ring finger 2	Multiple_Complex
TC2200006540.hg.1	4.78	7.99	-9.27	0.0005	0.0022	USP18	ubiquitin specific peptidase 18	Multiple_Complex
TC0300011109.hg.1	7.01	7.8	-1.73	0.0005	0.0022	TMEM115	transmembrane protein 115	Multiple_Complex
TC1200009734.hg.1	8.64	9.24	-1.52	0.0005	0.0022	VAMP1	vesicle associated membrane protein 1	Multiple_Complex
TC0X00007612.hg.1	5.65	5.92	-1.21	0.0005	0.0022	CHIC1	cysteine rich hydrophobic domain 1	Multiple_Complex
TC0200007058.hg.1	9.53	10.1	-1.49	0.0005	0.0022	ATRAID	all-trans retinoic acid-induced differentiation f	Multiple_Complex
TC2100006922.hg.1	6.16	6.9	-1.66	0.0005	0.0022	EVA1C	eva-1 homolog C (C. elegans)	Multiple_Complex
TC0600011457.hg.1	7.25	7.79	-1.46	0.0005	0.0022	LSM2	LSM2 homolog, U6 small nuclear RNA and mR	Multiple_Complex
TC1700012329.hg.1	5.98	6.6	-1.54	0.0005	0.0022	METTL16	methyltransferase like 16	Multiple_Complex
TC0400012143.hg.1	8.42	7.89	1.44	0.0005	0.0022	FBXW7	F-box and WD repeat domain containing 7, E3	Multiple_Complex
TC1700008303.hg.1	3.72	3.13	1.51	0.0005	0.0022	C17orf112	chromosome 17 open reading frame 112	Multiple_Complex
TC1200010281.hg.1	9.43	10.27	-1.8	0.0005	0.0022	FAM60A	family with sequence similarity 60, member A	Multiple_Complex
TC0600009683.hg.1	9.4	8.66	1.67	0.0005	0.0022	AIG1	androgen-induced 1	Multiple_Complex
TC0700007859.hg.1	5.1	5.93	-1.78	0.0005	0.0022	TYW1	tRNA-yW synthesizing protein 1 homolog (S. c	Multiple_Complex
TC0100008815.hg.1	6.9	14.02	-139.53	0.0005	0.0022	IFI44L	interferon-induced protein 44-like	Multiple_Complex
TC0300008086.hg.1	4.77	5.7	-1.9	0.0005	0.0022	ARL6	ADP-ribosylation factor like GTPase 6	Multiple_Complex
TC0900009437.hg.1	7.55	8.16	-1.52	0.0005	0.0022	AK3	adenylate kinase 3	Multiple_Complex
TC1900006835.hg.1	6.2	6.83	-1.55	0.0005	0.0022	ZNF557	zinc finger protein 557	Multiple_Complex
TC1100006812.hg.1	10.53	9.7	1.77	0.0005	0.0022	ZNF143	zinc finger protein 143	Multiple_Complex
TC1400007244.hg.1	6.92	6.07	1.8	0.0005	0.0022	KTN1	kinectin 1 (kinesin receptor)	Multiple_Complex
TC0100012946.hg.1	4.55	3.97	1.5	0.0005	0.0022	PRAMEF8	PRAME family member 8	Coding
TC0500013320.hg.1	5.25	6.33	-2.11	0.0005	0.0022	GTF2H2	general transcription factor IIH subunit 2	NonCoding
TC0600007508.hg.1	10.27	11.41	-2.19	0.0005	0.0022	PPP1R11	protein phosphatase 1, regulatory (inhibitor) s	Coding
TC1200012622.hg.1	6.13	7.37	-2.36	0.0005	0.0022	SLC48A1	solute carrier family 48 (heme transporter), m	Multiple_Complex
TC0500012568.hg.1	4.78	5.64	-1.82	0.0005	0.0022	GEMIN5	gem nuclear organelle associated protein 5	Multiple_Complex
TC1900010163.hg.1	7.96	8.67	-1.64	0.0005	0.0023	ZNF43	zinc finger protein 43	Multiple_Complex
TC0400008879.hg.1	9.43	10.37	-1.91	0.0005	0.0023	GAB1	GRB2-associated binding protein 1	Multiple_Complex
TC0200014899.hg.1	6.81	7.62	-1.75	0.0005	0.0023	METTL8	methyltransferase like 8	Multiple_Complex
TC1900011851.hg.1	7.99	8.69	-1.62	0.0005	0.0023	NDUFA11	NADH dehydrogenase (ubiquinone) 1 alpha su	Multiple_Complex
TC1400009709.hg.1	6.35	6.94	-1.51	0.0005	0.0023	MLH3	mutL homolog 3	Multiple_Complex
TC0500009649.hg.1	6.19	6.71	-1.43	0.0005	0.0023	RMND5B	required for meiotic nuclear division 5 homolo	Multiple_Complex
TC0100009611.hg.1	5.02	4.53	1.41	0.0005	0.0023	HSD3B2	hydroxy-delta-5-steroid dehydrogenase, 3 bet	Multiple_Complex
TC0700013059.hg.1	6.46	7.3	-1.79	0.0005	0.0023	FASTK	Fas-activated serine/threonine kinase	Multiple_Complex
TC0300011023.hg.1	16.06	17.5	-2.71	0.0005	0.0023	SHISA5	shisa family member 5	Multiple_Complex
TC1700006741.hg.1	5.23	5.83	-1.51	0.0005	0.0023	KCTD11	potassium channel tetramerization domain co	Multiple_Complex
TC0700009346.hg.1	4.8	4.43	1.29	0.0005	0.0023	CLEC2L	C-type lectin domain family 2, member L	Multiple_Complex
TC1900009765.hg.1	6.08	6.95	-1.83	0.0005	0.0023	LYL1	lymphoblastic leukemia associated hematopo	Multiple_Complex
TC1600007345.hg.1	9.49	8.61	1.83	0.0005	0.0023	APOBR	apolipoprotein B receptor	Coding
TC1800009233.hg.1	6.31	7.39	-2.11	0.0005	0.0023	ATP8B1	Salzman2013 ANTISENSE, CDS, coding, INTERN	NonCoding
TC1100010713.hg.1	5.7	6.38	-1.59	0.0005	0.0023	CKAP5; SN(	cytoskeleton associated protein 5; small nucle	Multiple_Complex
TC0200012091.hg.1	4.45	5.34	-1.85	0.0005	0.0023	ZNF512	Memczak2013 ANTISENSE, coding, INTERNAL,	NonCoding
TC0X00008866.hg.1	7.57	6.53	2.06	0.0005	0.0023	VBP1	von Hippel-Lindau binding protein 1	Multiple_Complex
TC2000009968.hg.1	6.93	8.61	-3.19	0.0005	0.0023	SIRPB2	signal-regulatory protein beta 2	Multiple_Complex
TSUnmapped00000252.h	5.77	4.81	1.94	0.0005	0.0023	TRAPPC4	trafficking protein particle complex 4	NonCoding
TC1400010729.hg.1	6.72	7.29	-1.48	0.0005	0.0023	DTD2	D-tyrosyl-tRNA deacylase 2 (putative)	Multiple_Complex
TC1800008123.hg.1	4.33	4.95	-1.54	0.0005	0.0023	CEP76	centrosomal protein 76kDa	Multiple_Complex
TC1900010209.hg.1	7.42	7.9	-1.4	0.0005	0.0023	ZNF724P	zinc finger protein 724, pseudogene	Multiple_Complex
TC1900006574.hg.1	6.25	5.67	1.5	0.0005	0.0023	IZUMO4	IZUMO family member 4	Multiple_Complex
TC0100009572.hg.1	10.16	11.93	-3.42	0.0005	0.0023	FAM46C	family with sequence similarity 46, member C	Coding

TC1600009088.hg.1	6.03	6.53	-1.41	0.0005	0.0023	PKD1; MIR(	polycystic kidney disease 1 (autosomal domin	Multiple_Complex
TC0800010696.hg.1	4.37	3.81	1.48	0.0005	0.0023	TCF24	transcription factor 24	Multiple_Complex
TC0500011712.hg.1	5.39	6.43	-2.05	0.0005	0.0023	EPB41L4A	erythrocyte membrane protein band 4.1 like 4	Multiple_Complex
TC0100017374.hg.1	8.67	9.11	-1.36	0.0005	0.0023	RAB3GAP2,	RAB3 GTPase activating protein subunit 2 (nor	Multiple_Complex
TC1600011565.hg.1	5.8	5.24	1.48	0.0005	0.0023	CFDP1	craniofacial development protein 1	Multiple_Complex
TC1900011814.hg.1	5.62	6.4	-1.72	0.0005	0.0023	ZNF17	zinc finger protein 17	Multiple_Complex
TC1200011567.hg.1	10.25	9.26	1.98	0.0005	0.0023	TMCC3; MI	transmembrane and coiled-coil domain family	Multiple_Complex
TC0200008468.hg.1	4.74	5.61	-1.83	0.0005	0.0023	FAHD2A	fumarylacetoacetate hydrolase domain contai	Multiple_Complex
TC1400010156.hg.1	4.96	5.37	-1.33	0.0005	0.0023	BCL11B	B-cell CLL/lymphoma 11B (zinc finger protein)	Multiple_Complex
TC1200007866.hg.1	9.16	9.79	-1.55	0.0005	0.0023	SHMT2	serine hydroxymethyltransferase 2 (mitochon	Multiple_Complex
TC0700011973.hg.1	7.01	7.65	-1.56	0.0005	0.0023	TAF6	TAF6 RNA polymerase II, TATA box binding pro	Multiple_Complex
TC2200008641.hg.1	13.84	15.32	-2.79	0.0005	0.0023	RAC2	ras-related C3 botulinum toxin substrate 2 (rh	Multiple_Complex
TC0300012900.hg.1	7.62	6.97	1.57	0.0005	0.0024	TIPARP	Memczak2013 ANTISENSE, CDS, coding, INTER	NonCoding
TC1000010651.hg.1	5.32	6.07	-1.68	0.0005	0.0024	CSTF2T	cleavage stimulation factor, 3 pre-RNA, subun	Coding
TC0800011172.hg.1	5.78	6.73	-1.93	0.0005	0.0024	MTERF3	mitochondrial transcription termination factor	Multiple_Complex
TC1100007363.hg.1	6.88	7.57	-1.61	0.0005	0.0024	API5	apoptosis inhibitor 5	Multiple_Complex
TC1900011822.hg.1	6.85	6.47	1.3	0.0005	0.0024	ZNF586	zinc finger protein 586	Multiple_Complex
TC0700012609.hg.1	4.65	4.07	1.5	0.0005	0.0024	KLF14	Kruppel-like factor 14	Coding
TC1600011438.hg.1	6.26	6.99	-1.66	0.0005	0.0024	MLYCD	malonyl-CoA decarboxylase	Multiple_Complex
TC1100010976.hg.1	8.09	7.5	1.5	0.0005	0.0024	OSBP	oxysterol binding protein	Multiple_Complex
TC2200008134.hg.1	5.06	5.84	-1.72	0.0005	0.0024	ZNF280B	zinc finger protein 280B	Multiple_Complex
TC2000007343.hg.1	5.43	6.23	-1.75	0.0005	0.0024	DHX35	DEAH (Asp-Glu-Ala-His) box polypeptide 35	Multiple_Complex
TC0X00010670.hg.1	4.57	5.93	-2.56	0.0005	0.0024	TMEM255A	transmembrane protein 255A	Multiple_Complex
TC0800011669.hg.1	9.97	10.59	-1.54	0.0005	0.0024	DERL1	derlin 1	Multiple_Complex
TC0200011745.hg.1	9.27	8.35	1.89	0.0005	0.0024	ROCK2	Rho-associated, coiled-coil containing protein	Multiple_Complex
TC0500010081.hg.1	4.7	5.64	-1.92	0.0005	0.0024	FAM173B	Transcript Identified by AceView, Entrez Gene	Unassigned
TC0300012773.hg.1	4.73	4.25	1.39	0.0005	0.0024	TM4SF1	transmembrane 4 L six family member 1	Multiple_Complex
TC0400007120.hg.1	10.28	9.21	2.11	0.0005	0.0024	RBPJ	recombination signal binding protein for immu	Multiple_Complex
TC1200012599.hg.1	9.41	10.21	-1.74	0.0005	0.0024	AEBP2	AE binding protein 2	Multiple_Complex
TC0X00008235.hg.1	6.51	5.82	1.61	0.0005	0.0024	LONRF3	LON peptidase N-terminal domain and ring fin	Multiple_Complex
TC0700009079.hg.1	7.73	6.84	1.86	0.0005	0.0024	TSPAN33	tetraspanin 33	Multiple_Complex
TC1900009841.hg.1	4.84	4.05	1.73	0.0005	0.0024	OR7A5	olfactory receptor, family 7, subfamily A, mem	Multiple_Complex
TC0600011438.hg.1	10.54	11.34	-1.75	0.0005	0.0024	LTB	lymphotoxin beta (TNF superfamily, member 3	Multiple_Complex
TC1900010961.hg.1	4.84	5.44	-1.52	0.0005	0.0024	IRF2BP1	interferon regulatory factor 2 binding protein	Coding
TC1600010174.hg.1	5.96	5.31	1.56	0.0005	0.0024	MYLK3	myosin light chain kinase 3	Multiple_Complex
TC1600011535.hg.1	8.97	7.92	2.07	0.0005	0.0024	SIAH1	siah E3 ubiquitin protein ligase 1	Multiple_Complex
TC0700008707.hg.1	6.81	7.51	-1.63	0.0005	0.0024	RINT1	RAD50 interactor 1	Multiple_Complex
TC0100006695.hg.1	5.27	4.56	1.63	0.0005	0.0024	TAS1R1	taste receptor, type 1, member 1	Coding
TC0500012405.hg.1	4.7	4.16	1.45	0.0005	0.0024	HTR4	5-hydroxytryptamine (serotonin) receptor 4, G	Multiple_Complex
TC2100007465.hg.1	7.95	6.53	2.68	0.0005	0.0024	PCNT	pericentrin	Multiple_Complex
TC1200012655.hg.1	9.22	10.11	-1.85	0.0005	0.0025	HELB	helicase (DNA) B	Multiple_Complex
TC0500011554.hg.1	12.59	11.8	1.73	0.0005	0.0025	CHD1	chromodomain helicase DNA binding protein 1	Multiple_Complex
TC0100012901.hg.1	6.27	7.05	-1.72	0.0005	0.0025	KIAA2013	KIAA2013	Multiple_Complex
TC1900007847.hg.1	10.09	9.24	1.81	0.0005	0.0025	USF2	upstream transcription factor 2, c-fos interact	Multiple_Complex
TC1700010653.hg.1	6.94	6.49	1.37	0.0005	0.0025	KRTAP2-3	keratin associated protein 2-3	Coding
TC0300011223.hg.1	7.82	6.97	1.8	0.0005	0.0025	SELK	selenoprotein K; selenoprotein K [Source:Entre	Multiple_Complex
TC1100012853.hg.1	7.53	8.07	-1.45	0.0005	0.0025	SNX19	sorting nexin 19	Multiple_Complex
TC0800010298.hg.1	7.29	6.51	1.72	0.0005	0.0025	THAP1	THAP domain containing, apoptosis associated	Multiple_Complex
TC0X00006891.hg.1	10.22	9.2	2.03	0.0005	0.0025	GK	glycerol kinase	Multiple_Complex
TC1600009322.hg.1	4.37	4.84	-1.38	0.0005	0.0025	CARHSP1	calcium regulated heat stable protein 1	Multiple_Complex
TC0900012179.hg.1	7.36	8.23	-1.84	0.0005	0.0025	SPTAN1	spectrin, alpha, non-erythrocytic 1	Multiple_Complex
TC1200009852.hg.1	4.75	5.51	-1.69	0.0005	0.0025	PZP	pregnancy-zone protein	Multiple_Complex
TC2000007243.hg.1	7.67	8.27	-1.52	0.0005	0.0025	AAR2	AAR2 splicing factor homolog	Multiple_Complex
TC0600014249.hg.1	10.57	9.61	1.95	0.0005	0.0025	HIST1H2BC	histone cluster 1, H2bc	Coding
TC1700012054.hg.1	7.74	8.4	-1.58	0.0005	0.0025	NPLOC4	NPL4 homolog, ubiquitin recognition factor	Multiple_Complex
TC1200011320.hg.1	6.07	6.56	-1.41	0.0005	0.0025	BBS10	Bardet-Biedl syndrome 10	Coding
TC1600008149.hg.1	8.1	8.88	-1.72	0.0005	0.0025	E2F4	E2F transcription factor 4, p107/p130-binding	Multiple_Complex
TC1900009408.hg.1	8.34	8.94	-1.52	0.0005	0.0025	RANBP3	RAN binding protein 3	Multiple_Complex
TC1200007521.hg.1	5.79	5.21	1.49	0.0005	0.0025	ANP32D	acidic nuclear phosphoprotein 32 family mem	Coding
TSUnmapped00000198.h	6.5	7.05	-1.47	0.0005	0.0025	ZNF852	zinc finger protein 852	NonCoding
TC1900010158.hg.1	9.84	10.73	-1.85	0.0005	0.0025	ZNF100	zinc finger protein 100	Multiple_Complex
TC1700007787.hg.1	5.93	6.74	-1.75	0.0005	0.0025	WIPF2	WAS/WASL interacting protein family, membe	Multiple_Complex
TC1600007978.hg.1	4.3	3.88	1.33	0.0005	0.0025	SLC12A3	solute carrier family 12 (sodium/chloride trans	Multiple_Complex
TC0200011040.hg.1	10.56	11.55	-1.99	0.0006	0.0025	ITM2C	integral membrane protein 2C	Multiple_Complex
TC0100018248.hg.1	9.01	9.9	-1.85	0.0006	0.0025	RNPC3	RNA binding region (RNP1, RRM) containing 3	Multiple_Complex
TC1600006893.hg.1	7.94	8.84	-1.87	0.0006	0.0025	CLEC16A	C-type lectin domain family 16, member A	Multiple_Complex
TC0300006719.hg.1	6.19	6.63	-1.36	0.0006	0.0026	BTD	biotinidase	Multiple_Complex
TC2200008856.hg.1	7.54	8.22	-1.6	0.0006	0.0026	NAGA	N-acetylgalactosaminidase, alpha-	Multiple_Complex
TC1200009847.hg.1	4.56	5.49	-1.91	0.0006	0.0026	A2M	alpha-2-macroglobulin	Multiple_Complex
TC0400009223.hg.1	3.98	3.4	1.5	0.0006	0.0026	CPE	carboxypeptidase E	Coding

TC1700010663.hg.1	4.37	3.58	1.73	0.0006	0.0026	KRTAP16-1	keratin associated protein 16-1	Coding
TC1800007805.hg.1	4.94	5.91	-1.96	0.0006	0.0026	NFATC1	nuclear factor of activated T-cells, cytoplasmic	Multiple_Complex
TC1400007628.hg.1	11.69	10.69	2	0.0006	0.0026	PSEN1	presenilin 1	Multiple_Complex
TC0200016753.hg.1	4.93	5.65	-1.65	0.0006	0.0026	CCDC141	coiled-coil domain containing 141	Multiple_Complex
TC1500010857.hg.1	6.56	8.32	-3.37	0.0006	0.0026	EPB42	erythrocyte membrane protein band 4.2	Multiple_Complex
TC0100015180.hg.1	8.57	7.67	1.86	0.0006	0.0026	WDR47	WD repeat domain 47	Multiple_Complex
TC0500007673.hg.1	7.58	8.4	-1.77	0.0006	0.0026	RAD17	RAD17 checkpoint clamp loader component	Multiple_Complex
TC0900009944.hg.1	6.74	7.7	-1.93	0.0006	0.0026	CD72	CD72 molecule	Multiple_Complex
TC0600010960.hg.1	9.02	7.89	2.19	0.0006	0.0026	TPMT	thiopurine S-methyltransferase	Multiple_Complex
TC0200007077.hg.1	7.83	8.5	-1.59	0.0006	0.0026	SLC4A1AP	solute carrier family 4 (anion exchanger), mem	Multiple_Complex
TC1400010727.hg.1	5.43	6.49	-2.09	0.0006	0.0026	RIPK3	receptor-interacting serine-threonine kinase 3	Multiple_Complex
TC1700009748.hg.1	8.61	9.12	-1.43	0.0006	0.0026	SCO1	SCO1 cytochrome c oxidase assembly protein	Multiple_Complex
TC1900009346.hg.1	7.07	8.66	-3	0.0006	0.0026	UBXN6	UBX domain protein 6	Multiple_Complex
TC0200010607.hg.1	9.68	10.53	-1.8	0.0006	0.0026	CREB1	cAMP responsive element binding protein 1	Multiple_Complex
TC0100011692.hg.1	9.18	10.06	-1.84	0.0006	0.0026	MIA3	melanoma inhibitory activity family, member	Multiple_Complex
TC1300009898.hg.1	5.1	4.43	1.59	0.0006	0.0026	GRTP1	growth hormone regulated TBC protein 1	Multiple_Complex
TC0900010387.hg.1	7.38	6.62	1.7	0.0006	0.0026	TMEM2	Transcript Identified by AceView, Entrez Gene	Unassigned
TC1700007105.hg.1	5.7	6.26	-1.48	0.0006	0.0026	LLGL1	lethal giant larvae homolog 1 (Drosophila)	Multiple_Complex
TC2000007717.hg.1	5.65	6.48	-1.78	0.0006	0.0026	MOCS3	molybdenum cofactor synthesis 3	Coding
TC1800006915.hg.1	4.87	6.11	-2.36	0.0006	0.0027	HRH4	histamine receptor H4	Coding
TC0100008321.hg.1	5.19	6.02	-1.78	0.0006	0.0027	GPX7	glutathione peroxidase 7	Multiple_Complex
TC0400008618.hg.1	4.71	4.16	1.46	0.0006	0.0027	FGF2	fibroblast growth factor 2 (basic)	Multiple_Complex
TC1100007400.hg.1	6.84	7.69	-1.8	0.0006	0.0027	TSPAN18	tetraspanin 18	Multiple_Complex
TC1900011537.hg.1	3.84	4.48	-1.56	0.0006	0.0027	ZNF835	Transcript Identified by AceView, Entrez Gene	Coding
TC1400008760.hg.1	7.74	8.17	-1.34	0.0006	0.0027	SDR39U1	short chain dehydrogenase/reductase family 3	Multiple_Complex
TC1600011424.hg.1	6.99	7.65	-1.58	0.0006	0.0027	DDX19B	DEAD (Asp-Glu-Ala-Asp) box polypeptide 19B	Multiple_Complex
TC0200016224.hg.1	5.24	4.64	1.52	0.0006	0.0027	PER2	period circadian clock 2	Multiple_Complex
TC1000011445.hg.1	7.09	7.89	-1.74	0.0006	0.0027	MYOF	myoferlin	Multiple_Complex
TC0300009651.hg.1	10.05	9.47	1.5	0.0006	0.0027	ATP11B	ATPase, class VI, type 11B	Multiple_Complex
TC0800012343.hg.1	6.18	5.69	1.41	0.0006	0.0027	ZFPM2	zinc finger protein, FOG family member 2	NonCoding
TC0100017368.hg.1	6.71	7.4	-1.61	0.0006	0.0027	BPNT1	3(2), 5-bisphosphate nucleotidase 1	Multiple_Complex
TC0700013611.hg.1	3.77	3.49	1.21	0.0006	0.0027	TFEC	transcription factor EC	NonCoding
TSUnmapped00000205.h	9.2	10.02	-1.76	0.0006	0.0027	SURF4	surfeit 4	Coding
TC0400011579.hg.1	6.79	5.77	2.02	0.0006	0.0027	PLA2G12A	phospholipase A2, group XIIA	Multiple_Complex
TC0900009513.hg.1	5.59	6.43	-1.8	0.0006	0.0027	TMEM261	transmembrane protein 261	Multiple_Complex
TC2200009364.hg.1	7.77	7.04	1.66	0.0006	0.0027	CPT1B	carnitine palmitoyltransferase 1B (muscle)	Multiple_Complex
TC1900009682.hg.1	6.4	7.39	-1.98	0.0006	0.0027	ELOF1	ELF1 homolog, elongation factor 1	Multiple_Complex
TC0X00010640.hg.1	7.37	6.86	1.43	0.0006	0.0027	CXorf56	chromosome X open reading frame 56	Multiple_Complex
TC0700008501.hg.1	5.87	6.64	-1.7	0.0006	0.0027	ZSCAN25	zinc finger and SCAN domain containing 25	Multiple_Complex
TC0400010609.hg.1	5.58	4.95	1.54	0.0006	0.0027	CORIN	corin, serine peptidase	Multiple_Complex
TC1100012610.hg.1	5.87	4.88	1.99	0.0006	0.0027	BSX	brain-specific homeobox	Multiple_Complex
TC0400011130.hg.1	7.12	7.7	-1.5	0.0006	0.0027	PAQR3	progestin and adipoQ receptor family membe	Multiple_Complex
TC0500007154.hg.1	7	6.22	1.73	0.0006	0.0027	SLC1A3	solute carrier family 1 (glial high affinity glutar	Multiple_Complex
TC0300012834.hg.1	5.43	6.53	-2.14	0.0006	0.0028	TMEM14EF	transmembrane protein 14E, pseudogene	Coding
TC0700008690.hg.1	12.62	12	1.54	0.0006	0.0028	KMT2E	lysine (K)-specific methyltransferase 2E	Multiple_Complex
TC1100009335.hg.1	5.08	5.81	-1.66	0.0006	0.0028	C11orf63	chromosome 11 open reading frame 63	Multiple_Complex
TC0300011324.hg.1	4.43	5.17	-1.67	0.0006	0.0028	FHIT	fragile histidine triad	Multiple_Complex
TC1200009749.hg.1	4.68	4.22	1.37	0.0006	0.0028	PIANP	PILR alpha associated neural protein	Coding
TC1500010712.hg.1	7.92	9.2	-2.43	0.0006	0.0028	GOLGA8N	golgin A8 family, member N	Multiple_Complex
TC0900006953.hg.1	5.34	4.8	1.46	0.0006	0.0028	TMEM215	transmembrane protein 215	Coding
TC0300011044.hg.1	6.56	7.23	-1.59	0.0006	0.0028	DALRD3	DALR anticodon binding domain containing 3	Multiple_Complex
TC0X00008492.hg.1	4.58	4.18	1.32	0.0006	0.0028	SMIM10	small integral membrane protein 10	Multiple_Complex
TC0700013435.hg.1	5.15	5.73	-1.5	0.0006	0.0028	CDHR3	cadherin-related family member 3	NonCoding
TC1200012100.hg.1	6.24	5.43	1.75	0.0006	0.0028	KSR2	kinase suppressor of ras 2	Multiple_Complex
TC1100010156.hg.1	7.77	7.27	1.42	0.0006	0.0028	BTBD10	BTB (POZ) domain containing 10	Multiple_Complex
TC2000007430.hg.1	11.08	10.29	1.73	0.0006	0.0028	SRSF6	serine/arginine-rich splicing factor 6	Multiple_Complex
TC0500010766.hg.1	5.52	5.95	-1.35	0.0006	0.0028	PLPP1; RNF	phospholipid phosphatase 1; ring finger prote	Multiple_Complex
TC1300007167.hg.1	11.06	12.1	-2.05	0.0006	0.0028	PHF11	PHD finger protein 11	Multiple_Complex
TC0900012215.hg.1	4.7	3.94	1.69	0.0006	0.0028	IFNA16	interferon, alpha 16	Coding
TC2100008465.hg.1	6.42	7.08	-1.58	0.0006	0.0028	MCM3AP	minichromosome maintenance complex comp	Multiple_Complex
TC0100018387.hg.1	9.43	8.41	2.04	0.0006	0.0028	CDK11B	cyclin-dependent kinase 11B	Multiple_Complex
TC0500009175.hg.1	9.86	10.88	-2.02	0.0006	0.0028	LARP1	La ribonucleoprotein domain family, member	Multiple_Complex
TC0100016035.hg.1	9.65	8.95	1.63	0.0006	0.0028	ETV3	ets variant 3	Multiple_Complex
TC1900010543.hg.1	4.09	4.61	-1.43	0.0006	0.0028	ZNF829	zinc finger protein 829	Multiple_Complex
TC1000012491.hg.1	7.55	6.74	1.75	0.0006	0.0028	ENTPD7	ectonucleoside triphosphate diphosphohydrol	Multiple_Complex
TC0200011624.hg.1	7.76	11.65	-14.8	0.0006	0.0028	CMPK2	cytidine monophosphate (UMP-CMP) kinase 2	Multiple_Complex
TC0500013175.hg.1	6.61	7.29	-1.6	0.0006	0.0028	IPO11; LRR	importin 11; leucine rich repeat containing 70	Multiple_Complex
TC0600008760.hg.1	7.13	7.68	-1.46	0.0006	0.0028	PM20D2	peptidase M20 domain containing 2	Multiple_Complex
TC2000009218.hg.1	4.88	4.22	1.58	0.0006	0.0028	SDC4	syndecan 4	Multiple_Complex
TC0100007378.hg.1	8.31	7.38	1.91	0.0006	0.0028	CLIC4	chloride intracellular channel 4	Multiple_Complex

TC0X00009218.hg.1	10.7	9.88	1.76	0.0006	0.0028	RPS6KA3	ribosomal protein S6 kinase, 90kDa, polypepti	Multiple_Complex
TC2000009947.hg.1	8.63	7.89	1.67	0.0006	0.0028	STX16-NPE	STX16-NPEPL1 readthrough (NMD candidate)	Multiple_Complex
TC0400009038.hg.1	11.01	11.71	-1.62	0.0006	0.0028	KIAA0922	KIAA0922	Multiple_Complex
TC1100009463.hg.1	5.73	5.26	1.39	0.0006	0.0028	KIRREL3-AS	KIRREL3 antisense RNA 3	Multiple_Complex
TC0400010260.hg.1	6.47	7.31	-1.79	0.0006	0.0028	SEPSECS	Sep (O-phosphoserine) tRNA:Sec (selenocyste	Multiple_Complex
TC1600010307.hg.1	4.82	4.08	1.67	0.0006	0.0028	C16orf97	chromosome 16 open reading frame 97	Multiple_Complex
TC1900009310.hg.1	5.14	5.98	-1.8	0.0006	0.0028	MATK	megakaryocyte-associated tyrosine kinase	Multiple_Complex
TC1200012808.hg.1	8.72	9.39	-1.59	0.0006	0.0028	R3HDM2	R3H domain containing 2	Multiple_Complex
TC1400007265.hg.1	8.71	9.43	-1.64	0.0006	0.0028	TMEM260	transmembrane protein 260	Multiple_Complex
TC1600011582.hg.1	4.19	4.7	-1.42	0.0006	0.0028	KLHDC4	kelch domain containing 4	Multiple_Complex
TC0600014055.hg.1	9.67	7.98	3.24	0.0006	0.0028	NQO2	NAD(P)H dehydrogenase, quinone 2	Multiple_Complex
TC0700013388.hg.1	9.88	10.36	-1.39	0.0006	0.0029	STAG3L1	stromal antigen 3-like 1 (pseudogene)	Multiple_Complex
TC0400012583.hg.1	7.29	6.92	1.29	0.0006	0.0029	RWDD4	RWD domain containing 4	Multiple_Complex
TSUnmapped00000763.h	6.67	7.21	-1.46	0.0006	0.0029	SLC16A1	solute carrier family 16 (monocarboxylate trar	Coding
TC0100013897.hg.1	6.52	7.03	-1.42	0.0006	0.0029	P3H1	prolyl 3-hydroxylase 1	Multiple_Complex
TC0200008447.hg.1	5.77	5.36	1.33	0.0006	0.0029	TEKT4	tektin 4	Multiple_Complex
TC1700006480.hg.1	7.32	7.87	-1.47	0.0006	0.0029	TIMM22	translocase of inner mitochondrial membrane	Multiple_Complex
TC0600013273.hg.1	10.63	9.73	1.87	0.0006	0.0029	BCLAF1	Memczak2013 ALT_DONOR, coding, INTERNA	NonCoding
TC0X00010052.hg.1	5.02	4.36	1.58	0.0006	0.0029	NAP1L2	Transcript Identified by AceView, Entrez Gene	Unassigned
TC0800006624.hg.1	4.43	3.89	1.46	0.0006	0.0029	DEFB4A	defensin, beta 4A	Coding
TC1500010463.hg.1	5.69	5.07	1.54	0.0006	0.0029	PRC1	protein regulator of cytokinesis 1	Multiple_Complex
TC1800007101.hg.1	5.44	6.11	-1.59	0.0006	0.0029	KIAA1328	KIAA1328	Multiple_Complex
TC0300014050.hg.1	6.52	7.28	-1.7	0.0006	0.0029	NPHP3	nephronophthisis 3 (adolescent)	Multiple_Complex
TC1700006665.hg.1	5.18	5.69	-1.42	0.0006	0.0029	ZFP3	ZFP3 zinc finger protein	Coding
TC0100018359.hg.1	9.48	8.57	1.88	0.0006	0.0029	TSNAX	translin-associated factor X	Multiple_Complex
TC0200012076.hg.1	8.2	9.13	-1.91	0.0006	0.0029	PPM1G	protein phosphatase, Mg2+/Mn2+ dependent	Multiple_Complex
TC1100006674.hg.1	4.39	3.66	1.66	0.0006	0.0029	OR51T1	olfactory receptor, family 51, subfamily T, mei	Coding
TC0800012323.hg.1	6.92	6	1.89	0.0006	0.0029	CA13	carbonic anhydrase XIII	Multiple_Complex
TC0700008495.hg.1	6.37	5.27	2.15	0.0007	0.0029	BUD31	Transcript Identified by AceView, Entrez Gene	Unassigned
TC0700009872.hg.1	6.01	6.85	-1.79	0.0007	0.0029	UBE3C	ubiquitin protein ligase E3C	Multiple_Complex
TC1500010049.hg.1	3.67	4.35	-1.6	0.0007	0.0029	FAM219B	family with sequence similarity 219, member	Multiple_Complex
TC1000011579.hg.1	5.87	6.79	-1.89	0.0007	0.0029	HPS1; MIR4	Hermansky-Pudlak syndrome 1; microRNA 46	Multiple_Complex
TC1200012713.hg.1	7.4	6.72	1.61	0.0007	0.0029	MAP1LC3B	microtubule-associated protein 1 light chain 3	Coding
TC1600006448.hg.1	4.89	5.64	-1.69	0.0007	0.003	HBQ1	hemoglobin, theta 1	Coding
TC1000009863.hg.1	10.5	9.61	1.85	0.0007	0.003	FAM107B	family with sequence similarity 107, member	Multiple_Complex
TC0600008066.hg.1	7.61	8.32	-1.63	0.0007	0.003	KLHDC3	kelch domain containing 3	Multiple_Complex
TC0100017029.hg.1	4.98	5.87	-1.85	0.0007	0.003	PIK3C2B	phosphatidylinositol-4-phosphate 3-kinase, ca	Multiple_Complex
TC0100016053.hg.1	5.19	4.51	1.61	0.0007	0.003	CD5L	CD5 molecule-like	Multiple_Complex
TC0300012145.hg.1	5.7	4.99	1.64	0.0007	0.003	FSTL1; MIR	follistatin like 1; microRNA 198	Multiple_Complex
TC1200012068.hg.1	5.96	6.48	-1.44	0.0007	0.003	C12orf49	chromosome 12 open reading frame 49	Multiple_Complex
TC0200010837.hg.1	7.96	8.56	-1.51	0.0007	0.003	ANKZF1	ankyrin repeat and zinc finger domain contain	Multiple_Complex
TC1200011996.hg.1	3.49	3.99	-1.41	0.0007	0.003	LHX5	LIM homeobox 5	Multiple_Complex
TC2200007614.hg.1	7.12	7.99	-1.83	0.0007	0.003	FAM118A	family with sequence similarity 118, member	Multiple_Complex
TC0X00007733.hg.1	4.39	3.71	1.59	0.0007	0.003	FAM46D	family with sequence similarity 46, member D	Coding
TC0900009339.hg.1	7.19	7.91	-1.65	0.0007	0.003	WASH1	WAS protein family homolog 1	Multiple_Complex
TC1600009066.hg.1	7.51	8.61	-2.15	0.0007	0.003	HAGH	hydroxyacylglutathione hydrolase	Multiple_Complex
TC1200012766.hg.1	4.89	4.54	1.28	0.0007	0.003	PRB1	proline-rich protein BstNI subfamily 1	Coding
TC0900008150.hg.1	7.34	8.59	-2.38	0.0007	0.003	TMOD1	tropomodulin 1	Multiple_Complex
TC0X00006809.hg.1	9.99	9.18	1.76	0.0007	0.003	ZFX	zinc finger protein, X-linked	Multiple_Complex
TC2200006862.hg.1	7.74	8.42	-1.6	0.0007	0.003	SMARCB1	SWI/SNF related, matrix associated, actin dep	Multiple_Complex
TC1000012565.hg.1	6.01	6.74	-1.66	0.0007	0.0031	DNAJC9	DnaJ (Hsp40) homolog, subfamily C, member	NonCoding
TC1700006574.hg.1	5.22	4.7	1.43	0.0007	0.0031	OR1D4	olfactory receptor, family 1, subfamily D, mem	Multiple_Complex
TC1100011182.hg.1	5.67	4.79	1.84	0.0007	0.0031	PYGM	phosphorylase, glycogen, muscle	Multiple_Complex
TC1400007155.hg.1	9.66	8.59	2.11	0.0007	0.0031	C14orf166	chromosome 14 open reading frame 166	Multiple_Complex
TC0700010035.hg.1	6.09	6.65	-1.48	0.0007	0.0031	MAD1L1	MAD1 mitotic arrest deficient-like 1 (yeast)	Multiple_Complex
TC0100007954.hg.1	16.55	15.4	2.22	0.0007	0.0031	SMAP2	small ArfGAP2	Multiple_Complex
TC2100007355.hg.1	7.54	8.17	-1.55	0.0007	0.0031	PFKL	phosphofructokinase, liver	Multiple_Complex
TC0600008183.hg.1	8	8.8	-1.74	0.0007	0.0031	CD2AP	CD2-associated protein	Multiple_Complex
TC0400008427.hg.1	5.9	6.41	-1.42	0.0007	0.0031	AP1AR	adaptor-related protein complex 1 associated	Multiple_Complex
TC0200012022.hg.1	6.44	5.64	1.75	0.0007	0.0031	KIF3C	kinesin family member 3C	Multiple_Complex
TC1100011090.hg.1	6.54	7.03	-1.4	0.0007	0.0031	EML3	echinoderm microtubule associated protein lil	Multiple_Complex
TSUnmapped00000528.h	4.32	3.89	1.34	0.0007	0.0031	HNRNPCL1	heterogeneous nuclear ribonucleoprotein C-lil	Coding
TC2000007627.hg.1	8.22	9.02	-1.75	0.0007	0.0031	CSE1L	CSE1 chromosome segregation 1-like (yeast)	Multiple_Complex
TC1700007779.hg.1	12.21	11.02	2.29	0.0007	0.0031	MSL1	male-specific lethal 1 homolog (Drosophila)	Multiple_Complex
TC1600007741.hg.1	5.23	6.15	-1.88	0.0007	0.0031	PHKB	Transcript Identified by AceView, Entrez Gene	Unassigned
TC1900008399.hg.1	8.4	9.2	-1.74	0.0007	0.0031	ARHGAP35	Rho GTPase activating protein 35	Multiple_Complex
TC0100010525.hg.1	10.49	11.06	-1.49	0.0007	0.0031	SFT2D2	SFT2 domain containing 2	Multiple_Complex
TC1200010109.hg.1	10.37	8.31	4.17	0.0007	0.0031	LDHB	lactate dehydrogenase B	Multiple_Complex
TC1700006735.hg.1	5.49	5.09	1.32	0.0007	0.0031	SLC2A4	solute carrier family 2 (facilitated glucose tran	Coding
TC1500009216.hg.1	4.06	3.56	1.41	0.0007	0.0031	TGM7	transglutaminase 7	Multiple_Complex

TC0900007010.hg.1	12.05	11.01	2.07	0.0007	0.0032	UBE2R2	ubiquitin-conjugating enzyme E2R 2	Multiple_Complex
TC0100018514.hg.1	4.95	4.51	1.36	0.0007	0.0032	VSIG8	V-set and immunoglobulin domain containing	Coding
TC1900008696.hg.1	6.54	7.25	-1.64	0.0007	0.0032	ZNF880	zinc finger protein 880	Multiple_Complex
TC0300009706.hg.1	5.15	5.93	-1.72	0.0007	0.0032	POLR2H	polymerase (RNA) II (DNA directed) polypepti	Multiple_Complex
TC1700007904.hg.1	8.17	8.83	-1.58	0.0007	0.0032	TUBG1	tubulin, gamma 1	Multiple_Complex
TC2200007085.hg.1	8.17	8.56	-1.31	0.0007	0.0032	RNF185	ring finger protein 185	Multiple_Complex
TC0200006833.hg.1	5.1	5.93	-1.78	0.0007	0.0032	GEN1	GEN1 Holliday junction 5 flap endonuclease	Multiple_Complex
TC0200012299.hg.1	5.83	6.5	-1.59	0.0007	0.0032	DHX57	DEAH (Asp-Glu-Ala-Asp/His) box polypeptide 5	Multiple_Complex
TC1100008800.hg.1	5.21	4.69	1.43	0.0007	0.0032	IZUMO1R	IZUMO1 receptor, JUNO	Coding
TC1000008769.hg.1	5.69	6.36	-1.59	0.0007	0.0032	TAF5	TAF5 RNA polymerase II, TATA box binding pro	Multiple_Complex
TC1400007942.hg.1	6.87	7.46	-1.5	0.0007	0.0032	TDP1	tyrosyl-DNA phosphodiesterase 1	Multiple_Complex
TC0X00007194.hg.1	8.45	8.97	-1.43	0.0007	0.0032	TBC1D25	TBC1 domain family, member 25	Multiple_Complex
TC0800010630.hg.1	4.6	4.08	1.43	0.0007	0.0032	GGH	gamma-glutamyl hydrolase (conjugase, folylp	Multiple_Complex
TC1900011684.hg.1	6.86	7.63	-1.7	0.0007	0.0032	BABAM1	BRISC and BRCA1 A complex member 1	Multiple_Complex
TC0100009658.hg.1	9.36	10.13	-1.69	0.0007	0.0032	SRGAP2C	SLIT-ROBO Rho GTPase activating protein 2C	Multiple_Complex
TC0100015784.hg.1	5.76	7.73	-3.91	0.0007	0.0032	SELENBP1	selenium binding protein 1	Multiple_Complex
TC1700009613.hg.1	7.42	6.03	2.62	0.0007	0.0032	ASGR2	asialoglycoprotein receptor 2	Multiple_Complex
TC1000006863.hg.1	6.1	6.88	-1.71	0.0007	0.0032	RPP38	ribonuclease P/MRP 38kDa subunit	Multiple_Complex
TC0600011876.hg.1	11.54	12.67	-2.2	0.0007	0.0032	YIPF3	Yip1 domain family member 3	Multiple_Complex
TC1100009817.hg.1	4.73	4.36	1.29	0.0007	0.0032	PHLDA2	pleckstrin homology-like domain, family A, me	Multiple_Complex
TC1600009531.hg.1	7.84	7.33	1.42	0.0007	0.0032	KIAA0430	Transcript Identified by AceView, Entrez Gene	Unassigned
TC0500009183.hg.1	5.17	4.48	1.61	0.0007	0.0032	MRPL22	mitochondrial ribosomal protein L22	Multiple_Complex
TC1700012446.hg.1	11.7	10.92	1.72	0.0007	0.0032	CCDC47	coiled-coil domain containing 47	Multiple_Complex
TC1800009221.hg.1	7.09	6.17	1.89	0.0007	0.0032	RBBP8; MIF	retinoblastoma binding protein 8; microRNA 4	Multiple_Complex
TC0600011134.hg.1	7.67	8.55	-1.84	0.0007	0.0032	HIST1H4D	histone cluster 1, H4d	Coding
TC1900007290.hg.1	5.64	5.02	1.54	0.0007	0.0032	TMEM38A	transmembrane protein 38A	Multiple_Complex
TC1600007191.hg.1	6.15	6.83	-1.61	0.0007	0.0032	EEF2K	eukaryotic elongation factor 2 kinase	Multiple_Complex
TC1200012654.hg.1	5.93	6.5	-1.49	0.0007	0.0032	TSFM	Ts translation elongation factor, mitochondria	Multiple_Complex
TC1900011389.hg.1	6.54	5.55	1.98	0.0007	0.0032	VSTM1	V-set and transmembrane domain containing	Multiple_Complex
TC1500007833.hg.1	8.18	9.75	-2.97	0.0007	0.0032	PML	promyelocytic leukemia	Multiple_Complex
TC0800011314.hg.1	8.33	7.49	1.8	0.0007	0.0032	UBR5	ubiquitin protein ligase E3 component n-recog	Multiple_Complex
TC0200013517.hg.1	10.38	11.27	-1.86	0.0007	0.0032	SNRNP200	small nuclear ribonucleoprotein, U5 200kDa su	Multiple_Complex
TC1100009686.hg.1	6.22	5.67	1.46	0.0007	0.0032	CDHR5	cadherin-related family member 5	Multiple_Complex
TC0100015952.hg.1	7.65	8.54	-1.85	0.0007	0.0032	CLK2	CDC like kinase 2	Multiple_Complex
TC0300011403.hg.1	4.86	4.28	1.49	0.0007	0.0032	MAGI1	membrane associated guanylate kinase, WW a	Multiple_Complex
TC1500010738.hg.1	4.37	3.8	1.49	0.0007	0.0033	CTXN2	cortexin 2	Coding
TC0900011217.hg.1	15.05	15.79	-1.67	0.0007	0.0033	PTBP3	polypyrimidine tract binding protein 3	Multiple_Complex
TC0X00008533.hg.1	7.46	8.04	-1.49	0.0007	0.0033	HTATSF1	HIV-1 Tat specific factor 1	Coding
TC1900006640.hg.1	8.04	7.58	1.37	0.0007	0.0033	FZR1	fizzy/cell division cycle 20 related 1	Multiple_Complex
TC0100017729.hg.1	8.24	7.62	1.54	0.0007	0.0033	EXOC8	exocyst complex component 8	Coding
TC0700006813.hg.1	8.5	9	-1.42	0.0007	0.0033	HDAC9	histone deacetylase 9	Multiple_Complex
TC1200010926.hg.1	11.66	11.06	1.51	0.0007	0.0033	BAZ2A	bromodomain adjacent to zinc finger domain	Multiple_Complex
TC0200010253.hg.1	5.97	6.78	-1.75	0.0007	0.0033	ANKAR	ankyrin and armadillo repeat containing	Multiple_Complex
TC1900007115.hg.1	5.7	6.25	-1.46	0.0007	0.0033	NFIX	nuclear factor I/X (CCAAT-binding transcriptio	Multiple_Complex
TC0200016647.hg.1	8.77	7.93	1.79	0.0007	0.0033	DPY30	dpy-30 histone methyltransferase complex re	Multiple_Complex
TC0700008263.hg.1	7	6.32	1.6	0.0007	0.0033	DBF4	DBF4 zinc finger	Multiple_Complex
TC1700010545.hg.1	6.5	7.19	-1.62	0.0007	0.0033	PIP4K2B	phosphatidylinositol-5-phosphate 4-kinase, ty	Multiple_Complex
TC0600012296.hg.1	18.67	17.48	2.29	0.0007	0.0033	EEF1A1	eukaryotic translation elongation factor 1 alph	Multiple_Complex
TC1900007349.hg.1	16.83	17.82	-1.99	0.0007	0.0033	RPL18A; SN	ribosomal protein L18a; small nucleolar RNA,	Multiple_Complex
TC1900008498.hg.1	10.8	10	1.75	0.0008	0.0033	NUCB1	nucleobindin 1	Multiple_Complex
TC0600007868.hg.1	5.64	6.34	-1.62	0.0008	0.0033	RNF8	ring finger protein 8, E3 ubiquitin protein ligas	Multiple_Complex
TC1700007344.hg.1	8.19	8.9	-1.64	0.0008	0.0033	NLK	nemo-like kinase	Multiple_Complex
TC0100008341.hg.1	4.64	5.22	-1.5	0.0008	0.0033	PODN	podocan	Multiple_Complex
TSUnmapped00000053.h	7.32	6.68	1.57	0.0008	0.0033	RPL7A	ribosomal protein L7a	NonCoding
TC1900011852.hg.1	6.42	5.45	1.96	0.0008	0.0033	RFX2	regulatory factor X, 2 (influences HLA class II e	Multiple_Complex
TC1200009247.hg.1	12.42	11.66	1.7	0.0008	0.0034	TMED2	transmembrane p24 trafficking protein 2	Multiple_Complex
TC0X00007200.hg.1	8.27	8.74	-1.39	0.0008	0.0034	SUV39H1	suppressor of variegation 3-9 homolog 1 (Dros	Multiple_Complex
TC1400009337.hg.1	11.22	10.29	1.91	0.0008	0.0034	DHRS7	dehydrogenase/reductase (SDR family) memb	Multiple_Complex
TC1900011282.hg.1	4.36	5.06	-1.62	0.0008	0.0034	ZNF614	zinc finger protein 614	Multiple_Complex
TC1500008900.hg.1	5.02	5.68	-1.58	0.0008	0.0034	LOC283710	uncharacterized LOC283710; novel transcript;	Multiple_Complex
TC1200008739.hg.1	6.64	7.42	-1.73	0.0008	0.0034	TMEM263	transmembrane protein 263	Multiple_Complex
TC1200012775.hg.1	3.87	4.56	-1.62	0.0008	0.0034	ST8SIA1	ST8 alpha-N-acetyl-neuraminide alpha-2,8-sia	Multiple_Complex
TSUnmapped00000377.h	9.71	10.34	-1.55	0.0008	0.0034	INPP5D	inositol polyphosphate-5-phosphatase D	NonCoding
TC0900011835.hg.1	8.44	7.75	1.61	0.0008	0.0034	OBP2B	odorant binding protein 2B	Multiple_Complex
TC1900008031.hg.1	7.08	6.57	1.43	0.0008	0.0034	LGALS7B	lectin, galactoside-binding, soluble, 7B	Multiple_Complex
TC0200016673.hg.1	9.7	9.08	1.53	0.0008	0.0034	RTN4	reticulon 4	Multiple_Complex
TC0300014092.hg.1	5.35	6.02	-1.59	0.0008	0.0034	TM4SF19-T	TM4SF19-TCTEX1D2 readthrough (NMD candi	Multiple_Complex
TC1900009630.hg.1	5.57	6.27	-1.63	0.0008	0.0034	KEAP1	kelch-like ECH-associated protein 1	Multiple_Complex
TC0X00009803.hg.1	4.63	4.06	1.48	0.0008	0.0034	MTRNR2L1	MT-RNR2-like 10	Coding
TC1500010883.hg.1	7.08	7.9	-1.76	0.0008	0.0034	SNAPC5	small nuclear RNA activating complex polypep	Coding

TC0100018443.hg.1	5.61	4.98	1.56	0.0008	0.0034	PLPP3	phospholipid phosphatase 3	Multiple_Complex
TC1900008300.hg.1	8.08	7.26	1.76	0.0008	0.0034	RELB	v-rel avian reticuloendotheliosis viral oncogen	Multiple_Complex
TC0X00006758.hg.1	7.04	7.74	-1.63	0.0008	0.0034	CNKSR2	connector enhancer of kinase suppressor of R	Multiple_Complex
TC1900007859.hg.1	11.44	10.28	2.25	0.0008	0.0034	FFAR2	free fatty acid receptor 2	Multiple_Complex
TC0600013539.hg.1	6.5	5.6	1.86	0.0008	0.0034	RAET1L	retinoic acid early transcript 1L	Coding
TC0500012588.hg.1	5.28	6.24	-1.94	0.0008	0.0034	HAVCR1	hepatitis A virus cellular receptor 1	Multiple_Complex
TC0Y00006858.hg.1	9.24	8.5	1.68	0.0008	0.0034	VAMP7	Homo sapiens vesicle-associated membrane p	Multiple_Complex
TC0200011980.hg.1	11.85	12.3	-1.37	0.0008	0.0034	ITSN2	intersectin 2	Multiple_Complex
TC0900007740.hg.1	6.25	6.98	-1.66	0.0008	0.0035	RMI1	RecQ mediated genome instability 1	Coding
TC0400007055.hg.1	8.21	7.31	1.87	0.0008	0.0035	CDC42	Transcript Identified by AceView, Entrez Gene	Unassigned
TC0400011407.hg.1	3.81	3.42	1.31	0.0008	0.0035	ADH1A	alcohol dehydrogenase 1A (class I), alpha poly	Multiple_Complex
TC0200016562.hg.1	4.95	5.29	-1.26	0.0008	0.0035	PHOSPHO2	phosphatase, orphan 2	Multiple_Complex
TC0800010524.hg.1	8.47	9.06	-1.51	0.0008	0.0035	IMPAD1	inositol monophosphatase domain containing	Multiple_Complex
TC0300013835.hg.1	5.74	6.28	-1.45	0.0008	0.0035	TEX264	testis expressed 264	Multiple_Complex
TC1400009131.hg.1	9.31	10.22	-1.88	0.0008	0.0035	MAP4K5	mitogen-activated protein kinase kinase kinas	Multiple_Complex
TSUnmapped00000777.h	7.17	8.04	-1.83	0.0008	0.0035	HYOU1	hypoxia up-regulated 1	Coding
TC0300008487.hg.1	4.58	3.94	1.56	0.0008	0.0035	MAATS1	MYCBP-associated, testis expressed 1	Multiple_Complex
TC1600011493.hg.1	15.39	12.62	6.82	0.0008	0.0035	RPS15A	ribosomal protein S15a	Multiple_Complex
TC2200007906.hg.1	10.6	9.75	1.8	0.0008	0.0035	BID	BH3 interacting domain death agonist	Multiple_Complex
TC0100018434.hg.1	8.32	7.51	1.74	0.0008	0.0035	MYCBP; GJ.	MYC binding protein; gap junction protein alp	Multiple_Complex
TC0X00009153.hg.1	6.27	6.86	-1.5	0.0008	0.0035	CTPS2	CTP synthase 2	Multiple_Complex
TC0100012523.hg.1	8.07	8.82	-1.69	0.0008	0.0035	MORN1	MORN repeat containing 1	Multiple_Complex
TC0200015242.hg.1	15.19	16.72	-2.88	0.0008	0.0035	STAT1	signal transducer and activator of transcriptio	Multiple_Complex
TC1900011791.hg.1	5.54	6.26	-1.65	0.0008	0.0035	ZNF613	zinc finger protein 613	Multiple_Complex
TC1200007810.hg.1	7.77	8.15	-1.3	0.0008	0.0035	ORMDL2	ORMDL sphingolipid biosynthesis regulator 2	Multiple_Complex
TC1200009964.hg.1	11.08	9.45	3.09	0.0008	0.0036	MANSC1	MANSC domain containing 1	Coding
TC0600007789.hg.1	5.56	6.24	-1.6	0.0008	0.0036	ZNF76	zinc finger protein 76	Multiple_Complex
TSUnmapped00000192.h	6.84	7.6	-1.69	0.0008	0.0036	DGKD	diacylglycerol kinase, delta 130kDa	NonCoding
TC0500011333.hg.1	12.12	11.31	1.76	0.0008	0.0036	TMEM167A	transmembrane protein 167A	Multiple_Complex
TC0200009370.hg.1	3.81	3.16	1.57	0.0008	0.0036	ZNF806	zinc finger protein 806	Multiple_Complex
TC0500008810.hg.1	13.43	12.07	2.57	0.0008	0.0036	MATR3; SN	matrin 3; small nucleolar RNA, H/ACA box 74A	Multiple_Complex
TC1500010255.hg.1	5.37	5.93	-1.47	0.0008	0.0036	C15orf40	chromosome 15 open reading frame 40	Multiple_Complex
TC0300006677.hg.1	14.56	13.81	1.67	0.0008	0.0036	SLC6A6	solute carrier family 6 (neurotransmitter trans	Multiple_Complex
TC0900007844.hg.1	7.78	8.56	-1.72	0.0008	0.0036	SPIN1	spindlin 1	Multiple_Complex
TC1600010407.hg.1	9.51	10.13	-1.54	0.0008	0.0036	NUDT21	nudix hydrolase 21	Multiple_Complex
TC1100011032.hg.1	7.47	8.21	-1.67	0.0008	0.0036	CYB561A3	cytochrome b561 family, member A3	Multiple_Complex
TC0100008697.hg.1	6.32	5.76	1.47	0.0008	0.0036	CTH	cystathionine gamma-lyase	Multiple_Complex
TC1900006852.hg.1	5.36	4.83	1.44	0.0008	0.0036	RETN	resistin	Coding
TC0700012190.hg.1	5.46	4.83	1.55	0.0008	0.0036	CCDC71L	coiled-coil domain containing 71-like	Multiple_Complex
TC0100012816.hg.1	7.52	8.23	-1.63	0.0008	0.0037	CLSTN1	calsyntenin 1	Multiple_Complex
TC1200011393.hg.1	5.58	4.95	1.55	0.0008	0.0037	PPFIA2	protein tyrosine phosphatase, receptor type, f	Multiple_Complex
TC1200012111.hg.1	12.3	11.27	2.04	0.0008	0.0037	TAOK3	TAO kinase 3	Multiple_Complex
TC0300011167.hg.1	5.19	4.65	1.45	0.0008	0.0037	BAP1	BRCA1 associated protein 1	Multiple_Complex
TC1700009492.hg.1	5.85	5.25	1.51	0.0008	0.0037	CAMKK1	calcium/calmodulin-dependent protein kinase	Multiple_Complex
TC1700010693.hg.1	9.21	9.99	-1.72	0.0008	0.0037	ACLY	ATP citrate lyase	Multiple_Complex
TC0500007604.hg.1	9.72	10.4	-1.6	0.0008	0.0037	PPWD1	peptidylprolyl isomerase domain and WD repe	Multiple_Complex
TC0600007599.hg.1	9.21	10.11	-1.86	0.0008	0.0037	PRRC2A	proline-rich coiled-coil 2A	Multiple_Complex
TC0800008032.hg.1	5.11	5.74	-1.54	0.0009	0.0037	ZC2HC1A	zinc finger, C2HC-type containing 1A	Multiple_Complex
TC1400009355.hg.1	5.02	5.68	-1.59	0.0009	0.0037	TRMT5	tRNA methyltransferase 5	Multiple_Complex
TC1600007464.hg.1	4.66	4.12	1.45	0.0009	0.0037	MYLPF	myosin light chain, phosphorylatable, fast ske	Multiple_Complex
TC1700012345.hg.1	8.52	9.51	-1.99	0.0009	0.0037	CTDNEP1	CTD nuclear envelope phosphatase 1	Multiple_Complex
TC1000010367.hg.1	5.09	5.7	-1.53	0.0009	0.0037	ZNF25	zinc finger protein 25	Multiple_Complex
TC1200011741.hg.1	5.62	6.54	-1.89	0.0009	0.0037	C12orf42	chromosome 12 open reading frame 42	Multiple_Complex
TC0900012119.hg.1	5.76	6.23	-1.39	0.0009	0.0037	GALT	galactose-1-phosphate uridylyltransferase	Multiple_Complex
TC1000012389.hg.1	9.1	8.02	2.11	0.0009	0.0037	ADAM8	ADAM metallopeptidase domain 8	Multiple_Complex
TC1300007590.hg.1	10.17	11.1	-1.9	0.0009	0.0037	RBM26	Memczak2013 ANTISENSE, CDS, coding, INTEF	NonCoding
TC0200016475.hg.1	6.64	7.05	-1.33	0.0009	0.0037	INO80B	INO80 complex subunit B	Multiple_Complex
TC1100007780.hg.1	4.48	3.99	1.41	0.0009	0.0037	MS4A10	membrane-spanning 4-domains, subfamily A,	Coding
TC0X00008874.hg.1	11.27	11.85	-1.5	0.0009	0.0037	F8A2	coagulation factor VIII-associated 2	Coding
TC1700012215.hg.1	7.25	8.18	-1.91	0.0009	0.0037	KSR1	kinase suppressor of ras 1	Multiple_Complex
TC1100006477.hg.1	7.09	7.74	-1.57	0.0009	0.0038	PHRF1	PHD and ring finger domains 1	Multiple_Complex
TC0100018483.hg.1	11.79	10.65	2.21	0.0009	0.0038	HIST2H2BE	histone cluster 2, H2be	Coding
TC0400011253.hg.1	8.13	7.28	1.8	0.0009	0.0038	KLHL8	kelch-like family member 8	Multiple_Complex
TC0500011875.hg.1	5.49	4.76	1.65	0.0009	0.0038	PPIC	peptidylprolyl isomerase C (cyclophilin C)	Multiple_Complex
TC1800009222.hg.1	7.32	8.78	-2.74	0.0009	0.0038	RNF125	ring finger protein 125, E3 ubiquitin protein lig	Multiple_Complex
TC0100017828.hg.1	5.65	6.19	-1.45	0.0009	0.0038	B3GALNT2	beta-1,3-N-acetylgalactosaminyltransferase 2	Multiple_Complex
TC0100009943.hg.1	9.94	9.13	1.76	0.0009	0.0038	SCNM1; TN	sodium channel modifier 1; tumor necrosis fac	Multiple_Complex
TC0X00011168.hg.1	11.28	10.58	1.63	0.0009	0.0038	BCAP31	B-cell receptor-associated protein 31	Multiple_Complex
TC0200013541.hg.1	4.83	5.16	-1.25	0.0009	0.0038	SEMA4C	sema domain, immunoglobulin domain (Ig), tr	Multiple_Complex
TC1000012472.hg.1	8.84	8.13	1.64	0.0009	0.0038	FAM25A	family with sequence similarity 25, member A	Coding

TC1400010649.hg.1	6.95	6.24	1.63	0.0009	0.0038	VRK1	vaccinia related kinase 1	Multiple_Complex
TC0100013076.hg.1	10.55	8.44	4.32	0.0009	0.0038	PADI2	peptidyl arginine deiminase, type II	Multiple_Complex
TC0100011621.hg.1	5.92	5.24	1.61	0.0009	0.0038	TGFB2; TGF	transforming growth factor beta 2; TGFB2 ove	Multiple_Complex
TC0800006880.hg.1	9.44	10.49	-2.07	0.0009	0.0038	PCM1	pericentriolar material 1	Multiple_Complex
TC2200006681.hg.1	12.14	12.81	-1.59	0.0009	0.0038	CRKL	v-crk avian sarcoma virus CT10 oncogene hon	Multiple_Complex
TC1500007975.hg.1	8.73	9.73	-2	0.0009	0.0038	IREB2	iron responsive element binding protein 2	Multiple_Complex
TC0X00009842.hg.1	4.19	4.77	-1.5	0.0009	0.0038	SPIN2A	spindlin family, member 2A	Coding
TC1900008464.hg.1	3.84	3.54	1.23	0.0009	0.0038	C19orf68	chromosome 19 open reading frame 68	Multiple_Complex
TC0200009687.hg.1	9.14	9.62	-1.39	0.0009	0.0038	ARL6IP6	ADP-ribosylation factor like GTPase 6 interacti	Multiple_Complex
TC1700006652.hg.1	5.34	4.94	1.32	0.0009	0.0038	C17orf107	chromosome 17 open reading frame 107	Multiple_Complex
TC1900007297.hg.1	4.53	4.85	-1.25	0.0009	0.0038	SIN3B	SIN3 transcription regulator family member B	Multiple_Complex
TC1400009690.hg.1	5.2	5.71	-1.42	0.0009	0.0038	ABCD4	ATP binding cassette subfamily D member 4	Multiple_Complex
TC0600011233.hg.1	7.54	8.32	-1.72	0.0009	0.0039	HIST1H3I	histone cluster 1, H3i	Coding
TC0200016637.hg.1	6.96	7.7	-1.66	0.0009	0.0039	SMC6	structural maintenance of chromosomes 6	Multiple_Complex
TC1900009493.hg.1	4.91	4.47	1.36	0.0009	0.0039	CD209	CD209 molecule	Multiple_Complex
TC1700010358.hg.1	5.84	6.36	-1.44	0.0009	0.0039	MYO1D	myosin ID	Multiple_Complex
TC1600010864.hg.1	6.58	7.2	-1.54	0.0009	0.0039	WDR59	WD repeat domain 59	Multiple_Complex
TSUnmapped00000445.h	9.46	9.97	-1.43	0.0009	0.0039	FBL	fibrillarin	Coding
TC1900011573.hg.1	3.26	3.78	-1.43	0.0009	0.0039	ZNF416	zinc finger protein 416	Coding
TC1900011682.hg.1	14.49	13.75	1.67	0.0009	0.0039	RAB8A	RAB8A, member RAS oncogene family	Multiple_Complex
TC0500013219.hg.1	8.18	8.86	-1.61	0.0009	0.0039	HSD17B4	hydroxysteroid (17-beta) dehydrogenase 4	Multiple_Complex
TC1200006850.hg.1	10.92	11.88	-1.94	0.0009	0.0039	ETV6	ets variant 6	Multiple_Complex
TC0800008550.hg.1	8.72	9.55	-1.77	0.0009	0.0039	EBAG9	estrogen receptor binding site associated, ant	Multiple_Complex
TC1200008255.hg.1	7.67	6.51	2.23	0.0009	0.0039	ZDHHC17	zinc finger, DHHC-type containing 17	Multiple_Complex
TC1000011660.hg.1	7.47	8.57	-2.15	0.0009	0.0039	MGEA5	Salzman2013 ALT_ACCEPTOR, ALT_DONOR, co	NonCoding
TC0900007221.hg.1	5.62	6.13	-1.42	0.0009	0.0039	LOC102724	uncharacterized LOC102724238; uncharacteri	Multiple_Complex
TC0100010233.hg.1	4.1	3.58	1.44	0.0009	0.0039	OR10K1	olfactory receptor, family 10, subfamily K, me	Coding
TC1100012702.hg.1	5.38	6.27	-1.86	0.0009	0.004	TMEM218	transmembrane protein 218	Multiple_Complex
TC2100008537.hg.1	6.02	6.63	-1.54	0.0009	0.004	C21orf33	chromosome 21 open reading frame 33	Multiple_Complex
TC1100011605.hg.1	5.2	5.75	-1.46	0.0009	0.004	UCP3	uncoupling protein 3 (mitochondrial, proton c	Multiple_Complex
TC0900007520.hg.1	7.41	8.02	-1.53	0.0009	0.004	SMC5	structural maintenance of chromosomes 5	Multiple_Complex
TC0100010553.hg.1	5.68	6.42	-1.67	0.0009	0.004	C1orf112	chromosome 1 open reading frame 112	Multiple_Complex
TC0500008736.hg.1	9.4	11.11	-3.26	0.0009	0.004	TGFBI	transforming growth factor, beta-induced, 68I	Multiple_Complex
TC0900007618.hg.1	4.63	5.47	-1.79	0.0009	0.004	PCSK5	proprotein convertase subtilisin/kexin type 5	Multiple_Complex
TC0100017945.hg.1	6.17	6.94	-1.7	0.0009	0.004	FH	fumarate hydratase	Multiple_Complex
TC1400010704.hg.1	6.8	7.68	-1.84	0.0009	0.004	CRIP1	cysteine-rich protein 1 (intestinal)	Multiple_Complex
TC1100010732.hg.1	5.16	4.63	1.45	0.0009	0.004	MYBPC3	myosin binding protein C, cardiac	Multiple_Complex
TC0500010199.hg.1	4.32	5.34	-2.03	0.0009	0.004	FAM134B	family with sequence similarity 134, member	Multiple_Complex
TC0800012066.hg.1	3.91	4.52	-1.53	0.0009	0.004	TSNARE1	t-SNARE domain containing 1	Multiple_Complex
TC1900012049.hg.1	5.45	6.28	-1.77	0.0009	0.004	TMEM86B	transmembrane protein 86B	Multiple_Complex
TC2200007576.hg.1	9.27	10.16	-1.85	0.0009	0.004	PARVG	parvin, gamma	Multiple_Complex
TC0100010606.hg.1	5.92	6.79	-1.83	0.0009	0.004	METTL13	methyltransferase like 13	Multiple_Complex
TC0600013165.hg.1	5.78	6.49	-1.63	0.0009	0.004	EPB41L2	erythrocyte membrane protein band 4.1-like 2	Multiple_Complex
TC0100007898.hg.1	9.91	10.81	-1.87	0.0009	0.004	MACF1; KIA	microtubule-actin crosslinking factor 1; KIAA0	Multiple_Complex
TC0200013109.hg.1	5.73	6.46	-1.66	0.0009	0.004	PCGF1	polycomb group ring finger 1	Multiple_Complex
TC0500013288.hg.1	5.35	4.82	1.45	0.0009	0.004	TAS2R1	taste receptor, type 2, member 1	Coding
TC1000006859.hg.1	4.6	5.28	-1.6	0.0009	0.004	SUV39H2	suppressor of variegation 3-9 homolog 2 (Dros	Multiple_Complex
TC1000011885.hg.1	5.29	6	-1.63	0.0009	0.004	DCLRE1A	DNA cross-link repair 1A	Multiple_Complex
TC0X00008886.hg.1	4.95	5.51	-1.47	0.0009	0.004	GTPBP6	GTP binding protein 6 (putative)	Multiple_Complex
TC0100012870.hg.1	6.89	7.48	-1.51	0.0009	0.004	EXOSC10	exosome component 10	Multiple_Complex
TC1600009966.hg.1	5.71	6.6	-1.85	0.0009	0.004	DCTPP1	dCTP pyrophosphatase 1	Multiple_Complex
TC2200006827.hg.1	8.05	6.73	2.51	0.0009	0.004	GNAZ	guanine nucleotide binding protein (G protein	Multiple_Complex
TC2200007987.hg.1	6.09	5.74	1.27	0.0009	0.004	CLDN5	claudin 5	Coding
TC0700012111.hg.1	5.13	4.14	1.99	0.0009	0.004	FBXL13	F-box and leucine-rich repeat protein 13	Multiple_Complex
TSUnmapped00000138.h	6.54	7.36	-1.77	0.0009	0.004	HYOU1	hypoxia up-regulated 1	Coding
TC0100013417.hg.1	8.25	7.37	1.84	0.0009	0.0041	SLC9A1	solute carrier family 9, subfamily A (NHE1, cat	Multiple_Complex
TC0300013684.hg.1	8.85	8.08	1.71	0.0009	0.0041	TFRC	transferrin receptor	Multiple_Complex
TC1400010775.hg.1	4.83	4.08	1.69	0.0009	0.0041	EFCAB11	EF-hand calcium binding domain 11	Multiple_Complex
TC1000010256.hg.1	10.1	10.57	-1.39	0.001	0.0041	EPC1	enhancer of polycomb homolog 1 (Drosophila	Multiple_Complex
TC0300007236.hg.1	4.39	4.98	-1.5	0.001	0.0041	LARS2	leucyl-tRNA synthetase 2, mitochondrial	Multiple_Complex
TC0100011211.hg.1	5.08	4.63	1.37	0.001	0.0041	GPR37L1	G protein-coupled receptor 37 like 1	Multiple_Complex
TC0800011634.hg.1	7.96	8.41	-1.37	0.001	0.0041	SNTB1	syntrophin, beta 1 (dystrophin-associated pro	Multiple_Complex
TC1800009234.hg.1	7.96	8.41	-1.37	0.001	0.0041	RP11-35G9	Salzman2013 ANTISENSE, coding, INTERNAL, i	NonCoding
TC1200008026.hg.1	9.33	9.87	-1.46	0.001	0.0041	LEMD3	LEM domain containing 3	Multiple_Complex
TC0100013458.hg.1	5.87	6.46	-1.5	0.001	0.0041	EYA3	EYA transcriptional coactivator and phosphata	Multiple_Complex
TC0900009043.hg.1	8.95	7.93	2.02	0.001	0.0041	GFI1B	growth factor independent 1B transcription re	Multiple_Complex
TC0700009518.hg.1	3.81	3.37	1.36	0.001	0.0041	OR2A5	olfactory receptor, family 2, subfamily A, mem	Coding
TC1900012020.hg.1	5.73	6.27	-1.46	0.001	0.0041	ZNF432	zinc finger protein 432	Multiple_Complex
TC1200009248.hg.1	5.8	6.32	-1.44	0.001	0.0041	DDX55	DEAD (Asp-Glu-Ala-Asp) box polypeptide 55	Multiple_Complex
TC1700010202.hg.1	8.75	9.56	-1.75	0.001	0.0041	KIAA0100	KIAA0100	Multiple_Complex

TC1400010779.hg.1	5.6	6.11	-1.42	0.001	0.0041	ATXN3	ataxin 3	NonCoding
TC1800007446.hg.1	6.55	5.95	1.51	0.001	0.0041	SEC11C	SEC11 homolog C, signal peptidase complex su	Multiple_Complex
TC1000009691.hg.1	6.01	6.8	-1.73	0.001	0.0041	IL2RA	interleukin 2 receptor, alpha	Coding
TC1100007812.hg.1	4.03	3.58	1.36	0.001	0.0041	LRRC10B	leucine rich repeat containing 10B	Coding
TC0500007349.hg.1	4.57	4.07	1.42	0.001	0.0041	ISL1	ISL LIM homeobox 1	Multiple_Complex
TC1300009013.hg.1	7.74	8.13	-1.32	0.001	0.0042	VPS36	vacuolar protein sorting 36 homolog (S. cerevi	Multiple_Complex
TC0600011615.hg.1	6.84	6.44	1.33	0.001	0.0042	DEF6	Memczak2013 ANTISENSE, CDS, coding, INTEF	NonCoding
TC0300008705.hg.1	10.35	8.98	2.58	0.001	0.0042	ABTB1	ankyrin repeat and BTB (POZ) domain contain	Multiple_Complex
TC0300013336.hg.1	6.46	7.91	-2.73	0.001	0.0042	LAMP3	lysosomal-associated membrane protein 3	Multiple_Complex
TC0800009782.hg.1	8.57	7.22	2.56	0.001	0.0042	CSGALNAC	chondroitin sulfate N-acetylgalactosaminyltrar	Multiple_Complex
TC1500009182.hg.1	8.39	9.06	-1.59	0.001	0.0042	VPS39	vacuolar protein sorting 39 homolog (S. cerevi	Multiple_Complex
TC0300006552.hg.1	11.59	10.94	1.58	0.001	0.0042	MTMR14	myotubularin related protein 14	Multiple_Complex
TSUnmapped00000272.h	3.9	4.46	-1.48	0.001	0.0042	ZKSCAN7	zinc finger with KRAB and SCAN domains 7	NonCoding
TSUnmapped00000504.h	4.5	5.16	-1.57	0.001	0.0042	ZNF197	zinc finger protein 197	Coding
TC0800007787.hg.1	5.1	4.57	1.44	0.001	0.0042	CLVS1	clavesin 1	Multiple_Complex
TC1700010709.hg.1	6.25	5.5	1.68	0.001	0.0042	KAT2A	Memczak2013 ALT_DONOR, coding, INTERNA	NonCoding
TSUnmapped00000477.h	9.95	10.71	-1.69	0.001	0.0042	SURF4	surfeit 4	Coding
TC0900012234.hg.1	7.94	8.59	-1.58	0.001	0.0042	CBWD6; CE	COBW domain containing 6; COBW domain cc	Multiple_Complex
TC0500013381.hg.1	5.34	4.75	1.51	0.001	0.0042	PRELID2	PRELI domain containing 2	Multiple_Complex
TC0100007512.hg.1	4.5	4.09	1.33	0.001	0.0042	GPR3	G protein-coupled receptor 3	Coding
TC0X00008831.hg.1	9.86	9.03	1.78	0.001	0.0042	ATP6AP1	ATPase, H+ transporting, lysosomal accessory	Multiple_Complex
TC1000007132.hg.1	5.13	4.51	1.54	0.001	0.0042	BAMBI	BMP and activin membrane-bound inhibitor	Multiple_Complex
TC1600010347.hg.1	4.48	5.38	-1.86	0.001	0.0042	RPGRIP1L	RPGRIP1-like	Multiple_Complex
TC1000008881.hg.1	10.97	12.21	-2.36	0.001	0.0042	MXI1	MAX interactor 1, dimerization protein	Multiple_Complex
TC0500009012.hg.1	7.8	6.98	1.76	0.001	0.0043	TCERG1	Transcript Identified by AceView, Entrez Gene	Unassigned
TC1900008112.hg.1	10.02	9.22	1.74	0.001	0.0043	SHKBP1	SH3KBP1 binding protein 1	Multiple_Complex
TC1500010726.hg.1	7.46	8.32	-1.81	0.001	0.0043	TMEM62	transmembrane protein 62	Multiple_Complex
TC0500007231.hg.1	9.43	10.05	-1.53	0.001	0.0043	PTGER4	prostaglandin E receptor 4 (subtype EP4)	Multiple_Complex
TC1900010765.hg.1	4.15	3.56	1.51	0.001	0.0043	CEACAM7	carcinoembryonic antigen-related cell adhesic	Multiple_Complex
TC0800010299.hg.1	4.64	5.42	-1.71	0.001	0.0043	RNF170; M	ring finger protein 170; microRNA 4469	Multiple_Complex
TC1700009392.hg.1	5.11	4.53	1.49	0.001	0.0043	SCARF1	scavenger receptor class F, member 1	Multiple_Complex
TC0100010332.hg.1	7.42	8.1	-1.6	0.001	0.0043	NIT1	nitrilase 1	Multiple_Complex
TC0600011368.hg.1	4.5	3.88	1.53	0.001	0.0043	GNL1	guanine nucleotide binding protein-like 1	Multiple_Complex
TC0600008486.hg.1	14.26	15.3	-2.06	0.001	0.0043	OGFRL1	opioid growth factor receptor-like 1	Multiple_Complex
TC0100017059.hg.1	7.16	8.13	-1.96	0.001	0.0043	RBBP5	retinoblastoma binding protein 5	Multiple_Complex
TC0100017538.hg.1	6.22	6.98	-1.69	0.001	0.0043	ITPKB	Transcript Identified by AceView, Entrez Gene	Coding
TC1700011088.hg.1	4.79	4.36	1.34	0.001	0.0043	COL1A1	collagen, type I, alpha 1	Multiple_Complex
TC1600010450.hg.1	4.75	5.38	-1.55	0.001	0.0043	CIAPIN1	cytokine induced apoptosis inhibitor 1	Multiple_Complex
TC0100012872.hg.1	7.03	7.67	-1.56	0.001	0.0043	MTOR	mechanistic target of rapamycin (serine/threc	Multiple_Complex
TC1400007395.hg.1	4.57	3.96	1.53	0.001	0.0043	SYT16	synaptotagmin XVI	Multiple_Complex
TC0100009020.hg.1	4.4	5.11	-1.63	0.001	0.0043	CDC7	cell division cycle 7	Multiple_Complex
TC1900011845.hg.1	5.3	5.9	-1.52	0.001	0.0043	MFSD12	major facilitator superfamily domain containir	Multiple_Complex
TC0300014090.hg.1	10.06	9.41	1.57	0.001	0.0043	ATP13A3	ATPase type 13A3	Multiple_Complex
TC0300009713.hg.1	4.41	4.05	1.29	0.001	0.0043	EPHB3	EPH receptor B3	Multiple_Complex
TC1000007625.hg.1	4.27	3.97	1.23	0.001	0.0043	PRKG1	protein kinase, cGMP-dependent, type I	Multiple_Complex
TC0200012288.hg.1	8.17	7.21	1.94	0.001	0.0044	HNRNPLL	heterogeneous nuclear ribonucleoprotein L-lik	Multiple_Complex
TC0700013512.hg.1	10.18	9.65	1.44	0.001	0.0044	CCZ1B	CCZ1 homolog B, vacuolar protein trafficking a	Multiple_Complex
TC0800009437.hg.1	15.41	10.9	22.73	0.001	0.0044	DEFA1B; DI	defensin, alpha 1B; defensin, alpha 1; defensir	Coding
TC0800012381.hg.1	15.41	10.9	22.73	0.001	0.0044	DEFA1; DEI	defensin, alpha 1; defensin, alpha 1B	Coding
TC1900007397.hg.1	15.34	16.05	-1.63	0.001	0.0044	UBA52	ubiquitin A-52 residue ribosomal protein fusic	Multiple_Complex
TC0600007263.hg.1	5.66	4.84	1.77	0.001	0.0044	HIST1H4A	histone cluster 1, H4a	Coding
TC0700013575.hg.1	9.7	10.08	-1.3	0.001	0.0044	STAG3L3; S	stromal antigen 3-like 3 (pseudogene); stroma	Multiple_Complex
TC1600009199.hg.1	6.4	7.02	-1.54	0.001	0.0044	SLX4	SLX4 structure-specific endonuclease subunit	Multiple_Complex
TC0100018137.hg.1	3.93	3.37	1.48	0.001	0.0044	OR14I1	olfactory receptor, family 14, subfamily I, mer	Coding
TC0300006483.hg.1	11.27	10.66	1.53	0.001	0.0044	BHLHE40	basic helix-loop-helix family, member e40	Multiple_Complex
TC0700008427.hg.1	4.66	4.21	1.37	0.001	0.0044	DLX6	distal-less homeobox 6	Multiple_Complex
TC0400007460.hg.1	6.81	7.6	-1.72	0.001	0.0044	OCIAD1	Transcript Identified by AceView, Entrez Gene	Unassigned
TC0500012539.hg.1	6.94	6.19	1.69	0.001	0.0044	FAM114A2	family with sequence similarity 114, member .	Multiple_Complex
TC0200014765.hg.1	4.63	5.49	-1.81	0.0011	0.0044	DPP4	Transcript Identified by AceView, Entrez Gene	Unassigned
TC1900009100.hg.1	6.49	7.08	-1.5	0.0011	0.0044	POLRMT	polymerase (RNA) mitochondrial (DNA directe	Multiple_Complex
TC0400009935.hg.1	4.62	4.14	1.39	0.0011	0.0045	CCDC96	coiled-coil domain containing 96	Coding
TC1600010623.hg.1	7.11	6.58	1.43	0.0011	0.0045	FHOD1	formin homology 2 domain containing 1	Multiple_Complex
TC1700008984.hg.1	14.92	13.98	1.91	0.0011	0.0045	SEC14L1; S	SEC14-like lipid binding 1; small Cajal body-sp	Multiple_Complex
TC0200008646.hg.1	8.48	9.36	-1.83	0.0011	0.0045	CNOT11	CCR4-NOT transcription complex subunit 11	Multiple_Complex
TC0600012597.hg.1	6.47	5.71	1.69	0.0011	0.0045	EPHA7	EPH receptor A7	Multiple_Complex
TC1900006468.hg.1	4.94	5.43	-1.4	0.0011	0.0045	GZMM	granzyme M	Coding
TC1300006543.hg.1	7.05	7.92	-1.83	0.0011	0.0045	FGF9	fibroblast growth factor 9	Multiple_Complex
TC0100013371.hg.1	6.74	7.32	-1.49	0.0011	0.0045	PAFAH2	platelet-activating factor acetylhydrolase 2	Multiple_Complex
TC1000010101.hg.1	13.01	12.42	1.51	0.0011	0.0045	ABI1	abl-interactor 1	Multiple_Complex
TC0900011992.hg.1	7.12	6.4	1.64	0.0011	0.0045	AGPAT2	1-acylglycerol-3-phosphate O-acyltransferase	Multiple_Complex

TC0400006689.hg.1	7.76	8.61	-1.81	0.0011	0.0045	MAN2B2	mannosidase, alpha, class 2B, member 2	Multiple_Complex
TC0300008514.hg.1	8.53	9.28	-1.69	0.0011	0.0045	NDUFB4	NADH dehydrogenase (ubiquinone) 1 beta sub	Multiple_Complex
TC1100006487.hg.1	12	10.8	2.29	0.0011	0.0045	TALDO1	transaldolase 1	Multiple_Complex
TC0200007096.hg.1	10.18	9.49	1.61	0.0011	0.0045	FOSL2	FOS-like antigen 2	Multiple_Complex
TC0200014260.hg.1	6.59	6.95	-1.28	0.0011	0.0045	CCDC115	coiled-coil domain containing 115	Multiple_Complex
TSUnmapped00000538.h	9.3	7.7	3.02	0.0011	0.0045	DGKD	diacylglycerol kinase, delta 130kDa	NonCoding
TC0300011436.hg.1	5.45	6	-1.46	0.0011	0.0045	SUCLG2	succinate-CoA ligase, GDP-forming, beta subu	Multiple_Complex
TC0100010159.hg.1	7.45	8.57	-2.18	0.0011	0.0045	SLC25A44	solute carrier family 25, member 44	Multiple_Complex
TC1900008750.hg.1	9.37	9.84	-1.38	0.0011	0.0045	ZNF761; TP	zinc finger protein 761; tropomyosin 3 pseudo	Multiple_Complex
TC0900009779.hg.1	10.57	9.76	1.76	0.0011	0.0046	C9orf72	chromosome 9 open reading frame 72	Multiple_Complex
TC0400008994.hg.1	4.12	3.52	1.52	0.0011	0.0046	PRSS48	protease, serine, 48	Coding
TC0300011110.hg.1	4.91	5.35	-1.35	0.0011	0.0046	CACNA2D2	calcium channel, voltage-dependent, alpha 2/	Multiple_Complex
TC1300008879.hg.1	8.88	8.29	1.51	0.0011	0.0046	MED4	mediator complex subunit 4	Multiple_Complex
TSUnmapped00000427.h	7.42	8	-1.49	0.0011	0.0046	FBL	fibrillarin	Coding
TC0300012377.hg.1	7.77	6.76	2	0.0011	0.0046	RAB43	Memczak2013 ALT_ACCEPTOR, ALT_DONOR,	NonCoding
TC1900011004.hg.1	6.46	7.23	-1.71	0.0011	0.0046	SLC1A5	solute carrier family 1 (neutral amino acid trar	Multiple_Complex
TC0900012195.hg.1	3.67	3.18	1.4	0.0011	0.0046	LCN12	lipocalin 12	Multiple_Complex
TC1200008474.hg.1	6.11	6.63	-1.44	0.0011	0.0046	SOCS2	suppressor of cytokine signaling 2	Coding
TSUnmapped00000286.h	5.1	5.71	-1.53	0.0011	0.0046	HMBS	hydroxymethylbilane synthase	Coding
TC0100013811.hg.1	5.21	5.65	-1.36	0.0011	0.0046	MYCL	v-myc avian myelocytomatosis viral oncogene	Coding
TC0100018424.hg.1	11.13	11.76	-1.54	0.0011	0.0046	HNRNPR	heterogeneous nuclear ribonucleoprotein R	Multiple_Complex
TC0300007051.hg.1	5.12	4.45	1.59	0.0011	0.0046	ITGA9	integrin alpha 9	Multiple_Complex
TC0300013955.hg.1	4.62	3.89	1.66	0.0011	0.0046	ZNF385D	zinc finger protein 385D	Multiple_Complex
TC1900007955.hg.1	4.55	5.1	-1.47	0.0011	0.0046	ZNF345	zinc finger protein 345	Multiple_Complex
TC0300014085.hg.1	6.36	7.21	-1.81	0.0011	0.0046	TBCCD1	TBCC domain containing 1	Multiple_Complex
TC1900011459.hg.1	4.61	5.2	-1.51	0.0011	0.0046	HSPBP1	HSPA (heat shock 70kDa) binding protein, cyto	Multiple_Complex
TC0300011448.hg.1	6.49	7.29	-1.74	0.0011	0.0046	EOGT	EGF domain-specific O-linked N-acetylglucosa	Multiple_Complex
TC1400007761.hg.1	9.74	10.46	-1.65	0.0011	0.0047	AHSA1	AHA1, activator of heat shock 90kDa protein A	Multiple_Complex
TC0100018185.hg.1	10.6	9.79	1.76	0.0011	0.0047	MINOS1	mitochondrial inner membrane organizing sys	Multiple_Complex
TC1900008482.hg.1	6.08	6.65	-1.49	0.0011	0.0047	SPHK2	sphingosine kinase 2	Multiple_Complex
TC1900010037.hg.1	5.52	6.26	-1.67	0.0011	0.0047	SUGP2	SURP and G-patch domain containing 2	Multiple_Complex
TC1300007187.hg.1	7.79	8.47	-1.6	0.0011	0.0047	TRIM13; KC	tripartite motif containing 13; potassium chan	Multiple_Complex
TC0200014250.hg.1	6.42	7.16	-1.67	0.0011	0.0047	SMPD4	sphingomyelin phosphodiesterase 4, neutral r	Multiple_Complex
TC0200010636.hg.1	5.33	4.62	1.63	0.0011	0.0047	MAP2	microtubule associated protein 2	Multiple_Complex
TC1500007034.hg.1	13.27	12.49	1.72	0.0011	0.0047	SNAP23	synaptosome associated protein 23kDa	Multiple_Complex
TC0700007398.hg.1	6.17	7.1	-1.92	0.0011	0.0047	ZMIZ2	zinc finger, MIZ-type containing 2	Multiple_Complex
TC1200008677.hg.1	10.98	11.96	-1.97	0.0011	0.0047	RPL18A	ribosomal protein L18a	Multiple_Complex
TC1400009391.hg.1	5.8	6.59	-1.74	0.0011	0.0047	WDR89	WD repeat domain 89	Multiple_Complex
TC0200016740.hg.1	9.03	9.59	-1.47	0.0011	0.0048	ARL5A	ADP-ribosylation factor like GTPase 5A	Multiple_Complex
TC0300009690.hg.1	5.8	6.56	-1.68	0.0011	0.0048	EIF2B5	eukaryotic translation initiation factor 2B, sub	Multiple_Complex
TC0900008869.hg.1	7.67	8.25	-1.5	0.0011	0.0048	ODF2	outer dense fiber of sperm tails 2	Multiple_Complex
TC1000008435.hg.1	5.31	4.59	1.64	0.0011	0.0048	HECTD2	HECT domain containing E3 ubiquitin protein l	Multiple_Complex
TC0500009098.hg.1	4.39	4.01	1.3	0.0011	0.0048	MYOZ3	myozenin 3	Multiple_Complex
TC0700013065.hg.1	3.62	4.28	-1.58	0.0011	0.0048	ABCF2	ATP binding cassette subfamily F member 2	Multiple_Complex
TSUnmapped00000785.h	4.75	5.47	-1.64	0.0011	0.0048	ZNF780B	zinc finger protein 780B	Coding
TC1000009104.hg.1	4.3	3.54	1.7	0.0011	0.0048	PLPP4	phospholipid phosphatase 4	Multiple_Complex
TC1700011697.hg.1	7.96	8.67	-1.64	0.0011	0.0048	CD300LB	CD300 molecule-like family member b	Multiple_Complex
TC1200012707.hg.1	9.12	8.36	1.69	0.0012	0.0048	ALDH2	aldehyde dehydrogenase 2 family (mitochond	Multiple_Complex
TC1100006444.hg.1	9.36	8.62	1.67	0.0012	0.0048	ATHL1	ATH1, acid trehalase-like 1 (yeast)	Multiple_Complex
TC1100007490.hg.1	4.79	5.36	-1.49	0.0012	0.0048	FAM180B	family with sequence similarity 180, member	Coding
TC1600010168.hg.1	6.34	5.39	1.93	0.0012	0.0048	SHCBP1	SHC SH2-domain binding protein 1	Multiple_Complex
TC1300008983.hg.1	8.28	9.05	-1.71	0.0012	0.0048	INTS6	integrator complex subunit 6	Multiple_Complex
TC1900010529.hg.1	4.19	4.92	-1.66	0.0012	0.0048	ZNF529	zinc finger protein 529	Multiple_Complex
TC1500007967.hg.1	8.13	8.87	-1.67	0.0012	0.0048	DNAJA4	DnaJ (Hsp40) homolog, subfamily A, member	Multiple_Complex
TC0900008484.hg.1	7	7.55	-1.46	0.0012	0.0048	PRPF4	pre-mRNA processing factor 4	Multiple_Complex
TC1900011900.hg.1	5.69	6.28	-1.51	0.0012	0.0048	CHERP	calcium homeostasis endoplasmic reticulum p	Multiple_Complex
TC0100011830.hg.1	5.65	6.46	-1.76	0.0012	0.0048	ZNF678	zinc finger protein 678	Multiple_Complex
TC0300009944.hg.1	4.43	4.78	-1.28	0.0012	0.0048	FAM43A	family with sequence similarity 43, member A	Multiple_Complex
TC1900011938.hg.1	6.27	6.92	-1.57	0.0012	0.0048	ZNF573	zinc finger protein 573	Multiple_Complex
TC0700012296.hg.1	8.97	8.27	1.63	0.0012	0.0049	C7orf60	chromosome 7 open reading frame 60	Multiple_Complex
TC0200014610.hg.1	11.79	10.68	2.16	0.0012	0.0049	NMI	N-myc (and STAT) interactor	Multiple_Complex
TC1700009162.hg.1	8.8	9.82	-2.03	0.0012	0.0049	C17orf89	chromosome 17 open reading frame 89	Multiple_Complex
TC0100011263.hg.1	5.02	4.26	1.7	0.0012	0.0049	OPTC	opticin	Coding
TC0100015956.hg.1	5.79	6.26	-1.39	0.0012	0.0049	RUSC1-AS1	RUSC1 antisense RNA 1	Multiple_Complex
TC1000009725.hg.1	7.54	8.23	-1.61	0.0012	0.0049	SFMBT2	Scm-like with four mbt domains 2	Multiple_Complex
TC0200014703.hg.1	4.58	4.12	1.38	0.0012	0.0049	CCDC148	coiled-coil domain containing 148	Multiple_Complex
TC1200012617.hg.1	10.83	9.8	2.04	0.0012	0.0049	FGD4	FYVE, RhoGEF and PH domain containing 4	Multiple_Complex
TC0200010980.hg.1	4.13	3.78	1.28	0.0012	0.0049	CCL20	chemokine (C-C motif) ligand 20	Multiple_Complex
TC0400012458.hg.1	7.41	6.38	2.04	0.0012	0.0049	HPGD	hydroxyprostaglandin dehydrogenase 15-(NAI	Multiple_Complex
TC1200008933.hg.1	6.15	6.94	-1.73	0.0012	0.0049	TPCN1	two pore segment channel 1	Multiple_Complex

TC0100013369.hg.1	6.56	7.44	-1.83	0.0012	0.0049	STMN1; MI	stathmin 1; microRNA 3917	Multiple_Complex
TC0900009279.hg.1	6.34	6.94	-1.52	0.0012	0.0049	TOR4A	torsin family 4, member A	Multiple_Complex
TC0300013923.hg.1	6.33	7.89	-2.94	0.0012	0.0049	IL5RA	interleukin 5 receptor, alpha	Multiple_Complex
TC1400008098.hg.1	8.48	10.68	-4.6	0.0012	0.0049	GLRX5	glutaredoxin 5	Multiple_Complex
TC1100006689.hg.1	4.78	4.08	1.63	0.0012	0.0049	OR51B6	olfactory receptor, family 51, subfamily B, me	Coding
TSUnmapped00000162.h	9.45	10.09	-1.56	0.0012	0.005	FBL	fibrillarin	Coding
TC1500008029.hg.1	6.16	5.23	1.9	0.0012	0.005	FAH	fumarylacetoacetate hydrolase (fumarylaceto	Multiple_Complex
TC2000007064.hg.1	5.36	4.81	1.47	0.0012	0.005	DEFB115	defensin, beta 115	Coding
TC2000009890.hg.1	4.42	5.14	-1.65	0.0012	0.005	ANKEF1	ankyrin repeat and EF-hand domain containin	Multiple_Complex
TC1500009946.hg.1	6.38	7.23	-1.8	0.0012	0.005	MYO9A	myosin IXA	Multiple_Complex
TC1500009773.hg.1	8.3	8.88	-1.49	0.0012	0.005	MTFMT	mitochondrial methionyl-tRNA formyltransfer	Multiple_Complex
TC1200007061.hg.1	5.75	6.71	-1.94	0.0012	0.005	CMAS	cytidine monophosphate N-acetylneuraminic	Multiple_Complex
TC0200016643.hg.1	7.02	7.72	-1.62	0.0012	0.005	DNMT3A	DNA (cytosine-5-)-methyltransferase 3 alpha	Multiple_Complex
TC0700013290.hg.1	5.02	5.68	-1.58	0.0012	0.005	NCAPG2	non-SMC condensin II complex subunit G2	Multiple_Complex
TC0100017365.hg.1	7.46	8.24	-1.71	0.0012	0.005	EPRS	glutamyl-prolyl-tRNA synthetase	Multiple_Complex
TC1600009482.hg.1	9.42	9.86	-1.36	0.0012	0.005	PARN	poly(A)-specific ribonuclease	Multiple_Complex
TC1100013170.hg.1	15.4	16.59	-2.28	0.0012	0.005	EEF1G; MIF	eukaryotic translation elongation factor 1 gam	Multiple_Complex
TC0500010780.hg.1	6.79	6.17	1.54	0.0012	0.005	SLC38A9	Transcript Identified by AceView, Entrez Gene	Unassigned
TC1200012263.hg.1	6.41	7.34	-1.91	0.0012	0.005	MPHOSPH9	M-phase phosphoprotein 9	Multiple_Complex
TC0800009523.hg.1	6.55	7.18	-1.56	0.0012	0.005	MFHAS1	malignant fibrous histiocytoma amplified sequ	Multiple_Complex
TC0600014126.hg.1	8.18	9.05	-1.83	0.0012	0.005	GLTSCR1L	GLTSCR1-like	Multiple_Complex
TC1900009320.hg.1	8.42	9.11	-1.62	0.0012	0.005	ZBTB7A	zinc finger and BTB domain containing 7A	Multiple_Complex
TC2000008130.hg.1	7.32	8.3	-1.98	0.0012	0.005	TBC1D20	TBC1 domain family, member 20	Multiple_Complex
TC1900006530.hg.1	8.52	9.13	-1.53	0.0012	0.0051	DAZAP1	DAZ associated protein 1	Multiple_Complex
TC0X00011290.hg.1	4.39	3.94	1.37	0.0012	0.0051	GAGE1	G antigen 1	Multiple_Complex
TC0100018235.hg.1	5.29	5.82	-1.44	0.0012	0.0051	FPGT-TNNI	FPGT-TNNI3K readthrough; fucose-1-phospha	Multiple_Complex
TC0200016768.hg.1	8.99	8.23	1.69	0.0012	0.0051	PECR	peroxisomal trans-2-enoyl-CoA reductase	Multiple_Complex
TC1700010810.hg.1	10.43	10.91	-1.39	0.0012	0.0051	LSM12	LSM12 homolog	Multiple_Complex
TC0400012901.hg.1	6.67	5.77	1.87	0.0012	0.0051	FBXL5	F-box and leucine-rich repeat protein 5	NonCoding
TC1000010555.hg.1	8.51	9.35	-1.79	0.0012	0.0051	AGAP9; BM	ArfGAP with GTPase domain, ankyrin repeat a	Multiple_Complex
TC1100012959.hg.1	7.39	8.18	-1.73	0.0012	0.0051	TIMM10B	translocase of inner mitochondrial membrane	Multiple_Complex
TC0600007636.hg.1	9.33	9.91	-1.49	0.0012	0.0051	RNF5	ring finger protein 5, E3 ubiquitin protein ligas	Multiple_Complex
TC1900007760.hg.1	6.34	5.77	1.48	0.0012	0.0051	FAAP24	Fanconi anemia core complex associated prote	Multiple_Complex
TC0500007690.hg.1	6.25	5.68	1.48	0.0012	0.0051	SERF1B; SE	small EDRK-rich factor 1B (centromeric); small	Multiple_Complex
TC0500007698.hg.1	6.25	5.68	1.48	0.0012	0.0051	SERF1A	small EDRK-rich factor 1A (telomeric)	Multiple_Complex
TC1700010717.hg.1	11.5	10.56	1.92	0.0012	0.0051	STAT5B	signal transducer and activator of transcriptio	Multiple_Complex
TC0200009883.hg.1	4.94	4.27	1.59	0.0012	0.0051	XIRP2	xin actin binding repeat containing 2	Coding
TC0X00010329.hg.1	4.14	4.82	-1.61	0.0012	0.0051	TSPAN6	tetraspanin 6	Multiple_Complex
TC1200006966.hg.1	10.99	10.39	1.51	0.0012	0.0051	STRAP	serine/threonine kinase receptor associated p	Multiple_Complex
TC1200006649.hg.1	8.7	8.09	1.53	0.0013	0.0051	TPI1	triosephosphate isomerase 1	Multiple_Complex
TC0600011065.hg.1	5.64	5.06	1.49	0.0013	0.0051	KIAA0319	KIAA0319	Coding
TC0400007876.hg.1	6.87	7.37	-1.41	0.0013	0.0051	THAP6	THAP domain containing 6	Multiple_Complex
TC1000008011.hg.1	5.39	4.75	1.55	0.0013	0.0052	OIT3	oncoprotein induced transcript 3	Multiple_Complex
TC0X00011244.hg.1	9.48	10.05	-1.48	0.0013	0.0052	F8A3	coagulation factor VIII-associated 3	Coding
TC0Y00006812.hg.1	4.53	3.99	1.46	0.0013	0.0052	DAZ2; DAZ4	deleted in azoospermia 2; deleted in azoosper	Multiple_Complex
TC1600007485.hg.1	8.55	7.83	1.64	0.0013	0.0052	FBRS	fibrosin	Multiple_Complex
TC0400009944.hg.1	4.37	3.97	1.32	0.0013	0.0052	PSAPL1	prosaposin-like 1 (gene/pseudogene)	Multiple_Complex
TC0100013908.hg.1	8.34	9.19	-1.8	0.0013	0.0052	SLC2A1	solute carrier family 2 (facilitated glucose tran	Multiple_Complex
TC0400008624.hg.1	4.26	4.96	-1.62	0.0013	0.0052	SPATA5	Transcript Identified by AceView, Entrez Gene	Unassigned
TC1600010801.hg.1	4.78	4.24	1.46	0.0013	0.0052	PKD1L3	polycystic kidney disease 1-like 3	Multiple_Complex
TC2200008459.hg.1	5.28	5.78	-1.42	0.0013	0.0052	MORC2	MORC family CW-type zinc finger 2	Multiple_Complex
TC1900008337.hg.1	8.63	8.19	1.36	0.0013	0.0052	EML2-AS1	EML2 antisense RNA 1	Multiple_Complex
TC0600014262.hg.1	8.82	8.25	1.49	0.0013	0.0052	ABHD16A	abhydrolase domain containing 16A	Multiple_Complex
TC1300008834.hg.1	4.34	3.77	1.48	0.0013	0.0052	SIAH3	siah E3 ubiquitin protein ligase family membe	Coding
TC0300007161.hg.1	7.56	8.16	-1.51	0.0013	0.0052	VIPR1	vasoactive intestinal peptide receptor 1	Multiple_Complex
TC0500009861.hg.1	4.72	4.13	1.51	0.0013	0.0053	SLC6A3	solute carrier family 6 (neurotransmitter trans	Multiple_Complex
TC1500009138.hg.1	5.47	6.22	-1.69	0.0013	0.0053	INO80	INO80 complex subunit	Multiple_Complex
TC1400010263.hg.1	7.28	6.73	1.47	0.0013	0.0053	CINP	cyclin-dependent kinase 2 interacting protein	Multiple_Complex
TC0200012129.hg.1	7.39	8.04	-1.57	0.0013	0.0053	TRMT61B	tRNA methyltransferase 61B	Multiple_Complex
TC0700009667.hg.1	5.78	6.42	-1.56	0.0013	0.0053	LRRC61	leucine rich repeat containing 61	Multiple_Complex
TC1700007444.hg.1	7.48	6.65	1.78	0.0013	0.0053	NSRP1; MIF	nuclear speckle splicing regulatory protein 1; r	Multiple_Complex
TC0400010898.hg.1	4.34	3.73	1.53	0.0013	0.0053	TMPRSS11F	transmembrane protease, serine 11F	Coding
TC1400009784.hg.1	8.1	8.79	-1.61	0.0013	0.0053	VIPAS39	VPS33B interacting protein, apical-basolateral	Multiple_Complex
TC1900009188.hg.1	8.4	8.97	-1.48	0.0013	0.0053	REXO1; MII	REX1, RNA exonuclease 1 homolog; microRNA	Multiple_Complex
TC1100007938.hg.1	10.4	9.61	1.72	0.0013	0.0053	FERMT3	fermitin family member 3	Multiple_Complex
TC0200006715.hg.1	7.7	8.25	-1.46	0.0013	0.0053	PQLC3	PQ loop repeat containing 3	Multiple_Complex
TC1200008931.hg.1	5.68	6.36	-1.61	0.0013	0.0053	RITA1	RBPJ interacting and tubulin associated 1	Coding
TC0800012382.hg.1	15.32	10.92	21.16	0.0013	0.0053	DEFA3	defensin, alpha 3, neutrophil-specific	Coding
TC0300011049.hg.1	6.58	5.84	1.67	0.0013	0.0053	QRICH1	Transcript Identified by AceView, Entrez Gene	Unassigned
TC0700008927.hg.1	6.65	6	1.56	0.0013	0.0053	ING3	Transcript Identified by AceView, Entrez Gene	Unassigned

TC0800008352.hg.1	12.92	13.56	-1.55	0.0013	0.0053	VPS13B	vacuolar protein sorting 13 homolog B (yeast)	Multiple_Complex
TC1700009522.hg.1	7.74	8.39	-1.57	0.0013	0.0054	PELP1	proline, glutamate and leucine rich protein 1	Multiple_Complex
TC1100011751.hg.1	4.3	4.87	-1.48	0.0013	0.0054	NARS2	asparaginyl-tRNA synthetase 2, mitochondrial	Multiple_Complex
TC0300010357.hg.1	5.74	6.18	-1.36	0.0013	0.0054	CHCHD4	coiled-coil-helix-coiled-coil-helix domain conta	Multiple_Complex
TC0600011357.hg.1	8.06	8.46	-1.32	0.0013	0.0054	TRIM26	tripartite motif containing 26	Multiple_Complex
TC1500010756.hg.1	4.69	4.11	1.49	0.0013	0.0054	RAB11A	RAB11A, member RAS oncogene family	NonCoding
TC1400010620.hg.1	7.83	6.85	1.97	0.0013	0.0054	SNAPC1	small nuclear RNA activating complex polypep	Multiple_Complex
TC1900011124.hg.1	4.89	5.51	-1.54	0.0013	0.0054	C19orf73	chromosome 19 open reading frame 73	Coding
TC2000008516.hg.1	4.84	5.88	-2.07	0.0013	0.0054	SNX5	Zhang2013 ALT_ACCEPTOR, ALT_DONOR, cod	NonCoding
TC0300014074.hg.1	5.83	6.74	-1.87	0.0013	0.0054	IFT80	intraflagellar transport 80	Multiple_Complex
TC0200010273.hg.1	8.48	7.96	1.43	0.0013	0.0054	NAB1	NGFI-A binding protein 1	Multiple_Complex
TC0100015588.hg.1	6.9	7.42	-1.43	0.0013	0.0054	RNF115	ring finger protein 115	Multiple_Complex
TSUnmapped00000327.h	7.08	7.56	-1.39	0.0013	0.0054	HYOU1	hypoxia up-regulated 1	Coding
TC0100018282.hg.1	7.29	6.57	1.65	0.0013	0.0054	POLR3GL	polymerase (RNA) III (DNA directed) polypepti	Multiple_Complex
TC1100007739.hg.1	4.56	3.99	1.48	0.0013	0.0054	OR4D11	olfactory receptor, family 4, subfamily D, mem	Coding
TC0200013284.hg.1	7.58	8.03	-1.36	0.0013	0.0055	C2orf68	chromosome 2 open reading frame 68	Multiple_Complex
TC0Y00007325.hg.1	4.64	4.26	1.3	0.0013	0.0055	LOC728395	Homo sapiens similar to testis specific protein	Multiple_Complex
TC1700010408.hg.1	5.47	4.77	1.63	0.0013	0.0055	CCT6B	chaperonin containing TCP1, subunit 6B (zeta	Multiple_Complex
TC1800006834.hg.1	6.95	6.33	1.53	0.0013	0.0055	SNRPD1	small nuclear ribonucleoprotein D1 polypeptic	Multiple_Complex
TC1700010314.hg.1	12.37	11.02	2.55	0.0013	0.0055	EVI2A; EVI2	ecotropic viral integration site 2A; ecotropic vi	Coding
TC1200008322.hg.1	6.14	6.84	-1.62	0.0013	0.0055	TMTC2	transmembrane and tetratricopeptide repeat	Multiple_Complex
TC0200010785.hg.1	14.91	16.11	-2.3	0.0013	0.0055	CXCR2	chemokine (C-X-C motif) receptor 2	Multiple_Complex
TC0X00009911.hg.1	5.29	4.65	1.55	0.0014	0.0055	VSIG4	V-set and immunoglobulin domain containing	Coding
TC0300011951.hg.1	4.44	3.69	1.68	0.0014	0.0055	MORC1	MORC family CW-type zinc finger 1	Coding
TC0500013227.hg.1	6.4	7.39	-1.99	0.0014	0.0055	CDC42SE2	CDC42 small effector 2	NonCoding
TC1700008134.hg.1	6.67	6.03	1.55	0.0014	0.0055	TBKBP1	TBK1 binding protein 1	Coding
TC0600007711.hg.1	4.41	5.36	-1.93	0.0014	0.0055	ITPR3	inositol 1,4,5-trisphosphate receptor, type 3	Multiple_Complex
TC0400007848.hg.1	5.35	4.69	1.58	0.0014	0.0055	MTHFD2L	methylenetetrahydrofolate dehydrogenase (N	Multiple_Complex
TC1000011592.hg.1	6.31	7	-1.62	0.0014	0.0055	COX15	cytochrome c oxidase assembly homolog 15 (y	Multiple_Complex
TC2200009366.hg.1	7.43	6.79	1.56	0.0014	0.0055	CHKB-CPT1	CHKB-CPT1B readthrough (NMD candidate)	Multiple_Complex
TC1100013051.hg.1	7.42	8.11	-1.62	0.0014	0.0055	RBM14-RBI	RBM14-RBM4 readthrough	Multiple_Complex
TC1000010976.hg.1	9.17	10	-1.78	0.0014	0.0055	SPOCK2	sparc/osteonectin, cwcv and kazal-like domair	Multiple_Complex
TC0900009333.hg.1	13.79	12.61	2.26	0.0014	0.0055	FAM157B	family with sequence similarity 157, member	Multiple_Complex
TC1700008587.hg.1	5.67	5.17	1.41	0.0014	0.0056	PRR29	proline rich 29	Multiple_Complex
TC0600012123.hg.1	4.29	5.07	-1.71	0.0014	0.0056	DST	dystonin	Multiple_Complex
TC1100012852.hg.1	3.98	4.42	-1.36	0.0014	0.0056	LOC103611	uncharacterized LOC103611081; uncharacteri	Multiple_Complex
TC0100011219.hg.1	7	8.23	-2.35	0.0014	0.0056	PPP1R12B	protein phosphatase 1, regulatory subunit 12E	Multiple_Complex
TC1600007440.hg.1	14.65	14.05	1.51	0.0014	0.0056	ALDOA	aldolase A, fructose-bisphosphate	Multiple_Complex
TC0300013846.hg.1	5.02	4.52	1.42	0.0014	0.0056	GXYLT2	glucoside xylosyltransferase 2	Multiple_Complex
TC1200009183.hg.1	8.22	9.04	-1.77	0.0014	0.0056	SETD1B	SET domain containing 1B	Multiple_Complex
TC0600009059.hg.1	12.32	11.11	2.33	0.0014	0.0056	FOXO3	forkhead box O3	Multiple_Complex
TC1700006684.hg.1	5.86	6.66	-1.74	0.0014	0.0056	MIS12	MIS12 kinetochore complex component	Multiple_Complex
TC0900008312.hg.1	7.56	6.6	1.95	0.0014	0.0056	SLC44A1	solute carrier family 44 (choline transporter),	Multiple_Complex
TC0700011500.hg.1	7.13	6.63	1.41	0.0014	0.0057	STX1A	syntaxin 1A (brain)	Multiple_Complex
TC1000012066.hg.1	6.3	7.07	-1.71	0.0014	0.0057	NSMCE4A	NSE4 homolog A, SMC5-SMC6 complex compo	Multiple_Complex
TC0100008090.hg.1	5.33	5.67	-1.27	0.0014	0.0057	DMAP1	DNA methyltransferase 1 associated protein 1	Multiple_Complex
TC0100016155.hg.1	7.68	7.07	1.53	0.0014	0.0057	USF1	upstream transcription factor 1	Multiple_Complex
TC0200015009.hg.1	12.46	12.92	-1.37	0.0014	0.0057	ATF2	activating transcription factor 2	Multiple_Complex
TSUnmapped00000615.h	7.84	8.53	-1.61	0.0014	0.0057	LRIG1		Coding
TC2000006444.hg.1	4.89	5.41	-1.43	0.0014	0.0057	TRIB3	tribbles pseudokinase 3	Multiple_Complex
TC0100007484.hg.1	15.69	14.81	1.84	0.0014	0.0057	ZDHHC18	zinc finger, DHHC-type containing 18	Multiple_Complex
TC0500013230.hg.1	6.11	6.63	-1.44	0.0014	0.0057	PCBD2	pterin-4 alpha-carbinolamine dehydratase/dir	Multiple_Complex
TC0800008300.hg.1	10.05	9.44	1.53	0.0014	0.0057	CPQ	carboxypeptidase Q	Multiple_Complex
TC0500012975.hg.1	5.95	5.45	1.41	0.0014	0.0057	DBN1	drebrin 1	Multiple_Complex
TC0600009488.hg.1	12.82	11.09	3.3	0.0014	0.0057	RPS12	ribosomal protein S12	Multiple_Complex
TC0500011277.hg.1	4.35	3.75	1.52	0.0014	0.0057	ANKRD34B	ankyrin repeat domain 34B	Coding
TC0700010619.hg.1	6.06	7.03	-1.95	0.0014	0.0057	SCRN1	secernin 1	Multiple_Complex
TC0200007411.hg.1	11.46	10.95	1.43	0.0014	0.0057	PPM1B	protein phosphatase, Mg2+/Mn2+ dependent	Multiple_Complex
TC0700009333.hg.1	7.87	8.47	-1.52	0.0014	0.0057	UBN2	ubinuclein 2	Multiple_Complex
TC0100016548.hg.1	6.73	6.42	1.24	0.0014	0.0057	ABL2	ABL proto-oncogene 2, non-receptor tyrosine	Multiple_Complex
TC1300008025.hg.1	6.83	5.96	1.83	0.0014	0.0057	ING1	inhibitor of growth family member 1	Coding
TC1600009079.hg.1	4.37	3.99	1.3	0.0014	0.0057	NOXO1	NADPH oxidase organizer 1	Multiple_Complex
TC1200010616.hg.1	17.27	17.67	-1.32	0.0014	0.0057	TUBA1B	tubulin, alpha 1b	Multiple_Complex
TC1700010675.hg.1	3.89	3.36	1.45	0.0014	0.0057	KRT13	keratin 13, type I	Multiple_Complex
TC0600007084.hg.1	5.12	4.68	1.36	0.0014	0.0058	RBM24	RNA binding motif protein 24	Multiple_Complex
TC1000007846.hg.1	9.41	10.14	-1.67	0.0014	0.0058	SIRT1	sirtuin 1	Multiple_Complex
TC0200014029.hg.1	6.96	6.37	1.51	0.0014	0.0058	C2orf76	chromosome 2 open reading frame 76	Multiple_Complex
TC0X00008824.hg.1	11.7	12.41	-1.64	0.0014	0.0058	TKTL1	transketolase-like 1	Multiple_Complex
TC0100015891.hg.1	9.61	10.71	-2.14	0.0014	0.0058	CRTC2	CREB regulated transcription coactivator 2	Multiple_Complex
TC1100009175.hg.1	5.76	6.18	-1.34	0.0014	0.0058	CEP164	centrosomal protein 164kDa	Multiple_Complex

TC0500011334.hg.1	8.07	9.34	-2.42	0.0014	0.0058	TMEM167A	Transcript Identified by AceView, Entrez Gene	Unassigned
TC1100009398.hg.1	9.3	7.98	2.49	0.0014	0.0058	NRGN	neurogranin (protein kinase C substrate, RC3)	Multiple_Complex
TC1900011469.hg.1	5.28	5.82	-1.45	0.0014	0.0058	TMEM238	transmembrane protein 238	Multiple_Complex
TC0300008242.hg.1	7.44	6.78	1.58	0.0014	0.0058	ALCAM	activated leukocyte cell adhesion molecule	Multiple_Complex
TC0600009393.hg.1	8.69	9.29	-1.51	0.0014	0.0058	RNF146	ring finger protein 146	Multiple_Complex
TC2200008169.hg.1	4.2	3.66	1.45	0.0015	0.0058	LOC388882	uncharacterized LOC388882; novel transcript	Multiple_Complex
TC0700009089.hg.1	5.03	4.6	1.34	0.0015	0.0059	STRIP2	striatin interacting protein 2	Multiple_Complex
TC0500007682.hg.1	10.32	11.09	-1.7	0.0015	0.0059	GTF2H2C_2	GTF2H2 family member C, copy 2; GTF2H2 far	Multiple_Complex
TC0200011023.hg.1	11.17	12.27	-2.14	0.0015	0.0059	SP100	SP100 nuclear antigen	Multiple_Complex
TC1200008016.hg.1	4.55	3.82	1.66	0.0015	0.0059	TBC1D30	TBC1 domain family, member 30	Multiple_Complex
TC1600006979.hg.1	5.53	6.37	-1.79	0.0015	0.0059	MKL2; TVP2	MKL/myocardin-like 2; TVP23C pseudogene 2	Multiple_Complex
TC1900009102.hg.1	5.31	6.03	-1.64	0.0015	0.0059	RNF126	ring finger protein 126	Multiple_Complex
TC1200007816.hg.1	6.94	7.52	-1.5	0.0015	0.0059	DGKA	diacylglycerol kinase alpha	Multiple_Complex
TC0100015890.hg.1	4.77	5.56	-1.73	0.0015	0.0059	DENND4B	DENN/MADD domain containing 4B	Multiple_Complex
TC0400012755.hg.1	6.36	7.06	-1.62	0.0015	0.0059	ZNF141	zinc finger protein 141	NonCoding
TC0500006507.hg.1	6.19	5.44	1.68	0.0015	0.0059	LPCAT1	Memczak2013 ANTISENSE, coding, INTERNAL,	NonCoding
TC2000007058.hg.1	6.8	8.03	-2.34	0.0015	0.0059	FRG1BP	FSHD region gene 1 family member B, pseudo	Multiple_Complex
TC0200012936.hg.1	5.25	5.76	-1.43	0.0015	0.0059	FBXO48	F-box protein 48	Coding
TC0200008587.hg.1	8.09	8.79	-1.62	0.0015	0.0059	EIF5B	eukaryotic translation initiation factor 5B	Multiple_Complex
TC1300009856.hg.1	8.06	8.42	-1.29	0.0015	0.0059	TUBGCP3	tubulin, gamma complex associated protein 3	Multiple_Complex
TC1000009414.hg.1	8.88	8.03	1.81	0.0015	0.0059	INPP5A	inositol polyphosphate-5-phosphatase A	Multiple_Complex
TC1700011260.hg.1	7.5	8.27	-1.7	0.0015	0.0059	MTMR4	myotubularin related protein 4	Multiple_Complex
TC0100011619.hg.1	6.88	7.36	-1.4	0.0015	0.006	RRP15	ribosomal RNA processing 15 homolog	Multiple_Complex
TC0900012277.hg.1	5.48	5.96	-1.4	0.0015	0.006	AK1	adenylate kinase 1	Coding
TC0X00008178.hg.1	3.73	3.23	1.41	0.0015	0.006	HTR2C	5-hydroxytryptamine (serotonin) receptor 2C,	Coding
TC0500008654.hg.1	3.99	4.84	-1.8	0.0015	0.006	LEAP2	liver expressed antimicrobial peptide 2	Multiple_Complex
TC1900007891.hg.1	9.54	10.98	-2.71	0.0015	0.006	HCST	hematopoietic cell signal transducer	Multiple_Complex
TC0100006801.hg.1	5.98	5.27	1.64	0.0015	0.006	NMNAT1	nicotinamide nucleotide adenylyltransferase 1	Multiple_Complex
TC1200009103.hg.1	9.29	9.86	-1.48	0.0015	0.006	PRKAB1	protein kinase, AMP-activated, beta 1 non-cat	Multiple_Complex
TC1700007897.hg.1	5.91	6.61	-1.63	0.0015	0.006	HSD17B1	hydroxysteroid (17-beta) dehydrogenase 1	Multiple_Complex
TC1600007575.hg.1	4	3.41	1.5	0.0015	0.006	TP53TG3D	TP53 target 3D	Multiple_Complex
TC2000006694.hg.1	6.46	7.35	-1.86	0.0015	0.006	SLX4IP	SLX4 interacting protein	Multiple_Complex
TC0800012444.hg.1	11.31	12.77	-2.76	0.0015	0.006	PABPC1; M	poly(A) binding protein, cytoplasmic 1; microR	Multiple_Complex
TC0800008478.hg.1	3.51	4.44	-1.91	0.0015	0.006	FZD6	frizzled class receptor 6	Coding
TC1100008139.hg.1	6.64	7.49	-1.8	0.0015	0.006	AIP	aryl hydrocarbon receptor interacting protein	Multiple_Complex
TC1100010254.hg.1	4.95	5.51	-1.48	0.0015	0.006	SAAL1	serum amyloid A-like 1	Multiple_Complex
TC1100008039.hg.1	6.43	7.03	-1.52	0.0015	0.0061	MUS81	MUS81 structure-specific endonuclease subur	Multiple_Complex
TC1200007764.hg.1	8.01	9.13	-2.17	0.0015	0.0061	ZNF385A	Memczak2013 ANTISENSE, CDS, coding, INTEF	NonCoding
TC0800006566.hg.1	6.26	7.24	-1.97	0.0015	0.0061	AGPAT5	1-acylglycerol-3-phosphate O-acyltransferase	Multiple_Complex
TC1100010261.hg.1	7.31	7.65	-1.27	0.0015	0.0061	HPS5	Hermansky-Pudlak syndrome 5	Multiple_Complex
TC0100018489.hg.1	5.61	6.51	-1.87	0.0015	0.0061	VPS72	vacuolar protein sorting 72 homolog (S. cerevi	Multiple_Complex
TC1600006656.hg.1	8.44	7.38	2.08	0.0015	0.0061	MMP25	matrix metallopeptidase 25	Multiple_Complex
TC0100015764.hg.1	8.04	7.37	1.59	0.0015	0.0061	FAM63A	family with sequence similarity 63, member A	Multiple_Complex
TC1600009351.hg.1	4.75	4.22	1.45	0.0015	0.0061	GRIN2A	Transcript Identified by AceView, Entrez Gene	Unassigned
TC0900007113.hg.1	5.27	5.88	-1.53	0.0015	0.0061	RECK	reversion-inducing-cysteine-rich protein with	Multiple_Complex
TC1200012640.hg.1	11.04	9.8	2.37	0.0015	0.0062	BLOC1S1	biogenesis of lysosomal organelles complex-1,	Multiple_Complex
TC0800006963.hg.1	9.26	10.12	-1.81	0.0015	0.0062	XPO7	exportin 7	Multiple_Complex
TC1100011101.hg.1	9.49	8.89	1.51	0.0015	0.0062	NXF1	nuclear RNA export factor 1	Multiple_Complex
TC0200009350.hg.1	3.94	5.09	-2.23	0.0016	0.0062	C2orf27A	chromosome 2 open reading frame 27A	Multiple_Complex
TC0200013063.hg.1	4.68	4.08	1.51	0.0016	0.0062	EGR4	early growth response 4	Coding
TC0400011192.hg.1	14.05	15.16	-2.17	0.0016	0.0062	PLAC8	placenta specific 8	Multiple_Complex
TC1000012513.hg.1	6.17	6.86	-1.6	0.0016	0.0062	MTG1	mitochondrial ribosome-associated GTPase 1	Multiple_Complex
TC0700008582.hg.1	4.89	4.46	1.35	0.0016	0.0062	SERPINE1	serpin peptidase inhibitor, clade E (nexin, plas	Multiple_Complex
TC1300008443.hg.1	4.91	5.46	-1.47	0.0016	0.0062	LNX2	ligand of numb-protein X 2	Multiple_Complex
TC1700011055.hg.1	6.32	6.91	-1.5	0.0016	0.0062	SPOP	speckle-type POZ protein	Multiple_Complex
TC0100008061.hg.1	8.71	9.11	-1.33	0.0016	0.0063	SZT2	seizure threshold 2 homolog (mouse)	Multiple_Complex
TC0X00008344.hg.1	7.81	8.38	-1.48	0.0016	0.0063	SH2D1A	SH2 domain containing 1A	Multiple_Complex
TC0100018534.hg.1	4.96	4.51	1.37	0.0016	0.0063	TNR	tenascin R	Multiple_Complex
TC1300009348.hg.1	4.65	4.18	1.38	0.0016	0.0063	SPRY2	sprouty RTK signaling antagonist 2	Coding
TC1200008517.hg.1	6.76	6.2	1.48	0.0016	0.0063	METAP2	methionyl aminopeptidase 2	Multiple_Complex
TC0400006591.hg.1	8	8.59	-1.51	0.0016	0.0063	HTT	huntingtin	Multiple_Complex
TSUnmapped00000667.h	7.15	6.33	1.76	0.0016	0.0063	TRAPPC4	trafficking protein particle complex 4	NonCoding
TC0100009479.hg.1	5.61	6.35	-1.67	0.0016	0.0063	DCLRE1B	DNA cross-link repair 1B	Multiple_Complex
TC1800009279.hg.1	6.08	5.71	1.29	0.0016	0.0063	TCEB3CL; T	transcription elongation factor B polypeptide 3	Coding
TC2100008545.hg.1	8.77	8.29	1.4	0.0016	0.0063	NRIP1	nuclear receptor interacting protein 1	Multiple_Complex
TSUnmapped00000270.h	7.52	6.95	1.49	0.0016	0.0063	TRAPPC4	trafficking protein particle complex 4	Coding
TC0100009646.hg.1	10.93	10.06	1.83	0.0016	0.0063	NBPF26	neuroblastoma breakpoint family, member 26	Coding
TC1100011811.hg.1	9.64	8.7	1.92	0.0016	0.0063	CCDC90B	coiled-coil domain containing 90B	Multiple_Complex
TC1100012834.hg.1	5.43	4.92	1.43	0.0016	0.0063	ADAMTS8	ADAM metallopeptidase with thrombospondi	Multiple_Complex
TC1100010091.hg.1	6.95	6.37	1.5	0.0016	0.0063	MRVI1	murine retrovirus integration site 1 homolog	Multiple_Complex

TC0900012245.hg.1	5.93	6.75	-1.76	0.0016	0.0064	ALDH1A1	aldehyde dehydrogenase 1 family, member A1	Multiple_Complex
TSUnmapped00000380.h	8.9	9.47	-1.49	0.0016	0.0064	FBL	fibrillarin	Coding
TC1100011259.hg.1	5.45	4.71	1.68	0.0016	0.0064	FOSL1	FOS-like antigen 1	Multiple_Complex
TC1800008482.hg.1	9.35	10.15	-1.75	0.0016	0.0064	TPGS2	tubulin polyglutamylase complex subunit 2	Multiple_Complex
TC0300010185.hg.1	5.54	5.09	1.37	0.0016	0.0064	OXTR	oxytocin receptor	Multiple_Complex
TC0600007198.hg.1	6.81	7.35	-1.45	0.0016	0.0064	ALDH5A1	aldehyde dehydrogenase 5 family, member A1	Multiple_Complex
TC1400007232.hg.1	7.53	7.12	1.32	0.0016	0.0064	FBXO34	F-box protein 34	Multiple_Complex
TC0100013042.hg.1	4.74	4.28	1.38	0.0016	0.0064	SPATA21	spermatogenesis associated 21	Multiple_Complex
TC0200007419.hg.1	5.81	6.67	-1.82	0.0016	0.0064	CAMKMT	calmodulin-lysine N-methyltransferase	Multiple_Complex
TC0800009247.hg.1	6.18	6.77	-1.51	0.0016	0.0064	HSF1	heat shock transcription factor 1	Multiple_Complex
TC1700012285.hg.1	7.17	8.02	-1.8	0.0016	0.0065	PRR11	proline rich 11	Multiple_Complex
TC1100009833.hg.1	4.95	5.46	-1.42	0.0016	0.0065	OSBPL5	Memczak2013 ALT_ACCEPTOR, ALT_DONOR,	NonCoding
TC0300010744.hg.1	11.65	10.87	1.71	0.0016	0.0065	ACAA1	acetyl-CoA acyltransferase 1	Multiple_Complex
TC0200012009.hg.1	6.36	6.83	-1.38	0.0016	0.0065	DTNB	dystrobrevin beta	Multiple_Complex
TC0300013947.hg.1	5.38	6.01	-1.55	0.0016	0.0065	COLQ	collagen-like tail subunit (single strand of hom	Multiple_Complex
TC0100013038.hg.1	7.29	7.83	-1.46	0.0016	0.0065	FBXO42	F-box protein 42	Multiple_Complex
TC0100013441.hg.1	13.58	12.76	1.76	0.0016	0.0065	FGR	FGR proto-oncogene, Src family tyrosine kinas	Multiple_Complex
TSUnmapped00000803.h	4.16	3.86	1.23	0.0017	0.0066	OR5P3	olfactory receptor, family 5, subfamily P, mem	Coding
TC0400008922.hg.1	7.81	8.6	-1.72	0.0017	0.0066	LSM6	LSM6 homolog, U6 small nuclear RNA and mR	Multiple_Complex
TC1300008229.hg.1	9.15	8.5	1.57	0.0017	0.0066	ZMYM5	zinc finger, MYM-type 5	Multiple_Complex
TC0800006592.hg.1	3.94	3.41	1.45	0.0017	0.0066	USP17L1	ubiquitin specific peptidase 17-like family mer	Coding
TC0X00009707.hg.1	4.59	4.13	1.38	0.0017	0.0066	MAGED4; M	melanoma antigen family D4; melanoma antig	Multiple_Complex
TC1800007186.hg.1	7.31	8.07	-1.7	0.0017	0.0066	SETBP1	SET binding protein 1	Multiple_Complex
TC1100010730.hg.1	5.15	5.8	-1.57	0.0017	0.0066	ACP2	acid phosphatase 2, lysosomal	Multiple_Complex
TC1400007029.hg.1	5.25	6.35	-2.14	0.0017	0.0066	C14orf28	chromosome 14 open reading frame 28	Multiple_Complex
TC1900010706.hg.1	6.39	5.78	1.53	0.0017	0.0066	SERTAD1	SERTA domain containing 1	Multiple_Complex
TC1100006704.hg.1	4.98	5.83	-1.8	0.0017	0.0066	OR52N4	olfactory receptor, family 52, subfamily N, me	Coding
TC0600014247.hg.1	15.5	16.34	-1.78	0.0017	0.0066	FAM65B	family with sequence similarity 65, member B	Multiple_Complex
TC0100016263.hg.1	7.27	6.17	2.14	0.0017	0.0066	TMCO1	transmembrane and coiled-coil domains 1	Multiple_Complex
TC0200015080.hg.1	7.01	7.71	-1.63	0.0017	0.0066	PRKRA	protein kinase, interferon-inducible double str	Multiple_Complex
TC0500008864.hg.1	7.22	7.96	-1.67	0.0017	0.0066	WDR55	WD repeat domain 55	Multiple_Complex
TC1700010425.hg.1	7.3	8.04	-1.66	0.0017	0.0067	SLFN12	schlafen family member 12	Multiple_Complex
TC1000012575.hg.1	8.99	9.6	-1.53	0.0017	0.0067	ATAD1	ATPase family, AAA domain containing 1	Multiple_Complex
TC0600006950.hg.1	7.26	6.73	1.44	0.0017	0.0067	TMEM170B	transmembrane protein 170B	Coding
TC0800010749.hg.1	11.63	12.45	-1.76	0.0017	0.0067	NCOA2	nuclear receptor coactivator 2	Multiple_Complex
TC0500013064.hg.1	7.13	6.46	1.59	0.0017	0.0067	MGAT4B	mannosyl (alpha-1,3-)-glycoprotein beta-1,4-N	Multiple_Complex
TC0700008063.hg.1	6.15	6.79	-1.56	0.0017	0.0067	SPDYE5	speedy/RINGO cell cycle regulator family mem	Coding
TC0700009649.hg.1	6.19	5.63	1.47	0.0017	0.0067	ATP6V0E2	ATPase, H+ transporting V0 subunit e2	Multiple_Complex
TC0400012931.hg.1	13.49	12.78	1.64	0.0017	0.0067	G3BP2	GTPase activating protein (SH3 domain) bindi	Multiple_Complex
TC1400007430.hg.1	7.42	7.95	-1.44	0.0017	0.0067	SYNE2	spectrin repeat containing, nuclear envelope 2	Multiple_Complex
TC0100015976.hg.1	8.7	9.11	-1.33	0.0017	0.0067	KIAA0907;	KIAA0907; small Cajal body-specific RNA 4	Multiple_Complex
TC1700011798.hg.1	6.63	7.46	-1.78	0.0017	0.0067	UBE2O	ubiquitin-conjugating enzyme E2O	Multiple_Complex
TC0500009077.hg.1	6.4	7.01	-1.52	0.0017	0.0067	HMGXB3	HMG box domain containing 3	Multiple_Complex
TC0X00008940.hg.1	5.09	4.59	1.42	0.0017	0.0067	MXRA5	matrix-remodelling associated 5	Coding
TC1900010008.hg.1	6.81	7.48	-1.59	0.0017	0.0067	KIAA1683	KIAA1683	Multiple_Complex
TC1900009692.hg.1	7.08	6.52	1.48	0.0017	0.0067	ZNF823	zinc finger protein 823	Multiple_Complex
TC1000008579.hg.1	8.75	7.82	1.91	0.0017	0.0067	UBTD1	ubiquitin domain containing 1	Multiple_Complex
TC0400009322.hg.1	7.94	8.51	-1.48	0.0017	0.0067	GALNT7	polypeptide N-acetylgalactosaminyltransferas	Multiple_Complex
TC0200016545.hg.1	4.12	4.83	-1.64	0.0017	0.0068	RIF1	replication timing regulatory factor 1	NonCoding
TC1_KI270713v1_random	7.4	7.82	-1.34	0.0017	0.0068	FAM231A	family with sequence similarity 231, member A	Coding
TC0X00010005.hg.1	6.93	7.66	-1.66	0.0017	0.0068	CXCR3	chemokine (C-X-C motif) receptor 3	Coding
TC1600011047.hg.1	5.79	5.59	1.14	0.0017	0.0068	HSDL1	hydroxysteroid dehydrogenase like 1	Multiple_Complex
TC0700008011.hg.1	17.67	16.49	2.27	0.0017	0.0068	NCF1	neutrophil cytosolic factor 1	Multiple_Complex
TC0200016707.hg.1	4.86	5.37	-1.42	0.0017	0.0068	ZNF514	zinc finger protein 514	Multiple_Complex
TC1100009078.hg.1	6.61	7.18	-1.49	0.0017	0.0068	TTC12	tetratricopeptide repeat domain 12	Multiple_Complex
TSUnmapped00000306.h	4.31	4.9	-1.51	0.0017	0.0068	ZKSCAN7	zinc finger with KRAB and SCAN domains 7	Coding
TC0100018478.hg.1	11.84	10.96	1.83	0.0017	0.0068	NBPF10	neuroblastoma breakpoint family, member 10	Multiple_Complex
TC1700010461.hg.1	6.19	5.32	1.83	0.0017	0.0068	CCL3L3; CC	chemokine (C-C motif) ligand 3-like 3; chemok	Multiple_Complex
TC0X00009498.hg.1	6.02	5.38	1.56	0.0017	0.0068	FUNDC1	FUN14 domain containing 1	Multiple_Complex
TC0600007693.hg.1	13.62	12.42	2.3	0.0017	0.0068	RPS18	ribosomal protein S18	Multiple_Complex
TC0900010388.hg.1	10.29	9.13	2.22	0.0017	0.0068	TMEM2	Transcript Identified by AceView, Entrez Gene	Unassigned
TC1700008661.hg.1	5.59	6.17	-1.49	0.0017	0.0068	PRKCA	protein kinase C, alpha	Multiple_Complex
TC1900009192.hg.1	6.44	7.02	-1.5	0.0017	0.0068	ABHD17A	abhydrolase domain containing 17A	Multiple_Complex
TC0600008104.hg.1	3.76	3.4	1.29	0.0017	0.0068	RSPH9	radial spoke head 9 homolog (Chlamydomona	Coding
TC1700011205.hg.1	6.33	5.72	1.52	0.0017	0.0068	C17orf67	chromosome 17 open reading frame 67	Multiple_Complex
TC1200012737.hg.1	4.96	5.78	-1.76	0.0017	0.0069	ZNF268	zinc finger protein 268	Multiple_Complex
TC0300010390.hg.1	9.71	9.19	1.44	0.0017	0.0069	RBSN	rabenosyn, RAB effector	Multiple_Complex
TC0800011710.hg.1	7.38	6.51	1.84	0.0017	0.0069	TATDN1; M	TatD DNase domain containing 1; microRNA 6	Multiple_Complex
TC0600007344.hg.1	4.28	3.88	1.32	0.0018	0.0069	PRSS16	protease, serine, 16 (thymus)	Multiple_Complex
TC1600007532.hg.1	4.86	4.36	1.41	0.0018	0.0069	ITGAD	integrin alpha D	Multiple_Complex

TC0200008224.hg.1	6.03	5.5	1.44	0.0018	0.0069	DNAH6	Transcript Identified by AceView, Entrez Gene	Unassigned
TC1400009766.hg.1	6.61	5.49	2.17	0.0018	0.0069	IRF2BPL	interferon regulatory factor 2 binding protein-	Multiple_Complex
TC1900009249.hg.1	9.45	8.71	1.68	0.0018	0.0069	GNG7	Memczak2013 ALT_ACCEPTOR, ALT_DONOR,	NonCoding
TC2200009161.hg.1	6.39	6.74	-1.27	0.0018	0.0069	LMF2	lipase maturation factor 2	Multiple_Complex
TC0200008285.hg.1	7.21	7.68	-1.39	0.0018	0.0069	MRPL35	mitochondrial ribosomal protein L35	Multiple_Complex
TC0600009406.hg.1	4.32	3.64	1.61	0.0018	0.0069	C6orf58	chromosome 6 open reading frame 58	Multiple_Complex
TC0900012021.hg.1	7.33	8.12	-1.72	0.0018	0.007	FBXW5	F-box and WD repeat domain containing 5	Multiple_Complex
TC1400010634.hg.1	5.27	5.72	-1.37	0.0018	0.007	CIPC	CLOCK-interacting pacemaker	Multiple_Complex
TC1700012349.hg.1	5.96	5.46	1.41	0.0018	0.007	NAA38	N(alpha)-acetyltransferase 38, NatC auxiliary s	Multiple_Complex
TC1100011614.hg.1	5.42	6.24	-1.77	0.0018	0.007	KCNE3	potassium channel, voltage gated subfamily E	Coding
TC0200006891.hg.1	8.05	9.02	-1.97	0.0018	0.007	RHOB	ras homolog family member B	Coding
TC0200016739.hg.1	4.64	5.41	-1.7	0.0018	0.007	ARL5A	ADP-ribosylation factor like GTPase 5A	NonCoding
TC0600011562.hg.1	7.57	8.12	-1.47	0.0018	0.007	LEMD2	LEM domain containing 2	Multiple_Complex
TC0700006484.hg.1	5.48	6.13	-1.56	0.0018	0.007	GPR146	G protein-coupled receptor 146	Multiple_Complex
TC0X00009250.hg.1	9.18	8.3	1.84	0.0018	0.007	ACOT9	acyl-CoA thioesterase 9	Multiple_Complex
TC0300011731.hg.1	11.97	12.43	-1.37	0.0018	0.007	CGGBP1	CGG triplet repeat binding protein 1	Multiple_Complex
TC0100006982.hg.1	12.57	11.95	1.55	0.0018	0.007	EFHD2	EF-hand domain family member D2	Coding
TC0600012495.hg.1	5.98	5.3	1.59	0.0018	0.007	CGA	glycoprotein hormones, alpha polypeptide	Multiple_Complex
TC0100018481.hg.1	9.08	8.47	1.52	0.0018	0.0071	NOTCH2NL	notch 2 N-terminal like	Coding
TC0600009753.hg.1	9.89	8.88	2.01	0.0018	0.0071	STXBP5	syntaxin binding protein 5 (tomosyn)	Multiple_Complex
TC1900010696.hg.1	7.84	8.87	-2.04	0.0018	0.0071	AKT2	v-akt murine thymoma viral oncogene homolo	Multiple_Complex
TC1200009528.hg.1	6	6.64	-1.56	0.0018	0.0071	ZNF140	zinc finger protein 140	Multiple_Complex
TC1900011798.hg.1	7.92	8.86	-1.92	0.0018	0.0071	LILRA1	leukocyte immunoglobulin-like receptor, subfa	Multiple_Complex
TC1700012094.hg.1	4.63	4.07	1.48	0.0018	0.0071	CCDC57	coiled-coil domain containing 57	Multiple_Complex
TC1400007725.hg.1	5.19	4.64	1.47	0.0018	0.0071	ESRRB	estrogen-related receptor beta	Multiple_Complex
TC0100015791.hg.1	5.56	4.28	2.42	0.0018	0.0071	POGZ	Transcript Identified by AceView, Entrez Gene	Unassigned
TC1100012002.hg.1	7.75	8.57	-1.78	0.0018	0.0071	MRE11A	MRE11 homolog A, double strand break repai	Multiple_Complex
TC0500012023.hg.1	4.6	5.24	-1.56	0.0018	0.0071	KIF3A	kinesin family member 3A	Multiple_Complex
TC0200009095.hg.1	4.68	4.25	1.35	0.0018	0.0071	INHBB	inhibin beta B	Coding
TC2000007994.hg.1	4.92	5.23	-1.24	0.0018	0.0071	MTG2	mitochondrial ribosome-associated GTPase 2	Multiple_Complex
TC1600007504.hg.1	7.27	8.06	-1.73	0.0018	0.0071	SETD1A	SET domain containing 1A	Multiple_Complex
TC0100015795.hg.1	7.62	6.71	1.88	0.0018	0.0071	POGZ	Transcript Identified by AceView, Entrez Gene	Unassigned
TC0800012245.hg.1	6.09	6.57	-1.4	0.0018	0.0072	ZNF250	zinc finger protein 250	Multiple_Complex
TC1900010005.hg.1	5	4.59	1.33	0.0018	0.0072	RAB3A	RAB3A, member RAS oncogene family	Multiple_Complex
TC0X00009339.hg.1	10.24	10.7	-1.38	0.0018	0.0072	TAB3	TGF-beta activated kinase 1/MAP3K7 binding	Multiple_Complex
TC1400009842.hg.1	11.97	11.15	1.78	0.0018	0.0072	SEL1L	sel-1 suppressor of lin-12-like (C. elegans)	Multiple_Complex
TSUnmapped00000742.h	6.01	6.47	-1.37	0.0018	0.0072	ZNF852	zinc finger protein 852	Coding
TC1000008063.hg.1	7.17	7.79	-1.54	0.0018	0.0072	ADK; MRPL	adenosine kinase; mitochondrial ribosomal pr	Multiple_Complex
TC1100010738.hg.1	6.49	5.9	1.51	0.0018	0.0072	PSMC3	proteasome 26S subunit, ATPase 3	Multiple_Complex
TC2000007448.hg.1	5.51	4.97	1.46	0.0018	0.0072	GDAP1L1	ganglioside induced differentiation associated	Multiple_Complex
TC1100008804.hg.1	7.78	6.41	2.6	0.0018	0.0072	ANKRD49	ankyrin repeat domain 49	Multiple_Complex
TC0500013358.hg.1	12.55	11.53	2.03	0.0018	0.0072	SKP1	S-phase kinase-associated protein 1	Multiple_Complex
TC1900008059.hg.1	10.76	10.07	1.61	0.0019	0.0072	SUPT5H	SPT5 homolog, DSIF elongation factor subunit	Multiple_Complex
TC1400010632.hg.1	10.03	9.65	1.31	0.0019	0.0072	GPATCH2L	G-patch domain containing 2 like	Multiple_Complex
TC1600011398.hg.1	5.09	4.35	1.67	0.0019	0.0072	MMP2	matrix metallopeptidase 2	Multiple_Complex
TC1800008609.hg.1	5.56	5.21	1.27	0.0019	0.0073	TCEB3C	transcription elongation factor B polypeptide :	Coding
TC0600007833.hg.1	7.19	5.99	2.3	0.0019	0.0073	PNPLA1	patatin-like phospholipase domain containing	Coding
TC0300008863.hg.1	14.48	13.71	1.71	0.0019	0.0073	CDV3	CDV3 homolog (mouse)	Multiple_Complex
TC0600009094.hg.1	7.07	6.55	1.44	0.0019	0.0073	SMPD2	sphingomyelin phosphodiesterase 2, neutral r	Multiple_Complex
TC0900008865.hg.1	4.92	5.45	-1.44	0.0019	0.0073	CERCAM	cerebral endothelial cell adhesion molecule	Multiple_Complex
TC0200013552.hg.1	3.87	4.13	-1.2	0.0019	0.0073	FAHD2B	fumarylacetoacetate hydrolase domain contai	Multiple_Complex
TC1500009728.hg.1	6.95	5.96	1.98	0.0019	0.0073	DAPK2	death-associated protein kinase 2	Multiple_Complex
TC1400008056.hg.1	7.1	10.81	-13.07	0.0019	0.0073	IFI27	interferon, alpha-inducible protein 27	Multiple_Complex
TC0500012302.hg.1	4	4.36	-1.29	0.0019	0.0074	GNPDA1	glucosamine-6-phosphate deaminase 1	Multiple_Complex
TC1900011839.hg.1	4.02	3.48	1.46	0.0019	0.0074	JSRP1	junctional sarcoplasmic reticulum protein 1	NonCoding
TC0700008743.hg.1	11.59	12.28	-1.62	0.0019	0.0074	PIK3CG	phosphatidylinositol-4,5-bisphosphate 3-kinas	Multiple_Complex
TC0400010085.hg.1	10.86	9.99	1.83	0.0019	0.0074	BOD1L1	biorientation of chromosomes in cell division	Multiple_Complex
TC1700007326.hg.1	4.9	4.44	1.37	0.0019	0.0074	TBC1D3P5	TBC1 domain family, member 3 pseudogene 5	Multiple_Complex
TC0100010486.hg.1	5.32	5.84	-1.44	0.0019	0.0074	POGK	pogo transposable element with KRAB domair	Coding
TC1500007004.hg.1	11.2	10.39	1.76	0.0019	0.0074	RTF1	RTF1 homolog, Paf1/RNA polymerase II compl	Multiple_Complex
TC0800012147.hg.1	9.02	9.46	-1.36	0.0019	0.0074	TSTA3	tissue specific transplantation antigen P35B	Multiple_Complex
TC2200008866.hg.1	8.1	8.32	-1.17	0.0019	0.0074	TCF20	transcription factor 20 (AR1)	Multiple_Complex
TC0X00009493.hg.1	5.68	4.78	1.87	0.0019	0.0074	EFHC2	EF-hand domain (C-terminal) containing 2	Multiple_Complex
TC1100012713.hg.1	5.19	4.65	1.45	0.0019	0.0074	PATE2	prostate and testis expressed 2	Coding
TC1400006711.hg.1	4.8	5.32	-1.44	0.0019	0.0074	DHRS4L1	dehydrogenase/reductase (SDR family) memb	Multiple_Complex
TC1400009793.hg.1	8.94	8.3	1.56	0.0019	0.0074	SNW1	SNW domain containing 1	Multiple_Complex
TC0600013004.hg.1	8.84	9.22	-1.3	0.0019	0.0074	MCM9	minichromosome maintenance 9 homologous	Multiple_Complex
TC1100011118.hg.1	5.1	4.61	1.41	0.0019	0.0074	SLC22A25	solute carrier family 22, member 25	Multiple_Complex
TC0600010056.hg.1	12.33	11.57	1.69	0.0019	0.0075	WTAP	Wilms tumor 1 associated protein	Multiple_Complex
TC1700010191.hg.1	7.6	8.08	-1.4	0.0019	0.0075	POLDIP2	polymerase (DNA-directed), delta interacting		Multiple_Complex

TC0100007505.hg.1	9.91	10.61	-1.62	0.0019	0.0075	WDTC1	WD and tetratricopeptide repeats 1	Multiple_Complex
TC0100007813.hg.1	5.6	4.43	2.25	0.0019	0.0075	CSF3R	Memczak2013 ANTISENSE, CDS, coding, INTEF	NonCoding
TC0600007842.hg.1	10.29	9.59	1.62	0.0019	0.0075	SRSF3	serine/arginine-rich splicing factor 3	Multiple_Complex
TC1000007888.hg.1	11.05	10.24	1.75	0.0019	0.0075	VPS26A	VPS26 retromer complex component A	Multiple_Complex
TC0800010158.hg.1	7.25	7.71	-1.37	0.0019	0.0075	PLPP5	phospholipid phosphatase 5	Multiple_Complex
TC1200011519.hg.1	7.28	8	-1.65	0.0019	0.0075	EEA1	early endosome antigen 1	Multiple_Complex
TC1900008608.hg.1	5.79	5.22	1.49	0.0019	0.0075	KLK2	kallikrein related peptidase 2	Multiple_Complex
TC0300010632.hg.1	6.43	7.23	-1.75	0.002	0.0076	OSBPL10	oxysterol binding protein-like 10	Multiple_Complex
TC1800009086.hg.1	8.91	7.78	2.19	0.002	0.0076	ZNF516	zinc finger protein 516	Multiple_Complex
TC1600008924.hg.1	7.1	7.41	-1.24	0.002	0.0076	POLR3K	polymerase (RNA) III (DNA directed) polypepti	Multiple_Complex
TC0100018344.hg.1	9.5	9.96	-1.38	0.002	0.0076	LYPLAL1	lysophospholipase-like 1	Multiple_Complex
TC1400008921.hg.1	11.14	10.25	1.84	0.002	0.0076	BAZ1A	bromodomain adjacent to zinc finger domain	Multiple_Complex
TC0300013827.hg.1	4.32	5.19	-1.83	0.002	0.0076	WDR6	WD repeat domain 6	Multiple_Complex
TC0X00011279.hg.1	5	5.57	-1.48	0.002	0.0076	TSPAN7	tetraspanin 7	Multiple_Complex
TSUnmapped00000333.h	9.94	10.73	-1.72	0.002	0.0076	SURF4	surfeit 4	Coding
TC1700007454.hg.1	11.24	11.53	-1.22	0.002	0.0077	GOSR1	golgi SNAP receptor complex member 1	Multiple_Complex
TC0X00009537.hg.1	6.75	7.32	-1.48	0.002	0.0077	ZNF674	zinc finger protein 674	Multiple_Complex
TC0X00007369.hg.1	5.09	5.44	-1.27	0.002	0.0077	APEX2	APEX nuclease (apurinic/apyrimidinic endonu	Multiple_Complex
TC1700009504.hg.1	6.94	7.78	-1.79	0.002	0.0077	ANKFY1	ankyrin repeat and FYVE domain containing 1	Multiple_Complex
TC0600010542.hg.1	8.64	8	1.56	0.002	0.0077	SERPINB6	serpin peptidase inhibitor, clade B (ovalbumin	Multiple_Complex
TC2000006818.hg.1	5.47	6.24	-1.7	0.002	0.0077	PET117; CS	PET117 homolog; CSRP2 binding protein	Multiple_Complex
TC0600010307.hg.1	5.48	6.11	-1.54	0.002	0.0077	ERMARD	ER membrane-associated RNA degradation	Multiple_Complex
TC0100010112.hg.1	6.78	6.18	1.52	0.002	0.0077	SLC50A1	solute carrier family 50 (sugar efflux transport	Multiple_Complex
TC0900011864.hg.1	4.92	5.81	-1.86	0.002	0.0077	VAV2	vav 2 guanine nucleotide exchange factor	Multiple_Complex
TC1100013091.hg.1	8.25	8.48	-1.17	0.002	0.0077	SDHD	succinate dehydrogenase complex subunit D,	Multiple_Complex
TC2000009588.hg.1	7.18	6.62	1.47	0.002	0.0077	MTRNR2L3	MT-RNR2-like 3	Coding
TC1600006733.hg.1	4.15	3.9	1.19	0.002	0.0077	GLIS2	GLIS family zinc finger 2	Multiple_Complex
TC0400009198.hg.1	5.07	4.48	1.51	0.002	0.0077	APELA	apelin receptor early endogenous ligand	Multiple_Complex
TC1400010580.hg.1	8.9	9.35	-1.37	0.002	0.0078	DHRS4L2	dehydrogenase/reductase (SDR family) memb	Multiple_Complex
TC0200015964.hg.1	6.59	5.81	1.71	0.002	0.0078	TRIP12	Transcript Identified by AceView, Entrez Gene	Unassigned
TC1500010784.hg.1	7.27	7.75	-1.39	0.002	0.0078	NGRN; TTLI	neugrin, neurite outgrowth associated; tubulir	Multiple_Complex
TC1100009896.hg.1	5.44	4.8	1.55	0.002	0.0078	OR51S1	olfactory receptor, family 51, subfamily S, mer	Coding
TC0500009409.hg.1	7.29	6.6	1.61	0.002	0.0079	GABRP	gamma-aminobutyric acid (GABA) A receptor,	Multiple_Complex
TC1400007222.hg.1	10.57	11.25	-1.6	0.002	0.0079	MAPK1IP1L	mitogen-activated protein kinase 1 interacting	Multiple_Complex
TC0900012202.hg.1	9.37	10.01	-1.56	0.002	0.0079	KIAA2026	KIAA2026	Multiple_Complex
TC0200009662.hg.1	10.27	9.07	2.3	0.002	0.0079	TNFAIP6	tumor necrosis factor, alpha-induced protein (	Multiple_Complex
TC0400008803.hg.1	9.97	9.08	1.85	0.002	0.0079	RAB33B	RAB33B, member RAS oncogene family	Multiple_Complex
TC0X00010734.hg.1	6.04	4.6	2.72	0.002	0.0079	TENM1	teneurin transmembrane protein 1	Multiple_Complex
TC1100010996.hg.1	13.02	12.03	1.99	0.002	0.0079	MS4A6A	membrane-spanning 4-domains, subfamily A,	Multiple_Complex
TC1000010070.hg.1	6.7	5.87	1.77	0.0021	0.0079	PRTFDC1	phosphoribosyl transferase domain containing	Multiple_Complex
TC0700013231.hg.1	5.57	5.31	1.2	0.0021	0.0079	MNX1	motor neuron and pancreas homeobox 1	Multiple_Complex
TC0800012026.hg.1	5.8	7.02	-2.33	0.0021	0.0079	SLC45A4	solute carrier family 45, member 4	Multiple_Complex
TC0900008148.hg.1	8.86	10.1	-2.36	0.0021	0.0079	TDRD7	tudor domain containing 7	Multiple_Complex
TC0100010234.hg.1	6.4	5.86	1.45	0.0021	0.008	OR10R2	olfactory receptor, family 10, subfamily R, me	Coding
TC1900011887.hg.1	7.76	8.26	-1.41	0.0021	0.008	ZNF490	zinc finger protein 490	Multiple_Complex
TC1500007318.hg.1	4.79	4.07	1.65	0.0021	0.008	TEX9	testis expressed 9	Multiple_Complex
TC1700011949.hg.1	5	5.42	-1.34	0.0021	0.008	CBX8	chromobox homolog 8	Multiple_Complex
TC0600012199.hg.1	4.97	4.51	1.38	0.0021	0.008	EYS	eyes shut homolog (Drosophila)	Multiple_Complex
TC0100009048.hg.1	7.96	7.02	1.92	0.0021	0.008	RPL5	Memczak2013 ALT_ACCEPTOR, ALT_DONOR,	NonCoding
TC0500007552.hg.1	8.64	7.94	1.62	0.0021	0.008	LOC100421	family with sequence similarity 133, member	Multiple_Complex
TC2000009016.hg.1	6.74	7.43	-1.62	0.0021	0.008	DSN1	DSN1 homolog, MIS12 kinetochore complex c	Multiple_Complex
TC0100010241.hg.1	15.58	14.28	2.46	0.0021	0.008	MNDA	myeloid cell nuclear differentiation antigen	Multiple_Complex
TC0500012204.hg.1	5.17	4.74	1.35	0.0021	0.008	SLC23A1	solute carrier family 23 (ascorbic acid transpo	Multiple_Complex
TC0300013911.hg.1	8.73	8.06	1.59	0.0021	0.008	DNAJB11	DnaJ (Hsp40) homolog, subfamily B, member	Multiple_Complex
TC1900011693.hg.1	7.3	6.74	1.48	0.0021	0.0081	ZNF429	zinc finger protein 429	Coding
TC1000010993.hg.1	9.49	8.83	1.58	0.0021	0.0081	MICU1	mitochondrial calcium uptake 1	Multiple_Complex
TC0400010772.hg.1	5.51	4.66	1.8	0.0021	0.0081	SRP72	Memczak2013 ANTISENSE, coding, INTERNAL,	NonCoding
TC1200006629.hg.1	17.66	17.26	1.32	0.0021	0.0081	GAPDH	glyceraldehyde-3-phosphate dehydrogenase	Multiple_Complex
TC0500009725.hg.1	8.37	9	-1.55	0.0021	0.0081	CNOT6	CCR4-NOT transcription complex subunit 6	Multiple_Complex
TC1400007691.hg.1	7.83	6.98	1.79	0.0021	0.0081	DLST	dihydrolipoamide S-succinyltransferase (E2 co	Multiple_Complex
TC1200008671.hg.1	7.43	8.33	-1.86	0.0021	0.0081	HCFC2	host cell factor C2	Multiple_Complex
TC0400012795.hg.1	10.88	11.57	-1.61	0.0021	0.0081	FIP1L1	factor interacting with PAPOLA and CPSF1	Multiple_Complex
TC0300013215.hg.1	7.11	8.03	-1.88	0.0021	0.0081	TBL1XR1	transducin (beta)-like 1 X-linked receptor 1	Multiple_Complex
TC2000007005.hg.1	16.45	15.31	2.2	0.0021	0.0082	CST7	cystatin F (leukocystatin)	Coding
TC0700009232.hg.1	8.96	10.06	-2.15	0.0021	0.0082	BPGM	2,3-bisphosphoglycerate mutase	Coding
TC1400007098.hg.1	12.09	12.54	-1.37	0.0021	0.0082	ARF6	ADP-ribosylation factor 6	Multiple_Complex
TC1200008370.hg.1	5.39	6.06	-1.59	0.0021	0.0082	C12orf29	chromosome 12 open reading frame 29	Multiple_Complex
TC0900007167.hg.1	7.57	8.19	-1.54	0.0021	0.0082	DCAF10	DDB1 and CUL4 associated factor 10	Multiple_Complex
TC1900007732.hg.1	5.12	4.6	1.44	0.0021	0.0082	THEG5; AC(	testis highly expressed protein 5; novel transc	Multiple_Complex
TC0400009503.hg.1	5.36	6.01	-1.57	0.0021	0.0082	PRIMPOL	primase and DNA directed polymerase	Multiple_Complex

TC0500009470.hg.1	14.31	13.39	1.89	0.0021	0.0082	ERGIC1	endoplasmic reticulum-golgi intermediate cor	Multiple_Complex
TSUnmapped00000627.h	4.91	4.3	1.53	0.0021	0.0082	KIF15	kinesin family member 15	Coding
TC0400012768.hg.1	11.12	10.35	1.69	0.0021	0.0082	FAM200B	family with sequence similarity 200, member	Multiple_Complex
TC0100013071.hg.1	4.02	3.72	1.23	0.0022	0.0082	MFAP2	microfibrillar associated protein 2	Multiple_Complex
TC1200010569.hg.1	10.63	11.44	-1.75	0.0022	0.0082	ASB8	ankyrin repeat and SOCS box containing 8	Multiple_Complex
TC1600009224.hg.1	6.36	5.75	1.53	0.0022	0.0082	CORO7; PA	coronin 7; presequence translocase-associate	Multiple_Complex
TC0100015805.hg.1	6.48	5.72	1.69	0.0022	0.0082	CELF3	CUGBP, Elav-like family member 3	Multiple_Complex
TC0700012479.hg.1	7.33	8.11	-1.72	0.0022	0.0083	POT1	protection of telomeres 1	Multiple_Complex
TC1200012678.hg.1	4.63	3.94	1.62	0.0022	0.0083	PTPRQ	protein tyrosine phosphatase, receptor type, (	Multiple_Complex
TC0300013931.hg.1	5.65	5.12	1.44	0.0022	0.0083	SSUH2	ssu-2 homolog (C. elegans)	NonCoding
TC0100011388.hg.1	5.73	5.07	1.57	0.0022	0.0083	IL19	interleukin 19	Multiple_Complex
TC0100013192.hg.1	6.46	6.18	1.21	0.0022	0.0083	SH2D5	SH2 domain containing 5	Multiple_Complex
TC1100012478.hg.1	6.39	7.58	-2.28	0.0022	0.0083	CD3D	CD3d molecule, delta (CD3-TCR complex)	Multiple_Complex
TC0300012449.hg.1	4.87	4.07	1.75	0.0022	0.0083	CPNE4	copine IV	Multiple_Complex
TC1600011306.hg.1	5.8	5.35	1.36	0.0022	0.0083	PRDM7	PR domain containing 7	Multiple_Complex
TC1300006474.hg.1	5.73	6.45	-1.65	0.0022	0.0083	MPHOSPH8	M-phase phosphoprotein 8	Multiple_Complex
TC0300006627.hg.1	5.54	6.11	-1.48	0.0022	0.0084	TSEN2	TSEN2 tRNA splicing endonuclease subunit	Multiple_Complex
TC1900008050.hg.1	5.07	4.52	1.46	0.0022	0.0084	IFNL2	interferon, lambda 2	Coding
TC0600011674.hg.1	6.43	7.13	-1.63	0.0022	0.0084	PPIL1	peptidylprolyl isomerase (cyclophilin)-like 1	Multiple_Complex
TC2200007398.hg.1	6.38	7.06	-1.6	0.0022	0.0084	SYNGR1	synaptogyrin 1	Multiple_Complex
TC0100007486.hg.1	7.08	6.5	1.5	0.0022	0.0084	SFN	stratifin	Coding
TC0900012002.hg.1	6.14	5.59	1.46	0.0022	0.0084	LCN8	lipocalin 8	Multiple_Complex
TC0X00010462.hg.1	5.92	6.54	-1.53	0.0022	0.0084	RBM41	RNA binding motif protein 41	Multiple_Complex
TC0100008034.hg.1	4.2	4.87	-1.59	0.0022	0.0084	ZNF691	zinc finger protein 691	Multiple_Complex
TC1900011838.hg.1	6	5.55	1.36	0.0022	0.0084	JSRP1	junctional sarcoplasmic reticulum protein 1	Multiple_Complex
TC1600009849.hg.1	13.82	12.72	2.15	0.0022	0.0084	XPO6	exportin 6	Multiple_Complex
TC1000010432.hg.1	6.85	6.34	1.43	0.0022	0.0084	RASGEF1A	RasGEF domain family member 1A	Multiple_Complex
TC1900009846.hg.1	5.32	4.74	1.5	0.0022	0.0085	OR7A17	olfactory receptor, family 7, subfamily A, mem	Multiple_Complex
TC1800008679.hg.1	8.02	8.41	-1.3	0.0022	0.0085	MBD1	methyl-CpG binding domain protein 1	Multiple_Complex
TC2000009360.hg.1	10.4	9.8	1.51	0.0022	0.0085	PREX1	Memczak2013 ALT_ACCEPTOR, ALT_DONOR,	NonCoding
TC0100014275.hg.1	5.6	6.15	-1.46	0.0022	0.0085	TTC22	tetratricopeptide repeat domain 22	Multiple_Complex
TC0300011000.hg.1	5.11	5.78	-1.59	0.0022	0.0085	MAP4	Transcript Identified by AceView, Entrez Gene	Unassigned
TC0100009881.hg.1	7.72	7.17	1.46	0.0022	0.0085	BOLA1	bolA family member 1	Multiple_Complex
TC1000006803.hg.1	6.68	5.98	1.63	0.0022	0.0085	CAMK1D	Memczak2013 ALT_ACCEPTOR, ALT_DONOR,	NonCoding
TC0100012787.hg.1	6.64	5.76	1.84	0.0022	0.0085	SLC2A5	solute carrier family 2 (facilitated glucose/fruc	Multiple_Complex
TC0200008923.hg.1	3.96	4.26	-1.23	0.0022	0.0085	POLR1B	polymerase (RNA) I polypeptide B	Multiple_Complex
TC0200008323.hg.1	4.96	5.89	-1.91	0.0022	0.0085	PLGLB2	plasminogen-like B2	Multiple_Complex
TC1200010113.hg.1	4.11	3.34	1.7	0.0022	0.0085	ABCC9	Transcript Identified by AceView, Entrez Gene	Unassigned
TC1500009756.hg.1	5.9	4.76	2.2	0.0022	0.0085	RBPMS2	RNA binding protein with multiple splicing 2	Coding
TC2100007205.hg.1	11.19	13.01	-3.54	0.0022	0.0086	MX2	MX dynamin-like GTPase 2	Multiple_Complex
TC1400006851.hg.1	6.88	7.46	-1.5	0.0023	0.0086	ARHGAP5	Rho GTPase activating protein 5	Multiple_Complex
TC1900008967.hg.1	5.96	6.56	-1.51	0.0023	0.0086	ZNF470	zinc finger protein 470	Multiple_Complex
TC0500009037.hg.1	4.35	3.88	1.39	0.0023	0.0086	SPINK7	serine peptidase inhibitor, Kazal type 7 (putati	Multiple_Complex
TC1900011933.hg.1	6.82	7.52	-1.63	0.0023	0.0086	ZNF585A	zinc finger protein 585A	Multiple_Complex
TC0100011573.hg.1	7.45	8.33	-1.84	0.0023	0.0086	SMYD2	SET and MYND domain containing 2	Multiple_Complex
TC1200008873.hg.1	7.19	7.77	-1.5	0.0023	0.0086	SH2B3	SH2B adaptor protein 3	Multiple_Complex
TC1900009079.hg.1	5.1	4.53	1.48	0.0023	0.0086	MIER2	Zhang2013 ALT_ACCEPTOR, ALT_DONOR, cod	NonCoding
TSUnmapped00000009.h	6.46	5.76	1.62	0.0023	0.0086	SURF1	surfeit 1	NonCoding
TC0100016977.hg.1	5.42	4.9	1.43	0.0023	0.0087	MYOG	myogenin (myogenic factor 4)	Coding
TC1100007675.hg.1	5.2	4.87	1.25	0.0023	0.0087	RTN4RL2	reticulon 4 receptor-like 2	Coding
TC0200010053.hg.1	5.18	4.73	1.36	0.0023	0.0087	HOXD1; MI	homeobox D1; microRNA 7704	Multiple_Complex
TC1100011754.hg.1	3.68	3.32	1.28	0.0023	0.0087	TENM4	teneurin transmembrane protein 4	Multiple_Complex
TC0200014407.hg.1	9.13	9.84	-1.64	0.0023	0.0087	DARS	aspartyl-tRNA synthetase	Multiple_Complex
TC1200010809.hg.1	7.46	8.25	-1.73	0.0023	0.0087	ATF7; NPFF	activating transcription factor 7; neuropeptide	Multiple_Complex
TC1900011150.hg.1	6.49	7.27	-1.72	0.0023	0.0087	NOSIP	nitric oxide synthase interacting protein	Multiple_Complex
TC0400006433.hg.1	7.03	7.82	-1.72	0.0023	0.0087	ZNF595	zinc finger protein 595	Multiple_Complex
TC1700012362.hg.1	4.37	5.12	-1.69	0.0023	0.0087	TRIM16	tripartite motif containing 16	Multiple_Complex
TC1900008469.hg.1	4.84	4.24	1.51	0.0023	0.0087	SYNGR4	synaptogyrin 4	Coding
TC1700012371.hg.1	5.56	6.18	-1.53	0.0023	0.0087	ZNF286B; F	zinc finger protein 286B; forkhead box O3B ps	Multiple_Complex
TC1100006906.hg.1	11.26	10.54	1.65	0.0023	0.0087	FAR1	fatty acyl CoA reductase 1	Multiple_Complex
TC1700009540.hg.1	5.7	5.35	1.27	0.0023	0.0087	SPAG7	sperm associated antigen 7	Multiple_Complex
TC1200012163.hg.1	9.22	8.67	1.46	0.0023	0.0087	SRSF9	serine/arginine-rich splicing factor 9	Multiple_Complex
TC0600011123.hg.1	5.22	4.41	1.76	0.0023	0.0087	HIST1H4B	histone cluster 1, H4b	Coding
TC1900008833.hg.1	6.78	7.82	-2.05	0.0023	0.0088	CNOT3	CCR4-NOT transcription complex subunit 3	Multiple_Complex
TC1700007791.hg.1	11.67	10.91	1.69	0.0023	0.0088	RARA	retinoic acid receptor, alpha	Multiple_Complex
TC0300007016.hg.1	5.16	4.63	1.44	0.0023	0.0088	ARPP21	Transcript Identified by AceView, Entrez Gene	Unassigned
TSUnmapped00000179.h	5.45	6.05	-1.51	0.0023	0.0088	DYRK1B	dual specificity tyrosine-(Y)-phosphorylation r	Coding
TC0X00006958.hg.1	16.2	17.11	-1.88	0.0023	0.0088	CYBB	cytochrome b-245, beta polypeptide	Multiple_Complex
TC1500009738.hg.1	6	5.25	1.68	0.0023	0.0088	KIAA0101;	KIAA0101; casein kinase 1, gamma 1	Multiple_Complex
TC0900009050.hg.1	18.68	17.35	2.52	0.0023	0.0088	EEF1A1P5	eukaryotic translation elongation factor 1 alph	Multiple_Complex

TC0100018453.hg.1	3.82	3.5	1.25	0.0023	0.0088	ARHGAP29	Rho GTPase activating protein 29	NonCoding
TC0600006779.hg.1	3.72	3.36	1.28	0.0023	0.0088	KU-MEL-3;	uncharacterized LOC497048; uncharacterized	Multiple_Complex
TC1500007753.hg.1	4.41	3.74	1.6	0.0023	0.0089	THSD4	Transcript Identified by AceView, Entrez Gene	Unassigned
TC1400008007.hg.1	8.38	8.78	-1.32	0.0023	0.0089	CPSF2	cleavage and polyadenylation specific factor 2	Multiple_Complex
TC1400010100.hg.1	6.2	6.83	-1.54	0.0023	0.0089	ATG2B	autophagy related 2B	Multiple_Complex
TC0X00009581.hg.1	6.68	7.21	-1.44	0.0023	0.0089	ELK1	ELK1, member of ETS oncogene family	Multiple_Complex
TC1600011551.hg.1	6.98	6.25	1.65	0.0023	0.0089	TK2	thymidine kinase 2, mitochondrial	Multiple_Complex
TC1900009429.hg.1	8.59	9.2	-1.52	0.0023	0.0089	KHSRP; MIF	KH-type splicing regulatory protein; microRNA	Multiple_Complex
TC2000007466.hg.1	4	3.54	1.38	0.0024	0.0089	KCNK15	potassium channel, two pore domain subfami	Multiple_Complex
TC0600012863.hg.1	6.7	7.34	-1.57	0.0024	0.0089	REV3L	REV3 like, DNA directed polymerase zeta catal	Multiple_Complex
TC1700009881.hg.1	10.89	11.45	-1.47	0.0024	0.0089	NCOR1	nuclear receptor corepressor 1	Multiple_Complex
TC0900007863.hg.1	5.93	5.25	1.6	0.0024	0.0089	CKS2	CDC28 protein kinase regulatory subunit 2	Multiple_Complex
TC1100013172.hg.1	4	4.74	-1.67	0.0024	0.0089	INTS5	integrator complex subunit 5	Multiple_Complex
TC1000009589.hg.1	6.1	6.87	-1.71	0.0024	0.0089	PITRM1	pitrilysin metallopeptidase 1	Multiple_Complex
TC0100014060.hg.1	6.2	6.72	-1.44	0.0024	0.009	ATPAF1	ATP synthase mitochondrial F1 complex assen	Multiple_Complex
TCUn_GL000218v100006	5.39	6.32	-1.91	0.0024	0.009	LOC389834	ankyrin repeat domain 57 pseudogene	Multiple_Complex
TC0500011448.hg.1	10.81	11.92	-2.17	0.0024	0.009	ARRDC3	arrestin domain containing 3	Multiple_Complex
TC0500012017.hg.1	12.61	11.85	1.7	0.0024	0.009	IRF1	interferon regulatory factor 1	Multiple_Complex
TC1100007805.hg.1	5.47	6.08	-1.53	0.0024	0.009	TMEM216	transmembrane protein 216	Multiple_Complex
TC0300010676.hg.1	7.93	8.68	-1.68	0.0024	0.009	CLASP2	cytoplasmic linker associated protein 2	Multiple_Complex
TC1200011310.hg.1	5.97	5.31	1.59	0.0024	0.009	PHLDA1	pleckstrin homology-like domain, family A, me	Multiple_Complex
TC0400012173.hg.1	4.72	5.71	-1.99	0.0024	0.009	SFRP2	secreted frizzled-related protein 2	Coding
TC0100015397.hg.1	10.97	10.43	1.46	0.0024	0.009	CD58	CD58 molecule	Multiple_Complex
TC1500007856.hg.1	9.49	10.51	-2.03	0.0024	0.009	CSK	c-src tyrosine kinase	Multiple_Complex
TC1700010662.hg.1	4.23	3.59	1.55	0.0024	0.009	KRTAP29-1	keratin associated protein 29-1	Coding
TC0200010726.hg.1	6.99	6.42	1.49	0.0024	0.0091	TMEM169	transmembrane protein 169	Coding
TC1000010602.hg.1	8.34	8.94	-1.52	0.0024	0.0091	ERCC6; ERC	excision repair cross-complementation group	Multiple_Complex
TSUnmapped00000674.h	5.98	5.29	1.61	0.0024	0.0091	CYP2D6	cytochrome P450, family 2, subfamily D, polyp	Coding
TC0300012881.hg.1	7.01	7.6	-1.51	0.0024	0.0091	SLC33A1	solute carrier family 33 (acetyl-CoA transporte	Multiple_Complex
TC1700008868.hg.1	7.95	8.54	-1.51	0.0024	0.0091	TMEM104	transmembrane protein 104	Multiple_Complex
TC1900009517.hg.1	4.33	3.96	1.29	0.0024	0.0091	FBN3	fibrillin 3	Multiple_Complex
TC1300009980.hg.1	4.54	5.09	-1.46	0.0024	0.0091	LMO7	LIM domain 7	Multiple_Complex
TC1000010926.hg.1	5.31	4.99	1.25	0.0024	0.0091	TBATA	thymus, brain and testes associated	Multiple_Complex
TC1700007099.hg.1	5.32	5.88	-1.48	0.0024	0.0091	DRG2	developmentally regulated GTP binding protei	Multiple_Complex
TC1200008920.hg.1	8.27	11.17	-7.45	0.0024	0.0091	OAS3	2-5-oligoadenylate synthetase 3	Multiple_Complex
TC1200006881.hg.1	7.17	8.02	-1.79	0.0024	0.0091	CREBL2	cAMP responsive element binding protein-like	Multiple_Complex
TC0600010211.hg.1	5.99	5.44	1.47	0.0024	0.0091	TTLL2	tubulin tyrosine ligase-like family member 2	Coding
TC0500010418.hg.1	10.01	10.47	-1.37	0.0024	0.0092	GOLPH3	golgi phosphoprotein 3 (coat-protein)	Multiple_Complex
TC0100011963.hg.1	8.23	7.59	1.56	0.0024	0.0092	SPRTN	SprT-like N-terminal domain	Multiple_Complex
TSUnmapped00000651.h	7.31	6.68	1.55	0.0024	0.0092	HNRNPCL1	heterogeneous nuclear ribonucleoprotein C-lil	Coding
TC0800012386.hg.1	6.32	6.79	-1.38	0.0024	0.0092	PINX1; MIR	PIN2/TERF1 interacting, telomerase inhibitor :	Multiple_Complex
TC0100018518.hg.1	7.56	8.01	-1.37	0.0024	0.0092	PEX19	peroxisomal biogenesis factor 19	Multiple_Complex
TC1400007466.hg.1	8.9	9.48	-1.49	0.0025	0.0092	FUT8	fucosyltransferase 8 (alpha (1,6) fucosyltransf	Multiple_Complex
TC1500010383.hg.1	5.16	5.74	-1.5	0.0025	0.0092	POLG	polymerase (DNA directed), gamma	Multiple_Complex
TC1000008234.hg.1	4.87	5.63	-1.69	0.0025	0.0092	FAM213A	family with sequence similarity 213, member .	Multiple_Complex
TC0200008262.hg.1	5.05	4.75	1.23	0.0025	0.0092	RNF181	ring finger protein 181	Multiple_Complex
TC1300007237.hg.1	5.82	6.42	-1.52	0.0025	0.0092	ALG11; UTF	ALG11, alpha-1,2-mannosyltransferase; UTP14	Multiple_Complex
TC0200011171.hg.1	5.1	4.78	1.25	0.0025	0.0092	SH3BP4	SH3-domain binding protein 4	Multiple_Complex
TC0500013425.hg.1	5.43	5.93	-1.42	0.0025	0.0093	C5orf45	chromosome 5 open reading frame 45	Multiple_Complex
TC0500007179.hg.1	5.51	6.19	-1.6	0.0025	0.0093	WDR70	WD repeat domain 70	Multiple_Complex
TC1900011708.hg.1	5.87	6.71	-1.79	0.0025	0.0093	PDCD2L	programmed cell death 2-like	Multiple_Complex
TC1100012917.hg.1	6.26	6.58	-1.25	0.0025	0.0093	NCAPD3	non-SMC condensin II complex subunit D3	Multiple_Complex
TC2100008382.hg.1	7.17	6.58	1.5	0.0025	0.0093	KRTAP12-1	keratin associated protein 12-1	Coding
TC1900008827.hg.1	5.93	5.44	1.41	0.0025	0.0093	CACNG8; M	calcium channel, voltage-dependent, gamma :	Multiple_Complex
TC0300010962.hg.1	6.16	5.58	1.49	0.0025	0.0093	MYL3	myosin, light chain 3, alkali; ventricular, skelet	Coding
TC1000011352.hg.1	4.86	4.21	1.56	0.0025	0.0093	KLLN	killin, p53-regulated DNA replication inhibitor	Coding
TC0100010133.hg.1	7.08	7.69	-1.53	0.0025	0.0093	MSTO1; MS	misato 1, mitochondrial distribution and morp	Multiple_Complex
TC0600011478.hg.1	6.12	5.44	1.6	0.0025	0.0094	FKBPL	FK506 binding protein like	Coding
TC0400012856.hg.1	7.25	6.62	1.54	0.0025	0.0094	RAPGEF2	Rap guanine nucleotide exchange factor 2	NonCoding
TC1900011959.hg.1	8.64	6.65	3.98	0.0025	0.0094	CEACAM8	carcinoembryonic antigen-related cell adhesic	Coding
TC1300009714.hg.1	10.41	9.3	2.17	0.0025	0.0094	ARGLU1	arginine and glutamate rich 1	Multiple_Complex
TSUnmapped00000483.h	7.58	7.2	1.31	0.0025	0.0094	RPS6KA1	ribosomal protein S6 kinase, 90kDa, polypepti	Coding
TC1900011640.hg.1	4.51	3.97	1.45	0.0025	0.0094	EFNA2	ephrin-A2	Multiple_Complex
TC1000008665.hg.1	4.35	4.92	-1.48	0.0025	0.0095	C10orf2	chromosome 10 open reading frame 2	Multiple_Complex
TC0700008073.hg.1	8.47	7.47	2	0.0025	0.0095	POR; MIR4(	P450 (cytochrome) oxidoreductase; microRNA	Multiple_Complex
TC0100006994.hg.1	9.02	8.58	1.36	0.0025	0.0095	PLEKHM2	pleckstrin homology domain containing, famil	Multiple_Complex
TC0100012329.hg.1	7.93	9.1	-2.24	0.0025	0.0095	OR2W3	olfactory receptor, family 2, subfamily W, mer	Coding
TC2200008621.hg.1	5.44	4.82	1.54	0.0025	0.0095	PVALB	parvalbumin	Multiple_Complex
TC0700013620.hg.1	10	9.14	1.82	0.0025	0.0095	IMPDH1	IMP (inosine 5-monophosphate) dehydrogena	Multiple_Complex
TC0100018200.hg.1	5.37	6.16	-1.73	0.0026	0.0095	SEPN1	selenoprotein N, 1	Multiple_Complex

TC0200010894.hg.1	5.21	4.63	1.5	0.0026	0.0096	CCDC140	coiled-coil domain containing 140	Coding
TC1700008938.hg.1	9.21	8.64	1.48	0.0026	0.0096	UBALD2	UBA-like domain containing 2	Multiple_Complex
TC2200009121.hg.1	7.69	8.13	-1.36	0.0026	0.0096	BRD1	bromodomain containing 1	Multiple_Complex
TC0200007237.hg.1	6.39	6.84	-1.37	0.0026	0.0096	CRIM1	cysteine rich transmembrane BMP regulator 1	Multiple_Complex
TC0700012099.hg.1	10.48	9.91	1.48	0.0026	0.0096	RASA4	Salzman2013 ALT_ACCEPTOR, ALT_DONOR, c	NonCoding
TC0500008232.hg.1	9.57	10.78	-2.3	0.0026	0.0097	PPIP5K2	diphosphoinositol pentakisphosphate kinase 2	Multiple_Complex
TC0500008917.hg.1	5.86	6.59	-1.65	0.0026	0.0097	KIAA0141	KIAA0141	Multiple_Complex
TC1500010764.hg.1	10.18	9.64	1.45	0.0026	0.0097	CLK3	CDC like kinase 3	Multiple_Complex
TC1400008665.hg.1	11.95	11.05	1.87	0.0026	0.0097	DAD1	defender against cell death 1	Multiple_Complex
TC0600007629.hg.1	5.22	4.56	1.58	0.0026	0.0097	CYP21A2	cytochrome P450, family 21, subfamily A, poly	Multiple_Complex
TC1500009845.hg.1	8.7	7.56	2.2	0.0026	0.0097	AAGAB	alpha- and gamma-adaptin binding protein	Multiple_Complex
TC1400009973.hg.1	7.38	8.07	-1.61	0.0026	0.0097	RPS6KA5	ribosomal protein S6 kinase, 90kDa, polypepti	Multiple_Complex
TC0500007415.hg.1	4	3.57	1.35	0.0026	0.0097	GPX8	glutathione peroxidase 8 (putative)	Multiple_Complex
TC1400007033.hg.1	7.24	8.21	-1.96	0.0026	0.0098	FAM179B	family with sequence similarity 179, member	Multiple_Complex
TC1300008998.hg.1	7.87	9.02	-2.21	0.0026	0.0098	LINC00282	long intergenic non-protein coding RNA 282	NonCoding
TC0300013146.hg.1	11.64	13.36	-3.29	0.0026	0.0098	TNFSF10	tumor necrosis factor (ligand) superfamily, me	Multiple_Complex
TC0700006634.hg.1	5.15	5.82	-1.6	0.0026	0.0098	AIMP2	aminoacyl tRNA synthetase complex-interacti	Multiple_Complex
TC0400006787.hg.1	4.57	4.09	1.4	0.0026	0.0098	USP17L12	ubiquitin specific peptidase 17-like family mer	Coding
TC1800009278.hg.1	5.25	4.8	1.37	0.0026	0.0099	TCEB3CL	transcription elongation factor B polypeptide	Coding
TC0100018435.hg.1	6.76	7.1	-1.26	0.0027	0.0099	EBNA1BP2;	EBNA1 binding protein 2; microRNA 6733	Multiple_Complex
TC1000008005.hg.1	8.34	7.5	1.79	0.0027	0.0099	MCU	mitochondrial calcium uniporter	Multiple_Complex
TC0600007460.hg.1	4.77	4.05	1.65	0.0027	0.0099	OR12D1	olfactory receptor, family 12, subfamily D, me	Pseudogene
TC0100012460.hg.1	7.54	6.92	1.53	0.0027	0.0099	UBE2J2	Transcript Identified by AceView, Entrez Gene	Unassigned
TC1100013035.hg.1	4.83	4.39	1.35	0.0027	0.0099	PPP2R5B	protein phosphatase 2, regulatory subunit B, b	NonCoding
TSUnmapped00000344.h	8.15	8.65	-1.42	0.0027	0.01	FBL	fibrillarin	Coding
TC0500009623.hg.1	5.2	4.83	1.3	0.0027	0.01	PRR7	proline rich 7 (synaptic)	Coding
TC0800008198.hg.1	3.62	4.3	-1.6	0.0027	0.01	OTUD6B	Transcript Identified by AceView, Entrez Gene	Unassigned
TC1100009997.hg.1	5.75	5.16	1.5	0.0027	0.01	ZNF214	zinc finger protein 214	Multiple_Complex
TC0800007309.hg.1	9.38	8.77	1.53	0.0027	0.01	PROSC	proline synthetase co-transcribed homolog (b	Multiple_Complex
TC1600011383.hg.1	9.18	10.15	-1.96	0.0027	0.01	TMEM265;	transmembrane protein 265; Snf2-related CRE	Multiple_Complex
TC0400011706.hg.1	5.53	4.65	1.84	0.0027	0.0101	FABP2	fatty acid binding protein 2, intestinal	Coding
TC0400010745.hg.1	6.23	6.5	-1.21	0.0027	0.0101	CLOCK	clock circadian regulator	Multiple_Complex
TC0500007681.hg.1	5.53	6.44	-1.89	0.0027	0.0101	OCLN	occludin	Multiple_Complex
TC0200016718.hg.1	5.35	4.6	1.69	0.0027	0.0102	TBC1D8	TBC1 domain family, member 8 (with GRAM d	NonCoding
TC0700008928.hg.1	5.83	6.73	-1.86	0.0027	0.0102	CPED1	cadherin-like and PC-esterase domain contain	Multiple_Complex
TC1900006856.hg.1	6.33	5.94	1.32	0.0027	0.0102	CLEC4M	C-type lectin domain family 4, member M	Multiple_Complex
TC1900008655.hg.1	4.77	5.46	-1.61	0.0027	0.0102	ZNF175	zinc finger protein 175	Multiple_Complex
TC1100007964.hg.1	5.53	6.1	-1.48	0.0028	0.0103	SLC22A11	solute carrier family 22 (organic anion/urate t	Multiple_Complex
TC2200007976.hg.1	5.57	5.93	-1.29	0.0028	0.0103	SLC25A1	solute carrier family 25 (mitochondrial carrier	Multiple_Complex
TC1900007325.hg.1	7.18	8.03	-1.81	0.0028	0.0103	MVB12A; B	multivesicular body subunit 12A; BST2 interfe	Multiple_Complex
TC1500010368.hg.1	5.58	5.96	-1.3	0.0028	0.0103	HAPLN3	hyaluronan and proteoglycan link protein 3	Multiple_Complex
TC0500009264.hg.1	4.81	4.31	1.41	0.0028	0.0103	ADRA1B	adrenoceptor alpha 1B	Coding
TC0100013881.hg.1	5.24	4.81	1.35	0.0028	0.0103	GUCA2A	guanylate cyclase activator 2A (guanylin)	Coding
TC1000008995.hg.1	4.53	3.91	1.54	0.0028	0.0103	ATRNL1	attractin-like 1	Multiple_Complex
TC0X00008097.hg.1	7.69	6.86	1.78	0.0028	0.0103	NXT2	nuclear transport factor 2-like export factor 2	Coding
TC0X00006955.hg.1	5.23	4.59	1.56	0.0028	0.0103	LANCL3	LanC lantibiotic synthetase component C-like	Coding
TC2000008499.hg.1	7.13	6.57	1.47	0.0028	0.0103	RRBP1	ribosome binding protein 1	Multiple_Complex
TC1900008600.hg.1	4.87	4.45	1.34	0.0028	0.0104	GPR32	G protein-coupled receptor 32	Coding
TC1200009497.hg.1	5.87	5.31	1.48	0.0028	0.0104	P2RX2	purinergic receptor P2X, ligand gated ion char	Coding
TC1300007074.hg.1	4.38	3.9	1.4	0.0028	0.0104	SPERT	spermatid associated	Coding
TC1400010714.hg.1	8.66	7.48	2.26	0.0028	0.0104	HAUS4; MI	HAUS augmin like complex subunit 4; microRN	Multiple_Complex
TC0300007392.hg.1	4.8	4.36	1.35	0.0028	0.0104	FAM212A	family with sequence similarity 212, member	Coding
TC2000006580.hg.1	6.74	5.69	2.07	0.0028	0.0104	RASSF2	Memczak2013 ANTISENSE, coding, INTERNAL,	NonCoding
TC0100010546.hg.1	9.37	8	2.58	0.0028	0.0104	BLZF1	basic leucine zipper nuclear factor 1	Multiple_Complex
TC1200007847.hg.1	4.49	4.26	1.18	0.0028	0.0104	HSD17B6	hydroxysteroid (17-beta) dehydrogenase 6	Multiple_Complex
TC0300006687.hg.1	7.26	7.72	-1.38	0.0028	0.0104	CCDC174	coiled-coil domain containing 174	Multiple_Complex
TC0300013635.hg.1	12.35	11.61	1.68	0.0028	0.0105	ACAP2	ArfGAP with coiled-coil, ankyrin repeat and PH	Multiple_Complex
TC1100007390.hg.1	7.11	7.67	-1.48	0.0028	0.0105	EXT2	exostosin glycosyltransferase 2	Multiple_Complex
TC0600009095.hg.1	8.38	7.54	1.79	0.0028	0.0105	MICAL1	Jeck2013 ANTISENSE, coding, INTERNAL, intro	NonCoding
TC1100010893.hg.1	5.54	6.15	-1.53	0.0028	0.0105	SLC43A1	solute carrier family 43 (amino acid system L t	Multiple_Complex
TC0200014821.hg.1	4.45	4.04	1.33	0.0028	0.0105	SCN7A	sodium channel, voltage gated, type VII alpha	Multiple_Complex
TC1100009799.hg.1	4.3	4.67	-1.3	0.0028	0.0105	TRPM5	transient receptor potential cation channel, su	Coding
TC0900007321.hg.1	5.7	5.2	1.41	0.0028	0.0105	SPATA31A5	SPATA31 subfamily A, member 5; SPATA31 su	Multiple_Complex
TC0100008599.hg.1	4.15	3.63	1.43	0.0028	0.0105	UBE2U	ubiquitin-conjugating enzyme E2U (putative)	Multiple_Complex
TC0100015957.hg.1	8.09	8.57	-1.39	0.0028	0.0105	ASH1L; MIF	ash1 (absent, small, or homeotic)-like (Drosop	Multiple_Complex
TC1000008750.hg.1	9.69	10.18	-1.4	0.0028	0.0105	WBP1L	WW domain binding protein 1-like	Multiple_Complex
TC2200008609.hg.1	7.12	7.49	-1.29	0.0028	0.0105	TXN2	thioredoxin 2	Multiple_Complex
TC0100013134.hg.1	14.58	15.08	-1.41	0.0028	0.0105	CAPZB	capping protein (actin filament) muscle Z-line,	Multiple_Complex
TSUnmapped00000776.h	7.91	8.54	-1.55	0.0028	0.0105	FBL	fibrillarin	Coding
TC0400012818.hg.1	12.68	11.54	2.2	0.0029	0.0105	CCNG2	cyclin G2	Multiple_Complex

TC0700011710.hg.1	9.14	10.03	-1.85	0.0029	0.0105	SLC25A40	solute carrier family 25, member 40	Multiple_Complex
TC0900011978.hg.1	4.38	4.87	-1.4	0.0029	0.0106	SDCCAG3	serologically defined colon cancer antigen 3	Multiple_Complex
TC0700009692.hg.1	6.44	6.92	-1.4	0.0029	0.0106	SLC4A2	solute carrier family 4 (anion exchanger), mem	Multiple_Complex
TC1000011577.hg.1	5.9	6.45	-1.46	0.0029	0.0106	PYROXD2; I	pyridine nucleotide-disulphide oxidoreductase	Multiple_Complex
TC0900008860.hg.1	6.25	6.72	-1.38	0.0029	0.0106	SLC27A4	solute carrier family 27 (fatty acid transporter	Multiple_Complex
TC2000006861.hg.1	6.93	7.95	-2.03	0.0029	0.0106	RIN2	Ras and Rab interactor 2	Multiple_Complex
TC1600009210.hg.1	5.16	5.77	-1.52	0.0029	0.0106	ADCY9	adenylate cyclase 9	Multiple_Complex
TC0300010968.hg.1	9.57	9.09	1.4	0.0029	0.0106	SETD2	SET domain containing 2	Multiple_Complex
TC1700010293.hg.1	8.58	9.34	-1.7	0.0029	0.0106	CRLF3	cytokine receptor-like factor 3	Multiple_Complex
TC0500008107.hg.1	6.65	5.89	1.69	0.0029	0.0106	SLF1	SMC5-SMC6 complex localization factor 1	Multiple_Complex
TC0400011580.hg.1	5.54	5	1.45	0.0029	0.0106	CFI	complement factor I	Multiple_Complex
TC1100009911.hg.1	3.81	3.39	1.34	0.0029	0.0107	OR52A5	olfactory receptor, family 52, subfamily A, me	Coding
TC1200009076.hg.1	7.2	6.28	1.89	0.0029	0.0107	SUDS3	SDS3 homolog, SIN3A corepressor complex co	Multiple_Complex
TC1700011261.hg.1	6.08	5.26	1.76	0.0029	0.0107	4-Sep	septin 4	Multiple_Complex
TC1100013224.hg.1	7.54	8.27	-1.66	0.0029	0.0107	POU2AF1	POU class 2 associating factor 1	Multiple_Complex
TC1900009087.hg.1	4.35	3.95	1.33	0.0029	0.0107	ODF3L2	outer dense fiber of sperm tails 3-like 2	Multiple_Complex
TC1600011048.hg.1	5.69	6.13	-1.36	0.0029	0.0107	TAF1C	TATA box binding protein (TBP)-associated fac	Multiple_Complex
TC1900010482.hg.1	6.92	6.34	1.5	0.0029	0.0108	ATP4A	ATPase, H+/K+ exchanging, alpha polypeptide	Multiple_Complex
TC0300012171.hg.1	8.29	8.88	-1.51	0.0029	0.0108	IQCB1	IQ motif containing B1	Multiple_Complex
TC0400007868.hg.1	4	4.75	-1.67	0.0029	0.0108	PARM1	prostate androgen-regulated mucin-like prote	Multiple_Complex
TC1600011518.hg.1	4.38	3.91	1.38	0.0029	0.0108	DOC2A	double C2-like domains, alpha	Multiple_Complex
TC2100007896.hg.1	3.78	3.31	1.38	0.0029	0.0108	KRTAP19-2	keratin associated protein 19-2	Coding
TC0900012025.hg.1	6.04	5.59	1.37	0.0029	0.0108	ABCA2	ATP binding cassette subfamily A member 2	Multiple_Complex
TC0700007132.hg.1	6.71	5.99	1.65	0.0029	0.0108	FKBP9	FK506 binding protein 9	Multiple_Complex
TC0600012540.hg.1	5.18	4.53	1.56	0.0029	0.0108	GABRR2	gamma-aminobutyric acid (GABA) A receptor,	Multiple_Complex
TC0400012900.hg.1	13.46	12.47	1.99	0.003	0.0109	FBXL5	F-box and leucine-rich repeat protein 5	Multiple_Complex
TC0100018389.hg.1	10.5	9.74	1.69	0.003	0.0109	CDK11A	cyclin-dependent kinase 11A	Multiple_Complex
TC2000008808.hg.1	4.68	4.24	1.36	0.003	0.0109	DEFB121	defensin, beta 121	Multiple_Complex
TC0400008347.hg.1	6.17	6.52	-1.27	0.003	0.0109	CYP2U1	cytochrome P450, family 2, subfamily U, polyp	Multiple_Complex
TC1300006811.hg.1	7.25	7.95	-1.62	0.003	0.0109	PDS5B	Memczak2013 ALT_ACCEPTOR, ALT_DONOR,	NonCoding
TC0800011533.hg.1	5.46	6.01	-1.47	0.003	0.0109	TRPS1	Transcript Identified by AceView, Entrez Gene	Unassigned
TC0400011882.hg.1	5.89	5.37	1.44	0.003	0.0109	PABPC4L	poly(A) binding protein, cytoplasmic 4-like	Coding
TC1900008516.hg.1	4.82	4.46	1.28	0.003	0.0109	CGB5; CGB	chorionic gonadotropin, beta polypeptide 5; c	Coding
TC0400011015.hg.1	6.49	5.79	1.62	0.003	0.0109	CXCL3	chemokine (C-X-C motif) ligand 3	Multiple_Complex
TC0600007203.hg.1	7.26	6.34	1.89	0.003	0.0109	ACOT13	acyl-CoA thioesterase 13	Multiple_Complex
TC0900012292.hg.1	4.22	3.85	1.29	0.003	0.0109	LRRC26	leucine rich repeat containing 26	Coding
TC0900010958.hg.1	7.98	8.5	-1.43	0.003	0.0109	TRMO	tRNA methyltransferase O	Multiple_Complex
TC1200007829.hg.1	9.53	10.55	-2.03	0.003	0.011	SMARCC2	Memczak2013 ANTISENSE, CDS, coding, INTEF	NonCoding
TC0900008277.hg.1	5.88	5.46	1.34	0.003	0.011	CYLC2	cylicin, basic protein of sperm head cytoskelet	Multiple_Complex
TC1700008418.hg.1	10.17	9.37	1.75	0.003	0.011	DYNLL2	dynein, light chain, LC8-type 2	Multiple_Complex
TC1900011764.hg.1	5.78	6.21	-1.35	0.003	0.011	GEMIN7	gem nuclear organelle associated protein 7	Multiple_Complex
TC0100013272.hg.1	4.77	4.34	1.35	0.003	0.011	HTR1D	5-hydroxytryptamine (serotonin) receptor 1D,	Coding
TC0300009613.hg.1	4.17	4.74	-1.48	0.003	0.011	USP13	ubiquitin specific peptidase 13 (isopeptidase T	Multiple_Complex
TC0500011943.hg.1	6.08	6.72	-1.56	0.003	0.011	C5orf63	chromosome 5 open reading frame 63	Multiple_Complex
TC1800006715.hg.1	8.63	7.79	1.79	0.003	0.011	IMPA2	inositol(myo)-1(or 4)-monophosphatase 2	Multiple_Complex
TC1200009767.hg.1	9.22	9.87	-1.56	0.003	0.011	PHB2; SCAI	prohibitin 2; small Cajal body-specific RNA 12	Multiple_Complex
TC1200009250.hg.1	6.19	6.86	-1.59	0.003	0.0111	GTF2H3	general transcription factor IIH subunit 3	Multiple_Complex
TC1600009074.hg.1	15.56	14.32	2.35	0.003	0.0111	MSRB1	methionine sulfoxide reductase B1	Multiple_Complex
TC2100007491.hg.1	5.35	4.74	1.52	0.003	0.0111	CBS	cystathionine-beta-synthase	Multiple_Complex
TC0600008229.hg.1	5.12	4.63	1.4	0.003	0.0111	TFAP2D	transcription factor AP-2 delta (activating enh	Multiple_Complex
TC1700009995.hg.1	4.97	5.9	-1.9	0.003	0.0111	GRAP	GRB2-related adaptor protein	Multiple_Complex
TC0300012670.hg.1	4.8	5.3	-1.41	0.003	0.0111	ATR	ATR serine/threonine kinase	Multiple_Complex
TC1900011751.hg.1	8.97	9.57	-1.52	0.003	0.0111	ZNF222	zinc finger protein 222	Multiple_Complex
TC0200011020.hg.1	8.97	9.88	-1.89	0.0031	0.0112	SP140	SP140 nuclear body protein	Multiple_Complex
TC0100012544.hg.1	4.45	3.98	1.39	0.0031	0.0112	MMEL1	membrane metallo-endopeptidase-like 1	Multiple_Complex
TC1900012006.hg.1	6.43	6.8	-1.29	0.0031	0.0112	AKT1S1	AKT1 substrate 1 (proline rich)	Multiple_Complex
TC0100016597.hg.1	9.54	8.6	1.91	0.0031	0.0112	STX6	syntaxin 6	Multiple_Complex
TC1500010921.hg.1	6.49	7.05	-1.48	0.0031	0.0112	MRPL46	mitochondrial ribosomal protein L46	Multiple_Complex
TC1900009039.hg.1	5.62	6.09	-1.39	0.0031	0.0112	ZSCAN22; N	zinc finger and SCAN domain containing 22; m	Multiple_Complex
TC0800009641.hg.1	9.2	9.69	-1.4	0.0031	0.0112	FAM86B1	family with sequence similarity 86, member B	Multiple_Complex
TSUnmapped00000394.h	4.76	5.49	-1.65	0.0031	0.0112	ATG16L1	autophagy related 16-like 1	Coding
TC0200015720.hg.1	4.41	4.85	-1.35	0.0031	0.0112	AAMP	angio-associated migratory cell protein	Multiple_Complex
TC1900007787.hg.1	5.35	4.94	1.32	0.0031	0.0112	KCTD15	potassium channel tetramerization domain co	Multiple_Complex
TC0X00009645.hg.1	8.32	7.86	1.38	0.0031	0.0113	GRIPAP1	GRIP1 associated protein 1	Multiple_Complex
TC0200008059.hg.1	8.05	8.88	-1.77	0.0031	0.0113	STAMBP	STAM binding protein	Multiple_Complex
TC2100008061.hg.1	6.47	6.89	-1.34	0.0031	0.0113	SETD4	SET domain containing 4	Multiple_Complex
TC1100013048.hg.1	6.91	7.33	-1.34	0.0031	0.0113	DPP3	dipeptidyl-peptidase 3	Multiple_Complex
TC0600013780.hg.1	5.53	6.17	-1.56	0.0031	0.0114	LPAL2	lipoprotein, Lp(a)-like 2, pseudogene	Multiple_Complex
TC0200007184.hg.1	9.75	10.25	-1.42	0.0031	0.0114	BIRC6	baculoviral IAP repeat containing 6	Multiple_Complex
TC1100007767.hg.1	6.37	5.63	1.67	0.0031	0.0114	MS4A4A	membrane-spanning 4-domains, subfamily A,	Multiple_Complex

TC1400006853.hg.1	3.6	4.33	-1.66	0.0031	0.0114	AKAP6	A kinase (PRKA) anchor protein 6	Multiple_Complex
TC1100009926.hg.1	4.66	4.13	1.44	0.0031	0.0114	OR51B2	olfactory receptor, family 51, subfamily B, me	Multiple_Complex
TC0200015348.hg.1	9.84	8.92	1.9	0.0031	0.0114	SF3B1	splicing factor 3b, subunit 1, 155kDa	Multiple_Complex
TC1000007333.hg.1	9.01	9.65	-1.57	0.0031	0.0114	ZNF33A	zinc finger protein 33A	Multiple_Complex
TC0X00010033.hg.1	5.23	5.49	-1.2	0.0031	0.0114	HDAC8	histone deacetylase 8	Multiple_Complex
TC1700010635.hg.1	4.73	4.15	1.49	0.0031	0.0114	KRT24	keratin 24, type I	Multiple_Complex
TC1500006683.hg.1	8.34	8.77	-1.35	0.0031	0.0114	APBA2	amyloid beta (A4) precursor protein-binding, f	Multiple_Complex
TC0300008746.hg.1	7.17	7.49	-1.25	0.0031	0.0114	ACAD9	acyl-CoA dehydrogenase family, member 9	Multiple_Complex
TC0500008602.hg.1	7.51	6.77	1.67	0.0031	0.0114	LYRM7	LYR motif containing 7	Multiple_Complex
TC1200010618.hg.1	13.99	13.21	1.72	0.0031	0.0115	TUBA1A	tubulin, alpha 1a	Multiple_Complex
TC0900010971.hg.1	5.99	6.77	-1.72	0.0031	0.0115	TBC1D2	TBC1 domain family, member 2	Multiple_Complex
TC0600008319.hg.1	3.85	3.38	1.38	0.0031	0.0115	FAM83B	family with sequence similarity 83, member B	Multiple_Complex
TC1500009996.hg.1	6.74	6.36	1.29	0.0031	0.0115	STOML1	stomatin (EPB72)-like 1	Multiple_Complex
TC1000008423.hg.1	6.66	7.3	-1.57	0.0032	0.0115	RPP30	ribonuclease P/MRP 30kDa subunit	Multiple_Complex
TC2200007876.hg.1	4.62	4.32	1.23	0.0032	0.0115	XKR3	X-linked Kx blood group related 3	Multiple_Complex
TC0200008911.hg.1	6.21	6.84	-1.54	0.0032	0.0115	ZC3H6	zinc finger CCCH-type containing 6	Multiple_Complex
TC0300009468.hg.1	7.4	8.15	-1.67	0.0032	0.0116	PRKCI	protein kinase C, iota	Multiple_Complex
TC0X00011204.hg.1	6.64	7	-1.29	0.0032	0.0116	LAGE3	L antigen family, member 3	Coding
TC2000008587.hg.1	10.67	9.4	2.41	0.0032	0.0116	RALGAPA2	Ral GTPase activating protein, alpha subunit 2	Multiple_Complex
TC0600010807.hg.1	4.47	4.15	1.25	0.0032	0.0116	ERVFRD-1	endogenous retrovirus group FRD, member 1	Coding
TC0100018507.hg.1	10.32	11.08	-1.69	0.0032	0.0117	ARHGEF2	Rho/Rac guanine nucleotide exchange factor 2	Multiple_Complex
TC0100013305.hg.1	6.39	6.91	-1.43	0.0032	0.0117	FUCA1	fucosidase, alpha-L- 1, tissue	Coding
TC0600014110.hg.1	11.91	12.91	-2	0.0032	0.0117	PSMB9	proteasome subunit beta 9	Multiple_Complex
TC1100007598.hg.1	4.29	3.77	1.43	0.0032	0.0117	OR4C16	olfactory receptor, family 4, subfamily C, mem	Multiple_Complex
TC1800006493.hg.1	6.78	6.33	1.36	0.0032	0.0117	EMILIN2	elastin microfibril interfacer 2	Multiple_Complex
TC1500007961.hg.1	3.98	3.57	1.33	0.0032	0.0117	SH2D7	SH2 domain containing 7	Coding
TC1400007712.hg.1	7.73	7.05	1.61	0.0032	0.0117	BATF	basic leucine zipper transcription factor, ATF-l	Multiple_Complex
TC0900009931.hg.1	6.53	6.86	-1.25	0.0032	0.0118	PIGO	phosphatidylinositol glycan anchor biosynthes	Multiple_Complex
TC1200006782.hg.1	6.8	6.14	1.58	0.0033	0.0118	CLEC9A	C-type lectin domain family 9, member A	Multiple_Complex
TC2200006713.hg.1	12.38	11.81	1.49	0.0033	0.0119	UBE2L3	ubiquitin conjugating enzyme E2L 3	Multiple_Complex
TC1900008692.hg.1	6.39	6.93	-1.45	0.0033	0.0119	ZNF480	zinc finger protein 480	Multiple_Complex
TC1600007217.hg.1	5.79	5.48	1.24	0.0033	0.0119	SCNN1B	sodium channel, non voltage gated 1 beta sub	Multiple_Complex
TC2000006823.hg.1	4.53	5.16	-1.55	0.0033	0.0119	ZNF133	zinc finger protein 133	Multiple_Complex
TC1100006998.hg.1	8.85	9.48	-1.55	0.0033	0.0119	GTF2H1	general transcription factor IIH subunit 1	Multiple_Complex
TC1200010597.hg.1	8.85	9.4	-1.46	0.0033	0.0119	DDX23	DEAD (Asp-Glu-Ala-Asp) box polypeptide 23	Multiple_Complex
TC0700009462.hg.1	8.1	7.43	1.59	0.0033	0.0119	MTRNR2L6	MT-RNR2-like 6	Coding
TC1900007063.hg.1	4.15	3.78	1.29	0.0033	0.0119	ZNF788	zinc finger family member 788	Multiple_Complex
TC0800008940.hg.1	10.86	10.36	1.41	0.0033	0.0119	PHF20L1	PHD finger protein 20-like 1	Multiple_Complex
TC1700012449.hg.1	5.97	5.49	1.39	0.0033	0.0119	CSHL1	chorionic somatomammotropin hormone-like	Multiple_Complex
TC0900012290.hg.1	6.67	6.2	1.39	0.0033	0.0119	FUT7	fucosyltransferase 7 (alpha (1,3) fucosyltransf	Coding
TC0100017451.hg.1	10.03	9.4	1.55	0.0033	0.0119	TP53BP2	tumor protein p53 binding protein 2	Multiple_Complex
TC0200014402.hg.1	5.81	5.26	1.46	0.0033	0.0119	LCT	lactase	Coding
TC1600010798.hg.1	5.23	4.69	1.45	0.0033	0.0119	ZNF821	zinc finger protein 821	Multiple_Complex
TC1700010222.hg.1	10.95	10.19	1.69	0.0033	0.012	PHF12	PHD finger protein 12	Multiple_Complex
TC0800009166.hg.1	3.57	3.14	1.35	0.0033	0.012	GML	glycosylphosphatidylinositol anchored molecu	Coding
TC1200012159.hg.1	5.16	4.56	1.51	0.0033	0.012	MSI1	musashi RNA binding protein 1	Multiple_Complex
TC2000007792.hg.1	6.38	5.76	1.53	0.0033	0.0121	PFDN4	prefoldin subunit 4	Multiple_Complex
TC0500010924.hg.1	4.73	4.31	1.34	0.0033	0.0121	ADAMTS6	ADAM metallopeptidase with thrombospondi	Multiple_Complex
TC0400010132.hg.1	9.52	10.06	-1.45	0.0033	0.0121	TAPT1	transmembrane anterior posterior transforma	Multiple_Complex
TC0500011228.hg.1	7.56	8.11	-1.46	0.0033	0.0121	ARSB	arylsulfatase B	Multiple_Complex
TC0600011697.hg.1	5.37	5.88	-1.42	0.0034	0.0122	CCDC167	coiled-coil domain containing 167	Coding
TC0300009916.hg.1	5.49	4.88	1.53	0.0034	0.0122	HES1	hes family bHLH transcription factor 1	Multiple_Complex
TC1100011048.hg.1	9.67	8.74	1.9	0.0034	0.0122	TMEM258;	transmembrane protein 258; microRNA 611	Multiple_Complex
TC0X00007236.hg.1	4.23	3.64	1.5	0.0034	0.0122	GAGE12C; (	G antigen 12C; G antigen 12E; G antigen 12H	Coding
TC0800006887.hg.1	4.83	5.19	-1.28	0.0034	0.0122	NAT1	N-acetyltransferase 1 (arylamine N-acetyltran	Multiple_Complex
TC0600009508.hg.1	9.63	8.76	1.82	0.0034	0.0122	TBPL1	TBP-like 1	Multiple_Complex
TC0500012879.hg.1	6.49	6.93	-1.36	0.0034	0.0123	BOD1	biorientation of chromosomes in cell division	Multiple_Complex
TC1400010721.hg.1	8.54	7.87	1.59	0.0034	0.0123	PSME2; MI	proteasome activator subunit 2; microRNA 77	Multiple_Complex
TC1100006815.hg.1	6.51	7.24	-1.66	0.0034	0.0123	WEE1	WEE1 G2 checkpoint kinase	Multiple_Complex
TC1200006654.hg.1	5.12	5.51	-1.31	0.0034	0.0123	ATN1	atrophin 1	Multiple_Complex
TC1900007330.hg.1	5.19	5.69	-1.41	0.0034	0.0123	FAM129C	family with sequence similarity 129, member (	Multiple_Complex
TC1900011956.hg.1	4.17	3.54	1.55	0.0034	0.0124	CYP2A7	cytochrome P450, family 2, subfamily A, polyp	Multiple_Complex
TC1600009957.hg.1	6.84	7.45	-1.52	0.0034	0.0124	LOC388242	Homo sapiens coiled-coil domain containing 1	Multiple_Complex
TC0700013368.hg.1	6.96	7.49	-1.45	0.0034	0.0124	ZNF736	zinc finger protein 736	Multiple_Complex
TC1900012038.hg.1	7.31	6.68	1.54	0.0034	0.0124	LILRA6	leukocyte immunoglobulin-like receptor, subf	Multiple_Complex
TC1400006486.hg.1	4.59	4	1.5	0.0034	0.0124	OR4M1	olfactory receptor, family 4, subfamily M, mer	Coding
TC1900009022.hg.1	4.8	5.43	-1.55	0.0034	0.0124	ZNF135	zinc finger protein 135	Multiple_Complex
TC0700011050.hg.1	4.38	5.06	-1.59	0.0035	0.0125	FIGNL1	fidgetin-like 1	Multiple_Complex
TC0200012742.hg.1	11.49	10.37	2.17	0.0035	0.0125	XPO1	exportin 1	Multiple_Complex
TC1300008424.hg.1	8.4	9.14	-1.67	0.0035	0.0125	USP12	ubiquitin specific peptidase 12	Multiple_Complex

TC1600008940.hg.1	9.65	9.04	1.53	0.0035	0.0125	AXIN1	Memczak2013 ALT_ACCEPTOR, ALT_DONOR,	NonCoding
TC0200009813.hg.1	6.98	6.51	1.39	0.0035	0.0125	PSMD14	proteasome 26S subunit, non-ATPase 14	Multiple_Complex
TC1100011107.hg.1	7.3	7.71	-1.34	0.0035	0.0125	WDR74	WD repeat domain 74	Multiple_Complex
TC1700012469.hg.1	13.49	14.13	-1.56	0.0035	0.0125	SUMO2	small ubiquitin-like modifier 2	Multiple_Complex
TC1900008141.hg.1	6.91	7.51	-1.52	0.0035	0.0125	CYP2S1	cytochrome P450, family 2, subfamily S, polyp	Multiple_Complex
TC1400008462.hg.1	4.1	3.65	1.37	0.0035	0.0125	C14orf79	chromosome 14 open reading frame 79	Multiple_Complex
TC0700006715.hg.1	6.28	7.05	-1.71	0.0035	0.0126	PHF14	PHD finger protein 14	Multiple_Complex
TC1400008590.hg.1	5.14	5.69	-1.46	0.0035	0.0126	OSGEP	O-sialoglycoprotein endopeptidase	Multiple_Complex
TC1900011981.hg.1	5.55	6.34	-1.72	0.0035	0.0126	ERCC2	excision repair cross-complementation group	Multiple_Complex
TC0500013377.hg.1	7.01	7.53	-1.44	0.0035	0.0126	HARS	histidyl-tRNA synthetase	Multiple_Complex
TC0200016369.hg.1	4.81	5.28	-1.39	0.0035	0.0126	DTYMK	deoxythymidylate kinase	Multiple_Complex
TC0700010924.hg.1	4.61	4.15	1.37	0.0035	0.0126	NPC1L1	NPC1-like 1	Multiple_Complex
TC0100018219.hg.1	11.22	11.75	-1.44	0.0035	0.0126	PPCS; CCD(	phosphopantothenoylcysteine synthetase; coi	Multiple_Complex
TC1500007814.hg.1	5.02	5.86	-1.79	0.0035	0.0127	NEO1	neogenin 1	Multiple_Complex
TC0100011200.hg.1	7.17	6.48	1.61	0.0035	0.0127	TIMM17A	translocase of inner mitochondrial membrane	Multiple_Complex
TC1300007148.hg.1	6.69	7.75	-2.09	0.0035	0.0127	CYSLTR2	cysteinyl leukotriene receptor 2	Coding
TC1400006508.hg.1	4.17	3.53	1.56	0.0035	0.0127	OR11H6	olfactory receptor, family 11, subfamily H, me	Coding
TC0500009319.hg.1	9.35	9.87	-1.44	0.0035	0.0127	CCNG1	cyclin G1	Multiple_Complex
TC1400010776.hg.1	4.87	5.54	-1.59	0.0035	0.0127	CATSPERB	catsper channel auxiliary subunit beta	Multiple_Complex
TC0300012482.hg.1	7.2	6.39	1.75	0.0035	0.0127	RAB6B	RAB6B, member RAS oncogene family	Multiple_Complex
TC0300013707.hg.1	7.46	7.8	-1.27	0.0035	0.0127	WDR53	WD repeat domain 53	Coding
TC0700010945.hg.1	5.59	5.03	1.48	0.0035	0.0128	NACAD	NAC alpha domain containing	Multiple_Complex
TC0500012182.hg.1	9.69	9.05	1.57	0.0036	0.0128	HSPA9	heat shock 70kDa protein 9 (mortalin)	Multiple_Complex
TC0300010540.hg.1	9.55	10.25	-1.62	0.0036	0.0128	TOP2B; MII	topoisomerase (DNA) II beta; microRNA 4442	Multiple_Complex
TC0700007121.hg.1	7.32	8.07	-1.68	0.0036	0.0128	AVL9	AVL9 homolog (S. cerevisiase)	Multiple_Complex
TC1200008706.hg.1	10.87	11.53	-1.58	0.0036	0.0128	KIAA1033	KIAA1033	Multiple_Complex
TC0100007532.hg.1	9.16	8.69	1.39	0.0036	0.0128	STX12	syntaxin 12	Multiple_Complex
TC1100007684.hg.1	6.89	9.17	-4.85	0.0036	0.0128	SERPING1	serpin peptidase inhibitor, clade G (C1 inhibito	Multiple_Complex
TC1700006522.hg.1	5	5.45	-1.37	0.0036	0.0128	WDR81	WD repeat domain 81	Multiple_Complex
TC0100014848.hg.1	7.94	8.34	-1.32	0.0036	0.0129	CCBL2; RBM	cysteine conjugate-beta lyase 2; RNA binding	Multiple_Complex
TC0200006912.hg.1	4.2	3.73	1.38	0.0036	0.0129	TDRD15	tudor domain containing 15	Multiple_Complex
TC0100017445.hg.1	10.66	9.61	2.06	0.0036	0.0129	TLR5	toll-like receptor 5	Multiple_Complex
TC0200016605.hg.1	7.28	6.92	1.29	0.0036	0.0129	UBE2F-SCL'	UBE2F-SCLY readthrough (NMD candidate)	Multiple_Complex
TC1400007673.hg.1	5.37	5	1.29	0.0036	0.0129	VRTN	vertebrae development associated	Multiple_Complex
TC0Y00007331.hg.1	4.16	3.77	1.31	0.0036	0.0129	CSPG4P1Y	chondroitin sulfate proteoglycan 4 pseudogen	Multiple_Complex
TC1200010705.hg.1	6.43	6.85	-1.33	0.0036	0.0129	GALNT6	polypeptide N-acetylgalactosaminyltransferas	Multiple_Complex
TC1000008081.hg.1	6.83	7.63	-1.74	0.0036	0.013	KAT6B	K(lysine) acetyltransferase 6B	Multiple_Complex
TC0800009636.hg.1	4.29	3.55	1.67	0.0036	0.013	USP17L7	ubiquitin specific peptidase 17-like family mer	Coding
TC1400006723.hg.1	9.09	8.26	1.78	0.0036	0.013	REC8	REC8 meiotic recombination protein	Multiple_Complex
TC0300007408.hg.1	6.41	6.09	1.25	0.0036	0.013	GNAT1	guanine nucleotide binding protein (G protein	Multiple_Complex
TC1200008508.hg.1	6.58	7.06	-1.39	0.0036	0.0131	VEZT	vezatin, adherens junctions transmembrane p	Multiple_Complex
TC0100017974.hg.1	8.63	9.16	-1.44	0.0036	0.0131	CEP170	centrosomal protein 170kDa	Multiple_Complex
TSUnmapped00000401.h	10.22	10.97	-1.68	0.0037	0.0131	INPP5D	inositol polyphosphate-5-phosphatase D	NonCoding
TC1700009189.hg.1	7.93	7.36	1.48	0.0037	0.0131	HGS	hepatocyte growth factor-regulated tyrosine k	Multiple_Complex
TC1600009580.hg.1	7.97	8.43	-1.38	0.0037	0.0131	XYLT1	xylosyltransferase I	Multiple_Complex
TC1100010778.hg.1	4	3.52	1.39	0.0037	0.0131	OR4C5	olfactory receptor, family 4, subfamily C, mem	Coding
TC1700007677.hg.1	4.03	4.49	-1.38	0.0037	0.0131	DUSP14	dual specificity phosphatase 14	Coding
TC0200014845.hg.1	5.22	4.48	1.66	0.0037	0.0131	SPC25	SPC25, NDC80 kinetochore complex compone	Multiple_Complex
TC0900009776.hg.1	7	7.4	-1.31	0.0037	0.0132	MOB3B	MOB kinase activator 3B	Multiple_Complex
TC1900008254.hg.1	4.86	5.4	-1.46	0.0037	0.0132	ZNF234	zinc finger protein 234	Multiple_Complex
TC0500008121.hg.1	6.13	6.48	-1.28	0.0037	0.0132	ARSK	arylsulfatase family, member K	Multiple_Complex
TC0100013902.hg.1	5.51	4.78	1.65	0.0037	0.0133	SVBP	small vasohibin binding protein	Multiple_Complex
TC1900009999.hg.1	6.81	7.38	-1.48	0.0037	0.0133	IL12RB1	interleukin 12 receptor, beta 1	Multiple_Complex
TC1300008780.hg.1	5.25	4.87	1.3	0.0037	0.0133	SMIM2	small integral membrane protein 2	Coding
TC0X00008495.hg.1	7.88	8.56	-1.6	0.0037	0.0133	FAM127A	family with sequence similarity 127, member ,	Multiple_Complex
TC1100010038.hg.1	4.66	3.92	1.66	0.0037	0.0133	ST5	suppression of tumorigenicity 5	Multiple_Complex
TC1900007436.hg.1	9.04	8.29	1.68	0.0037	0.0133	GATAD2A;	GATA zinc finger domain containing 2A; micro	Multiple_Complex
TC1200011491.hg.1	4.49	3.92	1.49	0.0037	0.0134	EPYC	epiphycan	Multiple_Complex
TC1400010765.hg.1	7.95	7.14	1.75	0.0037	0.0134	RDH11	retinol dehydrogenase 11 (all-trans/9-cis/11-c	Multiple_Complex
TC0X00007392.hg.1	9.33	10.06	-1.66	0.0038	0.0134	UBQLN2	ubiquilin 2	Coding
TC0600008670.hg.1	4.43	3.96	1.39	0.0038	0.0134	PRSS35	protease, serine 35	Coding
TC1900007045.hg.1	4.76	5.3	-1.46	0.0038	0.0134	ZNF441	zinc finger protein 441	Multiple_Complex
TC0100011953.hg.1	6.95	7.71	-1.7	0.0038	0.0134	ARV1	ARV1 homolog, fatty acid homeostasis modul	Multiple_Complex
TC1900010164.hg.1	8.86	9.36	-1.42	0.0038	0.0134	ZNF208	zinc finger protein 208	Multiple_Complex
TC1000010831.hg.1	10.64	11.13	-1.41	0.0038	0.0134	HERC4	HECT and RLD domain containing E3 ubiquitin	Multiple_Complex
TC1100008006.hg.1	4.04	3.84	1.15	0.0038	0.0134	SLC22A20	solute carrier family 22, member 20	Multiple_Complex
TC1000008743.hg.1	4.89	5.37	-1.4	0.0038	0.0134	SUFU	Transcript Identified by AceView, Entrez Gene	Unassigned
TC0900006762.hg.1	6.11	5.49	1.54	0.0038	0.0135	ACER2	alkaline ceramidase 2	Multiple_Complex
TC1200008916.hg.1	9.6	9.15	1.37	0.0038	0.0136	PTPN11	protein tyrosine phosphatase, non-receptor ty	Multiple_Complex
TC1200009209.hg.1	6.26	6.79	-1.44	0.0038	0.0136	KNTC1	kinetochore associated 1	Multiple_Complex

TC1100006695.hg.1	3.7	3	1.62	0.0038	0.0136	OR51I2	olfactory receptor, family 51, subfamily I, mer	Coding
TC0500007738.hg.1	4.32	3.85	1.38	0.0038	0.0136	MAP1B	microtubule associated protein 1B	Multiple_Complex
TC0500012909.hg.1	5.53	5.27	1.2	0.0038	0.0136	DRD1	dopamine receptor D1	Coding
TC1200011894.hg.1	6.99	6.23	1.69	0.0038	0.0136	C12orf76	chromosome 12 open reading frame 76	Multiple_Complex
TC0500010600.hg.1	5.49	6.38	-1.85	0.0038	0.0137	OXCT1	Transcript Identified by AceView, Entrez Gene	Unassigned
TC2200009347.hg.1	4.53	4.93	-1.32	0.0038	0.0137	ANKRD54;	ankyrin repeat domain 54; microRNA 658	Multiple_Complex
TC0800012168.hg.1	6.48	6.05	1.35	0.0038	0.0137	NRBP2; MI	nuclear receptor binding protein 2; microRNA	Multiple_Complex
TC1200010448.hg.1	6.64	7.19	-1.46	0.0038	0.0137	GXYLT1	glucoside xylosyltransferase 1	Multiple_Complex
TC1600009184.hg.1	5.07	5.81	-1.67	0.0038	0.0137	ZNF200	Transcript Identified by AceView, Entrez Gene	Unassigned
TC1500008269.hg.1	4.98	4.48	1.41	0.0038	0.0137	KIF7	Memczak2013 ANTISENSE, CDS, coding, INTEF	NonCoding
TC2000007227.hg.1	7.89	7.54	1.28	0.0038	0.0137	ROMO1	reactive oxygen species modulator 1	Multiple_Complex
TC1500008971.hg.1	4.89	5.14	-1.19	0.0038	0.0137	AVEN	apoptosis, caspase activation inhibitor	Multiple_Complex
TC1800008128.hg.1	9.68	8.99	1.62	0.0038	0.0137	PTPN2	protein tyrosine phosphatase, non-receptor ty	Multiple_Complex
TSUnmapped00000292.h	4.54	4	1.46	0.0039	0.0137	TGM4	transglutaminase 4	Coding
TC2000007503.hg.1	5.49	5.04	1.36	0.0039	0.0137	UBE2C	ubiquitin-conjugating enzyme E2C	Multiple_Complex
TSUnmapped00000493.h	6.95	6.38	1.48	0.0039	0.0137	TRAPPC4	trafficking protein particle complex 4	Coding
TC0600007887.hg.1	13.12	12.28	1.79	0.0039	0.0137	ZFAND3	zinc finger, AN1-type domain 3	Multiple_Complex
TC0700013602.hg.1	11.46	12.04	-1.5	0.0039	0.0137	POLR2J3	polymerase (RNA) II (DNA directed) polypeptic	Multiple_Complex
TC1600011300.hg.1	3.76	4.1	-1.26	0.0039	0.0138	CENPBD1	CENPB DNA-binding domain containing 1	Multiple_Complex
TC1100009445.hg.1	5.96	6.48	-1.43	0.0039	0.0138	FAM118B	family with sequence similarity 118, member	Multiple_Complex
TC1800007355.hg.1	4.12	4.82	-1.63	0.0039	0.0138	C18orf54	chromosome 18 open reading frame 54	Multiple_Complex
TC0100015627.hg.1	6.5	7	-1.41	0.0039	0.0138	PRKAB2	protein kinase, AMP-activated, beta 2 non-cat	Multiple_Complex
TC0300008711.hg.1	6.03	5.45	1.49	0.0039	0.0138	KBTBD12	kelch repeat and BTB (POZ) domain containing	Multiple_Complex
TC2200009346.hg.1	5.37	5.71	-1.27	0.0039	0.0139	C1QTNF6	C1q and tumor necrosis factor related protein	NonCoding
TC1400009668.hg.1	9.74	8.95	1.72	0.0039	0.0139	PNMA1	paraneoplastic Ma antigen 1	Coding
TC1900007869.hg.1	7.76	8.54	-1.72	0.0039	0.0139	RBM42	RNA binding motif protein 42	Multiple_Complex
TC0600014154.hg.1	7.26	7.79	-1.45	0.0039	0.0139	SLC35A1	solute carrier family 35 (CMP-sialic acid transp	Multiple_Complex
TC1200009748.hg.1	7.73	8.45	-1.65	0.0039	0.0139	ZNF384	zinc finger protein 384	Multiple_Complex
TC0600014069.hg.1	8.47	8.9	-1.34	0.0039	0.0139	TMEM14B	transmembrane protein 14B	Multiple_Complex
TC0900011454.hg.1	5.8	6.6	-1.74	0.0039	0.014	ZBTB26	zinc finger and BTB domain containing 26	Coding
TC1500010842.hg.1	6.19	7.02	-1.77	0.0039	0.014	GOLGA8R	golgin A8 family, member R	Multiple_Complex
TC0X00008689.hg.1	5.2	4.7	1.41	0.0039	0.014	MAGEA11	MAGE family member A11	Multiple_Complex
TC1200011862.hg.1	7.4	8	-1.51	0.0039	0.014	ALKBH2	alkB homolog 2, alpha-ketoglutarate-depende	Multiple_Complex
TC1200009489.hg.1	4.6	5.43	-1.77	0.0039	0.014	FBRSL1	fibrosin-like 1	Multiple_Complex
TC0400008165.hg.1	4.69	4.25	1.35	0.0039	0.014	ATOH1	atonal bHLH transcription factor 1	Coding
TC0800006864.hg.1	8.46	9.33	-1.83	0.004	0.014	ZDHHC2	zinc finger, DHHC-type containing 2	Multiple_Complex
TC0400010606.hg.1	7.7	7.14	1.48	0.004	0.014	COMMD8	COMM domain containing 8	Multiple_Complex
TC1500006972.hg.1	6.8	7.66	-1.82	0.004	0.014	SPINT1	serine peptidase inhibitor, Kunitz type 1	Multiple_Complex
TC0300013918.hg.1	5.68	5.26	1.34	0.004	0.0141	SLC51A	solute carrier family 51, alpha subunit	Multiple_Complex
TC0900011209.hg.1	5.53	4.84	1.62	0.004	0.0141	C9orf84	chromosome 9 open reading frame 84	Multiple_Complex
TC1700010592.hg.1	9.06	9.71	-1.57	0.004	0.0141	ORMDL3	ORMDL sphingolipid biosynthesis regulator 3	Multiple_Complex
TC0300008369.hg.1	10.61	9.86	1.69	0.004	0.0141	ATP6V1A	ATPase, H+ transporting, lysosomal 70kDa, V1	Multiple_Complex
TC0700008003.hg.1	7.05	6.5	1.47	0.004	0.0141	CLIP2	CAP-GLY domain containing linker protein 2	Multiple_Complex
TC0200010834.hg.1	8.11	8.7	-1.5	0.004	0.0141	FAM134A	family with sequence similarity 134, member	Multiple_Complex
TC0500007772.hg.1	10.81	9.28	2.89	0.004	0.0141	BTF3	basic transcription factor 3	Multiple_Complex
TC1600010262.hg.1	8.95	9.46	-1.42	0.004	0.0142	BRD7	bromodomain containing 7	Multiple_Complex
TC0600009143.hg.1	5.55	6.1	-1.46	0.004	0.0142	SLC16A10	solute carrier family 16 (aromatic amino acid t	Multiple_Complex
TC1500010777.hg.1	4.99	4.3	1.61	0.004	0.0142	IL16	interleukin 16	NonCoding
TC0100008156.hg.1	6.57	7.29	-1.65	0.004	0.0142	NSUN4	NOP2/Sun domain family, member 4	Multiple_Complex
TC0700008095.hg.1	7.16	7.55	-1.31	0.004	0.0142	DTX2	deltex 2, E3 ubiquitin ligase	Multiple_Complex
TC0300008962.hg.1	4.45	4.05	1.32	0.004	0.0143	FOXL2NB	FOXL2 neighbor	Multiple_Complex
TC1100010712.hg.1	10	10.97	-1.96	0.004	0.0143	ARHGAP1	Rho GTPase activating protein 1	Multiple_Complex
TC1100012052.hg.1	8.81	7.55	2.4	0.004	0.0143	CCDC82	coiled-coil domain containing 82	Multiple_Complex
TSUnmapped00000569.h	8.49	7.94	1.46	0.004	0.0143	ZDHHC3	zinc finger, DHHC-type containing 3	Coding
TC1200007714.hg.1	3.49	3.16	1.26	0.004	0.0143	AMHR2	anti-Mullerian hormone receptor, type II	Multiple_Complex
TC0200015714.hg.1	13.33	14.81	-2.78	0.0041	0.0143	CXCR1	chemokine (C-X-C motif) receptor 1	Multiple_Complex
TC0X00011288.hg.1	4.01	3.31	1.62	0.0041	0.0144	GAGE12I; G	G antigen 12I; G antigen 12F	Coding
TC0900010878.hg.1	5.16	5.71	-1.46	0.0041	0.0144	FANCC	Fanconi anemia complementation group C	Multiple_Complex
TC1600009642.hg.1	10.58	9.6	1.97	0.0041	0.0144	GDE1	glycerophosphodiester phosphodiesterase 1	Multiple_Complex
TC1300009810.hg.1	10.3	9.7	1.51	0.0041	0.0144	ANKRD10	ankyrin repeat domain 10	Multiple_Complex
TCUn_GL000219v100006	6.51	7.02	-1.43	0.0041	0.0144	LOC28378	FSHD region gene 1 pseudogene	Multiple_Complex
TC0100015608.hg.1	6.46	6.89	-1.34	0.0041	0.0144	LOC105371	peptidyl-prolyl cis-trans isomerase A-like 4G; F	Coding
TC0800011472.hg.1	5.56	6.48	-1.89	0.0041	0.0144	NUDCD1	NudC domain containing 1	Multiple_Complex
TC0200007473.hg.1	9.35	9.62	-1.21	0.0041	0.0144	CRIPT	cysteine-rich PDZ-binding protein	Multiple_Complex
TC1900009594.hg.1	5.46	4.89	1.49	0.0041	0.0145	ZNF812P	zinc finger protein 812, pseudogene	Multiple_Complex
TC1100009917.hg.1	19.36	19.66	-1.23	0.0041	0.0145	HBB	hemoglobin, beta	Multiple_Complex
TC1400010579.hg.1	5.5	5.89	-1.31	0.0041	0.0145	DHRS4	dehydrogenase/reductase (SDR family) memb	Coding
TC0700007399.hg.1	14.52	13.13	2.63	0.0041	0.0145	PPIA	peptidylprolyl isomerase A (cyclophilin A)	Multiple_Complex
TC1700012186.hg.1	7.65	8.36	-1.63	0.0041	0.0145	TNFSF13	tumor necrosis factor (ligand) superfamily, me	Multiple_Complex
TC0600013350.hg.1	7.59	8.25	-1.58	0.0041	0.0145	REPS1	RALBP1 associated Eps domain containing 1	Multiple_Complex

TC1900011464.hg.1	5.43	6.2	-1.7	0.0041	0.0145	TMEM150B	transmembrane protein 150B	Multiple_Complex
TC2200008000.hg.1	5.98	5.57	1.33	0.0041	0.0145	TXNRD2	Transcript Identified by AceView, Entrez Gene	Coding
TC1700012226.hg.1	6.47	6.89	-1.34	0.0041	0.0145	ADAP2	ArfGAP with dual PH domains 2	Multiple_Complex
TC1500010855.hg.1	5.07	5.5	-1.35	0.0041	0.0146	EPB42	erythrocyte membrane protein band 4.2	NonCoding
TC0500013241.hg.1	3.85	3.4	1.36	0.0041	0.0146	PCDHB5	protocadherin beta 5	Coding
TC0200014735.hg.1	9.91	9.01	1.87	0.0041	0.0146	RBMS1; MI	RNA binding motif, single stranded interacting	Multiple_Complex
TC0400012640.hg.1	3.97	3.49	1.4	0.0041	0.0146	CCDC110	coiled-coil domain containing 110	Coding
TC1800008231.hg.1	9	8.37	1.56	0.0042	0.0146	ESCO1	establishment of sister chromatid cohesion N-	Multiple_Complex
TC1400010730.hg.1	4.87	4.23	1.56	0.0042	0.0147	EGLN3	egl-9 family hypoxia-inducible factor 3	Multiple_Complex
TC0900011841.hg.1	6.47	5.81	1.58	0.0042	0.0147	SURF1	surfeit 1	Multiple_Complex
TC1200007147.hg.1	4.74	3.93	1.76	0.0042	0.0147	ARNTL2	aryl hydrocarbon receptor nuclear translocato	Multiple_Complex
TC1000010562.hg.1	5.21	4.96	1.19	0.0042	0.0147	SYT15; ABC	Homo sapiens synaptotagmin XV (SYT15), trar	Coding
TC1100011311.hg.1	4.67	5.06	-1.31	0.0042	0.0147	PC	Memczak2013 ALT_ACCEPTOR, ALT_DONOR,	NonCoding
TC1600009978.hg.1	4.72	5.26	-1.46	0.0042	0.0147	ZNF785	zinc finger protein 785	Coding
TC0300006956.hg.1	6	6.45	-1.37	0.0042	0.0148	GPD1L	glycerol-3-phosphate dehydrogenase 1-like	Multiple_Complex
TC2200009335.hg.1	9.91	9.19	1.65	0.0042	0.0148	THOC5	THO complex 5	Multiple_Complex
TC1900006999.hg.1	11.67	11.12	1.46	0.0042	0.0148	DNM2	Transcript Identified by AceView, Entrez Gene	Unassigned
TC0300011247.hg.1	4.68	4.4	1.22	0.0042	0.0148	WNT5A	wingless-type MMTV integration site family, n	Multiple_Complex
TC0600007616.hg.1	8.38	9.06	-1.6	0.0042	0.0148	HSPA1B; HS	heat shock 70kDa protein 1B; heat shock 70kD	Coding
TC1400006697.hg.1	4.74	4.27	1.38	0.0042	0.0148	DHRS2	dehydrogenase/reductase (SDR family) memb	Multiple_Complex
TSUnmapped00000107.h	6.43	7.23	-1.74	0.0042	0.0149	DUSP16	dual specificity phosphatase 16	Coding
TC0300014034.hg.1	6.74	6.11	1.55	0.0042	0.0149	COX17	COX17 cytochrome c oxidase copper chaperon	Multiple_Complex
TC1100009859.hg.1	9.24	8.85	1.32	0.0043	0.0149	NUP98	Transcript Identified by AceView, Entrez Gene	Unassigned
TC1500010728.hg.1	7.28	6.58	1.62	0.0043	0.015	SERF2	small EDRK-rich factor 2	Multiple_Complex
TC0700012847.hg.1	3.87	3.45	1.34	0.0043	0.015	PRSS37	protease, serine, 37	Coding
TC1600010006.hg.1	5.97	5.62	1.28	0.0043	0.015	PRSS8	protease, serine, 8	Multiple_Complex
TC1100009618.hg.1	4.89	5.63	-1.67	0.0043	0.015	GLB1L2	galactosidase beta 1 like 2	Multiple_Complex
TC1000009920.hg.1	8.87	8.2	1.59	0.0043	0.015	VIM-AS1	VIM antisense RNA 1	NonCoding
TC1800008804.hg.1	4.83	4.23	1.51	0.0043	0.015	ALPK2	alpha kinase 2	Multiple_Complex
TC0900011654.hg.1	7.21	8.03	-1.77	0.0043	0.015	ZDHHC12	zinc finger, DHHC-type containing 12	Multiple_Complex
TC1000008770.hg.1	6.67	7.11	-1.35	0.0043	0.015	PDCD11	programmed cell death 11	Multiple_Complex
TC0400012916.hg.1	6.85	7.24	-1.32	0.0043	0.015	ARAP2	ArfGAP with RhoGAP domain, ankyrin repeat a	NonCoding
TC0700006565.hg.1	5.36	4.72	1.55	0.0043	0.0151	IQCE	IQ motif containing E	Multiple_Complex
TC0100007469.hg.1	10.73	9.92	1.74	0.0043	0.0151	RPS6KA1	ribosomal protein S6 kinase, 90kDa, polypepti	Multiple_Complex
TC0800012395.hg.1	5.08	5.53	-1.37	0.0043	0.0151	ZNF395	zinc finger protein 395	Multiple_Complex
TC0Y00006861.hg.1	5.03	5.61	-1.5	0.0043	0.0151	GTPBP6	Homo sapiens GTP binding protein 6 (putative	Multiple_Complex
TC0600014186.hg.1	6.64	7.44	-1.74	0.0043	0.0151	PEX3	peroxisomal biogenesis factor 3	Multiple_Complex
TC0100014576.hg.1	4.43	3.96	1.39	0.0043	0.0151	PTGER3	prostaglandin E receptor 3 (subtype EP3)	Multiple_Complex
TC0300006948.hg.1	4.97	5.74	-1.71	0.0043	0.0152	ZNF860	zinc finger protein 860	Multiple_Complex
TC1500007240.hg.1	12.01	10.94	2.1	0.0043	0.0152	TMOD3	tropomodulin 3 (ubiquitous)	Multiple_Complex
TC2100006960.hg.1	11.38	10.84	1.45	0.0043	0.0152	IFNAR1	interferon (alpha, beta and omega) receptor 1	Multiple_Complex
TC0500011602.hg.1	5.81	6.72	-1.88	0.0043	0.0152	GIN1	gypsy retrotransposon integrase 1	Multiple_Complex
TC0700010538.hg.1	13.17	12.4	1.71	0.0043	0.0152	HNRNPA2B	heterogeneous nuclear ribonucleoprotein A2/	Multiple_Complex
TC1900011449.hg.1	4.71	4.18	1.44	0.0044	0.0152	TNNT1	troponin T type 1 (skeletal, slow)	Multiple_Complex
TC1500010688.hg.1	4.57	4.01	1.47	0.0044	0.0152	OR4N4	olfactory receptor, family 4, subfamily N, men	Coding
TC0Y00007341.hg.1	4.44	3.65	1.72	0.0044	0.0152	RBMY1E; R	RNA binding motif protein, Y-linked, family 1,	Coding
TC1900011859.hg.1	4.18	3.81	1.29	0.0044	0.0153	ZNF846	zinc finger protein 846	NonCoding
TC1100006713.hg.1	4.41	3.64	1.7	0.0044	0.0153	OR56A3	olfactory receptor, family 56, subfamily A, me	Coding
TC0100015866.hg.1	12.33	11.14	2.29	0.0044	0.0153	S100A4	S100 calcium binding protein A4	Multiple_Complex
TC0200013283.hg.1	6.34	6.77	-1.35	0.0044	0.0154	TMEM150A	transmembrane protein 150A	Multiple_Complex
TC0800010987.hg.1	5.25	4.86	1.31	0.0044	0.0154	C8orf59	chromosome 8 open reading frame 59	Multiple_Complex
TC1900009754.hg.1	6.18	7.07	-1.85	0.0044	0.0154	DNASE2	deoxyribonuclease II, lysosomal	Multiple_Complex
TC0600007472.hg.1	4.01	3.41	1.51	0.0044	0.0154	OR2H2	olfactory receptor, family 2, subfamily H, men	Coding
TC2200009303.hg.1	4.21	3.87	1.27	0.0044	0.0154	MAPK8IP2	mitogen-activated protein kinase 8 interacting	Coding
TC0700010697.hg.1	6.57	7.16	-1.51	0.0044	0.0154	RP9	retinitis pigmentosa 9 (autosomal dominant)	Multiple_Complex
TC0400012990.hg.1	14.1	15.84	-3.34	0.0044	0.0154	DDX60L	DEAD (Asp-Glu-Ala-Asp) box polypeptide 60-li	Multiple_Complex
TC1200011993.hg.1	7.97	8.73	-1.69	0.0044	0.0155	SLC8B1	solute carrier family 8 (sodium/lithium/calciur	Multiple_Complex
TC1900008555.hg.1	6.91	7.5	-1.5	0.0044	0.0155	AP2A1	adaptor-related protein complex 2, alpha 1 su	Multiple_Complex
TC1400010627.hg.1	9.95	10.41	-1.37	0.0044	0.0155	YLPM1	YLP motif containing 1	Multiple_Complex
TC1600006469.hg.1	7.2	7.52	-1.24	0.0044	0.0155	CAPN15; M	calpain 15; microRNA 5587	Multiple_Complex
TC1200008667.hg.1	5.66	6.56	-1.86	0.0044	0.0155	HSP90B1	Transcript Identified by AceView, Entrez Gene	Unassigned
TC0400012977.hg.1	5.35	4.89	1.38	0.0044	0.0155	SH3D19	SH3 domain containing 19	Multiple_Complex
TC0400007789.hg.1	4.49	5.51	-2.03	0.0045	0.0156	RUFY3	RUN and FYVE domain containing 3	Multiple_Complex
TC2100007331.hg.1	5.52	6.3	-1.72	0.0045	0.0156	AGPAT3	1-acylglycerol-3-phosphate O-acyltransferase	Multiple_Complex
TC0700012898.hg.1	5.52	5.89	-1.29	0.0045	0.0156	TCAF1	TRPM8 channel-associated factor 1	Multiple_Complex
TC2000007666.hg.1	9.32	9.84	-1.43	0.0045	0.0156	RNF114	ring finger protein 114	Multiple_Complex
TC1600008899.hg.1	5.78	6.41	-1.54	0.0045	0.0156	ZNF276	zinc finger protein 276	Multiple_Complex
TC0900012216.hg.1	4.62	4.07	1.46	0.0045	0.0156	IFNA17	interferon, alpha 17	Coding
TC1100007808.hg.1	7.25	7.82	-1.48	0.0045	0.0156	SDHAF2	Memczak2013 ALT_ACCEPTOR, ALT_DONOR,	NonCoding
TC0700010565.hg.1	4.43	4.19	1.19	0.0045	0.0157	HOXA11	homeobox A11	Coding

TC0100017223.hg.1	9.72	8.89	1.78	0.0045	0.0157	LPGAT1	lysophosphatidylglycerol acyltransferase 1	Multiple_Complex
TC0200016477.hg.1	7.63	8.06	-1.35	0.0045	0.0157	INO80B-WI	INO80B-WBP1 readthrough (NMD candidate)	Multiple_Complex
TC0200011121.hg.1	11.74	12.17	-1.35	0.0045	0.0157	INPP5D	inositol polyphosphate-5-phosphatase D	Multiple_Complex
TC0200014244.hg.1	4.47	4.18	1.22	0.0045	0.0157	FAR2P1	fatty acyl-CoA reductase 2 pseudogene 1	Multiple_Complex
TC2200008091.hg.1	7.59	8.12	-1.44	0.0045	0.0158	PI4KAP2	phosphatidylinositol 4-kinase, catalytic, alpha	Multiple_Complex
TC0500009610.hg.1	9.37	9.87	-1.42	0.0045	0.0158	NSD1	nuclear receptor binding SET domain protein 1	Multiple_Complex
TC1900006470.hg.1	12.48	13.92	-2.71	0.0045	0.0158	BSG	basigin (Ok blood group)	Multiple_Complex
TC0200014154.hg.1	3.68	4.52	-1.79	0.0045	0.0158	GYPC	Memczak2013 ANTISENSE, coding, INTERNAL,	NonCoding
TC0500013144.hg.1	4.99	5.53	-1.45	0.0045	0.0158	CEP72	centrosomal protein 72kDa	Multiple_Complex
TC0300010989.hg.1	4.4	3.79	1.54	0.0046	0.0158	CSPG5	chondroitin sulfate proteoglycan 5 (neuroglyc	Multiple_Complex
TC1500010234.hg.1	6.18	6.94	-1.7	0.0046	0.0159	EFTUD1	elongation factor Tu GTP binding domain cont	Multiple_Complex
TC0700010604.hg.1	9.27	10.29	-2.04	0.0046	0.0159	CPVL	carboxypeptidase, vitellogenic-like	Multiple_Complex
TC0600008912.hg.1	4.73	4.36	1.29	0.0046	0.0159	PRDM13	PR domain containing 13	Coding
TC1300008179.hg.1	8.92	9.57	-1.57	0.0046	0.0159	UPF3A	UPF3 regulator of nonsense transcripts homol	Multiple_Complex
TC1500008470.hg.1	7.37	6.54	1.78	0.0046	0.0159	ARRDC4	arrestin domain containing 4	Coding
TC2200006450.hg.1	3.98	3.37	1.53	0.0046	0.0159	OR11H1	olfactory receptor, family 11, subfamily H, me	Coding
TC0900008490.hg.1	4.84	4.41	1.35	0.0046	0.016	C9orf43	chromosome 9 open reading frame 43	Multiple_Complex
TC1000012474.hg.1	5.36	5.89	-1.44	0.0046	0.016	NUTM2D	NUT family member 2D	Multiple_Complex
TC0400009897.hg.1	5.02	5.69	-1.59	0.0046	0.016	JAKMIP1	janus kinase and microtubule interacting prot	Multiple_Complex
TC0300006561.hg.1	7	7.41	-1.32	0.0046	0.016	JAGN1	jagunal homolog 1	Multiple_Complex
TC0700010181.hg.1	5.43	4.8	1.55	0.0046	0.016	RSPH10B2;	radial spoke head 10 homolog B2 (Chlamydon	Multiple_Complex
TC1200012575.hg.1	5.89	6.51	-1.53	0.0046	0.016	DYRK4	dual specificity tyrosine-(Y)-phosphorylation r	Multiple_Complex
TC1200012700.hg.1	9.97	9.28	1.62	0.0046	0.016	C12orf75	chromosome 12 open reading frame 75	Multiple_Complex
TC0100007575.hg.1	8.66	9.21	-1.46	0.0046	0.016	EPB41	Memczak2013 ALT_ACCEPTOR, ALT_DONOR,	NonCoding
TC1100006629.hg.1	7.37	6.63	1.67	0.0046	0.016	ART1	ADP-ribosyltransferase 1	Multiple_Complex
TC1700011703.hg.1	6.86	6.37	1.41	0.0046	0.0161	CD300LD	CD300 molecule-like family member d	Coding
TC1200011608.hg.1	11.97	12.43	-1.37	0.0046	0.0161	CDK17	cyclin-dependent kinase 17	Multiple_Complex
TC1700006999.hg.1	4.65	4.49	1.12	0.0046	0.0161	ADORA2B	adenosine A2b receptor	Multiple_Complex
TC0100011733.hg.1	10.05	10.4	-1.27	0.0046	0.0161	FBXO28	F-box protein 28	Multiple_Complex
TSUnmapped00000050.h	7.47	8.19	-1.65	0.0046	0.0161	MLXIP	MLX interacting protein	Coding
TC0400009111.hg.1	4.52	3.88	1.56	0.0047	0.0161	GRIA2	Transcript Identified by AceView, Entrez Gene	Unassigned
TC1700006721.hg.1	6.85	7.15	-1.24	0.0047	0.0162	SLC16A13	solute carrier family 16, member 13	Multiple_Complex
TC1100011629.hg.1	5.05	5.79	-1.67	0.0047	0.0162	XRRA1	X-ray radiation resistance associated 1	Multiple_Complex
TC1700007642.hg.1	4.08	4.46	-1.31	0.0047	0.0162	MRM1	mitochondrial rRNA methyltransferase 1	Multiple_Complex
TC1600008737.hg.1	4.03	3.5	1.44	0.0047	0.0163	FLJ30679; F	uncharacterized protein FLJ30679; uncharacte	NonCoding
TC1900012033.hg.1	4.35	4.85	-1.41	0.0047	0.0163	ZNF347	zinc finger protein 347	Multiple_Complex
TC0200012499.hg.1	7.75	7.01	1.67	0.0047	0.0163	MCFD2	multiple coagulation factor deficiency 2	Multiple_Complex
TC0500007694.hg.1	10.31	11.12	-1.75	0.0047	0.0163	GTF2H2; G1	general transcription factor IIH subunit 2; gen	Multiple_Complex
TC0100007477.hg.1	9.35	10.45	-2.14	0.0047	0.0163	ARID1A	AT rich interactive domain 1A (SWI-like)	Multiple_Complex
TC0900011708.hg.1	6.28	5.91	1.29	0.0047	0.0163	PTGES	prostaglandin E synthase	Multiple_Complex
TC1900006864.hg.1	5.41	6.05	-1.56	0.0047	0.0164	MAP2K7	mitogen-activated protein kinase kinase 7	Multiple_Complex
TC0700007552.hg.1	5.52	5.17	1.27	0.0047	0.0164	POM121L1	POM121 transmembrane nucleoporin-like 12	Coding
TC0700009680.hg.1	5.8	8.69	-7.43	0.0047	0.0164	TMEM176A	transmembrane protein 176A	Multiple_Complex
TC0600011448.hg.1	5.27	4.89	1.29	0.0047	0.0164	LY6G6C	lymphocyte antigen 6 complex, locus G6C	Coding
TC0300012897.hg.1	11.18	11.5	-1.25	0.0048	0.0165	SSR3	signal sequence receptor, gamma (translocon-	Multiple_Complex
TC0300013881.hg.1	9.72	8.86	1.81	0.0048	0.0165	U2SURP	U2 snRNP-associated SURP domain containing	Multiple_Complex
TC1200007421.hg.1	4.96	6.02	-2.08	0.0048	0.0166	ANO6	Transcript Identified by AceView, Entrez Gene	Unassigned
TC0900011793.hg.1	7.3	7.65	-1.27	0.0048	0.0166	MED27	mediator complex subunit 27	Multiple_Complex
TC1700009327.hg.1	4.82	5.4	-1.5	0.0048	0.0166	GEMIN4	gem nuclear organelle associated protein 4	Multiple_Complex
TC0900008679.hg.1	4.5	4.1	1.32	0.0048	0.0166	OR1K1	olfactory receptor, family 1, subfamily K, mem	Coding
TC0900012168.hg.1	6.83	7.52	-1.62	0.0048	0.0166	MRRF	mitochondrial ribosome recycling factor	Multiple_Complex
TC1900011398.hg.1	6.13	5.68	1.37	0.0048	0.0167	TMC4	transmembrane channel like 4	Multiple_Complex
TC1100009973.hg.1	6.82	7.03	-1.16	0.0048	0.0167	ARFIP2	ADP-ribosylation factor interacting protein 2	Multiple_Complex
TC2000009461.hg.1	5.41	4.7	1.64	0.0048	0.0167	ATP9A	ATPase, class II, type 9A	Multiple_Complex
TC0400011464.hg.1	10.26	9.68	1.5	0.0048	0.0167	MANBA	mannosidase, beta A, lysosomal	Multiple_Complex
TC1900009134.hg.1	13.27	12.35	1.89	0.0048	0.0167	SBNO2	strawberry notch homolog 2 (Drosophila)	Multiple_Complex
TC0200014802.hg.1	3.98	4.46	-1.39	0.0048	0.0167	SCN3A	sodium channel, voltage gated, type III alpha s	Multiple_Complex
TC0600008735.hg.1	6.76	7.57	-1.75	0.0048	0.0167	ORC3	origin recognition complex subunit 3	Multiple_Complex
TC1900006989.hg.1	8.74	8.15	1.5	0.0049	0.0168	ATG4D	autophagy related 4D, cysteine peptidase	Multiple_Complex
TC0Y00007326.hg.1	4.62	4.2	1.34	0.0049	0.0168	TSPY1	testis specific protein, Y-linked 1	Coding
TC0500011520.hg.1	4.33	5.32	-1.99	0.0049	0.0168	ERAP1	Transcript Identified by AceView, Entrez Gene	Unassigned
TC1700012262.hg.1	9.74	10.38	-1.55	0.0049	0.0168	PSME3	proteasome activator subunit 3	Multiple_Complex
TC1200012755.hg.1	5.56	6.47	-1.89	0.0049	0.0168	PRR4	proline rich 4 (lacrimal)	Multiple_Complex
TC1700011749.hg.1	13.59	12.95	1.56	0.0049	0.0168	GRB2	growth factor receptor bound protein 2	Multiple_Complex
TC1400007688.hg.1	7.52	6.86	1.58	0.0049	0.0168	FCF1	FCF1 rRNA-processing protein	Multiple_Complex
TC2100007321.hg.1	10.03	9.4	1.55	0.0049	0.0168	PDXK	pyridoxal (pyridoxine, vitamin B6) kinase	Multiple_Complex
TC0X00010840.hg.1	3.75	3.47	1.22	0.0049	0.0168	HS6ST2	heparan sulfate 6-O-sulfotransferase 2	Coding
TSUnmapped00000045.h	6.08	6.55	-1.39	0.0049	0.0168	CCDC84	coiled-coil domain containing 84	Coding
TC0100013226.hg.1	9.54	10.24	-1.63	0.0049	0.0169	USP48	ubiquitin specific peptidase 48	Multiple_Complex
TC0600012032.hg.1	4.73	4.24	1.4	0.0049	0.0169	PKHD1	polycystic kidney and hepatic disease 1 (autos	Multiple_Complex

TC1900008384.hg.1	12.51	11.83	1.61	0.0049	0.0169	CALM3	calmodulin 3 (phosphorylase kinase, delta)	Multiple_Complex
TC1100011914.hg.1	6.18	5.78	1.33	0.0049	0.0169	TRIM64B	tripartite motif containing 64B	Multiple_Complex
TC0600009871.hg.1	4.69	4.23	1.37	0.0049	0.017	ESR1	estrogen receptor 1	Multiple_Complex
TC2200007493.hg.1	18.66	18.12	1.46	0.0049	0.017	MEI1	Salzman2013 ALT_ACCEPTOR, ALT_DONOR, c	NonCoding
TC1900008562.hg.1	5.8	6.3	-1.41	0.0049	0.017	TBC1D17; I	TBC1 domain family, member 17; microRNA 4	Multiple_Complex
TC1000008045.hg.1	7.58	8.04	-1.37	0.005	0.017	SEC24C	SEC24 homolog C, COPII coat complex compo	Multiple_Complex
TC0600007959.hg.1	4.32	3.6	1.65	0.005	0.0171	DAAM2	dishevelled associated activator of morphoge	Multiple_Complex
TSUnmapped00000161.h	6.09	5.61	1.4	0.005	0.0171	SURF1	surfeit 1	Coding
TC1400010758.hg.1	5.07	4.17	1.86	0.005	0.0171	CCDC175	coiled-coil domain containing 175	Multiple_Complex
TC0300013886.hg.1	3.7	3.43	1.21	0.005	0.0171	ZIC1	Zic family member 1	Coding
TC0100014921.hg.1	4.12	4.75	-1.55	0.005	0.0171	GLMN	glomulin, FKBP associated protein	Multiple_Complex
TC1900007051.hg.1	7.17	7.71	-1.46	0.005	0.0171	ZNF439	zinc finger protein 439	Multiple_Complex
TC0100017548.hg.1	4.04	3.69	1.27	0.005	0.0171	CDC42BPA	CDC42 binding protein kinase alpha (DMPK-lik	Multiple_Complex
TC0X00007203.hg.1	6.72	6.32	1.32	0.005	0.0171	GLOD5	glyoxalase domain containing 5	Multiple_Complex
TC2000007695.hg.1	12.04	11.43	1.54	0.005	0.0171	PTPN1	protein tyrosine phosphatase, non-receptor t	Multiple_Complex
TC2000008940.hg.1	5.99	5.39	1.51	0.005	0.0172	PIGU	Salzman2013 ALT_ACCEPTOR, ALT_DONOR, c	NonCoding
TC1100013191.hg.1	7.78	8.54	-1.69	0.005	0.0172	POLD4	polymerase (DNA-directed), delta 4, accessor	Multiple_Complex
TC1200012741.hg.1	4.57	4.15	1.33	0.005	0.0172	ANO2	anoctamin 2, calcium activated chloride chan	Multiple_Complex
TC1900010746.hg.1	5.63	6.5	-1.83	0.005	0.0172	EXOSC5	exosome component 5	Multiple_Complex
TC2000009673.hg.1	6.63	5.68	1.93	0.005	0.0172	SYCP2	synaptonemal complex protein 2	Multiple_Complex
TC0100010664.hg.1	6.2	6.65	-1.37	0.005	0.0172	ZBTB37	zinc finger and BTB domain containing 37	Multiple_Complex
TC0100007800.hg.1	6.52	7.27	-1.68	0.005	0.0173	THRAP3	Jeck2013 ALT_ACCEPTOR, ALT_DONOR, codin	NonCoding
TC0700008873.hg.1	5.95	5.14	1.75	0.005	0.0173	CAV1	caveolin 1	Multiple_Complex
TC1100013206.hg.1	8.63	7.63	2.01	0.005	0.0173	STARD10	StAR-related lipid transfer domain containing	Multiple_Complex
TC0X00007230.hg.1	4.24	3.78	1.37	0.005	0.0173	GAGE2D; G	G antigen 2D; G antigen 13; G antigen 2E; G a	Coding
TC1100009792.hg.1	5.32	5.79	-1.39	0.005	0.0173	C11orf21	chromosome 11 open reading frame 21	Multiple_Complex
TC0200011130.hg.1	9.41	8.91	1.42	0.005	0.0173	DGKD	diacylglycerol kinase, delta 130kDa	Multiple_Complex
TC0700006523.hg.1	6.36	6.74	-1.31	0.005	0.0173	PSMG3-AS:	PSMG3 antisense RNA 1 (head to head)	NonCoding
TC1700011097.hg.1	8.39	7.87	1.44	0.0051	0.0173	LRRC59	leucine rich repeat containing 59	Multiple_Complex
TC0100015196.hg.1	9.19	9.91	-1.64	0.0051	0.0173	PSMA5	proteasome subunit alpha 5	Multiple_Complex
TC0X00009684.hg.1	4.22	3.72	1.41	0.0051	0.0173	SHROOM4	shroom family member 4	Multiple_Complex
TC0600009333.hg.1	7.18	6.43	1.68	0.0051	0.0173	SMPDL3A	sphingomyelin phosphodiesterase, acid-like 3	Multiple_Complex
TC1000008574.hg.1	10.45	11.22	-1.7	0.0051	0.0174	PGAM1	phosphoglycerate mutase 1 (brain)	Multiple_Complex
TC1100013000.hg.1	5.07	4.58	1.41	0.0051	0.0174	CLP1; AP00	Transcript Identified by AceView, Entrez Gene	NonCoding
TC0300008563.hg.1	10.5	9.67	1.79	0.0051	0.0174	DIRC2	disrupted in renal carcinoma 2	Multiple_Complex
TC1700010230.hg.1	7.73	8.22	-1.4	0.0051	0.0174	TIAF1; MYC	TGFB1-induced anti-apoptotic factor 1; myosi	Multiple_Complex
TC0600008931.hg.1	5.59	4.92	1.6	0.0051	0.0175	GRIK2	glutamate receptor, ionotropic, kainate 2	Multiple_Complex
TC0200008894.hg.1	6.34	5.52	1.76	0.0051	0.0175	MERTK	MER proto-oncogene, tyrosine kinase	Multiple_Complex
TC1900009742.hg.1	5.03	4.66	1.29	0.0051	0.0175	FBXW9	F-box and WD repeat domain containing 9	Multiple_Complex
TC0100010134.hg.1	9.13	8.68	1.37	0.0051	0.0175	DAP3	death associated protein 3	Multiple_Complex
TC1200009467.hg.1	6.41	6.84	-1.34	0.0051	0.0176	NOC4L	nucleolar complex associated 4 homolog	Multiple_Complex
TC1500006748.hg.1	5.49	6.2	-1.64	0.0051	0.0176	FAN1	FANCD2/FANCI-associated nuclease 1	Multiple_Complex
TC1500010887.hg.1	5.62	6.21	-1.5	0.0051	0.0176	CLN6	ceroid-lipofuscinosis, neuronal 6, late infantil	Multiple_Complex
TC1600011315.hg.1	8.25	8.78	-1.45	0.0052	0.0176	FAM234A;	family with sequence similarity 234, member	Multiple_Complex
TC0100016157.hg.1	6.29	5.87	1.34	0.0052	0.0177	PVRL4	poliovirus receptor-related 4	Multiple_Complex
TSUnmapped00000182.h	3.77	4.72	-1.92	0.0052	0.0177	ZKSCAN7	zinc finger with KRAB and SCAN domains 7	Coding
TC1900011774.hg.1	15.96	16.26	-1.24	0.0052	0.0177	EMP3	epithelial membrane protein 3	Multiple_Complex
TC1300007045.hg.1	5.94	6.69	-1.69	0.0052	0.0177	GPALPP1	GPALPP motifs containing 1	Multiple_Complex
TC0400010785.hg.1	6.94	8.43	-2.81	0.0052	0.0177	IGFBP7	insulin like growth factor binding protein 7	Multiple_Complex
TC1700012053.hg.1	5.15	5.78	-1.55	0.0052	0.0177	FAAP100	Fanconi anemia core complex associated prot	Multiple_Complex
TC0500013253.hg.1	10.84	11.34	-1.42	0.0052	0.0177	RBM27	RNA binding motif protein 27	Multiple_Complex
TC0300010982.hg.1	7.35	7.92	-1.49	0.0052	0.0177	SCAP	SREBF chaperone	Multiple_Complex
TC1900009896.hg.1	5.24	4.83	1.33	0.0052	0.0177	CYP4F11	cytochrome P450, family 4, subfamily F, polyp	Multiple_Complex
TC0500009072.hg.1	5.28	5.82	-1.46	0.0052	0.0177	PPARGC1B	peroxisome proliferator-activated receptor ga	Multiple_Complex
TC0700013604.hg.1	9.43	9.88	-1.37	0.0052	0.0177	UPK3BL; PC	uroplakin 3B-like; polymerase (RNA) II (DNA d	Multiple_Complex
TC1200010641.hg.1	4.07	4.58	-1.43	0.0052	0.0177	BCDIN3D	BCDIN3 domain containing	Multiple_Complex
TC1200007277.hg.1	7.39	6.85	1.45	0.0052	0.0177	DNM1L	dynamin 1-like	Multiple_Complex
TC1900007952.hg.1	5.57	5.89	-1.25	0.0052	0.0177	LOC728485	uncharacterized LOC728485; novel transcript	Multiple_Complex
TC0700011898.hg.1	5.7	6.1	-1.32	0.0052	0.0178	TECPR1	tectonin beta-propeller repeat containing 1	Multiple_Complex
TC0X00007539.hg.1	4.03	3.53	1.42	0.0052	0.0178	GDPD2	glycerophosphodiester phosphodiesterase do	Multiple_Complex
TC0500011208.hg.1	7.86	7.16	1.63	0.0052	0.0178	WDR41	WD repeat domain 41	Multiple_Complex
TC2000007537.hg.1	4.05	3.51	1.45	0.0052	0.0178	SLC2A10	solute carrier family 2 (facilitated glucose tran	Multiple_Complex
TC0200007059.hg.1	5.65	6.24	-1.51	0.0052	0.0179	CAD	carbamoyl-phosphate synthetase 2, aspartate	Multiple_Complex
TC0100010255.hg.1	4.38	3.79	1.5	0.0052	0.0179	OR10J4	olfactory receptor, family 10, subfamily J, me	Pseudogene
TC0X00008759.hg.1	5.71	6.21	-1.42	0.0052	0.0179	NSDHL	NAD(P) dependent steroid dehydrogenase-lik	Multiple_Complex
TC1900008839.hg.1	5.78	5.19	1.5	0.0052	0.0179	TSEN34	TSEN34 tRNA splicing endonuclease subunit	Multiple_Complex
TC1200008224.hg.1	13.48	14.14	-1.59	0.0053	0.0179	GLIPR1	GLI pathogenesis-related 1	Multiple_Complex
TC1900011639.hg.1	8.73	9.27	-1.46	0.0053	0.0179	STK11	serine/threonine kinase 11	Multiple_Complex
TC1900011916.hg.1	9.66	10.18	-1.44	0.0053	0.0179	ZNF626	zinc finger protein 626	Multiple_Complex
TC2000006622.hg.1	6.6	7	-1.31	0.0053	0.0179	C20orf196	chromosome 20 open reading frame 196	Multiple_Complex

TC2000007150.hg.1	3.54	3.25	1.23	0.0053	0.0179	BPIFB1	BPI fold containing family B, member 1	Multiple_Complex
TC0200009596.hg.1	7.87	7.16	1.63	0.0053	0.0179	ACVR2A	activin A receptor type IIA	Multiple_Complex
TC0200016193.hg.1	4.13	3.67	1.38	0.0053	0.018	COL6A3	collagen, type VI, alpha 3	Multiple_Complex
TC0100008845.hg.1	4.13	3.69	1.36	0.0053	0.018	ADGRL2	adhesion G protein-coupled receptor L2	Multiple_Complex
TC0700008898.hg.1	4.33	3.92	1.33	0.0053	0.018	ANKRD7	ankyrin repeat domain 7	Multiple_Complex
TC0800009242.hg.1	6.33	6.69	-1.28	0.0053	0.018	HGH1	HGH1 homolog	Multiple_Complex
TC0700009693.hg.1	7.53	7.92	-1.31	0.0053	0.018	AGAP3	ArfGAP with GTPase domain, ankyrin repeat a	Multiple_Complex
TC2200007574.hg.1	6.38	5.55	1.79	0.0053	0.018	PARVB	parvin, beta	Multiple_Complex
TC1900011806.hg.1	5.27	5.96	-1.61	0.0053	0.0181	ZNF582-AS	ZNF582 antisense RNA 1 (head to head)	NonCoding
TC0500009223.hg.1	5	5.67	-1.59	0.0053	0.0181	THG1L	tRNA-histidine guanylyltransferase 1-like	Multiple_Complex
TC1600010517.hg.1	5.36	4.86	1.42	0.0053	0.0181	LOC729159	UPF0607 protein ENSP00000381418-like; Puta	Coding
TC0200009902.hg.1	4.38	3.83	1.47	0.0053	0.0181	NOSTRIN	nitric oxide synthase trafficking	Multiple_Complex
TC0200009680.hg.1	4.66	5.47	-1.76	0.0053	0.0181	FMNL2	formin like 2	Multiple_Complex
TC0400011108.hg.1	11.1	11.74	-1.56	0.0053	0.0181	CNOT6L	CCR4-NOT transcription complex subunit 6-lik	Multiple_Complex
TC0300009167.hg.1	7.53	7.03	1.42	0.0053	0.0181	EIF2A	eukaryotic translation initiation factor 2A, 65k	Multiple_Complex
TC0600014095.hg.1	5.17	5.54	-1.29	0.0053	0.0181	RPP21	ribonuclease P/MRP 21kDa subunit	Multiple_Complex
TC1400008486.hg.1	4.59	4.89	-1.23	0.0053	0.0181	CRIP2	cysteine-rich protein 2	Multiple_Complex
TC1000006639.hg.1	8.66	9.25	-1.5	0.0053	0.0181	FBXO18	F-box protein, helicase, 18	Multiple_Complex
TC0200016604.hg.1	4.73	4.06	1.6	0.0053	0.0181	RBM44	RNA binding motif protein 44	Multiple_Complex
TC1600007753.hg.1	9.91	10.11	-1.15	0.0053	0.0182	LONP2	lon peptidase 2, peroxisomal	Multiple_Complex
TC1900009198.hg.1	7.55	8.08	-1.45	0.0054	0.0182	BTBD2	BTB (POZ) domain containing 2	Multiple_Complex
TC2200008528.hg.1	6.04	5.42	1.54	0.0054	0.0182	LARGE	like-glycosyltransferase	Multiple_Complex
TC0200007257.hg.1	7.18	7.72	-1.46	0.0054	0.0182	NDUFAF7	NADH dehydrogenase (ubiquinone) complex I	Multiple_Complex
TC0100018361.hg.1	7.82	7.34	1.39	0.0054	0.0182	TSNAX-DISC	TSNAX-DISC1 readthrough (NMD candidate)	Multiple_Complex
TC1100007307.hg.1	5.68	5.24	1.35	0.0054	0.0182	C11orf74	chromosome 11 open reading frame 74	Multiple_Complex
TC0200014912.hg.1	6.36	6.01	1.27	0.0054	0.0183	DLX2	distal-less homeobox 2	Coding
TC1900009062.hg.1	7.69	8.36	-1.6	0.0054	0.0183	TRIM28	tripartite motif containing 28	Multiple_Complex
TC0800007132.hg.1	4.54	5.02	-1.4	0.0054	0.0183	PNOC	prepronociceptin	Multiple_Complex
TC1500006930.hg.1	5.83	6.4	-1.49	0.0054	0.0183	EIF2AK4	eukaryotic translation initiation factor 2 alpha	Multiple_Complex
TC0300006566.hg.1	5.12	5.62	-1.42	0.0054	0.0183	CRELD1	cysteine rich with EGF-like domains 1	Multiple_Complex
TC1700012309.hg.1	8.55	9.12	-1.49	0.0054	0.0183	METTL23	methyltransferase like 23	Multiple_Complex
TC0600009080.hg.1	5.89	5.16	1.66	0.0054	0.0183	CEP57L1	centrosomal protein 57kDa-like 1	Multiple_Complex
TC0200016318.hg.1	5.65	5.23	1.34	0.0054	0.0184	PP14571; A	uncharacterized LOC100130449; novel transcr	Multiple_Complex
TC1200011657.hg.1	7.33	6.72	1.53	0.0054	0.0184	ANKS1B	ankyrin repeat and sterile alpha motif domain	Multiple_Complex
TC0Y00006528.hg.1	6.95	8.25	-2.47	0.0055	0.0185	PRKY	protein kinase, Y-linked, pseudogene	Multiple_Complex
TC1900008987.hg.1	3.91	3.42	1.4	0.0055	0.0185	AURKC	aurora kinase C	Coding
TC0100015580.hg.1	10.35	9.91	1.36	0.0055	0.0185	NBPF20	neuroblastoma breakpoint family, member 20	Coding
TC0700007811.hg.1	8.1	8.5	-1.32	0.0055	0.0185	INTS4P2	integrator complex subunit 4 pseudogene 2	Multiple_Complex
TC0100015781.hg.1	6.77	7.23	-1.37	0.0055	0.0186	PI4KB	phosphatidylinositol 4-kinase, catalytic, beta	Multiple_Complex
TSUnmapped00000052.h	4.78	4.28	1.42	0.0055	0.0186	HHAT	hedgehog acyltransferase	Coding
TC0800007382.hg.1	4.99	5.75	-1.7	0.0055	0.0186	IDO1	indoleamine 2,3-dioxygenase 1	Multiple_Complex
TC0900011778.hg.1	8	8.5	-1.41	0.0055	0.0186	RAPGEF1	Rap guanine nucleotide exchange factor 1	Multiple_Complex
TC0500013235.hg.1	11.47	10.78	1.61	0.0055	0.0186	FAM53C	family with sequence similarity 53, member C	Multiple_Complex
TC1300007070.hg.1	7.74	8.42	-1.6	0.0055	0.0186	COG3	component of oligomeric golgi complex 3	Multiple_Complex
TC1200008535.hg.1	6.75	7.57	-1.77	0.0055	0.0186	ELK3	ELK3, ETS-domain protein (SRF accessory prot	Multiple_Complex
TC0200011070.hg.1	6.53	6.84	-1.24	0.0055	0.0186	C2orf57	chromosome 2 open reading frame 57	Coding
TC0100008996.hg.1	6.89	6.23	1.58	0.0055	0.0187	ZNF326	zinc finger protein 326	Multiple_Complex
TC1400007300.hg.1	7.47	6.63	1.79	0.0055	0.0187	PSMA3	proteasome subunit alpha 3	Multiple_Complex
TC1700007674.hg.1	6.43	7.02	-1.5	0.0055	0.0187	TADA2A	transcriptional adaptor 2A	Multiple_Complex
TC0X00009636.hg.1	7	6.13	1.82	0.0055	0.0187	SLC35A2	solute carrier family 35 (UDP-galactose transp	Multiple_Complex
TC1900011914.hg.1	6.59	7.21	-1.54	0.0055	0.0187	ZNF506	zinc finger protein 506	Multiple_Complex
TC0900010147.hg.1	6.75	5.67	2.11	0.0055	0.0187	CNTNAP3B	contactin associated protein-like 3B	Multiple_Complex
TC0700009065.hg.1	5.51	6.13	-1.54	0.0055	0.0187	CCDC136	coiled-coil domain containing 136	Multiple_Complex
TC1500008139.hg.1	6.22	5.8	1.33	0.0055	0.0187	GOLGA6L4	Homo sapiens golgin A6 family-like 4 (GOLGA6	Multiple_Complex
TC0500012864.hg.1	5.02	4.77	1.19	0.0056	0.0188	NKX2-5	NK2 homeobox 5	Coding
TC0800010084.hg.1	6.04	5.76	1.22	0.0056	0.0188	DUSP26	dual specificity phosphatase 26 (putative)	Coding
TC1500010465.hg.1	5.97	6.58	-1.53	0.0056	0.0188	VPS33B	vacuolar protein sorting 33 homolog B (yeast)	Multiple_Complex
TC0200014738.hg.1	8.18	8.76	-1.49	0.0056	0.0188	RBMS1	Memczak2013 ALT_ACCEPTOR, ALT_DONOR,	NonCoding
TC2100008517.hg.1	7.92	7.52	1.32	0.0056	0.0188	WRB	tryptophan rich basic protein	Multiple_Complex
TC0500013334.hg.1	7.75	7.31	1.36	0.0056	0.0188	DMGDH	dimethylglycine dehydrogenase	NonCoding
TC0200008627.hg.1	5.26	5.87	-1.52	0.0056	0.0189	NPAS2	neuronal PAS domain protein 2	Multiple_Complex
TC0300007409.hg.1	5.12	4.62	1.41	0.0056	0.0189	SLC38A3	solute carrier family 38, member 3	Multiple_Complex
TC0100015160.hg.1	8.8	8.36	1.36	0.0056	0.0189	SLC25A24	solute carrier family 25 (mitochondrial carrier;	Multiple_Complex
TC1400009967.hg.1	6.88	5.84	2.05	0.0056	0.0189	TTC7B	tetratricopeptide repeat domain 7B	Multiple_Complex
TC0700012960.hg.1	5.57	4.58	1.98	0.0056	0.0189	EZH2	Transcript Identified by AceView, Entrez Gene	Unassigned
TC1200012853.hg.1	4.83	4.38	1.37	0.0056	0.0189	NOS1	nitric oxide synthase 1 (neuronal)	Multiple_Complex
TC0100010764.hg.1	5.43	5.84	-1.33	0.0056	0.0189	TOR3A	torsin family 3, member A	Multiple_Complex
TC1500010854.hg.1	5.19	4.58	1.52	0.0056	0.0189	GPR176	G protein-coupled receptor 176	Multiple_Complex
TC0300008542.hg.1	6.97	7.85	-1.84	0.0056	0.0189	CD86	CD86 molecule	Multiple_Complex
TC0600007231.hg.1	6.14	6.65	-1.42	0.0056	0.019	LRRC16A	leucine rich repeat containing 16A	Multiple_Complex

TC1900007802.hg.1	5.96	6.44	-1.4	0.0056	0.019	KIAA0355	KIAA0355	Multiple_Complex
TC1100007026.hg.1	6.64	7.24	-1.52	0.0056	0.019	ZDHHC13	zinc finger, DHHC-type containing 13	Multiple_Complex
TSUnmapped00000224.h	7.17	6.76	1.33	0.0056	0.019	ZDHHC3	zinc finger, DHHC-type containing 3	NonCoding
TC1100007778.hg.1	4.48	3.95	1.44	0.0056	0.019	MS4A18	membrane-spanning 4-domains, subfamily A,	Multiple_Complex
TC1900010708.hg.1	8.09	9.04	-1.93	0.0057	0.019	BLVRB	biliverdin reductase B	Multiple_Complex
TC2100007462.hg.1	6.1	6.53	-1.35	0.0057	0.019	YBEY	ybeY metallopeptidase (putative)	Multiple_Complex
TC1100011744.hg.1	7.86	6.82	2.06	0.0057	0.019	GAB2	GRB2-associated binding protein 2	Multiple_Complex
TC2200009186.hg.1	7.22	7.59	-1.29	0.0057	0.019	PEX26	peroxisomal biogenesis factor 26	Multiple_Complex
TC2000007110.hg.1	5.12	5.47	-1.27	0.0057	0.019	POFUT1; M	protein O-fucosyltransferase 1; microRNA 182	Multiple_Complex
TC0900011631.hg.1	8.46	8.06	1.32	0.0057	0.0191	GOLGA2	golgin A2	Multiple_Complex
TC1900011571.hg.1	5.92	6.76	-1.78	0.0057	0.0191	ZNF550	zinc finger protein 550	Multiple_Complex
TC0100011648.hg.1	8.02	8.92	-1.88	0.0057	0.0191	IARS2	isoleucyl-tRNA synthetase 2, mitochondrial	Multiple_Complex
TC0500008541.hg.1	4.66	4.36	1.23	0.0057	0.0191	TEX43	testis expressed 43	Multiple_Complex
TC1900008036.hg.1	5.05	5.4	-1.27	0.0057	0.0191	MRPS12	mitochondrial ribosomal protein S12	Multiple_Complex
TC1300009008.hg.1	6.54	7	-1.38	0.0057	0.0191	NEK3	NIMA-related kinase 3	Multiple_Complex
TC1400007769.hg.1	5.25	5.8	-1.47	0.0057	0.0192	ADCK1	aarF domain containing kinase 1	Multiple_Complex
TC1900008445.hg.1	5.77	5.39	1.3	0.0057	0.0192	ZNF114	zinc finger protein 114	Multiple_Complex
TC0200016414.hg.1	5.96	6.42	-1.38	0.0057	0.0192	FAM228B	family with sequence similarity 228, member	Multiple_Complex
TC0400012068.hg.1	5.65	6.32	-1.6	0.0057	0.0193	PRMT9	protein arginine methyltransferase 9	Multiple_Complex
TC0100013619.hg.1	6.34	5.65	1.62	0.0058	0.0193	ZBTB8OS	zinc finger and BTB domain containing 8 oppo	Multiple_Complex
TC2100008559.hg.1	6.78	7.53	-1.68	0.0058	0.0194	C21orf59	chromosome 21 open reading frame 59	Multiple_Complex
TC1700009541.hg.1	9.67	10.43	-1.68	0.0058	0.0194	CAMTA2	calmodulin binding transcription activator 2	Multiple_Complex
TC1600010633.hg.1	13.72	12.9	1.77	0.0058	0.0195	ATP6V0D1	ATPase, H+ transporting, lysosomal 38kDa, V0	Multiple_Complex
TC0700007996.hg.1	6.68	6.3	1.31	0.0058	0.0195	ELN	elastin	Multiple_Complex
TC1900011635.hg.1	10.59	9.87	1.65	0.0058	0.0195	CHMP2A	charged multivesicular body protein 2A	Multiple_Complex
TC0300013926.hg.1	6.04	6.69	-1.57	0.0058	0.0195	SUMF1	sulfatase modifying factor 1	Multiple_Complex
TC0600007289.hg.1	5.63	4.6	2.04	0.0058	0.0196	HIST1H4F	histone cluster 1, H4f	Coding
TC0X00010875.hg.1	5.67	5.16	1.43	0.0058	0.0196	PLAC1	placenta specific 1	Multiple_Complex
TC1100008067.hg.1	13.53	14.43	-1.87	0.0058	0.0196	RAB1B	RAB1B, member RAS oncogene family	Multiple_Complex
TC0700008868.hg.1	11.51	12	-1.41	0.0058	0.0196	TES	testin LIM domain protein	Multiple_Complex
TC0100018555.hg.1	5.02	4.58	1.35	0.0058	0.0196	LEMD1	LEM domain containing 1	Multiple_Complex
TC0600012562.hg.1	4.66	5.37	-1.63	0.0059	0.0196	BACH2	Memczak2013 ALT_ACCEPTOR, ALT_DONOR,	NonCoding
TC0X00006709.hg.1	4.15	4.84	-1.61	0.0059	0.0196	NHS	Nance-Horan syndrome (congenital cataracts	Multiple_Complex
TC1200009252.hg.1	6.32	6.92	-1.51	0.0059	0.0196	TCTN2	tectonic family member 2	Multiple_Complex
TC0700012824.hg.1	5.73	5.06	1.58	0.0059	0.0196	MRPS33	mitochondrial ribosomal protein S33	Multiple_Complex
TC0100009199.hg.1	12.06	11.63	1.35	0.0059	0.0196	MFSD14A	major facilitator superfamily domain containi	Multiple_Complex
TC1900011836.hg.1	5.9	6.45	-1.47	0.0059	0.0196	MBD3	methyl-CpG binding domain protein 3	Multiple_Complex
TC1100009168.hg.1	9.15	9.79	-1.56	0.0059	0.0197	SIDT2	SID1 transmembrane family, member 2	Multiple_Complex
TC0100014188.hg.1	10.72	10.24	1.4	0.0059	0.0197	NRDC; MIR	nardilysin convertase; microRNA 761	Multiple_Complex
TC1600011312.hg.1	3.74	5.33	-3.02	0.0059	0.0198	HBZ	hemoglobin, zeta	Multiple_Complex
TC0200016220.hg.1	7.21	6.6	1.52	0.0059	0.0198	ILKAP	ILK associated serine/threonine phosphatase	Multiple_Complex
TC0600008346.hg.1	7.34	8.14	-1.75	0.0059	0.0198	KIAA1586	KIAA1586	Multiple_Complex
TC1500010106.hg.1	6.71	7.27	-1.47	0.0059	0.0198	TSPAN3	tetraspanin 3	Multiple_Complex
TC0X00008138.hg.1	3.93	4.72	-1.72	0.0059	0.0198	ALG13	Transcript Identified by AceView, Entrez Gene	Unassigned
TC0500007972.hg.1	14.89	13.05	3.56	0.0059	0.0199	VCAN	versican	Multiple_Complex
TC1500010872.hg.1	5.1	5.5	-1.32	0.0059	0.0199	ZNF280D	zinc finger protein 280D	NonCoding
TC0100009846.hg.1	4.61	5.27	-1.59	0.006	0.0199	PDE4DIP	Transcript Identified by AceView, Entrez Gene	Unassigned
TC1200008275.hg.1	4.37	4.01	1.29	0.006	0.0199	SYT1	synaptotagmin I	Multiple_Complex
TC0X00007390.hg.1	4.58	5.13	-1.46	0.006	0.02	KLF8	Kruppel-like factor 8	Multiple_Complex
TC1900008358.hg.1	6.16	6.67	-1.42	0.006	0.02	CCDC61	coiled-coil domain containing 61	Multiple_Complex
TC1000012403.hg.1	4.6	4.19	1.33	0.006	0.02	SPRN	shadow of prion protein homolog (zebrafish)	Coding
TC1000011683.hg.1	9.53	10.05	-1.43	0.006	0.02	ACTR1A	ARP1 actin-related protein 1 homolog A, centr	Multiple_Complex
TC0200016747.hg.1	4.86	5.61	-1.68	0.006	0.02	TTC21B	tetratricopeptide repeat domain 21B	Multiple_Complex
TC0200010972.hg.1	9.99	9.32	1.59	0.006	0.02	MFF	mitochondrial fission factor	Multiple_Complex
TC2200009260.hg.1	7.56	7.14	1.34	0.006	0.0201	DRG1	developmentally regulated GTP binding protei	Multiple_Complex
TC0400008557.hg.1	6.24	6.88	-1.56	0.006	0.0201	USP53	ubiquitin specific peptidase 53	Multiple_Complex
TC1100007704.hg.1	4.11	3.67	1.36	0.006	0.0201	OR9Q2	olfactory receptor, family 9, subfamily Q, men	Coding
TC0200008168.hg.1	4.19	3.7	1.41	0.006	0.0201	REG1A	regenerating islet-derived 1 alpha	Multiple_Complex
TC0900011192.hg.1	7.09	7.74	-1.57	0.006	0.0201	LPAR1	lysophosphatidic acid receptor 1	Multiple_Complex
TC0200013616.hg.1	7.23	8.23	-2	0.0061	0.0203	AFF3	AF4/FMR2 family, member 3	Multiple_Complex
TC0900009114.hg.1	9.39	9.86	-1.39	0.0061	0.0203	RXRA; MIR4	retinoid X receptor alpha; microRNA 4669	Multiple_Complex
TC1700006602.hg.1	6.48	5.98	1.42	0.0061	0.0203	ATP2A3	Memczak2013 ANTISENSE, CDS, coding, INTEF	NonCoding
TC0500012938.hg.1	8.91	8.35	1.47	0.0061	0.0203	CLTB	clathrin, light chain B	Multiple_Complex
TC0100013608.hg.1	5.43	5.8	-1.29	0.0061	0.0203	MARCKSL1	MARCKS-like 1	Multiple_Complex
TSUnmapped00000018.h	4.77	5.16	-1.31	0.0061	0.0203	ZNF35	zinc finger protein 35	Coding
TC0600009041.hg.1	3.91	3.47	1.35	0.0061	0.0204	NR2E1	nuclear receptor subfamily 2, group E, membe	Multiple_Complex
TC1500006977.hg.1	7.23	6.62	1.52	0.0061	0.0204	VPS18	VPS18 CORVET/HOPS core subunit	Multiple_Complex
TC0700011968.hg.1	6.64	7.21	-1.48	0.0061	0.0204	ZNF3	zinc finger protein 3	Multiple_Complex
TC0100012475.hg.1	8.02	8.6	-1.5	0.0061	0.0204	MRPL20	mitochondrial ribosomal protein L20	Multiple_Complex
TC1700012384.hg.1	5.9	6.24	-1.26	0.0061	0.0204	RAD51L3-R	RAD51L3-RFFL readthrough; HCG2039718, iso	Multiple_Complex

TC0800007888.hg.1	6.07	5.3	1.71	0.0061	0.0204	C8orf34	chromosome 8 open reading frame 34	Multiple_Complex
TC0200016687.hg.1	10.08	9.31	1.7	0.0061	0.0204	SFXN5	sideroflexin 5	Multiple_Complex
TC1700008844.hg.1	8.21	8.8	-1.51	0.0061	0.0204	TTYH2	tweety family member 2	Multiple_Complex
TC1100009929.hg.1	3.81	3.35	1.38	0.0061	0.0204	OR51I1	olfactory receptor, family 51, subfamily I, mer	Coding
TC1700008191.hg.1	4.1	3.67	1.35	0.0061	0.0204	B4GALNT2	beta-1,4-N-acetyl-galactosaminyl transferase :	Multiple_Complex
TC0200008499.hg.1	5.77	5.13	1.56	0.0061	0.0204	STARD7-AS	STARD7 antisense RNA 1	Multiple_Complex
TC0100017022.hg.1	3.97	3.56	1.33	0.0061	0.0205	GOLT1A	golgi transport 1A	Multiple_Complex
TC2100006674.hg.1	5.61	5.09	1.44	0.0062	0.0205	CHODL	chondrolectin	Multiple_Complex
TC0100007564.hg.1	5.5	5.2	1.23	0.0062	0.0205	RAB42	RAB42, member RAS oncogene family	Multiple_Complex
TC0600007446.hg.1	4.46	3.96	1.41	0.0062	0.0205	OR2J1	olfactory receptor, family 2, subfamily J, mem	Pseudogene
TC1700012193.hg.1	11.74	11.06	1.6	0.0062	0.0206	NDEL1	nudE neurodevelopment protein 1-like 1	Multiple_Complex
TC0200013376.hg.1	8.07	7.61	1.37	0.0062	0.0206	EIF2AK3	eukaryotic translation initiation factor 2-alpha	Multiple_Complex
TC1300007208.hg.1	10.25	9.67	1.5	0.0062	0.0206	RNASEH2B	ribonuclease H2, subunit B	Multiple_Complex
TC0X00010355.hg.1	3.89	4.46	-1.49	0.0062	0.0207	TIMM8A	translocase of inner mitochondrial membrane	Multiple_Complex
TC1200012804.hg.1	5.49	6.11	-1.53	0.0062	0.0207	PRIM1	primase, DNA, polypeptide 1 (49kDa)	Multiple_Complex
TC0200007835.hg.1	16.4	15.8	1.51	0.0062	0.0207	ACTR2	ARP2 actin-related protein 2 homolog (yeast)	Multiple_Complex
TC1200008669.hg.1	6.92	6.46	1.37	0.0062	0.0207	TDG	thymine DNA glycosylase	Multiple_Complex
TC1700011294.hg.1	8.15	8.71	-1.47	0.0062	0.0207	PTRH2	peptidyl-tRNA hydrolase 2	Multiple_Complex
TC0300010026.hg.1	3.75	4.25	-1.42	0.0062	0.0207	FBXO45	F-box protein 45	Coding
TC1000011499.hg.1	6.81	7.23	-1.34	0.0062	0.0207	ALDH18A1	aldehyde dehydrogenase 18 family, member A	Multiple_Complex
TC0100008271.hg.1	11.26	10.02	2.37	0.0063	0.0208	RNF11	ring finger protein 11	Multiple_Complex
TC0100007333.hg.1	11.08	11.85	-1.71	0.0063	0.0208	PITHD1	PITH (C-terminal proteasome-interacting dom	Multiple_Complex
TC0600012432.hg.1	5.92	6.31	-1.31	0.0063	0.0208	PGM3	phosphoglucomutase 3	Multiple_Complex
TC0100018532.hg.1	7.28	7.83	-1.46	0.0063	0.0209	PIGC	phosphatidylinositol glycan anchor biosynthes	Multiple_Complex
TC1900008544.hg.1	7.95	7.3	1.58	0.0063	0.0209	RCN3	reticulocalbin 3, EF-hand calcium binding dom	Multiple_Complex
TC1600006737.hg.1	4.34	4.01	1.26	0.0063	0.0209	VASN	vasorin	Coding
TC1600006597.hg.1	6.4	6.71	-1.23	0.0063	0.0209	MLST8	MTOR associated protein, LST8 homolog	Multiple_Complex
TC1600009193.hg.1	5.25	5.61	-1.28	0.0063	0.0209	ZSCAN32	zinc finger and SCAN domain containing 32	Multiple_Complex
TC0600006864.hg.1	6.5	7.22	-1.64	0.0063	0.0209	RIOK1	RIO kinase 1	Multiple_Complex
TSUnmapped00000537.h	8.3	7.77	1.44	0.0063	0.0209	PRAMEF6	PRAME family member 6 [Source:HGNC Symb	Coding
TSUnmapped00000781.h	8.3	7.77	1.44	0.0063	0.0209	PRAMEF6	PRAME family member 6 [Source:HGNC Symb	Coding
TC0300008140.hg.1	4.65	4	1.56	0.0063	0.0209	TMEM30C	transmembrane protein 30C	Multiple_Complex
TC1900011673.hg.1	7.37	8.19	-1.77	0.0063	0.0209	CCDC130	coiled-coil domain containing 130	Multiple_Complex
TC1600007489.hg.1	8.32	7.76	1.47	0.0063	0.0209	RNF40	ring finger protein 40, E3 ubiquitin protein liga	Multiple_Complex
TC0200010252.hg.1	7.95	7.14	1.75	0.0063	0.021	ASNSD1	asparagine synthetase domain containing 1	Multiple_Complex
TC2000008759.hg.1	5.59	5.14	1.36	0.0063	0.021	FAM182B	family with sequence similarity 182, member	Multiple_Complex
TC1000006648.hg.1	9.06	8.58	1.4	0.0063	0.021	RBM17	RNA binding motif protein 17	Multiple_Complex
TC0500010580.hg.1	12.58	12.11	1.39	0.0063	0.021	PRKAA1	protein kinase, AMP-activated, alpha 1 catalyt	Multiple_Complex
TC1900010503.hg.1	17.44	18.2	-1.69	0.0063	0.021	TYROBP	TYRO protein tyrosine kinase binding protein	Multiple_Complex
TC1800008977.hg.1	9.16	8.25	1.89	0.0064	0.0211	CD226	CD226 molecule	Multiple_Complex
TC1300008824.hg.1	6.05	6.57	-1.43	0.0064	0.0211	SLC25A30	solute carrier family 25, member 30	Multiple_Complex
TSUnmapped00000599.h	4.73	4.43	1.24	0.0064	0.0211	PRAMEF8	PRAME family member 8	Coding
TC1600010627.hg.1	4.72	5.27	-1.47	0.0064	0.0211	TPPP3	tubulin polymerization-promoting protein fam	Multiple_Complex
TC1000009879.hg.1	4.27	3.89	1.3	0.0064	0.0211	C10orf111	chromosome 10 open reading frame 111	Multiple_Complex
TC0300009363.hg.1	3.8	3.36	1.36	0.0064	0.0212	OTOL1	otolin 1	Coding
TC0600008462.hg.1	3.53	3.88	-1.27	0.0064	0.0212	COL19A1	collagen, type XIX, alpha 1	Multiple_Complex
TC0900009922.hg.1	5.3	4.84	1.38	0.0064	0.0212	FAM205C	family with sequence similarity 205, member	Multiple_Complex
TC0400007770.hg.1	5.72	4.9	1.76	0.0064	0.0213	STATH	statherin	Multiple_Complex
TC1400010737.hg.1	8.42	8.86	-1.36	0.0064	0.0213	KLHL28	kelch-like family member 28	Multiple_Complex
TC1600011317.hg.1	9.21	9.55	-1.26	0.0064	0.0213	NME4	NME/NM23 nucleoside diphosphate kinase 4	Multiple_Complex
TC1600008496.hg.1	5.12	5.64	-1.43	0.0064	0.0213	NUDT7	nudix hydrolase 7	Multiple_Complex
TC1700012391.hg.1	4.68	5.46	-1.72	0.0064	0.0213	MYO19	myosin XIX	Multiple_Complex
TC1000009916.hg.1	5.36	6.15	-1.73	0.0065	0.0213	CUBN	cubilin (intrinsic factor-cobalamin receptor)	Multiple_Complex
TC1600011343.hg.1	4.74	5.81	-2.1	0.0065	0.0213	CLUAP1	clusterin associated protein 1	Multiple_Complex
TC0500008544.hg.1	10.2	9.05	2.22	0.0065	0.0213	LMNB1	lamin B1	Multiple_Complex
TC0500012670.hg.1	3.75	3.34	1.32	0.0065	0.0213	GABRB2	gamma-aminobutyric acid (GABA) A receptor,	Multiple_Complex
TC2000006813.hg.1	5.75	6.19	-1.36	0.0065	0.0214	MGME1	mitochondrial genome maintenance exonucle	Multiple_Complex
TC0700012996.hg.1	7.33	7.84	-1.42	0.0065	0.0214	ZNF746	zinc finger protein 746	Multiple_Complex
TC0300007517.hg.1	6.24	5.8	1.36	0.0065	0.0214	CACNA1D	calcium channel, voltage-dependent, L type, a	Multiple_Complex
TC0600008601.hg.1	4.47	5	-1.45	0.0065	0.0214	IRAK1BP1	interleukin 1 receptor associated kinase 1 binc	Multiple_Complex
TC1000008474.hg.1	7.68	8.49	-1.75	0.0065	0.0214	HHEX	hematopoietically expressed homeobox	Multiple_Complex
TC2000008650.hg.1	6.02	5.63	1.31	0.0065	0.0214	THBD	thrombomodulin	Coding
TC1500006959.hg.1	6.51	6.89	-1.3	0.0065	0.0215	CHST14	carbohydrate (N-acetylgalactosamine 4-0) sulf	Coding
TC0900011385.hg.1	8.38	9.02	-1.56	0.0065	0.0215	PSMD5	proteasome 26S subunit, non-ATPase 5	Multiple_Complex
TC0700013599.hg.1	6.44	6.12	1.25	0.0065	0.0215	ACHE	acetylcholinesterase (Yt blood group)	Coding
TC1200007425.hg.1	9.17	9.69	-1.44	0.0065	0.0216	ARID2	AT rich interactive domain 2 (ARID, RFX-like)	Multiple_Complex
TSUnmapped00000006.h	6.63	6.07	1.48	0.0065	0.0216	RPS6KA1	ribosomal protein S6 kinase, 90kDa, polypepti	Coding
TC0100017084.hg.1	5.09	6.32	-2.34	0.0066	0.0216	CTSE	cathepsin E	Multiple_Complex
TC0100017261.hg.1	4.61	3.9	1.63	0.0066	0.0217	SPATA45	spermatogenesis associated 45	Multiple_Complex
TC1400006552.hg.1	5.09	4.75	1.27	0.0066	0.0217	RP11-998D	Transcript Identified by AceView, Entrez Gene	NonCoding

TC2200006630.hg.1	9.62	10.58	-1.94	0.0066	0.0217	TANGO2	transport and golgi organization 2 homolog	Multiple_Complex
TC1100010054.hg.1	3.95	3.3	1.56	0.0066	0.0218	NRIP3	nuclear receptor interacting protein 3	Multiple_Complex
TC1700006899.hg.1	11.25	10.69	1.48	0.0066	0.0218	MAP2K4; M	mitogen-activated protein kinase kinase 4; mi	Multiple_Complex
TC0200008955.hg.1	6.46	7.05	-1.51	0.0066	0.0218	PSD4	pleckstrin and Sec7 domain containing 4	Multiple_Complex
TC1100010393.hg.1	6.21	5.74	1.39	0.0066	0.0218	SLC5A12	solute carrier family 5 (sodium/monocarboxyl	Multiple_Complex
TC0600007158.hg.1	6.3	6.01	1.22	0.0066	0.0218	SOX4	SRY box 4	Multiple_Complex
TC2100008171.hg.1	5.17	4.28	1.86	0.0066	0.0218	LCA5L	Leber congenital amaurosis 5-like	Multiple_Complex
TC0X00006701.hg.1	5.01	5.64	-1.55	0.0066	0.0218	TXLNG	taxilin gamma	Multiple_Complex
TC0700009770.hg.1	4.27	3.74	1.44	0.0066	0.0218	DPP6	dipeptidyl-peptidase 6	Multiple_Complex
TC1900006662.hg.1	5.07	4.56	1.43	0.0066	0.0218	NMRK2	nicotinamide riboside kinase 2	Multiple_Complex
TC0900007752.hg.1	4.07	3.52	1.46	0.0066	0.0218	NTRK2	neurotrophic tyrosine kinase, receptor, type 2	Multiple_Complex
TC0100016167.hg.1	4.45	3.95	1.41	0.0066	0.0218	NR1I3	nuclear receptor subfamily 1, group I, membe	Multiple_Complex
TC1900011139.hg.1	4.71	4.34	1.29	0.0066	0.0218	SLC17A7	solute carrier family 17 (vesicular glutamate t	Multiple_Complex
TC0200016667.hg.1	8.08	8.58	-1.42	0.0066	0.0218	GPR75-ASB	GPR75-ASB3 readthrough	Multiple_Complex
TC1300008044.hg.1	8.16	8.73	-1.48	0.0066	0.0218	ARHGEF7	Rho guanine nucleotide exchange factor 7	Multiple_Complex
TC0100008621.hg.1	8.09	7.49	1.52	0.0067	0.0219	LEPR; LEPR	leptin receptor; leptin receptor overlapping tr	Multiple_Complex
TC0600011379.hg.1	5.01	5.57	-1.47	0.0067	0.0219	MDC1	mediator of DNA-damage checkpoint 1	Multiple_Complex
TC2200007114.hg.1	13.35	12.19	2.23	0.0067	0.0219	YWHAH	tyrosine 3-monooxygenase/tryptophan 5-mor	Multiple_Complex
TC1700008561.hg.1	4.01	3.53	1.39	0.0067	0.022	KCNH6	potassium channel, voltage gated eag related	Coding
TSUnmapped00000215.h	8.74	8.83	-1.06	0.0067	0.022	TCF20	transcription factor 20 (AR1)	Coding
TSUnmapped00000554.h	8.74	8.83	-1.06	0.0067	0.022	TCF20	transcription factor 20 (AR1)	Coding
TC2200009352.hg.1	8.01	8.65	-1.55	0.0067	0.022	LOC400927	TPTE and PTEN homologous inositol lipid phos	Multiple_Complex
TC0200011129.hg.1	5.63	4.95	1.61	0.0067	0.022	SAG	S-antigen; retina and pineal gland (arrestin)	Multiple_Complex
TC2200007790.hg.1	8.66	9.28	-1.53	0.0067	0.022	TRABD	TraB domain containing	Multiple_Complex
TC0X00007301.hg.1	4.58	3.98	1.51	0.0067	0.022	XAGE5	X antigen family, member 5	Multiple_Complex
TC1700012485.hg.1	5.87	5.41	1.37	0.0067	0.022	MYADML2	myeloid-associated differentiation marker-like	Coding
TC2100008555.hg.1	6.47	7.18	-1.64	0.0067	0.022	TIAM1	T-cell lymphoma invasion and metastasis 1	Multiple_Complex
TC1900006502.hg.1	4.64	4.95	-1.23	0.0067	0.0221	WDR18	WD repeat domain 18	Multiple_Complex
TC0200016661.hg.1	4.52	3.82	1.62	0.0067	0.0221	C2orf61	chromosome 2 open reading frame 61	Multiple_Complex
TC0600013201.hg.1	4.94	4.32	1.53	0.0067	0.0221	TAAR1	trace amine associated receptor 1	Coding
TC1200007786.hg.1	4.5	3.84	1.59	0.0067	0.0221	OR6C1	olfactory receptor, family 6, subfamily C, mem	Coding
TC1100010942.hg.1	4.95	4.25	1.62	0.0068	0.0222	OR5B2	olfactory receptor, family 5, subfamily B, mem	Coding
TSUnmapped00000339.h	6.3	5.78	1.44	0.0068	0.0222	SURF1	surfeit 1	Coding
TC0100008417.hg.1	5.62	5.22	1.31	0.0068	0.0223	PCSK9	proprotein convertase subtilisin/kexin type 9	Multiple_Complex
TC0900012258.hg.1	9.93	10.28	-1.28	0.0068	0.0223	MFSD14C	major facilitator superfamily domain containir	Multiple_Complex
TC1000012586.hg.1	6.52	7.41	-1.85	0.0068	0.0223	SEC31B	SEC31 homolog B, COPII coat complex compo	Multiple_Complex
TC1100011637.hg.1	3.6	3.18	1.34	0.0068	0.0223	OR2AT4	olfactory receptor, family 2, subfamily AT, me	Coding
TC1700011059.hg.1	8.97	9.34	-1.29	0.0068	0.0223	SLC35B1	solute carrier family 35, member B1	Multiple_Complex
TC0800009878.hg.1	11.34	11.65	-1.24	0.0068	0.0223	ENTPD4; L(	ectonucleoside triphosphate diphosphohydrol	Multiple_Complex
TC1600007374.hg.1	7.01	7.42	-1.33	0.0068	0.0223	NFATC2IP;	nuclear factor of activated T-cells, cytoplasmi	Multiple_Complex
TC1700012190.hg.1	11.98	11.43	1.47	0.0068	0.0223	EIF4A1; SN	eukaryotic translation initiation factor 4A1; sn	Multiple_Complex
TC1100011656.hg.1	5.61	5.29	1.25	0.0068	0.0223	SERPINH1	Jeck2013 ANTISENSE, coding, INTERNAL, intro	NonCoding
TC0600007284.hg.1	12.83	11.33	2.84	0.0068	0.0223	HIST1H4E	histone cluster 1, H4e	Multiple_Complex
TC0Y00006573.hg.1	3.82	3.47	1.28	0.0068	0.0224	TSPY3; LOC	Homo sapiens testis specific protein, Y-linked	Multiple_Complex
TC0400012891.hg.1	7.12	7.48	-1.29	0.0068	0.0224	NELFA; MIF	negative elongation factor complex member A	Multiple_Complex
TC0800011445.hg.1	4.86	4.15	1.64	0.0068	0.0224	ANGPT1	angiopoietin 1	Multiple_Complex
TC0200016559.hg.1	4.12	4.56	-1.36	0.0068	0.0224	BBS5	Bardet-Biedl syndrome 5	Multiple_Complex
TC0X00009836.hg.1	5.5	6.24	-1.68	0.0069	0.0224	SPIN3	spindlin family, member 3	Multiple_Complex
TC1600007514.hg.1	6.63	7.29	-1.59	0.0069	0.0224	KAT8	K(lysine) acetyltransferase 8	Multiple_Complex
TC0300006873.hg.1	3.34	3	1.27	0.0069	0.0224	LRRC3B	leucine rich repeat containing 3B	Multiple_Complex
TC0200011105.hg.1	4.61	4.31	1.23	0.0069	0.0225	CHRNG	cholinergic receptor, nicotinic gamma	Multiple_Complex
TC1900011789.hg.1	8.87	9.41	-1.46	0.0069	0.0226	EMC10	ER membrane protein complex subunit 10	Multiple_Complex
TC1900011392.hg.1	6.08	6.42	-1.27	0.0069	0.0226	TFPT	TCF3 (E2A) fusion partner (in childhood Leuke	Coding
TSUnmapped00000349.h	7.53	8.01	-1.4	0.0069	0.0226	FBL	fibrillarin	Coding
TC0X00008246.hg.1	5.64	6.25	-1.52	0.0069	0.0226	SLC25A43	solute carrier family 25, member 43	Multiple_Complex
TC1200009085.hg.1	4.38	4.01	1.29	0.0069	0.0226	SRRM4	serine/arginine repetitive matrix 4	Multiple_Complex
TC0100016402.hg.1	3.57	3.35	1.16	0.0069	0.0227	MYOC	myocilin, trabecular meshwork inducible gluco	Coding
TC0100013650.hg.1	4.54	4.18	1.28	0.0069	0.0227	A3GALT2	alpha 1,3-galactosyltransferase 2	Coding
TC0100016755.hg.1	4.05	3.65	1.33	0.007	0.0227	BRINP3	bone morphogenetic protein/retinoic acid ind	Multiple_Complex
TC2000006551.hg.1	7.79	8.1	-1.23	0.007	0.0228	ATRN	attractin	Multiple_Complex
TC1900011745.hg.1	6.51	7.27	-1.69	0.007	0.0228	ARHGEF1	Rho guanine nucleotide exchange factor 1	Multiple_Complex
TC0700008337.hg.1	5.98	6.41	-1.35	0.007	0.0228	VPS50	VPS50 EARP/GARPII complex subunit	Multiple_Complex
TC0500009097.hg.1	4.68	5.09	-1.33	0.007	0.0228	SYNPO	synaptopodin	Multiple_Complex
TC1900008623.hg.1	9.93	10.91	-1.97	0.007	0.0228	SIGLEC9	sialic acid binding Ig-like lectin 9	Coding
TC0400008666.hg.1	7.79	8.31	-1.44	0.007	0.0228	ABHD18	abhydrolase domain containing 18	Multiple_Complex
TC0600012827.hg.1	5.06	4.31	1.69	0.007	0.0229	WASF1	WAS protein family, member 1	Coding
TC0X00011378.hg.1	4	3.48	1.43	0.007	0.0229	RAB40A	RAB40A, member RAS oncogene family	Coding
TC1600007112.hg.1	6.9	6.53	1.3	0.0071	0.023	CCP110	centriolar coiled coil protein 110kDa	Multiple_Complex
TC0100018199.hg.1	11.87	12.81	-1.91	0.0071	0.023	SRRM1	serine/arginine repetitive matrix 1	Multiple_Complex
TC0500013297.hg.1	6.47	7.01	-1.46	0.0071	0.023	AMACR	alpha-methylacyl-CoA racemase	Multiple_Complex

TC0100015434.hg.1	9.63	10.07	-1.36	0.0071	0.023	GDAP2	ganglioside induced differentiation associated	Multiple_Complex
TC1700007017.hg.1	10.06	9.39	1.6	0.0071	0.023	SNORD49A	small nucleolar RNA, C/D box 49A; small nucle	Multiple_Complex
TC0500013426.hg.1	8.44	9.09	-1.57	0.0071	0.0231	TBC1D9B	TBC1 domain family, member 9B (with GRAM	Multiple_Complex
TC0200009041.hg.1	8.81	9.16	-1.28	0.0071	0.0231	DDX18	DEAD (Asp-Glu-Ala-Asp) box polypeptide 18	Multiple_Complex
TC0200013232.hg.1	7.06	6.51	1.47	0.0071	0.0231	SUCLG1	succinate-CoA ligase, alpha subunit	Multiple_Complex
TC0200013019.hg.1	4.6	4.81	-1.16	0.0071	0.0232	MCEE	methylmalonyl CoA epimerase	Multiple_Complex
TC0X00009271.hg.1	5.51	5.12	1.31	0.0071	0.0232	ARX	aristaless related homeobox	Coding
TC1200008942.hg.1	7	7.63	-1.54	0.0071	0.0232	PLBD2	phospholipase B domain containing 2	Multiple_Complex
TC0800009831.hg.1	4.57	4.23	1.27	0.0071	0.0233	LGI3	leucine-rich repeat LGI family, member 3	Multiple_Complex
TC0900010837.hg.1	8.8	9.38	-1.49	0.0072	0.0233	FAM120AC	family with sequence similarity 120A opposite	Multiple_Complex
TC0800011679.hg.1	6.94	6.39	1.47	0.0072	0.0233	ATAD2	ATPase family, AAA domain containing 2	Multiple_Complex
TC1000012090.hg.1	10.39	9.87	1.43	0.0072	0.0234	IKZF5	IKAROS family zinc finger 5	Multiple_Complex
TC0300013255.hg.1	6.73	7.36	-1.54	0.0072	0.0234	ZMAT3	zinc finger, matrin-type 3	Multiple_Complex
TC1600011562.hg.1	11.54	12	-1.37	0.0072	0.0234	ZNF19	zinc finger protein 19	Multiple_Complex
TC0100018394.hg.1	7.9	7.48	1.34	0.0072	0.0235	TTC34	tetratricopeptide repeat domain 34	Multiple_Complex
TC0500013292.hg.1	6.2	5.72	1.39	0.0072	0.0235	11-Mar	membrane associated ring finger 11	Coding
TC0100012456.hg.1	5.74	5.32	1.34	0.0072	0.0236	FAM132A	family with sequence similarity 132, member	Multiple_Complex
TC1600011257.hg.1	4.32	3.95	1.29	0.0072	0.0236	SLC22A31	solute carrier family 22, member 31	Multiple_Complex
TC1500010360.hg.1	3.51	4.1	-1.5	0.0072	0.0236	DET1	de-etiolated homolog 1 (Arabidopsis)	NonCoding
TC1000007748.hg.1	4.69	4.24	1.37	0.0073	0.0236	CDK1	cyclin-dependent kinase 1	Multiple_Complex
TC1200010908.hg.1	8.41	9.8	-2.63	0.0073	0.0236	STAT2	signal transducer and activator of transcriptio	Multiple_Complex
TC2100008392.hg.1	13.93	12.88	2.07	0.0073	0.0236	ITGB2	integrin, beta 2 (complement component 3 re	Multiple_Complex
TC0800012363.hg.1	4.41	3.95	1.38	0.0073	0.0236	KHDRBS3	KH domain containing, RNA binding, signal tra	NonCoding
TC1700012484.hg.1	4.85	5.17	-1.25	0.0073	0.0237	PYCR1	pyrroline-5-carboxylate reductase 1	Multiple_Complex
TSUnmapped00000284.h	4.25	3.64	1.52	0.0073	0.0237	LRP6	LDL receptor related protein 6	Coding
TC2200007035.hg.1	8.58	7.66	1.9	0.0073	0.0238	UQCR10	ubiquinol-cytochrome c reductase, complex II	Multiple_Complex
TC0200010729.hg.1	9.54	9	1.46	0.0073	0.0238	XRCC5	Transcript Identified by AceView, Entrez Gene	Unassigned
TC1000008716.hg.1	9.07	9.44	-1.3	0.0073	0.0238	GBF1	golgi brefeldin A resistant guanine nucleotide	Multiple_Complex
TC1800006484.hg.1	5.65	4.85	1.75	0.0073	0.0238	NDC80	NDC80 kinetochore complex component	Multiple_Complex
TC0100016618.hg.1	4.7	4.3	1.32	0.0073	0.0238	ZNF648	zinc finger protein 648	Coding
TC0500008349.hg.1	7.78	8.32	-1.46	0.0073	0.0238	YTHDC2	YTH domain containing 2	Multiple_Complex
TC0900007098.hg.1	6.77	7.2	-1.34	0.0073	0.0238	RGP1	RGP1 homolog, RAB6A GEF complex partner 1	Multiple_Complex
TC1500010900.hg.1	7.46	6.64	1.76	0.0074	0.0239	MTHFS	5,10-methenyltetrahydrofolate synthetase (5-	Multiple_Complex
TC1200008318.hg.1	6.96	7.4	-1.36	0.0074	0.024	METTL25	methyltransferase like 25	Multiple_Complex
TC1100013045.hg.1	7.5	6.9	1.52	0.0074	0.024	SIPA1	signal-induced proliferation-associated 1	Multiple_Complex
TC1600008174.hg.1	5.17	5.37	-1.15	0.0074	0.024	THAP11	THAP domain containing 11	Multiple_Complex
TC0300013104.hg.1	6	6.34	-1.27	0.0074	0.024	EIF5A2	eukaryotic translation initiation factor 5A2	Multiple_Complex
TC2100008550.hg.1	5.02	5.49	-1.39	0.0074	0.024	KRTAP19-1	keratin associated protein 19-1	Coding
TC1800008427.hg.1	4.39	4.74	-1.28	0.0074	0.024	KLHL14	kelch-like family member 14	Coding
TC0700008265.hg.1	4.83	5.39	-1.48	0.0074	0.0241	ADAM22	ADAM metallopeptidase domain 22	Multiple_Complex
TC0100007489.hg.1	6.15	6.66	-1.43	0.0074	0.0241	NUDC	nudC nuclear distribution protein	Multiple_Complex
TC1600011184.hg.1	6.84	5.94	1.88	0.0074	0.0241	KLHDC4	Transcript Identified by AceView, Entrez Gene	Unassigned
TC2100007846.hg.1	4.11	4.44	-1.25	0.0074	0.0241	N6AMT1	N-6 adenine-specific DNA methyltransferase 1	Multiple_Complex
TC0X00009654.hg.1	5.14	5.66	-1.43	0.0074	0.0241	GPKOW	G-patch domain and KOW motifs	Multiple_Complex
TC1100009969.hg.1	4.92	5.6	-1.6	0.0074	0.0241	APBB1	amyloid beta (A4) precursor protein-binding, f	Multiple_Complex
TC1700010816.hg.1	6.65	5.87	1.71	0.0074	0.0241	ATXN7L3	ataxin 7-like 3	Multiple_Complex
TC0600014196.hg.1	8.57	7.81	1.69	0.0074	0.0241	SNX9	sorting nexin 9	Multiple_Complex
TC1900009871.hg.1	7.47	7.87	-1.32	0.0074	0.0241	WIZ	widely interspaced zinc finger motifs	Multiple_Complex
TC0Y00006873.hg.1	6.97	6.57	1.32	0.0075	0.0241	CRLF2	cytokine receptor-like factor 2	Multiple_Complex
TC0100010855.hg.1	10.97	10.17	1.74	0.0075	0.0241	NPL	N-acetylneuraminate pyruvate lyase (dihydro	Multiple_Complex
TC1700007296.hg.1	14.11	13.81	1.24	0.0075	0.0241	MTRNR2L1	MT-RNR2-like 1	Coding
TC0500012491.hg.1	5.56	5.97	-1.33	0.0075	0.0242	ZNF300	zinc finger protein 300	Coding
TC1600008402.hg.1	10.17	9.52	1.57	0.0075	0.0242	PSMD7	proteasome 26S subunit, non-ATPase 7	Multiple_Complex
TC0900008667.hg.1	3.94	3.55	1.31	0.0075	0.0242	OR1N2	olfactory receptor, family 1, subfamily N, men	Coding
TSUnmapped00000594.h	5.37	5.02	1.28	0.0075	0.0242	GREM1	gremlin 1, DAN family BMP antagonist [Source	Coding
TC0200016302.hg.1	5.9	5.17	1.65	0.0075	0.0242	PRR21	proline rich 21	Coding
TC1000008328.hg.1	7.61	8.33	-1.64	0.0075	0.0243	FAM35A	family with sequence similarity 35, member A	Multiple_Complex
TC1000009287.hg.1	4.34	3.9	1.35	0.0075	0.0243	FOXI2	forkhead box I2	Coding
TC2000008210.hg.1	6.09	5.83	1.2	0.0075	0.0243	AVP	arginine vasopressin	Multiple_Complex
TC1200011057.hg.1	7.14	6.32	1.76	0.0075	0.0243	RPL14	Transcript Identified by AceView, Entrez Gene	Unassigned
TC1200011108.hg.1	5.41	5.01	1.32	0.0075	0.0243	WIF1	WNT inhibitory factor 1	Coding
TC1600011050.hg.1	3.72	3.97	-1.19	0.0075	0.0243	KCNG4	potassium channel, voltage gated modifier sul	Coding
TC0400010171.hg.1	4.84	5.36	-1.43	0.0075	0.0243	LCORL	Transcript Identified by AceView, Entrez Gene	Coding
TC0700012078.hg.1	6.57	5.96	1.53	0.0075	0.0243	SH2B2	Memczak2013 ANTISENSE, CDS, coding, INTEF	NonCoding
TC1600009220.hg.1	7.1	7.49	-1.31	0.0075	0.0243	TFAP4	transcription factor AP-4 (activating enhancer	Multiple_Complex
TSUnmapped00000446.h	9.14	9.65	-1.43	0.0075	0.0244	INPP5D	inositol polyphosphate-5-phosphatase D	Coding
TC1100012969.hg.1	3.91	3.62	1.22	0.0075	0.0244	CALCB	calcitonin-related polypeptide beta	Multiple_Complex
TC1700011262.hg.1	5.42	4.86	1.47	0.0075	0.0244	C17orf47	chromosome 17 open reading frame 47	Coding
TC0600011534.hg.1	6.12	6.65	-1.44	0.0075	0.0244	WDR46; M	WD repeat domain 46; microRNA 6873	Multiple_Complex
TC0500007725.hg.1	6.72	7.55	-1.78	0.0076	0.0244	BDP1	B double prime 1, subunit of RNA polymerase	Multiple_Complex

TC1900006480.hg.1	5.36	5	1.28	0.0076	0.0244	PALM	paralemmin	Multiple_Complex
TC1600010869.hg.1	3.24	3.03	1.16	0.0076	0.0244	BCAR1	breast cancer anti-estrogen resistance 1	Multiple_Complex
TC0500007123.hg.1	7.22	6.59	1.55	0.0076	0.0244	DNAJC21	DnaJ (Hsp40) homolog, subfamily C, member	Multiple_Complex
TC0800009529.hg.1	8.79	7.84	1.93	0.0076	0.0245	PPP1R3B	protein phosphatase 1, regulatory subunit 3B	Coding
TC2000007016.hg.1	5.66	4.96	1.61	0.0076	0.0245	GINS1	GINS complex subunit 1 (Psf1 homolog)	Multiple_Complex
TC1700012415.hg.1	5.89	6.62	-1.66	0.0076	0.0245	KAT2A	K(lysine) acetyltransferase 2A	Multiple_Complex
TC1500010880.hg.1	5.36	4.82	1.45	0.0076	0.0245	TIPIN	TIMELESS interacting protein	Multiple_Complex
TC0200012159.hg.1	3.99	3.47	1.43	0.0076	0.0245	CAPN13	calpain 13	Multiple_Complex
TC0400012452.hg.1	7.59	8.24	-1.57	0.0076	0.0245	FBXO8	F-box protein 8	Coding
TC1100006995.hg.1	4.66	4.36	1.23	0.0076	0.0245	SAA1	serum amyloid A1	Multiple_Complex
TC1100006547.hg.1	4.31	4.5	-1.14	0.0076	0.0246	MRPL23	mitochondrial ribosomal protein L23	Multiple_Complex
TC0200015887.hg.1	10.66	10.06	1.52	0.0076	0.0246	CUL3	cullin 3	Multiple_Complex
TC1300008219.hg.1	3.87	3.24	1.55	0.0076	0.0246	TPTE2	transmembrane phosphoinositide 3-phosphat	Multiple_Complex
TC0100011999.hg.1	5.3	5.92	-1.55	0.0077	0.0247	NTPCR	nucleoside-triphosphatase, cancer-related	Multiple_Complex
TC0100008289.hg.1	10.89	10.22	1.59	0.0077	0.0247	OSBPL9	oxysterol binding protein-like 9	Multiple_Complex
TC0400010682.hg.1	5.09	5.57	-1.39	0.0077	0.0247	SGCB	sarcoglycan beta	Coding
TC0400008455.hg.1	5.89	5.46	1.36	0.0077	0.0247	ANK2	Transcript Identified by AceView, Entrez Gene	Unassigned
TC0500009620.hg.1	8.84	9.49	-1.57	0.0077	0.0247	RGS14	regulator of G-protein signaling 14	Multiple_Complex
TC0100018306.hg.1	3.47	3.2	1.21	0.0077	0.0247	CADM3	cell adhesion molecule 3	Coding
TC0500009599.hg.1	5.41	5.87	-1.38	0.0077	0.0248	TSPAN17	tetraspanin 17	Multiple_Complex
TC0500009787.hg.1	5.17	5.74	-1.48	0.0077	0.0248	CCDC127	coiled-coil domain containing 127	Multiple_Complex
TC0700011510.hg.1	6.06	5.58	1.39	0.0077	0.0248	RFC2	replication factor C subunit 2	Multiple_Complex
TC0X00007951.hg.1	6.08	5.68	1.32	0.0077	0.0249	ARMCX3	armadillo repeat containing, X-linked 3	Multiple_Complex
TC0600012011.hg.1	5	4.55	1.37	0.0077	0.0249	DEFB110	defensin, beta 110	Coding
TC1500008225.hg.1	4.93	5.47	-1.46	0.0077	0.0249	MRPS11	mitochondrial ribosomal protein S11	Multiple_Complex
TC1200007356.hg.1	3.82	3.5	1.25	0.0077	0.0249	MUC19	mucin 19, oligomeric	Multiple_Complex
TC0700008918.hg.1	4.7	4.2	1.42	0.0078	0.0249	KCND2	potassium channel, voltage gated Shal related	Multiple_Complex
TC2100007373.hg.1	3.88	3.62	1.2	0.0078	0.0249	KRTAP10-4	keratin associated protein 10-4	Multiple_Complex
TC0100013949.hg.1	7.3	7.82	-1.43	0.0078	0.025	SLC6A9	solute carrier family 6 (neurotransmitter trans	Multiple_Complex
TC0900012080.hg.1	7.61	8.41	-1.75	0.0078	0.025	ARRDC1-AS	ARRDC1 antisense RNA 1	Multiple_Complex
TC0600008078.hg.1	5.41	5.11	1.23	0.0078	0.0251	SLC22A7	solute carrier family 22 (organic anion transpo	Multiple_Complex
TC1400008014.hg.1	8.75	9.53	-1.73	0.0078	0.0251	RIN3	Ras and Rab interactor 3	Multiple_Complex
TC1000012604.hg.1	6.66	7.24	-1.5	0.0078	0.0251	METTL10	methyltransferase like 10	Multiple_Complex
TC1400006652.hg.1	5.72	5.13	1.51	0.0078	0.0251	ABHD4	abhydrolase domain containing 4	Multiple_Complex
TC0X00008136.hg.1	8.03	8.51	-1.4	0.0078	0.0251	ALG13	ALG13, UDP-N-acetylglucosaminyltransferase	Multiple_Complex
TC1100011404.hg.1	5.76	5.28	1.4	0.0078	0.0251	MRPL21	mitochondrial ribosomal protein L21	Multiple_Complex
TC0800008267.hg.1	8.7	9.19	-1.41	0.0078	0.0251	INTS8	integrator complex subunit 8	Multiple_Complex
TC1200012680.hg.1	5.16	4.73	1.35	0.0078	0.0252	ACSS3	acyl-CoA synthetase short-chain family memb	Multiple_Complex
TC0200006788.hg.1	6.49	7.21	-1.64	0.0078	0.0252	DDX1	DEAD (Asp-Glu-Ala-Asp) box helicase 1	Multiple_Complex
TC1100006492.hg.1	6.18	6.83	-1.56	0.0078	0.0252	PNPLA2	patatin-like phospholipase domain containing	Multiple_Complex
TC2000009579.hg.1	4.02	3.59	1.34	0.0079	0.0252	BMP7	bone morphogenetic protein 7	Multiple_Complex
TC1100010844.hg.1	4.45	3.96	1.4	0.0079	0.0252	OR8J3	olfactory receptor, family 8, subfamily J, mem	Coding
TC0100010039.hg.1	4.56	4.06	1.42	0.0079	0.0252	LOC101928	uncharacterized LOC101928034; Salzman2013	NonCoding
TC0X00009727.hg.1	4.89	4.32	1.49	0.0079	0.0252	SPANXN5	SPANX family, member N5	Coding
TC1600008973.hg.1	4.64	4.97	-1.25	0.0079	0.0252	FBXL16	F-box and leucine-rich repeat protein 16	Multiple_Complex
TC0400008004.hg.1	5.52	4.8	1.64	0.0079	0.0252	MRPS18C	mitochondrial ribosomal protein S18C	Multiple_Complex
TC0100014608.hg.1	5.07	4.63	1.36	0.0079	0.0253	LRRC53	leucine rich repeat containing 53	Coding
TC1400008780.hg.1	4.94	4.57	1.29	0.0079	0.0253	NOVA1	neuro-oncological ventral antigen 1	Multiple_Complex
TC1200009843.hg.1	11.57	12.02	-1.37	0.0079	0.0253	M6PR	mannose-6-phosphate receptor (cation deper	Multiple_Complex
TC0400007991.hg.1	6.56	5.69	1.84	0.0079	0.0253	COPS4	COP9 signalosome subunit 4	Multiple_Complex
TC0300008539.hg.1	6.22	6.78	-1.48	0.0079	0.0253	SLC15A2	solute carrier family 15 (oligopeptide transpor	Multiple_Complex
TC0100006912.hg.1	4.75	4.3	1.36	0.0079	0.0254	PRAMEF7;	PRAME family member 7; PRAME family mem	Coding
TC1700007702.hg.1	5.94	6.51	-1.49	0.0079	0.0254	SOCS7	suppressor of cytokine signaling 7	Multiple_Complex
TC0700009233.hg.1	4.87	6.03	-2.24	0.0079	0.0254	BPGM	Transcript Identified by AceView, Entrez Gene	Unassigned
TC0200016717.hg.1	6.46	6.19	1.21	0.0079	0.0254	TBC1D8	TBC1 domain family, member 8 (with GRAM d	Multiple_Complex
TC0300013151.hg.1	6.45	7.12	-1.58	0.0079	0.0254	NCEH1	neutral cholesterol ester hydrolase 1	Multiple_Complex
TC0300009258.hg.1	11.31	9.02	4.87	0.008	0.0255	MME	membrane metallo-endopeptidase	Multiple_Complex
TC0100011920.hg.1	6.48	5.95	1.44	0.008	0.0255	GALNT2	polypeptide N-acetylgalactosaminyltransferas	Multiple_Complex
TC0300012118.hg.1	8.87	9.37	-1.41	0.008	0.0255	TMEM39A	transmembrane protein 39A	Multiple_Complex
TC0700013629.hg.1	9.98	10.14	-1.12	0.008	0.0255	BRAF	B-Raf proto-oncogene, serine/threonine kinas	Multiple_Complex
TC0100008336.hg.1	11.13	10.42	1.64	0.008	0.0255	SCP2	sterol carrier protein 2	Multiple_Complex
TC0200013870.hg.1	5.18	4.79	1.31	0.008	0.0255	LIMS3L	LIM and senescent cell antigen-like domains 3	Multiple_Complex
TC1900010789.hg.1	7.06	7.63	-1.49	0.008	0.0255	POU2F2	POU class 2 homeobox 2	Multiple_Complex
TC0300013945.hg.1	4.79	5.54	-1.68	0.008	0.0255	METTL6	methyltransferase like 6	Multiple_Complex
TC1900012031.hg.1	7.25	7.64	-1.31	0.008	0.0255	ZNF816	zinc finger protein 816	Coding
TC1100012350.hg.1	3.6	3.24	1.28	0.008	0.0255	DRD2	dopamine receptor D2	Multiple_Complex
TC0300009702.hg.1	8.22	8.9	-1.6	0.008	0.0255	EIF4G1	eukaryotic translation initiation factor 4 gamn	Multiple_Complex
TC0500007251.hg.1	6.16	6.56	-1.31	0.008	0.0256	FBXO4	F-box protein 4	Multiple_Complex
TC0100011958.hg.1	7.59	8.43	-1.8	0.008	0.0256	GNPAT	glyceronephosphate O-acyltransferase	Multiple_Complex
TC0100016122.hg.1	6.16	6.68	-1.43	0.008	0.0256	IGSF8	immunoglobulin superfamily, member 8	Multiple_Complex

TC0300011712.hg.1	4.63	4.15	1.39	0.008	0.0256	POU1F1	POU class 1 homeobox 1	Multiple_Complex
TC1700010657.hg.1	4.14	3.67	1.38	0.008	0.0256	KRTAP4-12	keratin associated protein 4-12	Coding
TSUnmapped00000041.h	5.15	5.57	-1.34	0.008	0.0256	ATG16L1	autophagy related 16-like 1	NonCoding
TC1600011318.hg.1	5.96	5.49	1.39	0.008	0.0256	DECR2	2,4-dienoyl-CoA reductase 2, peroxisomal	Multiple_Complex
TC1600011060.hg.1	15.4	15.81	-1.32	0.008	0.0257	COTL1	coactosin-like F-actin binding protein 1	Multiple_Complex
TC0600009002.hg.1	5.83	6.37	-1.45	0.008	0.0257	C6orf203	chromosome 6 open reading frame 203	Multiple_Complex
TC1000011114.hg.1	4.25	3.89	1.29	0.0081	0.0258	KCNMA1	potassium channel, calcium activated large co	Multiple_Complex
TC1200008295.hg.1	4.46	3.93	1.44	0.0081	0.0258	OTOGL	otogelin-like	Multiple_Complex
TC1700006533.hg.1	6.11	6.54	-1.35	0.0081	0.0258	DPH1; OVC	diphthamide biosynthesis 1; ovarian tumor su	Multiple_Complex
TC1600008173.hg.1	3.91	3.57	1.27	0.0081	0.0258	TSNAXIP1	translin-associated factor X interacting proteir	Multiple_Complex
TC1400006484.hg.1	4.9	4.25	1.57	0.0081	0.0259	OR4Q3	olfactory receptor, family 4, subfamily Q, men	Coding
TC0100016789.hg.1	5.33	5.95	-1.53	0.0081	0.0259	GLRX2	glutaredoxin 2	Multiple_Complex
TC0600012551.hg.1	6.56	7.14	-1.5	0.0081	0.0259	MDN1	midasin AAA ATPase 1	Multiple_Complex
TC1500009460.hg.1	7.88	8.27	-1.31	0.0081	0.0259	FAM214A	family with sequence similarity 214, member .	Multiple_Complex
TC0900007472.hg.1	3.85	4.26	-1.33	0.0081	0.0259	ANKRD20A	ankyrin repeat domain 20 family, member A1	Multiple_Complex
TC1000011376.hg.1	4.5	3.98	1.44	0.0081	0.0259	CH25H	cholesterol 25-hydroxylase	Coding
TC0200016720.hg.1	14.57	13.53	2.06	0.0081	0.0259	RNF149	ring finger protein 149	Multiple_Complex
TC1700010582.hg.1	5.49	5.74	-1.19	0.0081	0.0259	PGAP3	post-GPI attachment to proteins 3	Multiple_Complex
TC1300008705.hg.1	8.15	7.24	1.87	0.0081	0.0259	ELF1	Transcript Identified by AceView, Entrez Gene	Unassigned
TC0100006577.hg.1	7.65	8	-1.28	0.0081	0.0259	RER1	retention in endoplasmic reticulum sorting rec	Multiple_Complex
TC0300009892.hg.1	4.27	3.84	1.35	0.0081	0.0259	ATP13A5-A	ATP13A5 antisense RNA 1	NonCoding
TC1500007303.hg.1	8.37	8.93	-1.48	0.0081	0.0259	PIGB	phosphatidylinositol glycan anchor biosynthes	Multiple_Complex
TC0100010066.hg.1	9.89	10.51	-1.54	0.0081	0.0259	UBAP2L	ubiquitin associated protein 2 like	Multiple_Complex
TC0900011072.hg.1	10.58	9.29	2.44	0.0081	0.0259	ABCA1	ATP binding cassette subfamily A member 1	Multiple_Complex
TC2200009290.hg.1	5.47	4.82	1.56	0.0082	0.026	FAM19A5	family with sequence similarity 19 (chemokine	Multiple_Complex
TC1800007440.hg.1	5.45	6.29	-1.79	0.0082	0.026	ZNF532	zinc finger protein 532	Multiple_Complex
TC1700012433.hg.1	4.67	4.39	1.21	0.0082	0.026	HOXB6	homeobox B6	Multiple_Complex
TC2100007342.hg.1	7.06	7.69	-1.54	0.0082	0.026	C21orf33	chromosome 21 open reading frame 33	Multiple_Complex
TC1200008544.hg.1	6.46	7.23	-1.7	0.0082	0.026	NEDD1	neural precursor cell expressed, development	Multiple_Complex
TC2200008831.hg.1	4.33	4.12	1.15	0.0082	0.026	PMM1	phosphomannomutase 1	Multiple_Complex
TC0600009082.hg.1	4.76	4.13	1.55	0.0082	0.026	C6orf183	chromosome 6 open reading frame 183	Multiple_Complex
TC1200008924.hg.1	4.09	4.53	-1.36	0.0082	0.026	DTX1	deltex 1, E3 ubiquitin ligase	Multiple_Complex
TC0100011282.hg.1	7.67	8.1	-1.35	0.0082	0.026	SOX13	SRY box 13	Multiple_Complex
TC1600011508.hg.1	7.93	7.39	1.45	0.0082	0.026	CDR2	cerebellar degeneration related protein 2	Multiple_Complex
TC0500013298.hg.1	4.26	4.96	-1.62	0.0082	0.0261	C1QTNF3	C1q and tumor necrosis factor related protein	Multiple_Complex
TC0200008801.hg.1	4.96	4.55	1.33	0.0082	0.0261	SULT1C4	sulfotransferase family 1C member 4	Multiple_Complex
TC0600007687.hg.1	5.4	6.06	-1.58	0.0082	0.0261	RING1	ring finger protein 1	Multiple_Complex
TC1900008245.hg.1	4.68	5.47	-1.73	0.0082	0.0261	ZNF221	zinc finger protein 221	Multiple_Complex
TC1400007440.hg.1	7.19	7.89	-1.62	0.0082	0.0261	MTHFD1	methylenetetrahydrofolate dehydrogenase (N	Multiple_Complex
TC1000006975.hg.1	5.65	6.31	-1.57	0.0082	0.0261	MLLT10	myeloid/lymphoid or mixed-lineage leukemia;	Multiple_Complex
TC0900010063.hg.1	7.38	6.38	2	0.0082	0.0261	CNTNAP3	contactin associated protein-like 3	Multiple_Complex
TC0500007667.hg.1	6.55	6.08	1.39	0.0082	0.0261	MRPS36	mitochondrial ribosomal protein S36	Multiple_Complex
TC0500009856.hg.1	7.78	8.19	-1.33	0.0082	0.0262	CLPTM1L	CLPTM1-like	Multiple_Complex
TC0200010516.hg.1	6.06	6.7	-1.56	0.0082	0.0262	CYP20A1	cytochrome P450, family 20, subfamily A, poly	Multiple_Complex
TC1900011495.hg.1	4.9	4.49	1.33	0.0083	0.0262	NLRP11	NLR family, pyrin domain containing 11	Coding
TC1800008301.hg.1	7.55	7.28	1.21	0.0083	0.0262	OSBPL1A	oxysterol binding protein-like 1A	Multiple_Complex
TC1200006647.hg.1	5.33	4.98	1.27	0.0083	0.0262	GNB3	guanine nucleotide binding protein (G protein	Multiple_Complex
TC0900012272.hg.1	9.56	10.14	-1.5	0.0083	0.0262	RC3H2	ring finger and CCCH-type domains 2	Multiple_Complex
TC1100007614.hg.1	5.1	4.43	1.59	0.0083	0.0262	OR5D16	olfactory receptor, family 5, subfamily D, men	Coding
TC1700010910.hg.1	9.75	10.51	-1.69	0.0083	0.0262	KANSL1	KAT8 regulatory NSL complex subunit 1	Multiple_Complex
TC0500008114.hg.1	5.41	4.91	1.42	0.0083	0.0263	FAM81B	family with sequence similarity 81, member B	Multiple_Complex
TC0300010903.hg.1	8.2	7.65	1.46	0.0083	0.0263	KIAA1143	KIAA1143	Multiple_Complex
TC0100015483.hg.1	12.54	11.74	1.73	0.0083	0.0263	NOTCH2	notch 2	Multiple_Complex
TC1800007506.hg.1	4.85	4.52	1.26	0.0083	0.0263	TNFRSF11A	tumor necrosis factor receptor superfamily, m	Multiple_Complex
TC0500008642.hg.1	4.89	4.4	1.4	0.0083	0.0263	IL13	interleukin 13	Multiple_Complex
TC0300012346.hg.1	14.17	14.61	-1.36	0.0083	0.0263	RPN1	ribophorin I	Multiple_Complex
TC1700012320.hg.1	6.21	6.43	-1.16	0.0083	0.0263	RPH3AL	rabphilin 3A-like (without C2 domains)	Multiple_Complex
TC0700012228.hg.1	5.22	4.81	1.33	0.0083	0.0263	LAMB4	laminin, beta 4	Multiple_Complex
TC0600011600.hg.1	7.47	6.68	1.73	0.0083	0.0264	TAF11	TAF11 RNA polymerase II, TATA box binding p	Multiple_Complex
TC0100010621.hg.1	4.43	3.77	1.57	0.0083	0.0264	C1orf105	chromosome 1 open reading frame 105	Multiple_Complex
TC1900011305.hg.1	6.02	6.58	-1.48	0.0083	0.0264	ZNF83	zinc finger protein 83	Multiple_Complex
TC1400010646.hg.1	4.82	5.71	-1.86	0.0083	0.0264	TCL1B; TCL	T-cell leukemia/lymphoma 1B; T-cell leukemia	Multiple_Complex
TC0700010392.hg.1	5.4	4.89	1.42	0.0083	0.0264	FERD3L	Fer3-like bHLH transcription factor	Coding
TC1100011558.hg.1	4.33	3.82	1.42	0.0084	0.0264	PDE2A	phosphodiesterase 2A, cGMP-stimulated	Multiple_Complex
TC0200007536.hg.1	4.24	3.92	1.25	0.0084	0.0264	GTF2A1L; S	general transcription factor IIA 1-like; STON1-(	Multiple_Complex
TC1900006964.hg.1	3.72	3.35	1.3	0.0084	0.0264	RDH8	retinol dehydrogenase 8 (all-trans)	Multiple_Complex
TC0200014332.hg.1	4.18	3.88	1.23	0.0084	0.0264	C2orf27B	chromosome 2 open reading frame 27B	Multiple_Complex
TC0X00007999.hg.1	5.25	4.81	1.36	0.0084	0.0264	BEX4	brain expressed X-linked 4	Multiple_Complex
TC1300009954.hg.1	9.7	8.9	1.74	0.0084	0.0265	DNAJC15	DnaJ (Hsp40) homolog, subfamily C, member	Multiple_Complex
TC0300009686.hg.1	5.29	4.81	1.39	0.0084	0.0265	HTR3C	5-hydroxytryptamine (serotonin) receptor 3C,	Coding

TC0900007976.hg.1	6.7	7.28	-1.49	0.0084	0.0266	SUSD3	sushi domain containing 3	Coding
TC0600009240.hg.1	8.6	7.76	1.78	0.0084	0.0266	KPNA5	karyopherin alpha 5 (importin alpha 6)	Multiple_Complex
TC0Y00006909.hg.1	4.56	4.2	1.28	0.0084	0.0267	SRY	sex determining region Y	Coding
TC0600007307.hg.1	9.01	9.63	-1.54	0.0084	0.0267	BTN2A1	butyrophilin, subfamily 2, member A1	Multiple_Complex
TC1900011153.hg.1	6.27	6.98	-1.63	0.0085	0.0267	RRAS	related RAS viral (r-ras) oncogene homolog	Multiple_Complex
TC1500006562.hg.1	4.46	4.01	1.37	0.0085	0.0267	NPAP1	nuclear pore associated protein 1	Coding
TC0700010908.hg.1	4.42	4.52	-1.07	0.0085	0.0267	POLM; MIR	polymerase (DNA directed), mu; microRNA 68	Multiple_Complex
TC1900008012.hg.1	5.45	6.16	-1.64	0.0085	0.0267	CATSPERG	catsper channel auxiliary subunit gamma	Multiple_Complex
TC1600011058.hg.1	6.06	6.57	-1.42	0.0085	0.0267	TLDC1	TBC/LysM-associated domain containing 1	Multiple_Complex
TC1700012324.hg.1	7.05	7.66	-1.53	0.0085	0.0267	ABR	active BCR-related	Multiple_Complex
TC1900011723.hg.1	5.12	5.79	-1.59	0.0085	0.0268	ZNF793	zinc finger protein 793	Multiple_Complex
TC0500009490.hg.1	5.42	5.09	1.25	0.0085	0.0268	BNIP1	BCL2/adenovirus E1B 19kDa interacting prote	Coding
TSUnmapped00000823.h	5.91	5.6	1.23	0.0085	0.0268	DUX1	double homeobox 1	Coding
TC1700007971.hg.1	4.86	5.23	-1.29	0.0085	0.0268	FAM215A	family with sequence similarity 215, member	Multiple_Complex
TC0100006624.hg.1	4.85	5.13	-1.22	0.0085	0.0269	SMIM1	small integral membrane protein 1 (Vel blood	Multiple_Complex
TC1100009745.hg.1	9.92	9.45	1.38	0.0085	0.0269	KRTAP5-1	keratin associated protein 5-1	Coding
TC0800009418.hg.1	4.75	5.29	-1.45	0.0085	0.0269	ANGPT2	angiopoietin 2	Multiple_Complex
TC0900007318.hg.1	7.77	7.39	1.3	0.0085	0.0269	SPATA31A7	SPATA31 subfamily A, member 7; SPATA31 su	Multiple_Complex
TC0100015572.hg.1	8.81	9.34	-1.44	0.0086	0.027	SRGAP2B	SLIT-ROBO Rho GTPase activating protein 2B	Multiple_Complex
TC0900008763.hg.1	7.64	7.13	1.42	0.0086	0.027	PBX3	pre-B-cell leukemia homeobox 3	Multiple_Complex
TC0200016347.hg.1	12.25	12.78	-1.45	0.0086	0.027	HDLBP	high density lipoprotein binding protein	Multiple_Complex
TC0200011690.hg.1	11.43	12.02	-1.51	0.0086	0.0271	YWHAQ	tyrosine 3-monooxygenase/tryptophan 5-mor	Multiple_Complex
TSUnmapped00000276.h	3.59	3.92	-1.26	0.0086	0.0271	VPS11	VPS11, CORVET/HOPS core subunit [Source:H	Coding
TC0700013391.hg.1	6.91	7.39	-1.4	0.0086	0.0271	NSUN5P1	NOP2/Sun domain family, member 5 pseudog	Multiple_Complex
TC1200009065.hg.1	6.69	6.92	-1.17	0.0086	0.0271	RFC5	replication factor C subunit 5	Multiple_Complex
TC1400006828.hg.1	5.03	4.67	1.29	0.0086	0.0271	COCH	cochlin	Multiple_Complex
TC0600012752.hg.1	4.49	4.81	-1.25	0.0086	0.0271	PDSS2	prenyl (decaprenyl) diphosphate synthase, sul	Multiple_Complex
TC1400008406.hg.1	4.75	5.15	-1.32	0.0086	0.0271	TRMT61A	tRNA methyltransferase 61A	Multiple_Complex
TC0300011107.hg.1	7.39	6.77	1.54	0.0086	0.0272	ZMYND10	zinc finger, MYND-type containing 10	Multiple_Complex
TC0400009330.hg.1	7.55	6.81	1.67	0.0086	0.0272	SAP30	Sin3A associated protein 30kDa	Multiple_Complex
TC0100013896.hg.1	5.65	5.26	1.31	0.0086	0.0272	CLDN19	claudin 19	Coding
TC0X00009107.hg.1	5.23	5.67	-1.36	0.0086	0.0272	GEMIN8	gem nuclear organelle associated protein 8	Multiple_Complex
TC0600012764.hg.1	10.78	10.01	1.71	0.0087	0.0272	SEC63	SEC63 homolog, protein translocation regulat	Multiple_Complex
TC1900006700.hg.1	7.77	8.27	-1.42	0.0087	0.0272	HDGFRP2	hepatoma-derived growth factor-related prot	Multiple_Complex
TC0600012556.hg.1	6.58	7.36	-1.72	0.0087	0.0272	BACH2	Transcript Identified by AceView, Entrez Gene	Unassigned
TC0900010586.hg.1	5.6	6.21	-1.53	0.0087	0.0272	C9orf64	chromosome 9 open reading frame 64	Multiple_Complex
TC0200016606.hg.1	6.31	6.64	-1.26	0.0087	0.0273	SCLY	selenocysteine lyase	Multiple_Complex
TC0100007240.hg.1	5.65	6.05	-1.32	0.0087	0.0273	NBPF3	neuroblastoma breakpoint family, member 3	Multiple_Complex
TC1000010768.hg.1	5.2	4.69	1.43	0.0087	0.0273	EGR2	early growth response 2	Multiple_Complex
TC0100013057.hg.1	6.54	6.81	-1.2	0.0087	0.0273	FAM231A;	Homo sapiens family with sequence similarity	Coding
TC1700006784.hg.1	8.1	7.72	1.3	0.0087	0.0273	GUCY2D	guanylate cyclase 2D, membrane (retina-spec	Multiple_Complex
TC0900010907.hg.1	4.75	4.27	1.4	0.0087	0.0273	HSD17B3	hydroxysteroid (17-beta) dehydrogenase 3	Multiple_Complex
TC1600008220.hg.1	7.63	8.56	-1.89	0.0087	0.0273	UTP4	UTP4 small subunit (SSU) processome compo	Multiple_Complex
TSUnmapped00000294.h	4.67	4.33	1.26	0.0087	0.0273	VPS11	VPS11, CORVET/HOPS core subunit [Source:H	NonCoding
TC0700013468.hg.1	6.45	5.79	1.58	0.0087	0.0274	NDUFB2	NADH dehydrogenase (ubiquinone) 1 beta sul	Multiple_Complex
TC1100007884.hg.1	10.3	9.92	1.3	0.0087	0.0274	SLC3A2	solute carrier family 3 (amino acid transporter	Multiple_Complex
TSUnmapped00000358.h	4.7	5.23	-1.44	0.0087	0.0274	ZNF197	zinc finger protein 197	Coding
TC1100010552.hg.1	3.93	3.48	1.37	0.0087	0.0274	RAG2	recombination activating gene 2	Multiple_Complex
TC1000008539.hg.1	4.37	3.83	1.45	0.0087	0.0275	DNTT	DNA nucleotidylexotransferase	Multiple_Complex
TC1900012010.hg.1	5.43	5.08	1.27	0.0088	0.0275	SHANK1	SH3 and multiple ankyrin repeat domains 1	Multiple_Complex
TC0900009074.hg.1	6.53	6.94	-1.33	0.0088	0.0275	SURF2	surfeit 2	Multiple_Complex
TC0100016068.hg.1	6.03	5.69	1.26	0.0088	0.0275	OR10K2	olfactory receptor, family 10, subfamily K, me	Coding
TC0400012781.hg.1	6.42	6.78	-1.28	0.0088	0.0275	STIM2	stromal interaction molecule 2	NonCoding
TC1200012543.hg.1	6.69	7.12	-1.35	0.0088	0.0275	ANKLE2	ankyrin repeat and LEM domain containing 2	Multiple_Complex
TC0X00006977.hg.1	8.35	7.74	1.52	0.0088	0.0275	MID1IP1	MID1 interacting protein 1	Coding
TC0100009149.hg.1	8.5	7.7	1.74	0.0088	0.0275	PTBP2	Transcript Identified by AceView, Entrez Gene	Unassigned
TC0600008127.hg.1	6.21	6.88	-1.59	0.0088	0.0276	SLC29A1	solute carrier family 29 (equilibrative nucleosi	Multiple_Complex
TC0900009706.hg.1	5.83	5.39	1.36	0.0088	0.0276	IFNA13	interferon, alpha 13	Coding
TC0400011175.hg.1	4.75	4.3	1.37	0.0088	0.0276	TMEM150C	transmembrane protein 150C	Multiple_Complex
TC0300010388.hg.1	6.86	7.6	-1.66	0.0088	0.0276	MRPS25	mitochondrial ribosomal protein S25	Multiple_Complex
TC1400006821.hg.1	5.87	6.53	-1.58	0.0088	0.0276	G2E3	G2/M-phase specific E3 ubiquitin protein ligas	Multiple_Complex
TC1900008924.hg.1	7.59	8.07	-1.39	0.0088	0.0276	U2AF2	U2 small nuclear RNA auxiliary factor 2	Multiple_Complex
TC1300008827.hg.1	5.12	4.4	1.64	0.0088	0.0276	ERICH6B	glutamate rich 6B	Multiple_Complex
TC0800009113.hg.1	10.32	9.88	1.36	0.0088	0.0277	PTP4A3	protein tyrosine phosphatase type IVA, memb	Multiple_Complex
TC1700009430.hg.1	7.57	7.99	-1.34	0.0088	0.0277	TSR1	TSR1, 20S rRNA accumulation, homolog (S. ce	Multiple_Complex
TC1200011471.hg.1	6.76	7.28	-1.44	0.0088	0.0277	POC1B; PO	POC1 centriolar protein B; POC1B-GALNT4 rea	Multiple_Complex
TC0600009234.hg.1	4.97	4.42	1.46	0.0088	0.0277	FAM26E	family with sequence similarity 26, member E	Coding
TC0300009412.hg.1	4.85	4.48	1.29	0.0089	0.0278	SERPINI1	serpin peptidase inhibitor, clade I (neuroserpi	Multiple_Complex
TC0200013351.hg.1	8.86	7.76	2.14	0.0089	0.0278	RGPD1; RG	RANBP2-like and GRIP domain containing 1; R	Multiple_Complex
TC1400010049.hg.1	3.86	3.48	1.31	0.0089	0.0278	SERPINA12	serpin peptidase inhibitor, clade A (alpha-1 an	Coding

TC1000012086.hg.1	4.2	3.59	1.53	0.0089	0.0278	C10orf120	chromosome 10 open reading frame 120	Coding
TC1400006495.hg.1	4.22	3.87	1.27	0.0089	0.0278	OR4Q2	olfactory receptor, family 4, subfamily Q, men	Pseudogene
TC1100013201.hg.1	5.41	5.88	-1.38	0.0089	0.0279	ORAOV1	oral cancer overexpressed 1	NonCoding
TSUnmapped00000359.h	7.71	8.18	-1.39	0.0089	0.0279	SLC2A6	solute carrier family 2 (facilitated glucose tran	Coding
TC0X00008833.hg.1	7.38	6.88	1.41	0.0089	0.0279	FAM50A	family with sequence similarity 50, member A	Multiple_Complex
TC1100008524.hg.1	5.12	4.71	1.33	0.0089	0.0279	OMP	olfactory marker protein	Coding
TSUnmapped00000147.h	5.36	5.97	-1.52	0.0089	0.0279	ATG16L1	autophagy related 16-like 1	Coding
TC0100018166.hg.1	7.31	7.83	-1.44	0.0089	0.028	CLCN6	chloride channel, voltage-sensitive 6	Multiple_Complex
TC0200011287.hg.1	5.09	5.57	-1.4	0.0089	0.028	ASB1	ankyrin repeat and SOCS box containing 1	Multiple_Complex
TC1200010182.hg.1	5.47	6.16	-1.62	0.009	0.028	BHLHE41	basic helix-loop-helix family, member e41	Multiple_Complex
TC1100012020.hg.1	5	5.89	-1.86	0.009	0.028	SESN3	Transcript Identified by AceView, Entrez Gene	Unassigned
TC0100015332.hg.1	9.66	9.21	1.36	0.009	0.0281	RSBN1	round spermatid basic protein 1	Multiple_Complex
TC2000009253.hg.1	5.34	5.82	-1.39	0.009	0.0281	ZNF335	zinc finger protein 335	Multiple_Complex
TC1000007761.hg.1	10.49	11.42	-1.91	0.009	0.0282	ARID5B	AT rich interactive domain 5B (MRF1-like)	Multiple_Complex
TC1900007502.hg.1	6.06	6.97	-1.88	0.0091	0.0283	ZNF85	zinc finger protein 85	Multiple_Complex
TC0500012526.hg.1	3.21	2.98	1.17	0.0091	0.0283	GLRA1	glycine receptor alpha 1	Multiple_Complex
TC0600010991.hg.1	9.95	10.45	-1.42	0.0091	0.0283	MBOAT1	membrane bound O-acyltransferase domain c	Multiple_Complex
TC0100016052.hg.1	7.19	8.16	-1.96	0.0091	0.0283	FCRL1	Fc receptor-like 1	Multiple_Complex
TC0300012771.hg.1	4.94	4.41	1.44	0.0091	0.0283	TM4SF18	Transcript Identified by AceView, Entrez Gene	Unassigned
TC0200016751.hg.1	4.39	4.78	-1.31	0.0091	0.0283	LOC10013C	uncharacterized LOC100130691; Transcript Id	NonCoding
TC0700013060.hg.1	6.37	6.89	-1.43	0.0091	0.0284	TMUB1	transmembrane and ubiquitin-like domain cor	Multiple_Complex
TC0600007986.hg.1	4.46	4.88	-1.34	0.0091	0.0284	APOBEC2	apolipoprotein B mRNA editing enzyme, cataly	Coding
TC0100009656.hg.1	9.5	8.36	2.2	0.0091	0.0284	FCGR1B	Fc fragment of IgG, high affinity Ib, receptor ((	Multiple_Complex
TC1700008350.hg.1	5.17	5.84	-1.59	0.0091	0.0284	NOG	noggin	Coding
TC1700011905.hg.1	6.16	5.72	1.36	0.0091	0.0284	DNAH17	dynein, axonemal, heavy chain 17	Multiple_Complex
TC0400009791.hg.1	6.46	6.98	-1.44	0.0091	0.0284	NOP14	NOP14 nucleolar protein	Multiple_Complex
TC1900011113.hg.1	8.35	9.09	-1.67	0.0091	0.0285	GYS1	glycogen synthase 1 (muscle)	Multiple_Complex
TC0X00011245.hg.1	4.82	4.32	1.41	0.0091	0.0285	H2AFB3	H2A histone family, member B3	Coding
TC0400011616.hg.1	7.7	7.1	1.51	0.0092	0.0286	TIFA	TRAF-interacting protein with forkhead-associ	Coding
TC1100007868.hg.1	4.57	4.39	1.13	0.0092	0.0286	GNG3	guanine nucleotide binding protein (G protein	Multiple_Complex
TC0400007276.hg.1	9.82	8.84	1.97	0.0092	0.0286	KLHL5	kelch-like family member 5	Multiple_Complex
TSUnmapped00000127.h	4.74	4.02	1.65	0.0092	0.0287	KIF15	kinesin family member 15	Coding
TC0100013809.hg.1	7.57	7.98	-1.33	0.0092	0.0287	TRIT1	tRNA isopentenyltransferase 1	Multiple_Complex
TC1100010391.hg.1	3.78	3.43	1.28	0.0092	0.0287	MUC15	mucin 15, cell surface associated	Coding
TSUnmapped00000530.h	4.86	5.41	-1.46	0.0092	0.0287	ATG16L1	autophagy related 16-like 1	Coding
TC0200012035.hg.1	5.69	5.18	1.43	0.0092	0.0287	ADGRF3	adhesion G protein-coupled receptor F3	Multiple_Complex
TC1500010838.hg.1	5.02	5.29	-1.21	0.0092	0.0288	GOLGA8M	golgin A8 family, member M	Multiple_Complex
TC1300006979.hg.1	8.69	9.69	-2	0.0092	0.0288	RGCC	regulator of cell cycle	Multiple_Complex
TC0800011974.hg.1	6.21	6.88	-1.59	0.0093	0.0288	TRAPPC9	trafficking protein particle complex 9	Multiple_Complex
TC1000008744.hg.1	8.57	9.34	-1.71	0.0093	0.0288	TRIM8	tripartite motif containing 8	Multiple_Complex
TC1200011843.hg.1	7.03	7.51	-1.39	0.0093	0.0288	SART3	squamous cell carcinoma antigen recognized l	Multiple_Complex
TC0100014685.hg.1	4.86	4.33	1.45	0.0093	0.0289	ADGRL4	adhesion G protein-coupled receptor L4	Coding
TC2200007313.hg.1	8.98	9.87	-1.86	0.0093	0.0289	TRIOBP; NC	TRIO and F-actin binding protein; nucleolar pr	Multiple_Complex
TC0600011732.hg.1	6.24	6.68	-1.35	0.0093	0.0289	SAYSD1	SAYSVFN motif domain containing 1	Multiple_Complex
TC2000009624.hg.1	5.09	4.73	1.29	0.0093	0.0289	APCDD1L	adenomatosis polyposis coli down-regulated 1	Multiple_Complex
TC2200007909.hg.1	4.62	5.1	-1.4	0.0093	0.0289	MICAL3	microtubule associated monooxygenase, calp	Multiple_Complex
TC0200010410.hg.1	7.52	9.87	-5.1	0.0093	0.029	SPATS2L	spermatogenesis associated, serine-rich 2-like	Multiple_Complex
TC1400007308.hg.1	5.22	4.88	1.27	0.0093	0.029	TOMM20L	translocase of outer mitochondrial membrane	Coding
TC1400010756.hg.1	5.57	6.2	-1.54	0.0094	0.0291	GPR135	G protein-coupled receptor 135	Multiple_Complex
TC0700009410.hg.1	4.38	3.75	1.55	0.0094	0.0291	OR9A4	olfactory receptor, family 9, subfamily A, mem	Coding
TC0400008861.hg.1	5.18	5.94	-1.69	0.0094	0.0291	IL15	interleukin 15	Multiple_Complex
TC0500013197.hg.1	4.82	4.36	1.38	0.0094	0.0292	ATP6AP1L	ATPase, H+ transporting, lysosomal accessory	NonCoding
TC0300014093.hg.1	9.94	9.36	1.5	0.0094	0.0293	PCYT1A	phosphate cytidylyltransferase 1, choline, alph	Multiple_Complex
TC0900009702.hg.1	4.66	4.23	1.34	0.0094	0.0293	IFNA5	interferon, alpha 5	Coding
TC1500008015.hg.1	4.74	3.99	1.69	0.0094	0.0293	ST20-AS1	ST20 antisense RNA 1	Multiple_Complex
TC2200007477.hg.1	8.8	9.68	-1.84	0.0094	0.0293	ACO2	aconitase 2, mitochondrial	Multiple_Complex
TC1900011271.hg.1	19.19	18.77	1.34	0.0095	0.0293	FPR1	formyl peptide receptor 1	Coding
TC1300008938.hg.1	9.67	10.43	-1.69	0.0095	0.0293	DLEU2; MII	deleted in lymphocytic leukemia 2 (non-prote	Multiple_Complex
TC0300011135.hg.1	6.4	7	-1.52	0.0095	0.0294	VPRBP	Vpr (HIV-1) binding protein	Multiple_Complex
TC1200011225.hg.1	3.58	3.32	1.2	0.0095	0.0294	BEST3	bestrophin 3	Multiple_Complex
TC0700010139.hg.1	3.66	3.32	1.27	0.0095	0.0295	PAPOLB	poly(A) polymerase beta	Coding
TC0200009331.hg.1	4.34	4	1.27	0.0095	0.0295	POTEE	POTE ankyrin domain family, member E	Coding
TC2000010031.hg.1	7.39	8.01	-1.54	0.0095	0.0295	TAF4	TAF4 RNA polymerase II, TATA box binding pro	Multiple_Complex
TC0100013713.hg.1	10.52	9.84	1.61	0.0095	0.0295	STK40	serine/threonine kinase 40	Multiple_Complex
TC0400009379.hg.1	11.91	12.52	-1.53	0.0095	0.0296	SPCS3	signal peptidase complex subunit 3	Multiple_Complex
TC1900008020.hg.1	12.02	12.99	-1.95	0.0095	0.0296	ACTN4	actinin, alpha 4	Multiple_Complex
TC1200009980.hg.1	4.47	5	-1.45	0.0096	0.0296	GPRC5D	G protein-coupled receptor, class C, group 5, r	Coding
TC1200010149.hg.1	5.3	4.71	1.5	0.0096	0.0296	C12orf77	chromosome 12 open reading frame 77	Multiple_Complex
TC0100009394.hg.1	17.78	17.32	1.38	0.0096	0.0297	CD53	CD53 molecule	Multiple_Complex
TC2000009646.hg.1	12.09	11.52	1.48	0.0096	0.0297	CTSZ	cathepsin Z	Multiple_Complex

TC1400006915.hg.1	4.87	4.45	1.34	0.0096	0.0297	BRMS1L	Transcript Identified by AceView, Entrez Gene	Unassigned
TSUnmapped00000061.h	10.44	10.95	-1.42	0.0096	0.0297	FBL	fibrillarin	Coding
TC1000012399.hg.1	6.49	6.98	-1.41	0.0096	0.0297	ECHS1	enoyl-CoA hydratase, short chain, 1, mitochor	Multiple_Complex
TC0100014776.hg.1	5.68	5.26	1.34	0.0096	0.0297	DDAH1	dimethylarginine dimethylaminohydrolase 1	Multiple_Complex
TC1200012749.hg.1	8.33	7.69	1.55	0.0096	0.0297	CD163	CD163 molecule	Multiple_Complex
TC1600011425.hg.1	7.17	6.91	1.2	0.0096	0.0297	DDX19A	DEAD (Asp-Glu-Ala-Asp) box polypeptide 19A	Multiple_Complex
TC1700009590.hg.1	4.9	5.43	-1.44	0.0096	0.0297	KIAA0753	KIAA0753	Multiple_Complex
TC1700007372.hg.1	5.97	5.54	1.35	0.0096	0.0297	FOXN1	forkhead box N1	Multiple_Complex
TC1100006973.hg.1	4.82	4.37	1.37	0.0096	0.0297	OTOG	otogelin	Multiple_Complex
TC2200009336.hg.1	5.35	5.77	-1.34	0.0096	0.0297	NIPSNAP1	nipsnap homolog 1 (C. elegans)	Multiple_Complex
TC1900009576.hg.1	6.52	7.09	-1.49	0.0096	0.0297	ZNF699	zinc finger protein 699	Multiple_Complex
TC0500013089.hg.1	4.77	4.48	1.22	0.0096	0.0298	GFPT2	glutamine-fructose-6-phosphate transaminase	Multiple_Complex
TC0300013980.hg.1	4.02	4.3	-1.22	0.0096	0.0298	GMPPB	GDP-mannose pyrophosphorylase B	Multiple_Complex
TC0800009967.hg.1	4.29	4.81	-1.44	0.0096	0.0298	NUGGC	nuclear GTPase, germinal center associated	Coding
TC0200010046.hg.1	5.33	4.82	1.43	0.0096	0.0298	HOXD12	homeobox D12	Coding
TC0300012109.hg.1	5.5	5.06	1.36	0.0096	0.0298	IGSF11	immunoglobulin superfamily, member 11	Multiple_Complex
TC0900007351.hg.1	6.37	5.87	1.42	0.0096	0.0298	LINC01189	long intergenic non-protein coding RNA 1189	NonCoding
TSUnmapped00000397.h	7.88	8.64	-1.69	0.0096	0.0298	EIF3F	Eukaryotic translation initiation factor 3 subur	Coding
TC0100018416.hg.1	4.18	3.77	1.33	0.0097	0.0298	TAS1R2	taste receptor, type 1, member 2	Coding
TC2200008828.hg.1	8.11	7.58	1.45	0.0097	0.0299	PHF5A	PHD finger protein 5A	Multiple_Complex
TC0900008675.hg.1	5.08	4.51	1.49	0.0097	0.0299	OR1L6	olfactory receptor, family 1, subfamily L, mem	Coding
TC0200007035.hg.1	5.75	6.18	-1.36	0.0097	0.0299	EPT1	ethanolaminephosphotransferase 1	Multiple_Complex
TC1700011436.hg.1	9.76	9.24	1.44	0.0097	0.03	ERN1	endoplasmic reticulum to nucleus signaling 1	Multiple_Complex
TC0300008558.hg.1	10.29	11.52	-2.34	0.0097	0.03	DTX3L	deltex 3 like, E3 ubiquitin ligase	Multiple_Complex
TC1200012653.hg.1	7.9	7.5	1.31	0.0098	0.0301	METTL21B	methyltransferase like 21B	Multiple_Complex
TC2100007072.hg.1	9.25	9.99	-1.67	0.0098	0.0301	TTC3	tetratricopeptide repeat domain 3	Multiple_Complex
TC2000007633.hg.1	7.79	7.36	1.35	0.0098	0.0301	DDX27	DEAD (Asp-Glu-Ala-Asp) box polypeptide 27	Multiple_Complex
TC1000009851.hg.1	4.78	5.4	-1.53	0.0098	0.0301	FRMD4A	FERM domain containing 4A	Multiple_Complex
TC1700008328.hg.1	5.11	5.69	-1.49	0.0098	0.0303	STXBP4	syntaxin binding protein 4	Multiple_Complex
TC1300006633.hg.1	9.31	9.66	-1.27	0.0098	0.0303	PABPC3	poly(A) binding protein, cytoplasmic 3	Coding
TC1500007883.hg.1	5.4	5.85	-1.37	0.0098	0.0303	COMMD4	COMM domain containing 4	Multiple_Complex
TC1000009527.hg.1	6.81	7.5	-1.61	0.0098	0.0303	LARP4B	La ribonucleoprotein domain family, member	Multiple_Complex
TC0X00006839.hg.1	4.19	3.76	1.34	0.0099	0.0304	MAGEB6	MAGE family member B6	Coding
TC1700012184.hg.1	4.51	4.11	1.32	0.0099	0.0304	CHRNB1	cholinergic receptor, nicotinic beta 1	Multiple_Complex
TC1100011202.hg.1	6.36	6.01	1.27	0.0099	0.0304	TMEM262	transmembrane protein 262	Multiple_Complex
TC2200007963.hg.1	8.33	8.92	-1.5	0.0099	0.0304	DGCR2; DG	DiGeorge syndrome critical region gene 2; DiG	Multiple_Complex
TC1500009998.hg.1	6.7	6.41	1.22	0.0099	0.0305	GOLGA6A	golgin A6 family, member A	Multiple_Complex
TC0300013420.hg.1	7.93	8.91	-1.96	0.0099	0.0305	IGF2BP2	insulin-like growth factor 2 mRNA binding pro	Multiple_Complex
TC1200007844.hg.1	4.93	5.71	-1.72	0.0099	0.0305	RBMS2	RNA binding motif, single stranded interacting	Multiple_Complex
TC1500006818.hg.1	9.05	9.45	-1.32	0.0099	0.0305	EMC4	ER membrane protein complex subunit 4	Multiple_Complex
TC1600011225.hg.1	8.73	8.34	1.31	0.0099	0.0306	RNF166	ring finger protein 166	Multiple_Complex
TC0600013280.hg.1	9.51	9.76	-1.19	0.0099	0.0306	MAP3K5	mitogen-activated protein kinase kinase kinas	Multiple_Complex
TC0X00007053.hg.1	5.46	5.09	1.3	0.0099	0.0306	MAOA	monoamine oxidase A	Multiple_Complex
TC0500012145.hg.1	4.15	4.66	-1.43	0.01	0.0307	KLHL3	kelch-like family member 3	Multiple_Complex
TC1600009260.hg.1	4.91	5.56	-1.57	0.01	0.0307	EEF2KMT	eukaryotic elongation factor 2 lysine methyltr	Multiple_Complex
TC0100009866.hg.1	12.84	11.52	2.49	0.01	0.0307	FCGR1A	Fc fragment of IgG, high affinity Ia, receptor ((	Multiple_Complex
TC1000009841.hg.1	6.5	6.16	1.26	0.01	0.0307	PHYH	phytanoyl-CoA 2-hydroxylase	Multiple_Complex
TC1900011850.hg.1	5.34	5.1	1.18	0.01	0.0307	FUT5	fucosyltransferase 5 (alpha (1,3) fucosyltransf	Coding
TC1700011743.hg.1	7.44	7.96	-1.44	0.01	0.0308	GGA3	golgi-associated, gamma adaptin ear containir	Multiple_Complex
TC0400012212.hg.1	4.38	3.82	1.48	0.01	0.0308	ASIC5	acid sensing ion channel family member 5	Coding
TC1400007285.hg.1	8.54	8.95	-1.33	0.01	0.0308	NAA30	N(alpha)-acetyltransferase 30, NatC catalytic s	Multiple_Complex
TC1600010004.hg.1	4.3	4.71	-1.32	0.01	0.0308	ZNF646	Memczak2013 ANTISENSE, coding, INTERNAL,	NonCoding
TC0500010731.hg.1	8.27	9.32	-2.07	0.01	0.0308	ARL15	ADP-ribosylation factor like GTPase 15	Multiple_Complex
TC1100013189.hg.1	5.95	5.27	1.6	0.01	0.0309	CFL1	cofilin 1 (non-muscle)	NonCoding
TC1900007399.hg.1	5.89	5.47	1.34	0.01	0.0309	TMEM59L	transmembrane protein 59-like	Multiple_Complex
TC2000007105.hg.1	12.26	12.39	-1.09	0.0101	0.0309	TM9SF4	transmembrane 9 superfamily protein membe	Multiple_Complex
TC0100015994.hg.1	8.23	8.86	-1.55	0.0101	0.0309	CCT3	chaperonin containing TCP1, subunit 3 (gamm	Multiple_Complex
TC0500008884.hg.1	4.29	3.84	1.36	0.0101	0.031	PCDHB13	protocadherin beta 13	Coding
TC0500012127.hg.1	4.45	4.87	-1.33	0.0101	0.031	TRPC7	transient receptor potential cation channel, su	Multiple_Complex
TC2000009602.hg.1	8.85	10.41	-2.96	0.0101	0.031	ZBP1	Z-DNA binding protein 1	Multiple_Complex
TC1400010702.hg.1	4.46	4.81	-1.28	0.0101	0.031	TEX22	testis expressed 22	Multiple_Complex
TC0100013367.hg.1	4.93	4.5	1.35	0.0101	0.0311	AUNIP	aurora kinase A and ninein interacting protein	Multiple_Complex
TC0X00011354.hg.1	4.99	4.59	1.32	0.0101	0.0311	FIGF	c-fos induced growth factor (vascular endothe	Multiple_Complex
TC0100018510.hg.1	6.18	6.89	-1.64	0.0102	0.0312	GLMP	glycosylated lysosomal membrane protein	Multiple_Complex
TC0X00011198.hg.1	7.5	7.1	1.32	0.0102	0.0312	DNASE1L1	deoxyribonuclease I-like 1	Multiple_Complex
TC1100006749.hg.1	4.41	4.07	1.27	0.0102	0.0312	ZNF215	zinc finger protein 215	Multiple_Complex
TC0100011755.hg.1	4.77	4	1.7	0.0102	0.0312	DNAH14	dynein, axonemal, heavy chain 14	Multiple_Complex
TC0100011797.hg.1	7.35	7.03	1.25	0.0102	0.0313	C1orf95	chromosome 1 open reading frame 95	Multiple_Complex
TC0300011236.hg.1	3.64	3.27	1.29	0.0102	0.0313	LRTM1	leucine-rich repeats and transmembrane dom	Coding
TC1100009540.hg.1	6.4	5.98	1.34	0.0102	0.0313	C11orf44	chromosome 11 open reading frame 44	Multiple_Complex

TC0800006975.hg.1	4.9	4.76	1.11	0.0102	0.0314	BMP1	bone morphogenetic protein 1	Multiple_Complex
TC0100015789.hg.1	5.89	4.98	1.87	0.0102	0.0314	POGZ	Transcript Identified by AceView, Entrez Gene	Unassigned
TC1300009712.hg.1	6.34	5.81	1.45	0.0103	0.0314	EFNB2	ephrin-B2	Coding
TC0700013328.hg.1	3.46	3.88	-1.34	0.0103	0.0315	CHST12	carbohydrate (chondroitin 4) sulfotransferase	NonCoding
TC2100008577.hg.1	5.79	5.36	1.34	0.0103	0.0315	KRTAP10-6	keratin associated protein 10-6	Coding
TC0800012349.hg.1	5.25	5.79	-1.45	0.0103	0.0315	MTBP	MDM2 binding protein	Multiple_Complex
TC0200010817.hg.1	4.7	4.26	1.36	0.0103	0.0315	WNT6	wingless-type MMTV integration site family, n	Multiple_Complex
TC1700011430.hg.1	8.67	9.22	-1.47	0.0103	0.0315	CD79B	CD79b molecule, immunoglobulin-associated	Multiple_Complex
TC0400012967.hg.1	5.7	5.4	1.23	0.0103	0.0315	MGARP	mitochondria localized glutamic acid rich prot	Coding
TC1700009478.hg.1	4.6	4.19	1.33	0.0103	0.0315	OR1E1	olfactory receptor, family 1, subfamily E, mem	Coding
TC1200011855.hg.1	6.38	6.01	1.29	0.0103	0.0315	SVOP	SV2 related protein	Multiple_Complex
TC1200007881.hg.1	4.54	4.14	1.32	0.0103	0.0316	INHBE	inhibin beta E	Multiple_Complex
TC1200007796.hg.1	4.45	3.98	1.38	0.0103	0.0316	OR6C68	olfactory receptor, family 6, subfamily C, mem	Coding
TC1900010479.hg.1	5.03	4.47	1.47	0.0103	0.0316	DMKN	dermokine	Multiple_Complex
TC0X00007370.hg.1	3.3	3.75	-1.37	0.0103	0.0316	APEX2	Transcript Identified by AceView, Entrez Gene	Unassigned
TC0300012676.hg.1	6	5.55	1.36	0.0103	0.0316	PCOLCE2	procollagen C-endopeptidase enhancer 2	Multiple_Complex
TC1200007163.hg.1	5.07	5.5	-1.35	0.0103	0.0316	KLHL42	kelch-like family member 42	Multiple_Complex
TC0200016587.hg.1	6.11	5.65	1.37	0.0104	0.0317	PLCL1	phospholipase C-like 1	Multiple_Complex
TC0X00007229.hg.1	4.11	3.5	1.53	0.0104	0.0317	GAGE12F; (	G antigen 12F; G antigen 12J; G antigen 12D; (	Coding
TC0900009588.hg.1	5.72	6.17	-1.36	0.0104	0.0317	TTC39B	Transcript Identified by AceView, Entrez Gene	Unassigned
TC1000009893.hg.1	5.16	4.53	1.55	0.0104	0.0317	ITGA8	integrin alpha 8	Multiple_Complex
TC1700008228.hg.1	3.49	3.91	-1.34	0.0104	0.0318	ITGA3	integrin alpha 3	Multiple_Complex
TC1200010971.hg.1	9.62	9.43	1.14	0.0104	0.0318	DCTN2	dynactin 2 (p50)	Multiple_Complex
TC1400009786.hg.1	4.49	4.2	1.22	0.0104	0.0318	ISM2	isthmin 2	Multiple_Complex
TC1500007231.hg.1	4.81	4.46	1.27	0.0104	0.0318	GLDN	gliomedin	Multiple_Complex
TC0100018509.hg.1	6.38	5.85	1.44	0.0104	0.0319	GLMP	glycosylated lysosomal membrane protein	NonCoding
TC1500009508.hg.1	5.5	4.9	1.51	0.0104	0.0319	NEDD4	neural precursor cell expressed, development	Multiple_Complex
TC0800008349.hg.1	3.83	3.49	1.27	0.0104	0.0319	OSR2	odd-skipped related transciption factor 2	Multiple_Complex
TC1100013186.hg.1	10.1	10.32	-1.17	0.0104	0.0319	SYVN1	synovial apoptosis inhibitor 1, synoviolin	Multiple_Complex
TC0200014269.hg.1	4.12	3.66	1.37	0.0105	0.0319	POTEI	POTE ankyrin domain family, member I	Multiple_Complex
TC1100012381.hg.1	3.95	3.4	1.46	0.0105	0.0319	NXPE1	neurexophilin and PC-esterase domain family,	Coding
TC1200009926.hg.1	3.63	4.19	-1.48	0.0105	0.0319	TAS2R50	taste receptor, type 2, member 50	Coding
TC2000009242.hg.1	6.3	5.88	1.33	0.0105	0.0319	WFDC3	WAP four-disulfide core domain 3	Multiple_Complex
TC1200011882.hg.1	4.68	4.27	1.33	0.0105	0.0319	TRPV4	transient receptor potential cation channel, su	Multiple_Complex
TC1700012230.hg.1	12.87	13.33	-1.37	0.0105	0.0319	ZNF207; M	zinc finger protein 207; microRNA 632	Multiple_Complex
TC0100012588.hg.1	4.71	5.14	-1.35	0.0105	0.0319	MEGF6	multiple EGF-like-domains 6	Multiple_Complex
TC0500008702.hg.1	8.68	8.05	1.55	0.0105	0.0319	CAMLG	calcium modulating ligand	Multiple_Complex
TC1900011722.hg.1	7.66	8.29	-1.55	0.0105	0.032	HKR1	HKR1, GLI-Kruppel zinc finger family member	Multiple_Complex
TC0100014048.hg.1	5.42	5.83	-1.32	0.0105	0.032	POMGNT1	protein O-linked mannose N-acetylglucosamir	Multiple_Complex
TC0600010002.hg.1	5.31	5.76	-1.36	0.0105	0.032	SYNJ2	synaptojanin 2	Multiple_Complex
TC0200015019.hg.1	7.41	6.82	1.5	0.0105	0.0321	KIAA1715	KIAA1715	Multiple_Complex
TC1400008636.hg.1	7.47	7.99	-1.44	0.0105	0.0321	METTL3	methyltransferase like 3	Multiple_Complex
TC1400006506.hg.1	3.98	3.41	1.49	0.0105	0.0321	OR11G2	olfactory receptor, family 11, subfamily G, me	Coding
TC2000010008.hg.1	5.81	6.28	-1.38	0.0105	0.0321	NFS1	NFS1 cysteine desulfurase	Multiple_Complex
TC0300007448.hg.1	4.49	4.15	1.27	0.0106	0.0322	IQCF3	Transcript Identified by AceView, Entrez Gene	Unassigned
TC0300009451.hg.1	7.55	6.96	1.51	0.0106	0.0322	MYNN	myoneurin	Multiple_Complex
TC2200007439.hg.1	4.23	3.81	1.33	0.0106	0.0322	MCHR1	melanin-concentrating hormone receptor 1	Multiple_Complex
TC0800012280.hg.1	8.09	8.86	-1.71	0.0106	0.0322	PDLIM2	PDZ and LIM domain 2 (mystique)	Multiple_Complex
TC1200008601.hg.1	3.9	3.39	1.42	0.0106	0.0322	ANO4	anoctamin 4	Multiple_Complex
TC1100009284.hg.1	4.51	4.89	-1.3	0.0106	0.0323	POU2F3	POU class 2 homeobox 3	Multiple_Complex
TC0300011145.hg.1	3.76	3.36	1.32	0.0106	0.0324	IQCF1	IQ motif containing F1	Coding
TC1900011846.hg.1	6.19	5.83	1.28	0.0107	0.0324	PLIN5	perilipin 5	Multiple_Complex
TC0300013853.hg.1	4.13	3.68	1.37	0.0107	0.0324	ST3GAL6	ST3 beta-galactoside alpha-2,3-sialyltransfera:	NonCoding
TC1300007824.hg.1	8.32	8.81	-1.4	0.0107	0.0325	UBAC2	UBA domain containing 2	Multiple_Complex
TC1900011257.hg.1	4.19	4.79	-1.51	0.0107	0.0325	SIGLEC8	sialic acid binding Ig-like lectin 8	Multiple_Complex
TC0100013311.hg.1	10.42	10.79	-1.29	0.0107	0.0325	SRSF10	serine/arginine-rich splicing factor 10	Multiple_Complex
TC0100010518.hg.1	9.32	10.02	-1.62	0.0107	0.0325	DCAF6	DDB1 and CUL4 associated factor 6	Multiple_Complex
TC1600009162.hg.1	5.03	5.56	-1.45	0.0107	0.0325	HCFC1R1	host cell factor C1 regulator 1 (XPO1 depende	Multiple_Complex
TC1900007882.hg.1	4.87	5.4	-1.44	0.0107	0.0325	PROSER3	proline and serine rich 3	Multiple_Complex
TC0400007806.hg.1	4.01	3.62	1.31	0.0107	0.0325	NPFFR2	neuropeptide FF receptor 2	Multiple_Complex
TC0200014860.hg.1	4.55	4.01	1.45	0.0107	0.0326	CCDC173	coiled-coil domain containing 173	Coding
TC0300012170.hg.1	8.13	7.62	1.42	0.0107	0.0326	GOLGB1	golgin B1	Multiple_Complex
TC1100008126.hg.1	6.96	6.45	1.42	0.0107	0.0326	ANKRD13D	ankyrin repeat domain 13 family, member D	Multiple_Complex
TC0300008474.hg.1	6.09	6.71	-1.55	0.0107	0.0326	POGLUT1	protein O-glucosyltransferase 1	Multiple_Complex
TC1900011703.hg.1	4.89	4.39	1.42	0.0107	0.0326	ZNF536	zinc finger protein 536	NonCoding
TC1900007142.hg.1	3.83	4.13	-1.23	0.0107	0.0326	ZSWIM4	zinc finger, SWIM-type containing 4	Multiple_Complex
TC0500012596.hg.1	4.44	4.92	-1.4	0.0108	0.0327	FNDC9	fibronectin type III domain containing 9	Coding
TC0900006872.hg.1	5	4.47	1.44	0.0108	0.0327	LOC100506	putative deoxyuridine 5-triphosphate nucleoti	Multiple_Complex
TC1100009171.hg.1	5.62	6.15	-1.44	0.0108	0.0327	RNF214	ring finger protein 214	Multiple_Complex
TC0X00010954.hg.1	6.83	6.51	1.25	0.0108	0.0328	FGF13; LIN(	fibroblast growth factor 13; long intergenic nc	Multiple_Complex

TC0X00008836.hg.1	4.55	5.2	-1.57	0.0108	0.0328	PLXNA3	plexin A3	Multiple_Complex
TC0300008888.hg.1	11.55	10.76	1.73	0.0108	0.0328	CEP63	centrosomal protein 63kDa	Multiple_Complex
TC0300010521.hg.1	5.13	4.48	1.57	0.0108	0.0328	THRB	thyroid hormone receptor, beta	Multiple_Complex
TC0200009344.hg.1	4.77	4.32	1.36	0.0108	0.0328	CCDC74A	coiled-coil domain containing 74A	Multiple_Complex
TC0300013809.hg.1	13.23	14.45	-2.34	0.0108	0.0329	RPL14	ribosomal protein L14	Multiple_Complex
TC1600011409.hg.1	4.77	5.31	-1.46	0.0108	0.0329	PDP2	pyruvate dehyrogenase phosphatase catalytic	Multiple_Complex
TC0500006614.hg.1	7.67	8.11	-1.36	0.0108	0.0329	ICE1	interactor of little elongation complex ELL sub	Multiple_Complex
TC1700006644.hg.1	7.76	7.16	1.52	0.0109	0.0329	PSMB6	proteasome subunit beta 6	Multiple_Complex
TC1200010284.hg.1	5.84	6.49	-1.57	0.0109	0.033	DENND5B	DENN/MADD domain containing 5B	Multiple_Complex
TC1800008474.hg.1	8.02	8.46	-1.35	0.0109	0.0331	SLC39A6	solute carrier family 39 (zinc transporter), mer	Multiple_Complex
TC0600008262.hg.1	5.71	6.18	-1.38	0.0109	0.0331	TMEM14A	transmembrane protein 14A	Multiple_Complex
TC0500013370.hg.1	4.21	3.77	1.36	0.0109	0.0331	SIL1	SIL1 nucleotide exchange factor	NonCoding
TC0500013372.hg.1	5.52	5.03	1.41	0.0109	0.0331	ECSCR	endothelial cell surface expressed chemotaxis	Multiple_Complex
TC0100018464.hg.1	4.73	4.32	1.33	0.0109	0.0332	PPM1J	protein phosphatase, Mg2+/Mn2+ dependent	Multiple_Complex
TC1100006840.hg.1	7.46	8.06	-1.52	0.0109	0.0332	CTR9	CTR9 homolog, Paf1/RNA polymerase II comp	Multiple_Complex
TC0500007421.hg.1	7.67	6.76	1.88	0.0109	0.0332	SKIV2L2	superkiller viralicidic activity 2-like 2 (S. cerevi	Multiple_Complex
TC0500013336.hg.1	8.79	9.42	-1.55	0.011	0.0332	SSBP2	single-stranded DNA binding protein 2	Multiple_Complex
TC2000006956.hg.1	5.31	4.66	1.57	0.011	0.0332	NXT1	nuclear transport factor 2-like export factor 1	Multiple_Complex
TC0900010702.hg.1	12.27	12.63	-1.29	0.011	0.0332	SEMA4D	sema domain, immunoglobulin domain (Ig), tr	Multiple_Complex
TC2200007013.hg.1	9.91	10.36	-1.37	0.011	0.0332	EWSR1	EWS RNA binding protein 1	Multiple_Complex
TC0300013840.hg.1	6.79	6.28	1.42	0.011	0.0333	ABHD14A-/	ABHD14A-ACY1 readthrough	Multiple_Complex
TC2200006637.hg.1	6.66	7.28	-1.54	0.011	0.0333	DGCR8	Memczak2013 ALT_ACCEPTOR, ALT_DONOR,	NonCoding
TC1400009912.hg.1	10.35	9.98	1.29	0.011	0.0334	GALC	galactosylceramidase	Multiple_Complex
TC1200010552.hg.1	5.48	5.18	1.24	0.011	0.0334	ENDOU	endonuclease, polyU-specific	Multiple_Complex
TC1300008108.hg.1	6.6	6.2	1.32	0.011	0.0334	F7	coagulation factor VII (serum prothrombin cor	Multiple_Complex
TC0700012871.hg.1	4.78	4.32	1.38	0.0111	0.0335	OR9A2	olfactory receptor, family 9, subfamily A, mem	Coding
TC1600008568.hg.1	8.95	9.69	-1.67	0.0111	0.0336	ATMIN	ATM interactor	Multiple_Complex
TC0Y00007321.hg.1	5.24	4.8	1.36	0.0111	0.0336	ASMT	Homo sapiens acetylserotonin O-methyltransf	Coding
TC0500009629.hg.1	8.13	7.6	1.44	0.0111	0.0336	TMED9	transmembrane p24 trafficking protein 9	Multiple_Complex
TC1200012773.hg.1	3.85	3.36	1.4	0.0111	0.0336	ST8SIA1	ST8 alpha-N-acetyl-neuraminide alpha-2,8-sia	NonCoding
TC1700011919.hg.1	11.89	10.83	2.08	0.0111	0.0336	CEP295NL;	CEP295 N-terminal like; TIMP metallopeptidas	Multiple_Complex
TC1200011470.hg.1	11.16	11.87	-1.64	0.0111	0.0336	DUSP6	dual specificity phosphatase 6	Multiple_Complex
TC1900008882.hg.1	7.48	7.69	-1.16	0.0111	0.0336	NCR1	natural cytotoxicity triggering receptor 1	Multiple_Complex
TC0800012433.hg.1	8.38	8.89	-1.43	0.0111	0.0336	TPD52	tumor protein D52	Multiple_Complex
TC0700007034.hg.1	14.56	15.44	-1.84	0.0111	0.0336	CREB5	cAMP responsive element binding protein 5	Multiple_Complex
TC0300010889.hg.1	5.51	5.94	-1.34	0.0112	0.0337	ZNF445	zinc finger protein 445	Multiple_Complex
TC1800008189.hg.1	3.88	3.44	1.36	0.0112	0.0337	POTEC	POTE ankyrin domain family, member C	Multiple_Complex
TC1100007838.hg.1	3.97	3.53	1.36	0.0112	0.0337	SCGB2A1	secretoglobin, family 2A, member 1	Coding
TC1900011941.hg.1	9.53	10.25	-1.65	0.0112	0.0337	HNRNPL	heterogeneous nuclear ribonucleoprotein L	Multiple_Complex
TC0400008160.hg.1	4.08	3.68	1.32	0.0112	0.0337	GRID2	glutamate receptor, ionotropic, delta 2	Multiple_Complex
TC2000008533.hg.1	4.49	5.02	-1.44	0.0112	0.0337	RBBP9	retinoblastoma binding protein 9	Multiple_Complex
TC1100010751.hg.1	9.66	9.25	1.33	0.0112	0.0337	KBTBD4	kelch repeat and BTB (POZ) domain containing	Multiple_Complex
TC0500007607.hg.1	5.45	6.04	-1.5	0.0112	0.0337	NLN	neurolysin (metallopeptidase M3 family)	Multiple_Complex
TC0900010462.hg.1	6.5	7.15	-1.56	0.0112	0.0338	RFK	riboflavin kinase	Multiple_Complex
TC1800007411.hg.1	6.18	7.14	-1.94	0.0112	0.0338	NEDD4L	neural precursor cell expressed, development	Multiple_Complex
TC0500009423.hg.1	6.71	5.81	1.88	0.0112	0.0339	NPM1	Zhang2013 ALT_ACCEPTOR, ALT_DONOR, cod	NonCoding
TC0300011517.hg.1	14.66	13.6	2.08	0.0112	0.0339	PROK2	prokineticin 2	Coding
TC0200006951.hg.1	6.58	5.42	2.23	0.0112	0.0339	UBXN2A	UBX domain protein 2A	Multiple_Complex
TC1600008000.hg.1	5.08	4.82	1.19	0.0112	0.0339	CCL17	chemokine (C-C motif) ligand 17	Coding
TC0700013047.hg.1	6.89	10.31	-10.73	0.0112	0.0339	TMEM176E	transmembrane protein 176B	Coding
TC0700007467.hg.1	4.28	3.89	1.31	0.0113	0.034	C7orf65	chromosome 7 open reading frame 65	Multiple_Complex
TC0100013548.hg.1	9.12	10.17	-2.07	0.0113	0.034	PUM1	pumilio RNA binding family member 1	Multiple_Complex
TC1800009283.hg.1	10.76	10.34	1.34	0.0113	0.034	SMAD2	SMAD family member 2	Multiple_Complex
TC2200008682.hg.1	5.53	6.1	-1.48	0.0113	0.034	PLA2G6	phospholipase A2, group VI (cytosolic, calcium	Multiple_Complex
TC1900009287.hg.1	5.91	6.32	-1.33	0.0113	0.0341	SMIM24	small integral membrane protein 24	Multiple_Complex
TC0100007447.hg.1	4.12	4.5	-1.3	0.0113	0.0341	CEP85	centrosomal protein 85kDa	Multiple_Complex
TC0200010246.hg.1	8.99	9.36	-1.29	0.0113	0.0341	WDR75	WD repeat domain 75	Multiple_Complex
TC0100017420.hg.1	5.59	5.01	1.49	0.0113	0.0341	DUSP10	dual specificity phosphatase 10	Multiple_Complex
TC0500012501.hg.1	8.97	8.63	1.26	0.0113	0.0341	CCDC69	coiled-coil domain containing 69	Multiple_Complex
TC0X00007977.hg.1	4.81	5.33	-1.44	0.0114	0.0342	GPRASP1	G protein-coupled receptor associated sorting	Multiple_Complex
TC0200014717.hg.1	5.39	5.9	-1.42	0.0114	0.0342	WDSUB1	WD repeat, sterile alpha motif and U-box dom	Multiple_Complex
TC1400009711.hg.1	8.98	9.55	-1.48	0.0114	0.0342	NEK9	NIMA-related kinase 9	Multiple_Complex
TC1100009685.hg.1	4.65	5.63	-1.97	0.0114	0.0342	IRF7	interferon regulatory factor 7	Multiple_Complex
TC1200008845.hg.1	4.52	4.03	1.41	0.0114	0.0342	RAD9B	RAD9 checkpoint clamp component B	Multiple_Complex
TC0400009714.hg.1	5.86	5.56	1.23	0.0114	0.0343	SLC26A1	solute carrier family 26 (anion exchanger), me	Multiple_Complex
TC0900009301.hg.1	6.08	6.49	-1.32	0.0114	0.0343	MRPL41	mitochondrial ribosomal protein L41	Multiple_Complex
TC0200014192.hg.1	9.1	9.69	-1.51	0.0114	0.0344	SAP130	Sin3A associated protein 130kDa	Multiple_Complex
TC0100011153.hg.1	4.79	4.29	1.42	0.0114	0.0344	C1orf106	chromosome 1 open reading frame 106	Multiple_Complex
TC1700011096.hg.1	4.53	4.88	-1.27	0.0115	0.0344	MRPL27	mitochondrial ribosomal protein L27	Multiple_Complex
TC0100007303.hg.1	6.39	6.77	-1.3	0.0115	0.0344	KDM1A; M	lysine (K)-specific demethylase 1A; microRNA	Multiple_Complex

TC0200007805.hg.1	12.76	12.34	1.34	0.0115	0.0344	AFTPH	aftiphilin	Multiple_Complex
TC1900011587.hg.1	7.43	7.76	-1.25	0.0115	0.0344	ZNF552	zinc finger protein 552	Multiple_Complex
TC0700013395.hg.1	6.82	7.57	-1.68	0.0115	0.0344	GTPBP10	GTP-binding protein 10 (putative)	Multiple_Complex
TC1200011865.hg.1	4.87	4.53	1.26	0.0115	0.0344	FOXN4	forkhead box N4	Coding
TC0700013471.hg.1	5.19	4.57	1.54	0.0115	0.0344	MGAM	maltase-glucoamylase	NonCoding
TC2000007943.hg.1	6.9	6.43	1.38	0.0115	0.0345	CDH26	cadherin 26	Multiple_Complex
TC1700011768.hg.1	7.2	6.83	1.29	0.0115	0.0346	GALK1	galactokinase 1	Multiple_Complex
TC0300012009.hg.1	6.81	7.26	-1.36	0.0115	0.0346	C3orf17	chromosome 3 open reading frame 17	Multiple_Complex
TC1200011598.hg.1	10.28	9.3	1.98	0.0115	0.0346	HAL	histidine ammonia-lyase	Multiple_Complex
TC0X00006841.hg.1	5.51	4.84	1.59	0.0115	0.0346	MAGEB5	MAGE family member B5	Coding
TC1700006449.hg.1	5.77	6.07	-1.23	0.0116	0.0348	C17orf97	chromosome 17 open reading frame 97	Multiple_Complex
TC0900009673.hg.1	9.91	9.1	1.74	0.0116	0.0348	MLLT3	myeloid/lymphoid or mixed-lineage leukemia;	Multiple_Complex
TC2200007211.hg.1	3.69	3.32	1.29	0.0116	0.0348	RASD2	RASD family, member 2	Coding
TC0X00011144.hg.1	4.3	4.05	1.18	0.0116	0.0348	PNMA5	paraneoplastic Ma antigen family member 5	Multiple_Complex
TC0900009960.hg.1	4.2	3.8	1.32	0.0116	0.0348	OR13J1	olfactory receptor, family 13, subfamily J, mer	Coding
TC1700009861.hg.1	9.38	9.07	1.24	0.0116	0.0349	TVP23C; CI	trans-golgi network vesicle protein 23 homolo	Multiple_Complex
TC1900011920.hg.1	7.76	8.24	-1.39	0.0116	0.0349	ZNF98	zinc finger protein 98	Multiple_Complex
TC2200009365.hg.1	8.72	8.23	1.4	0.0117	0.035	CHKB	choline kinase beta	Multiple_Complex
TC1400006483.hg.1	4.39	3.82	1.48	0.0117	0.035	OR4N2	olfactory receptor, family 4, subfamily N, men	Coding
TC0500013391.hg.1	7.6	8.1	-1.41	0.0117	0.035	PWWP2A	PWWP domain containing 2A	Multiple_Complex
TC2000008279.hg.1	7.03	8.1	-2.09	0.0117	0.035	PCNA	proliferating cell nuclear antigen	Multiple_Complex
TC1700012412.hg.1	4.86	4.19	1.59	0.0117	0.035	KRT33B	keratin 33B, type I	Coding
TC2200008615.hg.1	4.5	4.01	1.4	0.0117	0.0351	CACNG2	calcium channel, voltage-dependent, gamma :	NonCoding
TC1200006450.hg.1	10.07	10.64	-1.48	0.0117	0.0351	WNK1	WNK lysine deficient protein kinase 1	Multiple_Complex
TC0300009531.hg.1	4.29	3.8	1.4	0.0117	0.0351	NLGN1	neuroligin 1	Multiple_Complex
TC0100007791.hg.1	4.85	5.32	-1.39	0.0117	0.0352	ADPRHL2	ADP-ribosylhydrolase like 2	Multiple_Complex
TC0100009902.hg.1	10.32	9.95	1.29	0.0118	0.0352	PRPF3	pre-mRNA processing factor 3	Multiple_Complex
TC1900009487.hg.1	6.39	6.08	1.24	0.0118	0.0352	XAB2	XPA binding protein 2	Multiple_Complex
TC0200011421.hg.1	6.17	5.69	1.4	0.0118	0.0352	ING5	inhibitor of growth family member 5	Multiple_Complex
TC1900006979.hg.1	4.21	4.54	-1.26	0.0118	0.0352	ICAM5	intercellular adhesion molecule 5	Multiple_Complex
TC0900007100.hg.1	4.57	4.84	-1.21	0.0118	0.0352	NPR2	natriuretic peptide receptor 2	Multiple_Complex
TC0700006650.hg.1	7.29	7.92	-1.54	0.0118	0.0352	C7orf26	chromosome 7 open reading frame 26	Multiple_Complex
TC0100014313.hg.1	4.67	4.13	1.46	0.0118	0.0353	PPAP2B	Transcript Identified by AceView, Entrez Gene	Unassigned
TC1600011451.hg.1	9.37	9.02	1.27	0.0118	0.0353	TCF25	transcription factor 25 (basic helix-loop-helix)	Multiple_Complex
TC1900008017.hg.1	6.37	6.05	1.25	0.0118	0.0353	RYR1	ryanodine receptor 1 (skeletal)	Multiple_Complex
TC1100010998.hg.1	7.34	7.9	-1.47	0.0118	0.0354	MS4A4E	membrane-spanning 4-domains, subfamily A,	Multiple_Complex
TC1400006529.hg.1	5.18	4.69	1.41	0.0118	0.0354	ANG; RNAS	angiogenin, ribonuclease, RNase A family, 5; r	Multiple_Complex
TC0100018179.hg.1	5.47	5.05	1.34	0.0118	0.0354	CLCNKA	chloride channel, voltage-sensitive Ka	NonCoding
TC0500013231.hg.1	5.28	5.71	-1.35	0.0118	0.0354	CATSPER3	cation channel, sperm associated 3	Multiple_Complex
TC0900012155.hg.1	4.31	3.87	1.36	0.0119	0.0354	MSANTD3-	MSANTD3-TMEFF1 readthrough	Coding
TC0100016339.hg.1	5.46	4.97	1.4	0.0119	0.0355	DPT	dermatopontin	Coding
TC1400010780.hg.1	7.4	7.02	1.3	0.0119	0.0355	ATXN3	ataxin 3	Multiple_Complex
TC1400006522.hg.1	4.18	3.72	1.38	0.0119	0.0355	RNASE10	ribonuclease, RNase A family, 10 (non-active)	Coding
TC1500008511.hg.1	8.17	8.55	-1.3	0.0119	0.0355	LRRC28	leucine rich repeat containing 28	Multiple_Complex
TC0500007808.hg.1	9.54	9.9	-1.28	0.0119	0.0355	POLK	polymerase (DNA directed) kappa	Multiple_Complex
TC2100008385.hg.1	8.91	8.51	1.31	0.0119	0.0355	SUMO3	small ubiquitin-like modifier 3	Multiple_Complex
TC0X00011402.hg.1	6.78	7.27	-1.4	0.0119	0.0355	SPANXA1	sperm protein associated with the nucleus, X-	Coding
TC0Y00007322.hg.1	9.12	7.82	2.46	0.0119	0.0355	RPS4Y1	ribosomal protein S4, Y-linked 1	Multiple_Complex
TC1300006879.hg.1	4.9	4.6	1.23	0.0119	0.0356	CCNA1	cyclin A1	Multiple_Complex
TC1700007474.hg.1	7.09	6.64	1.37	0.0119	0.0356	RNF135	ring finger protein 135	Multiple_Complex
TC1600008675.hg.1	12.2	11.69	1.43	0.012	0.0357	KIAA0513	KIAA0513	Multiple_Complex
TC0900011582.hg.1	5.37	4.8	1.48	0.012	0.0357	FAM129B	family with sequence similarity 129, member	Multiple_Complex
TC1700012235.hg.1	3.99	3.59	1.33	0.012	0.0357	UNC45B	unc-45 myosin chaperone B	Coding
TC0600007285.hg.1	9.8	8.96	1.79	0.012	0.0357	HIST1H2AE	histone cluster 1, H2ae	Coding
TC0100006784.hg.1	6.68	6.23	1.37	0.012	0.0357	TMEM201	transmembrane protein 201	Multiple_Complex
TC0900008969.hg.1	5.87	6.33	-1.37	0.012	0.0358	ABL1	ABL proto-oncogene 1, non-receptor tyrosine	Multiple_Complex
TC1600010122.hg.1	4.88	4.79	1.06	0.012	0.0359	LINC00273	long intergenic non-protein coding RNA 273	Multiple_Complex
TC2000010007.hg.1	9.74	10.47	-1.66	0.0121	0.036	RBM12; CP	RNA binding motif protein 12; copine I	Multiple_Complex
TC1900009684.hg.1	5.13	5.62	-1.4	0.0121	0.036	ACP5	acid phosphatase 5, tartrate resistant	Multiple_Complex
TC1700009122.hg.1	6.7	6.2	1.41	0.0121	0.036	SLC26A11	solute carrier family 26 (anion exchanger), me	Multiple_Complex
TC1900007852.hg.1	4.67	4.46	1.16	0.0121	0.036	FFAR3	free fatty acid receptor 3	Coding
TC0800007957.hg.1	5.24	5.99	-1.68	0.0121	0.036	RDH10	retinol dehydrogenase 10 (all-trans)	Multiple_Complex
TC0X00008736.hg.1	6.16	5.76	1.32	0.0121	0.036	FATE1	fetal and adult testis expressed 1	Coding
TC1900012023.hg.1	7.09	7.55	-1.37	0.0121	0.036	ZNF836	zinc finger protein 836	Multiple_Complex
TC0600009332.hg.1	4.62	4.2	1.34	0.0121	0.036	FABP7	fatty acid binding protein 7, brain	Coding
TC0300013386.hg.1	5	4.59	1.33	0.0121	0.036	THPO	thrombopoietin	Multiple_Complex
TC2100008380.hg.1	7.5	7.03	1.38	0.0121	0.0361	KRTAP12-2	keratin associated protein 12-2	Coding
TC0300007247.hg.1	5.41	5.66	-1.19	0.0121	0.0361	CCR9	chemokine (C-C motif) receptor 9	Multiple_Complex
TC1100010941.hg.1	4.38	3.91	1.38	0.0121	0.0361	OR5B3	olfactory receptor, family 5, subfamily B, mem	Coding
TC0500012039.hg.1	6.45	5.63	1.77	0.0121	0.0361	ZCCHC10	zinc finger, CCHC domain containing 10	Multiple_Complex

TC0700013606.hg.1	8.25	8.85	-1.51	0.0121	0.0361	THAP5	THAP domain containing 5	Multiple_Complex
TC0X00009203.hg.1	4.6	4.23	1.3	0.0121	0.0361	MAP3K15	mitogen-activated protein kinase kinase kinas	Multiple_Complex
TC2000007945.hg.1	6.29	6.88	-1.5	0.0122	0.0362	C20orf197	chromosome 20 open reading frame 197	Multiple_Complex
TC1500010845.hg.1	4.13	4.44	-1.24	0.0122	0.0362	OTUD7A	OTU deubiquitinase 7A	Multiple_Complex
TC1900008221.hg.1	4.95	4.59	1.28	0.0122	0.0362	PINLYP	phospholipase A2 inhibitor and LY6/PLAUR do	Multiple_Complex
TC1800008402.hg.1	6.47	6.06	1.33	0.0122	0.0362	SLC25A52	solute carrier family 25, member 52	Coding
TC1600008938.hg.1	7.11	7.49	-1.3	0.0122	0.0363	AXIN1	axin 1	Multiple_Complex
TC0900006451.hg.1	4.28	3.8	1.39	0.0122	0.0364	DMRT1	doublesex and mab-3 related transcription fac	Multiple_Complex
TC1000011522.hg.1	9.04	8.58	1.37	0.0123	0.0364	TM9SF3	transmembrane 9 superfamily member 3	Multiple_Complex
TC1200009566.hg.1	6.16	6.58	-1.35	0.0123	0.0365	RAD52	RAD52 homolog, DNA repair protein	Multiple_Complex
TC0100006806.hg.1	10.05	10.53	-1.4	0.0123	0.0365	UBE4B	ubiquitination factor E4B	Multiple_Complex
TC0800009240.hg.1	9.93	10.77	-1.79	0.0123	0.0365	MAF1	MAF1 homolog, negative regulator of RNA pol	Multiple_Complex
TC0200014345.hg.1	5.1	4.6	1.42	0.0123	0.0366	ANKRD30B	ankyrin repeat domain 30B-like	Multiple_Complex
TC0100014752.hg.1	11.38	10.87	1.43	0.0123	0.0366	GNG5	guanine nucleotide binding protein (G protein	Multiple_Complex
TC1500009263.hg.1	6.38	7.31	-1.91	0.0123	0.0366	PATL2	protein associated with topoisomerase II hom	Multiple_Complex
TSUnmapped00000098.h	5.52	5.08	1.35	0.0124	0.0367	DGKD	diacylglycerol kinase, delta 130kDa	Coding
TC0X00008365.hg.1	4	3.62	1.3	0.0124	0.0367	PRR32	proline rich 32	Coding
TC0700011865.hg.1	3.51	3.3	1.16	0.0124	0.0367	DLX5	distal-less homeobox 5	Multiple_Complex
TC0300006994.hg.1	4.15	3.75	1.32	0.0124	0.0367	FBXL2	F-box and leucine-rich repeat protein 2	Multiple_Complex
TC0700009329.hg.1	6.65	5.99	1.58	0.0124	0.0368	TTC26	tetratricopeptide repeat domain 26	Multiple_Complex
TC1400007445.hg.1	6.61	7.12	-1.42	0.0124	0.0368	PLEKHG3	pleckstrin homology domain containing, famil	Multiple_Complex
TC0800009433.hg.1	9.73	7.47	4.79	0.0124	0.0368	DEFA4	defensin, alpha 4, corticostatin	Coding
TC1700008566.hg.1	7.01	6.57	1.36	0.0124	0.0368	MAP3K3	mitogen-activated protein kinase kinase kinas	Multiple_Complex
TC1100010809.hg.1	4.54	4.27	1.21	0.0124	0.0368	OR4C12	olfactory receptor, family 4, subfamily C, mem	Coding
TSUnmapped00000310.h	5.88	6.33	-1.36	0.0124	0.0368	HYOU1	hypoxia up-regulated 1	Coding
TC0100012849.hg.1	7.42	7.86	-1.36	0.0124	0.0368	CASZ1	castor zinc finger 1	Multiple_Complex
TC0800011211.hg.1	6.21	7.31	-2.14	0.0124	0.0369	NIPAL2	NIPA-like domain containing 2	Multiple_Complex
TC1000012471.hg.1	4.44	4.83	-1.31	0.0125	0.037	ADIRF; AGA	adipogenesis regulatory factor; ankyrin repeat	Multiple_Complex
TC1100010718.hg.1	4.54	3.91	1.54	0.0125	0.037	LRP4	LDL receptor related protein 4	Multiple_Complex
TC0100015509.hg.1	5.66	6.51	-1.79	0.0125	0.037	FAM72B	family with sequence similarity 72, member B	Multiple_Complex
TC1900011743.hg.1	7.34	6.59	1.68	0.0125	0.037	TMEM91	transmembrane protein 91	Multiple_Complex
TC1700011045.hg.1	8.41	8.91	-1.41	0.0125	0.037	PHB	prohibitin	Multiple_Complex
TC0100009715.hg.1	10.33	10.72	-1.31	0.0125	0.0371	PPIAL4F	peptidylprolyl isomerase A (cyclophilin A)-like	Coding
TC1000011652.hg.1	8.2	8.52	-1.24	0.0125	0.0371	FBXW4	F-box and WD repeat domain containing 4	Multiple_Complex
TC0200013001.hg.1	4.99	5.5	-1.43	0.0125	0.0371	ADD2	adducin 2 (beta)	Multiple_Complex
TC1900007619.hg.1	5.65	6.08	-1.34	0.0125	0.0371	ZNF726; ZN	zinc finger protein 726; zinc finger protein 92	Multiple_Complex
TC0600008991.hg.1	5.75	6.36	-1.53	0.0125	0.0372	QRSL1	Transcript Identified by AceView, Entrez Gene	Unassigned
TC1100006644.hg.1	8.57	9.29	-1.64	0.0126	0.0372	RRM1	ribonucleotide reductase M1	Multiple_Complex
TC1700009372.hg.1	9.79	9.11	1.6	0.0126	0.0372	CRK	v-crk avian sarcoma virus CT10 oncogene hom	Multiple_Complex
TC0500013207.hg.1	3.63	4.72	-2.13	0.0126	0.0372	LIX1	Memczak2013 ANTISENSE, coding, INTERNAL,	NonCoding
TC1200006838.hg.1	4.5	4.92	-1.34	0.0126	0.0373	SMIM10L1	small integral membrane protein 10 like 1	Multiple_Complex
TC1000008309.hg.1	4.77	4.71	1.04	0.0126	0.0373	OPN4	opsin 4	Multiple_Complex
TC1400010707.hg.1	6.73	7.22	-1.41	0.0126	0.0373	KIAA0125	KIAA0125	Multiple_Complex
TC0100007191.hg.1	5.79	5.32	1.39	0.0126	0.0373	PLA2G2F	phospholipase A2, group IIF	Multiple_Complex
TC1600010616.hg.1	5.39	5.9	-1.42	0.0126	0.0373	TRADD	TNFRSF1A-associated via death domain	Multiple_Complex
TC1500006890.hg.1	4.38	3.94	1.35	0.0126	0.0373	TMCO5A	transmembrane and coiled-coil domains 5A	Multiple_Complex
TC1800007682.hg.1	4.82	4.45	1.29	0.0126	0.0374	CNDP1	carnosine dipeptidase 1 (metallopeptidase M2	Multiple_Complex
TC0200015894.hg.1	6.68	7.11	-1.34	0.0126	0.0374	DOCK10	Transcript Identified by AceView, Entrez Gene	Unassigned
TSUnmapped00000313.h	6.72	7.36	-1.56	0.0126	0.0374	CCDC84	coiled-coil domain containing 84	NonCoding
TC1400010563.hg.1	4.48	5.06	-1.5	0.0126	0.0374	MIR5195; I	microRNA 5195; immunoglobulin heavy variab	Multiple_Complex
TC1100010015.hg.1	4.68	4.39	1.22	0.0126	0.0374	NLRP10	NLR family, pyrin domain containing 10	Coding
TC2000009748.hg.1	5.4	5.86	-1.37	0.0127	0.0374	CABLES2	Cdk5 and Abl enzyme substrate 2	Multiple_Complex
TC1300009488.hg.1	4.09	3.78	1.24	0.0127	0.0374	DCT	dopachrome tautomerase	Multiple_Complex
TC1900010631.hg.1	6.6	7.03	-1.35	0.0127	0.0375	RINL	Ras and Rab interactor like	Multiple_Complex
TC1000011005.hg.1	7	6.53	1.39	0.0127	0.0375	P4HA1	prolyl 4-hydroxylase, alpha polypeptide I	Multiple_Complex
TC0800006837.hg.1	4.82	4.36	1.37	0.0127	0.0375	TUSC3	tumor suppressor candidate 3	Multiple_Complex
TC0700010789.hg.1	4.6	4.32	1.21	0.0127	0.0375	AMPH	amphiphysin	Multiple_Complex
TC1200007510.hg.1	4.39	3.92	1.39	0.0127	0.0375	CCDC184	coiled-coil domain containing 184	Coding
TC0300013687.hg.1	4.84	4.29	1.46	0.0127	0.0375	TFRC	Transcript Identified by AceView, Entrez Gene	Unassigned
TC1200009972.hg.1	4.66	5.05	-1.31	0.0127	0.0375	GPR19	G protein-coupled receptor 19	Multiple_Complex
TC0200011419.hg.1	7.4	6.77	1.55	0.0127	0.0376	ATG4B	autophagy related 4B, cysteine peptidase	Multiple_Complex
TC0300012815.hg.1	11.33	12.04	-1.64	0.0128	0.0376	P2RY13	purinergic receptor P2Y, G-protein coupled, 1	Coding
TC0900007069.hg.1	4.67	4.25	1.34	0.0128	0.0376	C9orf131	chromosome 9 open reading frame 131	Coding
TC1000011799.hg.1	3.54	4.16	-1.54	0.0128	0.0377	XPNPEP1	Transcript Identified by AceView, Entrez Gene	Unassigned
TC0600010064.hg.1	5.44	4.85	1.5	0.0128	0.0378	MAS1	MAS1 proto-oncogene, G protein-coupled rec	Coding
TC0400011749.hg.1	5.8	6.22	-1.34	0.0128	0.0378	BBS7	Bardet-Biedl syndrome 7	Multiple_Complex
TC0X00011334.hg.1	6.82	7.42	-1.51	0.0128	0.0378	BRCC3	BRCA1/BRCA2-containing complex subunit 3	Multiple_Complex
TC0400011823.hg.1	9.07	9.74	-1.58	0.0128	0.0378	PGRMC2	progesterone receptor membrane component	Multiple_Complex
TC0700012695.hg.1	9.01	9.73	-1.64	0.0128	0.0379	C7orf49	chromosome 7 open reading frame 49	Multiple_Complex
TC0X00007686.hg.1	6.1	5.5	1.51	0.0128	0.0379	PBDC1	polysaccharide biosynthesis domain containin	Multiple_Complex

TC0100018311.hg.1	5.61	5.36	1.19	0.0128	0.0379	NOS1AP	nitric oxide synthase 1 (neuronal) adaptor pro	Multiple_Complex
TC0100018551.hg.1	4.62	4.23	1.31	0.0128	0.0379	CHIT1	chitinase 1 (chitotriosidase)	Multiple_Complex
TC0900010796.hg.1	4.41	3.98	1.35	0.0129	0.0379	OMD	osteomodulin	Coding
TC1200012043.hg.1	13.24	12.57	1.59	0.0129	0.0379	MED13L	mediator complex subunit 13-like	Multiple_Complex
TSUnmapped00000421.h	6.19	6.69	-1.42	0.0129	0.0379	ATG16L1	autophagy related 16-like 1	Coding
TC0100015186.hg.1	4.62	4.15	1.39	0.0129	0.0379	C1orf194	chromosome 1 open reading frame 194	Multiple_Complex
TC1000008382.hg.1	7.5	6.79	1.63	0.0129	0.0379	LIPN	lipase, family member N	Coding
TC1500008796.hg.1	5.36	5.87	-1.42	0.0129	0.0379	HERC2	HECT and RLD domain containing E3 ubiquitin	Multiple_Complex
TC1900010531.hg.1	6.88	7.2	-1.25	0.0129	0.0379	ZNF461	zinc finger protein 461	Multiple_Complex
TC1500007163.hg.1	8.28	8.7	-1.34	0.0129	0.0379	DUT	deoxyuridine triphosphatase	Multiple_Complex
TC0100006781.hg.1	5.46	4.83	1.56	0.0129	0.0379	SLC25A33	solute carrier family 25 (pyrimidine nucleotide	Multiple_Complex
TC2000008515.hg.1	5.98	6.95	-1.96	0.0129	0.0379	SNX5	Jeck2013 ALT_DONOR, coding, INTERNAL, intr	NonCoding
TC1700009236.hg.1	10.11	9.39	1.64	0.0129	0.0379	SLC16A3; M	solute carrier family 16 (monocarboxylate tran	Multiple_Complex
TC0600011957.hg.1	5.66	5.2	1.37	0.0129	0.0379	ADGRF1	adhesion G protein-coupled receptor F1	Multiple_Complex
TC2000009017.hg.1	5.07	5.48	-1.33	0.0129	0.0379	SOGA1	suppressor of glucose, autophagy associated 1	Multiple_Complex
TC1100007414.hg.1	4.66	4.27	1.32	0.0129	0.0379	LOC399886	uncharacterized LOC399886; Transcript Identi	Multiple_Complex
TC0100018081.hg.1	3.9	4.25	-1.28	0.0129	0.0379	ZNF670; ZN	zinc finger protein 670; zinc finger protein 695	Multiple_Complex
TC1700012482.hg.1	5.87	5.51	1.29	0.0129	0.0379	SIRT7	sirtuin 7	Multiple_Complex
TC0800011216.hg.1	9.2	8.33	1.83	0.0129	0.0379	STK3	serine/threonine kinase 3	Multiple_Complex
TC0600014112.hg.1	4.4	4.68	-1.22	0.0129	0.0379	ZBTB9	zinc finger and BTB domain containing 9	Multiple_Complex
TC0100010016.hg.1	6.36	5.9	1.37	0.0129	0.038	SPRR4	small proline-rich protein 4	Coding
TC1100010851.hg.1	4.54	4.11	1.34	0.0129	0.038	OR8H1	olfactory receptor, family 8, subfamily H, mem	Coding
TC0100011901.hg.1	7.31	6.79	1.43	0.0129	0.038	RAB4A; SPH	RAB4A, member RAS oncogene family; S-phas	Multiple_Complex
TSUnmapped00000363.h	8.59	9.18	-1.5	0.0129	0.038	EIF3F	Eukaryotic translation initiation factor 3 subur	Coding
TC2000010002.hg.1	8.53	8.82	-1.23	0.0129	0.038	NCOA6	nuclear receptor coactivator 6	Multiple_Complex
TC0100014794.hg.1	7.21	7.81	-1.52	0.013	0.0381	ODF2L	outer dense fiber of sperm tails 2-like	Multiple_Complex
TC1700009032.hg.1	7.28	7.69	-1.33	0.013	0.0381	TNRC6C	trinucleotide repeat containing 6C	Multiple_Complex
TC2100008251.hg.1	4.46	4.19	1.21	0.013	0.0382	TFF1	trefoil factor 1	Multiple_Complex
TC1400008116.hg.1	7.05	6.29	1.7	0.013	0.0382	GSKIP	GSK3B interacting protein	Multiple_Complex
TC0600012428.hg.1	6.48	6.93	-1.36	0.0131	0.0383	UBE3D	ubiquitin protein ligase E3D	Multiple_Complex
TC1300008449.hg.1	5.73	5.2	1.44	0.0131	0.0383	CDX2	caudal type homeobox 2	Multiple_Complex
TC1200008005.hg.1	10.96	10.18	1.72	0.0131	0.0384	RASSF3	Ras association (RalGDS/AF-6) domain family	Multiple_Complex
TC0600007610.hg.1	4.95	5.47	-1.43	0.0131	0.0384	MSH5; MSH	mutS homolog 5; MSH5-SAPCD1 readthrough	Multiple_Complex
TSUnmapped00000395.h	7.15	6.8	1.27	0.0131	0.0384	ATG16L1	autophagy related 16-like 1	Coding
TC0100016709.hg.1	9.24	8.77	1.38	0.0131	0.0384	TPR	translocated promoter region, nuclear basket	Multiple_Complex
TC1400008762.hg.1	5.33	4.67	1.58	0.0131	0.0384	CTSG	cathepsin G	Multiple_Complex
TC0500010203.hg.1	5.15	4.66	1.41	0.0131	0.0384	MYO10	myosin X	Multiple_Complex
TC1900011665.hg.1	5.28	4.77	1.42	0.0131	0.0384	ZNF627	zinc finger protein 627	NonCoding
TC2000008351.hg.1	5.98	5.33	1.58	0.0131	0.0385	PLCB1	Memczak2013 ANTISENSE, coding, INTERNAL,	NonCoding
TC2000009128.hg.1	5.53	5.93	-1.31	0.0131	0.0385	ZHX3	zinc fingers and homeoboxes 3	Multiple_Complex
TC0400006798.hg.1	5.44	4.82	1.53	0.0131	0.0385	USP17L17	ubiquitin specific peptidase 17-like family mer	Coding
TC1100010271.hg.1	7.03	7.56	-1.44	0.0131	0.0385	UEVLD	UEV and lactate/malate dehyrogenase domair	Multiple_Complex
TC0600008071.hg.1	5.87	5.41	1.37	0.0132	0.0386	PTK7	protein tyrosine kinase 7 (inactive)	Multiple_Complex
TC0100010381.hg.1	7.48	6.93	1.47	0.0132	0.0386	UAP1	UDP-N-acetylglucosamine pyrophosphorylase	Multiple_Complex
TC0200015578.hg.1	8.03	7.35	1.6	0.0132	0.0386	IDH1	isocitrate dehydrogenase 1 (NADP+)	Multiple_Complex
TC0100011203.hg.1	9.26	10.08	-1.77	0.0132	0.0386	RNPEP	arginyl aminopeptidase (aminopeptidase B)	Multiple_Complex
TC1300008249.hg.1	4.43	4.02	1.33	0.0132	0.0386	GJB2	gap junction protein beta 2	Coding
TC1900011834.hg.1	7.94	8.37	-1.34	0.0132	0.0387	C19orf25	chromosome 19 open reading frame 25	Multiple_Complex
TC2100006883.hg.1	4.35	3.69	1.58	0.0132	0.0387	KRTAP20-3	keratin associated protein 20-3	Coding
TC1600010636.hg.1	5.16	4.85	1.24	0.0132	0.0387	AGRP	agouti related neuropeptide	Coding
TC0500009606.hg.1	7.36	7.79	-1.34	0.0132	0.0387	ZNF346	zinc finger protein 346	Multiple_Complex
TC0200016348.hg.1	5.4	4.54	1.83	0.0132	0.0387	HDLBP	Transcript Identified by AceView, Entrez Gene	Unassigned
TC1100006546.hg.1	5.46	4.94	1.43	0.0132	0.0388	TNNT3	troponin T type 3 (skeletal, fast)	Multiple_Complex
TC1600006789.hg.1	4.64	4.04	1.51	0.0132	0.0388	A2BP1	Transcript Identified by AceView, Entrez Gene	Unassigned
TC0900009417.hg.1	8.93	9.79	-1.82	0.0133	0.0388	RFX3	Memczak2013 ALT_ACCEPTOR, ALT_DONOR,	NonCoding
TC0400007096.hg.1	7.02	7.54	-1.43	0.0133	0.0388	ANAPC4	anaphase promoting complex subunit 4	Multiple_Complex
TC0200016309.hg.1	4.36	4.68	-1.25	0.0133	0.0388	OTOS	otospiralin	Coding
TC1600009883.hg.1	8.32	8.72	-1.31	0.0133	0.0388	TUFM; MIR	Tu translation elongation factor, mitochondria	Multiple_Complex
TC0200016000.hg.1	4.67	5.11	-1.36	0.0133	0.0389	GPR55	G protein-coupled receptor 55	Multiple_Complex
TC0800007846.hg.1	4.61	4.06	1.46	0.0133	0.0389	DNAJC5B	DnaJ (Hsp40) homolog, subfamily C, member	Multiple_Complex
TC0100006881.hg.1	13.98	14.72	-1.67	0.0133	0.0389	TNFRSF1B;	tumor necrosis factor receptor superfamily, m	Multiple_Complex
TC1100010187.hg.1	6.52	5.95	1.48	0.0133	0.0389	CALCA	calcitonin-related polypeptide alpha	Multiple_Complex
TC1300008552.hg.1	4.52	4	1.44	0.0133	0.0389	ZAR1L	zygote arrest 1-like	Coding
TC1200009705.hg.1	6.2	5.75	1.37	0.0133	0.0389	VWF	von Willebrand factor	Multiple_Complex
TC0200007155.hg.1	6.75	5.98	1.7	0.0133	0.039	EHD3	EH domain containing 3	Coding
TC0300013906.hg.1	4.43	4.15	1.22	0.0133	0.039	KCNMB2	potassium channel subfamily M regulatory be	Multiple_Complex
TC0200011753.hg.1	4.74	5.03	-1.22	0.0133	0.039	E2F6	E2F transcription factor 6	Multiple_Complex
TC1400006540.hg.1	13.97	12.32	3.14	0.0134	0.0391	RNASE2	ribonuclease, RNase A family, 2 (liver, eosinop	Coding
TC1700012287.hg.1	10.91	10.55	1.28	0.0134	0.0391	RPS6KB1	ribosomal protein S6 kinase, 70kDa, polypepti	Multiple_Complex
TC1400008677.hg.1	8.76	9.43	-1.59	0.0134	0.0392	SLC7A7	solute carrier family 7 (amino acid transporter	Multiple_Complex

TC1700009627.hg.1	4.97	4.68	1.22	0.0134	0.0392	YBX2	Y box binding protein 2	Multiple_Complex
TC0100009191.hg.1	6.58	7.35	-1.71	0.0134	0.0392	AGL	amylo-alpha-1, 6-glucosidase, 4-alpha-glucanc	Multiple_Complex
TC0200011548.hg.1	6.39	6.96	-1.48	0.0134	0.0392	TSSC1	tumor suppressing subtransferable candidate	Multiple_Complex
TC0700013467.hg.1	4.1	4.58	-1.4	0.0134	0.0392	ADCK2	aarF domain containing kinase 2	Multiple_Complex
TC0700010345.hg.1	4.5	4.05	1.37	0.0134	0.0392	ISPD	isoprenoid synthase domain containing	Multiple_Complex
TC2200008112.hg.1	6.45	6.99	-1.46	0.0134	0.0392	TOP3B	topoisomerase (DNA) III beta	Multiple_Complex
TC1700010433.hg.1	5.37	4.56	1.75	0.0135	0.0393	SLFN14	schlafen family member 14	Coding
TC0100018519.hg.1	11.52	10.96	1.48	0.0135	0.0393	F11R	F11 receptor	Multiple_Complex
TC1500010864.hg.1	5.26	5.85	-1.51	0.0135	0.0394	GATM	glycine amidinotransferase (L-arginine:glycine	Multiple_Complex
TC0200012074.hg.1	5.69	6.26	-1.49	0.0135	0.0394	EIF2B4	eukaryotic translation initiation factor 2B, sub	Multiple_Complex
TSUnmapped00000321.h	7.96	7.1	1.82	0.0135	0.0394	RPS25	ribosomal protein S25	Coding
TSUnmapped00000080.h	6.51	6.71	-1.15	0.0135	0.0394	ATG16L1	autophagy related 16-like 1	NonCoding
TC1200008685.hg.1	8.89	8.18	1.64	0.0135	0.0394	CHST11	Transcript Identified by AceView, Entrez Gene	Unassigned
TC2100007796.hg.1	5.14	5.61	-1.39	0.0135	0.0395	MRPL39	mitochondrial ribosomal protein L39	Multiple_Complex
TC0400008391.hg.1	5.59	5.1	1.41	0.0136	0.0395	EGF	epidermal growth factor	Multiple_Complex
TC0900010800.hg.1	4.99	4.49	1.42	0.0136	0.0395	ECM2	extracellular matrix protein 2, female organ ar	Coding
TC1000011081.hg.1	5.44	5.03	1.32	0.0136	0.0396	ZNF503	zinc finger protein 503	Multiple_Complex
TC0500012979.hg.1	9.44	9.84	-1.32	0.0136	0.0397	DDX41	DEAD (Asp-Glu-Ala-Asp) box polypeptide 41	Multiple_Complex
TC0100014612.hg.1	5.17	5.66	-1.41	0.0136	0.0397	CRYZ	crystallin zeta	Multiple_Complex
TC1200011002.hg.1	4.11	3.71	1.32	0.0136	0.0397	LRIG3	leucine-rich repeats and immunoglobulin-like	Multiple_Complex
TC0200016447.hg.1	5.59	5.94	-1.27	0.0136	0.0397	ACYP2	acylphosphatase 2, muscle type	Multiple_Complex
TC0700012159.hg.1	5.2	4.56	1.56	0.0137	0.0398	PUS7	pseudouridylate synthase 7 (putative)	Multiple_Complex
TC1800008399.hg.1	5.42	5.12	1.23	0.0137	0.0398	B4GALT6	UDP-Gal:betaGlcNAc beta 1,4- galactosyltrans	Multiple_Complex
TC0800011206.hg.1	14.34	12.51	3.53	0.0137	0.0398	RPL30	ribosomal protein L30	Multiple_Complex
TC0200009539.hg.1	14	12.82	2.27	0.0137	0.0398	ARHGAP15	Rho GTPase activating protein 15	Multiple_Complex
TC0700009082.hg.1	5.26	4.87	1.31	0.0137	0.0399	SMO	smoothened, frizzled class receptor	Multiple_Complex
TC1900011786.hg.1	8.39	8.08	1.24	0.0137	0.0399	NR1H2	nuclear receptor subfamily 1, group H, membe	Multiple_Complex
TC0800011395.hg.1	5.14	4.57	1.49	0.0137	0.0399	LRP12	LDL receptor related protein 12	Multiple_Complex
TC0X00010099.hg.1	6.75	7.47	-1.65	0.0137	0.0399	ABCB7	ATP binding cassette subfamily B member 7	Multiple_Complex
TC1900012050.hg.1	9.41	10.07	-1.59	0.0138	0.04	PPP6R1	protein phosphatase 6, regulatory subunit 1	Multiple_Complex
TC0X00010406.hg.1	4.57	4.16	1.33	0.0138	0.04	TCEAL8	transcription elongation factor A (SII)-like 8	Multiple_Complex
TC0400011650.hg.1	4.38	3.9	1.4	0.0138	0.04	ARSJ	arylsulfatase family, member J	Multiple_Complex
TC0800009468.hg.1	5.73	5.32	1.33	0.0138	0.0401	DEFB106B;	defensin, beta 106B; defensin, beta 106A	Coding
TC0500010999.hg.1	8.55	8.17	1.3	0.0138	0.0401	TAF9; AK6	TAF9 RNA polymerase II, TATA box binding pro	Multiple_Complex
TC1500009452.hg.1	7.49	6.89	1.51	0.0138	0.0401	GNB5	guanine nucleotide binding protein (G protein	Multiple_Complex
TC1900009311.hg.1	3.62	3.32	1.23	0.0138	0.0401	ZFR2	zinc finger RNA binding protein 2	Multiple_Complex
TC0100011004.hg.1	4.46	3.94	1.44	0.0138	0.0401	RGS21	regulator of G-protein signaling 21	Coding
TC0200007053.hg.1	7.27	7.71	-1.35	0.0138	0.0401	KHK	ketohexokinase	Multiple_Complex
TC0200013332.hg.1	7.85	8.29	-1.36	0.0138	0.0401	PLGLB1	Transcript Identified by AceView, Entrez Gene	Unassigned
TC0200007174.hg.1	9.63	8.89	1.66	0.0138	0.0402	SPAST	spastin	Multiple_Complex
TC0600006653.hg.1	8.27	8.69	-1.33	0.0138	0.0402	RIPK1	receptor (TNFRSF)-interacting serine-threonin	Multiple_Complex
TC0X00008830.hg.1	9.65	9.07	1.49	0.0139	0.0402	TAZ	tafazzin	Multiple_Complex
TC1400010574.hg.1	6.18	5.72	1.37	0.0139	0.0402	BCL2L2-PAI	BCL2L2-PABPN1 readthrough	Multiple_Complex
TC0500011435.hg.1	8.79	9.44	-1.57	0.0139	0.0403	LYSMD3	LysM, putative peptidoglycan-binding, domair	Multiple_Complex
TC1900010783.hg.1	6.93	6.54	1.31	0.0139	0.0403	GRIK5	glutamate receptor, ionotropic, kainate 5	Multiple_Complex
TC0100009181.hg.1	3.77	3.52	1.19	0.0139	0.0403	PLPPR4	phospholipid phosphatase related 4	Multiple_Complex
TC1100013225.hg.1	3.62	3.36	1.19	0.0139	0.0403	POU2AF1	POU class 2 associating factor 1	Multiple_Complex
TC1200007653.hg.1	6.42	5.98	1.35	0.0139	0.0403	NR4A1	nuclear receptor subfamily 4, group A, membe	Multiple_Complex
TC0200009973.hg.1	8.18	9	-1.76	0.0139	0.0404	ITGA6	integrin alpha 6	Multiple_Complex
TC2000009953.hg.1	12.9	12.5	1.32	0.0139	0.0404	OSBPL2	oxysterol binding protein-like 2	Multiple_Complex
TC0500013149.hg.1	6.47	6.91	-1.35	0.0139	0.0404	SRD5A1	steroid-5-alpha-reductase, alpha polypeptide	Multiple_Complex
TC0600009886.hg.1	3.94	3.35	1.51	0.0139	0.0404	VIP	vasoactive intestinal peptide	Coding
TC2200006678.hg.1	9.24	10.03	-1.72	0.014	0.0405	SNAP29	synaptosome associated protein 29kDa	Multiple_Complex
TC0X00010141.hg.1	8.75	9.53	-1.72	0.014	0.0405	CYSLTR1	cysteinyl leukotriene receptor 1	Multiple_Complex
TC1000009155.hg.1	5.24	4.84	1.32	0.014	0.0405	DMBT1	deleted in malignant brain tumors 1	Multiple_Complex
TC0100010858.hg.1	9.46	8.93	1.44	0.014	0.0405	DHX9	DEAH (Asp-Glu-Ala-His) box helicase 9	Multiple_Complex
TC1900007809.hg.1	9.35	9.81	-1.38	0.014	0.0405	UBA2	ubiquitin-like modifier activating enzyme 2	Multiple_Complex
TC0600010343.hg.1	8.63	8.99	-1.29	0.014	0.0406	TBP	TATA box binding protein	Multiple_Complex
TC0100018479.hg.1	12.58	11.94	1.56	0.0141	0.0408	NOTCH2NL	notch 2 N-terminal like	Multiple_Complex
TC0600011304.hg.1	4.94	4.6	1.27	0.0141	0.0408	OR12D3	olfactory receptor, family 12, subfamily D, me	Coding
TC1600010955.hg.1	4.57	4.07	1.42	0.0141	0.0408	MAFTRR	MAF transcriptional regulator RNA	Multiple_Complex
TC0300010310.hg.1	14.37	13.06	2.47	0.0141	0.0408	RPL32; SNC	ribosomal protein L32; small nucleolar RNA, H	Multiple_Complex
TC0800009833.hg.1	4.8	4.47	1.26	0.0141	0.0408	PHYHIP	phytanoyl-CoA 2-hydroxylase interacting prote	Multiple_Complex
TC2000008666.hg.1	8.28	7.95	1.26	0.0141	0.0408	NAPB	N-ethylmaleimide-sensitive factor attachment	Multiple_Complex
TC0900010667.hg.1	4.23	3.77	1.38	0.0141	0.0408	SPATA31C2	SPATA31 subfamily C, member 2	Multiple_Complex
TC0900006782.hg.1	7.12	7.51	-1.3	0.0141	0.0408	FOCAD	focadhesin	Multiple_Complex
TC0400007785.hg.1	4.09	3.76	1.26	0.0141	0.0409	ENAM	enamelin	Multiple_Complex
TC1500009224.hg.1	4.13	3.69	1.36	0.0142	0.041	STRC	stereocilin	Multiple_Complex
TC0600009772.hg.1	4.76	5.12	-1.28	0.0142	0.041	SASH1	SAM and SH3 domain containing 1	Multiple_Complex
TSUnmapped00000248.h	4.72	4.49	1.17	0.0142	0.0411	BCL2L14	BCL2-like 14 (apoptosis facilitator)	Coding

TC0300013736.hg.1	9.9	10.43	-1.45	0.0142	0.0411	DLG1	discs, large homolog 1 (Drosophila)	Multiple_Complex
TC1900011762.hg.1	7.88	7.48	1.32	0.0142	0.0411	APOC2	apolipoprotein C-II	Multiple_Complex
TC1900011253.hg.1	9.65	9.93	-1.21	0.0142	0.0411	C19orf84	chromosome 19 open reading frame 84	Multiple_Complex
TC1400007204.hg.1	6.96	6.48	1.4	0.0142	0.0412	CGRRF1	cell growth regulator with ring finger domain	Multiple_Complex
TC1900009196.hg.1	6.3	5.93	1.29	0.0142	0.0412	CSNK1G2-A	CSNK1G2 antisense RNA 1	Multiple_Complex
TC0900008415.hg.1	4.5	4.07	1.35	0.0142	0.0412	MUSK	muscle, skeletal, receptor tyrosine kinase	Coding
TC0100016567.hg.1	7.61	7	1.52	0.0143	0.0412	TOR1AIP2	torsin A interacting protein 2	Multiple_Complex
TC1200007161.hg.1	7.73	7.34	1.31	0.0143	0.0412	MRPS35	mitochondrial ribosomal protein S35	Multiple_Complex
TC0200009312.hg.1	4.56	4.14	1.34	0.0143	0.0413	POTEJ	POTE ankyrin domain family, member J	Multiple_Complex
TC1100010726.hg.1	6.22	6.86	-1.56	0.0143	0.0413	PACSIN3	protein kinase C and casein kinase substrate ir	Multiple_Complex
TC1200006627.hg.1	5.83	6.44	-1.53	0.0143	0.0413	NCAPD2	non-SMC condensin I complex subunit D2	Multiple_Complex
TC0600014085.hg.1	6.61	6.08	1.44	0.0143	0.0414	HIST1H2AH	histone cluster 1, H2ah	Coding
TC0200016771.hg.1	5.6	5.13	1.39	0.0143	0.0414	TNS1	tensin 1	NonCoding
TC0900011820.hg.1	5.79	5.88	-1.06	0.0143	0.0414	TSC1	Transcript Identified by AceView, Entrez Gene	Unassigned
TC0600010962.hg.1	13.99	13.29	1.62	0.0144	0.0416	DEK	DEK proto-oncogene	Multiple_Complex
TC1100011183.hg.1	6.95	6.49	1.38	0.0144	0.0416	SF1	splicing factor 1	Multiple_Complex
TC0500013225.hg.1	6.28	6.88	-1.52	0.0144	0.0416	CDC42SE2	CDC42 small effector 2	NonCoding
TC1400009002.hg.1	8.98	8.37	1.52	0.0144	0.0416	SEC23A	Sec23 homolog A, COPII coat complex compor	Multiple_Complex
TC0100011262.hg.1	4.6	4.27	1.25	0.0144	0.0416	PRELP	proline/arginine-rich end leucine-rich repeat p	Coding
TC2000008802.hg.1	5.64	5.21	1.35	0.0144	0.0417	DEFB116	defensin, beta 116	Coding
TC1100007637.hg.1	5.47	4.93	1.45	0.0145	0.0418	OR8U1; OR	olfactory receptor, family 8, subfamily U, men	Coding
TC0900009424.hg.1	3.72	3.6	1.09	0.0145	0.0418	GLIS3	GLIS family zinc finger 3	Multiple_Complex
TC1900011812.hg.1	5.29	5.76	-1.38	0.0145	0.0418	ZNF547; TR	zinc finger protein 547; trafficking protein par	Multiple_Complex
TC1500010745.hg.1	4.99	4.62	1.29	0.0145	0.0419	POLR2M	polymerase (RNA) II (DNA directed) polypeptic	NonCoding
TC1400008616.hg.1	3.71	3.44	1.21	0.0145	0.0419	RNASE13	ribonuclease, RNase A family, 13 (non-active)	Coding
TC1100009906.hg.1	4.84	4.37	1.38	0.0145	0.0419	OR52E2	olfactory receptor, family 52, subfamily E, mer	Coding
TC2000008379.hg.1	5.06	5.64	-1.5	0.0145	0.0419	MKKS	McKusick-Kaufman syndrome	Multiple_Complex
TC0800010275.hg.1	8.61	8.44	1.12	0.0145	0.0419	PLAT	plasminogen activator, tissue	Multiple_Complex
TC1700006775.hg.1	8.16	8.67	-1.42	0.0145	0.0419	CHD3	chromodomain helicase DNA binding protein :	Multiple_Complex
TC0100012948.hg.1	3.68	3.26	1.34	0.0146	0.0419	PRAMEF19	PRAME family member 19	Coding
TC0800009231.hg.1	10.85	9.83	2.02	0.0146	0.042	GRINA	glutamate receptor, ionotropic, N-methyl D-as	Multiple_Complex
TC1700009619.hg.1	6.89	6.49	1.31	0.0146	0.0421	DLG4	discs, large homolog 4 (Drosophila)	Multiple_Complex
TC1200008792.hg.1	4.93	5.5	-1.48	0.0146	0.0421	UNG	uracil DNA glycosylase	Multiple_Complex
TC1700011189.hg.1	5.87	5.39	1.39	0.0146	0.0421	TMEM100	transmembrane protein 100	Multiple_Complex
TC0700009521.hg.1	3.84	3.5	1.26	0.0146	0.0421	OR2A12	olfactory receptor, family 2, subfamily A, mem	Coding
TC1200008814.hg.1	12.16	11.45	1.64	0.0146	0.0421	ANKRD13A	ankyrin repeat domain 13A	Multiple_Complex
TC0300011706.hg.1	4.61	4.02	1.51	0.0146	0.0421	VGLL3	vestigial-like family member 3	Multiple_Complex
TC0100018301.hg.1	5.97	5.58	1.31	0.0146	0.0421	DCST1	DC-STAMP domain containing 1	Multiple_Complex
TC1900011929.hg.1	4.87	5.54	-1.59	0.0147	0.0422	IGFLR1	IGF like family receptor 1	Multiple_Complex
TC0500012394.hg.1	4.19	3.53	1.58	0.0147	0.0422	C5orf46	chromosome 5 open reading frame 46	Multiple_Complex
TC1900007783.hg.1	7.49	6.86	1.55	0.0147	0.0422	CHST8	carbohydrate (N-acetylgalactosamine 4-0) sul	Multiple_Complex
TC0600007869.hg.1	6.81	7.6	-1.73	0.0147	0.0422	CMTR1	cap methyltransferase 1	Multiple_Complex
TC0200009316.hg.1	5.72	5.35	1.29	0.0147	0.0423	GPR148	G protein-coupled receptor 148	Coding
TC1500009348.hg.1	6.19	5.52	1.6	0.0147	0.0423	FBN1	fibrillin 1	Multiple_Complex
TC0700012886.hg.1	4.8	4.5	1.23	0.0147	0.0423	EPHA1	EPH receptor A1	Multiple_Complex
TC1300008048.hg.1	4.95	4.52	1.35	0.0147	0.0423	TEX29	testis expressed 29	Coding
TC0X00008509.hg.1	6.26	6.64	-1.3	0.0147	0.0423	ZNF449	zinc finger protein 449	Coding
TC1700008907.hg.1	5.67	6.1	-1.35	0.0148	0.0424	TMEM94; N	transmembrane protein 94; microRNA 6785	Multiple_Complex
TC0300013959.hg.1	7.68	7.02	1.59	0.0148	0.0424	CCDC13	coiled-coil domain containing 13	Multiple_Complex
TC0700008882.hg.1	6.1	5.45	1.57	0.0148	0.0424	ST7; ST7-O	suppression of tumorigenicity 7; ST7 overlapp	Multiple_Complex
TC1100007457.hg.1	8.45	7.72	1.66	0.0148	0.0424	ATG13	autophagy related 13	Multiple_Complex
TC0600014238.hg.1	5.79	6.15	-1.29	0.0148	0.0425	BLOC1S5	biogenesis of lysosomal organelles complex-1,	Multiple_Complex
TC2000009555.hg.1	5.3	4.77	1.45	0.0148	0.0425	GCNT7	glucosaminyl (N-acetyl) transferase family me	Coding
TC0100008696.hg.1	4.11	4.42	-1.24	0.0148	0.0426	HHLA3	HERV-H LTR-associating 3	Multiple_Complex
TC0200016252.hg.1	9.47	9.93	-1.37	0.0148	0.0426	HDAC4	histone deacetylase 4	Multiple_Complex
TC1000011427.hg.1	6.03	6.66	-1.55	0.0148	0.0426	IDE	insulin-degrading enzyme	Multiple_Complex
TC1100011367.hg.1	9.56	10.48	-1.89	0.0149	0.0426	UNC93B1	unc-93 homolog B1 (C. elegans)	Multiple_Complex
TC0400010048.hg.1	6	6.35	-1.27	0.0149	0.0427	ZNF518B	zinc finger protein 518B	Multiple_Complex
TC1900006492.hg.1	5.48	5.16	1.25	0.0149	0.0427	PRTN3	proteinase 3	Multiple_Complex
TC0700007157.hg.1	5.44	5.1	1.26	0.0149	0.0427	NPSR1	neuropeptide S receptor 1	Multiple_Complex
TC0600008505.hg.1	4.62	4.27	1.27	0.0149	0.0428	RIMS1	regulating synaptic membrane exocytosis 1	Multiple_Complex
TC0100006548.hg.1	3.73	3.56	1.12	0.0149	0.0428	GABRD	gamma-aminobutyric acid (GABA) A receptor,	Coding
TC2000007114.hg.1	7.03	6.56	1.38	0.0149	0.0428	KIF3B	kinesin family member 3B	Multiple_Complex
TC1900011658.hg.1	8.75	8.01	1.68	0.0149	0.0428	TRAPPC5	trafficking protein particle complex 5	Multiple_Complex
TC0600013330.hg.1	3.75	3.33	1.34	0.0149	0.0429	PBOV1	prostate and breast cancer overexpressed 1	Coding
TC1900011826.hg.1	4.56	4.94	-1.31	0.015	0.0429	ZNF544	zinc finger protein 544	Multiple_Complex
TC1700010698.hg.1	8.05	7.54	1.43	0.015	0.0429	CNP	Memczak2013 ANTISENSE, CDS, coding, INTEF	NonCoding
TC1200010650.hg.1	6.39	6.8	-1.32	0.015	0.0429	RACGAP1	Rac GTPase activating protein 1	Multiple_Complex
TC0500012592.hg.1	3.31	3.14	1.13	0.015	0.0429	FAM71B	family with sequence similarity 71, member B	Coding
TC0600006659.hg.1	3.85	4.34	-1.41	0.015	0.043	BPHL	biphenyl hydrolase-like (serine hydrolase)	Multiple_Complex

TC0100010581.hg.1	5	4.59	1.33	0.015	0.0431	FMO6P	flavin containing monooxygenase 6 pseudoge	Multiple_Complex
TC0100010365.hg.1	7.92	8.53	-1.53	0.0151	0.0432	FCRLA	Fc receptor-like A	Multiple_Complex
TC0300011047.hg.1	7.17	7.73	-1.48	0.0151	0.0432	IMPDH2	IMP (inosine 5-monophosphate) dehydrogena	Multiple_Complex
TC1200007783.hg.1	4.52	3.97	1.46	0.0151	0.0433	OR6C74	olfactory receptor, family 6, subfamily C, mem	Coding
TC1100012474.hg.1	7.22	7.91	-1.61	0.0151	0.0433	MPZL2	myelin protein zero-like 2	Multiple_Complex
TSUnmapped00000660.h	4.62	4.24	1.3	0.0151	0.0433	PRAMEF18	PRAME family member 18	Coding
TC1400008568.hg.1	4.18	3.74	1.35	0.0151	0.0433	OR11H2	olfactory receptor, family 11, subfamily H, me	Coding
TC1900010494.hg.1	5.43	5.11	1.25	0.0152	0.0435	HSPB6	heat shock protein, alpha-crystallin-related, B	Multiple_Complex
TC1900006923.hg.1	3.58	3.37	1.16	0.0152	0.0435	OR1M1	olfactory receptor, family 1, subfamily M, mer	Coding
TC1200012572.hg.1	6.05	6.6	-1.47	0.0152	0.0435	ITFG2	integrin alpha FG-GAP repeat containing 2	NonCoding
TC1100009667.hg.1	4.69	5.07	-1.3	0.0152	0.0435	ANO9	anoctamin 9	Multiple_Complex
TC0900012175.hg.1	11.43	11	1.35	0.0153	0.0436	CDK9	cyclin-dependent kinase 9	Multiple_Complex
TC1600007130.hg.1	3.85	3.54	1.24	0.0153	0.0437	ACSM5	acyl-CoA synthetase medium-chain family me	Multiple_Complex
TC1000012566.hg.1	6.19	6.63	-1.36	0.0153	0.0437	MRPS16	mitochondrial ribosomal protein S16	Multiple_Complex
TC1200009791.hg.1	4.12	3.7	1.33	0.0153	0.0437	GDF3	growth differentiation factor 3	Coding
TC1200011885.hg.1	7.03	6.62	1.33	0.0153	0.0438	GLTP	glycolipid transfer protein	Coding
TC1200008771.hg.1	10.45	9.96	1.4	0.0153	0.0439	ISCU	iron-sulfur cluster assembly enzyme	Multiple_Complex
TC0100013281.hg.1	9.21	8.51	1.62	0.0153	0.0439	ASAP3	ArfGAP with SH3 domain, ankyrin repeat and	Multiple_Complex
TC0200007609.hg.1	8.53	9.13	-1.52	0.0154	0.0439	SPTBN1	spectrin, beta, non-erythrocytic 1	Multiple_Complex
TC0900007094.hg.1	5.59	5.25	1.27	0.0154	0.0439	CA9	carbonic anhydrase IX	Multiple_Complex
TC1200012149.hg.1	5.94	6.52	-1.49	0.0154	0.0439	GCN1; MIR	GCN1 eIF2 alpha kinase activator homolog; mi	Multiple_Complex
TC0400012873.hg.1	5.55	5.07	1.39	0.0154	0.044	FAM149A	family with sequence similarity 149, member	Multiple_Complex
TC1500009405.hg.1	5.01	4.67	1.27	0.0154	0.044	CYP19A1	cytochrome P450, family 19, subfamily A, poly	Multiple_Complex
TC0100018473.hg.1	8.3	7.9	1.31	0.0154	0.044	SEC22B	Salzman2013 ALT_ACCEPTOR, CDS, coding, IN	Multiple_Complex
TC1200012424.hg.1	4.62	4.13	1.4	0.0154	0.044	TMEM132I	transmembrane protein 132D	Multiple_Complex
TC1100007537.hg.1	5.14	4.49	1.57	0.0154	0.0441	TRIM49B	tripartite motif containing 49B	Coding
TC0600011953.hg.1	5.67	6.84	-2.24	0.0154	0.0441	PLA2G7	phospholipase A2, group VII (platelet-activatir	Multiple_Complex
TSUnmapped00000263.h	3.76	4.22	-1.38	0.0155	0.0442	BCL2L14	BCL2-like 14 (apoptosis facilitator)	NonCoding
TC1500010877.hg.1	6.31	7.03	-1.65	0.0155	0.0442	PARP16	poly(ADP-ribose) polymerase family member	Multiple_Complex
TC0100016086.hg.1	8.94	8.37	1.49	0.0155	0.0442	AIM2	absent in melanoma 2	Multiple_Complex
TC2200007464.hg.1	5.79	6.21	-1.34	0.0155	0.0442	L3MBTL2	l(3)mbt-like 2 (Drosophila)	Multiple_Complex
TC1700008677.hg.1	3.6	3.57	1.02	0.0155	0.0443	CACNG4	calcium channel, voltage-dependent, gamma	Coding
TC1900009808.hg.1	5.24	5.65	-1.33	0.0155	0.0443	ADGRL1	adhesion G protein-coupled receptor L1	Multiple_Complex
TC0Y00007327.hg.1	3.81	3.44	1.29	0.0155	0.0443	TSPY10	testis specific protein, Y-linked 10	Coding
TC0700010621.hg.1	5.93	5.61	1.24	0.0155	0.0443	FKBP14	FK506 binding protein 14	Multiple_Complex
TC0100009056.hg.1	6.2	5.47	1.66	0.0156	0.0444	CCDC18	coiled-coil domain containing 18	Multiple_Complex
TC0100016071.hg.1	4.72	4.09	1.55	0.0156	0.0444	OR6Y1	olfactory receptor, family 6, subfamily Y, mem	Coding
TC2200007662.hg.1	4.29	4.69	-1.32	0.0156	0.0444	GTSE1	G-2 and S-phase expressed 1	Multiple_Complex
TC0200011673.hg.1	5.85	5.14	1.64	0.0156	0.0444	MBOAT2	Memczak2013 ALT_ACCEPTOR, ALT_DONOR,	NonCoding
TC0500008846.hg.1	5.09	5.89	-1.74	0.0156	0.0444	IGIP	IgA-inducing protein	Coding
TC1900011919.hg.1	8.43	9.1	-1.59	0.0156	0.0445	ZNF708	zinc finger protein 708	Multiple_Complex
TC1700009040.hg.1	9.23	10.06	-1.77	0.0156	0.0445	SYNGR2	synaptogyrin 2	Multiple_Complex
TC0X00009128.hg.1	3.93	3.57	1.29	0.0156	0.0445	ACE2	angiotensin I converting enzyme 2	Multiple_Complex
TC1600007934.hg.1	4.07	4.51	-1.36	0.0156	0.0446	CAPNS2	calpain, small subunit 2	Coding
TC1600007465.hg.1	6.01	5.84	1.12	0.0157	0.0447	ZNF48	zinc finger protein 48	Multiple_Complex
TC1100007274.hg.1	4.74	5.5	-1.69	0.0157	0.0447	CD44	Memczak2013 ALT_ACCEPTOR, ALT_DONOR,	NonCoding
TC1500008053.hg.1	4.53	3.82	1.63	0.0157	0.0448	CFAP161	cilia and flagella associated protein 161	Multiple_Complex
TC0500006523.hg.1	9.05	9.54	-1.4	0.0157	0.0448	NDUFS6	NADH dehydrogenase (ubiquinone) Fe-S prote	Multiple_Complex
TC1100009950.hg.1	4.23	3.87	1.28	0.0158	0.0448	OR56A5	olfactory receptor, family 56, subfamily A, me	Coding
TC1400010331.hg.1	4.66	5.02	-1.28	0.0158	0.0449	XRCC3	X-ray repair complementing defective repair i	Multiple_Complex
TC1400010640.hg.1	5.06	5.66	-1.51	0.0158	0.0449	TMEM251	transmembrane protein 251	Coding
TC2000009014.hg.1	9.94	10.25	-1.24	0.0158	0.0449	NDRG3	NDRG family member 3	Multiple_Complex
TC0900008010.hg.1	5.54	5.85	-1.24	0.0158	0.045	PTPDC1	protein tyrosine phosphatase domain contain	Multiple_Complex
TC1200007861.hg.1	9.47	8.33	2.2	0.0158	0.045	LRP1	LDL receptor related protein 1	Multiple_Complex
TC1000010129.hg.1	4.67	4.2	1.39	0.0158	0.0451	PTCHD3	patched domain containing 3	Coding
TC0800011312.hg.1	8.96	9.5	-1.46	0.0159	0.0451	RRM2B	ribonucleotide reductase M2 B (TP53 inducibl	Multiple_Complex
TC0X00007238.hg.1	4.64	4.17	1.39	0.0159	0.0451	PAGE4	P antigen family, member 4 (prostate associat	Multiple_Complex
TC0300008506.hg.1	4.14	4.5	-1.28	0.0159	0.0451	LRRC58	Memczak2013 ANTISENSE, CDS, coding, INTEF	NonCoding
TC1900008276.hg.1	4.44	4.21	1.17	0.0159	0.0451	CEACAM16	carcinoembryonic antigen-related cell adhesic	Multiple_Complex
TC1900008968.hg.1	5.17	5.56	-1.31	0.0159	0.0452	ZNF71	zinc finger protein 71	Multiple_Complex
TC0800012202.hg.1	7.52	8.05	-1.44	0.0159	0.0452	BOP1; MIR	block of proliferation 1; microRNA 7112	Multiple_Complex
TC0100018112.hg.1	5.25	4.74	1.43	0.0159	0.0452	OR11L1	olfactory receptor, family 11, subfamily L, mer	Coding
TC0500008055.hg.1	4.32	3.85	1.39	0.0159	0.0453	ADGRV1	adhesion G protein-coupled receptor V1	Multiple_Complex
TC0700010472.hg.1	11.75	11.38	1.29	0.016	0.0453	TRA2A	transformer 2 alpha homolog (Drosophila)	Multiple_Complex
TC0600014153.hg.1	3.71	4.21	-1.42	0.016	0.0453	CFAP206	cilia and flagella associated protein 206	Multiple_Complex
TC1100011035.hg.1	8.84	9.52	-1.61	0.016	0.0453	CPSF7	cleavage and polyadenylation specific factor 7	Multiple_Complex
TC1500010894.hg.1	5.73	6.19	-1.38	0.016	0.0454	SNUPN	snurportin 1	Multiple_Complex
TC0100010924.hg.1	7.2	6.52	1.6	0.016	0.0454	C1orf27	chromosome 1 open reading frame 27	Multiple_Complex
TC1100012810.hg.1	6.18	5.6	1.5	0.016	0.0454	NFRKB	Transcript Identified by AceView, Entrez Gene	Unassigned
TC0600007412.hg.1	3.94	3.67	1.21	0.016	0.0454	NKAPL	NFKB activating protein-like	Coding

TC0100011746.hg.1	10.03	9.56	1.39	0.016	0.0454	CNIH4	cornichon family AMPA receptor auxiliary prot	Multiple_Complex
TC1100008669.hg.1	4.4	4.9	-1.42	0.016	0.0454	PRSS23	protease, serine, 23	Multiple_Complex
TC0200016402.hg.1	8.19	12.94	-27	0.016	0.0454	RSAD2	radical S-adenosyl methionine domain contair	Multiple_Complex
TC0100014408.hg.1	5.92	6.46	-1.45	0.016	0.0454	DOCK7	dedicator of cytokinesis 7	Multiple_Complex
TC1100006986.hg.1	3.9	3.5	1.32	0.016	0.0455	MRGPRX3	MAS-related GPR, member X3	Coding
TC0500009417.hg.1	4.03	3.74	1.23	0.016	0.0455	TLX3	T-cell leukemia homeobox 3	Coding
TC1900011932.hg.1	6.35	6.72	-1.29	0.016	0.0455	ALKBH6	alkB homolog 6	Multiple_Complex
TC1700012258.hg.1	5.7	5.31	1.31	0.016	0.0455	KRTAP9-8	keratin associated protein 9-8	Coding
TC0400008813.hg.1	6.62	6.23	1.31	0.0161	0.0455	MGST2	microsomal glutathione S-transferase 2	Multiple_Complex
TC0600011914.hg.1	4.11	3.84	1.21	0.0161	0.0455	TCTE1	t-complex-associated-testis-expressed 1	Coding
TC1900007386.hg.1	5.07	5.32	-1.2	0.0161	0.0456	SSBP4	single stranded DNA binding protein 4	Multiple_Complex
TC0100012921.hg.1	7.41	7.8	-1.31	0.0161	0.0456	DHRS3; MII	dehydrogenase/reductase (SDR family) memb	Multiple_Complex
TC0900009073.hg.1	10.47	11.37	-1.86	0.0161	0.0457	RPL7A; SNC	ribosomal protein L7a; small nucleolar RNA, C	Multiple_Complex
TC0400012606.hg.1	10.49	10.82	-1.26	0.0161	0.0457	IRF2	interferon regulatory factor 2	Multiple_Complex
TC0X00008860.hg.1	11.41	11.92	-1.42	0.0161	0.0457	F8A2; F8A3	coagulation factor VIII-associated 2; coagulati	Coding
TC0400006491.hg.1	6.09	6.47	-1.29	0.0161	0.0457	IDUA	iduronidase, alpha-L-	Multiple_Complex
TC1100006726.hg.1	6.21	6	1.16	0.0161	0.0457	SMPD1	sphingomyelin phosphodiesterase 1, acid lyso	Multiple_Complex
TC1400007441.hg.1	4.87	5.62	-1.69	0.0161	0.0457	AKAP5	A kinase (PRKA) anchor protein 5	Coding
TC1000011072.hg.1	4.21	3.9	1.24	0.0162	0.0458	DUSP13	dual specificity phosphatase 13	Multiple_Complex
TC0X00009964.hg.1	6.74	7.17	-1.35	0.0162	0.0458	PDZD11	PDZ domain containing 11	Multiple_Complex
TC0600007695.hg.1	7.77	7.45	1.25	0.0162	0.0458	PFDN6; MII	prefoldin subunit 6; microRNA 6834	Multiple_Complex
TC0200008270.hg.1	5.27	4.93	1.26	0.0162	0.0458	ATOH8	atonal bHLH transcription factor 8	Multiple_Complex
TC0400008195.hg.1	3.94	3.52	1.34	0.0162	0.0459	PDHA2	pyruvate dehydrogenase (lipoamide) alpha 2	Coding
TC1100006509.hg.1	6.11	5.72	1.31	0.0162	0.0459	MUC5B	mucin 5B, oligomeric mucus/gel-forming	Multiple_Complex
TC1300008084.hg.1	9.09	8.16	1.9	0.0162	0.0459	ATP11A	ATPase, class VI, type 11A	Multiple_Complex
TC0600007568.hg.1	6.87	7.58	-1.64	0.0162	0.046	HCG27	HLA complex group 27 (non-protein coding)	Multiple_Complex
TC1900006525.hg.1	6.86	7.28	-1.34	0.0163	0.046	C19orf24	chromosome 19 open reading frame 24	Multiple_Complex
TC2200007337.hg.1	7.24	6.98	1.2	0.0163	0.046	MAFF	v-maf avian musculoaponeurotic fibrosarcoma	Multiple_Complex
TC0700009243.hg.1	11.34	12.07	-1.67	0.0163	0.0461	TMEM140	transmembrane protein 140	Multiple_Complex
TC1100013171.hg.1	6.82	7.06	-1.18	0.0163	0.0461	TUT1	terminal uridylyl transferase 1, U6 snRNA-spe	Multiple_Complex
TC0500008664.hg.1	7.21	7.78	-1.48	0.0163	0.0461	HSPA4	heat shock 70kDa protein 4	Multiple_Complex
TC1200009921.hg.1	4.34	4.9	-1.47	0.0163	0.0461	TAS2R13	taste receptor, type 2, member 13	Coding
TC1700008603.hg.1	8.31	7.79	1.43	0.0163	0.0461	CEP95	centrosomal protein 95kDa	Multiple_Complex
TC0800011826.hg.1	14.02	12.93	2.12	0.0164	0.0462	ASAP1; ASA	ArfGAP with SH3 domain, ankyrin repeat and	Multiple_Complex
TC0300006513.hg.1	4.52	4.17	1.28	0.0164	0.0462	GRM7	glutamate receptor, metabotropic 7	Multiple_Complex
TC1600011405.hg.1	4.36	4.78	-1.34	0.0164	0.0463	GINS3	GINS complex subunit 3 (Psf3 homolog)	Multiple_Complex
TC1400008973.hg.1	4.44	4.11	1.26	0.0164	0.0463	SLC25A21	solute carrier family 25 (mitochondrial oxoadi	Multiple_Complex
TC0100018208.hg.1	5.44	5.19	1.19	0.0164	0.0463	ZBTB8B	zinc finger and BTB domain containing 8B	Coding
TC0100010280.hg.1	3.56	3.36	1.15	0.0164	0.0463	KCNJ9	potassium channel, inwardly rectifying subfan	Multiple_Complex
TC0300012338.hg.1	3.9	3.56	1.27	0.0164	0.0463	DNAJB8	DnaJ (Hsp40) homolog, subfamily B, member	Coding
TC0500010628.hg.1	7.23	7.53	-1.23	0.0164	0.0464	ANXA2R	annexin A2 receptor	Coding
TC0800012311.hg.1	6.2	5.86	1.26	0.0164	0.0464	SGK3	serum/glucocorticoid regulated kinase family,	NonCoding
TC1900008946.hg.1	6.8	6.36	1.36	0.0164	0.0464	GALP	galanin-like peptide	Coding
TC0400011202.hg.1	8.55	8.81	-1.19	0.0164	0.0464	COQ2	coenzyme Q2 4-hydroxybenzoate polyprenylt	Multiple_Complex
TC0400007026.hg.1	4.6	4.17	1.35	0.0164	0.0464	RP11-362J1	novel transcript, antisense to KCNIP4; Transcr	NonCoding
TC1500007093.hg.1	8.91	8.34	1.48	0.0164	0.0464	EIF3J	eukaryotic translation initiation factor 3, subu	Multiple_Complex
TC1900011233.hg.1	4.17	3.82	1.27	0.0165	0.0465	KLK10	kallikrein related peptidase 10	Multiple_Complex
TC0X00011332.hg.1	4.47	4.06	1.33	0.0165	0.0465	PLXNB3	plexin B3	Multiple_Complex
TC1100012651.hg.1	4.66	4.36	1.23	0.0165	0.0465	OR10G7	olfactory receptor, family 10, subfamily G, me	Coding
TSUnmapped00000298.h	3.95	3.56	1.31	0.0165	0.0467	TGM4	transglutaminase 4	NonCoding
TC0600010383.hg.1	4.72	5.16	-1.36	0.0165	0.0467	HUS1B	HUS1 checkpoint clamp component B	Coding
TC1900010478.hg.1	5.29	4.86	1.35	0.0166	0.0467	KRTDAP	keratinocyte differentiation-associated protei	Multiple_Complex
TC0900011846.hg.1	7.91	8.48	-1.48	0.0166	0.0467	REXO4	REX4 homolog, 3-5 exonuclease	Multiple_Complex
TC1400010621.hg.1	10.05	9.25	1.74	0.0166	0.0467	CHURC1	churchill domain containing 1	Multiple_Complex
TC0X00008276.hg.1	4.22	3.76	1.37	0.0166	0.0468	ATP1B4	ATPase, Na+/K+ transporting, beta 4 polypept	Coding
TC0700008659.hg.1	6.71	6.06	1.58	0.0166	0.0468	NFE4	nuclear factor, erythroid 4	Multiple_Complex
TC1100009110.hg.1	4.29	3.99	1.23	0.0166	0.0468	NNMT	nicotinamide N-methyltransferase	Multiple_Complex
TC0800011861.hg.1	7.92	6.77	2.22	0.0166	0.0468	LRRC6	leucine rich repeat containing 6	Multiple_Complex
TC0100015876.hg.1	7.3	6.5	1.74	0.0166	0.0468	ILF2	Jeck2013 ALT_DONOR, coding, INTERNAL, intr	NonCoding
TC0800011764.hg.1	5.49	5.82	-1.26	0.0167	0.0469	FAM84B	family with sequence similarity 84, member B	Multiple_Complex
TC0500009987.hg.1	9.14	9.71	-1.49	0.0167	0.0469	MED10	mediator complex subunit 10	Multiple_Complex
TC1700008085.hg.1	7.6	7.33	1.2	0.0167	0.0469	STH	saitohin	Coding
TC1200007615.hg.1	13.06	13.5	-1.35	0.0167	0.0469	DIP2B	disco-interacting protein 2 homolog B	Multiple_Complex
TC1100009983.hg.1	6.29	5.92	1.29	0.0167	0.0469	MRPL17	mitochondrial ribosomal protein L17	Multiple_Complex
TC0500011100.hg.1	5.29	5.65	-1.29	0.0167	0.047	ZNF366	zinc finger protein 366	Multiple_Complex
TC1700008426.hg.1	3.89	3.62	1.2	0.0167	0.047	LPO	lactoperoxidase	Multiple_Complex
TC1900011742.hg.1	9.08	8.56	1.44	0.0167	0.047	CEACAM6	carcinoembryonic antigen-related cell adhesic	Coding
TC0600013667.hg.1	6.01	6.55	-1.46	0.0167	0.047	ARID1B	Memczak2013 ANTISENSE, CDS, coding, INTEF	NonCoding
TC0100013751.hg.1	5.95	6.3	-1.28	0.0167	0.047	C1orf109	chromosome 1 open reading frame 109	Multiple_Complex
TC1700010747.hg.1	6.59	6.92	-1.25	0.0167	0.047	COA3	cytochrome c oxidase assembly factor 3	Multiple_Complex

TC1900010582.hg.1	4.77	4.4	1.29	0.0167	0.047	WDR87	WD repeat domain 87	Multiple_Complex
TC1600006637.hg.1	5.7	5.3	1.32	0.0167	0.0471	ZG16B	zymogen granule protein 16B	Multiple_Complex
TC1600008984.hg.1	4.53	4.95	-1.34	0.0167	0.0471	RPUSD1	RNA pseudouridylate synthase domain contai	Multiple_Complex
TC2000009149.hg.1	4.42	4.09	1.26	0.0168	0.0471	PTPRT	protein tyrosine phosphatase, receptor type, T	Multiple_Complex
TC1900007294.hg.1	3.53	2.96	1.48	0.0168	0.0471	NWD1	NACHT and WD repeat domain containing 1	Multiple_Complex
TC0600008870.hg.1	3.38	3.11	1.21	0.0168	0.0472	FUT9	fucosyltransferase 9 (alpha (1,3) fucosyltransf	Multiple_Complex
TC0100015896.hg.1	4.11	3.67	1.36	0.0168	0.0473	NUP210L; M	nucleoporin 210kDa like; microRNA 5698	Multiple_Complex
TC0200008742.hg.1	9.22	8.49	1.66	0.0169	0.0474	NCK2	NCK adaptor protein 2	Multiple_Complex
TC2000009828.hg.1	6.59	6.96	-1.29	0.0169	0.0475	ARFRP1	ADP-ribosylation factor related protein 1	Multiple_Complex
TC1100011520.hg.1	7.2	6.84	1.28	0.0169	0.0475	KRTAP5-11	keratin associated protein 5-11	Multiple_Complex
TC0300008316.hg.1	3.88	4.34	-1.37	0.0169	0.0475	PVRL3	poliovirus receptor-related 3	Multiple_Complex
TC0400009396.hg.1	6.62	6.26	1.28	0.0169	0.0475	NEIL3	nei-like DNA glycosylase 3	Multiple_Complex
TC0900008474.hg.1	6.35	6.93	-1.49	0.0169	0.0475	SNX30	sorting nexin family member 30	Multiple_Complex
TC1100007940.hg.1	7.61	7.17	1.36	0.0169	0.0476	VEGFB	vascular endothelial growth factor B	Multiple_Complex
TC1800009228.hg.1	5.62	6.13	-1.42	0.0169	0.0476	ELAC1	elaC ribonuclease Z 1	Multiple_Complex
TC0500011759.hg.1	9.78	9.33	1.36	0.017	0.0476	ATG12	autophagy related 12	Multiple_Complex
TC0900009346.hg.1	9.58	10.24	-1.57	0.017	0.0476	CBWD1	COBW domain containing 1	Multiple_Complex
TC1900011730.hg.1	6.21	5.7	1.43	0.017	0.0476	TIMM50	translocase of inner mitochondrial membrane	NonCoding
TC0600014099.hg.1	5.36	5.57	-1.16	0.017	0.0477	GTF2H4	general transcription factor IIH subunit 4	Multiple_Complex
TC1600011445.hg.1	8.11	8.61	-1.41	0.017	0.0477	SPG7	spastic paraplegia 7 (pure and complicated au	NonCoding
TC0900009209.hg.1	7.86	7.15	1.64	0.017	0.0477	PMPCA	peptidase (mitochondrial processing) alpha	Multiple_Complex
TC1300009004.hg.1	4.41	4.11	1.23	0.017	0.0478	ATP7B	ATPase, Cu++ transporting, beta polypeptide	Multiple_Complex
TC1100006441.hg.1	10.98	10.38	1.52	0.017	0.0478	PSMD13	proteasome 26S subunit, non-ATPase 13	Multiple_Complex
TC1900011905.hg.1	6.26	5.95	1.24	0.017	0.0478	TMEM221	transmembrane protein 221	Multiple_Complex
TC1900008416.hg.1	4.92	4.41	1.42	0.0171	0.048	INAFM1	InaF-motif containing 1	Multiple_Complex
TC1900009350.hg.1	5.14	4.82	1.25	0.0171	0.0481	PLIN4	perilipin 4	Coding
TC2000008110.hg.1	5.8	5.32	1.39	0.0172	0.0481	OPRL1	opiate receptor-like 1	Multiple_Complex
TC0700012812.hg.1	14.05	15.06	-2.02	0.0172	0.0482	MKRN1	makorin ring finger protein 1	Multiple_Complex
TC2200008367.hg.1	5.24	4.91	1.26	0.0172	0.0482	CHEK2	checkpoint kinase 2	Multiple_Complex
TC0800007959.hg.1	4.73	4.39	1.27	0.0172	0.0482	STAU2-AS1	STAU2 antisense RNA 1	NonCoding
TC0800011731.hg.1	9.23	9.69	-1.38	0.0172	0.0482	KIAA0196	KIAA0196	Multiple_Complex

TSUnmapped00000677.h	10.72	11.29	-1.48	0.0172	0.0482	RPL7A	ribosomal protein L7a	Coding
TC0600014096.hg.1	6.14	6.56	-1.34	0.0172	0.0482	TRIM39-RP	TRIM39-RPP21 readthrough	Coding
TC0500007665.hg.1	6.11	5.76	1.28	0.0172	0.0483	CCNB1	cyclin B1	Multiple_Complex
TC1900006552.hg.1	4.98	4.73	1.19	0.0173	0.0484	ONECUT3	one cut homeobox 3	Coding
TC2000008436.hg.1	6.74	7.33	-1.51	0.0173	0.0484	TASP1	taspase 1	Multiple_Complex
TC1700009474.hg.1	5.12	4.71	1.33	0.0173	0.0484	OR1P1	olfactory receptor, family 1, subfamily P, mem	Pseudogene
TC1100006932.hg.1	4.61	4.16	1.36	0.0173	0.0484	INSC	inscuteable homolog (Drosophila)	Multiple_Complex
TC0700010910.hg.1	5.89	6.33	-1.35	0.0173	0.0485	POLD2	polymerase (DNA directed), delta 2, accessory	Multiple_Complex
TC1000012164.hg.1	5.77	6.15	-1.3	0.0173	0.0485	DHX32	DEAH (Asp-Glu-Ala-His) box polypeptide 32	Multiple_Complex
TC0100013700.hg.1	5.1	4.66	1.36	0.0173	0.0485	CLSPN	claspin	Multiple_Complex
TC0200013265.hg.1	8.37	8.15	1.17	0.0173	0.0485	CAPG	capping protein (actin filament), gelsolin-like	Multiple_Complex
TSUnmapped00000010.h	4.29	3.82	1.39	0.0174	0.0485	NLRP10	NLR family, pyrin domain containing 10	Coding
TC0700013342.hg.1	5.31	5.71	-1.32	0.0174	0.0486	CHN2	chimerin 2	Multiple_Complex
TC1100010984.hg.1	4.79	4.26	1.45	0.0174	0.0487	OR10V1	olfactory receptor, family 10, subfamily V, me	Coding
TC1700010684.hg.1	8.67	8.03	1.55	0.0174	0.0487	HAP1	huntingtin-associated protein 1	Coding
TC2200007556.hg.1	5.35	5.09	1.2	0.0174	0.0488	MPPED1	metallophosphoesterase domain containing 1	Multiple_Complex
TC1600009535.hg.1	8.16	7.73	1.35	0.0175	0.0488	FOPNL	FGFR1OP N-terminal like	Multiple_Complex
TC0100008402.hg.1	4.24	3.9	1.27	0.0175	0.0488	MROH7; TT	maestro heat-like repeat family member 7; te	Multiple_Complex
TC0500011721.hg.1	9.87	10.36	-1.4	0.0175	0.0489	REEP5	receptor accessory protein 5	Multiple_Complex
TC0800009266.hg.1	4.77	5.17	-1.32	0.0175	0.049	MFSD3	major facilitator superfamily domain containir	Multiple_Complex
TC0X00007707.hg.1	6.42	6.8	-1.3	0.0175	0.049	ATP7A	ATPase, Cu++ transporting, alpha polypeptide	Multiple_Complex
TC0X00010914.hg.1	4.37	4.12	1.19	0.0175	0.049	CT45A7; CT	cancer/testis antigen family 45, member A7; c	Coding
TC0600011880.hg.1	8.7	8.28	1.33	0.0176	0.049	GTPBP2	GTP binding protein 2	Multiple_Complex
TC0700009337.hg.1	9.5	9.07	1.35	0.0176	0.0491	C7orf55-LU	C7orf55-LUC7L2 readthrough; LUC7-like 2 pre	Multiple_Complex
TC0400011188.hg.1	7.13	6.61	1.43	0.0176	0.0491	LIN54	lin-54 DREAM MuvB core complex componen	Multiple_Complex
TC0300006961.hg.1	4.34	4.55	-1.15	0.0176	0.0491	CMTM8	CKLF-like MARVEL transmembrane domain co	Coding
TC1200010774.hg.1	6.97	6.59	1.3	0.0176	0.0492	KRT76	keratin 76, type II	Coding
TC1900011106.hg.1	6.43	5.88	1.47	0.0176	0.0492	NUCB1	Memczak2013 ANTISENSE, CDS, coding, INTEF	NonCoding
TC0800009444.hg.1	3.86	3.65	1.16	0.0176	0.0493	DEFA5	defensin, alpha 5, Paneth cell-specific	Coding
TC0900011441.hg.1	3.78	3.42	1.29	0.0177	0.0493	OR1N1	olfactory receptor, family 1, subfamily N, men	Coding
TC1700011746.hg.1	3.99	4.42	-1.34	0.0177	0.0493	SLC25A19	solute carrier family 25 (mitochondrial thiamir	Multiple_Complex
TC0200016623.hg.1	6.15	6.72	-1.49	0.0177	0.0493	KIDINS220	kinase D-interacting substrate 220kDa	NonCoding
TC0200008281.hg.1	7.84	8.34	-1.42	0.0177	0.0493	PTCD3	pentatricopeptide repeat domain 3	Multiple_Complex
TC0100008106.hg.1	4.72	4.41	1.24	0.0177	0.0493	BTBD19	BTB (POZ) domain containing 19	Multiple_Complex
TC0Y00006569.hg.1	3.86	3.59	1.2	0.0177	0.0495	TSPY3; TSP	testis specific protein, Y-linked 3; testis specifi	Multiple_Complex
TC0900011440.hg.1	4.28	3.9	1.3	0.0177	0.0495	OR1J1	olfactory receptor, family 1, subfamily J, mem	Coding
TC1300009740.hg.1	6.49	5.73	1.69	0.0178	0.0495	LIG4	ligase IV, DNA, ATP-dependent	Coding
TC1300006987.hg.1	5.92	6.27	-1.27	0.0178	0.0496	DGKH	diacylglycerol kinase, eta	Multiple_Complex
TC2100007399.hg.1	3.79	3.64	1.11	0.0178	0.0497	FAM207A	family with sequence similarity 207, member ,	Multiple_Complex
TC0200012172.hg.1	3.21	2.99	1.17	0.0178	0.0497	SRD5A2	steroid-5-alpha-reductase, alpha polypeptide	Multiple_Complex
TC0600011733.hg.1	4.94	4.6	1.27	0.0179	0.0498	KCNK5	potassium channel, two pore domain subfami	Multiple_Complex
TC0100010585.hg.1	5.12	4.6	1.43	0.0179	0.0498	FMO1	Transcript Identified by AceView, Entrez Gene	Unassigned
TC0300006542.hg.1	6.67	6.53	1.11	0.0179	0.0498	THUMPD3	THUMP domain containing 3	Multiple_Complex
TC1100012314.hg.1	3.77	3.41	1.29	0.0179	0.0498	PIH1D2	PIH1 domain containing 2	Multiple_Complex
TC1900008513.hg.1	4.73	4.47	1.2	0.0179	0.0498	CGB2	chorionic gonadotropin, beta polypeptide 2	Coding
TC1100008983.hg.1	5.77	5.35	1.33	0.0179	0.0498	ACAT1	acetyl-CoA acetyltransferase 1	Multiple_Complex
TC1500007015.hg.1	4.37	4.77	-1.32	0.0179	0.0499	JMJD7; PLA	jumonji domain containing 7; phospholipase A	Multiple_Complex
TC0800011021.hg.1	6.94	7.65	-1.63	0.0179	0.0499	RMDN1	regulator of microtubule dynamics 1	Multiple_Complex

Supplementary Table 4. Symptomatic versus Asymptomatic

ID	Description	setSize	enrichmen	NES	pvalue	p.adjust	qvalues	rank	leading_edge	core_enrichment
hsa05340	Primary immunodeficiency	15	-0.84331	-2.60704	2.14E-05	0.007138	0.006363	120	tags=47%, list=4%, sig	ZAP70/CIITA/CD4/LCK/CD79A/CD3E/IL7R
hsa05146	Amoebiasis	22	0.970433	2.464056	4.73E-05	0.00787	0.007016	8	tags=9%, list=0%, sigr	CXCL8/IL1R2
hsa04622	RIG-I-like receptor signaling pathway	14	0.981222	2.338225	0.000132558	0.011378	0.010143	9	tags=13%, list=0%, sig	CXCL8/NFKBIA
hsa05134	Legionellosis	18	0.971765	2.425981	0.000136672	0.011378	0.010143	9	tags=11%, list=0%, sig	CXCL8/NFKBIA
hsa04064	NF-kappa B signaling pathway	35	0.946949	2.578675	0.000214327	0.013207	0.011773	9	tags=11%, list=0%, sig	CXCL8/BCL2A1/TNFAIP3/NFKBIA
hsa05219	Bladder cancer	13	0.967593	2.331597	0.000237968	0.013207	0.011773	36	tags=15%, list=1%, sig	CXCL8/MMP9
hsa05144	Malaria	18	0.951883	2.376346	0.000453105	0.019285	0.017191	1	tags=5%, list=0%, sigr	CXCL8
hsa04933	AGE-RAGE signaling pathway in diabetic complications	22	0.960582	2.439042	0.000464192	0.019285	0.017191	48	tags=14%, list=1%, sig	CXCL8/EGR1/JUN
hsa04072	Phospholipase D signaling pathway	26	0.961682	2.527692	0.000521213	0.019285	0.017191	1	tags=4%, list=0%, sigr	CXCL8
hsa05120	Epithelial cell signaling in Helicobacter pylori infection	20	0.963419	2.424798	0.000602955	0.020078	0.017898	9	tags=10%, list=0%, sig	CXCL8/NFKBIA
hsa05133	Pertussis	20	0.952718	2.397865	0.000895731	0.027116	0.024172	1	tags=5%, list=0%, sigr	CXCL8
hsa05417	Lipid and atherosclerosis	57	0.895568	2.550634	0.001771302	0.048779	0.043483	48	tags=7%, list=1%, sigr	CXCL8/NFKBIA/MMP9/JUN
hsa05332	Graft-versus-host disease	19	-0.7335	-2.37641	0.002215798	0.048779	0.043483	515	tags=68%, list=15%, s	HLA-DMB/FASLG/HLA-DQB1/KLRC1/HLA-DRB1/HLA-DQA1/CD28/HLA-DRA/HLA-DMA/HLA-DPB1/PRF1/KLRD1/HLA-DPA1
hsa05202	Transcriptional misregulation in cancer	65	0.903703	2.583629	0.002230851	0.048779	0.043483	78	tags=12%, list=2%, sig	CXCL8/BCL2A1/IL1R2/MMP9/DEFA1B/DEFA1/DEFA3/NFKBIZ
hsa04657	IL-17 signaling pathway	25	0.954488	2.475543	0.002267223	0.048779	0.043483	54	tags=28%, list=2%, sig	CXCL8/S100A8/TNFAIP3/NFKBIA/MMP9/JUN/S100A9
hsa04940	Type I diabetes mellitus	17	-0.71224	-2.28248	0.002734136	0.048779	0.043483	515	tags=65%, list=15%, s	HLA-DMB/FASLG/HLA-DQB1/HLA-DRB1/HLA-DQA1/CD28/HLA-DRA/HLA-DMA/HLA-DPB1/PRF1/HLA-DPA1
hsa05167	Kaposi sarcoma-associated herpesvirus infection	56	0.882561	2.530445	0.003107683	0.048779	0.043483	137	tags=14%, list=4%, sig	CXCL8/NFKBIA/JUN/CLEC2B/FOS/GNG11/PTGS2/HIF1A
hsa05142	Chagas disease	33	0.924825	2.524755	0.003266238	0.048779	0.043483	9	tags=6%, list=0%, sigr	CXCL8/NFKBIA
hsa04620	Toll-like receptor signaling pathway	25	0.943831	2.447901	0.003279783	0.048779	0.043483	9	tags=8%, list=0%, sigr	CXCL8/NFKBIA
hsa05130	Pathogenic Escherichia coli infection	54	0.913173	2.589678	0.003297306	0.048779	0.043483	9	tags=4%, list=0%, sigr	CXCL8/NFKBIA
hsa05161	Hepatitis B	48	0.917499	2.569112	0.003440869	0.048779	0.043483	48	tags=8%, list=1%, sigr	CXCL8/NFKBIA/MMP9/JUN
hsa04218	Cellular senescence	44	0.923514	2.586131	0.003650994	0.048779	0.043483	1	tags=2%, list=0%, sigr	CXCL8
hsa05132	Salmonella infection	74	0.88306	2.544307	0.003722987	0.048779	0.043483	48	tags=5%, list=1%, sigr	CXCL8/NFKBIA/TXN/JUN
hsa05131	Shigellosis	81	0.878635	2.548849	0.003761012	0.048779	0.043483	9	tags=2%, list=0%, sigr	CXCL8/NFKBIA
hsa05171	Coronavirus disease - COVID-19	92	0.855915	2.501187	0.003852965	0.048779	0.043483	187	tags=36%, list=6%, sig	CXCL8/NFKBIA/RPL34/RPL7/RPL26/RPL23/RPS24/RPL21/RPL21/RPL37/RPL36A/JUN/RPS7/RPL41/RPS17/RPL21/RPL11/RPL41/RPL36AL/RPL9/RPL17/RPL9/RPL17-C18orf32/RPL36A-HNRNPH2/FOS/RPS25/RPL41/RPL41/RPS25/RPS21/F13A1/RPL31/RPL7/RPS27/RPS6/RPL22/RPS23/RPL39/SELP
hsa05320	Autoimmune thyroid disease	18	-0.69018	-2.24392	0.003955085	0.048779	0.043483	515	tags=61%, list=15%, s	HLA-DMB/FASLG/HLA-DQB1/HLA-DRB1/HLA-DQA1/CD28/HLA-DRA/HLA-DMA/HLA-DPB1/PRF1/HLA-DPA1
hsa05330	Allograft rejection	18	-0.69018	-2.24392	0.003955085	0.048779	0.043483	515	tags=61%, list=15%, s	HLA-DMB/FASLG/HLA-DQB1/HLA-DRB1/HLA-DQA1/CD28/HLA-DRA/HLA-DMA/HLA-DPB1/PRF1/HLA-DPA1

Supplementary Table 5. Severe versus mild
#%result_name=Analysis_34
#%array_type=Clariom_S_Human
#%annotation=Clariom_S_Human.r1.na36.hg38.a1.transcript.csv
#%comparison=sev-COVID vs mild-COVID

ID	sev-COVID	mild-COVI[	Fold Chang	P-val	FDR P-val	Gene Symbol	Description	Group
TC0100017793.hg.1	11.18	9.46	3.28	9.40E-05	0.9761	IRF2BP2	interferon regulatory factor 2 binding protein 2	Multiple_Complex
TC0100011384.hg.1	12.05	10.91	2.19	0.0003	0.9761	MAPKAPK2	mitogen-activated protein kinase-activated protein kinase 2	Multiple_Complex
TC0600007384.hg.1	5.16	6.29	-2.19	0.0004	0.9761	HIST1H2BO	histone cluster 1, H2bo	Coding
TC1900010017.hg.1	10.41	9.37	2.05	0.0005	0.9761	ELL	elongation factor RNA polymerase II	Multiple_Complex
TC0700008147.hg.1	8.92	10.16	-2.37	0.0005	0.9761	RSBN1L	round spermatid basic protein 1-like	Multiple_Complex
TC0100013126.hg.1	6.21	4.78	2.68	0.0006	0.9761	EMC1	ER membrane protein complex subunit 1	Multiple_Complex
TC0900007654.hg.1	4.74	3.51	2.34	0.0006	0.9761	GNAQ; skeyplorb	Memczak2013 ANTISENSE, coding, INTERNAL, intronic best transcript NM_002072; Transcript Identified by AceView	NonCoding
TC0200007188.hg.1	7.17	8.45	-2.43	0.0006	0.9761	BIRC6	Transcript Identified by AceView, Entrez Gene ID(s) 57448	Coding
TC2000007083.hg.1	6.18	4.41	3.42	0.0007	0.9761	ID1	inhibitor of DNA binding 1, dominant negative helix-loop-helix protein	Multiple_Complex
TC1700009528.hg.1	12.96	10.84	4.37	0.0007	0.9761	CXCL16	chemokine (C-X-C motif) ligand 16	Multiple_Complex
TC0500007337.hg.1	13.71	14.97	-2.39	0.0008	0.9761	PARP8	poly(ADP-ribose) polymerase family member 8	Multiple_Complex
TC0200007194.hg.1	5.71	7.34	-3.07	0.001	0.9761	BIRC6	Transcript Identified by AceView, Entrez Gene ID(s) 57448	Unassigned
TC0100007552.hg.1	9.39	8.3	2.12	0.001	0.9761	SESN2	sestrin 2	Multiple_Complex
TC0300009789.hg.1	7.84	9.24	-2.64	0.0012	0.9761	ST6GAL1	ST6 beta-galactosamide alpha-2,6-sialyltranferase 1	Multiple_Complex
TC0200007999.hg.1	5.56	7.24	-3.2	0.0013	0.9761	ZNF638	Transcript Identified by AceView, Entrez Gene ID(s) 27332	Coding
TC0700013594.hg.1	6.48	7.63	-2.22	0.0017	0.9761	TRIM4	tripartite motif containing 4	Multiple_Complex
TC1700007402.hg.1	11.59	9.61	3.93	0.0018	0.9761	FLOT2	Memczak2013 ANTISENSE, coding, INTERNAL, UTR3 best transcript NM_004475	NonCoding
TC0100014653.hg.1	9.89	10.94	-2.07	0.0019	0.9761	USP33	ubiquitin specific peptidase 33	Multiple_Complex
TC0200007035.hg.1	5.44	6.69	-2.37	0.002	0.9761	EPT1	ethanolaminephosphotransferase 1	Multiple_Complex
TC1000010925.hg.1	8.89	10.88	-3.98	0.0027	0.9761	PRF1	perforin 1 (pore forming protein)	Multiple_Complex
TC0300011936.hg.1	10.7	11.74	-2.06	0.0027	0.9761	CD47	CD47 molecule	Multiple_Complex
TC0200009074.hg.1	6.92	8.89	-3.92	0.0033	0.9761	PTPN4	protein tyrosine phosphatase, non-receptor type 4 (megakaryocyte)	Multiple_Complex
TC1500008135.hg.1	5.08	6.19	-2.16	0.0035	0.9761	UBE2Q2L	ubiquitin conjugating enzyme E2Q family member 2-like	Coding
TC0900006758.hg.1	7.88	9.03	-2.22	0.0035	0.9761	DENND4C	DENN/MADD domain containing 4C	Multiple_Complex
TC0100010674.hg.1	6.67	7.75	-2.12	0.0037	0.9761	GPR52	G protein-coupled receptor 52	Coding
TC1600009620.hg.1	14.04	15.29	-2.38	0.0037	0.9761	SMG1	SMG1 phosphatidylinositol 3-kinase-related kinase	Multiple_Complex
TC0400009330.hg.1	7.31	8.56	-2.38	0.0039	0.9761	SAP30	Sin3A associated protein 30kDa	Multiple_Complex
TC0600011145.hg.1	9.8	12.82	-8.07	0.0041	0.9761	HIST1H4H	histone cluster 1, H4h	Coding
TC0100017059.hg.1	6.9	7.99	-2.14	0.0041	0.9761	RBBP5	retinoblastoma binding protein 5	Multiple_Complex
TC1900008214.hg.1	10.22	6.54	12.78	0.0041	0.9761	CD177	CD177 molecule	Coding
TC0600014247.hg.1	14.84	16.3	-2.75	0.0042	0.9761	FAM65B	family with sequence similarity 65, member B	Multiple_Complex
TC0600007301.hg.1	10.93	13.58	-6.28	0.0042	0.9761	BTN3A2	butyrophilin, subfamily 3, member A2	Multiple_Complex
TC0600009697.hg.1	4.23	5.53	-2.46	0.0042	0.9761	PHACTR2	Jeck2013 ALT_ACCEPTOR, ALT_DONOR, coding, INTERNAL, intronic best transcript NM_001100164	NonCoding
TC0100016318.hg.1	9.02	7.99	2.04	0.0042	0.9761	MPC2	mitochondrial pyruvate carrier 2	Multiple_Complex
TC0600011386.hg.1	14.89	12.59	4.94	0.0043	0.9761	IER3	immediate early response 3	Coding
TC1000009919.hg.1	7.18	8.19	-2.01	0.0044	0.9761	TRDMT1	tRNA aspartic acid methyltransferase 1	Multiple_Complex
TC1700010953.hg.1	4.26	5.68	-2.68	0.0044	0.9761	KPNB1	Memczak2013 ANTISENSE, CDS, coding, INTERNAL best transcript NM_002265	NonCoding
TC0300009706.hg.1	5.16	6.24	-2.12	0.0044	0.9761	POLR2H	polymerase (RNA) II (DNA directed) polypeptide H	Multiple_Complex
TC0200007702.hg.1	9.37	10.4	-2.04	0.0045	0.9761	PAPOLG	poly(A) polymerase gamma	Multiple_Complex
TC1900011381.hg.1	5	3.84	2.23	0.0045	0.9761	AC008753.6; MYA	Memczak2013 ANTISENSE, coding, INTERNAL, intronic best transcript NM_001020819; novel transcript, antisense to MYADM	NonCoding
TC0100011755.hg.1	5.42	4.37	2.08	0.0048	0.9761	DNAH14	dynein, axonemal, heavy chain 14	Multiple_Complex
TC0200016442.hg.1	6.47	7.73	-2.39	0.0052	0.9761	MSH2	mutS homolog 2	Multiple_Complex
TC1700009398.hg.1	7.34	5.9	2.72	0.0054	0.9761	MIR22HG	MIR22 host gene	Multiple_Complex

TC0100010244.hg.1	11.9	14.66	-6.75	0.0055	0.9761	IFI16	interferon, gamma-inducible protein 16	Multiple_Complex
TC0300012596.hg.1	7.65	9.09	-2.7	0.0058	0.9761	COPB2	coatomer protein complex subunit beta 2 (beta prime)	Multiple_Complex
TC0600007375.hg.1	8.44	10.49	-4.14	0.0059	0.9761	HIST1H3H	histone cluster 1, H3h	Coding
TC0100009555.hg.1	8.34	11.01	-6.36	0.006	0.9761	CD2	CD2 molecule	Coding
TC0200012307.hg.1	9.61	10.66	-2.08	0.0061	0.9761	SOS1	SOS Ras/Rac guanine nucleotide exchange factor 1	Multiple_Complex
TC0300008888.hg.1	11.08	12.25	-2.25	0.0063	0.9761	CEP63	centrosomal protein 63kDa	Multiple_Complex
TC0800006880.hg.1	9.18	10.95	-3.41	0.0065	0.9761	PCM1	pericentriolar material 1	Multiple_Complex
TC1200007251.hg.1	12.05	14.06	-4.04	0.0065	0.9761	KIAA1551	KIAA1551	Multiple_Complex
TC0100015023.hg.1	12.74	14.43	-3.21	0.0066	0.9761	DPYD	dihydropyrimidine dehydrogenase	Multiple_Complex
TC0600013125.hg.1	7.5	10.07	-5.95	0.0067	0.9761	THEMIS	thymocyte selection associated	Coding
TC0600012875.hg.1	9.53	10.88	-2.54	0.0069	0.9761	FYN	FYN proto-oncogene, Src family tyrosine kinase	Multiple_Complex
TC0800012469.hg.1	8.76	10.4	-3.12	0.0069	0.9761	TMEM71	transmembrane protein 71	NonCoding
TC0600007270.hg.1	13.62	14.63	-2.02	0.0072	0.9761	HIST1H2AC	histone cluster 1, H2ac	Multiple_Complex
TC2100007140.hg.1	14.55	13	2.92	0.0076	0.9761	ETS2	v-ets avian erythroblastosis virus E26 oncogene homolog 2	Multiple_Complex
TC0X00010357.hg.1	7.99	5.81	4.53	0.0077	0.9761	GLA	galactosidase, alpha	Multiple_Complex
TC0700012111.hg.1	4.99	6.44	-2.74	0.008	0.9761	FBXL13	F-box and leucine-rich repeat protein 13	Multiple_Complex
TC0500011896.hg.1	6.39	7.47	-2.11	0.0082	0.9761	ZNF608	zinc finger protein 608	Multiple_Complex
TC1100013081.hg.1	7.94	6.86	2.11	0.0088	0.9761	FUT4	fucosyltransferase 4 (alpha (1,3) fucosyltransferase, myeloid-specific)	Coding
TC0400012638.hg.1	6.29	5.13	2.24	0.009	0.9761	UFSP2	UFM1-specific peptidase 2	Multiple_Complex
TC0500007138.hg.1	11.09	13.41	-4.99	0.0092	0.9761	IL7R	interleukin 7 receptor	Multiple_Complex
TC1700010218.hg.1	12.1	9.98	4.36	0.0092	0.9761	FLOT2	flotillin 2	Multiple_Complex
TC0300010775.hg.1	9.54	11.7	-4.45	0.0093	0.9761	CX3CR1	chemokine (C-X3-C motif) receptor 1	Coding
TC0600007303.hg.1	9.05	10.45	-2.64	0.0095	0.9761	BTN3A1	butyrophilin, subfamily 3, member A1	Multiple_Complex
TC1700010497.hg.1	9.26	10.68	-2.67	0.0096	0.9761	SYNRG	synergin, gamma	Multiple_Complex
TC1700010587.hg.1	7.06	8.2	-2.2	0.0096	0.9761	IKZF3	IKAROS family zinc finger 3	Multiple_Complex
TC0400012900.hg.1	12.62	14.65	-4.08	0.0098	0.9761	FBXL5	F-box and leucine-rich repeat protein 5	Multiple_Complex
TC0200014269.hg.1	4.56	3.33	2.36	0.0099	0.9761	POTEI	POTE ankyrin domain family, member I	Multiple_Complex
TC1600011186.hg.1	9.84	8.49	2.55	0.0099	0.9761	SLC7A5	solute carrier family 7 (amino acid transporter light chain, L system), member 5	Multiple_Complex
TC1000006754.hg.1	14.09	15.33	-2.36	0.01	0.9761	CELF2	CUGBP, Elav-like family member 2	Multiple_Complex
TC0200007900.hg.1	10.88	12.14	-2.4	0.01	0.9761	ARHGAP25	Rho GTPase activating protein 25	Multiple_Complex
TC1000010117.hg.1	7.66	8.7	-2.06	0.0101	0.9761	ACBD5	acyl-CoA binding domain containing 5	Multiple_Complex
TC1100007273.hg.1	12.42	11.06	2.58	0.0102	0.9761	CD44	CD44 molecule (Indian blood group)	Multiple_Complex
TC0600007306.hg.1	9.79	11.74	-3.86	0.0102	0.9761	BTN3A3	butyrophilin, subfamily 3, member A3	Multiple_Complex
TC0100016944.hg.1	12.1	10.97	2.18	0.0105	0.9761	ARL8A	ADP-ribosylation factor like GTPase 8A	Multiple_Complex
TC0700011797.hg.1	10.36	13.18	-7.04	0.0105	0.9761	SAMD9L	sterile alpha motif domain containing 9-like	Multiple_Complex
TC0200014857.hg.1	5	6.36	-2.57	0.0105	0.9761	FASTKD1	FAST kinase domains 1	Multiple_Complex
TC1100011545.hg.1	8.84	7.57	2.42	0.0106	0.9761	ANAPC15	anaphase promoting complex subunit 15	Multiple_Complex
TC1100007728.hg.1	10.16	11.38	-2.32	0.0108	0.9761	FAM111A	family with sequence similarity 111, member A	Multiple_Complex
TC1000011660.hg.1	6.83	8.62	-3.47	0.0108	0.9761	MGEA5	Salzman2013 ALT_ACCEPTOR, ALT_DONOR, coding, INTERNAL, intronic best transcript NM_012215	NonCoding
TC0500012497.hg.1	11.66	10.45	2.32	0.0109	0.9761	TNIP1	TNFAIP3 interacting protein 1	Multiple_Complex
TC1900008328.hg.1	5.91	4.75	2.23	0.011	0.9761	PPM1N	protein phosphatase, Mg2+/Mn2+ dependent, 1N (putative)	Multiple_Complex
TC0200013943.hg.1	7.82	8.97	-2.22	0.011	0.9761	SLC35F5	solute carrier family 35, member F5	Multiple_Complex
TC1400010617.hg.1	9.27	11.74	-5.55	0.0112	0.9761	PRKCH	protein kinase C, eta	Multiple_Complex
TC0X00008081.hg.1	6.28	7.34	-2.09	0.0117	0.9761	ATG4A	autophagy related 4A, cysteine peptidase	Multiple_Complex
TC0600007274.hg.1	9.36	10.44	-2.12	0.0119	0.9761	HIST1H2BD	histone cluster 1, H2bd	Coding
TC1300009249.hg.1	7.57	9.42	-3.6	0.0122	0.9761	KLF12	Kruppel-like factor 12	Multiple_Complex
TC1700007749.hg.1	7.25	8.37	-2.17	0.0125	0.9761	CDK12	Transcript Identified by AceView, Entrez Gene ID(s) 51755	Unassigned
TC1300008359.hg.1	9.96	11.3	-2.53	0.0127	0.9761	PARP4	poly(ADP-ribose) polymerase family member 4	Multiple_Complex
TC1500009005.hg.1	6.19	7.26	-2.1	0.0127	0.9761	ZNF770	zinc finger protein 770	Multiple_Complex
TC0900006438.hg.1	12.93	14.08	-2.22	0.0128	0.9761	DOCK8	dedicator of cytokinesis 8	Multiple_Complex
TC0200008534.hg.1	8.54	9.86	-2.49	0.0128	0.9761	ANKRD36	ankyrin repeat domain 36	Multiple_Complex
TC0200007181.hg.1	8.37	9.83	-2.74	0.013	0.9761	YIPF4	Yip1 domain family member 4	Multiple_Complex
TC1200010518.hg.1	11.39	12.52	-2.19	0.0135	0.9761	SLC38A2	solute carrier family 38, member 2	Multiple_Complex
TC1000011469.hg.1	4.39	5.59	-2.3	0.0138	0.9761	NOC3L	NOC3-like DNA replication regulator	Multiple_Complex

TC0X00006671.hg.1	8.68	9.71	-2.03	0.0138	0.9761	MOSPD2	motile sperm domain containing 2	Multiple_Complex
TC0100016135.hg.1	8.28	10.59	-4.96	0.0148	0.9761	SLAMF6	SLAM family member 6	Coding
TC1800006487.hg.1	15.21	16.64	-2.7	0.0151	0.9761	SMCHD1	structural maintenance of chromosomes flexible hinge domain containing 1	Multiple_Complex
TC0100010526.hg.1	5.36	4.24	2.17	0.0157	0.9761	TBX19	T-box 19	Multiple_Complex
TC0400012964.hg.1	11.34	12.87	-2.88	0.0159	0.9761	SCLT1	sodium channel and clathrin linker 1	Multiple_Complex
TC0800007127.hg.1	6.38	8.36	-3.94	0.0161	0.9761	ELP3	elongator acetyltransferase complex subunit 3	Multiple_Complex
TC1900011655.hg.1	8.12	6.76	2.56	0.0162	0.9761	PET100	PET100 homolog	Multiple_Complex
TC0100010243.hg.1	8.64	11.78	-8.83	0.0165	0.9761	PYHIN1	pyrin and HIN domain family, member 1	Multiple_Complex
TC0100009870.hg.1	8.93	10.22	-2.44	0.0173	0.9761	HIST2H4B; HIST2H	histone cluster 2, H4b; histone cluster 2, H4a	Coding
TC1700009318.hg.1	13.87	12.21	3.14	0.0173	0.9761	FAM101B	family with sequence similarity 101, member B	Multiple_Complex
TC0900009825.hg.1	9.41	11.45	-4.11	0.0174	0.9761	DDX58	DEAD (Asp-Glu-Ala-Asp) box polypeptide 58	Multiple_Complex
TC0100012242.hg.1	6.82	8.08	-2.4	0.0174	0.9761	EFCAB2	EF-hand calcium binding domain 2	Multiple_Complex
TC0300007256.hg.1	9.07	10.53	-2.77	0.0176	0.9761	CCR2	chemokine (C-C motif) receptor 2	Multiple_Complex
TC0600014220.hg.1	9.82	10.84	-2.03	0.0176	0.9761	SERPINB9	serpin peptidase inhibitor, clade B (ovalbumin), member 9	Multiple_Complex
TC0300010561.hg.1	6.98	8.08	-2.14	0.0179	0.9761	SLC4A7	solute carrier family 4, sodium bicarbonate cotransporter, member 7	Multiple_Complex
TC0300013108.hg.1	7.2	8.45	-2.38	0.018	0.9761	TNIK	TRAF2 and NCK interacting kinase	Multiple_Complex
TC0X00008344.hg.1	7.33	8.84	-2.86	0.018	0.9761	SH2D1A	SH2 domain containing 1A	Multiple_Complex
TC0300011502.hg.1	6.25	7.68	-2.69	0.0183	0.9761	FOXP1; RP11-298	Transcript Identified by AceView, Entrez Gene ID(s) 27086; novel transcript, sense intronic to FOXP1	NonCoding
TC0600008050.hg.1	11.2	12.86	-3.17	0.0184	0.9761	UBR2	ubiquitin protein ligase E3 component n-recognin 2	Multiple_Complex
TC0500009055.hg.1	6.53	7.82	-2.45	0.0187	0.9761	ABLIM3	actin binding LIM protein family, member 3	Multiple_Complex
TC0100014579.hg.1	7.95	9.35	-2.64	0.0191	0.9761	ZRANB2	zinc finger, RAN-binding domain containing 2	Multiple_Complex
TC0100007396.hg.1	4.19	5.83	-3.11	0.0191	0.9761	RHD	Rh blood group, D antigen	Multiple_Complex
TC1100010090.hg.1	4.9	5.94	-2.06	0.0192	0.9761	LYVE1	lymphatic vessel endothelial hyaluronan receptor 1	Multiple_Complex
TC0100018246.hg.1	8.19	9.65	-2.76	0.0196	0.9761	LRRC8C	leucine rich repeat containing 8 family, member C	Multiple_Complex
TC0600013880.hg.1	7.62	8.76	-2.2	0.0199	0.9761	SFT2D1	SFT2 domain containing 1	Multiple_Complex
TC1500009261.hg.1	8.26	9.45	-2.29	0.0199	0.9761	SPG11	spastic paraplegia 11 (autosomal recessive)	Multiple_Complex
TC0800012371.hg.1	10.44	14.12	-12.8	0.0199	0.9761	LY6E	lymphocyte antigen 6 complex, locus E	Multiple_Complex
TC0100008132.hg.1	6.3	7.5	-2.3	0.0199	0.9761	TMEM69	transmembrane protein 69	Multiple_Complex
TC1800007595.hg.1	5.86	4.6	2.39	0.02	0.9761	CCDC102B	coiled-coil domain containing 102B	Multiple_Complex
TC1600010809.hg.1	9.13	10.36	-2.35	0.02	0.9761	TXNL4B	thioredoxin-like 4B	Multiple_Complex
TC0700007831.hg.1	6.58	7.93	-2.56	0.0201	0.9761	TPST1	tyrosylprotein sulfotransferase 1	Multiple_Complex
TC0400012366.hg.1	8.25	10.74	-5.65	0.0211	0.9761	DDX60	DEAD (Asp-Glu-Ala-Asp) box polypeptide 60	Multiple_Complex
TC0900008148.hg.1	9	10.18	-2.27	0.0211	0.9761	TDRD7	tudor domain containing 7	Multiple_Complex
TC1200010105.hg.1	7.48	8.51	-2.05	0.0214	0.9761	RECQL	RecQ helicase-like	Multiple_Complex
TC1200006788.hg.1	8.87	11.85	-7.91	0.0214	0.9761	KLRD1	killer cell lectin-like receptor subfamily D, member 1	Multiple_Complex
TC0600007257.hg.1	9.43	11.21	-3.45	0.0218	0.9761	TRIM38	tripartite motif containing 38	Multiple_Complex
TC1100013205.hg.1	9.77	8.48	2.44	0.0218	0.9761	ARAP1	ArfGAP with RhoGAP domain, ankyrin repeat and PH domain 1	Multiple_Complex
TC0100018483.hg.1	11.57	12.82	-2.38	0.0219	0.9761	HIST2H2BE	histone cluster 2, H2be	Coding
TC0100015707.hg.1	9.57	10.82	-2.38	0.0222	0.9761	HIST2H4A; HIST2H	histone cluster 2, H4a; histone cluster 2, H4b	Multiple_Complex
TC0900007029.hg.1	12.9	11.82	2.11	0.0226	0.9761	UBAP1	ubiquitin associated protein 1	Multiple_Complex
TC0800010002.hg.1	5.9	4.82	2.12	0.0227	0.9761	DUSP4	dual specificity phosphatase 4	Multiple_Complex
TC0200014623.hg.1	8.7	9.81	-2.16	0.023	0.9761	STAM2	signal transducing adaptor molecule (SH3 domain and ITAM motif) 2	Multiple_Complex
TC0100010926.hg.1	6.6	8.04	-2.71	0.023	0.9761	OCLM	Transcript Identified by AceView, Entrez Gene ID(s) 10896	Unassigned
TC1200008906.hg.1	9.21	10.32	-2.15	0.0232	0.9761	TRAFD1	TRAF-type zinc finger domain containing 1	Multiple_Complex
TC0100009935.hg.1	5.92	7.21	-2.44	0.0233	0.9761	PRUNE	prune exopolyphosphatase	Multiple_Complex
TC1700007585.hg.1	8.33	10.45	-4.36	0.0238	0.9761	SLFN5	schlafen family member 5	Multiple_Complex
TC0900011523.hg.1	13.06	11.92	2.22	0.0241	0.9761	HSPA5	heat shock 70kDa protein 5 (glucose-regulated protein, 78kDa)	Multiple_Complex
TC0600013204.hg.1	8.29	9.57	-2.43	0.0242	0.9761	VNN3	vanin 3	Multiple_Complex
TC0200016679.hg.1	9.47	8.32	2.22	0.0245	0.9761	SERTAD2	SERTA domain containing 2	Multiple_Complex
TC1700012440.hg.1	7.2	8.37	-2.25	0.0247	0.9761	RNFT1	ring finger protein, transmembrane 1	Multiple_Complex
TC0300008346.hg.1	7.06	8.48	-2.68	0.0249	0.9761	SLC35A5	solute carrier family 35, member A5	Multiple_Complex

TC0300008561.hg.1	11.58	14.08	-5.65	0.0249	0.9761	PARP14	poly(ADP-ribose) polymerase family member 14	Multiple_Complex
TC1200012753.hg.1	5.98	7.61	-3.1	0.0251	0.9761	KLRC2	killer cell lectin-like receptor subfamily C, member 2	Coding
TC0300011853.hg.1	8.32	9.43	-2.15	0.0252	0.9761	SENP7	SUMO1/sentrin specific peptidase 7	Multiple_Complex
TC0600007307.hg.1	8.73	9.96	-2.33	0.0253	0.9761	BTN2A1	butyrophilin, subfamily 2, member A1	Multiple_Complex
TC0200012405.hg.1	6.43	8.18	-3.36	0.0254	0.9761	THADA	thyroid adenoma associated	Multiple_Complex
TC1600011075.hg.1	11.95	10.9	2.07	0.0262	0.9761	ZDHHC7	zinc finger, DHHC-type containing 7	Multiple_Complex
TC0300007242.hg.1	8.4	9.46	-2.08	0.0263	0.9761	SACM1L	SAC1 suppressor of actin mutations 1-like (yeast)	Multiple_Complex
TC1100009101.hg.1	5.92	7.16	-2.35	0.0265	0.9761	ZBTB16	zinc finger and BTB domain containing 16	Multiple_Complex
TC1900008326.hg.1	7.4	5.34	4.17	0.0266	0.9761	FOSB	FBJ murine osteosarcoma viral oncogene homolog B	Multiple_Complex
TC1700012198.hg.1	5.29	6.51	-2.33	0.0268	0.9761	PIGL	phosphatidylinositol glycan anchor biosynthesis class L	Multiple_Complex
TC0100014459.hg.1	7.56	8.57	-2.02	0.0269	0.9761	LINC01359	long intergenic non-protein coding RNA 1359	NonCoding
TC1000008400.hg.1	6.98	12.89	-60.48	0.0272	0.9761	IFIT1	interferon-induced protein with tetratricopeptide repeats 1	Coding
TSUnmapped00000401.hg.	9.8	10.87	-2.1	0.0274	0.9761	INPP5D	inositol polyphosphate-5-phosphatase D	NonCoding
TC0600008346.hg.1	6.92	7.94	-2.02	0.0275	0.9761	KIAA1586	KIAA1586	Multiple_Complex
TC1000009149.hg.1	6.73	7.96	-2.34	0.0278	0.9761	PLEKHA1	pleckstrin homology domain containing, family A (phosphoinositide binding specific) member 1	Multiple_Complex
TC0100009331.hg.1	9.76	11.28	-2.88	0.0278	0.9761	STXBP3	syntaxin binding protein 3	Multiple_Complex
TC0100008978.hg.1	7.63	8.76	-2.19	0.0281	0.9761	LRRC8B	leucine rich repeat containing 8 family, member B	Multiple_Complex
TC0100011219.hg.1	6.6	8.29	-3.23	0.0283	0.9761	PPP1R12B	protein phosphatase 1, regulatory subunit 12B	Multiple_Complex
TC0800011861.hg.1	7.38	8.94	-2.95	0.0287	0.9761	LRRC6	leucine rich repeat containing 6	Multiple_Complex
TC1200008921.hg.1	7.31	10.39	-8.47	0.0287	0.9761	OAS2	2-5-oligoadenylate synthetase 2	Multiple_Complex
TC1200012708.hg.1	9.29	13.13	-14.35	0.029	0.9761	OAS1	2-5-oligoadenylate synthetase 1	Multiple_Complex
TC0800006983.hg.1	8.8	9.92	-2.17	0.0292	0.9761	PPP3CC	protein phosphatase 3, catalytic subunit, gamma isozyme	Multiple_Complex
TC0900011302.hg.1	7.21	8.63	-2.68	0.0297	0.9761	TNFSF8	tumor necrosis factor (ligand) superfamily, member 8	Multiple_Complex
TC2000009673.hg.1	6.82	5.62	2.29	0.0299	0.9761	SYCP2	synaptonemal complex protein 2	Multiple_Complex
TC0100007964.hg.1	6.17	7.36	-2.29	0.0299	0.9761	ZNF684	zinc finger protein 684	Multiple_Complex
TC0100015182.hg.1	10.68	9.64	2.06	0.0301	0.9761	TAF13	TAF13 RNA polymerase II, TATA box binding protein (TBP)-associated factor, 18kDa	Multiple_Complex
TC1700006676.hg.1	8.47	9.9	-2.7	0.0302	0.9761	RABEP1	rabaptin, RAB GTPase binding effector protein 1	Multiple_Complex
TC1900009637.hg.1	8.61	7.6	2.01	0.0306	0.9761	CDKN2D	cyclin-dependent kinase inhibitor 2D (p19, inhibits CDK4)	Coding
TC1100007461.hg.1	7.66	6.62	2.06	0.0308	0.9761	ARHGAP1	Memczak2013 ANTISENSE, CDS, coding, INTERNAL, UTR3 best transcript NM_004308	NonCoding
TC0600011136.hg.1	7.33	8.93	-3.02	0.0309	0.9761	HIST1H2BG	histone cluster 1, H2bg	Multiple_Complex
TC0500012247.hg.1	12.56	14.23	-3.18	0.0311	0.9761	CD14	CD14 molecule	Coding
TC0300013877.hg.1	7.63	9.15	-2.87	0.0317	0.9761	EPHB1	EPH receptor B1	Multiple_Complex
TC1900008496.hg.1	11.95	10.69	2.41	0.032	0.9761	PPP1R15A	protein phosphatase 1, regulatory subunit 15A	Multiple_Complex
TC0X00010915.hg.1	4.52	5.62	-2.14	0.0327	0.9761	CT45A2	cancer/testis antigen family 45, member A2	Coding
TC0500009481.hg.1	17.91	18.95	-2.05	0.0335	0.9761	ATP6V0E1	ATPase, H+ transporting, lysosomal 9kDa, V0 subunit e1	Multiple_Complex
TC1500010698.hg.1	9.26	10.74	-2.8	0.0335	0.9761	SNRPN; SNURF; SI	small nuclear ribonucleoprotein polypeptide N; SNRPN upstream reading frame; small nucleolar RNA, C/D box 107; Prader Willi/Angelman region RNA, SNRPN neighbor	Multiple_Complex
TC1200006738.hg.1	6.66	9.24	-6	0.0338	0.9761	KLRG1	killer cell lectin-like receptor subfamily G, member 1	Multiple_Complex
TC0300013146.hg.1	11.2	13.23	-4.09	0.0341	0.9761	TNFSF10	tumor necrosis factor (ligand) superfamily, member 10	Multiple_Complex
TC1300008760.hg.1	10.18	12.66	-5.57	0.0344	0.9761	EPSTI1	epithelial stromal interaction 1 (breast)	Multiple_Complex
TC2100007208.hg.1	11.02	14.25	-9.37	0.0348	0.9761	MX1	MX dynamin-like GTPase 1	Multiple_Complex
TC0200012159.hg.1	4.33	3.33	2	0.0349	0.9761	CAPN13	calpain 13	Multiple_Complex
TC0100015797.hg.1	6.29	7.33	-2.05	0.035	0.9761	POGZ	Transcript Identified by AceView, Entrez Gene ID(s) 23126	Unassigned
TC1700007319.hg.1	9.8	11.37	-2.97	0.035	0.9761	WSB1	WD repeat and SOCS box containing 1	Multiple_Complex
TC0700008560.hg.1	12.39	11.34	2.06	0.0353	0.9761	GNB2	guanine nucleotide binding protein (G protein), beta polypeptide 2	Multiple_Complex
TC0500009268.hg.1	8.99	10.06	-2.11	0.0354	0.9761	TTC1	tetratricopeptide repeat domain 1	Multiple_Complex

TC0100012496.hg.1	11.66	12.97	-2.48	0.0359	0.9761	NADK	NAD kinase	Multiple_Complex
TC0300011223.hg.1	8.25	7.2	2.08	0.036	0.9761	SELK	selenoprotein K; selenoprotein K [Source:EntrezGene;Acc:58515]; Transcript Identified by AceView, Entrez Gene ID(s) 58515, RefSeq ID(s) NM_021237	Multiple_Complex
TC0500010781.hg.1	5.52	6.55	-2.04	0.0363	0.9761	SLC38A9	Transcript Identified by AceView, Entrez Gene ID(s) 153129	Unassigned
TC2200007783.hg.1	8.93	7.6	2.53	0.0366	0.9761	PIM3	Pim-3 proto-oncogene, serine/threonine kinase	Multiple_Complex
TC0X00006938.hg.1	4.59	3.44	2.22	0.0366	0.9761	CFAP47	cilia and flagella associated protein 47	Multiple_Complex
TC0100016476.hg.1	10.72	11.77	-2.07	0.0367	0.9761	KIAA0040	KIAA0040	Multiple_Complex
TC0600007293.hg.1	5.15	6.41	-2.4	0.0369	0.9761	HIST1H2BI	histone cluster 1, H2bi	Coding
TC1700010459.hg.1	8.33	7.1	2.33	0.0372	0.9761	CCL3	chemokine (C-C motif) ligand 3	Multiple_Complex
TC1100008779.hg.1	4.42	3.2	2.32	0.0373	0.9761	CCDC67	coiled-coil domain containing 67	Multiple_Complex
TC1200007626.hg.1	10.42	11.69	-2.41	0.0373	0.9761	METTL7A	methyltransferase like 7A	Coding
TC0100016185.hg.1	16.16	17.47	-2.49	0.0375	0.9761	FCGR3A	Fc fragment of IgG, low affinity IIIa, receptor (CD16a)	Multiple_Complex
TC1900009824.hg.1	13	11.77	2.35	0.0383	0.9761	DNAJB1	DnaJ (Hsp40) homolog, subfamily B, member 1	Multiple_Complex
TC0500011881.hg.1	6.45	7.76	-2.48	0.0386	0.9761	CEP120	centrosomal protein 120kDa	Multiple_Complex
TC0100013189.hg.1	4.29	3.28	2.01	0.0389	0.9761	KIF17	kinesin family member 17	Multiple_Complex
TC0800006692.hg.1	8.66	7.41	2.37	0.0395	0.9761	MSRA	methionine sulfoxide reductase A	Multiple_Complex
TC0200011020.hg.1	8.69	10.09	-2.64	0.0399	0.9761	SP140	SP140 nuclear body protein	Multiple_Complex
TC0Y00006898.hg.1	5.84	7.16	-2.49	0.0404	0.9761	DHRSX	Jeck2013 ALT_ACCEPTOR, ALT_DONOR, coding, INTERNAL, intronic best transcript NM_145177	NonCoding
TC0100018525.hg.1	5.65	4.5	2.23	0.0408	0.9761	ILDR2	immunoglobulin-like domain containing receptor 2	NonCoding
TC1200012190.hg.1	8.94	11.42	-5.6	0.0415	0.9761	OASL	2-5-oligoadenylate synthetase-like	Multiple_Complex
TC1100011372.hg.1	8.73	7.46	2.42	0.0416	0.9761	CHKA	choline kinase alpha	Multiple_Complex
TC0100008874.hg.1	9.83	10.88	-2.07	0.0417	0.9761	PRKACB	protein kinase, cAMP-dependent, catalytic, beta	Multiple_Complex
TC0800011472.hg.1	5.56	6.71	-2.22	0.0418	0.9761	NUDCD1	NudC domain containing 1	Multiple_Complex
TC1900006477.hg.1	5.23	6.36	-2.19	0.042	0.9761	FSTL3	follistatin-like 3 (secreted glycoprotein)	Multiple_Complex
TC0200008675.hg.1	13.46	14.83	-2.58	0.0421	0.9761	IL18RAP	interleukin 18 receptor accessory protein	Multiple_Complex
TC1500007615.hg.1	5.55	6.58	-2.04	0.0422	0.9761	MAP2K1	Transcript Identified by AceView, Entrez Gene ID(s) 5604	Unassigned
TC0200012091.hg.1	4.59	3.52	2.11	0.0423	0.9761	ZNF512	Memczak2013 ANTISENSE, coding, INTERNAL, UTR3 best transcript NM_032434	NonCoding
TC0600011814.hg.1	7.81	9.32	-2.83	0.0425	0.9761	CCND3	Memczak2013 ALT_ACCEPTOR, ALT_DONOR, coding, INTERNAL, intronic best transcript NM_001136017	NonCoding
TC1200010908.hg.1	8.27	9.48	-2.3	0.0425	0.9761	STAT2	signal transducer and activator of transcription 2	Multiple_Complex
TC1100012037.hg.1	7.49	8.85	-2.56	0.0426	0.9761	MAML2	mastermind-like transcriptional coactivator 2	Multiple_Complex
TC1900007748.hg.1	7.58	6.57	2.02	0.0427	0.9761	PDCD5	programmed cell death 5	Multiple_Complex
TC1000011704.hg.1	12.46	13.48	-2.02	0.0428	0.9761	NT5C2	5-nucleotidase, cytosolic II	Multiple_Complex
TC0100009939.hg.1	7.39	8.52	-2.19	0.0433	0.9761	GABPB2	GA binding protein transcription factor, beta subunit 2	Multiple_Complex
TC0900010582.hg.1	5.85	7.09	-2.35	0.0437	0.9761	KIF27	kinesin family member 27	Multiple_Complex
TC0600014110.hg.1	11.7	13.07	-2.59	0.0449	0.9761	PSMB9	proteasome subunit beta 9	Multiple_Complex
TC1300006658.hg.1	6.19	4.99	2.3	0.0459	0.9761	WASF3	WAS protein family, member 3	Multiple_Complex
TC0100014852.hg.1	11.07	13.03	-3.88	0.0463	0.9761	GBP1	guanylate binding protein 1, interferon-inducible	Multiple_Complex
TC0700009677.hg.1	8.5	9.89	-2.61	0.0466	0.9761	GIMAP4	GTPase, IMAP family member 4	Multiple_Complex
TC1200012864.hg.1	7.71	8.79	-2.12	0.0477	0.9761	CCDC92	coiled-coil domain containing 92	Multiple_Complex
TC0200014995.hg.1	15.79	16.81	-2.03	0.0477	0.9761	WIPF1	WAS/WASL interacting protein family, member 1	Multiple_Complex
TC1000009714.hg.1	6.95	8.17	-2.34	0.0479	0.9761	PRKCQ	protein kinase C, theta	Multiple_Complex
TC0600008863.hg.1	6.7	7.92	-2.32	0.0479	0.9761	MANEA	mannosidase, endo-alpha	Multiple_Complex
TC1700010447.hg.1	15.96	17.68	-3.3	0.0479	0.9761	CCL5	chemokine (C-C motif) ligand 5	Coding
TC1700012387.hg.1	7.15	8.19	-2.05	0.0483	0.9761	SLFN12L	schlafen family member 12-like	Multiple_Complex
TC1800008331.hg.1	9.49	10.77	-2.42	0.0485	0.9761	SS18	synovial sarcoma translocation, chromosome 18	Multiple_Complex
TC0700012797.hg.1	9.68	11.62	-3.84	0.0486	0.9761	PARP12	poly(ADP-ribose) polymerase family member 12	Multiple_Complex
TC2000009013.hg.1	7.21	8.67	-2.74	0.0488	0.9761	SLA2	Src-like-adaptor 2	Coding
TC1000008397.hg.1	9.73	14.38	-25.12	0.0496	0.9761	IFIT3	interferon-induced protein with tetratricopeptide repeats 3	Coding
TC0100007676.hg.1	7.71	9.07	-2.56	0.0498	0.9761	LCK	LCK proto-oncogene, Src family tyrosine kinase	Multiple_Complex

Supplementary Fig 6. Asymptomatic versus uninfected
#%result_name=Analysis_31
#%array_type=Clariom_S_Human
#%annotation=Clariom_S_Human.r1.na36.hg38.a1.transcript.csv
#%comparison=Asymp vs uninfected

ID	Asymp Avg	uninfected	Fold Chang	P-val	FDR P-val	Gene Symbol	Description	Group
TC1600007014.hg.1	9.76	11.63	-3.65	1.03E-09	1.68E-05	NDE1; MIR484	nudE neurodevelopment protein 1; microRNA 484	Multiple_Complex
TC0600007377.hg.1	11.58	8.07	11.42	1.57E-09	1.68E-05	HIST1H2BM	histone cluster 1, H2bm	Coding
TC1600009935.hg.1	10.88	8.84	4.12	5.44E-09	3.89E-05	SPN	Memczak2013 ANTISENSE, CDS, coding, INTERNAL be	NonCoding
TC1400007950.hg.1	14.18	12.65	2.9	7.39E-09	3.96E-05	CALM1	calmodulin 1 (phosphorylase kinase, delta)	Multiple_Complex
TC1000010498.hg.1	7.65	8.94	-2.45	1.12E-08	4.80E-05		Transcript Identified by AceView, Entrez Gene ID(s) 2	Unassigned
TC1300008998.hg.1	9.02	11.21	-4.56	2.17E-08	6.67E-05	LINC00282	long intergenic non-protein coding RNA 282	NonCoding
TC1400008056.hg.1	10.81	5.74	33.55	2.18E-08	6.67E-05	IFI27	interferon, alpha-inducible protein 27	Multiple_Complex
TC0200006677.hg.1	8	5.84	4.49	5.38E-08	0.0001	RRM2	ribonucleotide reductase M2	Multiple_Complex
TC1900011852.hg.1	5.45	6.75	-2.47	6.12E-08	0.0001	RFX2	regulatory factor X, 2 (influences HLA class II expressi	Multiple_Complex
TC1900010073.hg.1	9.77	11.22	-2.72	6.31E-08	0.0001	LPAR2	lysophosphatidic acid receptor 2	Multiple_Complex
TC0300008563.hg.1	9.67	11.15	-2.8	6.86E-08	0.0001	DIRC2	disrupted in renal carcinoma 2	Multiple_Complex
TC0900007457.hg.1	6.35	9.11	-6.76	7.21E-08	0.0001	CNTNAP3P2; CNT	contactin associated protein-like 3 pseudogene 2; co	Multiple_Complex
TC0400008105.hg.1	9.85	8.42	2.7	7.36E-08	0.0001	HERC6	HECT and RLD domain containing E3 ubiquitin protei	Multiple_Complex
TC0600012814.hg.1	13.17	12.01	2.23	8.03E-08	0.0001	CD164	CD164 molecule, sialomucin	Multiple_Complex
TC1400008905.hg.1	9.17	7.9	2.4	8.36E-08	0.0001	SPTSSA	serine palmitoyltransferase, small subunit A	Multiple_Complex
TC0300009855.hg.1	8.65	10.75	-4.28	1.21E-07	0.0002	IL1RAP	interleukin 1 receptor accessory protein	Multiple_Complex
TC1900007108.hg.1	15.39	13.98	2.66	1.32E-07	0.0002	CALR	calreticulin	Multiple_Complex
TC0X00009387.hg.1	7.47	9.11	-3.13	1.34E-07	0.0002	RPGR	retinitis pigmentosa GTPase regulator	Multiple_Complex
TC0200011690.hg.1	12.02	11.09	1.91	1.39E-07	0.0002	YWHAQ	tyrosine 3-monooxygenase/tryptophan 5-monooxyge	Multiple_Complex
TC1300008760.hg.1	11.7	8.22	11.12	1.41E-07	0.0002	EPSTI1	epithelial stromal interaction 1 (breast)	Multiple_Complex
TC0900010968.hg.1	9.91	8.62	2.44	1.51E-07	0.0002	TRIM14	tripartite motif containing 14	Multiple_Complex
TSUnmapped0000028	6.27	7.19	-1.89	1.88E-07	0.0002	MLXIP	MLX interacting protein	Coding
TC1100008010.hg.1	8.21	9.27	-2.09	2.15E-07	0.0002	CDC42EP2	CDC42 effector protein (Rho GTPase binding) 2	Multiple_Complex
TC2100008385.hg.1	8.51	7.54	1.97	2.27E-07	0.0002	SUMO3	small ubiquitin-like modifier 3	Multiple_Complex
TC2000007479.hg.1	9.99	14.67	-25.69	2.34E-07	0.0002	PI3	peptidase inhibitor 3, skin-derived	Multiple_Complex
TC0900010959.hg.1	6.57	9.54	-7.81	2.65E-07	0.0002	HEMGN	hemogen	Multiple_Complex
TC2000009376.hg.1	11.33	12.39	-2.09	3.00E-07	0.0002	STAU1	staufen double-stranded RNA binding protein 1	Multiple_Complex
TC1100009942.hg.1	7.16	5.47	3.24	3.60E-07	0.0003	TRIM22	Memczak2013 ANTISENSE, CDS, coding, INTERNAL, U	NonCoding
TC1800006506.hg.1	14.07	12.59	2.78	3.92E-07	0.0003	MYL12A	myosin light chain 12A	Multiple_Complex
TC1700012190.hg.1	11.43	10.56	1.83	4.00E-07	0.0003	EIF4A1; SNORD10	eukaryotic translation initiation factor 4A1; small nuc	Multiple_Complex
TC1800009268.hg.1	7.63	9.98	-5.11	4.10E-07	0.0003	DSC2	desmocollin 2	Multiple_Complex
TC1500008485.hg.1	9.04	11.1	-4.17	4.36E-07	0.0003	IGF1R	insulin-like growth factor 1 receptor	Multiple_Complex
TC0400011057.hg.1	7.59	6.79	1.74	4.36E-07	0.0003	SCARB2	scavenger receptor class B, member 2	Multiple_Complex
TC0600013431.hg.1	6.62	7.94	-2.49	4.45E-07	0.0003	PLAGL1; HYMAI	pleiomorphic adenoma gene-like 1; hydatidiform mo	Multiple_Complex
TC0700008588.hg.1	7.59	6.92	1.59	4.86E-07	0.0003	ZNHIT1	zinc finger, HIT-type containing 1	Multiple_Complex
TC1400010764.hg.1	8.35	9.25	-1.86	5.39E-07	0.0003	VTI1B	vesicle transport through interaction with t-SNAREs 1	Multiple_Complex
TC1800008584.hg.1	9.27	8.45	1.75	5.52E-07	0.0003	ATP5A1	ATP synthase, H+ transporting, mitochondrial F1 com	Multiple_Complex
TC0100007574.hg.1	14.5	16.95	-5.49	5.65E-07	0.0003	EPB41	erythrocyte membrane protein band 4.1	Multiple_Complex
TC1200008726.hg.1	10.92	12.84	-3.79	5.66E-07	0.0003	TCP11L2	t-complex 11, testis-specific-like 2	Multiple_Complex
TC0800012371.hg.1	15.19	11.2	15.91	6.25E-07	0.0003	LY6E	lymphocyte antigen 6 complex, locus E	Multiple_Complex
TC1200012708.hg.1	14.63	10.55	16.81	6.80E-07	0.0004	OAS1	2-5-oligoadenylate synthetase 1	Multiple_Complex
TC1200008920.hg.1	11.17	7.71	10.96	6.94E-07	0.0004	OAS3	2-5-oligoadenylate synthetase 3	Multiple_Complex
TC0X00008824.hg.1	12.41	11.38	2.04	8.60E-07	0.0004	TKTL1	transketolase-like 1	Multiple_Complex
TC0500010680.hg.1	9.74	7.82	3.8	8.62E-07	0.0004	PARP8	Memczak2013 ANTISENSE, CDS, coding, INTERNAL, ir	NonCoding
TC1700010254.hg.1	14.49	15.69	-2.3	9.03E-07	0.0004	SSH2	slingshot protein phosphatase 2	Multiple_Complex
TC2000008228.hg.1	8.17	6.98	2.29	9.61E-07	0.0004	SIGLEC1	sialic acid binding Ig-like lectin 1, sialoadhesin	Coding
TC0600011225.hg.1	14.71	13.65	2.09	9.99E-07	0.0005	HIST1H2AJ	histone cluster 1, H2aj	Multiple_Complex
TC0200011075.hg.1	16.04	14.93	2.16	1.20E-06	0.0005	PTMA	prothymosin, alpha	Multiple_Complex
TC0200016626.hg.1	9.3	10.82	-2.86	1.21E-06	0.0005	MBOAT2	membrane bound O-acyltransferase domain containi	Multiple_Complex
TC2200006540.hg.1	7.99	5.05	7.65	1.30E-06	0.0005	USP18	ubiquitin specific peptidase 18	Multiple_Complex
TC1100012966.hg.1	8.78	10.73	-3.88	1.32E-06	0.0005	MICAL2	microtubule associated monooxygenase, calponin an	Multiple_Complex
TC1000010990.hg.1	8.16	9.11	-1.94	1.33E-06	0.0005	DNAJB12	DnaJ (Hsp40) homolog, subfamily B, member 12	Multiple_Complex
TC0X00009493.hg.1	4.78	5.92	-2.2	1.36E-06	0.0005	EFHC2	EF-hand domain (C-terminal) containing 2	Multiple_Complex
TC0600011235.hg.1	7.24	6.16	2.1	1.38E-06	0.0005	HIST1H2AM; HIST	histone cluster 1, H2am; histone cluster 1, H3j	Multiple_Complex
TC2000008678.hg.1	13.03	11.28	3.36	1.76E-06	0.0007	CST3	cystatin C	Multiple_Complex
TC0600007274.hg.1	9.58	8.49	2.13	1.84E-06	0.0007	HIST1H2BD	histone cluster 1, H2bd	Coding
TC1100013048.hg.1	7.33	6.73	1.51	1.94E-06	0.0007	DPP3	dipeptidyl-peptidase 3	Multiple_Complex
TC2000007820.hg.1	6.42	7.9	-2.79	1.96E-06	0.0007	CASS4	Cas scaffolding protein family member 4	Multiple_Complex
TC0600011124.hg.1	6.9	5.2	3.25	2.16E-06	0.0008	HIST1H3B	histone cluster 1, H3b	Coding
TC0400006706.hg.1	10.05	11.45	-2.64	2.18E-06	0.0008	TBC1D14	TBC1 domain family, member 14	Multiple_Complex
TC0100011721.hg.1	13.66	12.73	1.91	2.27E-06	0.0008	CAPN2	calpain 2, (m/II) large subunit	Multiple_Complex
TC0X00010837.hg.1	14.12	16.83	-6.54	2.29E-06	0.0008	MBNL3	muscleblind-like splicing regulator 3	Multiple_Complex
TC1600008164.hg.1	9.02	10.19	-2.25	2.37E-06	0.0008	FAM65A	family with sequence similarity 65, member A	Multiple_Complex
TC0200011624.hg.1	11.65	7.82	14.23	2.40E-06	0.0008	CMPK2	cytidine monophosphate (UMP-CMP) kinase 2, mitoc	Multiple_Complex
TC0X00007026.hg.1	11.78	12.8	-2.03	2.45E-06	0.0008	USP9X	ubiquitin specific peptidase 9, X-linked	Multiple_Complex
TC1200010927.hg.1	14.46	13.79	1.6	2.46E-06	0.0008	ATP5B	ATP synthase, H+ transporting, mitochondrial F1 com	Multiple_Complex

TC1000008881.hg.1	12.21	14.69	-5.61	2.53E-06	0.0008	MXI1	MAX interactor 1, dimerization protein	Multiple_Complex
TC0600014236.hg.1	8.6	6.95	3.13	2.80E-06	0.0009	TXNDC5	thioredoxin domain containing 5 (endoplasmic reticu	Multiple_Complex
TC2200008036.hg.1	8.17	4.88	9.82	2.90E-06	0.0009	USP41	ubiquitin specific peptidase 41	Multiple_Complex
TC2200006641.hg.1	7.13	6.46	1.59	3.00E-06	0.0009	RANBP1	RAN binding protein 1	Multiple_Complex
TC2200008370.hg.1	10.04	9.36	1.6	3.04E-06	0.0009	XBP1	X-box binding protein 1	Multiple_Complex
TC1700010677.hg.1	6.16	5.19	1.97	3.43E-06	0.001	KRT19	keratin 19, type I	Multiple_Complex
TC2100007208.hg.1	15.91	11.54	20.6	3.44E-06	0.001	MX1	MX dynamin-like GTPase 1	Multiple_Complex
TC0900008879.hg.1	13.07	12.07	2	3.47E-06	0.001	SET	SET nuclear proto-oncogene	Multiple_Complex
TC1300008424.hg.1	9.14	10.83	-3.22	3.77E-06	0.0011	USP12	ubiquitin specific peptidase 12	Multiple_Complex
TC2200009219.hg.1	14.38	11.4	7.9	3.87E-06	0.0011	IGLL5; MIR5571; I	immunoglobulin lambda-like polypeptide 5; microRN	Multiple_Complex
TC1900009838.hg.1	10.68	12.15	-2.78	3.89E-06	0.0011	ADGRE2	adhesion G protein-coupled receptor E2	Multiple_Complex
TC1600009683.hg.1	12.86	14.28	-2.67	3.92E-06	0.0011	IGSF6	immunoglobulin superfamily, member 6	Multiple_Complex
TC2000009968.hg.1	8.61	10.46	-3.6	4.02E-06	0.0011	SIRPB2	signal-regulatory protein beta 2	Multiple_Complex
TC0100013369.hg.1	7.44	6.21	2.35	4.18E-06	0.0011	STMN1; MIR3917	stathmin 1; microRNA 3917	Multiple_Complex
TC0100016625.hg.1	14.91	16.12	-2.32	4.24E-06	0.0011	GLUL	glutamate-ammonia ligase	Multiple_Complex
TC0600010797.hg.1	6.54	7.88	-2.52	4.60E-06	0.0012	MAK	male germ cell-associated kinase	Coding
TC1900011251.hg.1	14.77	12.7	4.22	4.64E-06	0.0012	NKG7	natural killer cell granule protein 7	Coding
TC1200008921.hg.1	12.13	8.85	9.72	4.68E-06	0.0012	OAS2	2-5-oligoadenylate synthetase 2	Multiple_Complex
TC0400011744.hg.1	13.66	12.39	2.4	4.69E-06	0.0012	ANXA5	annexin A5	Multiple_Complex
TC1700008128.hg.1	9.76	10.74	-1.96	4.93E-06	0.0012	NPEPPS	aminopeptidase puromycin sensitive	Multiple_Complex
TC1700008984.hg.1	13.98	15.48	-2.82	5.13E-06	0.0013	SEC14L1; SCARNA	SEC14-like lipid binding 1; small Cajal body-specific R	Multiple_Complex
TC0800008300.hg.1	9.44	10.69	-2.38	5.19E-06	0.0013	CPQ	carboxypeptidase Q	Multiple_Complex
TC0400008879.hg.1	10.37	11.79	-2.68	5.28E-06	0.0013	GAB1	GRB2-associated binding protein 1	Multiple_Complex
TC1100009301.hg.1	9.06	10.44	-2.61	5.34E-06	0.0013	TBCEL	tubulin folding cofactor E-like	Multiple_Complex
TSUnmapped000005(	10.1	11.38	-2.43	5.50E-06	0.0013	MLXIP	MLX interacting protein	NonCoding
TC1900009625.hg.1	9.95	11.46	-2.84	5.57E-06	0.0013	ICAM3	intercellular adhesion molecule 3	Multiple_Complex
TC0600011232.hg.1	8.24	6.61	3.11	5.78E-06	0.0013	HIST1H1B	histone cluster 1, H1b	Multiple_Complex
TSUnmapped000001(	9.58	11.16	-2.99	5.98E-06	0.0014	MLXIP	MLX interacting protein	NonCoding
TC0100008815.hg.1	14.02	8.13	59.44	6.02E-06	0.0014	IFI44L	interferon-induced protein 44-like	Multiple_Complex
TC0400012857.hg.1	8.92	9.89	-1.95	6.35E-06	0.0014	RAPGEF2	Rap guanine nucleotide exchange factor 2	Multiple_Complex
TC1100006876.hg.1	5.51	6.82	-2.48	6.37E-06	0.0014	MICALCL	MICAL C-terminal like	Multiple_Complex
TC1800006448.hg.1	7.14	5.77	2.59	6.41E-06	0.0014	TYMS	thymidylate synthetase	Multiple_Complex
TC1900007679.hg.1	7.32	6.42	1.86	6.70E-06	0.0015	PLEKHF1	pleckstrin homology domain containing, family F (wit	Coding
TC0700010796.hg.1	10.88	11.59	-1.63	7.26E-06	0.0016	VPS41	vacuolar protein sorting 41 homolog (S. cerevisiae)	Multiple_Complex
TC1400009147.hg.1	10.97	12.47	-2.83	7.48E-06	0.0016	PYGL	phosphorylase, glycogen, liver	Multiple_Complex
TC0600011233.hg.1	8.32	7.22	2.14	7.60E-06	0.0016	HIST1H3I	histone cluster 1, H3i	Coding
TC0300006925.hg.1	13.08	13.9	-1.76	7.62E-06	0.0016	TGFBR2	transforming growth factor beta receptor II	Multiple_Complex
TC1000006802.hg.1	7.58	9.23	-3.14	7.69E-06	0.0016	CAMK1D	calcium/calmodulin-dependent protein kinase ID	Multiple_Complex
TC1100012121.hg.1	17.6	15.71	3.72	7.80E-06	0.0016	TMEM123	transmembrane protein 123	Multiple_Complex
TC0400012366.hg.1	11.57	9.08	5.63	7.94E-06	0.0016	DDX60	DEAD (Asp-Glu-Ala-Asp) box polypeptide 60	Multiple_Complex
TC0400010612.hg.1	5.9	7.09	-2.29	7.95E-06	0.0016	NFXL1	nuclear transcription factor, X-box binding-like 1	Multiple_Complex
TC1100013015.hg.1	8.93	10.01	-2.11	8.36E-06	0.0017	MS4A14	membrane-spanning 4-domains, subfamily A, membe	Multiple_Complex
TC0600008379.hg.1	14.05	13.16	1.85	8.45E-06	0.0017	MTRNR2L9	MT-RNR2-like 9	Multiple_Complex
TC0500013392.hg.1	8.62	10.88	-4.79	8.51E-06	0.0017	CCNJL	cyclin J-like	Multiple_Complex
TC0900009933.hg.1	7.83	9.04	-2.31	8.59E-06	0.0017	FAM214B	family with sequence similarity 214, member B	Multiple_Complex
TC0500009751.hg.1	9.62	8.92	1.62	8.69E-06	0.0017	BTNL9	butyrophilin-like 9	Multiple_Complex
TC1000010699.hg.1	11.16	12.08	-1.88	9.03E-06	0.0017	IPMK	inositol polyphosphate multikinase	Multiple_Complex
TC1100011184.hg.1	9.7	10.86	-2.23	9.14E-06	0.0017	SF1	Memczak2013 ALT_ACCEPTOR, ALT_DONOR, coding,	NonCoding
TC1900009316.hg.1	15.38	16.48	-2.15	9.41E-06	0.0018	EEF2; SNORD37	eukaryotic translation elongation factor 2; small nucl	Multiple_Complex
TC0700009394.hg.1	8.2	7.35	1.8	9.52E-06	0.0018	AGK	acylglycerol kinase	Multiple_Complex
TC1200007887.hg.1	7.89	8.62	-1.66	9.96E-06	0.0018	MBD6	methyl-CpG binding domain protein 6	Multiple_Complex
TC0800007027.hg.1	13.63	15.18	-2.92	1.01E-05	0.0018	SLC25A37	solute carrier family 25 (mitochondrial iron transport	Multiple_Complex
TC1200008895.hg.1	11.31	10.56	1.68	1.01E-05	0.0018	MAPKAPK5; ADAI	mitogen-activated protein kinase-activated protein k	Multiple_Complex
TC1700009256.hg.1	9.85	10.95	-2.15	1.08E-05	0.0019	NARF	nuclear prelamin A recognition factor	Multiple_Complex
TC2000009970.hg.1	13.42	15.03	-3.05	1.12E-05	0.002	SIRPB1	signal-regulatory protein beta 1	Multiple_Complex
TC1600008007.hg.1	9.73	11.03	-2.45	1.14E-05	0.002	ADGRG3	adhesion G protein-coupled receptor G3	Multiple_Complex
TC1700012313.hg.1	9.31	8.44	1.83	1.17E-05	0.002	MRPL12	mitochondrial ribosomal protein L12	Multiple_Complex
TC0400011291.hg.1	7.65	6.29	2.57	1.19E-05	0.0021	HERC6	Memczak2013 ANTISENSE, coding, INTERNAL, introni	NonCoding
TC0600007290.hg.1	8.39	7.35	2.05	1.20E-05	0.0021	HIST1H2BH	histone cluster 1, H2bh	Coding
TC2200009218.hg.1	6.19	5.39	1.74	1.21E-05	0.0021	MIR650; IGLV3-9;	microRNA 650; immunoglobulin lambda variable 3-9	Multiple_Complex
TSUnmapped000002(	9.83	9.2	1.54	1.22E-05	0.0021	KAT6B	K(lysine) acetyltransferase 6B	NonCoding
TC0900010444.hg.1	8.96	8.3	1.58	1.24E-05	0.0021	CARNMT1	carnosine N-methyltransferase 1	Multiple_Complex
TC0800008940.hg.1	10.36	11.05	-1.61	1.24E-05	0.0021	PHF20L1	PHD finger protein 20-like 1	Multiple_Complex
TC0600007585.hg.1	10.06	9.01	2.07	1.28E-05	0.0021	MICB	MHC class I polypeptide-related sequence B	Multiple_Complex
TC0300010966.hg.1	7.06	6.26	1.75	1.28E-05	0.0021	CCDC12	coiled-coil domain containing 12	Multiple_Complex
TC1700007931.hg.1	8.16	7.18	1.96	1.29E-05	0.0021	IFI35	interferon-induced protein 35	Multiple_Complex
TC1700007451.hg.1	9.76	11.18	-2.66	1.32E-05	0.0021	CPD	carboxypeptidase D	Multiple_Complex
TC1100009309.hg.1	13.35	14.84	-2.81	1.33E-05	0.0021	SORL1	sortilin-related receptor, L(DLR class) A repeats conta	Multiple_Complex
TC2100008168.hg.1	9.18	8.57	1.53	1.35E-05	0.0021	HMGN1	high mobility group nucleosome binding domain 1	Multiple_Complex
TC0100013445.hg.1	13.53	8.97	23.61	1.36E-05	0.0021	IFI6	interferon, alpha-inducible protein 6	Multiple_Complex
TC1900011729.hg.1	8.81	7.97	1.79	1.38E-05	0.0022	TIMM50	translocase of inner mitochondrial membrane 50 hor	Multiple_Complex
TC0900008482.hg.1	10.11	10.92	-1.76	1.41E-05	0.0022	SLC31A2	solute carrier family 31 (copper transporter), membe	Multiple_Complex

TC0100016112.hg.1	13.98	15.23	-2.38	1.44E-05	0.0022	TAGLN2	transgelin 2	Multiple_Complex
TC0700007034.hg.1	15.44	17.28	-3.58	1.45E-05	0.0022	CREB5	cAMP responsive element binding protein 5	Multiple_Complex
TC0200016402.hg.1	12.94	8.14	27.98	1.45E-05	0.0022	RSAD2	radical S-adenosyl methionine domain containing 2	Multiple_Complex
TC0500013235.hg.1	10.78	11.82	-2.05	1.46E-05	0.0022	FAM53C	family with sequence similarity 53, member C	Multiple_Complex
TC1400008764.hg.1	12.42	11.11	2.48	1.47E-05	0.0022	GZMB	granzyme B	Coding
TC0800012372.hg.1	8.45	7.76	1.61	1.48E-05	0.0022	LY6E	lymphocyte antigen 6 complex, locus E	NonCoding
TC0800008943.hg.1	9.21	10.15	-1.91	1.50E-05	0.0022	PHF20L1	Transcript Identified by AceView, Entrez Gene ID(s) 5	Unassigned
TC1200006629.hg.1	17.26	16.38	1.84	1.50E-05	0.0022	GAPDH	glyceraldehyde-3-phosphate dehydrogenase	Multiple_Complex
TC1700012469.hg.1	14.13	13.36	1.7	1.50E-05	0.0022	SUMO2	small ubiquitin-like modifier 2	Multiple_Complex
TC1100013012.hg.1	10.96	12	-2.06	1.61E-05	0.0023	STX3	syntaxin 3	Multiple_Complex
TC0200013096.hg.1	14.12	13.35	1.71	1.63E-05	0.0023	MOB1A	MOB kinase activator 1A	Multiple_Complex
TC2100008424.hg.1	10.88	12.47	-3.01	1.63E-05	0.0023	SLC19A1	solute carrier family 19 (folate transporter), member	Multiple_Complex
TC0900008750.hg.1	8.33	9.06	-1.66	1.69E-05	0.0024	GAPVD1	GTPase activating protein and VPS9 domains 1	Multiple_Complex
TC1100013062.hg.1	6.28	7.04	-1.68	1.72E-05	0.0024	NADSYN1	NAD synthetase 1	Multiple_Complex
TC0200010795.hg.1	10.48	12.23	-3.37	1.72E-05	0.0024	SLC11A1	solute carrier family 11 (proton-coupled divalent met	Multiple_Complex
TC0100018097.hg.1	8.76	8.11	1.58	1.75E-05	0.0024	ZNF496	zinc finger protein 496	Multiple_Complex
TC0300013330.hg.1	8.33	9.86	-2.89	1.83E-05	0.0025	DCUN1D1	DCN1, defective in cullin neddylation 1, domain cont	Multiple_Complex
TC0400010961.hg.1	13.74	10.22	11.51	1.83E-05	0.0025	JCHAIN	joining chain of multimeric IgA and IgM	Multiple_Complex
TC1700010827.hg.1	9.5	7.84	3.16	1.85E-05	0.0025	GRN	Memczak2013 ANTISENSE, CDS, coding, INTERNAL be	NonCoding
TC0400012856.hg.1	6.62	7.42	-1.73	1.86E-05	0.0025	RAPGEF2	Rap guanine nucleotide exchange factor 2	NonCoding
TC1800006889.hg.1	10.46	12.69	-4.69	1.89E-05	0.0026	RIOK3	RIO kinase 3	Multiple_Complex
TC0100007243.hg.1	8.78	11.78	-7.99	1.91E-05	0.0026	ALPL	alkaline phosphatase, liver/bone/kidney	Multiple_Complex
TC0900011754.hg.1	8.92	8.33	1.5	1.93E-05	0.0026	QRFP	pyroglutamylated RFamide peptide	Coding
TC1000012510.hg.1	5.82	5.16	1.59	1.97E-05	0.0026	ZNF511	zinc finger protein 511	Multiple_Complex
TC1100008041.hg.1	13.31	11.82	2.8	2.05E-05	0.0027	CTSW	cathepsin W	Multiple_Complex
TC0300012339.hg.1	4.35	5.3	-1.93	2.06E-05	0.0027	GATA2	GATA binding protein 2	Multiple_Complex
TC1500009904.hg.1	11.33	12.72	-2.61	2.11E-05	0.0027	TLE3	transducin-like enhancer of split 3	Multiple_Complex
TC1900011399.hg.1	11.03	12.44	-2.65	2.11E-05	0.0027	MBOAT7	membrane bound O-acyltransferase domain containi	Multiple_Complex
TC0200016559.hg.1	4.56	5.09	-1.44	2.11E-05	0.0027	BBS5	Bardet-Biedl syndrome 5	Multiple_Complex
TC1800007198.hg.1	8.51	10.65	-4.4	2.12E-05	0.0027	SLC14A1	solute carrier family 14 (urea transporter), member 1	Multiple_Complex
TC1300006683.hg.1	13.25	12.4	1.8	2.22E-05	0.0028	GTF3A	general transcription factor IIIA	Multiple_Complex
TC0700010376.hg.1	9.73	10.61	-1.83	2.27E-05	0.0029	SNX13	sorting nexin 13	Multiple_Complex
TC0100007484.hg.1	14.81	15.99	-2.27	2.32E-05	0.0029	ZDHHC18	zinc finger, DHHC-type containing 18	Multiple_Complex
TC0100012329.hg.1	9.1	11.08	-3.96	2.34E-05	0.0029	OR2W3	olfactory receptor, family 2, subfamily W, member 3	Coding
TC1700008738.hg.1	6.01	6.98	-1.95	2.40E-05	0.003	PRKAR1A	Memczak2013 ALT_ACCEPTOR, ALT_DONOR, coding,	NonCoding
TC2100008462.hg.1	7.98	7.37	1.52	2.40E-05	0.003	LSS	lanosterol synthase (2,3-oxidosqualene-lanosterol cy	Multiple_Complex
TC0500013124.hg.1	5.06	4.18	1.84	2.47E-05	0.003	TRIM7	tripartite motif containing 7	Multiple_Complex
TC0900010063.hg.1	6.38	7.93	-2.92	2.48E-05	0.003	CNTNAP3	contactin associated protein-like 3	Multiple_Complex
TC0200015538.hg.1	12.61	14.06	-2.73	2.49E-05	0.003	KLF7	Kruppel-like factor 7 (ubiquitous)	Multiple_Complex
TC1000012482.hg.1	11.26	12.43	-2.24	2.51E-05	0.003	ENTPD1	ectonucleoside triphosphate diphosphohydrolase 1	Multiple_Complex
TC0100017118.hg.1	8.05	9.55	-2.83	2.53E-05	0.003	YOD1	YOD1 deubiquitinase	Multiple_Complex
TC0300007278.hg.1	10.57	11.83	-2.39	2.55E-05	0.003	NBEAL2	neurobeachin like 2	Multiple_Complex
TC0100013471.hg.1	11.56	12.88	-2.5	2.57E-05	0.003	PTAFR	platelet-activating factor receptor	Multiple_Complex
TC0300012132.hg.1	11.48	12.1	-1.53	2.58E-05	0.003	GSK3B	glycogen synthase kinase 3 beta	Multiple_Complex
TC0200008268.hg.1	16.25	14.09	4.49	2.61E-05	0.0031	GNLY	granulysin	Multiple_Complex
TC1700007904.hg.1	8.83	8.05	1.71	2.69E-05	0.0031	TUBG1	tubulin, gamma 1	Multiple_Complex
TC0600009843.hg.1	5.8	6.7	-1.86	2.74E-05	0.0032	PLEKHG1	pleckstrin homology domain containing, family G (wi	Multiple_Complex
TC0100014947.hg.1	12.11	11.59	1.43	2.80E-05	0.0032	TMED5	transmembrane p24 trafficking protein 5	Multiple_Complex
TC0500007088.hg.1	9.63	8.7	1.9	2.81E-05	0.0032	TARS	threonyl-tRNA synthetase	Multiple_Complex
TC0200016115.hg.1	10.68	9.65	2.04	3.00E-05	0.0034	ARL4C	ADP-ribosylation factor like GTPase 4C	Multiple_Complex
TC0100007463.hg.1	12.42	11.6	1.77	3.03E-05	0.0034	HMGN2	high mobility group nucleosomal binding domain 2	Multiple_Complex
TC1200011711.hg.1	9.97	9.24	1.66	3.05E-05	0.0034	GNPTAB	N-acetylglucosamine-1-phosphate transferase, alpha	Multiple_Complex
TC2000009602.hg.1	10.41	8.58	3.56	3.06E-05	0.0034	ZBP1	Z-DNA binding protein 1	Multiple_Complex
TC1900007431.hg.1	7.85	8.53	-1.6	3.20E-05	0.0036	MAU2	MAU2 sister chromatid cohesion factor	Multiple_Complex
TC0200009955.hg.1	8.18	9.76	-3	3.22E-05	0.0036	CYBRD1	cytochrome b reductase 1	Multiple_Complex
TC1900008419.hg.1	7.77	8.88	-2.16	3.24E-05	0.0036	C5AR2	complement component 5a receptor 2	Multiple_Complex
TC2000009874.hg.1	14.72	16.72	-4	3.25E-05	0.0036	PSMF1	proteasome inhibitor subunit 1	Multiple_Complex
TC2000006861.hg.1	7.95	6.83	2.16	3.30E-05	0.0036	RIN2	Ras and Rab interactor 2	Multiple_Complex
TC1900009832.hg.1	12.22	13.81	-3.02	3.30E-05	0.0036	ADGRE3	adhesion G protein-coupled receptor E3	Coding
TC0100015866.hg.1	11.14	10.07	2.11	3.35E-05	0.0036	S100A4	S100 calcium binding protein A4	Multiple_Complex
TC0700013597.hg.1	5.67	6.24	-1.49	3.38E-05	0.0036	SAP25	Sin3A associated protein 25kDa	Multiple_Complex
TC1900010633.hg.1	9.62	8.97	1.57	3.39E-05	0.0036	CCER2	coiled-coil glutamate rich protein 2	Coding
TC0900011838.hg.1	7.1	6.43	1.58	3.43E-05	0.0037	SURF6	surfeit 6	Multiple_Complex
TC1900011821.hg.1	9.82	10.52	-1.62	3.44E-05	0.0037	ZNF776	zinc finger protein 776	Multiple_Complex
TC1000012561.hg.1	13.08	13.95	-1.84	3.47E-05	0.0037	JMJD1C	jumonji domain containing 1C	Multiple_Complex
TC0100006989.hg.1	9.11	9.79	-1.6	3.60E-05	0.0038	DNAJC16	DnaJ (Hsp40) homolog, subfamily C, member 16	Multiple_Complex
TC1900007882.hg.1	5.4	6.32	-1.89	3.60E-05	0.0038	PROSER3	proline and serine rich 3	Multiple_Complex
TC1900011958.hg.1	7.24	7.93	-1.61	3.63E-05	0.0038	ERF	Ets2 repressor factor	Multiple_Complex
TC0100011406.hg.1	17.64	18.27	-1.55	3.64E-05	0.0038	CD55	CD55 molecule, decay accelerating factor for comple	Multiple_Complex
TC1800008776.hg.1	9.09	10.96	-3.66	3.78E-05	0.0038	FECH	ferrochelatase	Multiple_Complex
TC0300013934.hg.1	7.62	8.6	-1.98	3.80E-05	0.0038	EMC3	ER membrane protein complex subunit 3	Multiple_Complex
TC1100007866.hg.1	7.45	6.89	1.48	3.80E-05	0.0038	UQCC3	ubiquinol-cytochrome c reductase complex assembly	Multiple_Complex

TC0200016682.hg.1	11.92	13.12	-2.29	3.81E-05	0.0038	PPP3R1	protein phosphatase 3, regulatory subunit B, alpha	Coding
TC0100016158.hg.1	6.89	6.41	1.39	3.81E-05	0.0038	PFDN2	prefoldin subunit 2	Multiple_Complex
TC0600014235.hg.1	9.1	7.78	2.49	3.82E-05	0.0038	BLOC1S5-TXNDC5	BLOC1S5-TXNDC5 readthrough (NMD candidate)	Multiple_Complex
TC1200008771.hg.1	9.96	10.67	-1.63	4.01E-05	0.004	ISCU	iron-sulfur cluster assembly enzyme	Multiple_Complex
TC0500009061.hg.1	6.51	7.21	-1.62	4.11E-05	0.0041	PCYOX1L	prenylcysteine oxidase 1 like	Multiple_Complex
TC0600011135.hg.1	6.69	5.74	1.92	4.16E-05	0.0041	HIST1H3D; HIST1I	histone cluster 1, H3d; histone cluster 1, H2ad	Multiple_Complex
TC0300007194.hg.1	7.64	8.72	-2.11	4.20E-05	0.0041	ABHD5	abhydrolase domain containing 5	Multiple_Complex
TC1600011545.hg.1	7.83	7.01	1.76	4.20E-05	0.0041	PLLP	plasmolipin	NonCoding
TC0700009827.hg.1	12.09	12.78	-1.61	4.21E-05	0.0041	RBM33	RNA binding motif protein 33	Multiple_Complex
TC1500008360.hg.1	9.81	10.71	-1.87	4.21E-05	0.0041	SLCO3A1	solute carrier organic anion transporter family, mem	Multiple_Complex
TC0600009094.hg.1	6.55	7.3	-1.69	4.24E-05	0.0041	SMPD2	sphingomyelin phosphodiesterase 2, neutral membra	Multiple_Complex
TC0800010765.hg.1	14.64	14.07	1.48	4.34E-05	0.0042	TRAM1	translocation associated membrane protein 1	Multiple_Complex
TC0100008025.hg.1	14.76	15.9	-2.21	4.38E-05	0.0042	YBX1	Y box binding protein 1	Multiple_Complex
TC0800009529.hg.1	7.84	9.12	-2.44	4.70E-05	0.0045	PPP1R3B	protein phosphatase 1, regulatory subunit 3B	Coding
TC1700012249.hg.1	7.46	6.91	1.46	4.71E-05	0.0045	MIR4728; ERBB2	microRNA 4728; erb-b2 receptor tyrosine kinase 2	Multiple_Complex
TC2000008279.hg.1	8.1	7.04	2.09	4.79E-05	0.0045	PCNA	proliferating cell nuclear antigen	Multiple_Complex
TC0400011383.hg.1	9.57	11.96	-5.26	4.81E-05	0.0045	TSPAN5	tetraspanin 5	Multiple_Complex
TC0300009799.hg.1	8.08	6.32	3.38	4.84E-05	0.0045	RTP4	receptor (chemosensory) transporter protein 4	Coding
TC0400012019.hg.1	6.6	9.14	-5.81	4.84E-05	0.0045	GYPA	glycophorin A (MNS blood group)	Multiple_Complex
TC0100008816.hg.1	11.68	7.88	13.96	4.90E-05	0.0046	IFI44	interferon-induced protein 44	Multiple_Complex
TC0800007080.hg.1	16.63	18.48	-3.61	4.92E-05	0.0046	BNIP3L	BCL2/adenovirus E1B 19kDa interacting protein 3-like	Multiple_Complex
TC1200012190.hg.1	10.43	8.26	4.49	4.94E-05	0.0046	OASL	2-5-oligoadenylate synthetase-like	Multiple_Complex
TC0100010369.hg.1	10.74	11.6	-1.81	4.96E-05	0.0046	ATF6	activating transcription factor 6	Multiple_Complex
TC1300008997.hg.1	8	9.5	-2.83	5.06E-05	0.0046	DHRS12	dehydrogenase/reductase (SDR family) member 12	Multiple_Complex
TC0100009572.hg.1	11.93	14.27	-5.05	5.08E-05	0.0046	FAM46C	family with sequence similarity 46, member C	Coding
TC0800009239.hg.1	7.32	6.73	1.51	5.10E-05	0.0046	CYC1	cytochrome c-1	Multiple_Complex
TC0200011720.hg.1	10.2	11.38	-2.26	5.12E-05	0.0046	ODC1; SNORA80E	ornithine decarboxylase 1; small nucleolar RNA, H/AC	Multiple_Complex
TC0X00006581.hg.1	9.62	10.77	-2.22	5.18E-05	0.0047	TBL1X	transducin (beta)-like 1X-linked	Multiple_Complex
TC2000009969.hg.1	8.73	9.79	-2.09	5.24E-05	0.0047	SIRPD	signal-regulatory protein delta	Multiple_Complex
TC1600009102.hg.1	9.87	9.26	1.52	5.26E-05	0.0047	RNPS1	RNA binding protein S1, serine-rich domain	Multiple_Complex
TC0600009789.hg.1	12.6	13.21	-1.52	5.35E-05	0.0047	TAB2	TGF-beta activated kinase 1/MAP3K7 binding protein	Multiple_Complex
TC0100013087.hg.1	9.39	8.56	1.77	5.36E-05	0.0047	RCC2	regulator of chromosome condensation 2	Multiple_Complex
TC1600009397.hg.1	6.14	5.57	1.48	5.57E-05	0.0049	PRM3	protamine 3	Coding
TC1300009229.hg.1	7.66	7.02	1.55	5.63E-05	0.005	MZT1	mitotic spindle organizing protein 1	Coding
TC1700011436.hg.1	9.24	10.03	-1.74	5.69E-05	0.005	ERN1	endoplasmic reticulum to nucleus signaling 1	Multiple_Complex
TC0600011777.hg.1	13.5	14.95	-2.73	5.82E-05	0.0051	TREM1	triggering receptor expressed on myeloid cells 1	Multiple_Complex
TSUnmapped0000000	7.37	8.13	-1.7	5.82E-05	0.0051	DUSP16	dual specificity phosphatase 16	Coding
TC2200007427.hg.1	5	5.7	-1.63	6.07E-05	0.0052	TNRC6B	Transcript Identified by AceView, Entrez Gene ID(s) 2	Unassigned
TC2100007452.hg.1	6.37	5.3	2.09	6.09E-05	0.0052	COL6A2	Jeck2013 ALT_ACCEPTOR, ALT_DONOR, coding, INTE	NonCoding
TC0100010303.hg.1	6.37	5.42	1.94	6.10E-05	0.0052	SLAMF6	Memczak2013 ANTISENSE, coding, INTERNAL, introni	NonCoding
TC0200010410.hg.1	9.87	7.49	5.2	6.12E-05	0.0052	SPATS2L	spermatogenesis associated, serine-rich 2-like	Multiple_Complex
TC0200009829.hg.1	13.17	14.39	-2.34	6.14E-05	0.0052	GCA	grancalcin, EF-hand calcium binding protein	Multiple_Complex
TC0600013914.hg.1	5.28	5.76	-1.4	6.24E-05	0.0053	GPR31	G protein-coupled receptor 31	Multiple_Complex
TC0X00006626.hg.1	11.81	13.38	-2.99	6.29E-05	0.0053	TLR8	toll-like receptor 8	Coding
TC0900011501.hg.1	4.98	5.64	-1.58	6.46E-05	0.0054	NR6A1	nuclear receptor subfamily 6, group A, member 1	Multiple_Complex
TC0600007067.hg.1	8.69	10.6	-3.75	6.52E-05	0.0055	GMPR	guanosine monophosphate reductase	Multiple_Complex
TC0600008120.hg.1	8.54	10.41	-3.66	6.53E-05	0.0055	TMEM63B	transmembrane protein 63B	Multiple_Complex
TC0300013859.hg.1	6.15	7.04	-1.86	6.57E-05	0.0055	CD200	CD200 molecule	Multiple_Complex
TC0100010140.hg.1	10.16	9.38	1.71	6.74E-05	0.0056	SYT11	synaptotagmin XI	Coding
TC1100008477.hg.1	11.51	12.87	-2.56	6.78E-05	0.0056	DGAT2	diacylglycerol O-acyltransferase 2	Multiple_Complex
TC0600014083.hg.1	8.35	7.04	2.48	6.92E-05	0.0057	HIST1H2AG	histone cluster 1, H2ag	Coding
TC2100008494.hg.1	14.25	14.91	-1.57	6.99E-05	0.0057	IFNAR2	interferon (alpha, beta and omega) receptor 2	Multiple_Complex
TC0X00007204.hg.1	7.88	9.04	-2.23	7.17E-05	0.0058	GATA1	GATA binding protein 1 (globin transcription factor 1	Multiple_Complex
TC0600011140.hg.1	7.16	6.16	1.99	7.18E-05	0.0058	HIST1H3F	histone cluster 1, H3f	Coding
TC0X00010880.hg.1	6.5	7.58	-2.12	7.22E-05	0.0058	MOSPD1	motile sperm domain containing 1	Multiple_Complex
TC2200007909.hg.1	5.1	5.87	-1.7	7.23E-05	0.0058	MICAL3	microtubule associated monooxygenase, calponin an	Multiple_Complex
TC0100017471.hg.1	11.84	12.98	-2.21	7.29E-05	0.0058	WDR26; MIR4742	WD repeat domain 26; microRNA 4742	Multiple_Complex
TC1600011515.hg.1	8.41	7.53	1.84	7.30E-05	0.0058	AC009133.12; PA	novel transcript, antisense to C16orf53 and MVP; PA	NonCoding
TC0600011142.hg.1	6.57	5.16	2.66	7.44E-05	0.0059	HIST1H3G	histone cluster 1, H3g	Coding
TC0400006951.hg.1	12.74	11.22	2.88	7.48E-05	0.0059	CD38	CD38 molecule	Multiple_Complex
TC0100012674.hg.1	5.65	5.06	1.51	7.51E-05	0.0059	GPR153	G protein-coupled receptor 153	Multiple_Complex
TC1000010925.hg.1	12.52	11.31	2.32	7.54E-05	0.0059	PRF1	perforin 1 (pore forming protein)	Multiple_Complex
TC1100009858.hg.1	10.56	11.39	-1.78	7.59E-05	0.006	CHRNA10; NUP98	cholinergic receptor, nicotinic alpha 10; nucleoporin	Multiple_Complex
TC0200016401.hg.1	6.93	5.94	1.99	7.64E-05	0.006	RSAD2	radical S-adenosyl methionine domain containing 2	NonCoding
TC0X00011223.hg.1	11.83	13.63	-3.49	7.81E-05	0.0061	MPP1	membrane protein, palmitoylated 1	Multiple_Complex
TC0600007293.hg.1	5.13	4.3	1.77	7.83E-05	0.0061	HIST1H2BI	histone cluster 1, H2bi	Coding
TC1600008146.hg.1	8.5	7.65	1.8	7.89E-05	0.0061	HSF4; FBXL8	heat shock transcription factor 4; F-box and leucine-r	Multiple_Complex
TC0100017094.hg.1	6.28	5.39	1.86	8.01E-05	0.0061	FAM72A	family with sequence similarity 72, member A	Multiple_Complex
TC1700008719.hg.1	8.17	7.3	1.84	8.01E-05	0.0061	KPNA2	karyopherin alpha 2 (RAG cohort 1, importin alpha 1)	Multiple_Complex
TC1900011775.hg.1	6.55	5.82	1.66	8.01E-05	0.0061	GRWD1	glutamate-rich WD repeat containing 1	Multiple_Complex
TC1200009242.hg.1	8.65	8.05	1.52	8.05E-05	0.0061	SNRNP35	small nuclear ribonucleoprotein, U11/U12 35kDa sub	Multiple_Complex
TC0X00008230.hg.1	10.66	12.28	-3.09	8.23E-05	0.0062	IL13RA1	interleukin 13 receptor, alpha 1	Multiple_Complex

TC0X00011397.hg.1	5.69	5.04	1.57	8.24E-05	0.0062	FAM127C	family with sequence similarity 127, member C	Coding
TC0300008371.hg.1	5.41	6.76	-2.56	8.26E-05	0.0062	GRAMD1C	GRAM domain containing 1C	Multiple_Complex
TC0100010310.hg.1	10.64	9.67	1.96	8.28E-05	0.0062	SLAMF7	SLAM family member 7	Multiple_Complex
TC0100008621.hg.1	7.49	8.44	-1.94	8.51E-05	0.0064	LEPR; LEPROT	leptin receptor; leptin receptor overlapping transcrip	Multiple_Complex
TC1700011743.hg.1	7.96	8.63	-1.59	8.98E-05	0.0067	GGA3	golgi-associated, gamma adaptin ear containing, ARF	Multiple_Complex
TC0800009878.hg.1	11.65	12.21	-1.47	9.02E-05	0.0067	ENTPD4; LOXL2	ectonucleoside triphosphate diphosphohydrolase 4;	Multiple_Complex
TC1600010604.hg.1	6.01	5.35	1.58	9.05E-05	0.0067	FAM96B	family with sequence similarity 96, member B	Multiple_Complex
TC1900009224.hg.1	6.47	7.07	-1.51	9.08E-05	0.0067	C19orf35	chromosome 19 open reading frame 35	Coding
TC0600014102.hg.1	11.8	10.84	1.94	9.10E-05	0.0067	HCP5	HLA complex P5 (non-protein coding)	Multiple_Complex
TC0600007380.hg.1	6.92	5.82	2.15	9.18E-05	0.0067	HIST1H2AL; HIST1	histone cluster 1, H2al; histone cluster 1, H2bn	Multiple_Complex
TC0800007460.hg.1	10	10.75	-1.68	9.21E-05	0.0067	HGSNAT	heparan-alpha-glucosaminide N-acetyltransferase	Multiple_Complex
TC0400012150.hg.1	13.76	15.33	-2.96	9.28E-05	0.0068	TMEM154	transmembrane protein 154	Multiple_Complex
TC1100013021.hg.1	7.09	6.37	1.65	9.36E-05	0.0068	FEN1	flap structure-specific endonuclease 1	Coding
TC0700012658.hg.1	8.35	7.68	1.59	9.37E-05	0.0068	CHCHD3	coiled-coil-helix-coiled-coil-helix domain containing 3	Multiple_Complex
TC2000008750.hg.1	7.71	7.17	1.46	9.37E-05	0.0068	NANP	N-acetylneuraminic acid phosphatase	Coding
TC1500007303.hg.1	8.93	9.66	-1.66	9.47E-05	0.0068	PIGB	phosphatidylinositol glycan anchor biosynthesis class	Multiple_Complex
TC1900007891.hg.1	10.98	9.11	3.67	9.48E-05	0.0068	HCST	hematopoietic cell signal transducer	Multiple_Complex
TC0500007163.hg.1	11.15	11.83	-1.6	9.53E-05	0.0068	NIPBL	Nipped-B homolog (Drosophila)	Multiple_Complex
TC1200012807.hg.1	7.11	6.45	1.57	9.57E-05	0.0068	STAC3	SH3 and cysteine rich domain 3	Multiple_Complex
TC1500010463.hg.1	5.07	4.43	1.56	9.59E-05	0.0068	PRC1	protein regulator of cytokinesis 1	Multiple_Complex
TC0700011166.hg.1	10.81	10.07	1.68	9.65E-05	0.0068	CHCHD2	coiled-coil-helix-coiled-coil-helix domain containing 2	Multiple_Complex
TC0800011074.hg.1	5.71	6.36	-1.56	9.67E-05	0.0068	C8orf88	chromosome 8 open reading frame 88	Multiple_Complex
TC0100016447.hg.1	11.46	12.24	-1.72	9.74E-05	0.0069	RC3H1	ring finger and CCCH-type domains 1	Multiple_Complex
TC1000008382.hg.1	6.79	8.04	-2.39	9.86E-05	0.0069	LIPN	lipase, family member N	Coding
TSUnmapped000006:	8.53	7.74	1.73	0.0001	0.0071	LRIG1		Coding
TC1600010618.hg.1	5.84	5.47	1.29	0.0001	0.0071	EXOC3L1	exocyst complex component 3-like 1	Multiple_Complex
TC0100011197.hg.1	4.17	5.44	-2.4	0.0001	0.0071	SHISA4	shisa family member 4	Multiple_Complex
TC2000007817.hg.1	8.93	10.14	-2.31	0.0001	0.0072	FAM210B	family with sequence similarity 210, member B	Multiple_Complex
TC2000007288.hg.1	5.85	5.39	1.38	0.0001	0.0074	NNAT	neuronatin	Multiple_Complex
TC1600007741.hg.1	6.15	6.92	-1.71	0.0001	0.0074	PHKB	Transcript Identified by AceView, Entrez Gene ID(s) 5	Unassigned
TC1400007354.hg.1	12.46	13.56	-2.15	0.0001	0.0074	PPM1A	protein phosphatase, Mg2+/Mn2+ dependent, 1A	Multiple_Complex
TC0500008544.hg.1	9.05	8.03	2.02	0.0001	0.0074	LMNB1	lamin B1	Multiple_Complex
TC0300013639.hg.1	9.72	9.1	1.54	0.0001	0.0074	PPP1R2	protein phosphatase 1, regulatory (inhibitor) subunit	Multiple_Complex
TC1700011097.hg.1	7.87	7.16	1.64	0.0001	0.0074	LRRC59	leucine rich repeat containing 59	Multiple_Complex
TC0300013990.hg.1	4.64	5.31	-1.59	0.0001	0.0074	TLR9	toll-like receptor 9	Coding
TC0100013602.hg.1	6.87	7.88	-2.02	0.0001	0.0075	TMEM234	transmembrane protein 234	Multiple_Complex
TC1100013028.hg.1	9.61	9.07	1.45	0.0001	0.0075	OTUB1	OTU deubiquitinase, ubiquitin aldehyde binding 1	Multiple_Complex
TC0500009562.hg.1	9.86	10.98	-2.18	0.0001	0.0075	HRH2	histamine receptor H2	Multiple_Complex
TC0600011553.hg.1	8.4	7.72	1.61	0.0001	0.0076	BAK1	BCL2-antagonist/killer 1	Multiple_Complex
TC1700006738.hg.1	13.52	12.78	1.67	0.0001	0.0076	EIF5A	eukaryotic translation initiation factor 5A	Multiple_Complex
TC1900008432.hg.1	4.78	5.34	-1.47	0.0001	0.0076	EHD2	EH domain containing 2	Multiple_Complex
TC0600011535.hg.1	10.14	11.3	-2.24	0.0001	0.0076	RGL2	ral guanine nucleotide dissociation stimulator-like 2	Multiple_Complex
TC1200011385.hg.1	6.86	8.29	-2.69	0.0001	0.0076	LIN7A	lin-7 homolog A (C. elegans)	Multiple_Complex
TC1600009202.hg.1	13	14.18	-2.26	0.0001	0.0076	CREBBP	CREB binding protein	Multiple_Complex
TC1900009608.hg.1	10	9.42	1.5	0.0001	0.0076	EIF3G	eukaryotic translation initiation factor 3, subunit G	Multiple_Complex
TC0600012072.hg.1	14.38	15.2	-1.77	0.0001	0.0076	ELOVL5	ELOVL fatty acid elongase 5	Multiple_Complex
TC0500007314.hg.1	11.37	10.61	1.69	0.0001	0.0078	MRPS30	mitochondrial ribosomal protein S30	Multiple_Complex
TC0X00006446.hg.1	10.37	11.42	-2.07	0.0001	0.0078	CSF2RA	colony stimulating factor 2 receptor, alpha, low-affin	Multiple_Complex
TC0700010585.hg.1	10.15	11.79	-3.11	0.0001	0.0078	JAZF1	JAZF zinc finger 1	Multiple_Complex
TC1900007173.hg.1	14.24	15.12	-1.85	0.0001	0.0078	ADGRE5	adhesion G protein-coupled receptor E5	Multiple_Complex
TC0800008198.hg.1	4.3	5.26	-1.95	0.0001	0.0078	OTUD6B	Transcript Identified by AceView, Entrez Gene ID(s) 5	Unassigned
TC0500011280.hg.1	9.5	8.53	1.96	0.0001	0.0078	DHFR	dihydrofolate reductase	Multiple_Complex
TC0300009727.hg.1	6.05	7.08	-2.04	0.0001	0.0078	VPS8	Transcript Identified by AceView, Entrez Gene ID(s) 2	Unassigned
TC1100009245.hg.1	10.19	11.14	-1.92	0.0001	0.0078	CBL	Cbl proto-oncogene, E3 ubiquitin protein ligase	Multiple_Complex
TC1600009530.hg.1	10.97	12.24	-2.41	0.0001	0.0078	KIAA0430; MIR65	KIAA0430; microRNA 6506	Multiple_Complex
TC0600014055.hg.1	7.98	9.44	-2.75	0.0001	0.0078	NQO2	NAD(P)H dehydrogenase, quinone 2	Multiple_Complex
TC0600014074.hg.1	8.95	9.62	-1.6	0.0001	0.0078	HIVEP1	human immunodeficiency virus type I enhancer bind	Multiple_Complex
TC0900010387.hg.1	6.62	7.44	-1.77	0.0001	0.0078	TMEM2	Transcript Identified by AceView, Entrez Gene ID(s) 2	Unassigned
TC0600011773.hg.1	9.48	10.87	-2.62	0.0001	0.0078	TREML2	triggering receptor expressed on myeloid cells-like 2	Coding
TC0300014095.hg.1	5.66	5.03	1.55	0.0001	0.0078	TM4SF19	transmembrane 4 L six family member 19	Multiple_Complex
TC1100013186.hg.1	10.32	11.23	-1.87	0.0001	0.0079	SYVN1	synovial apoptosis inhibitor 1, synoviolin	Multiple_Complex
TC0600010565.hg.1	5.67	6.41	-1.67	0.0001	0.0079	TUBB2A	tubulin, beta 2A class IIa	Multiple_Complex
TC0700009256.hg.1	10.76	12.13	-2.58	0.0001	0.0079	C7orf73	chromosome 7 open reading frame 73	Multiple_Complex
TC1000007275.hg.1	10.36	11.02	-1.58	0.0001	0.0079	CCNY	cyclin Y	Multiple_Complex
TC0X00008794.hg.1	11.79	14.68	-7.44	0.0001	0.008	SLC6A8	solute carrier family 6 (neurotransmitter transporter)	Multiple_Complex
TC0Y00006444.hg.1	10.58	11.58	-2	0.0001	0.008	CSF2RA	colony stimulating factor 2 receptor, alpha, low-affin	Multiple_Complex
TC0600010962.hg.1	13.29	12.43	1.82	0.0001	0.008	DEK	DEK proto-oncogene	Multiple_Complex
TC1500010730.hg.1	7.97	7.47	1.41	0.0001	0.0082	HYPK	huntingtin interacting protein K	Multiple_Complex
TC1900011012.hg.1	7.64	6.98	1.58	0.0001	0.0083	TMEM160	transmembrane protein 160	Multiple_Complex
TC1700006758.hg.1	10.86	10.07	1.74	0.0001	0.0083	MPDU1	mannose-P-dolichol utilization defect 1	Multiple_Complex
TC1700010599.hg.1	9.04	8.26	1.72	0.0001	0.0083	MED24; MIR6884	mediator complex subunit 24; microRNA 6884	Multiple_Complex
TC1500007034.hg.1	12.49	13.3	-1.76	0.0001	0.0084	SNAP23	synaptosome associated protein 23kDa	Multiple_Complex

TC1700012387.hg.1	9.84	8.6	2.36	0.0001	0.0084	SLFN12L	schlafen family member 12-like	Multiple_Complex
TC1000010497.hg.1	12.33	14.31	-3.94	0.0001	0.0084	8-Mar	membrane associated ring finger 8	Multiple_Complex
TC0200007296.hg.1	8.44	7.52	1.89	0.0001	0.0084	GALM	galactose mutarotase (aldose 1-epimerase)	Multiple_Complex
TC0300009122.hg.1	4.61	6	-2.63	0.0001	0.0085	CPA3	carboxypeptidase A3 (mast cell)	Multiple_Complex
TC0100014910.hg.1	10.59	9.41	2.26	0.0001	0.0086	TGFBR3	transforming growth factor beta receptor III	Multiple_Complex
TC2200007419.hg.1	11.06	11.8	-1.66	0.0001	0.0086	TNRC6B	trinucleotide repeat containing 6B	Multiple_Complex
TC1300008463.hg.1	3.92	4.61	-1.61	0.0001	0.0087	FLT1	fms-related tyrosine kinase 1	Multiple_Complex
TC0600007273.hg.1	12.73	11.5	2.34	0.0001	0.0088	HIST1H1E	histone cluster 1, H1e	Coding
TC0300013998.hg.1	8.68	8.08	1.51	0.0001	0.0088	SFMBT1	Scm-like with four mbt domains 1	Multiple_Complex
TC1100011029.hg.1	4.34	5.18	-1.79	0.0001	0.0088	VWCE	von Willebrand factor C and EGF domains	Multiple_Complex
TC0100015819.hg.1	16.04	15.17	1.83	0.0002	0.0088	S100A10	S100 calcium binding protein A10	Multiple_Complex
TC0500011263.hg.1	5.58	6.25	-1.59	0.0002	0.0088	SERINC5	Memczak2013 ALT_ACCEPTOR, ALT_DONOR, coding,	NonCoding
TC1900011255.hg.1	10.44	12.19	-3.35	0.0002	0.009	SIGLEC10	sialic acid binding Ig-like lectin 10	Multiple_Complex
TC0100017075.hg.1	9.46	9.02	1.36	0.0002	0.009	NUCKS1	nuclear casein kinase and cyclin-dependent kinase su	Multiple_Complex
TC2000007089.hg.1	5.46	4.5	1.94	0.0002	0.009	TPX2	TPX2, microtubule-associated	Multiple_Complex
TC2000009956.hg.1	9.38	9.95	-1.49	0.0002	0.0091	PCMTD2	protein-L-isoaspartate (D-aspartate) O-methyltransfe	Multiple_Complex
TC1700009974.hg.1	5.92	5.34	1.5	0.0002	0.0091	SHMT1; MIR6778	serine hydroxymethyltransferase 1 (soluble); microRI	Multiple_Complex
TC0600011525.hg.1	10.5	11.04	-1.45	0.0002	0.0093	RXRB	retinoid X receptor beta	Multiple_Complex
TC0100007333.hg.1	11.85	13.59	-3.33	0.0002	0.0094	PITHD1	PITH (C-terminal proteasome-interacting domain of t	Multiple_Complex
TC0300007296.hg.1	8.97	8.01	1.94	0.0002	0.0094	SCAP	Memczak2013 ANTISENSE, CDS, coding, INTERNAL be	NonCoding
TC1900011982.hg.1	5.62	5.13	1.41	0.0002	0.0094	OPA3	optic atrophy 3 (autosomal recessive, with chorea an	Coding
TC1100012471.hg.1	13.04	14.09	-2.08	0.0002	0.0094	AMICA1	adhesion molecule, interacts with CXADR antigen 1	Multiple_Complex
TC1700006808.hg.1	6.11	5.57	1.46	0.0002	0.0094	RANGRF	RAN guanine nucleotide release factor	Multiple_Complex
TC0500013296.hg.1	6.37	6.83	-1.37	0.0002	0.0094	C1QTNF3-AMACR	C1QTNF3-AMACR readthrough (NMD candidate)	Multiple_Complex
TC0100011512.hg.1	6.56	5.82	1.67	0.0002	0.0094	DTL	denticleless E3 ubiquitin protein ligase homolog (Dro	Multiple_Complex
TC1100013209.hg.1	9.72	10.67	-1.93	0.0002	0.0094	PAK1	p21 protein (Cdc42/Rac)-activated kinase 1	Multiple_Complex
TC0200012091.hg.1	5.34	4.41	1.91	0.0002	0.0094	ZNF512	Memczak2013 ANTISENSE, coding, INTERNAL, UTR3 b	NonCoding
TC0100015509.hg.1	6.51	5.37	2.2	0.0002	0.0094	FAM72B	family with sequence similarity 72, member B	Multiple_Complex
TC0800011285.hg.1	10.21	9.6	1.52	0.0002	0.0096	ZNF706	zinc finger protein 706	Multiple_Complex
TC0100012935.hg.1	4.11	4.67	-1.47	0.0002	0.0097	PRAMEF10	PRAME family member 10	Coding
TC1400007558.hg.1	9.61	10.43	-1.76	0.0002	0.0098	SUSD6	sushi domain containing 6	Multiple_Complex
TC0X00008718.hg.1	9.43	10.2	-1.7	0.0002	0.0098	MTMR1	myotubularin related protein 1	Multiple_Complex
TC0700013327.hg.1	8.52	7.9	1.54	0.0002	0.0099	CHST12	carbohydrate (chondroitin 4) sulfotransferase 12	Coding
TC2100007032.hg.1	7.3	6.48	1.76	0.0002	0.01	CBR1	carbonyl reductase 1	Multiple_Complex
TC1900007291.hg.1	6.11	5.26	1.8	0.0002	0.0101	TMEM38A	Transcript Identified by AceView, Entrez Gene ID(s) 7	Unassigned
TC0800007057.hg.1	14.13	15.54	-2.65	0.0002	0.0101	DOCK5	dedicator of cytokinesis 5	Multiple_Complex
TC1200008488.hg.1	13.7	14.85	-2.23	0.0002	0.0102	PLXNC1	plexin C1	Multiple_Complex
TC0200015764.hg.1	10.48	11.79	-2.47	0.0002	0.0102	CNPPD1	cyclin Pas1/PHO80 domain containing 1	Multiple_Complex
TC0100015891.hg.1	10.71	12.11	-2.65	0.0002	0.0102	CRTC2	CREB regulated transcription coactivator 2	Multiple_Complex
TC1900009765.hg.1	6.95	7.53	-1.5	0.0002	0.0102	LYL1	lymphoblastic leukemia associated hematopoiesis re	Multiple_Complex
TC0100012760.hg.1	8.81	9.36	-1.47	0.0002	0.0102	RERE	arginine-glutamic acid dipeptide (RE) repeats	Multiple_Complex
TC1900010286.hg.1	7.17	6.65	1.43	0.0002	0.0102	UQCRFS1	ubiquinol-cytochrome c reductase, Rieske iron-sulfur	Coding
TC0X00008108.hg.1	9.36	10.38	-2.01	0.0002	0.0102	TMEM164	transmembrane protein 164	Multiple_Complex
TC2000008237.hg.1	11.99	10.04	3.86	0.0002	0.0103	MAVS	Jeck2013 ANTISENSE, coding, INTERNAL, OVEXON, U	NonCoding
TC0600007544.hg.1	12.72	12.08	1.56	0.0002	0.0104	TUBB	tubulin, beta class I	Multiple_Complex
TC0800012284.hg.1	6.57	7.28	-1.63	0.0002	0.0107	EXTL3	exostosin-like glycosyltransferase 3	Multiple_Complex
TC1200009846.hg.1	8.84	7.72	2.17	0.0002	0.0108	LINC00612	long intergenic non-protein coding RNA 612	Multiple_Complex
TC0100018080.hg.1	10.13	11.08	-1.93	0.0002	0.0108	AHCTF1	AT hook containing transcription factor 1	Multiple_Complex
TC0100015894.hg.1	10.53	10.04	1.4	0.0002	0.0108	JTB	jumping translocation breakpoint	Multiple_Complex
TC2200009259.hg.1	7.21	6.62	1.51	0.0002	0.0108	SMTN	smoothelin	Multiple_Complex
TC0100009029.hg.1	9.82	8.91	1.88	0.0002	0.0108	BRDT	bromodomain, testis-specific	Multiple_Complex
TC0700012812.hg.1	15.06	17.04	-3.94	0.0002	0.0108	MKRN1	makorin ring finger protein 1	Multiple_Complex
TC1000009221.hg.1	9.61	10.52	-1.88	0.0002	0.0108	ZRANB1	zinc finger, RAN-binding domain containing 1	Multiple_Complex
TC0500011478.hg.1	8.04	9.23	-2.28	0.0002	0.0108	KIAA0825	KIAA0825	Multiple_Complex
TC1500010856.hg.1	7.71	10.2	-5.62	0.0002	0.0108	EPB42	erythrocyte membrane protein band 4.2	NonCoding
TC1400008036.hg.1	5.04	5.54	-1.42	0.0002	0.0109	COX8C	cytochrome c oxidase subunit VIIIC	Coding
TC0600011697.hg.1	5.88	5.28	1.52	0.0002	0.0109	CCDC167	coiled-coil domain containing 167	Coding
TC1000008401.hg.1	9.91	8.29	3.07	0.0002	0.0109	IFIT5	interferon-induced protein with tetratricopeptide re	Coding
TC0200014772.hg.1	11.7	10.12	2.98	0.0002	0.0109	IFIH1	interferon induced, with helicase C domain 1	Multiple_Complex
TC0500012942.hg.1	7.86	8.84	-1.98	0.0002	0.011	RNF44	ring finger protein 44	Multiple_Complex
TC1800006650.hg.1	13.08	14.36	-2.43	0.0002	0.011	RAB31	RAB31, member RAS oncogene family	Multiple_Complex
TC0400011932.hg.1	10.52	11.25	-1.65	0.0002	0.0112	ELF2	E74-like factor 2 (ets domain transcription factor)	Multiple_Complex
TC1600007959.hg.1	7.32	5.7	3.07	0.0002	0.0114	MT1E	metallothionein 1E	Multiple_Complex
TC1100012473.hg.1	8.28	8.94	-1.58	0.0002	0.0114	MPZL3	myelin protein zero-like 3	Multiple_Complex
TC0900010958.hg.1	8.5	9.16	-1.58	0.0002	0.0114	TRMO	tRNA methyltransferase O	Multiple_Complex
TSUnmapped000003	10.84	10.1	1.67	0.0002	0.0114	RCC2	regulator of chromosome condensation 2	Coding
TC1000008092.hg.1	11.19	10.64	1.47	0.0002	0.0115	VDAC2	voltage-dependent anion channel 2	Multiple_Complex
TC1700006480.hg.1	7.87	7.3	1.49	0.0002	0.0115	TIMM22	translocase of inner mitochondrial membrane 22 hor	Multiple_Complex
TC0300008782.hg.1	6.23	6.96	-1.65	0.0002	0.0115	IFT122	intraflagellar transport 122	Multiple_Complex
TC1100010207.hg.1	6.44	7.64	-2.29	0.0002	0.0116	SOX6; MIR6073	SRY box 6; microRNA 6073	Multiple_Complex
TC0100010855.hg.1	10.17	11.23	-2.09	0.0002	0.0118	NPL	N-acetylneuraminate pyruvate lyase (dihydrodipicoli	Multiple_Complex
TC0500007792.hg.1	11.33	10.63	1.62	0.0002	0.0119	HEXB	hexosaminidase B (beta polypeptide)	Multiple_Complex

TC1600010665.hg.1	8.08	8.73	-1.57	0.0002	0.0119	DPEP2	dipeptidase 2	Multiple_Complex
TC0100010625.hg.1	6.9	6.19	1.64	0.0002	0.0119	FASLG	Fas ligand (TNF superfamily, member 6)	Coding
TC0700009082.hg.1	4.87	4.44	1.35	0.0002	0.0121	SMO	smoothened, frizzled class receptor	Multiple_Complex
TC0100008955.hg.1	12.21	13.05	-1.78	0.0002	0.0121	PKN2	protein kinase N2	Multiple_Complex
TC0100015364.hg.1	7.91	9.1	-2.28	0.0002	0.0122	TSPAN2	tetraspanin 2	Multiple_Complex
TC0700009232.hg.1	10.06	12.25	-4.57	0.0002	0.0122	BPGM	2,3-bisphosphoglycerate mutase	Coding
TC0800010524.hg.1	9.06	8.41	1.57	0.0002	0.0122	IMPAD1	inositol monophosphatase domain containing 1	Multiple_Complex
TC0100016044.hg.1	5.87	6.75	-1.84	0.0002	0.0123	FCRL5	Fc receptor-like 5	Multiple_Complex
TC0200016469.hg.1	8.34	7.74	1.52	0.0002	0.0123	ANXA4	annexin A4	Multiple_Complex
TC1600007931.hg.1	14.99	15.99	-2	0.0003	0.0124	LPCAT2	lysophosphatidylcholine acyltransferase 2	Multiple_Complex
TC0100010514.hg.1	7.82	9.43	-3.04	0.0003	0.0124	MPZL1	myelin protein zero-like 1	Multiple_Complex
TC0X00010670.hg.1	5.93	4.81	2.17	0.0003	0.0125	TMEM255A	transmembrane protein 255A	Multiple_Complex
TC1600009066.hg.1	8.61	9.68	-2.1	0.0003	0.0126	HAGH	hydroxyacylglutathione hydrolase	Multiple_Complex
TC2200007069.hg.1	7.43	9.76	-5.03	0.0003	0.0127	OSBP2	oxysterol binding protein 2	Multiple_Complex
TC1100012957.hg.1	12.75	11.01	3.34	0.0003	0.0127	TRIM22	tripartite motif containing 22	Multiple_Complex
TC1700007865.hg.1	6.29	5.66	1.55	0.0003	0.0127	CNP	2,3-cyclic nucleotide 3 phosphodiesterase	Multiple_Complex
TC1000008399.hg.1	5.68	7.09	-2.66	0.0003	0.0128	IFIT1B	interferon-induced protein with tetratricopeptide re	Coding
TC0500006744.hg.1	7.45	8.51	-2.09	0.0003	0.0128	ROPN1L	rhophilin associated tail protein 1-like	Multiple_Complex
TC0200014004.hg.1	9.89	10.35	-1.37	0.0003	0.0129	CCDC93	coiled-coil domain containing 93	Multiple_Complex
TC1500010747.hg.1	9.66	10.49	-1.78	0.0003	0.0129	RNF111	ring finger protein 111	Multiple_Complex
TC0X00011378.hg.1	3.48	3.93	-1.36	0.0003	0.013	RAB40A	RAB40A, member RAS oncogene family	Coding
TC0100012778.hg.1	14.04	13.13	1.87	0.0003	0.013	ENO1	enolase 1, (alpha)	Multiple_Complex
TC0400012795.hg.1	11.57	12.4	-1.77	0.0003	0.013	FIP1L1	factor interacting with PAPOLA and CPSF1	Multiple_Complex
TC1800009206.hg.1	10.21	9.51	1.63	0.0003	0.0131	TXNL4A	thioredoxin-like 4A	Multiple_Complex
TC2100007964.hg.1	7.97	8.73	-1.69	0.0003	0.0132	SYNJ1	synaptojanin 1	Multiple_Complex
TC0900011918.hg.1	13.73	12.94	1.72	0.0003	0.0132	FCN1	ficolin (collagen/fibrinogen domain containing) 1	Coding
TC2200007312.hg.1	9.69	8.64	2.07	0.0003	0.0132	LGALS1	lectin, galactoside-binding, soluble, 1	Multiple_Complex
TC2000008915.hg.1	7.08	6.49	1.51	0.0003	0.0133	AHCY	adenosylhomocysteinase	Multiple_Complex
TC0500013269.hg.1	4.69	5.3	-1.53	0.0003	0.0133	FAM153B	family with sequence similarity 153, member B	NonCoding
TC0200015425.hg.1	9.47	10.61	-2.2	0.0003	0.0133	TRAK2	trafficking protein, kinesin binding 2	Multiple_Complex
TC0700008857.hg.1	10.14	9.36	1.72	0.0003	0.0133	MDFIC	MyoD family inhibitor domain containing	Multiple_Complex
TC1200006700.hg.1	9.48	10.42	-1.92	0.0003	0.0133	FOXJ2	forkhead box J2	Multiple_Complex
TC1100011035.hg.1	9.52	10.21	-1.61	0.0003	0.0134	CPSF7	cleavage and polyadenylation specific factor 7	Multiple_Complex
TC0200016680.hg.1	5.73	6.23	-1.42	0.0003	0.0134	SERTAD2	SERTA domain containing 2	NonCoding
TC1000011798.hg.1	10.5	9.72	1.72	0.0003	0.0136	XPNPEP1	X-prolyl aminopeptidase (aminopeptidase P) 1, solub	Multiple_Complex
TC0200015012.hg.1	8.37	7.76	1.52	0.0003	0.0136	ATP5G3	ATP synthase, H+ transporting, mitochondrial Fo com	Multiple_Complex
TC0200011188.hg.1	6.13	5.5	1.54	0.0003	0.0136	AGAP1	ArfGAP with GTPase domain, ankyrin repeat and PH	Multiple_Complex
TC1400009481.hg.1	6.54	7.91	-2.58	0.0003	0.0136	PLEK2	pleckstrin 2	Multiple_Complex
TC1200006604.hg.1	8.56	9.7	-2.2	0.0003	0.0136	CD9	CD9 molecule	Multiple_Complex
TC1900011997.hg.1	4.78	4.33	1.37	0.0003	0.0137	CGB7	chorionic gonadotropin, beta polypeptide 7	Multiple_Complex
TSUnmapped000001!	4.39	4.91	-1.43	0.0003	0.0137	LRP6	LDL receptor related protein 6	Coding
TC1100009837.hg.1	4.6	5.3	-1.63	0.0003	0.0137	MRGPRE	MAS-related GPR, member E	Coding
TC0400012826.hg.1	10.95	11.6	-1.57	0.0003	0.0137	HERC3	HECT and RLD domain containing E3 ubiquitin protei	Multiple_Complex
TC1500008982.hg.1	7.37	8.22	-1.8	0.0003	0.0137	KATNBL1	katanin p80 subunit B-like 1	Multiple_Complex
TC0300006791.hg.1	12.18	12.91	-1.66	0.0003	0.0137	KAT2B	K(lysine) acetyltransferase 2B	Multiple_Complex
TC1900007386.hg.1	5.32	4.91	1.34	0.0003	0.0137	SSBP4	single stranded DNA binding protein 4	Multiple_Complex
TC0900010177.hg.1	6.81	7.67	-1.82	0.0003	0.0137	CNTNAP3B	Salzman2013 ANNOTATED, CDS, coding, INTERNAL, C	NonCoding
TC0X00007325.hg.1	7.21	6.43	1.72	0.0003	0.0137	RIBC1	RIB43A domain with coiled-coils 1	Multiple_Complex
TC2000006501.hg.1	11.03	12.35	-2.49	0.0003	0.0137	SIRPA	signal-regulatory protein alpha	Coding
TC0500008342.hg.1	9.55	10.29	-1.67	0.0003	0.0138	DCP2	decapping mRNA 2	Multiple_Complex
TC0700008565.hg.1	7.27	8.16	-1.85	0.0003	0.014	SLC12A9	solute carrier family 12, member 9	Multiple_Complex
TC1700010707.hg.1	8.56	7.32	2.35	0.0003	0.0143	DHX58	DEXH (Asp-Glu-X-His) box polypeptide 58	Multiple_Complex
TC2000008650.hg.1	5.63	6.15	-1.44	0.0003	0.0144	THBD	thrombomodulin	Coding
TC1600011428.hg.1	10.52	9.9	1.54	0.0003	0.0144	TERF2IP	telomeric repeat binding factor 2, interacting protein	Coding
TC0900009267.hg.1	7.24	6.75	1.4	0.0003	0.0147	SSNA1	Sjogren syndrome nuclear autoantigen 1	Multiple_Complex
TC1400010721.hg.1	7.87	7.16	1.64	0.0003	0.0152	PSME2; MIR7703	proteasome activator subunit 2; microRNA 7703	Multiple_Complex
TC1600008005.hg.1	10.26	8.78	2.8	0.0003	0.0153	ADGRG1	adhesion G protein-coupled receptor G1	Multiple_Complex
TC0300008705.hg.1	8.98	10.15	-2.24	0.0003	0.0154	ABTB1	ankyrin repeat and BTB (POZ) domain containing 1	Multiple_Complex
TC0900008207.hg.1	7.13	6.73	1.32	0.0004	0.0155	SEC61B	Sec61 translocon beta subunit	Multiple_Complex
TC0400009217.hg.1	8.41	9.67	-2.39	0.0004	0.0155	KLHL2	kelch-like family member 2	Multiple_Complex
TC0800007738.hg.1	13.39	14.41	-2.02	0.0004	0.0156	SDCBP	syndecan binding protein	Multiple_Complex
TC0300013795.hg.1	8.44	9.17	-1.66	0.0004	0.0156	OXNAD1	oxidoreductase NAD-binding domain containing 1	Multiple_Complex
TC1400008763.hg.1	8.82	7.41	2.66	0.0004	0.0157	GZMH	granzyme H	Coding
TC1300008179.hg.1	9.57	8.79	1.71	0.0004	0.0159	UPF3A	UPF3 regulator of nonsense transcripts homolog A (y	Multiple_Complex
TC0500008229.hg.1	6.9	6.24	1.58	0.0004	0.016	PAM	peptidylglycine alpha-amidating monooxygenase	Multiple_Complex
TC1400009701.hg.1	10.14	10.93	-1.74	0.0004	0.0162	AREL1	apoptosis resistant E3 ubiquitin protein ligase 1	Multiple_Complex
TC0100010252.hg.1	6.09	7.62	-2.9	0.0004	0.0162	FCER1A	Fc fragment of IgE, high affinity I, receptor for; alpha	Coding
TC1600006561.hg.1	8.27	8.88	-1.52	0.0004	0.0162	MAPK8IP3	mitogen-activated protein kinase 8 interacting protei	Multiple_Complex
TC0600007374.hg.1	10.58	9.68	1.87	0.0004	0.0162	HIST1H2AI	histone cluster 1, H2ai	Coding
TC1900007025.hg.1	8.88	10.14	-2.4	0.0004	0.0164	PLPPR2	phospholipid phosphatase related 2	Multiple_Complex
TC1600008454.hg.1	8.23	9.06	-1.78	0.0004	0.0165	GABARAPL2	GABA(A) receptor-associated protein like 2	Multiple_Complex
TC1400009973.hg.1	8.07	9.51	-2.71	0.0004	0.0165	RPS6KA5	ribosomal protein S6 kinase, 90kDa, polypeptide 5	Multiple_Complex

TC1400007975.hg.1	5.71	6.69	-1.97	0.0004	0.0165	C14orf159	chromosome 14 open reading frame 159	Multiple_Complex
TC1100007884.hg.1	9.92	9.36	1.47	0.0004	0.0165	SLC3A2	solute carrier family 3 (amino acid transporter heavy	Multiple_Complex
TC0800011611.hg.1	3.58	4.09	-1.42	0.0004	0.0165	TNFRSF11B	tumor necrosis factor receptor superfamily, member	Multiple_Complex
TC0X00009213.hg.1	5.43	4.85	1.5	0.0004	0.0165	MAP7D2	MAP7 domain containing 2	Multiple_Complex
TC0200010416.hg.1	6.38	4.86	2.87	0.0004	0.0165	SPATS2L	Transcript Identified by AceView, Entrez Gene ID(s) 2	Unassigned
TC0300009458.hg.1	8.29	9.12	-1.78	0.0004	0.0165	SEC62	SEC62 homolog, preprotein translocation factor	Multiple_Complex
TC0200016643.hg.1	7.72	8.51	-1.73	0.0004	0.0165	DNMT3A	DNA (cytosine-5-)-methyltransferase 3 alpha	Multiple_Complex
TC1200012679.hg.1	5.99	6.85	-1.81	0.0004	0.0165	ACSS3	acyl-CoA synthetase short-chain family member 3	NonCoding
TC1700008135.hg.1	7.33	6.87	1.38	0.0004	0.0165	TBX21	T-box 21	Multiple_Complex
TC0X00006623.hg.1	9.18	8.62	1.48	0.0004	0.0166	PRPS2	phosphoribosyl pyrophosphate synthetase 2	Multiple_Complex
TC0900011655.hg.1	9.86	11.25	-2.63	0.0004	0.0168	ZER1	zyg-11 related, cell cycle regulator	Multiple_Complex
TC2000007277.hg.1	12.63	11.98	1.57	0.0004	0.0168	RPN2	ribophorin II	Multiple_Complex
TC1100010897.hg.1	14.72	12.65	4.2	0.0004	0.0168	UBE2L6	ubiquitin-conjugating enzyme E2L 6	Multiple_Complex
TC1700007097.hg.1	7.91	8.76	-1.81	0.0004	0.0168	GID4	GID complex subunit 4 homolog	Multiple_Complex
TC1600010889.hg.1	11.8	11.22	1.49	0.0004	0.0168	KARS	lysyl-tRNA synthetase	Multiple_Complex
TC0200012608.hg.1	9.53	10.15	-1.53	0.0004	0.0168	PSME4	proteasome activator subunit 4	Multiple_Complex
TC1800007392.hg.1	3.26	3.51	-1.19	0.0004	0.0168	BOD1L2	biorientation of chromosomes in cell division 1-like 2	Coding
TC0600009362.hg.1	4.25	3.74	1.42	0.0004	0.0168	HEY2	hes-related family bHLH transcription factor with YRP	Multiple_Complex
TC0700012512.hg.1	7.94	8.97	-2.04	0.0004	0.0168	LRRC4	leucine rich repeat containing 4	Coding
TC0500012498.hg.1	10.61	9.72	1.86	0.0004	0.0168	ANXA6	annexin A6	Multiple_Complex
TC1200007654.hg.1	6.71	6.27	1.35	0.0004	0.0168	ATG101	autophagy related 101	Multiple_Complex
TC0300007474.hg.1	9.29	9.82	-1.45	0.0004	0.0168	DNAH1	dynein, axonemal, heavy chain 1	Multiple_Complex
TSUnmapped0000074	6.26	6.92	-1.58	0.0004	0.0169	RPS6KA1	ribosomal protein S6 kinase, 90kDa, polypeptide 1	NonCoding
TC0300013606.hg.1	3.75	3.29	1.38	0.0004	0.0169	TMEM44	transmembrane protein 44	Multiple_Complex
TC1000008716.hg.1	9.44	9.84	-1.31	0.0004	0.0169	GBF1	golgi brefeldin A resistant guanine nucleotide exchan	Multiple_Complex
TC1900011940.hg.1	15.78	14.81	1.96	0.0004	0.0169	ECH1	enoyl-CoA hydratase 1, peroxisomal	Multiple_Complex
TC0400010418.hg.1	10.04	10.89	-1.8	0.0004	0.017	RELL1	RELT-like 1	Multiple_Complex
TC0800012470.hg.1	13.6	14.67	-2.1	0.0004	0.017	TMEM71	transmembrane protein 71	Multiple_Complex
TC1500010776.hg.1	10.25	9.83	1.34	0.0004	0.017	IDH3A	isocitrate dehydrogenase 3 (NAD+) alpha	Multiple_Complex
TC1700010230.hg.1	8.22	7.61	1.52	0.0004	0.017	TIAF1; MYO18A	TGFB1-induced anti-apoptotic factor 1; myosin XVIIIA	Multiple_Complex
TC2000009975.hg.1	12.72	12.1	1.53	0.0004	0.0171	TMEM230	transmembrane protein 230	Multiple_Complex
TC1200009768.hg.1	9.47	10.28	-1.76	0.0004	0.0171	LPCAT3	lysophosphatidylcholine acyltransferase 3	Multiple_Complex
TC2000009957.hg.1	8.68	9.45	-1.71	0.0004	0.0172	LINC00266-1	long intergenic non-protein coding RNA 266-1	Multiple_Complex
TC1100012019.hg.1	8.68	9.77	-2.14	0.0004	0.0173	SESN3	sestrin 3	Multiple_Complex
TSUnmapped0000068	6.51	6.95	-1.35	0.0004	0.0174	MLXIP	MLX interacting protein	NonCoding
TC2000008653.hg.1	6.77	7.95	-2.25	0.0004	0.0174	CD93	CD93 molecule	Multiple_Complex
TC0800007040.hg.1	7.98	8.75	-1.7	0.0004	0.0174	ADAM28	ADAM metallopeptidase domain 28	Multiple_Complex
TC0100012734.hg.1	7.41	8.81	-2.64	0.0004	0.0174	TNFRSF9	tumor necrosis factor receptor superfamily, member	Coding
TC1900010538.hg.1	6.7	6.15	1.46	0.0004	0.0177	ZNF790	zinc finger protein 790	Coding
TC0300007257.hg.1	8.81	7.95	1.81	0.0004	0.0177	CCR5	chemokine (C-C motif) receptor 5 (gene/pseudogene	Multiple_Complex
TC0800008314.hg.1	13.23	12.69	1.46	0.0004	0.0177	MTDH	metadherin	Multiple_Complex
TC1100007394.hg.1	9.12	9.76	-1.55	0.0004	0.0177	CD82	CD82 molecule	Multiple_Complex
TC0100009475.hg.1	7.86	7.23	1.55	0.0004	0.0177	MAGI3	Jeck2013 ALT_ACCEPTOR, ALT_DONOR, coding, INTEI	Multiple_Complex
TC1800007518.hg.1	11.21	9.64	2.98	0.0004	0.0177	ZCCHC2	zinc finger, CCHC domain containing 2	Multiple_Complex
TC0100017519.hg.1	9.07	8.61	1.38	0.0005	0.0178	ACBD3	acyl-CoA binding domain containing 3	Multiple_Complex
TC0500013155.hg.1	11.32	12.72	-2.64	0.0005	0.0178	BASP1	brain abundant, membrane attached signal protein 1	Coding
TC1900009633.hg.1	8.46	7.73	1.67	0.0005	0.0178	S1PR5	sphingosine-1-phosphate receptor 5	Coding
TC1900007181.hg.1	10.45	9.63	1.76	0.0005	0.0178	TECR; MIR639	trans-2,3-enoyl-CoA reductase; microRNA 639	Multiple_Complex
TC0400007344.hg.1	4.57	4.04	1.44	0.0005	0.0178	UCHL1	ubiquitin C-terminal hydrolase L1	Multiple_Complex
TC0600014206.hg.1	7	6.54	1.37	0.0005	0.0178	TCP10L2	t-complex 10-like 2	Multiple_Complex
TC1400008355.hg.1	8.08	8.88	-1.73	0.0005	0.0178	RCOR1	REST corepressor 1	Multiple_Complex
TC1100009171.hg.1	6.15	6.84	-1.61	0.0005	0.0178	RNF214	ring finger protein 214	Multiple_Complex
TC2200009248.hg.1	7.31	8.54	-2.35	0.0005	0.018	KREMEN1	kringle containing transmembrane protein 1	Multiple_Complex
TC0100015196.hg.1	9.91	9.14	1.7	0.0005	0.018	PSMA5	proteasome subunit alpha 5	Multiple_Complex
TC0300008849.hg.1	7.84	7.36	1.39	0.0005	0.0181	UBA5	ubiquitin-like modifier activating enzyme 5	Multiple_Complex
TC0300007161.hg.1	8.16	8.62	-1.38	0.0005	0.0183	VIPR1	vasoactive intestinal peptide receptor 1	Multiple_Complex
TC1900008013.hg.1	9.46	8.81	1.57	0.0005	0.0183	PSMD8	proteasome 26S subunit, non-ATPase 8	Multiple_Complex
TC1000008908.hg.1	11.02	11.66	-1.57	0.0005	0.0183	SHOC2	SHOC2 leucine-rich repeat scaffold protein	Multiple_Complex
TSUnmapped0000035	8.18	7.64	1.45	0.0005	0.0183	SLC2A6	solute carrier family 2 (facilitated glucose transporter	Coding
TC1500010857.hg.1	8.32	11.15	-7.13	0.0005	0.0183	EPB42	erythrocyte membrane protein band 4.2	Multiple_Complex
TC0500008975.hg.1	7.23	6.64	1.51	0.0005	0.0185	HMHB1	histocompatibility (minor) HB-1	Multiple_Complex
TC0400011313.hg.1	13.69	15.78	-4.25	0.0005	0.0186	SNCA	synuclein alpha	Multiple_Complex
TC0400008318.hg.1	11.76	12.65	-1.85	0.0005	0.019	TET2	tet methylcytosine dioxygenase 2	Multiple_Complex
TC0700010046.hg.1	6.04	5.6	1.35	0.0005	0.0191	FTSJ2	FtsJ RNA methyltransferase homolog 2 (E. coli)	Multiple_Complex
TC0300008558.hg.1	11.52	10.21	2.46	0.0005	0.0191	DTX3L	deltex 3 like, E3 ubiquitin ligase	Multiple_Complex
TC0200008230.hg.1	12.79	11.66	2.2	0.0005	0.0191	TMSB10	thymosin beta 10	Multiple_Complex
TC1900011776.hg.1	4.68	4.15	1.44	0.0005	0.0193	KCNJ14	potassium channel, inwardly rectifying subfamily J, m	Multiple_Complex
TC0600014202.hg.1	5.93	6.61	-1.6	0.0005	0.0194	FGFR1OP	FGFR1 oncogene partner	Multiple_Complex
TC0100006874.hg.1	9.21	8.76	1.36	0.0005	0.0195	PLOD1	procollagen-lysine, 2-oxoglutarate 5-dioxygenase 1	Multiple_Complex
TC0700008868.hg.1	12	11.38	1.53	0.0005	0.0195	TES	testin LIM domain protein	Multiple_Complex
TC0500013010.hg.1	7.4	7.04	1.29	0.0005	0.0196	NHP2	NHP2 ribonucleoprotein	Multiple_Complex
TC1900011153.hg.1	6.98	6.39	1.5	0.0005	0.0196	RRAS	related RAS viral (r-ras) oncogene homolog	Multiple_Complex

TC1200009749.hg.1	4.22	4.69	-1.38	0.0005	0.0196	PIANP	PILR alpha associated neural protein	Coding
TC0600011354.hg.1	5.55	6.21	-1.58	0.0005	0.0198	TRIM10	tripartite motif containing 10	Coding
TC0200008351.hg.1	6.08	7.18	-2.15	0.0005	0.0198	RPIA	ribose 5-phosphate isomerase A	Multiple_Complex
TC1000010273.hg.1	3.3	3.63	-1.26	0.0005	0.0198	NRP1	neuropilin 1	Multiple_Complex
TC1500008309.hg.1	5.48	4.9	1.49	0.0005	0.0199	ZNF774	zinc finger protein 774	Multiple_Complex
TC1100008054.hg.1	8.94	9.78	-1.79	0.0005	0.0201	PACS1	phosphofurin acidic cluster sorting protein 1	Multiple_Complex
TC0200016412.hg.1	5.06	5.54	-1.4	0.0005	0.0202	MFSD2B	major facilitator superfamily domain containing 2B	Multiple_Complex
TC0800011334.hg.1	9.84	9.12	1.65	0.0005	0.0202	KLF10	Kruppel-like factor 10	Multiple_Complex
TC1400008333.hg.1	14.47	13.86	1.53	0.0005	0.0202	PPP2R5C	protein phosphatase 2, regulatory subunit B, gamma	Multiple_Complex
TC1900006730.hg.1	6.42	7.21	-1.73	0.0005	0.0203	KDM4B	lysine (K)-specific demethylase 4B	Multiple_Complex
TC1900011994.hg.1	4.59	4.33	1.2	0.0006	0.0204	CGB1	chorionic gonadotropin, beta polypeptide 1	Coding
TC1600010617.hg.1	5.46	4.95	1.42	0.0006	0.0204	KIAA0895L	KIAA0895-like	Multiple_Complex
TC0400011013.hg.1	10.88	12.76	-3.67	0.0006	0.0204	PPBP	pro-platelet basic protein	Coding
TC1200011471.hg.1	7.28	7.81	-1.44	0.0006	0.0204	POC1B; POC1B-G	POC1 centriolar protein B; POC1B-GALNT4 readthrou	Multiple_Complex
TC0100018539.hg.1	6.39	7.1	-1.64	0.0006	0.0204	LHX4-AS1; ACBD6	LHX4 antisense RNA 1; acyl-CoA binding domain cont	NonCoding
TC1200012654.hg.1	6.5	6.18	1.25	0.0006	0.0204	TSFM	Ts translation elongation factor, mitochondrial	Multiple_Complex
TC1200009196.hg.1	7.09	8.18	-2.13	0.0006	0.0204	MLXIP	MLX interacting protein	Multiple_Complex
TC0700010965.hg.1	5.96	5.22	1.67	0.0006	0.0204	IGFBP3	insulin like growth factor binding protein 3	Multiple_Complex
TC0900010147.hg.1	5.67	6.62	-1.93	0.0006	0.0204	CNTNAP3B	contactin associated protein-like 3B	Multiple_Complex
TC0300013828.hg.1	10.47	11.01	-1.46	0.0006	0.0205	RBM6	RNA binding motif protein 6	Multiple_Complex
TC1700009385.hg.1	10.59	11.48	-1.85	0.0006	0.0205	SLC43A2	solute carrier family 43 (amino acid system L transpo	Multiple_Complex
TC1800009203.hg.1	8.53	7.68	1.8	0.0006	0.0205	PQLC1	PQ loop repeat containing 1	Multiple_Complex
TC0X00007557.hg.1	9.92	10.65	-1.65	0.0006	0.0205	MED12	mediator complex subunit 12	Multiple_Complex
TC2200009357.hg.1	9.45	10.22	-1.71	0.0006	0.0205	PACSIN2	protein kinase C and casein kinase substrate in neurc	Multiple_Complex
TC1100008761.hg.1	5.72	5.24	1.39	0.0006	0.0205	FAT3	FAT atypical cadherin 3	Multiple_Complex
TC0800006966.hg.1	6.92	6.39	1.44	0.0006	0.0206	NPM2	nucleophosmin/nucleoplasmin 2	Multiple_Complex
TC0600007278.hg.1	6.47	5.57	1.87	0.0006	0.0209	HIST1H2BE	histone cluster 1, H2be	Coding
TC0Y00006890.hg.1	6.42	6	1.34	0.0006	0.0209	ZBED1	zinc finger, BED-type containing 1	Multiple_Complex
TC0200014991.hg.1	9.86	10.54	-1.6	0.0006	0.0209	GPR155	G protein-coupled receptor 155	Multiple_Complex
TC0200009813.hg.1	6.51	5.95	1.47	0.0006	0.0209	PSMD14	proteasome 26S subunit, non-ATPase 14	Multiple_Complex
TC1200006555.hg.1	13.84	12.95	1.85	0.0006	0.021	CCND2	cyclin D2	Multiple_Complex
TC0200012652.hg.1	7.65	6.97	1.61	0.0006	0.0211	PNPT1	polyribonucleotide nucleotidyltransferase 1	Multiple_Complex
TC1000009810.hg.1	6.51	7.48	-1.97	0.0006	0.0211	NUDT5	nudix hydrolase 5	Multiple_Complex
TC1900010715.hg.1	6.42	5.97	1.37	0.0006	0.0211	NUMBL	numb homolog (Drosophila)-like	Multiple_Complex
TC0500012781.hg.1	10.46	8.7	3.37	0.0006	0.0212	DOCK2	Memczak2013 ANTISENSE, CDS, coding, INTERNAL be	NonCoding
TC2200009352.hg.1	8.65	9.31	-1.58	0.0006	0.0212	LOC400927; CSNI	TPTE and PTEN homologous inositol lipid phosphatas	Multiple_Complex
TC1600010651.hg.1	9.8	11.67	-3.67	0.0006	0.0215	RANBP10	RAN binding protein 10	Multiple_Complex
TC1100012469.hg.1	5.54	5.05	1.4	0.0006	0.0215	SCN4B	sodium channel, voltage gated, type IV beta subunit	Multiple_Complex
TC0400012824.hg.1	5.18	4.72	1.37	0.0006	0.0215	HERC3	HECT and RLD domain containing E3 ubiquitin protei	NonCoding
TC1700006773.hg.1	6.26	7.1	-1.79	0.0006	0.0215	TMEM88	transmembrane protein 88	Coding
TC0100008094.hg.1	11.26	10.8	1.38	0.0006	0.0215	RNF220	ring finger protein 220	Multiple_Complex
TC1100007257.hg.1	9.87	11.33	-2.75	0.0006	0.0215	CAT	catalase	Multiple_Complex
TC0100016072.hg.1	4	4.49	-1.4	0.0006	0.0215	OR6P1	olfactory receptor, family 6, subfamily P, member 1	Coding
TC1500009636.hg.1	12.35	10.92	2.7	0.0006	0.0217	ANXA2	annexin A2	Multiple_Complex
TC1900010391.hg.1	8.8	8.26	1.46	0.0006	0.0217	PEPD	peptidase D	Multiple_Complex
TC1600011361.hg.1	4.24	4.93	-1.6	0.0006	0.0217	ACSM3	acyl-CoA synthetase medium-chain family member 3	NonCoding
TSUnmapped000000!	8.19	9.54	-2.55	0.0006	0.0217	MLXIP	MLX interacting protein	Coding
TC0100008056.hg.1	3.41	3.89	-1.4	0.0006	0.0218	MPL	MPL proto-oncogene, thrombopoietin receptor	Coding
TC1000007678.hg.1	6.67	5.93	1.68	0.0006	0.0218	MTRNR2L5	MT-RNR2-like 5	Coding
TC0100017022.hg.1	3.56	3.91	-1.27	0.0006	0.0218	GOLT1A	golgi transport 1A	Multiple_Complex
TC1100011161.hg.1	9.04	8.32	1.64	0.0006	0.0219	TRMT112	tRNA methyltransferase 11-2 homolog (S. cerevisiae)	Multiple_Complex
TC0900008933.hg.1	8.25	7.53	1.65	0.0006	0.0219	TOR1B	torsin family 1, member B (torsin B)	Multiple_Complex
TC1600011570.hg.1	6.86	6.18	1.6	0.0006	0.0219	CMC2	C-x(9)-C motif containing 2	Multiple_Complex
TC1700008769.hg.1	8.22	9.57	-2.55	0.0006	0.022	KCNJ2	potassium channel, inwardly rectifying subfamily J, m	Coding
TC2000009588.hg.1	6.62	5.9	1.65	0.0006	0.022	MTRNR2L3	MT-RNR2-like 3	Coding
TC1600007235.hg.1	8.17	7.09	2.12	0.0006	0.022	PLK1	polo-like kinase 1	Multiple_Complex
TC0100013696.hg.1	10.33	9.82	1.42	0.0006	0.022	PSMB2	proteasome subunit beta 2	Multiple_Complex
TC1200012790.hg.1	9.37	10.33	-1.96	0.0006	0.022	KMT2D	lysine (K)-specific methyltransferase 2D	Multiple_Complex
TC0100016971.hg.1	16.89	18	-2.17	0.0006	0.022	ADIPOR1	adiponectin receptor 1	Multiple_Complex
TC2000006899.hg.1	10.45	11.17	-1.65	0.0007	0.0222	XRN2	5-3 exoribonuclease 2	Multiple_Complex
TC1900007831.hg.1	5.85	5.36	1.41	0.0007	0.0223	HPN	hepsin	Multiple_Complex
TC0500011523.hg.1	7.22	6.16	2.09	0.0007	0.0223	LNPEP	Memczak2013 ANTISENSE, coding, INTERNAL, UTR3 k	NonCoding
TC1100012685.hg.1	5.62	6.07	-1.37	0.0007	0.0223	VSIG2	V-set and immunoglobulin domain containing 2	Coding
TC0200012038.hg.1	5.5	4.8	1.63	0.0007	0.0225	OTOF	otoferlin	Multiple_Complex
TC1900007115.hg.1	6.25	6.73	-1.4	0.0007	0.0225	NFIX	nuclear factor I/X (CCAAT-binding transcription facto	Multiple_Complex
TC0100013076.hg.1	8.44	10.05	-3.06	0.0007	0.0226	PADI2	peptidyl arginine deiminase, type II	Multiple_Complex
TC0800007384.hg.1	5.5	4.94	1.47	0.0007	0.0226	IDO2	indoleamine 2,3-dioxygenase 2	Multiple_Complex
TC0900009253.hg.1	8.8	8.52	1.21	0.0007	0.0226	C9orf142	chromosome 9 open reading frame 142	Multiple_Complex
TC2000008267.hg.1	13.89	14.83	-1.91	0.0007	0.0226	RASSF2	Ras association (RalGDS/AF-6) domain family membe	Multiple_Complex
TC0400011202.hg.1	8.81	9.48	-1.59	0.0007	0.0228	COQ2	coenzyme Q2 4-hydroxybenzoate polyprenyltransfer	Multiple_Complex
TC0300010775.hg.1	17.13	15.66	2.77	0.0007	0.0228	CX3CR1	chemokine (C-X3-C motif) receptor 1	Coding
TC1200011936.hg.1	9.53	10.37	-1.8	0.0007	0.0229	ATXN2	ataxin 2	Multiple_Complex

TC0800010252.hg.1	8.59	11.11	-5.74	0.0007	0.0229	ANK1	ankyrin 1, erythrocytic	Multiple_Complex
TC11_KI270721v1_ra	5.48	5.07	1.32	0.0007	0.0229	MRPL23; LOC102	Homo sapiens mitochondrial ribosomal protein L23 (	Coding
TC0300013985.hg.1	6.35	5.89	1.37	0.0007	0.0229	TUSC2	tumor suppressor candidate 2	Multiple_Complex
TC0300006677.hg.1	13.81	14.6	-1.72	0.0007	0.023	SLC6A6	solute carrier family 6 (neurotransmitter transporter)	Multiple_Complex
TC0200007015.hg.1	13.15	12.27	1.84	0.0007	0.023	RAB10	RAB10, member RAS oncogene family	Multiple_Complex
TC0300008385.hg.1	6.45	5.93	1.43	0.0007	0.023	TIGIT	T-cell immunoreceptor with Ig and ITIM domains	Multiple_Complex
TC1900010632.hg.1	8.63	8.08	1.46	0.0007	0.023	SIRT2	sirtuin 2	Multiple_Complex
TC1900007313.hg.1	8.99	8.38	1.52	0.0007	0.023	USE1	unconventional SNARE in the ER 1 homolog (S. cerev	Multiple_Complex
TC1100009434.hg.1	5.5	4.99	1.43	0.0007	0.023	DDX25	DEAD (Asp-Glu-Ala-Asp) box helicase 25	Multiple_Complex
TC1400010763.hg.1	6.37	5.84	1.45	0.0007	0.0231	TMEM229B	transmembrane protein 229B	Coding
TC0300010632.hg.1	7.23	8.18	-1.92	0.0007	0.0231	OSBPL10	oxysterol binding protein-like 10	Multiple_Complex
TC0500012948.hg.1	5.67	5.28	1.32	0.0007	0.0233	SNCB	synuclein beta	Multiple_Complex
TC1400007098.hg.1	12.54	12.09	1.37	0.0007	0.0233	ARF6	ADP-ribosylation factor 6	Multiple_Complex
TC0500013236.hg.1	6.17	6.48	-1.24	0.0007	0.0233	FAM53C	family with sequence similarity 53, member C	NonCoding
TC0600007285.hg.1	8.96	8.11	1.8	0.0007	0.0233	HIST1H2AE	histone cluster 1, H2ae	Coding
TC0X00007363.hg.1	6.9	6.49	1.34	0.0007	0.0233	MAGED2	MAGE family member D2	Multiple_Complex
TC0900009882.hg.1	14.95	16.83	-3.69	0.0007	0.0233	DCAF12	DDB1 and CUL4 associated factor 12	Multiple_Complex
TC1300007167.hg.1	12.1	11.31	1.73	0.0007	0.0233	PHF11	PHD finger protein 11	Multiple_Complex
TC1600007957.hg.1	16.17	13.82	5.1	0.0007	0.0234	MT2A	metallothionein 2A	Multiple_Complex
TC1900008558.hg.1	6.88	8	-2.17	0.0007	0.0234	MED25; MIR6800	mediator complex subunit 25; microRNA 6800	Multiple_Complex
TC0800006968.hg.1	5.28	4.89	1.31	0.0007	0.0234	FGF17	fibroblast growth factor 17	Multiple_Complex
TC1000011736.hg.1	8.06	8.79	-1.66	0.0007	0.0234	OBFC1	oligonucleotide/oligosaccharide-binding fold contain	Multiple_Complex
TC1200009913.hg.1	15.27	17.08	-3.5	0.0007	0.0234	YBX3	Y box binding protein 3	Multiple_Complex
TC1700011032.hg.1	6.45	6.05	1.32	0.0007	0.0235	GNGT2	guanine nucleotide binding protein (G protein), gamı	Coding
TC0600012240.hg.1	13	13.73	-1.65	0.0007	0.0236	LMBRD1	LMBR1 domain containing 1	Multiple_Complex
TC1200008683.hg.1	14.15	14.86	-1.63	0.0007	0.0236	CHST11	carbohydrate (chondroitin 4) sulfotransferase 11	Multiple_Complex
TC0700012797.hg.1	12.79	11.51	2.42	0.0007	0.0237	PARP12	poly(ADP-ribose) polymerase family member 12	Multiple_Complex
TC0200007047.hg.1	3.9	3.54	1.28	0.0007	0.0239	DPYSL5	dihydropyrimidinase-like 5	Multiple_Complex
TC0100013561.hg.1	4.65	4.17	1.4	0.0007	0.0239	FABP3	fatty acid binding protein 3, muscle and heart	Multiple_Complex
TC0600014256.hg.1	9.28	9.89	-1.53	0.0007	0.0239	GABBR1	gamma-aminobutyric acid (GABA) B receptor, 1	Multiple_Complex
TC0100006862.hg.1	7.38	6.38	2	0.0008	0.024	FBXO6	F-box protein 6	Multiple_Complex
TC0900008150.hg.1	8.59	10.32	-3.31	0.0008	0.0241	TMOD1	tropomodulin 1	Multiple_Complex
TC2100007039.hg.1	7.47	8	-1.44	0.0008	0.0242	DOPEY2	dopey family member 2	Multiple_Complex
TC0700013512.hg.1	9.65	9.2	1.37	0.0008	0.0242	CCZ1B	CCZ1 homolog B, vacuolar protein trafficking and bio	Multiple_Complex
TC1200010983.hg.1	7.13	8.03	-1.86	0.0008	0.0242	AVIL	advillin	Multiple_Complex
TC0600011508.hg.1	11.39	10.58	1.76	0.0008	0.0242	TAP1	transporter 1, ATP-binding cassette, sub-family B (MI	Multiple_Complex
TC0100007291.hg.1	6.52	5.91	1.53	0.0008	0.0243	C1QC	complement component 1, q subcomponent, C chair	Coding
TC1900011033.hg.1	12.26	11.29	1.96	0.0008	0.0243	NAPA	N-ethylmaleimide-sensitive factor attachment protei	Multiple_Complex
TC0700013435.hg.1	5.73	6.15	-1.34	0.0008	0.0243	CDHR3	cadherin-related family member 3	NonCoding
TC0300010982.hg.1	7.92	8.66	-1.67	0.0008	0.0243	SCAP	SREBF chaperone	Multiple_Complex
TC1200011993.hg.1	8.73	9.4	-1.6	0.0008	0.0244	SLC8B1	solute carrier family 8 (sodium/lithium/calcium excha	Multiple_Complex
TC1900010798.hg.1	6.75	6.2	1.46	0.0008	0.0244	PAFAH1B3	platelet-activating factor acetylhydrolase 1b, catalyti	Multiple_Complex
TC0100013275.hg.1	5.04	5.44	-1.31	0.0008	0.0245	ZNF436	zinc finger protein 436	Coding
TC1700010686.hg.1	6.6	5.73	1.84	0.0008	0.0245	JUP	junction plakoglobin	Multiple_Complex
TC0700013050.hg.1	5.14	5.8	-1.59	0.0008	0.0245	KCNH2	potassium channel, voltage gated eag related subfan	Multiple_Complex
TC2200007273.hg.1	13.6	14.9	-2.45	0.0008	0.0247	CSF2RB	colony stimulating factor 2 receptor, beta, low-affinit	Multiple_Complex
TC2000006818.hg.1	6.24	5.67	1.48	0.0008	0.0248	PET117; CSRP2BP	PET117 homolog; CSRP2 binding protein	Multiple_Complex
TC0X00008848.hg.1	5.84	5.36	1.39	0.0008	0.0249	CTAG1B; CTAG1A	cancer/testis antigen 1B; cancer/testis antigen 1A	Coding
TC0300013420.hg.1	8.91	10.22	-2.49	0.0008	0.0249	IGF2BP2	insulin-like growth factor 2 mRNA binding protein 2	Multiple_Complex
TC1400010068.hg.1	9.6	10.49	-1.85	0.0008	0.025	DICER1	dicer 1, ribonuclease type III	Multiple_Complex
TC0600011546.hg.1	9.29	8.62	1.59	0.0008	0.025	CUTA	cutA divalent cation tolerance homolog (E. coli)	Multiple_Complex
TC1600007531.hg.1	11.02	12.02	-2	0.0008	0.0251	ITGAX	integrin alpha X	Multiple_Complex
TC0100010664.hg.1	6.65	7.33	-1.61	0.0008	0.0251	ZBTB37	zinc finger and BTB domain containing 37	Multiple_Complex
TC0200014170.hg.1	10.59	11.44	-1.81	0.0008	0.0251	MAP3K2	mitogen-activated protein kinase kinase kinase 2	Multiple_Complex
TC0100015206.hg.1	6.7	6.23	1.39	0.0008	0.0252	GNAT2	guanine nucleotide binding protein (G protein), alpha	Coding
TC0700013441.hg.1	10.31	11.48	-2.26	0.0008	0.0258	IFRD1	interferon-related developmental regulator 1	Multiple_Complex
TC1100008002.hg.1	7.13	6.45	1.59	0.0008	0.0259	MRPL49	mitochondrial ribosomal protein L49	Multiple_Complex
TC1200008434.hg.1	4.85	5.33	-1.39	0.0008	0.0259	C12orf74	chromosome 12 open reading frame 74	Coding
TC1900011942.hg.1	7.76	7.14	1.54	0.0009	0.0261	SARS2	seryl-tRNA synthetase 2, mitochondrial	Multiple_Complex
TC1900006968.hg.1	7.5	6.78	1.65	0.0009	0.0261	C19orf66	chromosome 19 open reading frame 66	Multiple_Complex
TC1400007033.hg.1	8.21	8.63	-1.34	0.0009	0.0261	FAM179B	family with sequence similarity 179, member B	Multiple_Complex
TC1900009669.hg.1	5.44	5.63	-1.15	0.0009	0.0262	KANK2	KN motif and ankyrin repeat domains 2	Multiple_Complex
TC0600011483.hg.1	10.33	11.05	-1.65	0.0009	0.0263	PBX2	pre-B-cell leukemia homeobox 2	Multiple_Complex
TC0100012401.hg.1	5.21	5.95	-1.66	0.0009	0.0263	OR4F29; OR4F3; (	olfactory receptor, family 4, subfamily F, member 29;	Coding
TC0100012410.hg.1	5.21	5.95	-1.66	0.0009	0.0263	OR4F16; OR4F29	olfactory receptor, family 4, subfamily F, member 16;	Coding
TC0300013447.hg.1	7.88	7.46	1.34	0.0009	0.0263	RFC4	replication factor C subunit 4	Multiple_Complex
TC0200010448.hg.1	14.9	17.68	-6.87	0.0009	0.0265	STRADB	STE20-related kinase adaptor beta	Multiple_Complex
TC1200008525.hg.1	5.55	5.18	1.29	0.0009	0.0265	SNRPF	small nuclear ribonucleoprotein polypeptide F	Multiple_Complex
TC0400010449.hg.1	8.87	9.66	-1.74	0.0009	0.0265	TLR10	toll-like receptor 10	Multiple_Complex
TC1000008397.hg.1	13.26	10.08	9.08	0.0009	0.0267	IFIT3	interferon-induced protein with tetratricopeptide re	Coding
TC0200015402.hg.1	10.92	11.95	-2.03	0.0009	0.0268	FAM126B	family with sequence similarity 126, member B	Multiple_Complex
TC2000008001.hg.1	7.97	7.4	1.48	0.0009	0.0268	ADRM1	adhesion regulating molecule 1	Multiple_Complex

TC0900006782.hg.1	7.51	8	-1.41	0.0009	0.0269	FOCAD	focadhesin	Multiple_Complex
TC1500009736.hg.1	13.24	12.46	1.72	0.0009	0.0269	PPIB	peptidylprolyl isomerase B (cyclophilin B)	Multiple_Complex
TC0400010940.hg.1	9.1	10.4	-2.45	0.0009	0.027	SULT1B1	sulfotransferase family 1B member 1	Coding
TC0X00007501.hg.1	7.89	7.45	1.35	0.0009	0.0271	YIPF6	Yip1 domain family member 6	Multiple_Complex
TC1500007546.hg.1	5.65	6.22	-1.48	0.0009	0.0274	SNX22	sorting nexin 22	Multiple_Complex
TC0400011283.hg.1	9.37	8.72	1.56	0.0009	0.0275	PPM1K	protein phosphatase, Mg2+/Mn2+ dependent, 1K	Multiple_Complex
TC0300012813.hg.1	8.81	10.18	-2.59	0.0009	0.0275	P2RY14	purinergic receptor P2Y, G-protein coupled, 14	Coding
TC0600006993.hg.1	10.69	10.17	1.44	0.0009	0.0275	NOL7	nucleolar protein 7	Multiple_Complex
TC1300010036.hg.1	3.68	4.19	-1.42	0.0009	0.0277	LINC00598	long intergenic non-protein coding RNA 598	NonCoding
TC0200016668.hg.1	6.17	6.7	-1.44	0.0009	0.0278	GPR75	G protein-coupled receptor 75	Coding
TC1500006948.hg.1	5.21	5.74	-1.45	0.0009	0.0278	PHGR1	proline/histidine/glycine-rich 1	Multiple_Complex
TC1700008549.hg.1	7.23	7.64	-1.33	0.0009	0.0278	TANC2	tetratricopeptide repeat, ankyrin repeat and coiled-c	Multiple_Complex
TC0500007663.hg.1	10.44	9.98	1.38	0.0009	0.0278	SLC30A5	solute carrier family 30 (zinc transporter), member 5	Multiple_Complex
TC0900011597.hg.1	9.64	10.56	-1.88	0.0009	0.0279	SH2D3C	SH2 domain containing 3C	Multiple_Complex
TC1100011014.hg.1	6.13	6.9	-1.71	0.001	0.0279	SLC15A3	solute carrier family 15 (oligopeptide transporter), m	Multiple_Complex
TC1900007447.hg.1	9.92	9.31	1.53	0.001	0.0279	ZNF101	zinc finger protein 101	Multiple_Complex
TC0600014328.hg.1	11.49	10.49	2	0.001	0.0282	OSTM1	osteopetrosis associated transmembrane protein 1	Multiple_Complex
TC1100011126.hg.1	6.61	6.21	1.32	0.001	0.0282	PLA2G16	phospholipase A2, group XVI	Multiple_Complex
TC0100018280.hg.1	13.73	12.84	1.85	0.001	0.0282	FAM231D; LINC0(	family with sequence similarity 231, member D; long	Multiple_Complex
TC2000009198.hg.1	5.17	4.62	1.46	0.001	0.0283	ADA	adenosine deaminase	Multiple_Complex
TC1700009318.hg.1	10.42	11.64	-2.33	0.001	0.0283	FAM101B	family with sequence similarity 101, member B	Multiple_Complex
TC0600009144.hg.1	7.92	8.61	-1.61	0.001	0.0283	KIAA1919	KIAA1919	Multiple_Complex
TC1600007967.hg.1	6.22	5.23	1.98	0.001	0.0283	MT1IP	metallothionein 1I, pseudogene	Multiple_Complex
TC1100007978.hg.1	12.39	11.84	1.47	0.001	0.0283	MEN1	Jeck2013 ANTISENSE, CDS, coding, INTERNAL, OVCOD	NonCoding
TC0600008050.hg.1	12.17	13.14	-1.96	0.001	0.0283	UBR2	ubiquitin protein ligase E3 component n-recognin 2	Multiple_Complex
TC1300006847.hg.1	7.24	6.79	1.36	0.001	0.0283	RFC3	replication factor C subunit 3	Multiple_Complex
TC0700012111.hg.1	4.14	4.8	-1.58	0.001	0.0283	FBXL13	F-box and leucine-rich repeat protein 13	Multiple_Complex
TC0900010804.hg.1	6.29	5.94	1.27	0.001	0.0284	IPPK	inositol 1,3,4,5,6-pentakisphosphate 2-kinase	Multiple_Complex
TC0100011855.hg.1	11.73	12.91	-2.27	0.001	0.0284	GUK1	guanylate kinase 1	Multiple_Complex
TC0400011014.hg.1	6.91	7.92	-2.03	0.001	0.0285	CXCL5	chemokine (C-X-C motif) ligand 5	Multiple_Complex
TC0400010048.hg.1	6.35	6.91	-1.47	0.001	0.0285	ZNF518B	zinc finger protein 518B	Multiple_Complex
TC0500011935.hg.1	6.26	6.91	-1.57	0.001	0.0286	3-Mar	membrane associated ring finger 3	Multiple_Complex
TC0100006483.hg.1	6.49	5.55	1.91	0.001	0.0286	ISG15	ISG15 ubiquitin-like modifier	Multiple_Complex
TC1900008566.hg.1	7.94	8.48	-1.46	0.001	0.0286	SIGLEC16	sialic acid binding Ig-like lectin 16 (gene/pseudogene	Multiple_Complex
TC1000009065.hg.1	7.02	7.7	-1.61	0.001	0.0286	FAM45A	Transcript Identified by AceView, Entrez Gene ID(s) 4	Unassigned
TC0100009969.hg.1	4.93	4.31	1.54	0.001	0.0286	RIIAD1	regulatory subunit of type II PKA R-subunit (RIIa) don	Multiple_Complex
TC1900006896.hg.1	13.88	13.36	1.44	0.001	0.0286	HNRNPM	heterogeneous nuclear ribonucleoprotein M	Multiple_Complex
TC1900009755.hg.1	5.24	5.95	-1.64	0.001	0.0286	KLF1	Kruppel-like factor 1 (erythroid)	Multiple_Complex
TC2000007859.hg.1	13.03	14.94	-3.76	0.001	0.029	RBM38	RNA binding motif protein 38	Multiple_Complex
TC0100006881.hg.1	14.72	13.58	2.2	0.001	0.029	TNFRSF1B; MIR4(	tumor necrosis factor receptor superfamily, member	Multiple_Complex
TC0500008855.hg.1	9.76	9.23	1.44	0.001	0.0291	SLC35A4	solute carrier family 35, member A4	Multiple_Complex
TC1200010642.hg.1	3.81	4.25	-1.36	0.001	0.0292	FAIM2	Fas apoptotic inhibitory molecule 2	Coding
TC0100016678.hg.1	13.26	14.55	-2.45	0.001	0.0292	FAM129A	family with sequence similarity 129, member A	Multiple_Complex
TC0600007282.hg.1	7.24	6.45	1.72	0.001	0.0292	HIST1H2BF	histone cluster 1, H2bf	Coding
TC0600011227.hg.1	6.79	5.8	1.99	0.001	0.0293	HIST1H4K	histone cluster 1, H4k	Coding
TSUnmapped0000048	8.97	8.53	1.35	0.001	0.0293	SURF4	surfeit 4	NonCoding
TC0200008063.hg.1	8.18	7.67	1.43	0.001	0.0294	DGUOK	deoxyguanosine kinase	Multiple_Complex
TC1600010599.hg.1	7.16	6.36	1.74	0.001	0.0294	NAE1	NEDD8 activating enzyme E1 subunit 1	Multiple_Complex
TC0100016360.hg.1	9.21	9.96	-1.68	0.001	0.0294	SELL	Memczak2013 ALT_ACCEPTOR, ALT_DONOR, coding,	NonCoding
TC0500009652.hg.1	9.43	8.94	1.4	0.001	0.0294	HNRNPAB	heterogeneous nuclear ribonucleoprotein A/B	Multiple_Complex
TC2000008746.hg.1	6.81	6.45	1.28	0.0011	0.0295	NINL	ninein-like	Multiple_Complex
TC1500008114.hg.1	8.96	9.5	-1.45	0.0011	0.0295	TM6SF1	transmembrane 6 superfamily member 1	Multiple_Complex
TC1200012758.hg.1	6.65	7.37	-1.65	0.0011	0.0295	TAS2R14	taste receptor, type 2, member 14	Coding
TC1200010601.hg.1	8.93	8.37	1.47	0.0011	0.0295	FKBP11; ARF3	FK506 binding protein 11; ADP-ribosylation factor 3	Multiple_Complex
TC1800008333.hg.1	8.34	7.74	1.52	0.0011	0.0295	DHFR; DHFRP1	dihydrofolate reductase; dihydrofolate reductase pse	Multiple_Complex
TC0X00006891.hg.1	9.2	10.03	-1.78	0.0011	0.0296	GK	glycerol kinase	Multiple_Complex
TC0900009414.hg.1	8.32	8.93	-1.53	0.0011	0.0297	RFX3	regulatory factor X, 3 (influences HLA class II expressi	Multiple_Complex
TC0400011748.hg.1	7.19	6.16	2.05	0.0011	0.0298	CCNA2	cyclin A2	Multiple_Complex
TC0700010185.hg.1	11.42	13.07	-3.13	0.0011	0.0298	EIF2AK1	eukaryotic translation initiation factor 2-alpha kinase	Multiple_Complex
TC0100018233.hg.1	6.31	6.8	-1.4	0.0011	0.0298	FPGT	fucose-1-phosphate guanylyltransferase	Coding
TC0200009548.hg.1	8.17	8.69	-1.43	0.0011	0.0298	ARHGAP15	Rho GTPase activating protein 15	NonCoding
TC1200007595.hg.1	9.52	9.05	1.39	0.0011	0.0298	SMARCD1	SWI/SNF related, matrix associated, actin dependent	Multiple_Complex
TC1300009330.hg.1	10.35	10.84	-1.4	0.0011	0.0299	RBM26	RNA binding motif protein 26	Multiple_Complex
TC1200010908.hg.1	9.8	8.31	2.82	0.0011	0.0299	STAT2	signal transducer and activator of transcription 2	Multiple_Complex
TC1700011128.hg.1	10.53	11.13	-1.51	0.0011	0.03	SPAG9	sperm associated antigen 9	Multiple_Complex
TC0400012757.hg.1	11.97	11.14	1.78	0.0011	0.03	RNF4	ring finger protein 4	Multiple_Complex
TC0200007144.hg.1	9.06	9.73	-1.6	0.0011	0.03	LCLAT1	lysocardiolipin acyltransferase 1	Multiple_Complex
TC1000007574.hg.1	5.88	6.55	-1.59	0.0011	0.03	WDFY4	WDFY family member 4	Multiple_Complex
TC0400012018.hg.1	6.54	8.32	-3.44	0.0011	0.03	GYPB	glycophorin B (MNS blood group)	Multiple_Complex
TC0800009231.hg.1	9.83	11.29	-2.75	0.0011	0.03	GRINA	glutamate receptor, ionotropic, N-methyl D-aspartat	Multiple_Complex
TC0900011610.hg.1	4.69	5.58	-1.86	0.0011	0.03	ST6GALNAC4	ST6 (alpha-N-acetyl-neuraminyl-2,3-beta-galactosyl-1	Multiple_Complex
TC1000012495.hg.1	7.57	7.15	1.33	0.0011	0.0301	BORCS7	BLOC-1 related complex subunit 7	Multiple_Complex

TC1900009346.hg.1	8.66	10.09	-2.7	0.0011	0.0301	UBXN6	UBX domain protein 6	Multiple_Complex
TSUnmapped0000051	6.11	6.88	-1.71	0.0011	0.0301	DGKD	diacylglycerol kinase, delta 130kDa	NonCoding
TSUnmapped0000022	4.72	5.2	-1.4	0.0011	0.0301	RPS6KA1	ribosomal protein S6 kinase, 90kDa, polypeptide 1	NonCoding
TC1500007874.hg.1	9.99	10.88	-1.85	0.0011	0.0301	C15orf39	chromosome 15 open reading frame 39	Coding
TC1000007333.hg.1	9.65	10.47	-1.76	0.0011	0.0302	ZNF33A	zinc finger protein 33A	Multiple_Complex
TC1600009446.hg.1	11.92	12.97	-2.06	0.0011	0.0302	CPPED1	calcineurin-like phosphoesterase domain containing	Multiple_Complex
TC0100008271.hg.1	10.02	11.42	-2.63	0.0011	0.0303	RNF11	ring finger protein 11	Multiple_Complex
TC0600007769.hg.1	5.74	5.36	1.31	0.0011	0.0304	SNRPC	small nuclear ribonucleoprotein polypeptide C	Multiple_Complex
TSUnmapped0000072	3.16	3.66	-1.41	0.0011	0.0305	PRAMEF10	PRAME family member 10	Coding
TC0300007479.hg.1	4.4	4.95	-1.46	0.0011	0.0306	PHF7	PHD finger protein 7	Multiple_Complex
TC0900008463.hg.1	8.17	8.93	-1.7	0.0011	0.0306	HSDL2	hydroxysteroid dehydrogenase like 2	Multiple_Complex
TC0800012026.hg.1	7.02	7.99	-1.96	0.0011	0.0306	SLC45A4	solute carrier family 45, member 4	Multiple_Complex
TC1400007307.hg.1	8.83	9.56	-1.66	0.0011	0.0306	ARID4A	AT rich interactive domain 4A (RBP1-like)	Multiple_Complex
TC1900011004.hg.1	7.23	8.25	-2.02	0.0011	0.0306	SLC1A5	solute carrier family 1 (neutral amino acid transporte	Multiple_Complex
TC1200008685.hg.1	8.18	8.95	-1.71	0.0011	0.0306	CHST11	Transcript Identified by AceView, Entrez Gene ID(s) 5	Unassigned
TC1000008005.hg.1	7.5	8.13	-1.55	0.0011	0.0306	MCU	mitochondrial calcium uniporter	Multiple_Complex
TC0100014008.hg.1	7.13	6.78	1.27	0.0011	0.0306	MUTYH	mutY DNA glycosylase	Multiple_Complex
TC1300008084.hg.1	8.16	9.14	-1.96	0.0011	0.0306	ATP11A	ATPase, class VI, type 11A	Multiple_Complex
TC1100012917.hg.1	6.58	6.22	1.29	0.0012	0.0306	NCAPD3	non-SMC condensin II complex subunit D3	Multiple_Complex
TC0200015601.hg.1	7	7.73	-1.65	0.0012	0.0306	KANSL1L	KAT8 regulatory NSL complex subunit 1 like	Multiple_Complex
TC1300009905.hg.1	6.99	6.43	1.47	0.0012	0.0306	LOC101928841; R	collagen alpha-1(II) chain-like; novel transcript	Multiple_Complex
TC1400009239.hg.1	8.79	7.93	1.81	0.0012	0.0307	GCH1	GTP cyclohydrolase 1	Multiple_Complex
TC0800012475.hg.1	9.05	8.47	1.49	0.0012	0.0307	JRK	Jrk helix-turn-helix protein	Multiple_Complex
TC1900006470.hg.1	13.92	15.74	-3.53	0.0012	0.0307	BSG	basigin (Ok blood group)	Multiple_Complex
TC1700012191.hg.1	14.13	13.25	1.84	0.0012	0.0307	CD68	CD68 molecule	Coding
TC1200012334.hg.1	17.43	16.87	1.47	0.0012	0.0307	UBC	ubiquitin C	Multiple_Complex
TC0500012207.hg.1	5.44	4.91	1.44	0.0012	0.0308	SPATA24	spermatogenesis associated 24	Multiple_Complex
TC0X00007523.hg.1	4.68	4.33	1.27	0.0012	0.0308	EDA	ectodysplasin A	Multiple_Complex
TC0600011732.hg.1	6.68	6.25	1.34	0.0012	0.0308	SAYSD1	SAYSVFN motif domain containing 1	Multiple_Complex
TC1500008978.hg.1	9.51	9.12	1.31	0.0012	0.0308	EMC7	ER membrane protein complex subunit 7	Multiple_Complex
TC0400012916.hg.1	7.24	6.7	1.45	0.0012	0.031	ARAP2	ArfGAP with RhoGAP domain, ankyrin repeat and PH	NonCoding
TC0300012281.hg.1	10.22	11.09	-1.83	0.0012	0.031	ZXDC	ZXD family zinc finger C	Multiple_Complex
TC0400008106.hg.1	10.64	8.3	5.07	0.0012	0.031	HERC5	HECT and RLD domain containing E3 ubiquitin protei	Multiple_Complex
TC2200009271.hg.1	14.46	13.73	1.66	0.0012	0.0313	APOBEC3C	apolipoprotein B mRNA editing enzyme, catalytic pol	Multiple_Complex
TC0300010209.hg.1	9.71	9.15	1.48	0.0012	0.0313	TADA3	transcriptional adaptor 3	Multiple_Complex
TC0200008663.hg.1	9.51	11.54	-4.06	0.0012	0.0313	IL1R2	interleukin 1 receptor, type II	Multiple_Complex
TC1400010261.hg.1	3.53	4.06	-1.44	0.0012	0.0314	MOK	MOK protein kinase	Multiple_Complex
TC0300011324.hg.1	5.17	5.9	-1.66	0.0012	0.0319	FHIT	fragile histidine triad	Multiple_Complex
TC1200008568.hg.1	10.47	9.96	1.42	0.0012	0.0319	TMPO	thymopoietin	Multiple_Complex
TC0600013272.hg.1	9.62	10.46	-1.79	0.0012	0.0319	BCLAF1	BCL2-associated transcription factor 1	Multiple_Complex
TC1900007836.hg.1	5.07	5.58	-1.43	0.0012	0.032	FXYD3; MIR6887	FXYD domain containing ion transport regulator 3; m	Multiple_Complex
TC0100015921.hg.1	10.68	9.63	2.07	0.0012	0.032	ADAR	adenosine deaminase, RNA-specific	Multiple_Complex
TC0100016051.hg.1	5.92	6.56	-1.56	0.0012	0.032	FCRL2	Fc receptor-like 2	Multiple_Complex
TC1200010839.hg.1	10.58	11.51	-1.91	0.0012	0.0322	ITGA5	integrin alpha 5	Multiple_Complex
TC0800012202.hg.1	8.05	7.51	1.46	0.0012	0.0322	BOP1; MIR7112	block of proliferation 1; microRNA 7112	Multiple_Complex
TC1100010091.hg.1	6.37	6.83	-1.37	0.0013	0.0323	MRVI1	murine retrovirus integration site 1 homolog	Multiple_Complex
TC1700011313.hg.1	9.23	10.04	-1.76	0.0013	0.0324	USP32	ubiquitin specific peptidase 32	Multiple_Complex
TC1900010789.hg.1	7.63	8.38	-1.68	0.0013	0.0324	POU2F2	POU class 2 homeobox 2	Multiple_Complex
TC0X00011318.hg.1	5.99	5.57	1.34	0.0013	0.0328	TMSB15B	thymosin beta 15B	NonCoding
TC0700010564.hg.1	5.23	4.52	1.63	0.0013	0.0328	HOXA10; HOXA9;	homeobox A10; homeobox A9; microRNA 196b	Multiple_Complex
TC0800012212.hg.1	8.39	8.97	-1.49	0.0013	0.0328	SLC39A4	solute carrier family 39 (zinc transporter), member 4	Multiple_Complex
TC0200012382.hg.1	6.65	7.25	-1.52	0.0013	0.0328	OXER1	oxoeicosanoid (OXE) receptor 1	Coding
TC0100009760.hg.1	13.13	13.97	-1.8	0.0013	0.0329	NUDT4P1; NUDT4	nudix hydrolase 4 pseudogene 1; nudix hydrolase 4 p	Multiple_Complex
TC0100009833.hg.1	13.13	13.97	-1.8	0.0013	0.0329	NUDT4; NUDT4P1	nudix hydrolase 4; nudix hydrolase 4 pseudogene 1	Multiple_Complex
TC0100009877.hg.1	6.79	6.2	1.5	0.0013	0.0329	HIST2H3A	histone cluster 2, H3a	Coding
TC0700009411.hg.1	9.01	10.82	-3.52	0.0013	0.0329	MGAM2	maltase-glucoamylase 2 (putative)	Multiple_Complex
TC1700006709.hg.1	9.77	7.75	4.05	0.0013	0.033	XAF1	XIAP associated factor 1	Multiple_Complex
TC1900010008.hg.1	7.48	8.07	-1.51	0.0013	0.033	KIAA1683	KIAA1683	Multiple_Complex
TC0200012257.hg.1	13.35	11.46	3.71	0.0013	0.033	EIF2AK2	eukaryotic translation initiation factor 2-alpha kinase	Multiple_Complex
TC1100011339.hg.1	6.06	5.67	1.31	0.0013	0.033	CABP2	calcium binding protein 2	Coding
TC0500008921.hg.1	11.68	11.05	1.54	0.0013	0.0331	NDFIP1	Nedd4 family interacting protein 1	Multiple_Complex
TC1700011731.hg.1	8.28	7.72	1.47	0.0013	0.0331	ATP5H	ATP synthase, H+ transporting, mitochondrial Fo com	Multiple_Complex
TC2000007686.hg.1	11.42	12.7	-2.43	0.0013	0.0331	LINC01272	long intergenic non-protein coding RNA 1272	Multiple_Complex
TC0800012087.hg.1	5.88	6.52	-1.56	0.0013	0.0331	LYNX1	Ly6/neurotoxin 1	Multiple_Complex
TC0600007265.hg.1	4.5	3.87	1.54	0.0013	0.0331	HIST1H3C	histone cluster 1, H3c	Coding
TC0800010894.hg.1	4.21	4.78	-1.48	0.0013	0.0331	HEY1	hes-related family bHLH transcription factor with YRP	Multiple_Complex
TC0100006806.hg.1	10.53	11.41	-1.84	0.0013	0.0332	UBE4B	ubiquitination factor E4B	Multiple_Complex
TC0700013428.hg.1	11.63	12.63	-2.01	0.0013	0.0332	PILRB; STAG3L5P;	paired immunoglobin-like type 2 receptor beta; stror	Multiple_Complex
TC0300012728.hg.1	13.03	10.98	4.14	0.0013	0.0335	PLSCR1	phospholipid scramblase 1	Multiple_Complex
TC0700012996.hg.1	7.84	8.49	-1.57	0.0014	0.0339	ZNF746	zinc finger protein 746	Multiple_Complex
TC1600007130.hg.1	3.54	3.85	-1.24	0.0014	0.0339	ACSM5	acyl-CoA synthetase medium-chain family member 5	Multiple_Complex
TC1900008906.hg.1	6.06	5.77	1.22	0.0014	0.034	TMEM190	transmembrane protein 190	Coding

TC0600012777.hg.1	10.08	10.76	-1.61	0.0014	0.0343	SNX3	sorting nexin 3	Multiple_Complex
TC0100008897.hg.1	4.5	4	1.41	0.0014	0.0343	WDR63	WD repeat domain 63	Multiple_Complex
TC0300012897.hg.1	11.5	11.02	1.4	0.0014	0.0343	SSR3	signal sequence receptor, gamma (translocon-associa	Multiple_Complex
TC1500007072.hg.1	11.83	11.4	1.35	0.0014	0.0343	PDIA3	protein disulfide isomerase family A member 3	Multiple_Complex
TC1100011113.hg.1	3.87	3.46	1.33	0.0014	0.0344	SLC22A8	solute carrier family 22 (organic anion transporter), r	Multiple_Complex
TC1500008983.hg.1	12.53	13.13	-1.51	0.0014	0.0345	SLC12A6	solute carrier family 12 (potassium/chloride transpor	Multiple_Complex
TC1200010023.hg.1	9.71	10.36	-1.56	0.0014	0.0345	ERP27	endoplasmic reticulum protein 27	Multiple_Complex
TC1700007536.hg.1	4.76	4.39	1.29	0.0014	0.0345	SPACA3	sperm acrosome associated 3	Multiple_Complex
TSUnmapped000004:	5.51	5.21	1.23	0.0014	0.0345	HMBS	hydroxymethylbilane synthase	Coding
TC0800012180.hg.1	6.05	5.49	1.47	0.0014	0.0346	PARP10	poly(ADP-ribose) polymerase family member 10	Multiple_Complex
TC0600011134.hg.1	8.55	7.75	1.73	0.0014	0.0347	HIST1H4D	histone cluster 1, H4d	Coding
TC0900008842.hg.1	4.27	4.79	-1.43	0.0014	0.0347	SLC25A25	Zhang2013 ALT_ACCEPTOR, ALT_DONOR, coding, INT	NonCoding
TC0X00010155.hg.1	9.32	8.55	1.7	0.0014	0.0347	ITM2A	integral membrane protein 2A	Multiple_Complex
TC0700007352.hg.1	7.1	6.43	1.59	0.0014	0.0347	BLVRA	biliverdin reductase A	Multiple_Complex
TC1700007779.hg.1	11.02	12.22	-2.3	0.0014	0.0348	MSL1	male-specific lethal 1 homolog (Drosophila)	Multiple_Complex
TC0100010230.hg.1	6.83	7.36	-1.45	0.0014	0.0348	CD1C	CD1c molecule	Multiple_Complex
TC0200014738.hg.1	8.76	9.53	-1.71	0.0014	0.0348	RBMS1	Memczak2013 ALT_ACCEPTOR, ALT_DONOR, coding,	NonCoding
TC1600010656.hg.1	4.37	3.93	1.35	0.0014	0.035	CENPT	centromere protein T	Multiple_Complex
TC1100007006.hg.1	5.5	5.9	-1.32	0.0014	0.0351	LDHAL6A	lactate dehydrogenase A-like 6A	Coding
TC1500010658.hg.1	7.07	6.42	1.57	0.0014	0.0351	VIMP	VCP-interacting membrane selenoprotein	Multiple_Complex
TC1200012080.hg.1	7.66	9.04	-2.61	0.0014	0.0351	TESC	tescalcin	Multiple_Complex
TC0600013409.hg.1	8.21	8.99	-1.71	0.0015	0.0355	HIVEP2	human immunodeficiency virus type I enhancer bind	Multiple_Complex
TC1400006552.hg.1	4.75	5.05	-1.24	0.0015	0.0355	RP11-998D10.7; F	Transcript Identified by AceView, Entrez Gene ID(s) 5	NonCoding
TC1100010175.hg.1	7.96	7.41	1.47	0.0015	0.0355	RRAS2	related RAS viral (r-ras) oncogene homolog 2	Multiple_Complex
TC0500008782.hg.1	10.59	11.36	-1.7	0.0015	0.0356	KDM3B	lysine (K)-specific demethylase 3B	Multiple_Complex
TC0300013763.hg.1	9.79	9.21	1.49	0.0015	0.0357	RUBCN; MIR922	RUN domain and cysteine-rich domain containing, Be	Multiple_Complex
TC1200012723.hg.1	5.73	6.17	-1.35	0.0015	0.0357	ZNF664	zinc finger protein 664	Multiple_Complex
TC1300008342.hg.1	8.73	8.07	1.59	0.0015	0.0357	C1QTNF9B	C1q and tumor necrosis factor related protein 9B	Multiple_Complex
TC1000008891.hg.1	7.18	6.5	1.6	0.0015	0.0357	DUSP5	dual specificity phosphatase 5	Multiple_Complex
TC0300012029.hg.1	12.53	12.18	1.27	0.0015	0.0358	NAA50	N(alpha)-acetyltransferase 50, NatE catalytic subunit	Multiple_Complex
TC0400009322.hg.1	8.51	9.21	-1.62	0.0015	0.036	GALNT7	polypeptide N-acetylgalactosaminyltransferase 7	Multiple_Complex
TC1100013003.hg.1	10.03	9.39	1.56	0.0015	0.0361	TMX2; C11orf31	thioredoxin-related transmembrane protein 2; chron	Multiple_Complex
TC0300011175.hg.1	8.55	8.25	1.24	0.0015	0.0362	PBRM1	polybromo 1	Multiple_Complex
TC0900012202.hg.1	10.01	10.42	-1.32	0.0015	0.0363	KIAA2026	KIAA2026	Multiple_Complex
TSUnmapped000007(	7.28	6.85	1.35	0.0015	0.0364	HYOU1	hypoxia up-regulated 1	NonCoding
TC0700011194.hg.1	3.36	3.74	-1.3	0.0015	0.0365	LOC401357; RP13	uncharacterized LOC401357; novel piRNA host transc	NonCoding
TC1700012322.hg.1	4.09	3.58	1.43	0.0015	0.0365	GLOD4	glyoxalase domain containing 4	Multiple_Complex
TC1900009959.hg.1	5.9	5.56	1.27	0.0015	0.0365	ABHD8	abhydrolase domain containing 8	Multiple_Complex
TC0200015815.hg.1	6.23	7.18	-1.93	0.0015	0.0365	EPHA4	EPH receptor A4	Multiple_Complex
TC0100014562.hg.1	7.4	6.96	1.36	0.0015	0.0365	LRRC40	leucine rich repeat containing 40	Multiple_Complex
TC0800012086.hg.1	4.9	5.49	-1.5	0.0015	0.0367	LYPD2	LY6/PLAUR domain containing 2	Coding
TC0300013636.hg.1	6.88	7.66	-1.71	0.0015	0.0367	ACAP2; ACAP2-IT	Transcript Identified by AceView, Entrez Gene ID(s) 2	NonCoding
TC2200009107.hg.1	3.47	3.85	-1.3	0.0015	0.0367	C22orf34	chromosome 22 open reading frame 34	Multiple_Complex
TC1500010422.hg.1	9.3	8.89	1.33	0.0016	0.037	IDH2	isocitrate dehydrogenase 2 (NADP+), mitochondrial	Multiple_Complex
TC0600011136.hg.1	6.39	5.48	1.87	0.0016	0.037	HIST1H2BG	histone cluster 1, H2bg	Multiple_Complex
TC1900009651.hg.1	5.28	5.86	-1.49	0.0016	0.0371	YIPF2	Yip1 domain family member 2	Multiple_Complex
TC0600007869.hg.1	7.6	7.05	1.47	0.0016	0.0371	CMTR1	cap methyltransferase 1	Multiple_Complex
TC1700007790.hg.1	5.4	4.88	1.44	0.0016	0.0371	CDC6	cell division cycle 6	Multiple_Complex
TC0600009059.hg.1	11.11	12.08	-1.97	0.0016	0.0372	FOXO3	forkhead box O3	Multiple_Complex
TC1600011358.hg.1	4.89	4.34	1.47	0.0016	0.0372	C16orf45	chromosome 16 open reading frame 45	Multiple_Complex
TC0500008691.hg.1	11.63	12.06	-1.35	0.0016	0.0373	UBE2B	ubiquitin conjugating enzyme E2B	Multiple_Complex
TC1000009172.hg.1	11	10.39	1.53	0.0016	0.0373	BUB3	BUB3 mitotic checkpoint protein	Multiple_Complex
TC0X00009128.hg.1	3.57	3.99	-1.34	0.0016	0.0374	ACE2	angiotensin I converting enzyme 2	Multiple_Complex
TC0800012277.hg.1	4.14	4.56	-1.34	0.0016	0.0375	SFTPC	surfactant protein C	Multiple_Complex
TC0200013600.hg.1	6.62	6.02	1.52	0.0016	0.0375	KIAA1211L	KIAA1211-like	Multiple_Complex
TC1300008080.hg.1	6.8	6.32	1.4	0.0016	0.0375	TUBGCP3	Memczak2013 ANTISENSE, coding, INTERNAL, introni	NonCoding
TC1100010268.hg.1	9.38	9.91	-1.45	0.0016	0.0375	TSG101	tumor susceptibility 101	Multiple_Complex
TC0100010017.hg.1	5.79	6.16	-1.3	0.0016	0.0375	SPRR1A	small proline-rich protein 1A	Multiple_Complex
TC0X00008731.hg.1	8.72	8.28	1.35	0.0016	0.0375	VMA21	VMA21 vacuolar H+-ATPase homolog (S. cerevisiae)	Multiple_Complex
TC0500007666.hg.1	6.41	5.68	1.67	0.0016	0.0379	CENPH	centromere protein H	Multiple_Complex
TC1000009404.hg.1	6.56	6.19	1.29	0.0016	0.0382	PWWP2B	PWWP domain containing 2B	Coding
TC0300009728.hg.1	5.09	5.87	-1.72	0.0016	0.0382	VPS8	Transcript Identified by AceView, Entrez Gene ID(s) 2	Unassigned
TC1500008245.hg.1	13.75	14.44	-1.61	0.0016	0.0382	ABHD2	abhydrolase domain containing 2	Multiple_Complex
TC0X00011214.hg.1	6.21	5.81	1.32	0.0016	0.0383	CTAG1B	cancer/testis antigen 1B	Multiple_Complex
TC0200015211.hg.1	9.67	10.6	-1.9	0.0016	0.0383	SLC40A1	solute carrier family 40 (iron-regulated transporter),	Multiple_Complex
TC1900008358.hg.1	6.67	6.14	1.44	0.0017	0.0383	CCDC61	coiled-coil domain containing 61	Multiple_Complex
TC1500007238.hg.1	7.41	8.06	-1.57	0.0017	0.0383	TMOD2	tropomodulin 2 (neuronal)	Multiple_Complex
TC0900010886.hg.1	9.08	8.49	1.51	0.0017	0.0383	PTCH1	patched 1	Multiple_Complex
TC0800011064.hg.1	5.77	5.14	1.55	0.0017	0.0383	CALB1	calbindin 1	Multiple_Complex
TC1900011841.hg.1	5.75	5.13	1.53	0.0017	0.0383	LMNB2; MIR7108	lamin B2; microRNA 7108	Multiple_Complex
TC1500010778.hg.1	10.79	11.55	-1.69	0.0017	0.0383	IL16	interleukin 16	Multiple_Complex
TC0X00009549.hg.1	7.95	7.42	1.45	0.0017	0.0383	NDUFB11	NADH dehydrogenase (ubiquinone) 1 beta subcompl	Multiple_Complex

TC1100006812.hg.1	9.7	10.18	-1.39	0.0017	0.0384	ZNF143	zinc finger protein 143	Multiple_Complex
TC0100011242.hg.1	8	8.57	-1.49	0.0017	0.0384	TMEM183A	transmembrane protein 183A	Multiple_Complex
TC0300009632.hg.1	4.85	4.51	1.27	0.0017	0.0387	SOX2	SRY box 2	Coding
TC0600007287.hg.1	5.39	4.81	1.5	0.0017	0.0387	HIST1H3E	histone cluster 1, H3e	Coding
TC0600012542.hg.1	12.72	12.06	1.57	0.0017	0.0387	UBE2J1	ubiquitin-conjugating enzyme E2, J1	Multiple_Complex
TC1200012700.hg.1	9.28	8.39	1.85	0.0017	0.0387	C12orf75	chromosome 12 open reading frame 75	Multiple_Complex
TC1200010201.hg.1	8.23	7.72	1.43	0.0017	0.0388	TM7SF3	transmembrane 7 superfamily member 3	Multiple_Complex
TC0800012351.hg.1	7.9	7.39	1.42	0.0017	0.0389	WDYHV1	WDYHV motif containing 1	Multiple_Complex
TC0100012889.hg.1	6.99	6.62	1.29	0.0017	0.0389	MAD2L2	MAD2 mitotic arrest deficient-like 2 (yeast)	Multiple_Complex
TC0400006987.hg.1	12.72	11.07	3.14	0.0017	0.0394	LAP3	leucine aminopeptidase 3	Multiple_Complex
TC1200007833.hg.1	6.45	6.18	1.2	0.0017	0.0394	SLC39A5	solute carrier family 39 (zinc transporter), member 5	Multiple_Complex
TC0900009944.hg.1	7.7	8.35	-1.57	0.0017	0.0394	CD72	CD72 molecule	Multiple_Complex
TC0200016661.hg.1	3.82	4.29	-1.39	0.0017	0.0394	C2orf61	chromosome 2 open reading frame 61	Multiple_Complex
TC0500008307.hg.1	3.34	3.77	-1.35	0.0017	0.0394	TSLP	thymic stromal lymphopoietin	Multiple_Complex
TC1800008824.hg.1	11.18	10.7	1.4	0.0017	0.0394	LMAN1	lectin, mannose-binding, 1	Multiple_Complex
TC0X00007540.hg.1	5.74	5.26	1.4	0.0017	0.0395	DLG3	discs, large homolog 3 (Drosophila)	Multiple_Complex
TC0700008659.hg.1	6.06	6.62	-1.48	0.0017	0.0395	NFE4	nuclear factor, erythroid 4	Multiple_Complex
TC1600010500.hg.1	7.11	6.56	1.47	0.0017	0.0395	GOT2	glutamic-oxaloacetic transaminase 2, mitochondrial	Multiple_Complex
TC1600006488.hg.1	7.2	6.76	1.35	0.0018	0.0396	STUB1	STIP1 homology and U-box containing protein 1, E3 u	Multiple_Complex
TC1100009191.hg.1	12.7	12.13	1.48	0.0018	0.0397	IL10RA	interleukin 10 receptor, alpha	Multiple_Complex
TC1600008568.hg.1	9.69	9.36	1.26	0.0018	0.0397	ATMIN	ATM interactor	Multiple_Complex
TC1700010738.hg.1	9.45	9.95	-1.42	0.0018	0.0397	EZH1	enhancer of zeste 1 polycomb repressive complex 2 s	Multiple_Complex
TC0100008080.hg.1	8.05	7.71	1.27	0.0018	0.0397	DPH2	DPH2 homolog	Multiple_Complex
TC1100009685.hg.1	5.63	4.89	1.67	0.0018	0.0398	IRF7	interferon regulatory factor 7	Multiple_Complex
TC0300007387.hg.1	8.53	9.45	-1.9	0.0018	0.0398	RNF123	ring finger protein 123	Multiple_Complex
TC1500010050.hg.1	9.47	8.88	1.51	0.0018	0.0399	COX5A	cytochrome c oxidase subunit Va	Multiple_Complex
TC0700010947.hg.1	7.68	7.05	1.54	0.0018	0.04	TBRG4; SNORA5E	transforming growth factor beta regulator 4; small nu	Multiple_Complex
TC1200010450.hg.1	8.7	8.32	1.3	0.0018	0.04	YAF2	YY1 associated factor 2	Multiple_Complex
TC0100017716.hg.1	3.64	3.27	1.29	0.0018	0.04	FAM89A; MIR118	family with sequence similarity 89, member A; micro	Multiple_Complex
TC1600011399.hg.1	12.35	10.62	3.33	0.0018	0.04	MT1X	metallothionein 1X	Multiple_Complex
TC1200008255.hg.1	6.51	7.17	-1.59	0.0018	0.04	ZDHHC17	zinc finger, DHHC-type containing 17	Multiple_Complex
TC0400006538.hg.1	7.56	8.16	-1.52	0.0018	0.0403	TACC3	transforming, acidic coiled-coil containing protein 3	Multiple_Complex
TC1100007826.hg.1	6.68	7.32	-1.56	0.0018	0.0403	BEST1	bestrophin 1	Multiple_Complex
TC0800007949.hg.1	10.06	9.54	1.43	0.0018	0.0403	TERF1	telomeric repeat binding factor (NIMA-interacting) 1	Multiple_Complex
TC1400007595.hg.1	7.31	8.3	-1.98	0.0018	0.0403	SIPA1L1	signal-induced proliferation-associated 1 like 1	Multiple_Complex
TC1900011542.hg.1	6.16	5.75	1.33	0.0018	0.0405	PEG3; ZIM2	paternally expressed 3; zinc finger, imprinted 2	Multiple_Complex
TC0200012339.hg.1	7.32	8.19	-1.83	0.0018	0.0405	SLC8A1	solute carrier family 8 (sodium/calcium exchanger), r	Multiple_Complex
TSUnmapped0000007	3.99	4.61	-1.54	0.0018	0.0405	HMBS	hydroxymethylbilane synthase	Coding
TC1800009228.hg.1	6.13	5.72	1.33	0.0018	0.0405	ELAC1	elaC ribonuclease Z 1	Multiple_Complex
TC0900008091.hg.1	7.66	8.23	-1.49	0.0018	0.0405	ERCC6L2	excision repair cross-complementation group 6-like 2	Multiple_Complex
TC1400008415.hg.1	6.02	6.63	-1.52	0.0018	0.0405	ZFYVE21	zinc finger, FYVE domain containing 21	Multiple_Complex
TC0700009499.hg.1	4.48	5.75	-2.41	0.0018	0.0405	TAS2R60	taste receptor, type 2, member 60	Coding
TC1100009449.hg.1	6.7	7.07	-1.29	0.0018	0.0405	TIRAP	toll-interleukin 1 receptor (TIR) domain containing ad	Multiple_Complex
TC0100009064.hg.1	4.59	5.29	-1.63	0.0018	0.0406	FNBP1L	formin binding protein 1-like	Multiple_Complex
TC1700010221.hg.1	6.98	8.09	-2.15	0.0018	0.0406	DHRS13	dehydrogenase/reductase (SDR family) member 13	Multiple_Complex
TC0600007792.hg.1	7.76	8.34	-1.49	0.0019	0.0407	PPARD	peroxisome proliferator-activated receptor delta	Multiple_Complex
TC0500012459.hg.1	5.5	5.01	1.4	0.0019	0.0411	PDGFRB	platelet-derived growth factor receptor, beta polype	Multiple_Complex
TC0600009228.hg.1	8.16	8.79	-1.55	0.0019	0.0411	NT5DC1	5-nucleotidase domain containing 1	Multiple_Complex
TC0700011575.hg.1	8.64	9.33	-1.61	0.0019	0.0411	POMZP3	POM121 and ZP3 fusion	Multiple_Complex
TC0400007120.hg.1	9.21	10.22	-2.03	0.0019	0.0411	RBPJ	recombination signal binding protein for immunoglol	Multiple_Complex
TC0500013222.hg.1	5.86	5.4	1.37	0.0019	0.0414	LOC100505841	zinc finger protein 474-like	Multiple_Complex
TC1900011770.hg.1	14.11	15.1	-1.98	0.0019	0.0414	C5AR1	complement component 5a receptor 1	Multiple_Complex
TC1000009997.hg.1	4.36	4.03	1.26	0.0019	0.0414	CASC10	cancer susceptibility candidate 10	Multiple_Complex
TC1800006715.hg.1	7.79	8.48	-1.62	0.0019	0.0415	IMPA2	inositol(myo)-1(or 4)-monophosphatase 2	Multiple_Complex
TC0100008631.hg.1	4.69	5.57	-1.84	0.0019	0.0415	SGIP1	SH3-domain GRB2-like (endophilin) interacting prote	Multiple_Complex
TC1500008545.hg.1	8.38	9.01	-1.55	0.0019	0.0416	ASB7	ankyrin repeat and SOCS box containing 7	Multiple_Complex
TC0200013616.hg.1	8.23	9.25	-2.02	0.0019	0.0416	AFF3	AF4/FMR2 family, member 3	Multiple_Complex
TC0800010138.hg.1	11.51	12.48	-1.95	0.0019	0.0416	RAB11FIP1	RAB11 family interacting protein 1 (class I)	Multiple_Complex
TC1400009697.hg.1	10.36	9.63	1.66	0.0019	0.0416	NPC2; MIR4709	Niemann-Pick disease, type C2; microRNA 4709	Multiple_Complex
TC1300007780.hg.1	8.51	8.09	1.34	0.0019	0.0416	RAP2A	RAP2A, member of RAS oncogene family	Coding
TC1900007809.hg.1	9.81	9.32	1.41	0.0019	0.0417	UBA2	ubiquitin-like modifier activating enzyme 2	Multiple_Complex
TC0600012289.hg.1	3.81	4.15	-1.26	0.0019	0.0417	OOEP	oocyte expressed protein	Coding
TC0500008830.hg.1	12.55	12.12	1.34	0.0019	0.0418	UBE2D2	ubiquitin conjugating enzyme E2D 2	Multiple_Complex
TC2100008098.hg.1	9.05	8.68	1.29	0.0019	0.042	DSCR3	Down syndrome critical region 3	Multiple_Complex
TC1700011922.hg.1	8.21	7.75	1.38	0.002	0.0421	LGALS3BP	lectin, galactoside-binding, soluble, 3 binding protein	Multiple_Complex
TC1900009550.hg.1	5	4.75	1.19	0.002	0.0422	ADAMTS10	ADAM metallopeptidase with thrombospondin type	Multiple_Complex
TC1100010762.hg.1	8.18	7.65	1.45	0.002	0.0422	NUP160	nucleoporin 160kDa	Multiple_Complex
TC0200007739.hg.1	5.4	4.79	1.53	0.002	0.0425	COMMD1	copper metabolism (Murr1) domain containing 1	Multiple_Complex
TC1100011520.hg.1	6.84	7.24	-1.32	0.002	0.0425	KRTAP5-11	keratin associated protein 5-11	Multiple_Complex
TC1500008052.hg.1	8.9	8.5	1.32	0.002	0.0425	MESDC1	mesoderm development candidate 1	Coding
TC2000009267.hg.1	9.79	10.25	-1.38	0.002	0.0425	SLC35C2	solute carrier family 35 (GDP-fucose transporter), me	Multiple_Complex
TC0200009189.hg.1	13.77	15.18	-2.66	0.002	0.0425	GYPC	glycophorin C (Gerbich blood group)	Multiple_Complex

TC1200012636.hg.1	17.58	18.5	-1.89	0.002	0.0425	PCBP2; PCBP2-OT	poly(rC) binding protein 2; PCBP2 overlapping transc	Multiple_Complex	
TC0400012828.hg.1	8.3	9.2	-1.86	0.002	0.0425	BANK1	B-cell scaffold protein with ankyrin repeats 1	Multiple_Complex	
TC2200009273.hg.1	9.81	9.21	1.52	0.002	0.0426	APOBEC3F	apolipoprotein B mRNA editing enzyme, catalytic pol	Multiple_Complex	
TC0100008536.hg.1	6.71	7.55	-1.79	0.002	0.0426	INADL	InaD-like (Drosophila)	Multiple_Complex	
TC1100006547.hg.1	4.5	4.2	1.23	0.002	0.0426	MRPL23	mitochondrial ribosomal protein L23	Multiple_Complex	
TC1200008134.hg.1	6.71	6.12	1.51	0.002	0.0426	YEATS4	YEATS domain containing 4	Multiple_Complex	
TC1200009911.hg.1	8.9	8.62	1.21	0.002	0.0426	MAGOHB	mago homolog B, exon junction complex core compo	Multiple_Complex	
TC1700012377.hg.1	3.52	3.98	-1.37	0.002	0.0428	LYRM9	LYR motif containing 9	Multiple_Complex	
TC1900009102.hg.1	6.03	5.42	1.53	0.002	0.0428	RNF126	ring finger protein 126	Multiple_Complex	
TC1700011892.hg.1	5.16	4.5	1.58	0.002	0.0428	TK1	thymidine kinase 1, soluble	Multiple_Complex	
TC0100010078.hg.1	11.38	12.4	-2.02	0.002	0.0429	IL6R	interleukin 6 receptor	Multiple_Complex	
TC2100008000.hg.1	8.56	7.98	1.5	0.002	0.0429	GART	phosphoribosylglycinamide formyltransferase, phosp	Multiple_Complex	
TC0900012292.hg.1	3.85	4.2	-1.27	0.002	0.043	LRRC26	leucine rich repeat containing 26	Coding	
TC1600010756.hg.1	5.73	6.31	-1.49	0.002	0.0431	ST3GAL2	ST3 beta-galactoside alpha-2,3-sialyltransferase 2	Multiple_Complex	
TC1200006450.hg.1	10.64	11.96	-2.5	0.002	0.0431	WNK1	WNK lysine deficient protein kinase 1	Multiple_Complex	
TC0100010379.hg.1	11.58	11.08	1.42	0.002	0.0431	UHMK1	U2AF homology motif (UHM) kinase 1	Multiple_Complex	
TC0500013351.hg.1	9.38	9.89	-1.42	0.0021	0.0431	FNIP1	folliculin interacting protein 1	Multiple_Complex	
TC1700007897.hg.1	6.61	5.93	1.6	0.0021	0.0431	HSD17B1	hydroxysteroid (17-beta) dehydrogenase 1	Multiple_Complex	
TSUnmapped000000	4.57	4.13	1.36	0.0021	0.0432	PSMC4	proteasome 26S subunit, ATPase 4 [Source:HGNC Syr	NonCoding	
TC1500010908.hg.1	9.32	8.55	1.71	0.0021	0.0437	STARD5	StAR-related lipid transfer domain containing 5	NonCoding	
TC0X00007053.hg.1	5.09	4.61	1.39	0.0021	0.0437	MAOA	monoamine oxidase A	Multiple_Complex	
TC0300009289.hg.1	3.76	4.28	-1.44	0.0021	0.0437	LEKR1	leucine, glutamate and lysine rich 1	Multiple_Complex	
TC0100010155.hg.1	12.84	14.1	-2.4	0.0021	0.0437	SEMA4A	sema domain, immunoglobulin domain (Ig), transme	Multiple_Complex	
TC1000009152.hg.1	4.31	3.6	1.64	0.0021	0.0437	HTRA1	HtrA serine peptidase 1	Multiple_Complex	
TC0700009996.hg.1	7.04	6.63	1.33	0.0021	0.0437	C7orf50	chromosome 7 open reading frame 50	Multiple_Complex	
TC0800010990.hg.1	11.39	13.92	-5.77	0.0021	0.0438	CA1	carbonic anhydrase I	Multiple_Complex	
TC2000007620.hg.1	8.62	9.25	-1.54	0.0021	0.044	ARFGEF2	ADP-ribosylation factor guanine nucleotide-exchange	Multiple_Complex	
TC0100017078.hg.1	7.72	7.11	1.52	0.0021	0.044	RAB29	RAB29, member RAS oncogene family	Multiple_Complex	
TC1100011794.hg.1	9.82	10.46	-1.56	0.0021	0.0441	PRCP	prolylcarboxypeptidase	Multiple_Complex	
TC1100009225.hg.1	7.69	8.6	-1.87	0.0021	0.0441	CXCR5	chemokine (C-X-C motif) receptor 5	Coding	
TC1100012474.hg.1	7.91	8.47	-1.47	0.0021	0.0441	MPZL2	myelin protein zero-like 2	Multiple_Complex	
TC0300010358.hg.1	8.71	9.29	-1.49	0.0021	0.0442	XPC	xeroderma pigmentosum, complementation group C	Multiple_Complex	
TC0900009996.hg.1	7.24	7.75	-1.42	0.0021	0.0442	PAX5	paired box 5	Multiple_Complex	
TC1700011783.hg.1	7.79	7.28	1.43	0.0022	0.0446	SRP68	signal recognition particle 68kDa	Multiple_Complex	
TC1600008657.hg.1	10.34	9.71	1.55	0.0022	0.0448	USP10	ubiquitin specific peptidase 10	Multiple_Complex	
TC1100006896.hg.1	5.16	5.54	-1.3	0.0022	0.0448	RASSF10	Ras association (RalGDS/AF-6) domain family (N-term	Coding	
TC0100008517.hg.1	6.61	7.53	-1.89	0.0022	0.0452	NFIA	nuclear factor I/A	Multiple_Complex	
TC0600010066.hg.1	10.63	11.94	-2.48	0.0022	0.0454	IGF2R	insulin-like growth factor 2 receptor	Multiple_Complex	
TC0X00009384.hg.1	5.58	5.01	1.49	0.0022	0.0454	DYNLT3	dynein, light chain, Tctex-type 3	Coding	
TC1700011033.hg.1	13.09	14.09	-1.99	0.0022	0.0455	PHOSPHO1	phosphatase, orphan 1	Coding	
TC2200007101.hg.1	9.71	9.24	1.39	0.0022	0.0457	LINC01521	long intergenic non-protein coding RNA 1521	NonCoding	
TC1300006799.hg.1	7.67	8.42	-1.68	0.0022	0.0457	FRY	FRY microtubule binding protein	Multiple_Complex	
TC1900008967.hg.1	6.56	6.22	1.27	0.0022	0.0458	ZNF470	zinc finger protein 470	Multiple_Complex	
TC1900009970.hg.1	6.39	5.51	1.84	0.0022	0.0458	BST2	bone marrow stromal cell antigen 2	Multiple_Complex	
TC1600009855.hg.1	13.21	13.8	-1.5	0.0022	0.0459	NPIPB6	nuclear pore complex interacting protein family, mer	Coding	
TC0100015764.hg.1	7.37	7.75	-1.3	0.0022	0.046	FAM63A	family with sequence similarity 63, member A	Multiple_Complex	
TC0300008863.hg.1	13.71	13.25	1.37	0.0022	0.046	CDV3	CDV3 homolog (mouse)	Multiple_Complex	
TC0800009735.hg.1	7.98	7.3	1.61	0.0023	0.046	MTUS1	microtubule associated tumor suppressor 1	Multiple_Complex	
TC0600007605.hg.1	12.78	12.16	1.53	0.0023	0.046	LY6G5B; CSNK2B	lymphocyte antigen 6 complex, locus G5B; casein kin	Multiple_Complex	
TC1200010931.hg.1	12.49	11.99	1.41	0.0023	0.046	PTGES3	prostaglandin E synthase 3 (cytosolic)	Multiple_Complex	
TC1100006659.hg.1	4.7	5.11	-1.32	0.0023	0.0461	OR52M1	olfactory receptor, family 52, subfamily M, member :	Coding	
TC1100012424.hg.1	9.12	10.08	-1.95	0.0023	0.0461	SIK3	SIK family kinase 3	Multiple_Complex	
TC0100013629.hg.1	7.36	6.97	1.3	0.0023	0.0461	YARS	tyrosyl-tRNA synthetase	Multiple_Complex	
TC0900010582.hg.1	5.84	6.68	-1.79	0.0023	0.0463	KIF27	kinesin family member 27	Multiple_Complex	
TC0900009339.hg.1	7.91	8.38	-1.38	0.0023	0.0464	WASH1	WAS protein family homolog 1	Multiple_Complex	
TC1600008190.hg.1	8.19	8.73	-1.45	0.0023	0.0465	SLC7A6	solute carrier family 7 (amino acid transporter light c	Multiple_Complex	
TC1900007619.hg.1	6.08	5.64	1.36	0.0023	0.0466	ZNF726; ZNF92P3	zinc finger protein 726; zinc finger protein 92 pseudo	Multiple_Complex	
TC1200012606.hg.1	11.2	11.83	-1.55	0.0023	0.0468	LRMP	lymphoid-restricted membrane protein	Multiple_Complex	
TC0600011441.hg.1	12.32	13.07	-1.68	0.0023	0.0469	BAG6	BCL2 associated athanogene 6	Multiple_Complex	
TC1000010849.hg.1	8.72	8.95	-1.17	0.0023	0.047	SLC25A16	solute carrier family 25 (mitochondrial carrier), mem	Multiple_Complex	
TC0900012076.hg.1	6.67	6.31	1.28	0.0023	0.047	ZMYND19	zinc finger, MYND-type containing 19	Multiple_Complex	
TC1600010914.hg.1	3.6	3.97	-1.29	0.0023	0.047	ADAMTS18	ADAM metallopeptidase with thrombospondin type	Multiple_Complex	
TC1200011327.hg.1	4.61	4.18	1.35	0.0023	0.047	CSRP2	cysteine and glycine-rich protein 2	Multiple_Complex	
TC0600014287.hg.1	3.05	3.42	-1.3	0.0023	0.0471	MOCS1	molybdenum cofactor synthesis 1	Multiple_Complex	
TC1700012015.hg.1	7.26	6.88	1.3	0.0023	0.0471	AATK	apoptosis-associated tyrosine kinase	Multiple_Complex	
TC0X00009160.hg.1	8.77	8.38	1.31	0.0023	0.0471	RBBP7	retinoblastoma binding protein 7	Multiple_Complex	
TC1900011445.hg.1	6.49	6.07	1.34	0.0024	0.0472	PPP1R12C	protein phosphatase 1, regulatory subunit 12C	Multiple_Complex	
TC0600008156.hg.1	8.78	7.89	1.85	0.0024	0.0473	ENPP4	ectonucleotide pyrophosphatase/phosphodiesterase	Coding	
TC0300007445.hg.1	3.92	4.37	-1.37	0.0024	0.0473	GRM2	glutamate receptor, metabotropic 2	Multiple_Complex	
TC0200016444.hg.1	3.6	3.92	-1.25	0.0024	0.0473	MSH6	mutS homolog 6	NonCoding	
TC1600006888.hg.1	10.34	11.06	-1.64	0.0024	0.0473	CIITA	class II, major histocompatibility complex, transactiva	Multiple_Complex	
TC1700009173.hg.1	6.36	5.91	1.36	0.0024	0.0474	BAHCC1	BAH domain and coiled-coil containing 1	Multiple_Complex	

TC2200009262.hg.1	10.5	11.27	-1.7	0.0024	0.0475	EIF3L	eukaryotic translation initiation factor 3, subunit L	Multiple_Complex
TC0400012780.hg.1	8.7	8.27	1.35	0.0024	0.0475	PI4K2B	phosphatidylinositol 4-kinase type 2 beta	Multiple_Complex
TC1900007098.hg.1	5.69	5.2	1.41	0.0024	0.0476	RNASEH2A	ribonuclease H2, subunit A	Multiple_Complex
TC0400011440.hg.1	11.93	12.57	-1.56	0.0024	0.0476	PPP3CA	protein phosphatase 3, catalytic subunit, alpha isozy	Multiple_Complex
TC0300014093.hg.1	9.36	9.9	-1.46	0.0024	0.0478	PCYT1A	phosphate cytidylyltransferase 1, choline, alpha	Multiple_Complex
TSUnmapped0000030	7.95	7.53	1.34	0.0024	0.0478	HYOU1	hypoxia up-regulated 1	Coding
TC1500006666.hg.1	7.78	7.47	1.24	0.0024	0.048	GOLGA8F; GOLGA	golgin A8 family, member F; golgin A8 family, membe	Multiple_Complex
TC0300012403.hg.1	7.68	8.72	-2.05	0.0024	0.0482	TMCC1	transmembrane and coiled-coil domain family 1	Multiple_Complex
TC1100011410.hg.1	6.19	5.81	1.3	0.0024	0.0483	MRGPRF	MAS-related GPR, member F	Coding
TC1200007764.hg.1	9.13	7.65	2.78	0.0024	0.0483	ZNF385A	Memczak2013 ANTISENSE, CDS, coding, INTERNAL be	NonCoding
TSUnmapped0000026	6.2	6.59	-1.31	0.0025	0.0485	MLXIP	MLX interacting protein	Coding
TC0600013427.hg.1	7.88	7.53	1.28	0.0025	0.0485	FUCA2	fucosidase, alpha-L- 2, plasma	Multiple_Complex
TC0300010399.hg.1	9.43	9.07	1.28	0.0025	0.0485	SH3BP5	SH3-domain binding protein 5 (BTK-associated)	Multiple_Complex
TC0400012881.hg.1	4.8	5.4	-1.51	0.0025	0.0485	DUX4	double homeobox 4	Multiple_Complex
TC0900007129.hg.1	4.93	4.29	1.55	0.0025	0.0488	MELK	maternal embryonic leucine zipper kinase	Multiple_Complex
TC0100015200.hg.1	5.36	5.72	-1.28	0.0025	0.0488	AMIGO1	adhesion molecule with Ig-like domain 1	Coding
TC1100010991.hg.1	3.63	4.07	-1.35	0.0025	0.0488	GIF	gastric intrinsic factor (vitamin B synthesis)	Multiple_Complex
TC0600011282.hg.1	8.74	9.29	-1.46	0.0025	0.0488	TRIM27	tripartite motif containing 27	Multiple_Complex
TC2200007138.hg.1	17.67	18.88	-2.31	0.0025	0.0488	FBXO7	F-box protein 7	Multiple_Complex
TC1600006566.hg.1	7.06	6.75	1.24	0.0025	0.0488	EME2	essential meiotic structure-specific endonuclease sub	Multiple_Complex
TC1400008940.hg.1	12.28	11.61	1.59	0.0025	0.0488	NFKBIA	nuclear factor of kappa light polypeptide gene enhar	Multiple_Complex
TC1500007174.hg.1	8.75	8.03	1.64	0.0025	0.0488	EID1	EP300 interacting inhibitor of differentiation 1	Multiple_Complex
TC0500011754.hg.1	12.12	11.72	1.32	0.0025	0.0491	TMED7-TICAM2;	TMED7-TICAM2 readthrough; toll-like receptor adapt	Multiple_Complex
TC0400008287.hg.1	7.97	8.88	-1.88	0.0025	0.0493	CISD2	CDGSH iron sulfur domain 2	Coding
TC0400010780.hg.1	4.82	5.37	-1.46	0.0025	0.0493	SPINK2	serine peptidase inhibitor, Kazal type 2 (acrosin-tryps	Multiple_Complex
TC1300007228.hg.1	8.49	8.92	-1.34	0.0025	0.0493	WDFY2	WD repeat and FYVE domain containing 2	Multiple_Complex
TC1600009412.hg.1	9.87	9.58	1.22	0.0025	0.0493	TXNDC11	thioredoxin domain containing 11	Multiple_Complex
TC1600011483.hg.1	16.12	17.11	-1.97	0.0025	0.0493	LITAF	lipopolysaccharide-induced TNF factor	Multiple_Complex
TC1900007950.hg.1	6.96	7.78	-1.77	0.0025	0.0493	ZNF567	zinc finger protein 567	Multiple_Complex
TC0100013432.hg.1	12.43	13.26	-1.77	0.0025	0.0495	WASF2	WAS protein family, member 2	Multiple_Complex
TC0500009996.hg.1	8.08	7.66	1.34	0.0025	0.0495	NSUN2	NOP2/Sun RNA methyltransferase family, member 2	Multiple_Complex
TC0800012308.hg.1	6.43	6.9	-1.39	0.0026	0.0496	ADHFE1; C8orf46	alcohol dehydrogenase, iron containing 1; chromoso	Multiple_Complex
TC0600014246.hg.1	5.62	6.41	-1.73	0.0026	0.0499	C6orf229	chromosome 6 open reading frame 229	Multiple_Complex

Supplementary Table 7. Array accession numbers submitted at Gene Expression Omnibus (GEO), NCBI. Bioproject number: PRJNA737074

GSM5374839	S1	Symptomatic
GSM5374840	S2	Symptomatic
GSM5374841	S3	Symptomatic
GSM5374843	S5	Symptomatic
GSM5374844	S6	Symptomatic
GSM5374845	S7	Symptomatic
GSM5374846	S8	Symptomatic
GSM5374847	S9	Symptomatic
GSM5374848	S10	Symptomatic
GSM5374849	S11	Symptomatic
GSM5374850	S12	Asymptomatic
GSM5374851	S13	Asymptomatic
GSM5374852	S14	Asymptomatic
GSM5374853	S15	Asymptomatic
GSM5374854	S16	Asymptomatic
GSM5374855	S17	Asymptomatic
GSM5374856	S18	Asymptomatic
GSM5374857	S19	Asymptomatic
GSM5374858	S20	Asymptomatic
GSM5374859	S21	Asymptomatic
GSM5374860	S22	Asymptomatic
GSM5374861	S23	Asymptomatic
GSM5374862	S24	Asymptomatic
GSM5374863	S25	Asymptomatic
GSM5374864	S26	Asymptomatic
GSM5374865	S27	Asymptomatic
GSM5374866	S28	Asymptomatic
GSM5374867	S29	Asymptomatic
GSM5374868	S30	Uninfected controls
GSM5374869	S31	Uninfected controls
GSM5374870	S32	Uninfected controls
GSM5374871	S33	Uninfected controls
GSM5374872	S34	Uninfected controls
GSM5374873	S35	Uninfected controls
GSM5374874	S36	Uninfected controls

GSM5374875	S37 Uninfected controls
GSM5374876	S38 Uninfected controls
GSM5374877	S39 Uninfected controls
GSM5374878	S40 Uninfected controls
GSM5374879	S41 Uninfected controls
GSM5374880	S42 Uninfected controls
GSM5374881	S43 Uninfected controls
GSM5374882	S44 Uninfected controls
GSM5374883	S45 Uninfected controls
GSM5374884	S46 Uninfected controls
GSM5374885	S47 Uninfected controls
